# The scenicness of historic buildings rivals that of natural features: evidence from crowdsourced photographs of English urban areas

**DOI:** 10.3389/fpsyg.2025.1645424

**Published:** 2026-01-05

**Authors:** Sidney Sherborne, Eugene Malthouse

**Affiliations:** 1 Department of Psychology, University of Warwick, Coventry, United Kingdom; 2 Centre for Decision Research and Experimental Economics, University of Nottingham, Nottingham, United Kingdom

**Keywords:** scenicness, historic buildings, urban areas, natural beauty, listed buildings

## Abstract

Spending time in more scenic areas is associated with better health and wellbeing, making it important to understand how scenicness is influenced by environmental features. Urban areas, where natural features have often been replaced by buildings, are generally perceived as less scenic than rural ones. However, historic architecture may enhance urban scenicness. Using data from Historic England and Scenic-or-Not, a crowdsourced platform rating the scenicness of UK photographs, we investigate the relationship between listed buildings (as recorded on the National Heritage List for England) and scenicness. We find that the presence of a listed building in a photograph of an urban scene is associated with a 0.61-point increase in scenicness on a 10-point scale (equivalent to 0.53 standard deviations). This association is comparable to that of forests and lakes and is greater for buildings designated as more significant, listed earlier, and dating from earlier periods. These findings highlight the positive contribution of historic buildings to urban environments and provide empirical support for their continued preservation as public goods.

## 1 Introduction

What do we mean when we describe a place as *scenic*? According to the Cambridge English Dictionary (2025), a place is scenic if it has ‘beautiful natural features’. Consistent with this, photographs of landscapes containing natural features such as rivers and trees are generally rated as more scenic than those without, and urban areas, which often lack such features, are generally judged to be less scenic than rural areas (Seresinhe et al., 2017a,b).

Some urban areas are more scenic than others, however, and this variation matters. Scenicness influences both the decision to visit particular areas (Hanley et al., 2001, 2002; Hynes et al., 2006) and the wellbeing derived from doing so. Being in a more scenic area is associated with greater in-the-moment happiness (Seresinhe et al., 2019), and those who live in more scenic areas report better health (Seresinhe et al., 2015). But while the wellbeing effects of scenicness are found across both rural and urban settings, urban areas face particular challenges in achieving scenic quality. So what makes some urban areas more scenic than others? As cities become home to a greater proportion of the world’s population, finding answers to this question is increasingly important.

Prior research suggests two main answers. The first relates to incorporating natural features, with studies finding that urban environments containing certain natural elements—such as trees, gardens, and ponds—are judged as more scenic (Seresinhe et al., 2017b). The second answer relates to buildings and architecture, with certain building types showing strong positive associations with scenicness. Specifically, Seresinhe et al. (2017b) found that churches, castles, cottages and viaducts—structures that are typically, though not exclusively, historic in nature—were associated with greater scenicness, while modern buildings such as industrial sites, hospitals and parking garages were associated with lower scenicness. Relatedly, Liang et al. (2024) find that perceiving a building as being more historic is associated with judging its surrounding streetscape being more beautiful, though they did not directly measure scenicness. This evidence suggests that historic aspects of the built environment may confer scenicness to urban areas in a way similar to natural features and thereby function as public goods that enhance societal wellbeing (Serageldin, 1999).

These findings about urban scenicness emerge from a growing body of research that has developed robust methods for measuring scenic quality. Early studies measured scenicness on 4-point Likert scales from ‘not scenic’ to ‘very scenic’ (e.g., Brown, 2003; Hanley et al., 2001). More recent studies use data from Scenic-or-Not which asks participants to rate the scenicness of photographs of the United Kingdom on a 1 to 10 scale with 1 labelled as ‘not scenic’ and 10 labelled as ‘very scenic’. These ratings have been validated against several measures of naturalness and aesthetic quality. For example, photographs uploaded to the social media platform Flickr are more likely to be described as ‘scenic’ (or words with similar semantic meaning, such as ‘bucolic’, ‘rustic’ and ‘picturesque’) if taken near to Geograph photographs given higher scores on Scenic-or-Not (Seresinhe et al., 2017a); urban areas have lower average scenicness compared to rural areas (Seresinhe et al., 2017a,b); greater scenicness is associated with perceptions of naturalness, being rugged, and containing fewer human artifacts (Chang Chien et al., 2020); and machine learning studies consistently find that words describing natural features are most positively associated with scenicness and those describing the urban built environment are among those most negatively associated with scenicness (Arendsen et al., 2020; Chesnokova et al., 2017; Havinga et al., 2021). These ratings also predict the wellbeing outcomes discussed earlier, with higher scenicness associated with greater happiness and better health (Seresinhe et al., 2015, 2019). These studies demonstrate that scenicness reliably captures something meaningful about environments. However, the construct itself warrants theoretical consideration. Though the word ‘scenic’ implies natural beauty, scenicness could encompass multiple more established psychological constructs: perceived naturalness (Lamb and Purcell, 1990), perceived restorative potential (Herzog et al., 2002), and aesthetic preference (Ulrich, 1983). So while scenicness possesses predictive utility for wellbeing, and is worthy of study, there is necessarily some interpretive complexity about the specific qualities that drive ratings.

The possibility that both natural and historic environments are conducive for scenicness and wellbeing is supported by research into environment types that are restorative for humans. Extensive evidence demonstrates that engaging with natural beauty is restorative for human wellbeing (Capaldi et al., 2017; Gu et al., 2023; Richardson and McEwan, 2018; Zhang et al., 2014a), as is exposure to greenspaces (e.g., forests and parks) and bluespaces (e.g., lakes and rivers) (Berman et al., 2008; Corazon et al., 2019; Gaekwad et al., 2023; Mostajeran et al., 2021; Mygind et al., 2019; Tillmann et al., 2018; White et al., 2021). Natural environments are generally experienced as more restorative than urban environments (Menardo et al., 2021), as are urban areas containing more natural features (Hernández and Hidalgo, 2005; Kaplan, 1983; Lindal and Hartig, 2015; Lohr and Pearson-Mims, 2006; White and Gatersleben, 2011). Yet historic built environments can also offer restorative effects comparable to those of greenspaces and bluespaces (Bornioli et al., 2018; Macdonald et al., 2023; Reece et al., 2022b; Scopelliti et al., 2019). Notably, recent studies have found that some historic built environments may be even more restorative than natural spaces (Reece et al., 2022a); and that architectural characteristics can have a significantly greater impact on the perceived restorativeness of urban environments than vegetation (Lindal and Hartig, 2015). Traditional architectural styles, specifically, are evaluated as more relaxing than contemporary styles (Mouratidis and Hassan, 2020). Collectively, these findings suggest that the architectural attributes of historic areas may substantially contribute to their restorative capacities.

Given the established relationship between historic environments and wellbeing, and initial results linking higher levels of scenicness to historic building types, scenic quality may serve as a key mechanism through which historic buildings achieve their restorative effects. In other words, if historic buildings enhance scenicness, and scenic environments are known to promote wellbeing, then scenicness may represent the mediating mechanism connecting historic architecture to psychological restoration.

Why might historic buildings be judged as scenic, and why are they restorative? One explanation relates to the degree to which their architectural features successfully emulate nature, or more technically, how ‘biomimetic’ their architecture is (Aboulnaga and Helmy, 2022). It is suggested that via biomimesis historic buildings can increase people’s connection to nature (Richardson and Butler, 2021) and potentially yield restorative effects for their wellbeing (Lavdas and Schirpke, 2020; Van den Berg et al., 2016; Zhong et al., 2022). Biomimetic features can evoke nature in a range of ways, from employing design principles and mathematical relationships found in nature, to the use of natural materials, patterns and colours—all of which tend to be more common in historic than modern buildings (Arslan and Sorguc, 2004; Ramzy, 2015a,b). According to this theory, architects of historic buildings designed urban environments to be like natural ones, potentially due to an intuitive understanding of the restorative effects of nature (Hayles and Aranda-Mena, 2018). Evidence for this theory indicates that, even within the architectural and urban contexts, low-level visual features are systematically associated with perceived naturalness (Berman et al., 2014; Coburn et al., 2019; Ibarra et al., 2017; Kardan et al., 2015), and that the presence of naturalistic patterns in architecture predicts aesthetic preference (Coburn et al., 2019). However, these associations weaken when high-level scene properties, such as objects and semantic context, are removed (Menzel and Reese, 2022), with high-level features proving stronger predictors of both aesthetic preference and perceived naturalness than low-level characteristics alone (Ibarra et al., 2017). Additionally, eye-tracking studies show that historic buildings sharing visual properties with nature attract the gaze and hold it for longer than modern buildings lacking such features (Rosas et al., 2023).

Beyond biomimesis, there are other possible explanations for the scenicness and restorativeness of historic buildings. Historic buildings differ from modern structures in several ways that may enhance scenicness independently of any biomimetic qualities. For example, historic buildings typically feature greater ornamentation (Elrayies, 2018), different architectural styles (Nasar, 1989), and were built by different methods (Vagtholm et al., 2023) in comparison to modern buildings. Another possible explanation is that aesthetic preferences, including those for architecture, are products of education and social conditioning (Bourdieu, 2017), suggesting that our appreciation of historic buildings could partly reflect learned associations with heritage, permanence, and cultural value. These potential mechanisms are not mutually exclusive and may work together to enhance the perceived scenicness of historic architecture.

So, while the current evidence hints at a broader relationship between historic architecture and scenicness, no research to date has directly tested whether the presence of officially designated historic buildings is associated with an increase in urban scenicness. The present study addresses this gap, assessing whether representative photographs of English urban areas are rated as more scenic when they feature historic buildings—and, if so, by how much?

In England, buildings with special historic or architectural value are comprehensively ‘listed’ on the National Heritage List for England (NHLE) by Historic England (Department for Digital, Culture, Media, and Sport, 2018). To investigate the relationship between historic buildings and local scenicness, we combined NHLE data with scenicness scores from Scenic-or-Not, an online platform where users rate the scenicness of photographs taken across the UK. Specifically, we analysed whether photographs taken in urban areas and containing listed buildings received higher scenicness scores compared to photographs without such buildings. This approach allowed us to quantify the change in urban scenicness associated with the presence of a listed building.

In addition, we explored the marginal effects of more significant listed buildings on scenicness. We defined ‘more significant’ according to two additional NHLE metrics. The first was the grade of the listing buildings, which are all either Grade I (‘exceptional interest’); Grade II* (‘particularly important buildings of more than special interest’); or Grade II (‘special interest’). The second was the year in which the buildings were listed by Historic England, ranging from 1949 to the present day. We take this as indicative of prioritisation, with more significant buildings likely being listed at an earlier date. We predicted that photographs featuring buildings of greater significance—i.e., those with a higher grade and those listed earlier—would be associated with a greater increase in urban scenicness.

Finally, we examined the effect of building age on scenicness, using estimated construction dates recorded by Historic England. Buildings were grouped by century, allowing us to test whether older structures were associated with greater scenicness than more modern listed buildings.

This approach allowed us to assess whether, and to what extent, the presence, significance, and age of historic buildings are associated with the scenicness of English urban areas.

## 2 Materials and methods

### 2.1 Photographs with scenicness ratings

The main data for our analysis comes from Scenic-or-Not.^1^ Scenic-or-Not presents participants with a random photograph from Geograph.^2^ Geograph “aims to collect geographically representative photographs and information for every square kilometre of Great Britain and Ireland.” These photographs are crowd-sourced following moderation guidelines that emphasise documentary accuracy over aesthetic appeal. To determine what photographs are appropriate, contributors are prompted to “think what a child looking at a map in a geography lesson might find useful when trying to make sense of what the human and physical geographical features in a given grid square actually look like,” or what they would see if they “looked further afield from a given viewpoint.” Indicative subjects are also given, namely: physical landscape; human land-use; built environment; social interaction; geology; flora and fauna and historical interest. Each photo must “clearly show one of the main geographical features within the square, showing enough of the surroundings to provide some context” and photographs of buildings should “show all of it (or a significant part of a very large building); it should also show some of its surroundings, usually what is in front, preferably more.” Photographs are moderated according to these stipulations and additional guidelines (see https://www.geograph.org.uk/article/Geograph-or-supplemental for details of these).

Participants rate one of these photographs at a time on a 1–10 integer scale where 1 corresponds with “not scenic” and 10 corresponds with “very scenic.” Only photographs that received more than three ratings were included in the dataset. The complete dataset contains the results of 1,568,176 ratings for 212,212 photographs taken in the United Kingdom. These ratings are the only information provided by participants; no demographic data are collected.

### 2.2 Shapefile for England

The shapefile for England is provided by Eurostat’s Geographical information system of the Commission service.^3^ 125,359 of the Scenic-or-Not photographs are located in England.

### 2.3 Land cover data

We used data from the UK Land Cover Map 2007 to identify which photographs from Scenic-or-Not are located in urban areas.^4^ Data from several years were available. To determine which year to use, we randomly sampled 100 photographs from the complete Scenic-or-Not dataset. The mean year of these photographs was calculated as 2006.68. We therefore decided to use data from 2007 as this was much closer to the mean than the alternatives from 2000 or 2015. The resolution of the raster data was 25 metres. This data distinguishes between urban and suburban areas. The map’s documentation states that the urban classification is used for “dense urban [areas], such as town and city centres, where there is typically little vegetation, [and] areas such as dock sides, car parks and industrial estates.” The suburban classification is used for “suburban areas where the spectral signature is a mix of urban and vegetation signatures.” We used only photographs taken within areas classified as ‘urban’ to limit, as far as is possible, the presence of vegetation.

In our analysis we only used photographs geolocated within English urban areas. This subset contains 28,547 ratings of 3,843 photographs.

### 2.4 Listed buildings

Historic England state that the older a building is the more likely it is to be listed (Department for Digital, Culture, Media, and Sport, 2018). All buildings built before 1700 are listed and so are most of those built between 1700 and 1850. There is then greater selection for buildings constructed between 1850 and 1945; “careful selection” for those built after 1945; and buildings less than 30 years old are not normally considered as “they have yet to stand the test of time.” Where selectivity is required, a building’s addition on the list is based on its aesthetic merits, architectural interest, and national or regional significance. In total, there are more than 370,000 listed buildings in the country, all of which are legally protected against redevelopment for the benefit of present and future generations.

Data on the location of English buildings legally recognised for their historical or architectural value is provided by Historic England.^5^ The data we used contains 379,280 listings. In addition to the location of the buildings, the data also contains the grade of the listing (I, II* or II) and the date upon which the entry was created. The period in which the building was constructed is usually also provided; no date was provided for two listed buildings. Where a range of centuries was given we used the earliest date provided.

Grade I buildings (of ‘exceptional interest’; 2.5% of all listings) include major churches, cathedrals, castles, and palaces of exceptional architectural quality or historical importance. Grade II* buildings (‘particularly important buildings of more than special interest’; 5.8% of listings) are those that stand out for their architectural sophistication, intactness, or historical associations—often including manor houses, significant civic buildings, and architecturally distinguished churches. Grade II buildings (‘special interest’; 91.7% of listings) encompass the majority of listed structures and include architecturally interesting residential buildings, minor churches, and locally significant structures. Generally, Grade I and II* buildings tend to be larger in scale, more architecturally elaborate, and more likely to be landmark structures that dominate their surroundings, while Grade II buildings are more typically integrated into the street scene. This hierarchy thus captures not only heritage value but also differences in visual prominence and architectural complexity that could influence scenicness ratings.

### 2.5 Identifying photographs containing listed buildings

We used the Land Cover Data and England shapefile to identify the Scenic-or-Not photographs that were taken in urban English areas. We then used QGIS via the qgis R package to draw a 100 metre radius around the geolocated point of each Scenic-or-Not photograph. We then identified which of these areas contain at least one listed building. This yielded a list of 749 photographs. We manually checked each of these photographs against the Historic England database to see if a listed building featured in the photograph. We followed a conservative approach designed to maximise objectivity: if a listed building was featured, even if only marginally—for example, if it was obscured, if only a small part of it was visible, or if it was only visible in the distance—its list entry number was recorded. If more than one listed building featured in the photograph then the list entry of the most prominent listed building was recorded. If no listed building featured in the photo, or no listed buildings were located within the 100 metre radius of the photo, then it was tagged as not containing a listed building. For robustness, the prominence of the main listed building in the photograph was coded as either ‘only building (main subject)’ (40%), ‘only building (not main subject)’ (2%),‘one among many listed’ (11%), ‘one among mixture [of listed and non-listed buildings]’ (11%), ‘one among other [non-listed] buildings’ (20%), or ‘marginal/obscured’ (16%; see Supplementary materials for additional results based on these categorisations). All manual checks using these criteria were conducted blindly by the authors, meaning that photograph scenicness scores were not visible to either of them during this process. To assess inter-rater reliability, a random subset of 50 images coded by the first author was independently examined by the second author. In total, 48 of 50 (96%) of images were coded identically; the two discrepancies were for photographs featuring a marginal or obscured listed building identified by the first author, suggesting that these buildings were not salient features of their respective photographs.

This process split the dataset of photographs into two: one set of 358 photographs that contained at least one listed building; and another set of 3,487 photographs that did not contain a listed building. As an example, Table 1 contains the photographs rated as the most and the least scenic for each of these sets.

**Table 1:** The first column indicates whether the most or least scenic photographs in the category are displayed.

Scenic	LB	Rank 1	Rank 2	Rank 3
Most	Yes	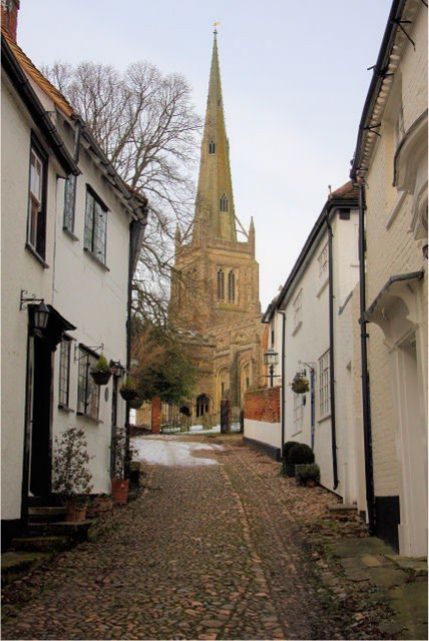 7.20	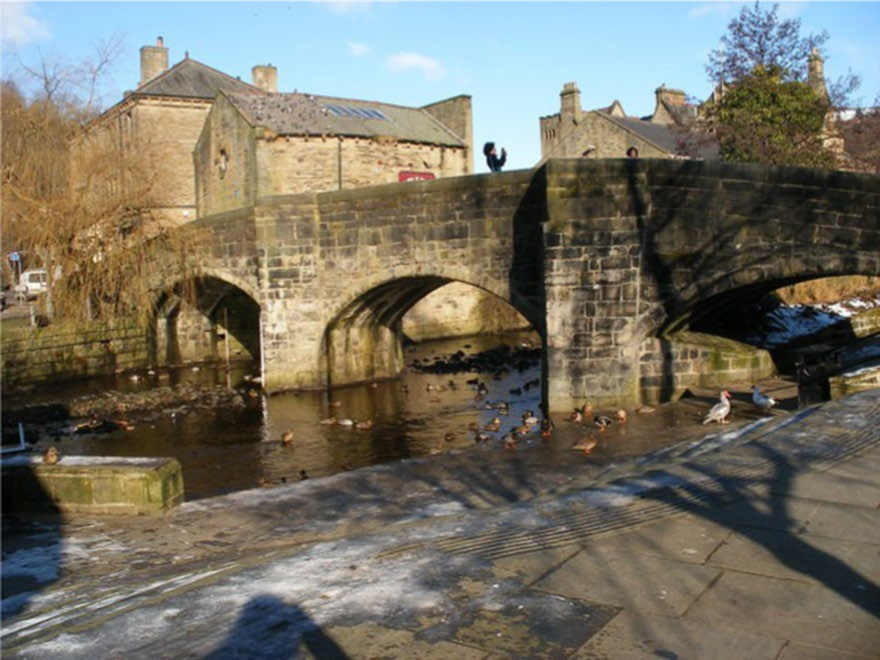 7.20	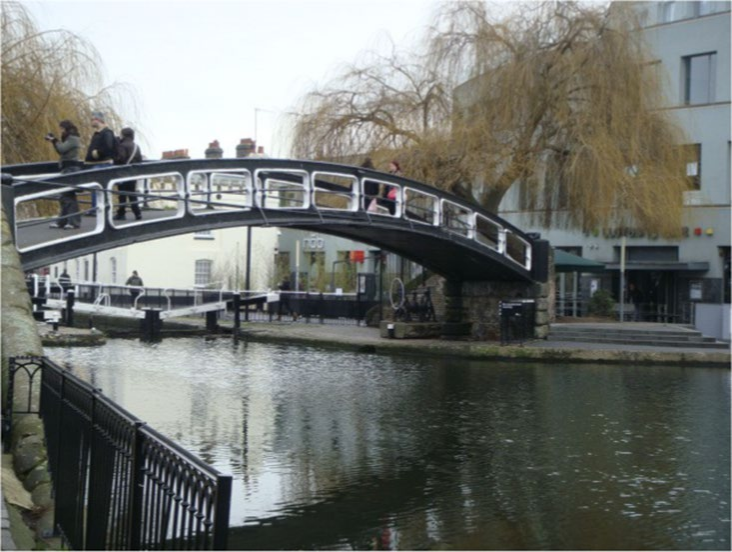 7.17
Most	No	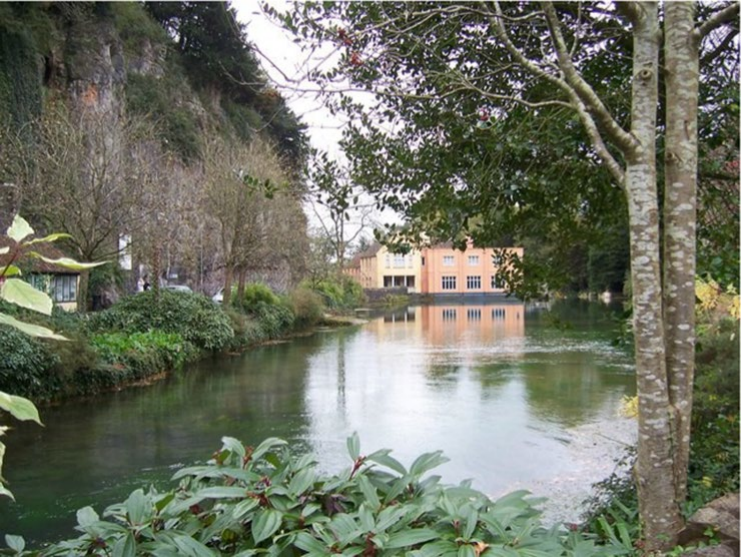 7.00	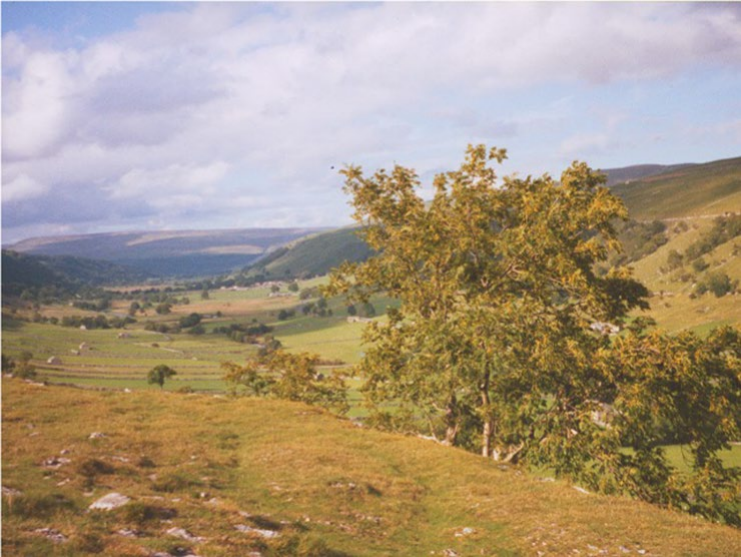 6.83	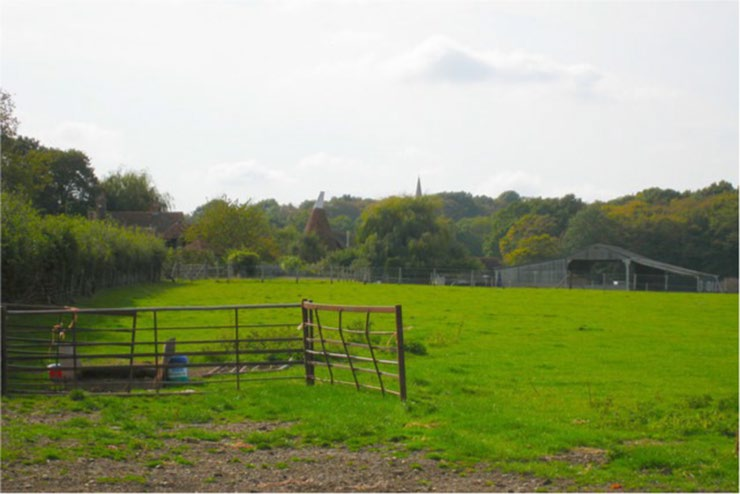 6.6
Least	Yes	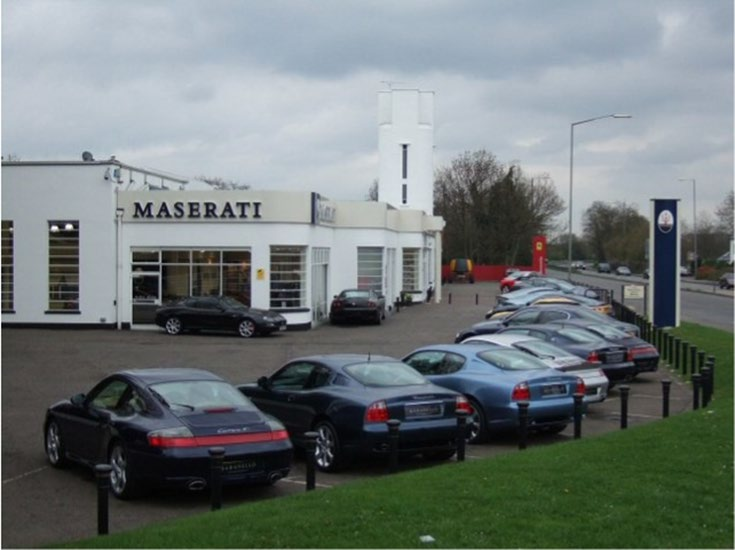 1.00	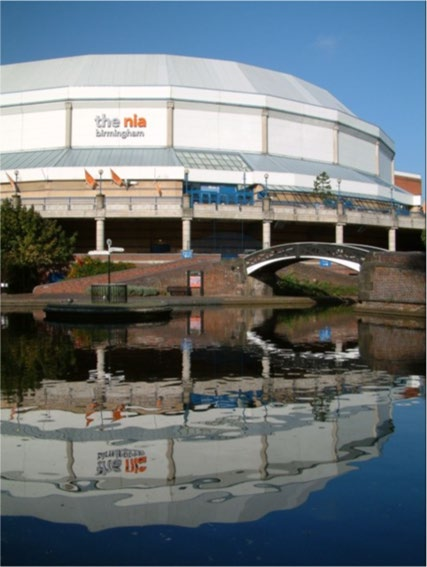 1.00	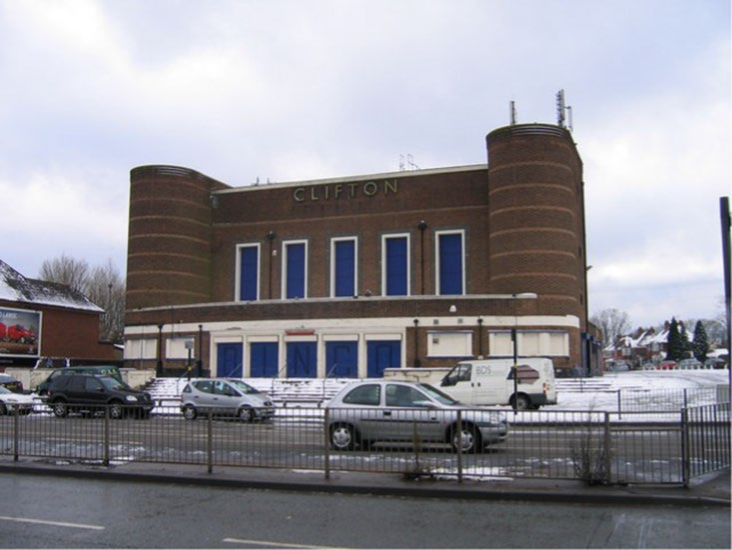 1.00
Least	No	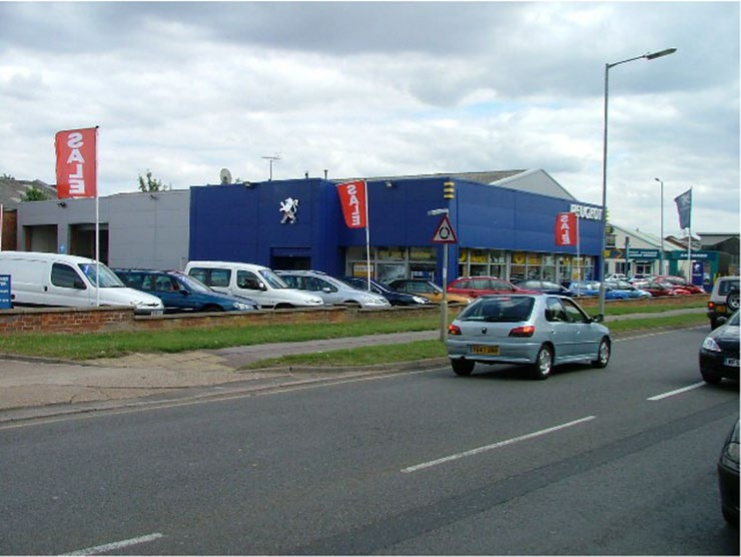 1.00	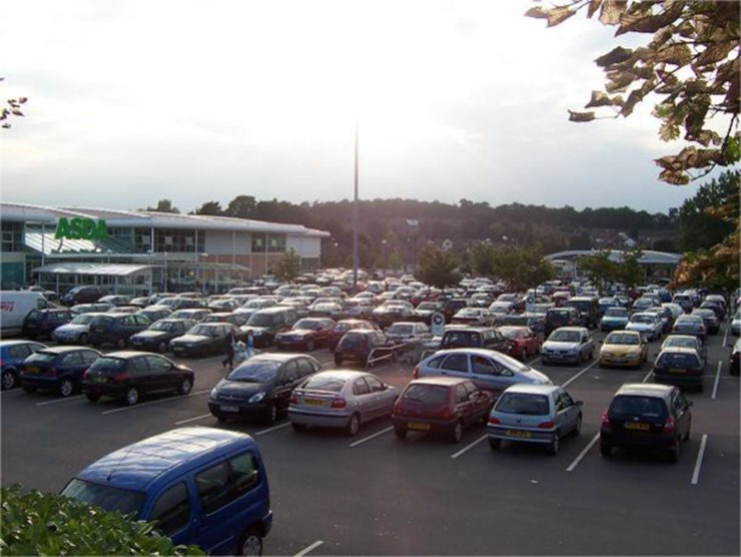 1.00	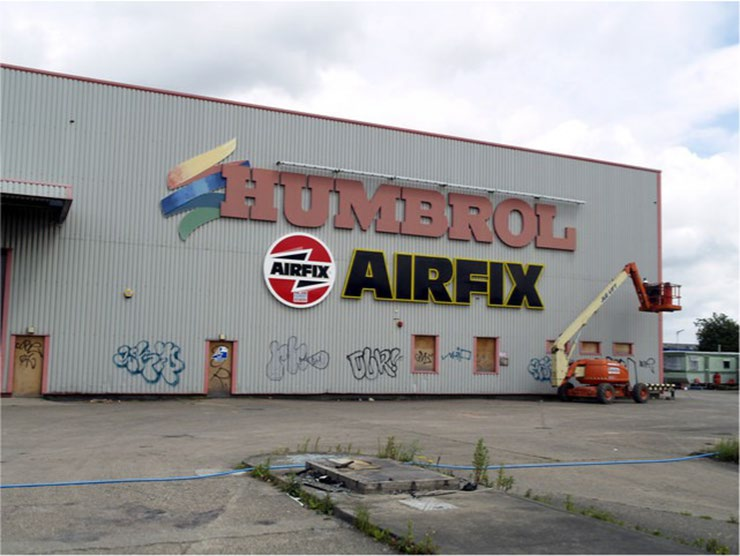 1.00

The second column (LB; Listed Building) indicates whether the presented photographs contain a listed building or not. The first two rows detail the three most scenic photographs in each category, where Rank 1 refers to the most scenic photograph, Rank 2 refers to the second most scenic photograph and so on. The third row contains all photographs containing listed buildings with an average scenicness of 1.00. The bottom row contains a sample of photographs without a listed building with an average scenicness of 1.00. Photo credits, left-to-right top-to-bottom: © Bob Jones; © SMJ; © Sheila Madhvani; © Pam Goodey; © Stephen Craven; © Oast House Archive; © Martyn Davies; © Graham Taylor; © Roy Hughes; © Robin Hall; © Adrian Bailey; © Andy Beecroft (https://creativecommons.org/licenses/by-sa/2.0/).

### 2.6 Google vision

**Figure 1:**
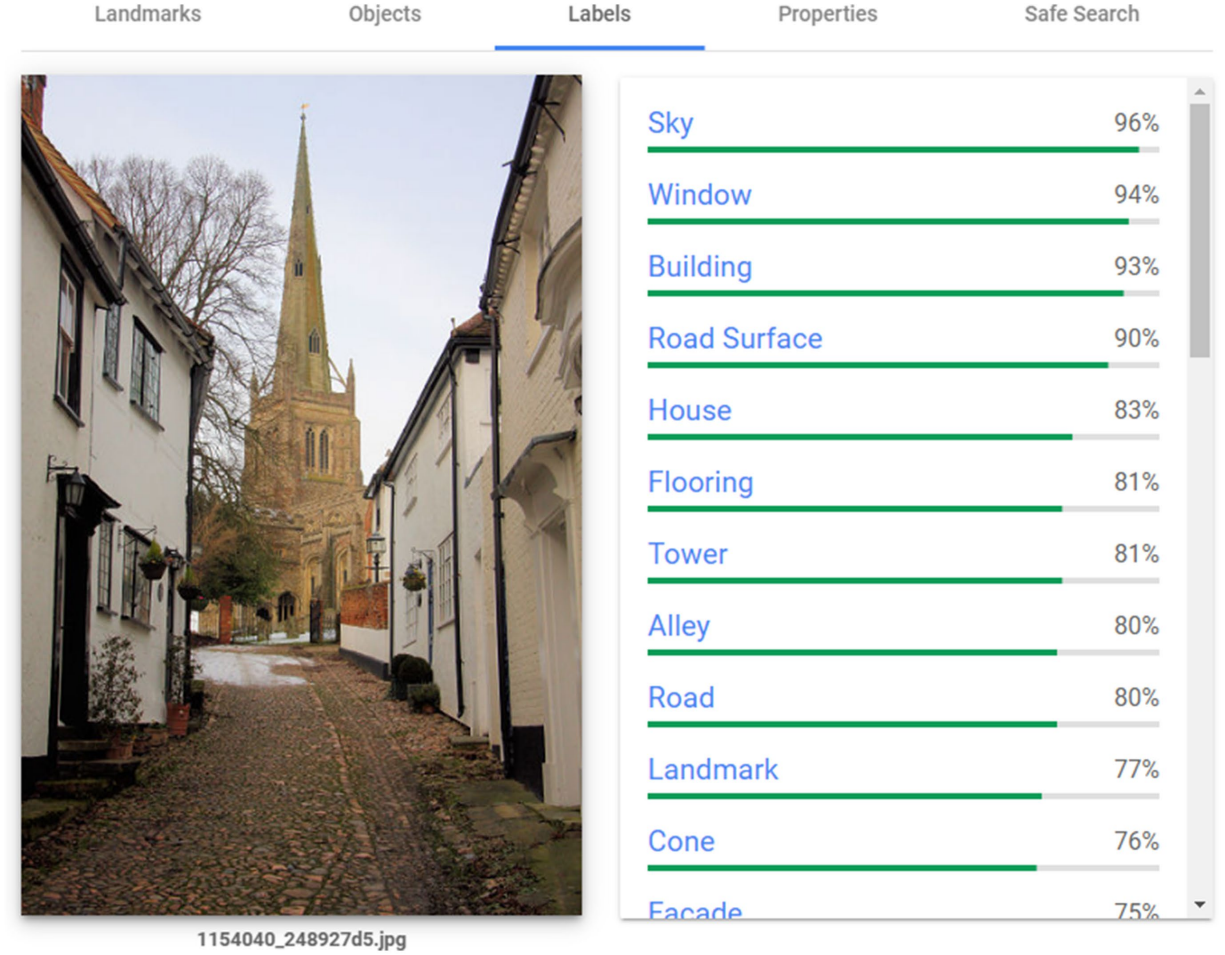
Top Google Vision labels for an example photo. Geograph photograph © Bob Jones (cc-by-sa/2.0).

In order to determine other features of note in the Geograph photograph used by Scenic-or-Not, we utilised Google Vision’s label detection feature.^6^ This tool extracts information about entities within an image and provides a confidence score for each of those entities. A partial example of the output of this process is presented in Figure 1. The total list of labels Google Vision is capable of assigning is not publicly available; for our data, the tool yielded 1,094 distinct labels (using Google’s default confidence cutoff of 0.5). Existing research using Google Vision has taken 0.6 as a confidence cutoff for labels (Gosal et al., 2019; Lee and Son, 2023) and we too use this value. This left 1,020 distinct labels. Some of these labels occurred a small number of times across the photographs. We therefore restricted the labels to those that were found for 30 or more different photographs; this left distinct labels. Two labels were dropped due to their content being similar to that of listed buildings, namely “medieval architecture” and “history.” A total of 25 labels were dropped for signifying particular architectural features. Nine further labels were dropped due to having a correlation of greater than 0.8 with another label (see Supplementary material for details). This left 174 labels. A sample of nine random photographs for each of these labels can be found in the Supplementary material. A dummy variable was created for each label; these indicated whether Google Vision identified the corresponding entity in a photograph. This set of dummy variables was included as controls for all regression analyses.

### 2.7 Hypotheses

Our first hypothesis (Hypothesis 1) was that photographs of urban areas containing listed buildings would be judged as more scenic than those that do not contain a listed building. Our second hypothesis (Hypothesis 2) was that more significant listed buildings—either of a higher listing grade, or listed by Historic England at an earlier date—would be judged as being more scenic. Our third hypothesis (Hypothesis 3) was that older listed buildings would be judged as being more scenic.

To test these hypotheses we used the following ordinary least squares regression model (Equation 1):


(1) $$\text{Scenicnes}s_{i}=\alpha +\beta_{1}\text{Liste}d_{i}+\beta_{2}X_{i}+\in_{i}$$

where *Scenicness_i_* is the mean scenicness score for photograph *i*; *Listed_i_* is a dummy variable for if photograph *i* contains one or more listed buildings; and *X_i_* is a vector of dummy variables indicating whether Google Vision identified a particular entity as present in photograph *i*.

For subsequent analyses we replaced *Listed_i_* with sets of dummy variables according to properties of the most prominent listed building in photograph *i*. For Hypothesis 2, this was firstly a set of dummy variable for each listing grade (I, II*, II or not listed); and secondly a set of dummy variables corresponding to four equally sized groups of photographs, grouped according to the year in which the most prominent listed building they contain was added to the list. For Hypothesis 3, this was a set of dummy variables corresponding to the approximate century in which the most prominent listed building was constructed. The reference category in each case was photographs without listed buildings.

## 3 Results

**Figure 2:**
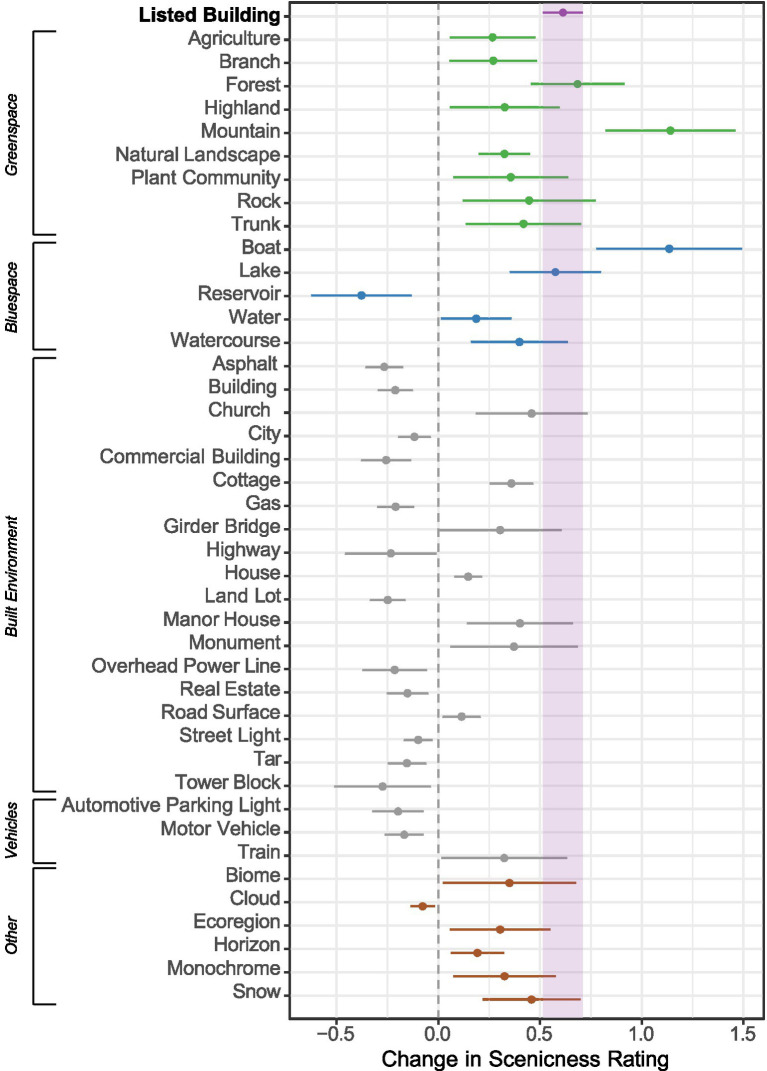
The figure presents the regression coefficients for listed buildings and those entities identified by Google Vision that were statistically significant (*p <* 0.05). Greenspace entities are coloured green; bluespace entities are coloured blue; artificial and vehicle entities are coloured grey; and other entities that did not fit into one of these categories are coloured red. *R*^2^ = 51.5%; Adjusted *R*^2^ = 49.2%; 3,843 observations. The reference category consists of photographs without listed buildings. Full regression results can be found in the Supplementary material.

Our analysis revealed that the mean scenicness rating of photographs taken in urban areas was 2.43, and the standard deviation was 1.16. Figure 2 shows that the presence of at least one listed building in these photographs was associated with a 0.61 point (or 25.2%; or 0.53 standard deviations) increase in scenicness [*β* = 0.61, *CI* = (0.52, 0.71), *p* < 0.001, *N* = 3,843], controlling for the presence of entities identified by Google Vision. Additional tests for coefficient equality showed that this represented an increase in scenicness greater than the increase produced by several greenspace and bluespace entities such as agriculture (*χ*^2^ = 8.64; *p* = 0.003), natural landscape (*χ*^2^ = 12.41; *p* < 0.001), reservoir (χ^2^ = 53.94; *p* < 0.001) and water (*χ*^2^ = 17.88; *p* < 0.001). As can be inferred from Figure 2, the coefficient for listed buildings was not statistically different from those of entities such as forests, highlands, plant community, trunk, lake, and watercourse. Lastly, when Google Vision controls were not included the associated increase in scenicness was 34.7% or 0.72 standard deviations [*β* = 0.85, *CI* = (0.73, 0.97), *p* < 0.001, *N* = 3,843]. These findings support our primary hypothesis that photographs containing listed buildings are more scenic.

A possible explanation for these results is that listed buildings exist in particularly scenic contexts, and that it is these contexts that drive scenicness judgements. To test this we categorised photographs according to the prominence of the listed building(s) in the photograph and found that the more prominent the listed building(s) the higher the scenicness scores for those photographs (see Supplementary material). Notably, we found that photographs containing multiple listed buildings were rated as significantly more scenic [*β* = 0.98, *CI* = (0.71, 1.25), *p* < 0.001, *n* = 3,843] than photographs which contained one listed building among other buildings [*β* = 0.27, *CI* = (0.07, 0.47), *p* = 0.009], where the reference category was photographs with no listed buildings. As a further robustness check, we used propensity score matching to create covariate balance in Google Vision controls between photographs containing listed buildings and those that did not; doing so did not significantly change the scenicness effect associated with the presence of a listed building (see Supplementary material).

**Figure 3:**
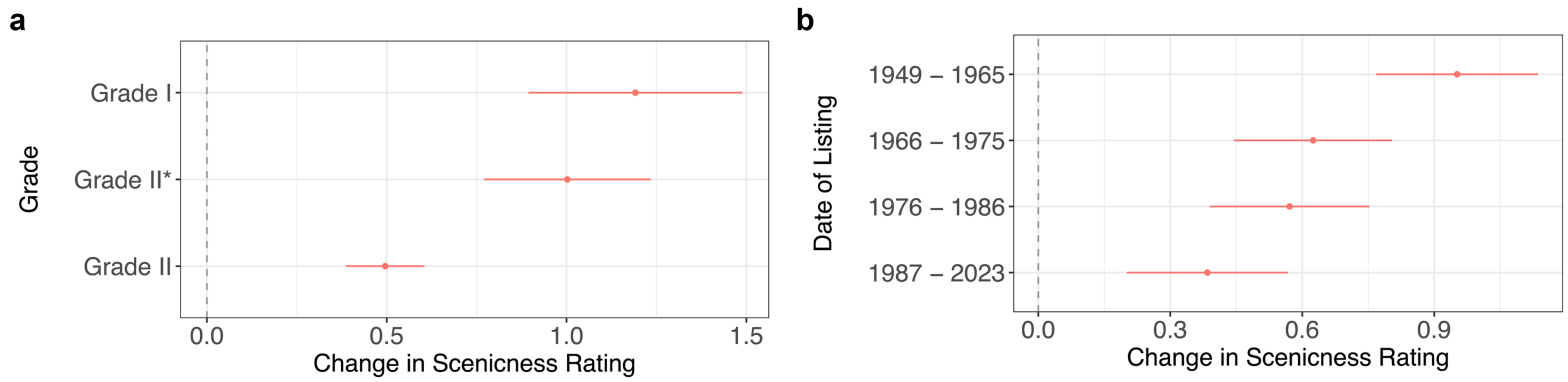
These models investigate the effect of **(a)** the grade of the most prominent listed building (*R*^2^ = 51.9%; Adjusted *R*^2^ = 49.6%) and **(b)** the year in which the most prominent listed building was added to the list (*R*^2^ = 51.8%; Adjusted *R*^2^ = 49.5%). Both of these measures are proxies for the significance of the building in question. In both regression analyses, 3,843 photographs were used. The reference category is no listed buildings present in the photograph. Google Vision controls included.

Our secondary hypotheses were that more significant (Hypothesis 2) and older (Hypothesis 3) listed buildings would be associated with an even greater increase in scenicness. For Hypothesis 2 we first looked at how many of the most prominent listed buildings featuring in the photographs were Grade I (9.2%), Grade II* (15.4%), and Grade II (75.4%) listed. Figure 3a shows that photographs in which the most prominent listed building was Grade II were judged as more scenic than photographs not containing a listed building (*β* = 0.50, *CI* = [0.39, 0.61], *p* < 0.001, *N* = 3,843). This value was not statistically different from that found for all listed buildings in the first analysis. However, photographs where the most prominent listed building was Grade I [*β* = 1.19, *CI* = (0.89, 1.49), *p* < 0.001] or Grade II* (*β* = 1.00, *CI* = [0.77, 1.23], *p* < 0.001) were significantly more scenic than photographs in which the most prominent listed building was Grade II. There was no significant difference between Grade I and Grade II* buildings.

Our second measure of a building’s significance was the time period in which it was listed. Figure 3b shows that photographs in which the most prominent listed building was listed in the earliest period (1949−1965) were judged as significantly more scenic (*β* = 0.95, *CI* = [0.77, 1.14], *p* < 0.001, *N* = 3,843) compared to photographs in which the most prominent listed building was listed in the most recent period (1987−2023; *β* = 0.38, *CI* = [0.20, 0.57], *p* < 0.001), where the reference category was photographs with no listed buildings. The scenicness of photographs in which the most prominent listed building belonged to the intermediate groups were not statistically different from either the earliest or most recent groups. When year of listing was entered in the regression as a continuous variable results indicated that being listed one year later was associated with an 0.012 point decrease in scenicness [*CI* = (−0.02, −0.0037), *p* = 0.005, *N* = 3,843 Google Vision controls included]. In summary, these results provide support for our hypothesis that more significant buildings are associated with greater scenicness (Hypothesis 2).

**Figure 4:**
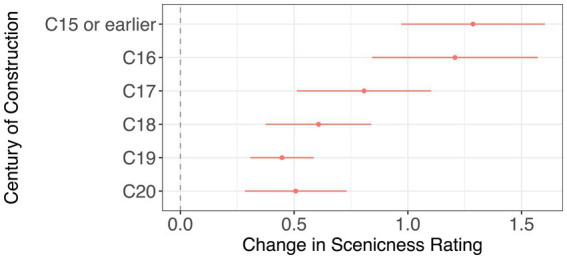
This model investigates whether the approximate date of construction of the most prominent listed building is associated with scenicness. *R*^2^ = 52.0%; Adjusted *R*^2^ = 49.6%; 3,841 photographs. Two photographs were omitted as their date of construction could not be determined. Where a range of dates were given the earliest date was selected. The reference category is no listed buildings present in the photograph. Google Vision controls included.

Our final hypothesis (Hypothesis 3) was that photographs containing older listed buildings would be associated with greater scenicness. Figure 4 indicates that photographs in which the most prominent listed building dates from the 16th century [*β* = 1.29, *CI* = (0.97, 1.60), *p* < 0.001, *N* = 3,841] or 15th century or earlier [*β* = 1.21, *CI* = (0.84, 1.57), *p* < 0.001] were judged as significantly more scenic than photographs where the most prominent listed building dates from the 18th [*β* = 0.61, *CI* = (0.38, 0.84), *p* < 0.001], 19th [*β* = 0.45, *CI* = (0.31, 0.58), *p* < 0.001] and 20th centuries [*β* = 0.51, *CI* = (0.28, 0.73), *p* < 0.001]. This finding is in line with our prediction under Hypothesis 3 and previous research documenting the relationship between scenicness and historic urban environments. It also removes the possibility that the relationship between listing and scenicness is being driven by selection because, as previously mentioned, all buildings built prior to 1700 are listed, as are most of those up to 1850 (Department for Digital, Culture, Media, and Sport, 2018). Under these principles, some buildings may have been listed due to their age alone, potentially despite a lack of architectural significance or scenicness-conferring features.

## 4 Discussion

Is the presence of listed buildings in English urban areas associated with greater scenicness? To investigate this question we combined data from Historic England’s listed buildings registry with scenicness ratings from Scenic-or-Not. We found that the presence of legally recognised historic and architecturally significant (‘listed’) buildings was associated with higher scenicness ratings for photographs of English urban areas. Our study builds upon previous work linking higher scenicness to specific building types such as churches and cottages—structures that are often, though not always, historic (Seresinhe et al., 2017b)—by using formal heritage designations to show that scenicness is specifically associated with legally recognized historic status. Whereas previous studies have related subjective perceptions of historicness to aesthetic judgements (Liang et al., 2024), our results indicate that the presence of officially listed buildings is associated with higher scenicness even when viewers may not recognise them as historic. Specifically, photographs that featured a listed building were rated 0.61 points higher on a 10-point scale than photographs that did not—a 25.2% increase when evaluated at the mean scenicness score. This association is comparable to those of natural features such as forests and lakes. Furthermore, we found that listed buildings constructed furthest into the past exhibited the strongest positive associations with scenicness.

Our second finding was that the significance attributed to historic buildings by Historic England was associated with greater increases in scenicness. That Grade I and Grade II* buildings are associated with larger increases in scenicness than Grade II buildings indicates the degree of interest a building inspires, as judged by regulators, correlates with general judgements of scenic quality. This was supported by our finding that buildings legally recognised for their significance during earlier periods, which we interpreted as an indicator of prioritisation from regulators. However, no differences were found in the scenicness effects of Grade I and Grade II* buildings. These results contribute to the literature that the architectural preferences of experts and the public sometimes align (Gjerde, 2011) and sometimes diverge (Stamps, 1999; Fawcett et al., 2008). A limitation is that these measures of significance are determined by factors in addition to a building’s historic status, such as aesthetic quality, location or prominence. Nonetheless, together these findings constitute evidence that legal designations made by Historic England correlate with scenicness.

While we establish an association between historic buildings and scenicness comparable to that of natural features, our correlational design limits causal inference about underlying mechanisms. The similar associations found for historic buildings and natural features suggest these different environmental aspects may achieve scenicness through shared or distinct pathways that converge on similar perceptual outcomes. A possibility is that there are real visual commonalities between historic architecture and natural features, possibly due to architects taking inspiration from nature (e.g. Ramzy, 2015a,b). However, the similar scenicness associations for historic and natural features could also indicate that scenicness, as measured here, captures something broader than naturalness alone, such as a general aesthetic satisfaction that can be achieved through multiple routes. Thus the architectural differences between historic and modern buildings, such as greater ornamentation and visual complexity, may contribute to scenicness independent of any biomimetic qualities. Non-architectural differences between historic and modern buildings, such as the effects of age on building materials, could also play a role (Wells, 2017). Additionally, historic buildings, like natural features, may provide psychological distance from everyday experiences (Scopelliti et al., 2019) or signal opportunities for prospect or refuge (Akcelik et al., 2024; Dosen and Ostwald, 2016) which in turn drive scenicness ratings. Finally, cultural conditioning may influence aesthetic preferences for both historic architecture and nature (Bourdieu, 2017). Future experimental research manipulating specific architectural features could help disentangle these competing explanations. It should be noted that our findings may reflect survivorship bias, where surviving historic buildings may have been preserved precisely because they are particularly scenic. This possibility could be studied using images of now-demolished buildings from different historic periods.

Our findings are particularly relevant to urban planning. Designing attractive environments where people want to spend time is a key strategy for encouraging specific behaviours in particular locations (Creutzig and Reisch, 2024). More scenic areas are associated with people sharing greater numbers of photographs of that area online (Seresinhe et al., 2017a) and this may be useful for information dissemination and civic participation. In other contexts, engagement with natural beauty is associated with pro-environmental behaviours (Barrows et al., 2022; Diessner et al., 2022; Diessner and Niemiec, 2023), greater pro-sociality (Zhang et al., 2014b), increased cooperation (Kirkland et al., 2024), and enhanced cognitive functioning (Joye et al., 2022, 2024); and scenic historic environments may engender similar behaviours. Future research could: a) test this by conducting behavioural experiments to examine how everyday exposure to historic buildings, natural features, and scenicness relates to cooperative behaviour; and b) integrate these factors into existing interdisciplinary frameworks of cooperation (e.g., Gächter et al., 2017; 2025). Furthermore, given established links between higher scenicness and improved mental health and happiness (Seresinhe et al., 2015, 2019), our findings suggest that policies that promote historic building preservation may have indirect wellbeing benefits for urban populations. Since governments may view the promotion of wellbeing as a policy goal—(arguably even *the* goal; Frijters et al., 2020)—the positive effect of scenicness on wellbeing provides a *prima facie* justification for such policies. As such, the scenic quality of urban environments is a consideration not only for sustainability and productivity behaviours, but also for public health and wellbeing (Coburn et al., 2022). Our findings therefore suggest that policies relating to the preservation of historic buildings should be framed not just as cultural conservation but as a tool for enhancing urban aesthetic quality and wellbeing. More generally, these findings support the characterisation of historic buildings as public goods (Serageldin, 1999) and have implications for policies that encourage the integration of scenic architectural features into new developments (see also Levi, 2005; Mouratidis and Hassan, 2020).

Our analysis has some limitations, however. The first is that our analysis necessarily examines ratings of photographs rather than direct experiences of places, which could have influenced our results. Photographs reflect the photographer’s subjective viewpoint, and it may be that photographers are more motivated to take and submit well-composed, aesthetically pleasing photographs of historic buildings than of modern buildings or streetscapes. Since it was not possible for us to control for these factors, we cannot rule out the possibility that they influenced scenicness ratings. However, Geograph’s emphasis on geographical documentation rather than artistic photography reduces this concern, as photographers are instructed to create representative records rather than aesthetically optimised images. Photographs are also moderated according to these instructions before being accepted and rated. While we cannot entirely eliminate the possibility that photographers exercise more care when documenting historic buildings, our finding that photographs containing multiple listed buildings were rated as significantly more scenic than those containing just one suggests that the buildings themselves, rather than photographic composition alone, are driving the observed effect. This aligns with the “historic ensemble effect” identified in research on house prices, which proposes that clusters of historic buildings can have a collective aesthetic impact greater than the sum of their individual contributions (Lazrak et al., 2014). Future studies could use systematically taken photographs, such as those from Google Street View, to control for photographer intent.

It is also possible that some relevant feature(s) of the photographs were not detected and labelled by Google Vision. Specifically, Google Vision may not reliably identify negative urban elements such as litter, graffiti, poorly maintained surfaces, or temporary visual obstructions that could influence scenicness ratings. Conversely, it may also miss positive aesthetic elements such as fine architectural details, quality of materials, subtle lighting effects, well-maintained plantings, or human activities that add vibrancy to urban scenes. Comprehensive manual coding of all visual elements of thousands of photographs would be unfeasible; as such we cannot definitively determine whether the scenicness effect we observe may reflect: (1) undetected negative elements being less prevalent around listed buildings, (2) undetected positive elements being more common in these areas, or (3) the inherent aesthetic contribution of the listed buildings themselves. Most likely, all three factors contribute to some degree.

A second limitation concerns the construct of scenicness itself. It is possible that users of Scenic-or-Not understood this term in different ways—as a measure of natural beauty, naturalness, architectural quality, restorative potential, or simply overall attractiveness. While this conceptual ambiguity is a valid concern, previous research has shown that scenicness ratings correlate with natural features (Seresinhe et al., 2017a,b) and with wellbeing outcomes such as health, happiness, and other measures of scenic quality (Seresinhe et al., 2015, 2017a, 2019). Our own results also identified expected associations between natural features and higher scenicness. However, it remains unclear whether scenicness represents a single construct or multiple overlapping perceptual processes. Future research could address this by collecting complementary measures—such as perceived naturalness, aesthetic preference, and perceived restorativeness—to test the degree to which these constructs converge or diverge from scenicness.

A third limitation relates to the lack of demographic information about Scenic-or-Not raters. If raters are internationally distributed, scenicness ratings may reflect participants’ local perceptions of beauty or broader, potentially culturally biased, evaluations. Even if raters are largely UK-based, they may not be representative of the national population. Previous research has found that socio-demographic characteristics—such as age and gender—can shape landscape preferences (Li et al., 2021) and perceptions of scenicness (Oku and Fukamachi, 2006). Future studies could address this by collecting demographic data to assess whether the effects observed here hold across populations and subgroups. More broadly, our findings apply specifically to photographs of English urban areas, and to the listed building system used by Historic England. Scenicness preferences may differ in countries with distinct architectural traditions, heritage policies, or cultural associations with historic buildings. Even within England, the impact of listed buildings may vary by regional context, and different associations may be identified for rural and suburban areas. Cross-cultural and regional replication would therefore be a valuable future step in establishing the generalisability of these findings.

Finally, our study is necessarily observational, as architectural and historical attributes cannot be randomly assigned. Despite this, we have taken steps to reduce the risk of spurious associations and improve causal interpretability, including using Google Vision to control for non-historic visual features and applying propensity score matching to address observable confounders. However, as Grosz et al. (2020) caution, observational studies often imply causal relationships without making their assumptions explicit, creating a disconnect between what the data actually show and how findings are interpreted. To mitigate this, we interpret our results as suggestive rather than definitive, and we explicitly acknowledge that our approach rests on the assumption that observable confounders have been adequately controlled. That said unmeasured factors may still influence our results, so residual confounding cannot be ruled out.

Notwithstanding these potential limitations, our results indicate that listed buildings are positively associated with the scenicness of English urban areas. This effect parallels the impact of natural features such as forests and lakes, indicating the substantial contribution of historic and architecturally significant structures to the scenicness of urban environments.

## Data Availability

This study used publicly available data from Historic England (https://historicengland.org.uk/listing/the-list/data-downloads/) and Scenic-or-Not (http://scenicornot.datasciencelab.co.uk/). Labelled data used for analysis are available at https://osf.io/fcvmr/.

## Footnotes

^1^ http://scenicornot.datasciencelab.co.uk/

^2^ http://www.geograph.org.uk

^3^ https://ec.europa.eu/eurostat/web/gisco/geodata/statistical-units/territorial-units-statistics

^4^ https://catalogue.ceh.ac.uk/documents/a1f88807-4826-44bc-994d-a902da5119c2

^5^ https://historicengland.org.uk/listing/the-list/data-downloads/

^6^ https://cloud.google.com/vision/docs/labels
