# Supplementary material for: The scenicness of historic buildings rivals that of natural features: evidence from crowdsourced photographs of English urban areas
Source: Front Psychol. 2026 Jan 5;16:1645424. doi: 10.3389/fpsyg.2025.1645424 (PMC12812532; doi:10.3389/fpsyg.2025.1645424)
Supplement: Supplementary file 1 [file Data_Sheet_1.pdf]

Supplementary Information *

1 Map of Photos from Urban Areas

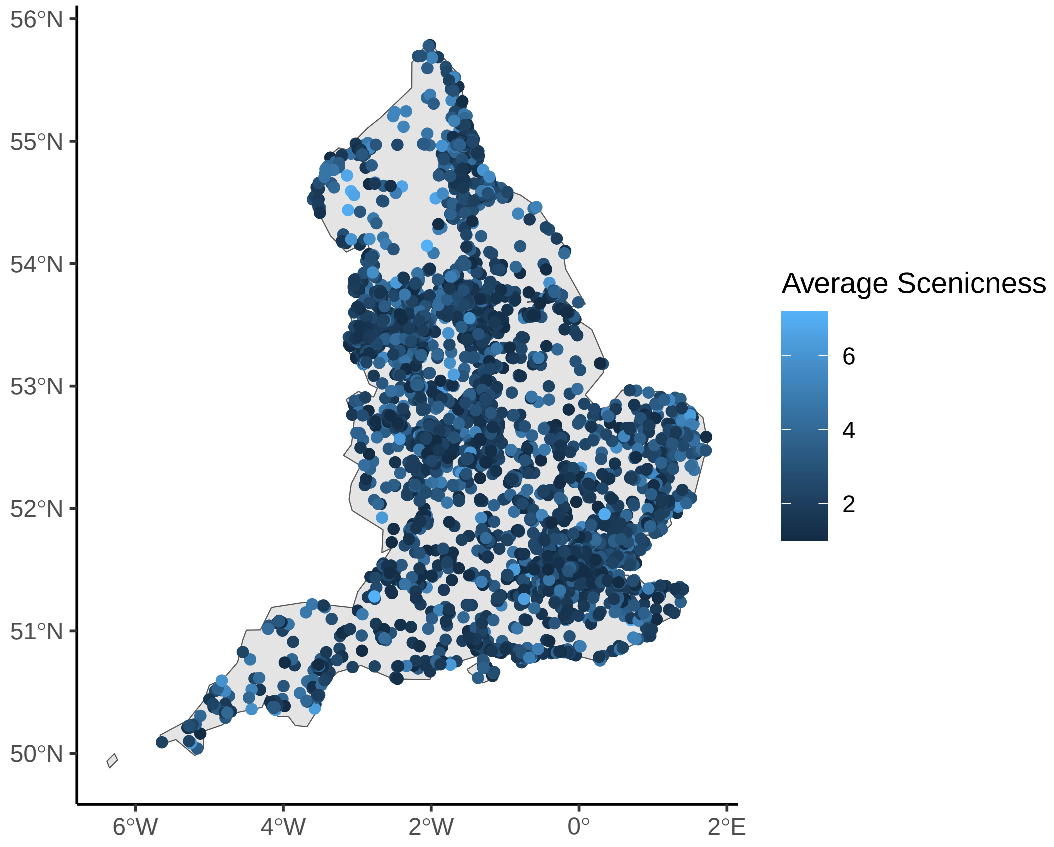

Figure 1: Map of photos.

2 Selection of Google Vision Controls

Two labels were dropped due to being synonymous with historic, namely `history` and `medieval architecture`.

25 labels were dropped due to being architectural features, namely `arch`, `architecture`, `art`, `brick`, `brickwork`, `chimney`, `composite material`, `concrete`, `door`, `facade`, `fence`, `flooring`, `home fencing`, `metal`, `roof`, `sash window`, `siding`, `signage`, `spire`, `steel`, `steeple`, `spire`, `symmetry`, `turret`, `wall` and `window`.

9 labels were dropped due to having a correlation larger than 0.8 with another label. See table below.

*Current version: July 23, 2024.

Variable Kept	Variable Dropped	Correlation
boat	watercraft	0.937
railway	track	0.926
railroad car	locomotive	0.865
locomotive	railroad car	0.865
monochrome	monochrome photography	0.849
tire	wheel	0.844
train	railroad car	0.821
train	locomotive	0.814
watercourse	bank	0.809
railway	rolling stock	0.804
snow	freezing	0.803

3 Photos By Label

Each subsection below contains nine random photographs which have been identified with a certain label by Google Cloud Vision. That label should reflect a common theme contained within each set of photos. Thus, in order to interpret what a particular label means, it may be grasped through the examination of commonalities between its corresponding set of images presented here.

3.1 Listed Building

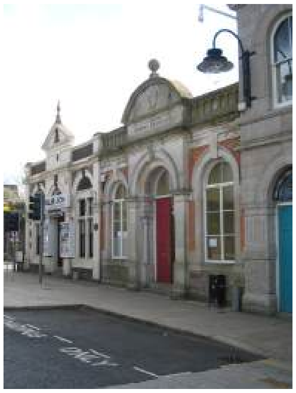

The Mining Exchange Redruth © Rod Allday (cc-by-sa/2.0)

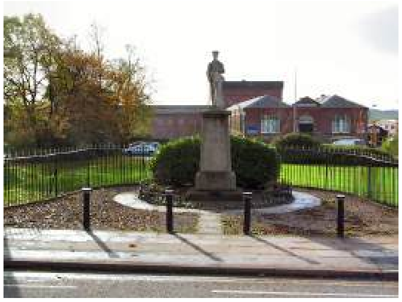

War Memorial at Horwich © Roger May (cc-by-sa/2.0)

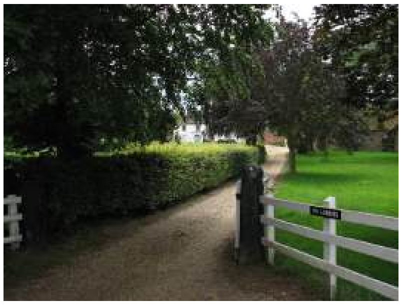

Entrance to Oaks Farm © Evelyn Simak (cc-by-sa/2.0)

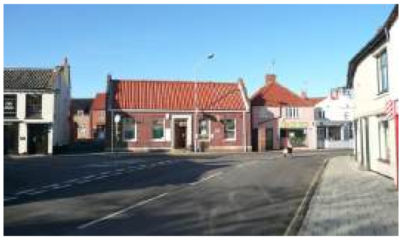

The Post Office, Holt © Humphrey Bolton (cc-by-sa/2.0)

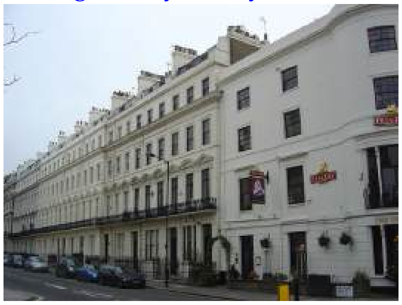

Strathearn Place, London W2 © Steve Millar (cc-by-sa/2.0)

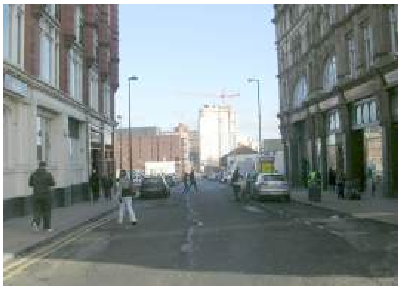

Ludgate Hill - Vicar Lane © Betty Longbottom (cc-by-sa/2.0)

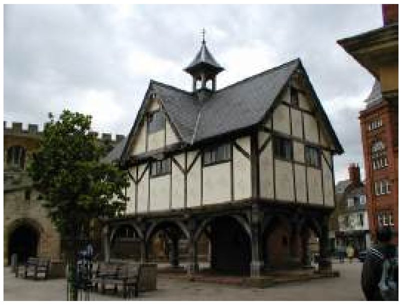

The old Market Place © Row17 (cc-by-sa/2.0)

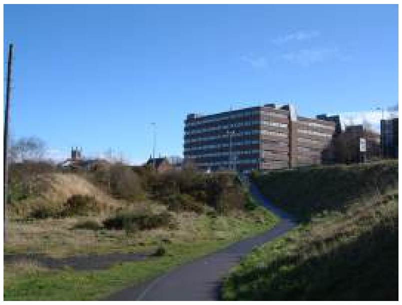

Telecom House, Aldershot © Stacey Harris (cc-by-sa/2.0)

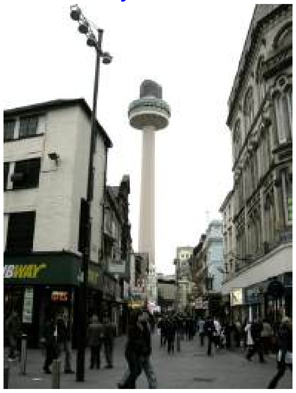

Liverpool-Radio City Tower © Ian Rob (cc-by-sa/2.0)

3.2 Agriculture

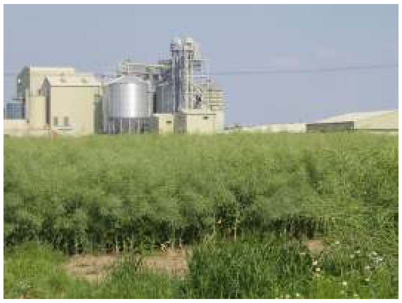

Green Farm © Graham Horn (cc-by-sa/2.0)

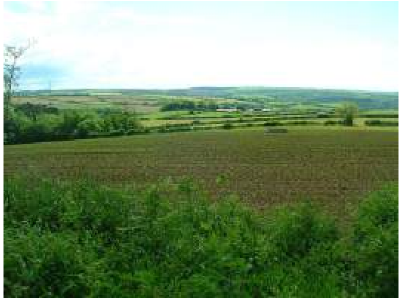

Looking down to Lower Croan © William Bartlett (cc-by-sa/2.0)

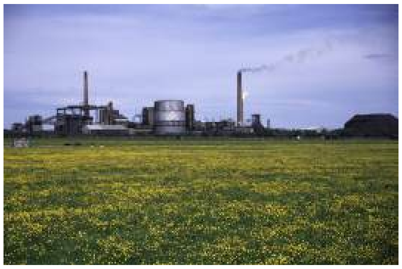

Monkton Coking Works © Chris Allen (cc-by-sa/2.0)

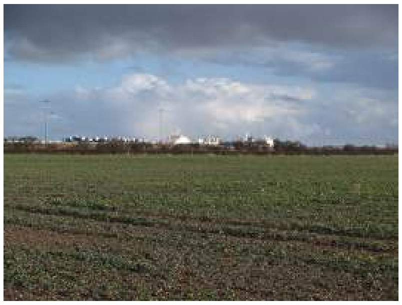

Looking towards the Humber © David Wright (cc-by-sa/2.0)

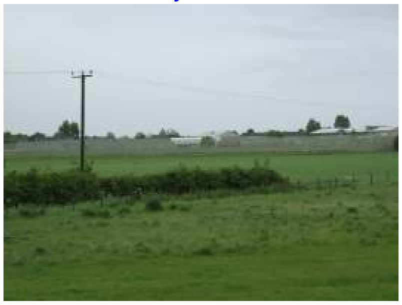

H.M.P. Whatton © Donnylad (cc-by-sa/2.0)

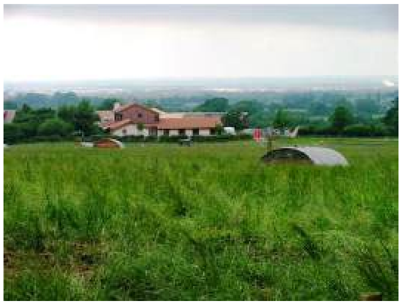

Larchfield Community © Mick Garratt (cc-by-sa/2.0)

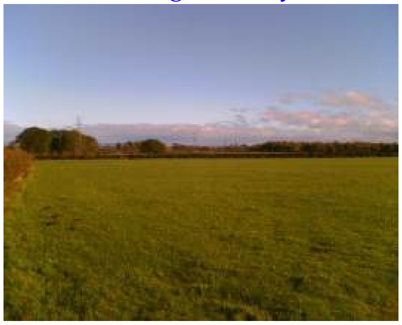

Farmland near Blackhamilton © Adrian Taylor (cc-by-sa/2.0)

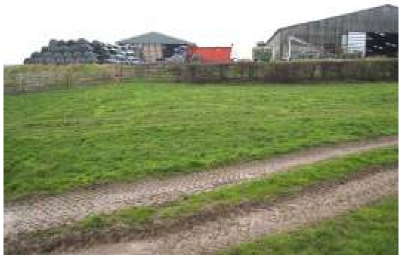

Low Stotfold © Oliver Dixon (cc-by-sa/2.0)

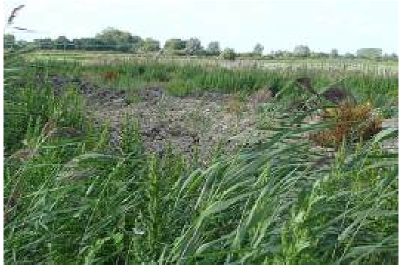

Here be dredgings © Graham Horn (cc-by-sa/2.0)

3.3 Apartment

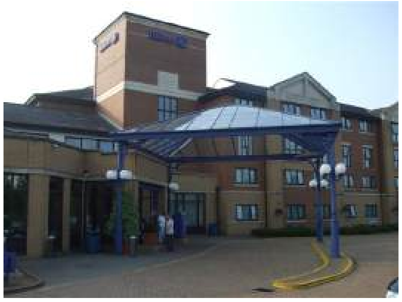

Hilton Hotel, Coventry © Kenneth Allen (cc-by-sa/2.0)

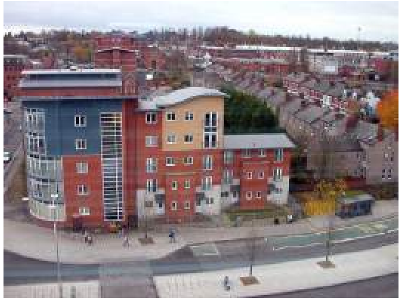

Coventry-Croft Road © Ian Rob (cc-by-sa/2.0)

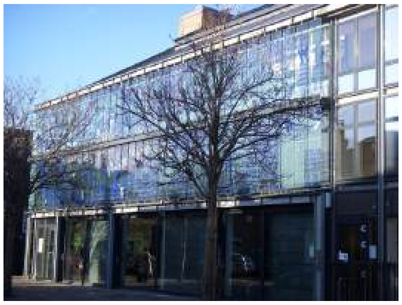

The Place © Freethinker (cc-by-sa/2.0)

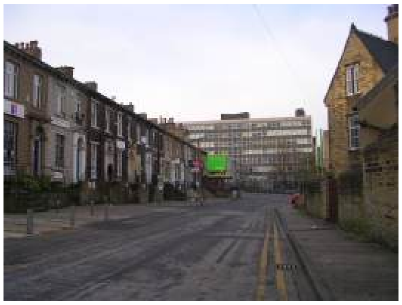

Southbrook Terrace - Morley Street © Betty Longbottom (cc-by-sa/2.0)

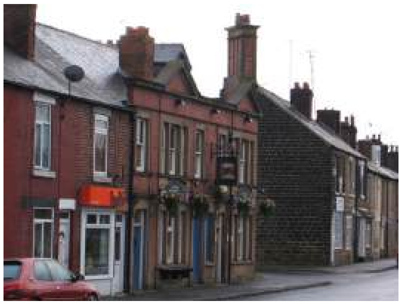

The Commercial, Chapeltown, Sheffield © Chris Whippet (cc-by-sa/2.0)

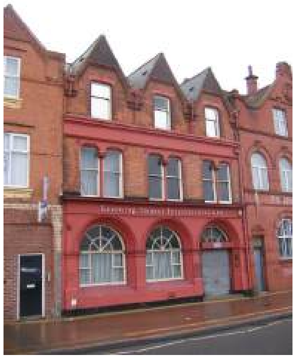

Midland Bank Aston Cross 40-11-02 © Roy Hughes (cc-by-sa/2.0)

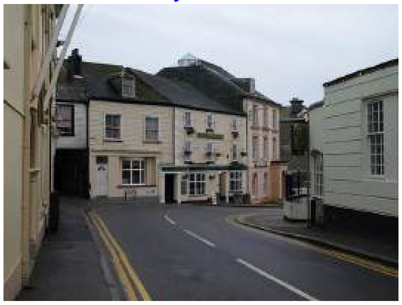

Fountain Hotel, Cornwall © Derek Harper (cc-by-sa/2.0)

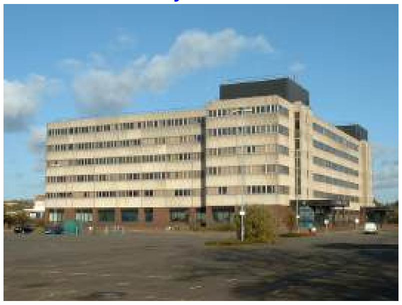

Anzani House © Keith Evans (cc-by-sa/2.0)

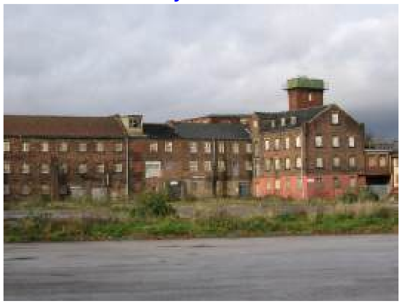

Chesterfield - Robinsons works © Dave Bevis (cc-by-sa/2.0)

3.4 Asphalt

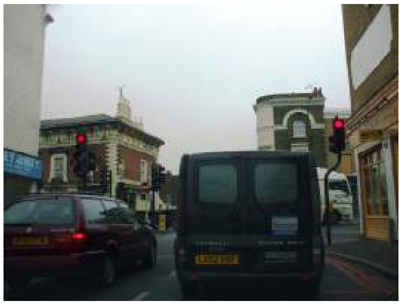

Besson Street, SE14 © Phillip Perry (cc-by-sa/2.0)

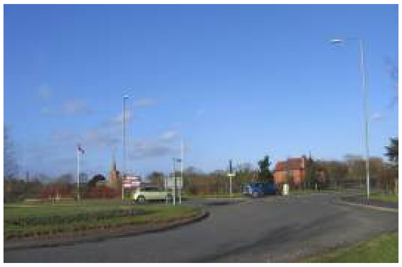

A38 Traffic Island, Upton Warren. © Roy Hughes (cc-by-sa/2.0)

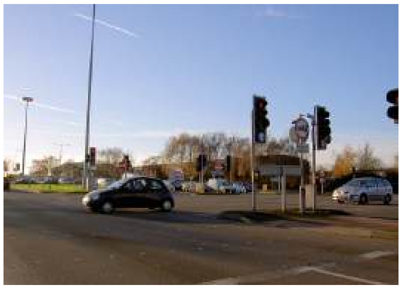

Waiting for the lights © Steve Fareham (cc-by-sa/2.0)

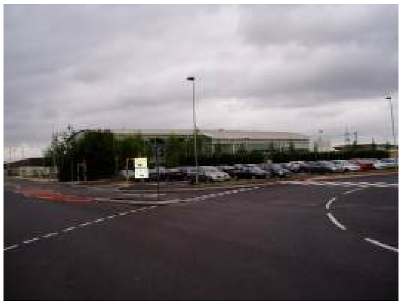

Capenhurst Technology Park © Eirian Evans (cc-by-sa/2.0)

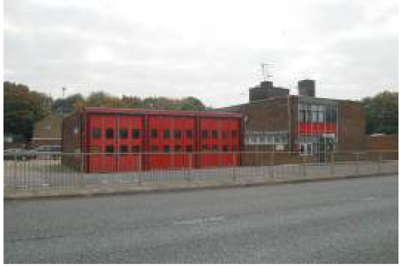

Stevenage fire station © Kevin Hale (cc-by-sa/2.0)

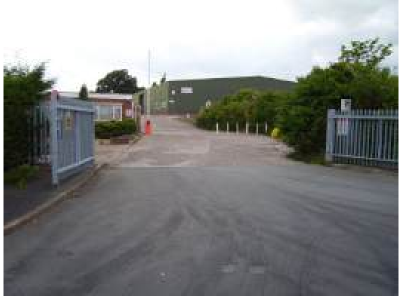

Muller England © Richard Webb (cc-by-sa/2.0)

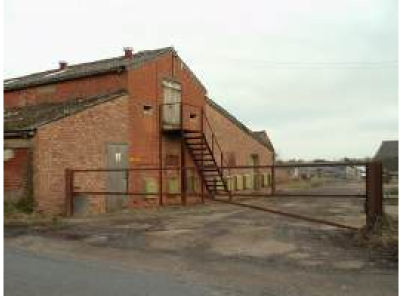

Part of Sycamore Farm © Robert Edwards (cc-by-sa/2.0)

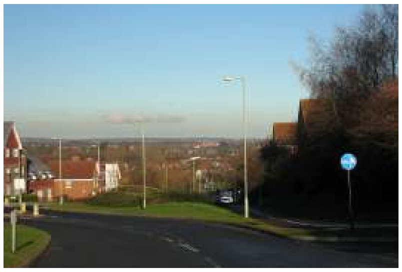

Bucksford Lane, Ashford, Kent © Oast House Archive (cc-by-sa/2.0)

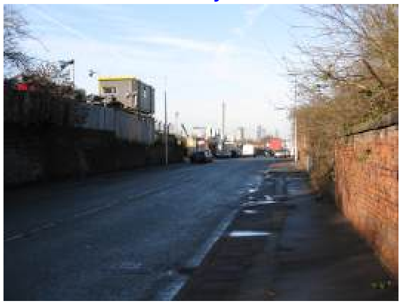

Gorton Road, Ashburys © Peter Whatley (cc-by-sa/2.0)

3.5 Atmospheric Phenomenon

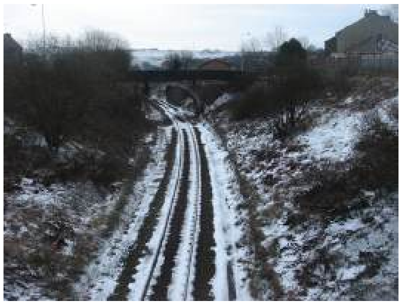

Railway at Sough/Spring Vale/Rosehill area of Darwen © Margaret Clough (cc-by-sa/2.0)

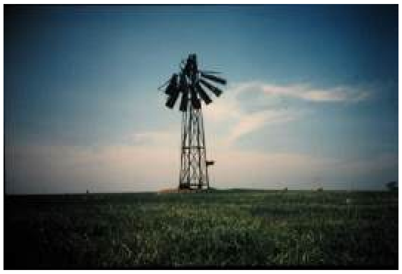

Sevington windpump © Michael Roots (cc-by-sa/2.0)

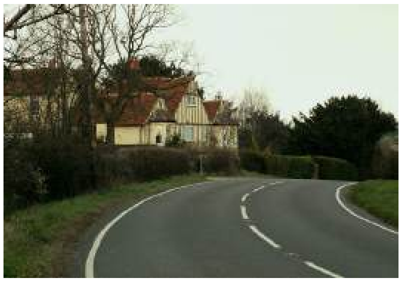

The farmhouse at Brownings Farm © Robert Edwards (cc-by-sa/2.0)

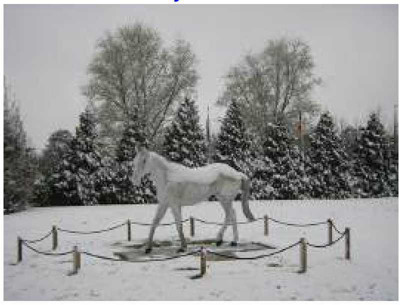

Kempton Park Racecourse © Brian Henman (cc-by-sa/2.0)

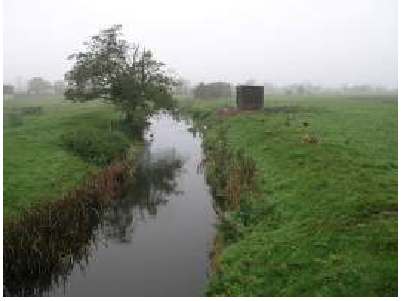

Hens and River © Michael Patterson (cc-by-sa/2.0)

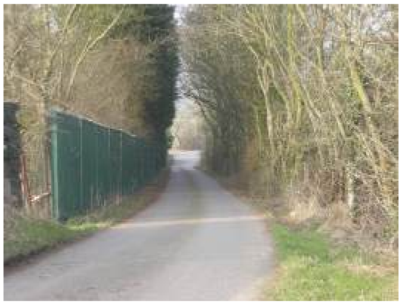

Lane beside Industrial Estate © Row17 (cc-by-sa/2.0)

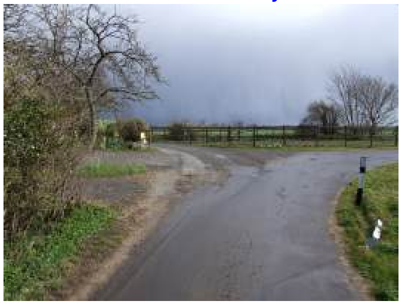

Paddock and Stables Entrance, Kemp's Corner © Ian Robertson (cc-by-sa/2.0)

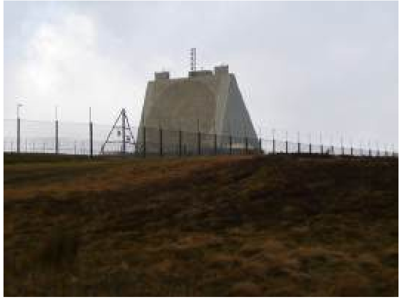

Phased array at RAF Fylingdales © Phil Catterall (cc-by-sa/2.0)

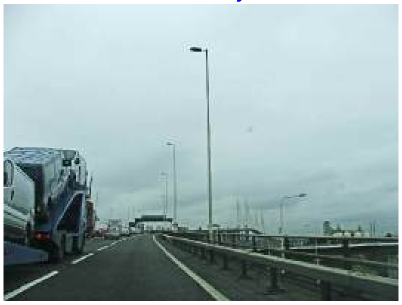

Approach to QE2 Bridge © Christine Matthews (cc-by-sa/2.0)

3.6 Automotive Design

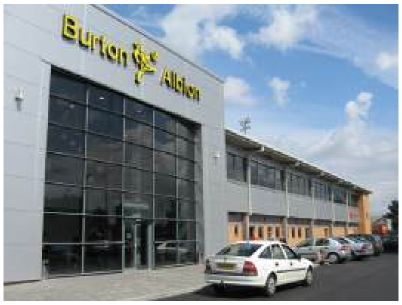

Burton Albion FC, Pirelli Stadium, Burton upon Trent, Staffordshire © Alan Slater (cc-by-sa/2.0)

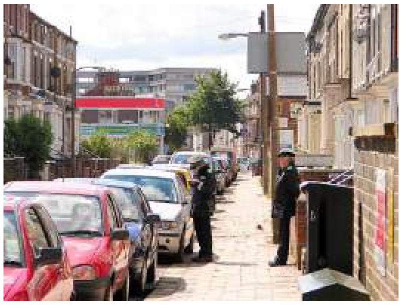

Alexandra Road, Bedford © John Lucas (cc-by-sa/2.0)

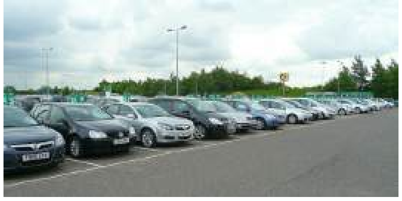

Car hire return, Stansted Airport © Jonathan Billinger (cc-by-sa/2.0)

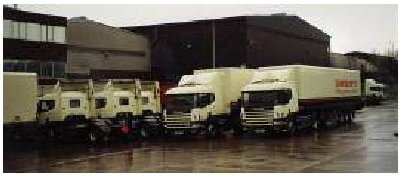

Sainsbury's Distribution Depot Middleton © Paul Anderson (cc-by-sa/2.0)

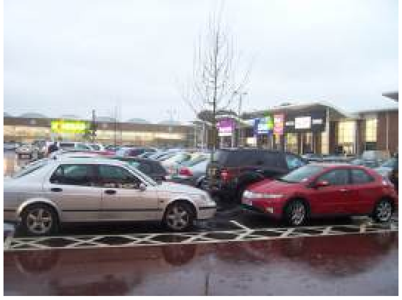

Bournemouth : Castlemore Retail Park © Lewis Clarke (cc-by-sa/2.0)

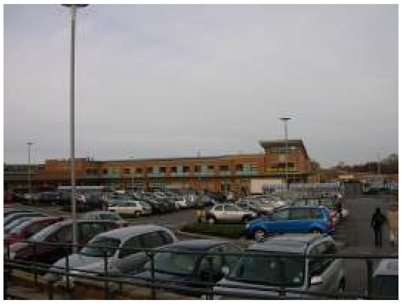

Gloucester, Abbeydale: Morrisons © Alby (cc-by-sa/2.0)

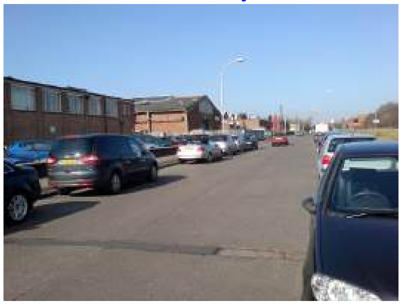

Scudamore Road © Keith Williams (cc-by-sa/2.0)

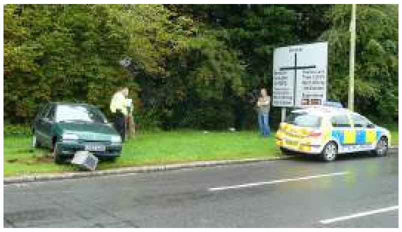

Oh, Dear! © Jonathan Billinger (cc-by-sa/2.0)

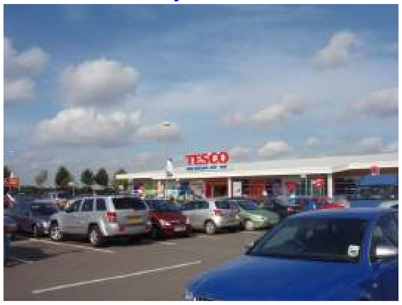

TESCO Car Park © Ajay Tegala (cc-by-sa/2.0)

3.7 Automotive Exterior

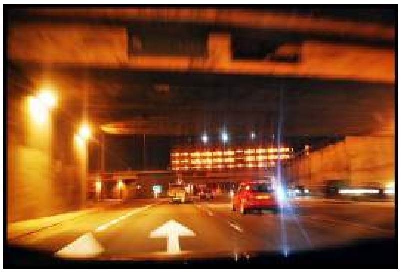
Leeds Inner ring road tunnels © philld (cc-by-sa/2.0)

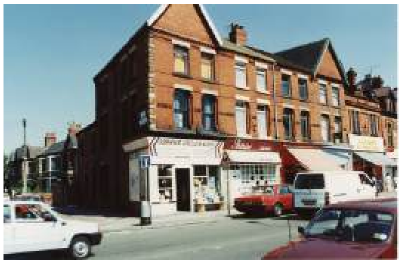
Shops in Green Lane © stan benbow (cc-by-sa/2.0)

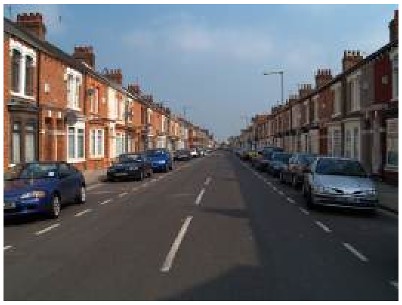
Abingdon Road © Mike Guess (cc-by-sa/2.0)

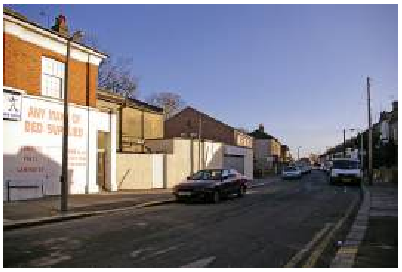
Canonbury Road, Enfield © Christine Matthews (cc-by-sa/2.0)

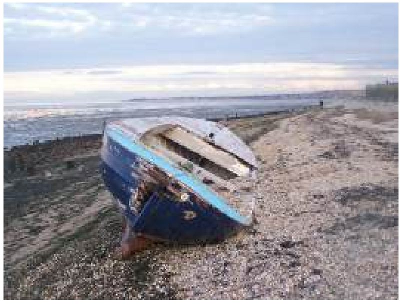
Abandoned Boat © David Anstiss (cc-by-sa/2.0)

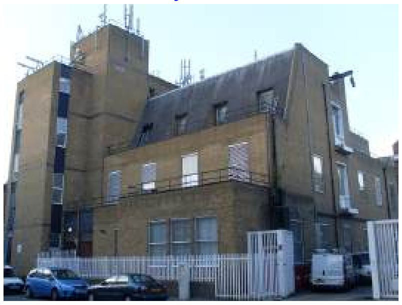
Walworth Telephone Exchange © David Hillas (cc-by-sa/2.0)

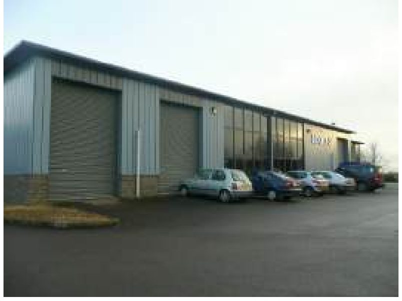
Business at Bishop's Cleeve 2 © Jonathan Billinger (cc-by-sa/2.0)

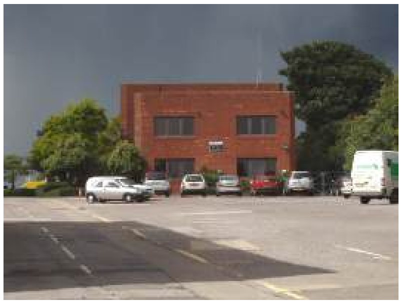
Haddenham Business Park © David Hawgood (cc-by-sa/2.0)

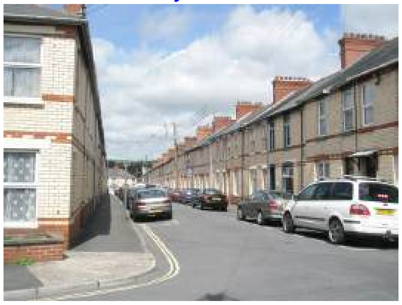
Charles Street © Basher Eyre (cc-by-sa/2.0)

3.8 Automotive Lighting

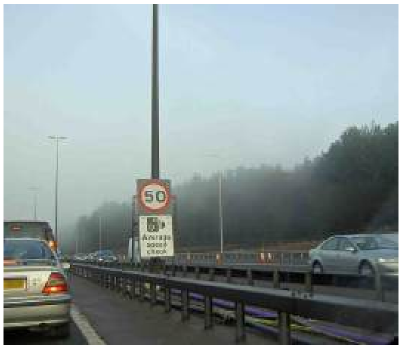

If only! © Steve Fareham (cc-by-sa/2.0)

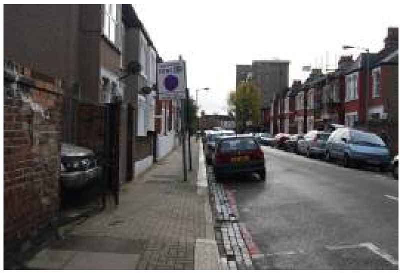

Nutwell Street, Tooting © N Chadwick (cc-by-sa/2.0)

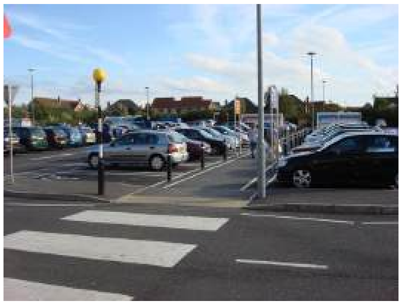

Tesco Supermarket Car Park © Oxyman (cc-by-sa/2.0)

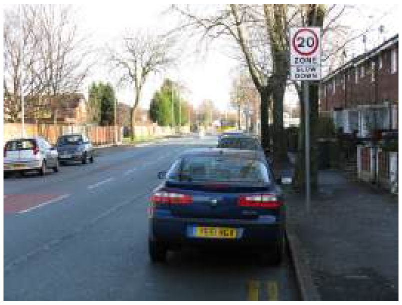

Kirkmanshulme Lane © Peter Whatley (cc-by-sa/2.0)

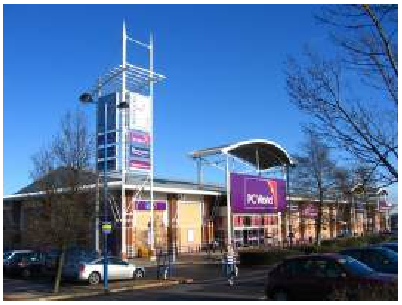

Bolton Gate Retail Park © John Tustin (cc-by-sa/2.0)

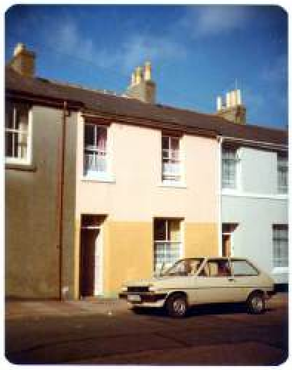

21 St Paul's Road, Babbacombe, in 1983 © NA (cc-by-sa/2.0)

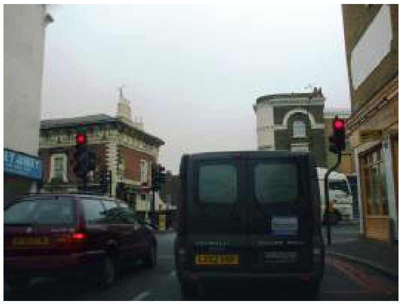

Besson Street, SE14 © Phillip Perry (cc-by-sa/2.0)

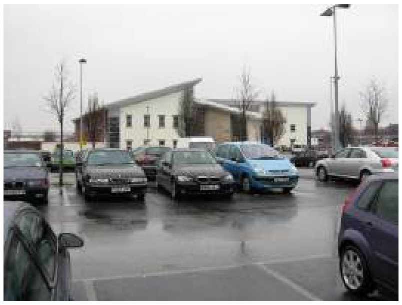

Wythenshawe Hospital - car park and new buildings © Peter Whatley (cc-by-sa/2.0)

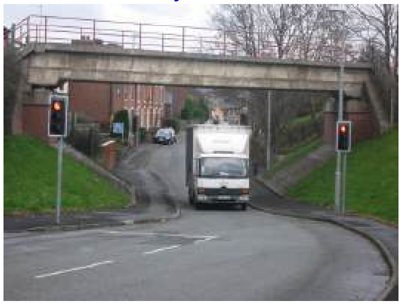

Pilsworth Road Railway Bridge © Paul Anderson (cc-by-sa/2.0)

3.9 Automotive Parking Light

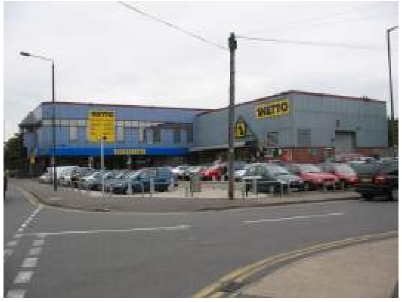

Shops on the corner of Love Lane © Stephen Craven (cc-by-sa/2.0)

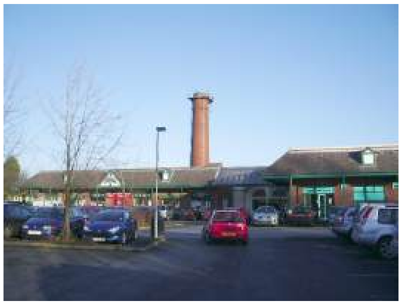

The Carrington Centre, Eccleston © Alexander P Kapp (cc-by-sa/2.0)

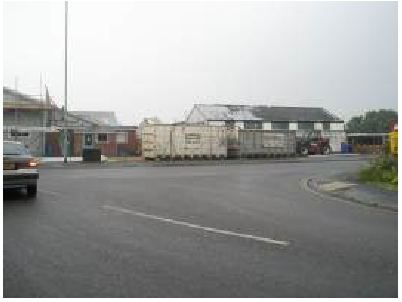

Junction of Dundas lane and Airport Service Road © Basher Eyre (cc-by-sa/2.0)

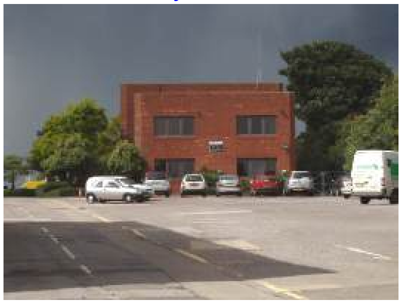

Haddenham Business Park © David Hawgood (cc-by-sa/2.0)

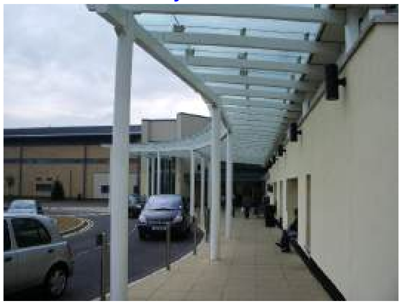

Waiting area at the main entrance to The Royal Blackburn Hospital © Alexander P Kapp (cc-by-sa/2.0)

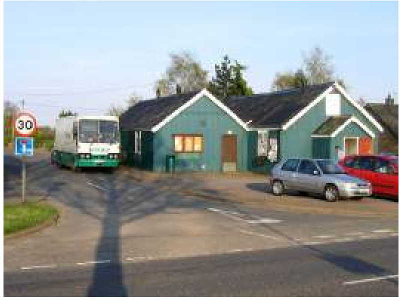

Poringland Village hall © Graham Hardy (cc-by-sa/2.0)

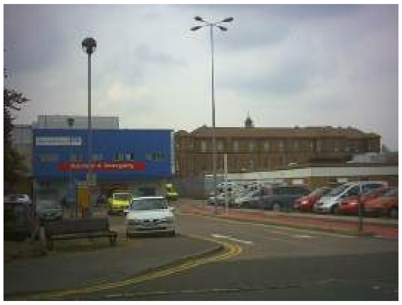

Mayday University Hospital, Mayday Road, Croydon. © Noel Foster (cc-by-sa/2.0)

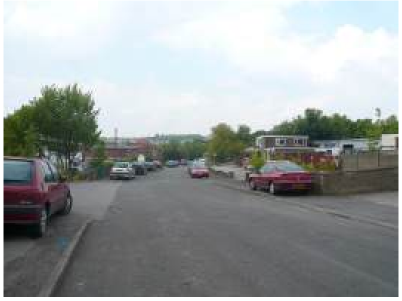

Dronfield - Looking back down Callywhite Lane © Alan Heardman (cc-by-sa/2.0)

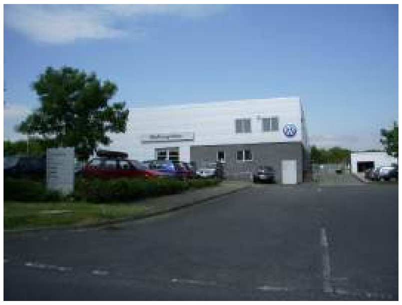

"Walkingshaw" © Alexander P Kapp (cc-by-sa/2.0)

3.10 Automotive Tail Brake Light

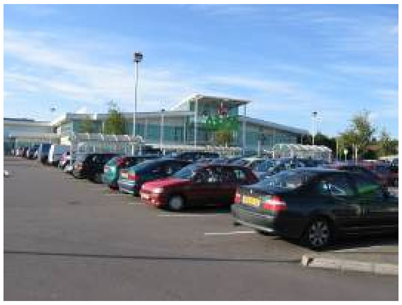

ASDA, Longwell Green © William Avery (cc-by-sa/2.0)

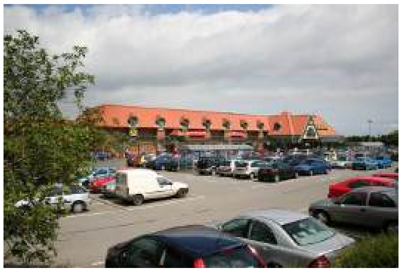

Morrison's Supermarket © Richard Croft (cc-by-sa/2.0)

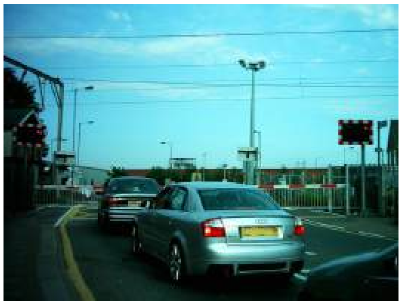

Brimsdown Level crossing (Green Street) © Matthew Singh (cc-by-sa/2.0)

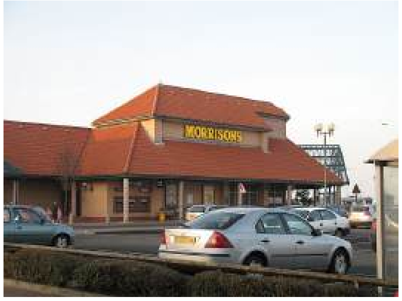

Morrisons, Berwick © Richard Webb (cc-by-sa/2.0)

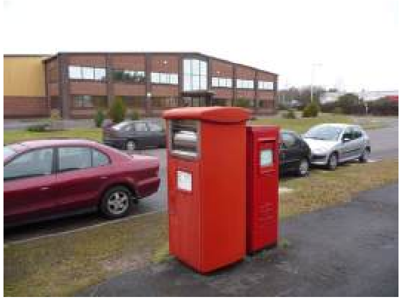

Holton Heath: postbox s BH16 290 and BH16 512, Holton Road © Chris Downer (cc-by-sa/2.0)

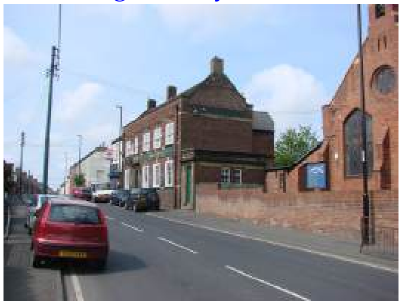

The Travellers Rest, Shiney Row © Bill Henderson (cc-by-sa/2.0)

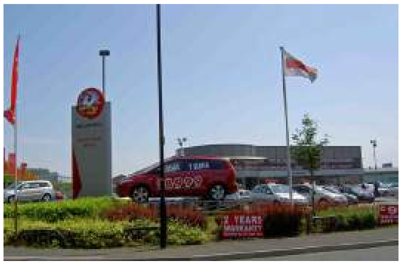

Bristol Street Motors © Steve Fareham (cc-by-sa/2.0)

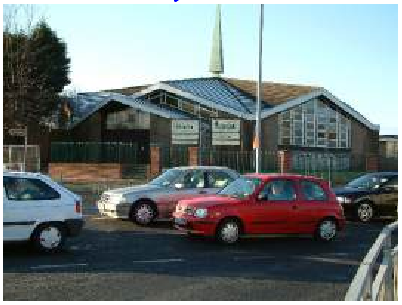

Our Lady of Walsingham © Peter Hodge (cc-by-sa/2.0)

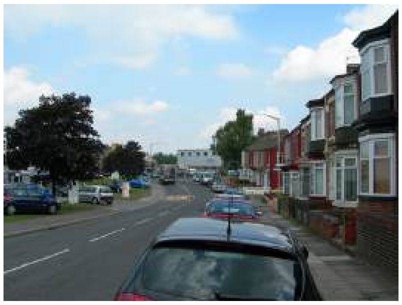

East Mount Road, Darlington © Danny P Robinson (cc-by-sa/2.0)

3.11 Automotive Tire

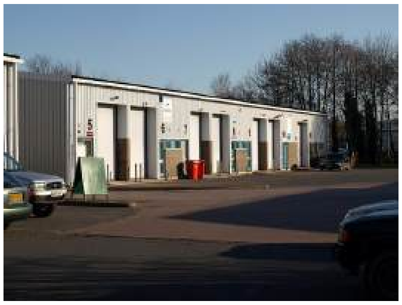

Swift Industrial Estate, Kingsteignton © Derek Harper (cc-by-sa/2.0)

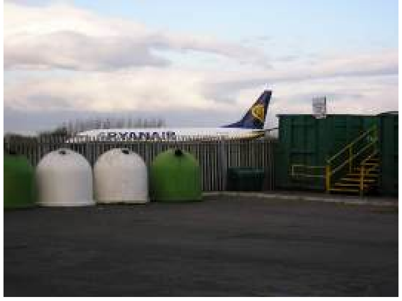

Destructor Road © Roger W Haworth (cc-by-sa/2.0)

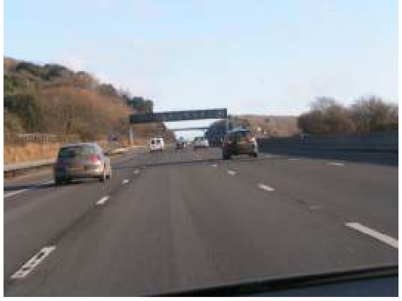

M5 northbound, leaving the Somerset Levels © Rob Purvis (cc-by-sa/2.0)

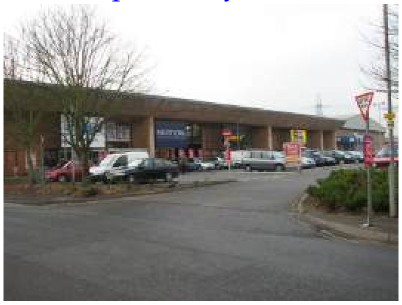

Changed use © Bill Nicholls (cc-by-sa/2.0)

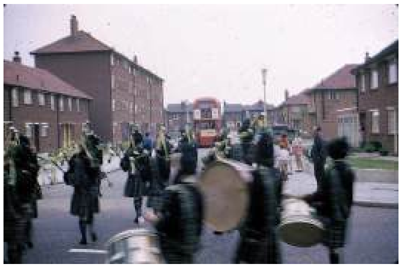

Girl Pipers in Magdala Road © Clive Warneford (cc-by-sa/2.0)

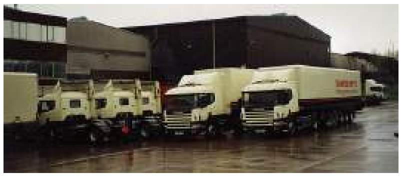

Sainsbury's Distribution Depot Middleton © Paul Anderson (cc-by-sa/2.0)

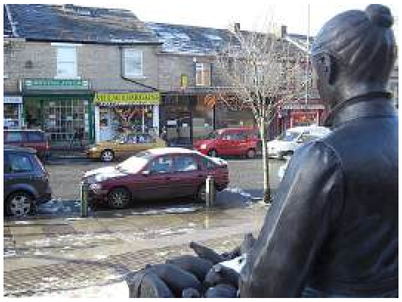

Stamford Street, Top Mossley © michael ely (cc-by-sa/2.0)

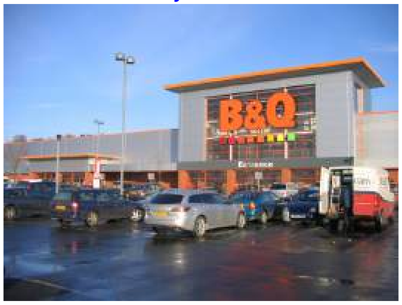

B&Q Warehouse, Halesowen. © Roy Hughes (cc-by-sa/2.0)

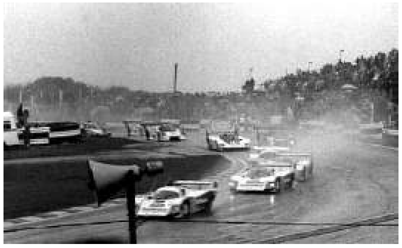

Group C race in the rain, Brands Hatch, 1987 © John Goldsmith (cc-by-sa/2.0)

3.12 Biome

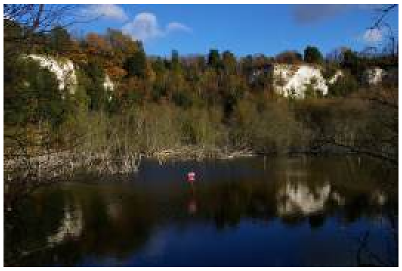

Pond in the Pit © Glyn Baker (cc-by-sa/2.0)

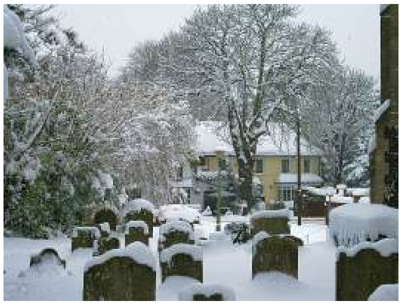

View from churchyard of St Martin of Tours © Hugh Craddock (cc-by-sa/2.0)

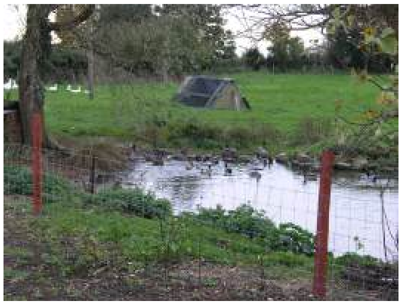

Duckpond in Shingleford Farm © Hywel Williams (cc-by-sa/2.0)

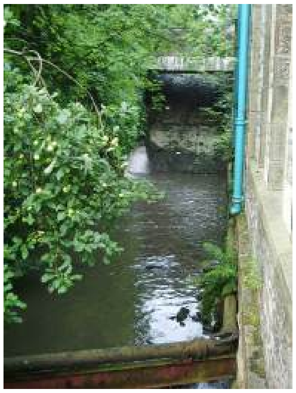

Mearley Brook as it flows along Stalwart Carpet Works © Alexander P Kapp (cc-by-sa/2.0)

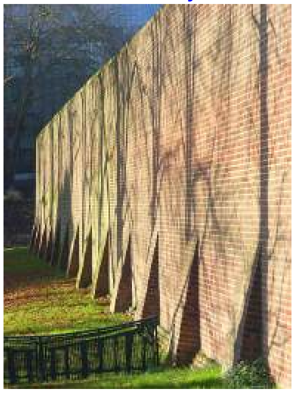

Prison wall, Reading © Andrew Smith (cc-by-sa/2.0)

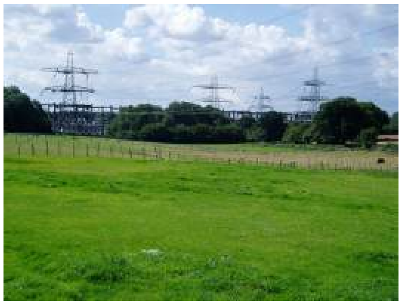

Is it safe to live under power cables? © Peter Holmes (cc-by-sa/2.0)

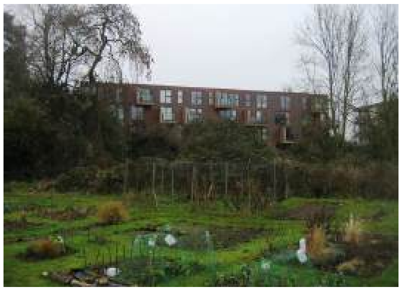

Do I fit in? © Mr Ignavy (cc-by-sa/2.0)

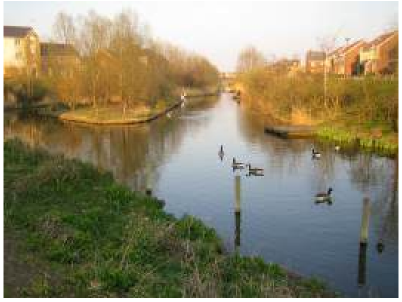

Thamesmead: Gallions Canal © Nigel Cox (cc-by-sa/2.0)

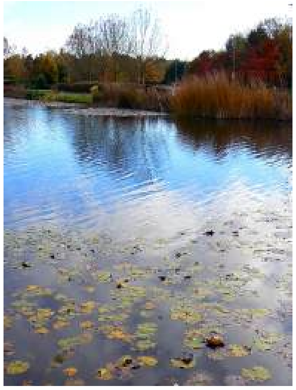

Nationwide Building Society, Piper's Way, Swindon © Brian Robert Marshall (cc-by-sa/2.0)

3.13 Boat

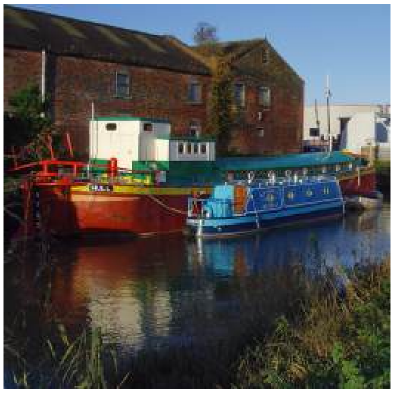

Barges at Grovehill, Beverley © Paul Harrop (cc-by-sa/2.0)

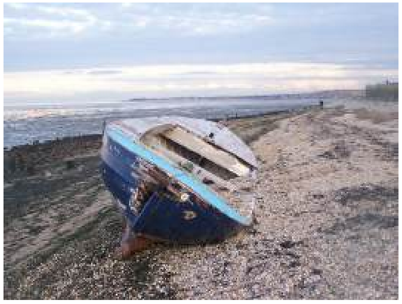

Abandoned Boat © David Anstiss (cc-by-sa/2.0)

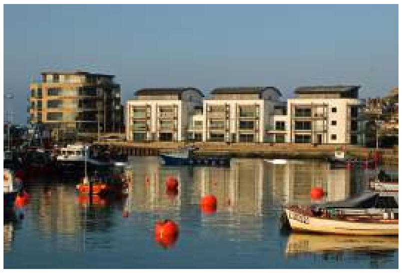

West Bay Development © Tony Watkins (cc-by-sa/2.0)

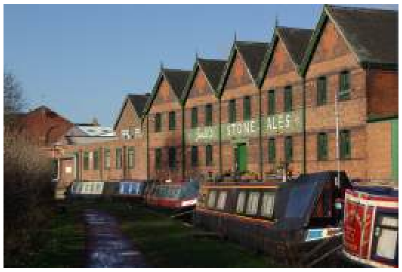

Trent & Mersey Canal, Stone © Stephen McKay (cc-by-sa/2.0)

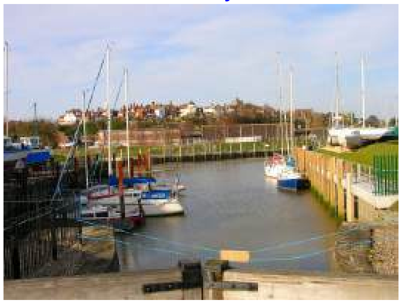

Confluence of the Brede and Tillingham Rivers © Simon Carey (cc-by-sa/2.0)

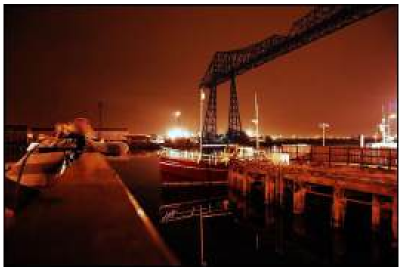

Transporter bridge at night © philld (cc-by-sa/2.0)

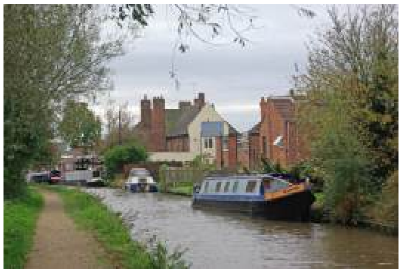

Coventry Canal, Longford © Stephen McKay (cc-by-sa/2.0)

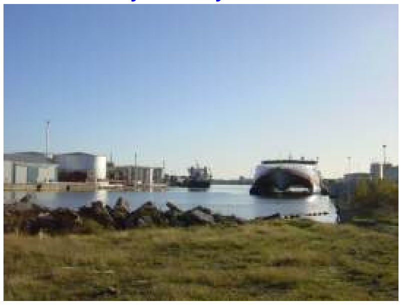

West Float from Poulton Bridge © Sue Adair (cc-by-sa/2.0)

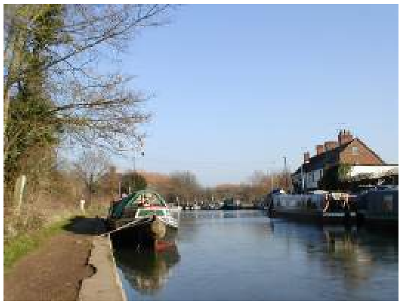

Grand Union Canal beside The Cape of Good Hope © Row17 (cc-by-sa/2.0)

3.14 Body of Water

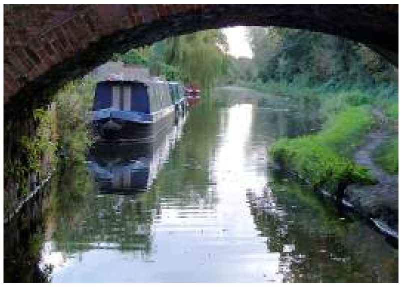

Staffordshire and Worcestershire Canal at Newbridge, Wolverhampton © Roger D Kidd (cc-by-sa/2.0)

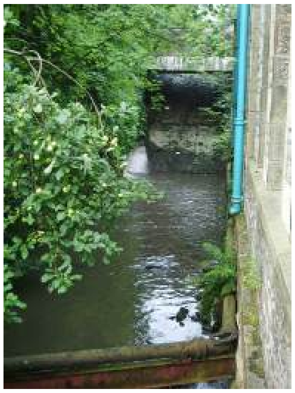

Mearley Brook as it flows along Stalwart Carpet Works © Alexander P Kapp (cc-by-sa/2.0)

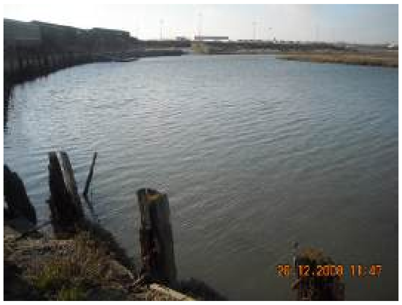

Murston Wharf, Milton Creek © Joe White (cc-by-sa/2.0)

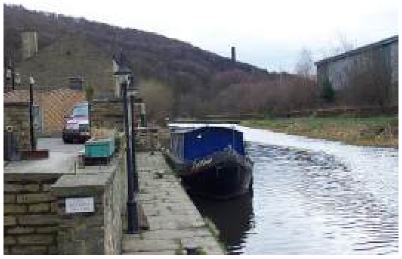

Narrow Boat on the Calder & Hebble Navigation at Elland Park Wood © Richard Kay (cc-by-sa/2.0)

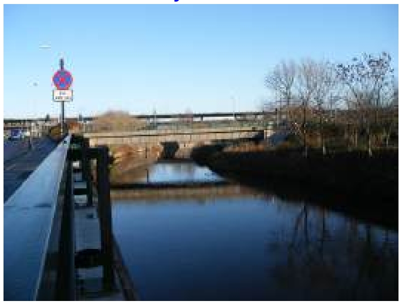

The River Don © SMJ (cc-by-sa/2.0)

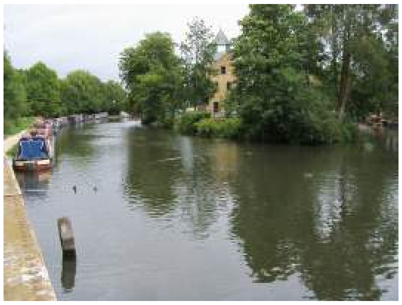

Grand Union Canal © Shaun Ferguson (cc-by-sa/2.0)

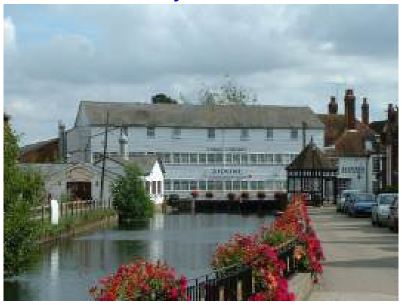

Antiques centre and restaurant © Keith Evans (cc-by-sa/2.0)

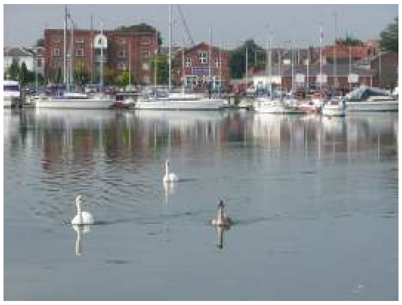

A visit from the family © Trevor Holmes (cc-by-sa/2.0)

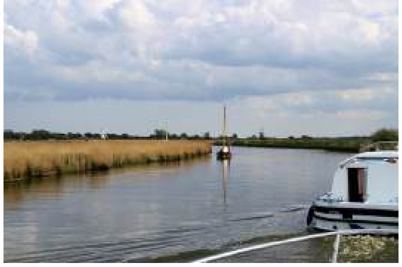

River Thurne above Thurne © Pierre Terre (cc-by-sa/2.0)

3.15 Branch

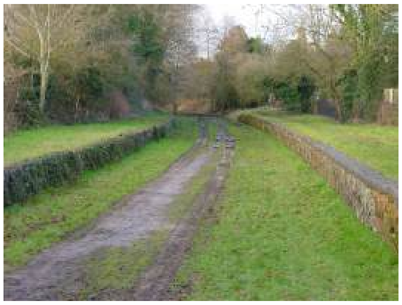

Site of Denstone Railway Station © Linda Bailey (cc-by-sa/2.0)

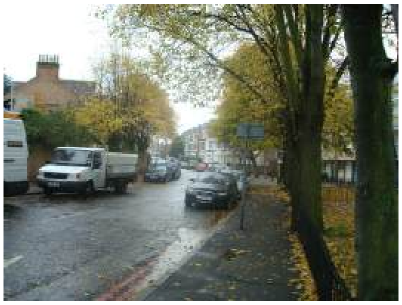

Montrell Road, SW2 © Stacey Harris (cc-by-sa/2.0)

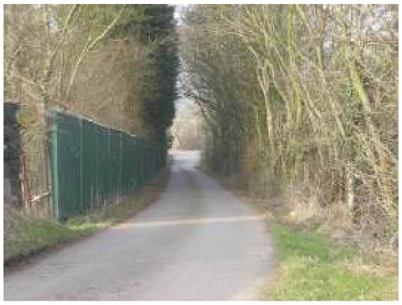

Lane beside Industrial Estate © Row17 (cc-by-sa/2.0)

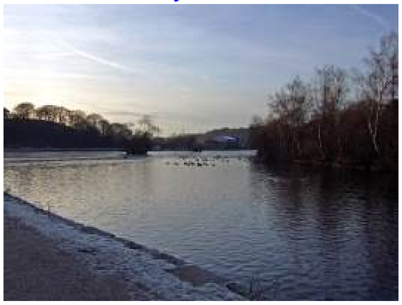

Etherow Country Park © Bob Abell (cc-by-sa/2.0)

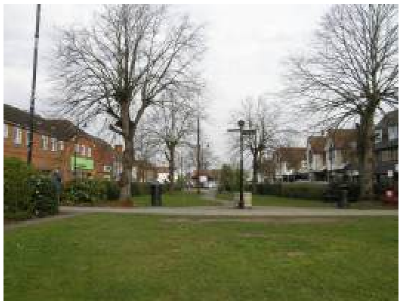

Thatcham: The Broadway © Chris Downer (cc-by-sa/2.0)

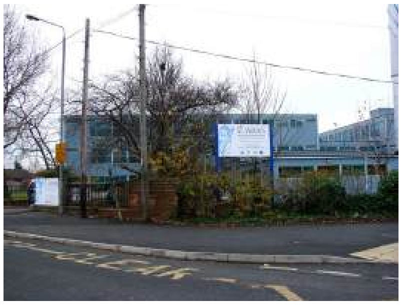

St Wilfreds High School, North Featherstone © Bill Henderson (cc-by-sa/2.0)

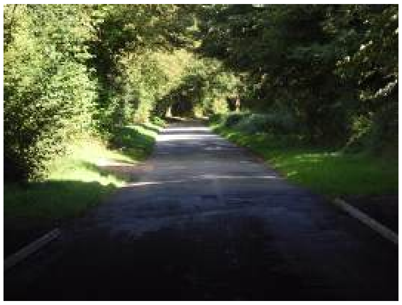

Welford Road, Wickham © Andrew Smith (cc-by-sa/2.0)

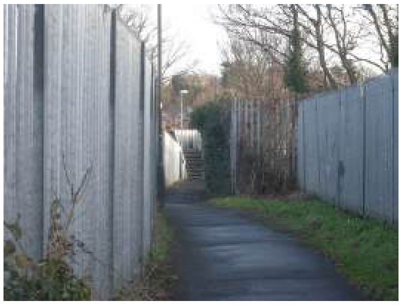

Footpath to the footbridge © Row17 (cc-by-sa/2.0)

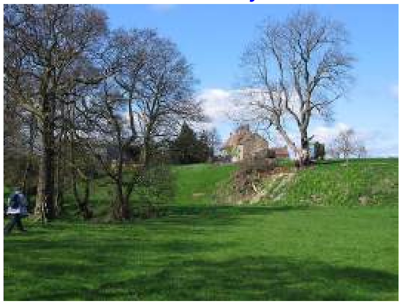

Streatlam Grove Farm © Roger Smith (cc-by-sa/2.0)

3.16 Bridge

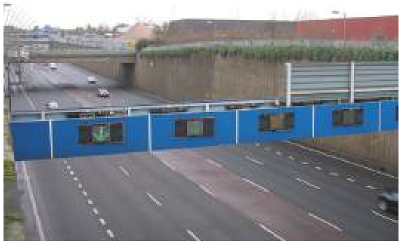

HP Sauce Factory Aston Cross after demolition had taken place. © Roy Hughes (cc-by-sa/2.0)

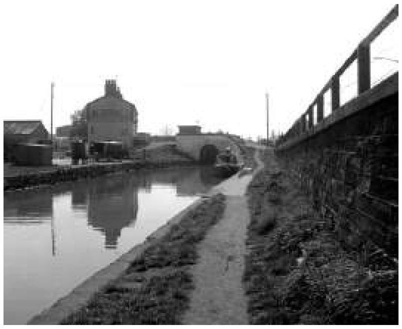

Below Kings Lock, Middlewich © Dr Neil Clifton (cc-by-sa/2.0)

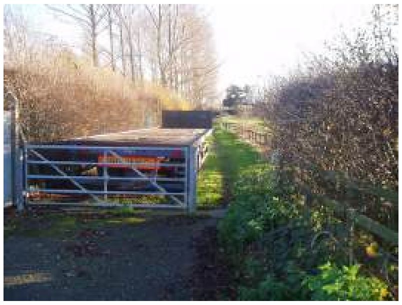

Entrance to bridleway © Oliver White (cc-by-sa/2.0)

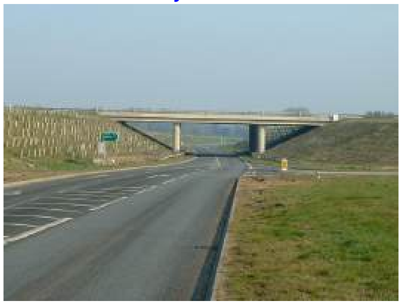

A11 crossing B1111 © Keith Evans (cc-by-sa/2.0)

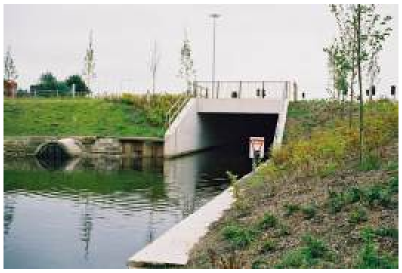

New Tunnel, Rochdale Canal © Dr Neil Clifton (cc-by-sa/2.0)

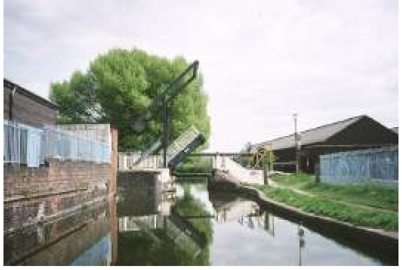

Ivy House Lift Bridge © David Stowell (cc-by-sa/2.0)

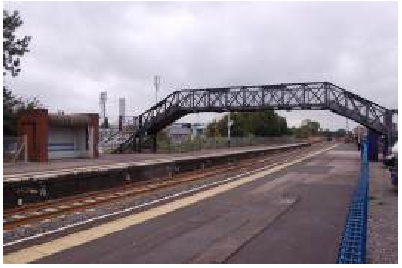

Patchway railway station © Roger Davies (cc-by-sa/2.0)

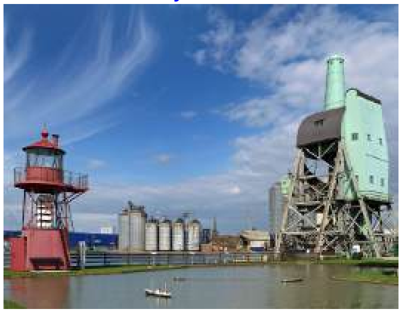

Goole Model Boat Club pond © Steve Fareham (cc-by-sa/2.0)

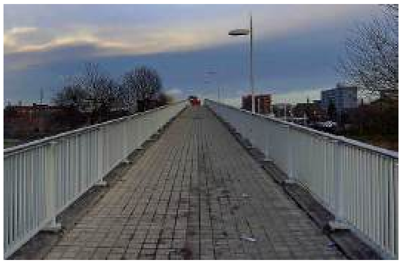

The Aquarius footbridge from Archway Hulme © Steve Fareham (cc-by-sa/2.0)

3.17 Building

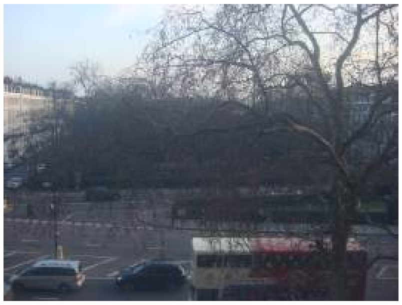

Thurloe Square from the V & A © Oxyman (cc-by-sa/2.0)

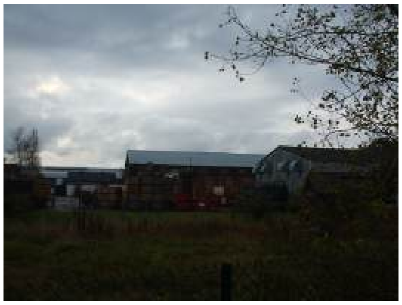

Melton Constable Industrial estate © Ashley Dace (cc-by-sa/2.0)

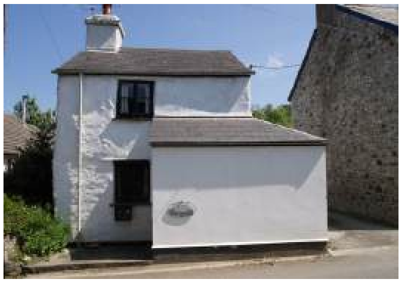

Old Post Office, Lawhitton © Derek Harper (cc-by-sa/2.0)

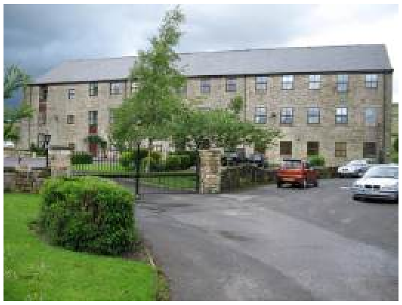

The Meadows at Red Lumb © Paul Anderson (cc-by-sa/2.0)

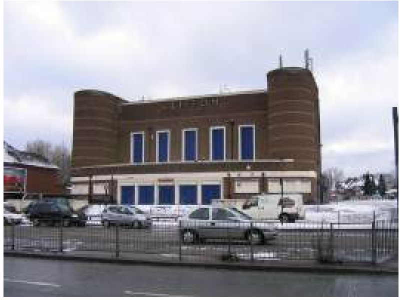

Clifton Bingo Ex-Clifton Cinema Perry Barr. © Roy Hughes (cc-by-sa/2.0)

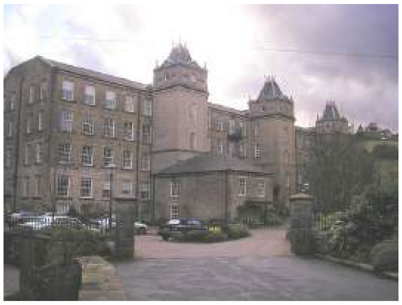

Barkisland Mill © John Illingworth (cc-by-sa/2.0)

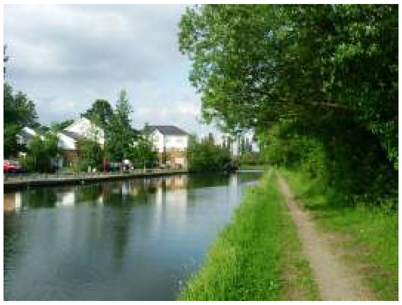

Grand Union Canal, Southall © Phillip Perry (cc-by-sa/2.0)

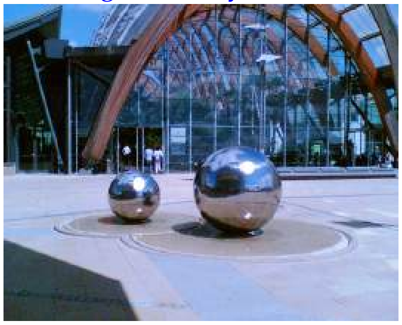

Great Balls Of Steel © Tim Marchant (cc-by-sa/2.0)

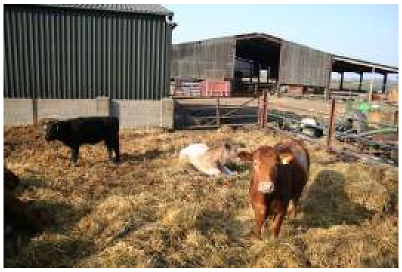

Manor farm cattle © Richard Croft (cc-by-sa/2.0)

3.18 Building Material

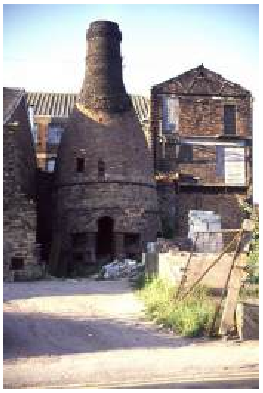

Bottle oven, Minkstone Works, Longton © Chris Allen (cc-by-sa/2.0)

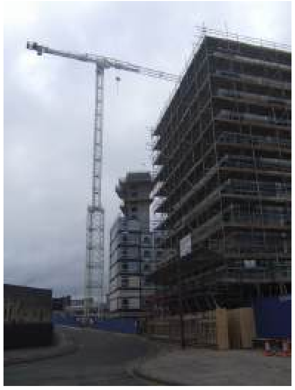

Victoria Hall - University of Wolverhampton © John M (cc-by-sa/2.0)

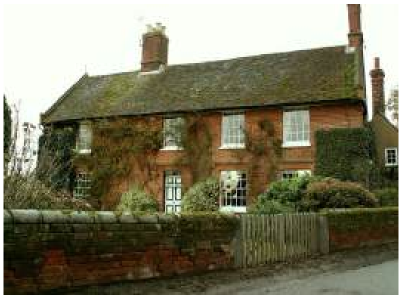

Farmhouse at Red House Farm © Robert Edwards (cc-by-sa/2.0)

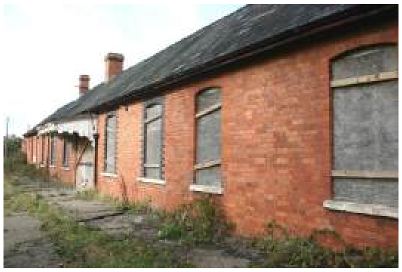

Lydd Station © Mark Duncan (cc-by-sa/2.0)

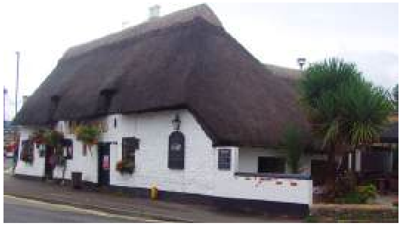

King's Head, Bishops Cleeve © John Matthews (cc-by-sa/2.0)

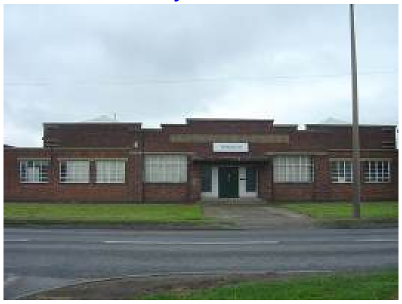

Training Centre, Stanton Works © Alan Murray-Rust (cc-by-sa/2.0)

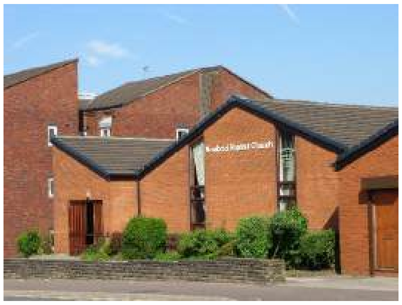

Newbold Baptist Church © Michael Fowler (cc-by-sa/2.0)

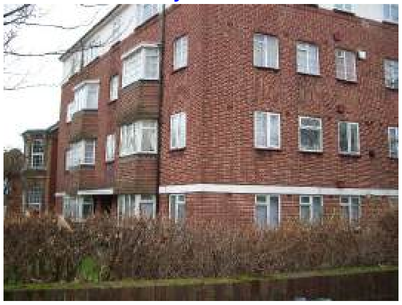

Apartment Block, Leytonstone © John Davies (cc-by-sa/2.0)

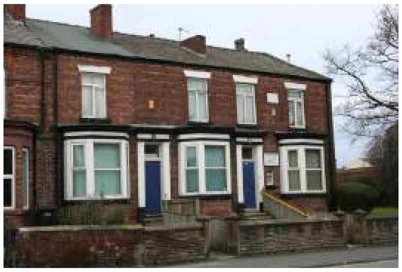

The old Doctors' Surgery. © David Ashcroft (cc-by-sa/2.0)

3.19 Bus

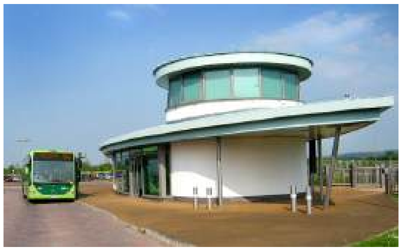

Park and ride bus station © Dave Croker (cc-by-sa/2.0)

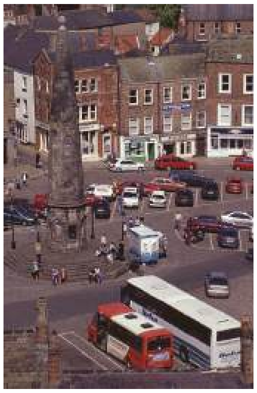

Richmond Market Place © Stephen McKay (cc-by-sa/2.0)

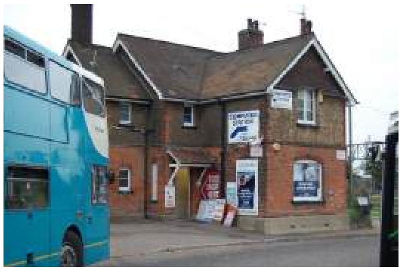

Rayleigh Railway Station © Trevor Harris (cc-by-sa/2.0)

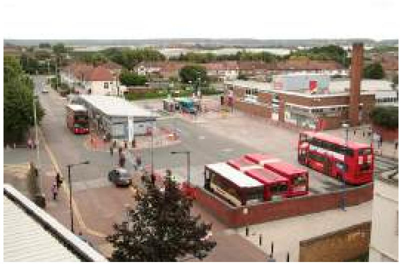

Waltham Cross bus station © Richard Croft (cc-by-sa/2.0)

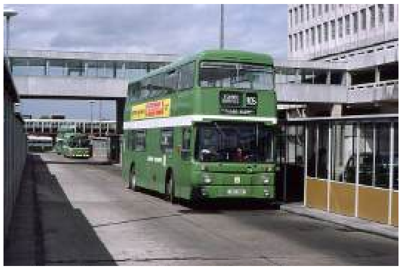

Harlow Bus Station © Martin Addison (cc-by-sa/2.0)

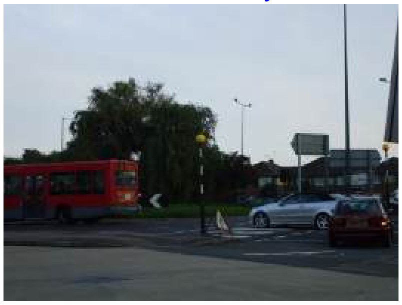

A4/A30 roundabout, Hounslow © Phillip Perry (cc-by-sa/2.0)

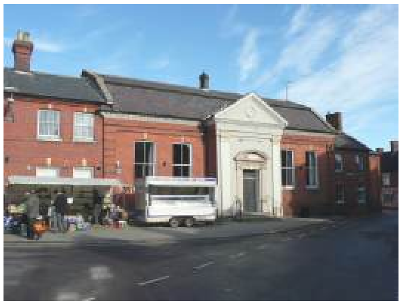

Town Hall and market stalls, Aylsham © Humphrey Bolton (cc-by-sa/2.0)

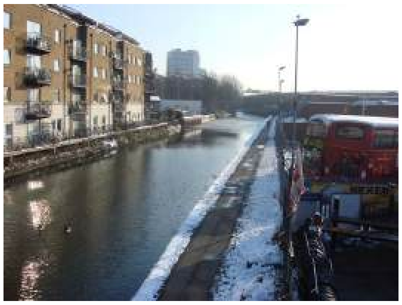

Grand Union Canal from Great Western Rd © Oxyman (cc-by-sa/2.0)

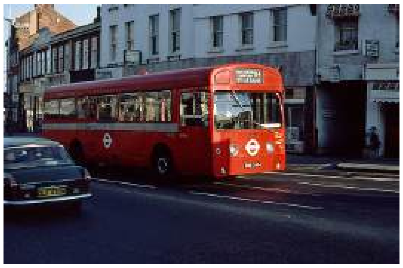

84 in Barnet © Martin Addison (cc-by-sa/2.0)

3.20 Canal

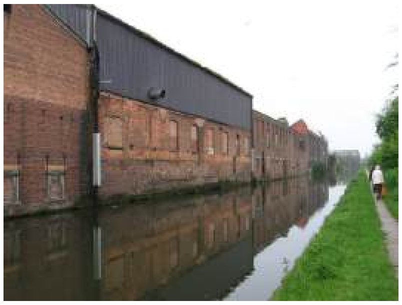

Small Heath - canal-side factories © Dave Bevis (cc-by-sa/2.0)

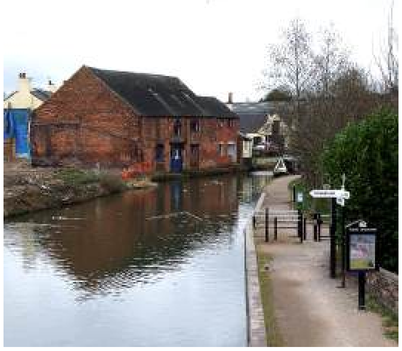

Birmingham & Fazeley Canal, Fazeley Junction © Rob Farrow (cc-by-sa/2.0)

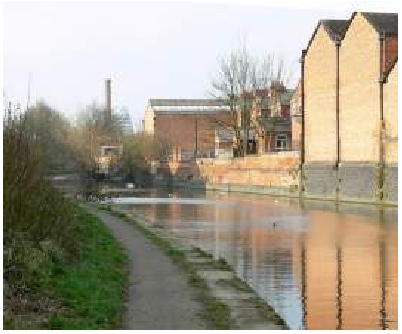

Grand Union Canal and towpath in Leicester. © Mat Fascione (cc-by-sa/2.0)

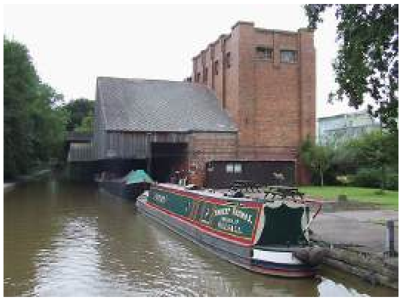

Cadbury Wharf, Knighton, Staffordshire © Roger D Kidd (cc-by-sa/2.0)

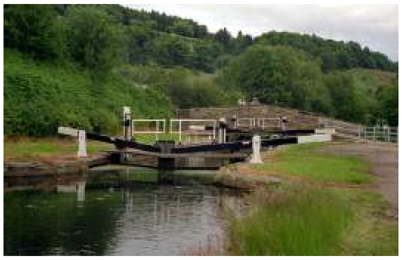

Riddings Lock No 6, Huddersfield Broad Canal © Dr Neil Clifton (cc-by-sa/2.0)

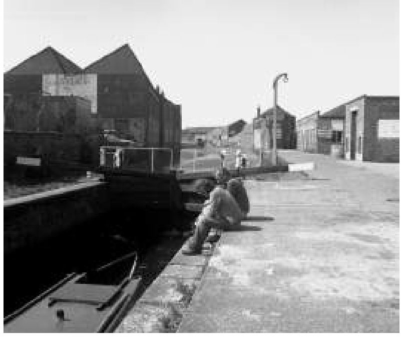

North Lock, Soar Navigation, Leicester © Dr Neil Clifton (cc-by-sa/2.0)

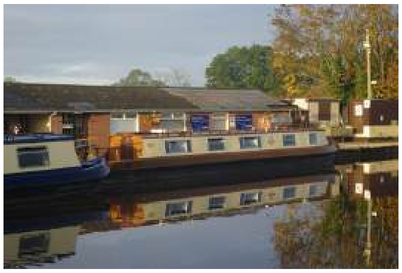

Maestermyn Marine © Stephen McKay (cc-by-sa/2.0)

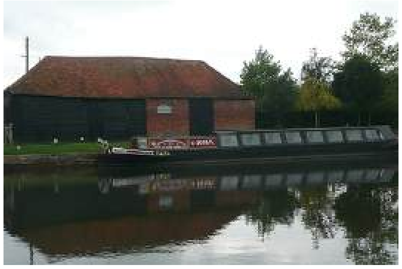

The most southerly point on the inland waterways system © Graham Horn (cc-by-sa/2.0)

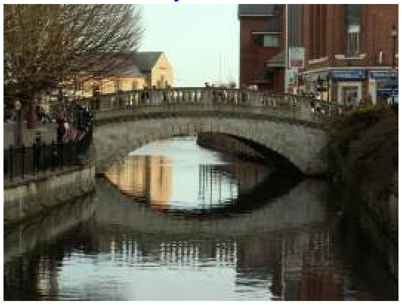

Moulsham Bridge over the river Can in Chelmsford © Robert Edwards (cc-by-sa/2.0)

3.21 Car

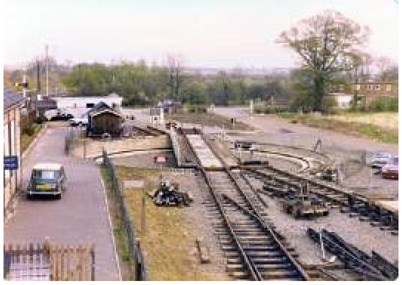

Turntable Installation © Clive Warneford (cc-by-sa/2.0)

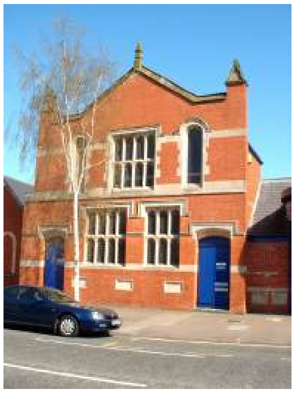

The former South Bar Congregational Church © Wendy Parkinson (cc-by-sa/2.0)

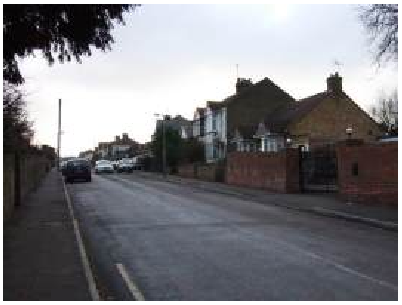

Salisbury Avenue, Rainham © Chris Whippet (cc-by-sa/2.0)

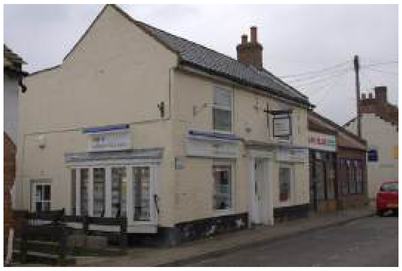

Estate Agents, Stalham High St © Pauline A Marsh (cc-by-sa/2.0)

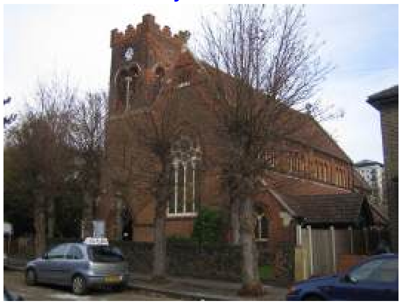

Chadwell Heath: St Chad's Church © Nigel Cox (cc-by-sa/2.0)

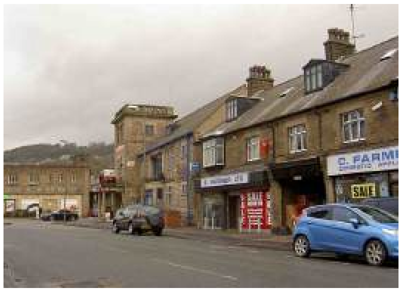

Matlock Bridge shops © Steve Fareham (cc-by-sa/2.0)

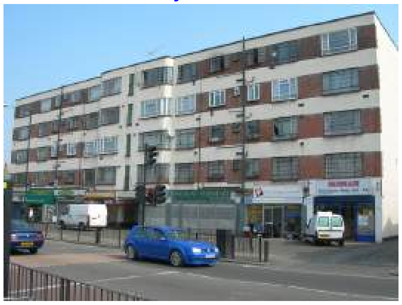

Beaumont Court, Clapton © Danny P Robinson (cc-by-sa/2.0)

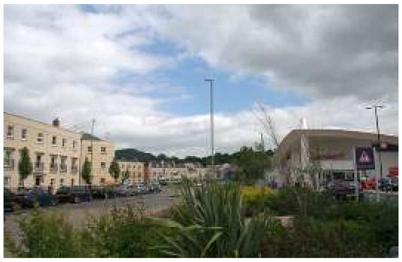

New Development, Oakley © Bob Embleton (cc-by-sa/2.0)

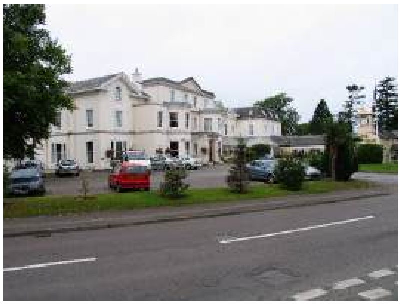

Cheltenham Park Hotel, Charlton Kings © Terry Jacombs (cc-by-sa/2.0)

3.22 Channel

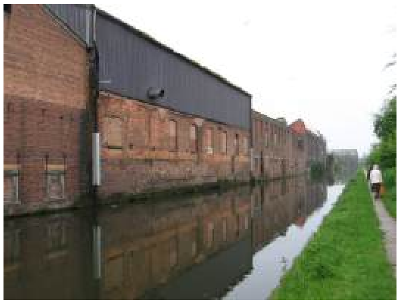

Small Heath - canal-side factories © Dave Bevis (cc-by-sa/2.0)

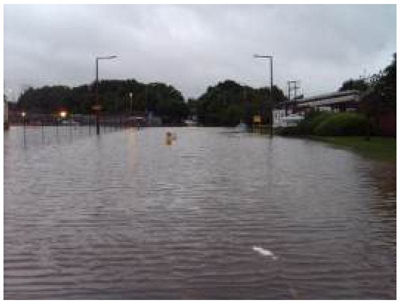

Wombwell Lane flooded © Jeff Pearson (cc-by-sa/2.0)

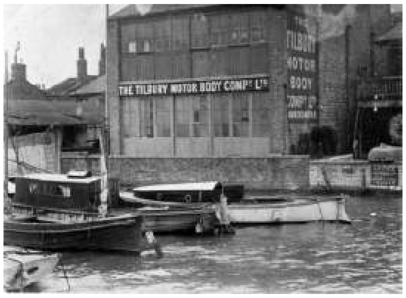

The West Lodge Tilbury works at Lower Mall. Circa 1905 © NA (cc-by-sa/2.0)

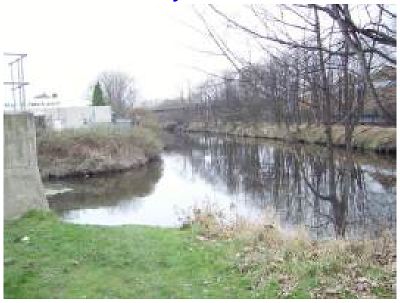

Confluence of River Rother with River Don © Shelagh Craven (cc-by-sa/2.0)

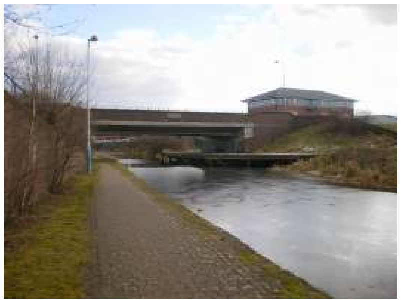

The Causeway Bridge over the Rochdale Canal © Alexander P Kapp (cc-by-sa/2.0)

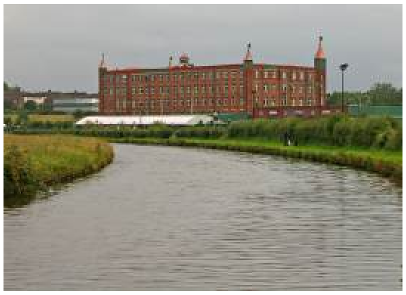

Botany Bay © Mr T (cc-by-sa/2.0)

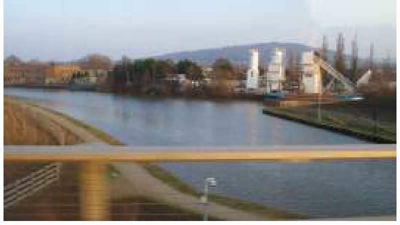

View from the new canal bridge © David Robinson (cc-by-sa/2.0)

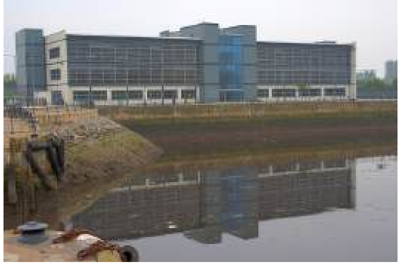

New Office Block Overlooking the Docks © Mick Garratt (cc-by-sa/2.0)

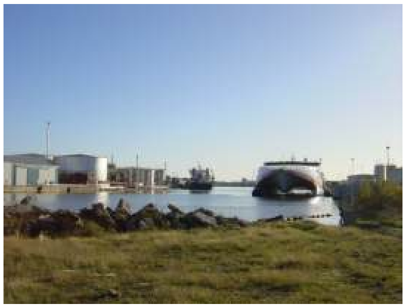

West Float from Poulton Bridge © Sue Adair (cc-by-sa/2.0)

3.23 Chapel

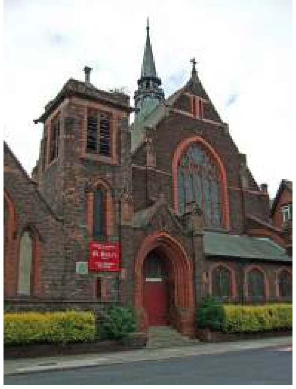

St Bede, Toxteth © S Parish (cc-by-sa/2.0)

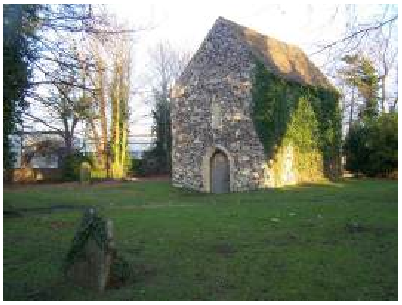

Remains of Church © David Anstiss (cc-by-sa/2.0)

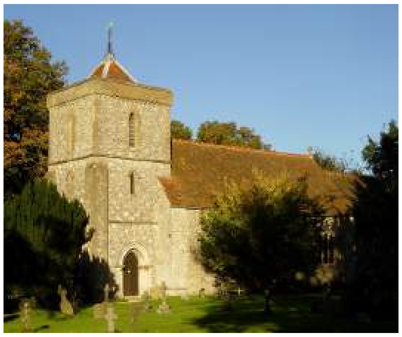

St Mary's Church, Herriard © Hugh Chevallier (cc-by-sa/2.0)

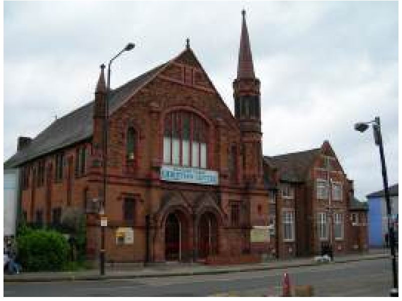

Manor Park Christian Centre, 34 High Street North, E12 © Danny P Robinson (cc-by-sa/2.0)

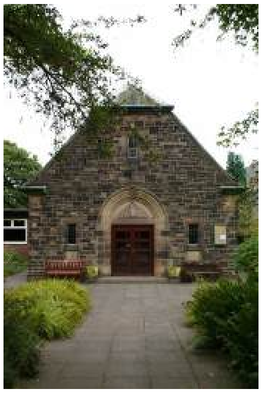

The Parish Church of St James, Woolfold © Alexander P Kapp (cc-by-sa/2.0)

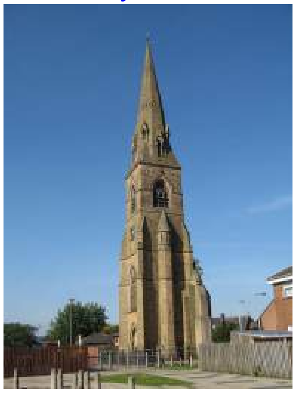

St George, Charlestown © Sue Adair (cc-by-sa/2.0)

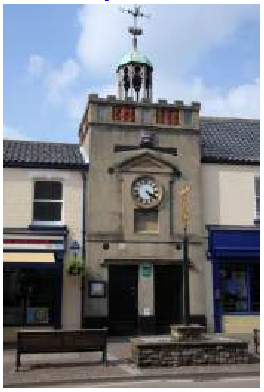

Watton Clock Tower © Bob Jones (cc-by-sa/2.0)

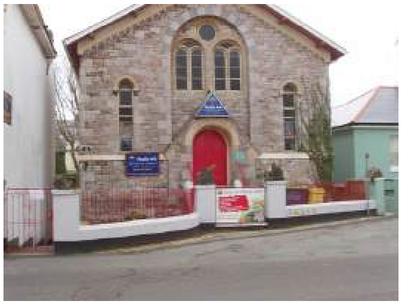

Childcare Centre in Babbacombe © Jennifer Vaughan (cc-by-sa/2.0)

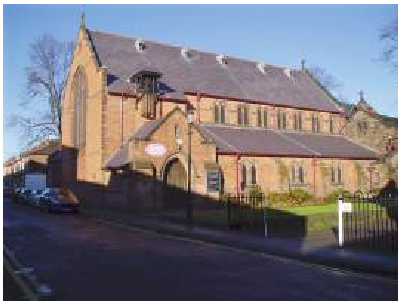

Christ Church, Chester © Eirian Evans (cc-by-sa/2.0)

3.24 Church

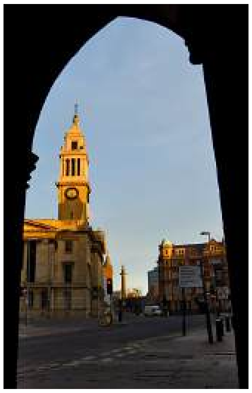

Lowgate, Hull © Paul Harrop (cc-by-sa/2.0)

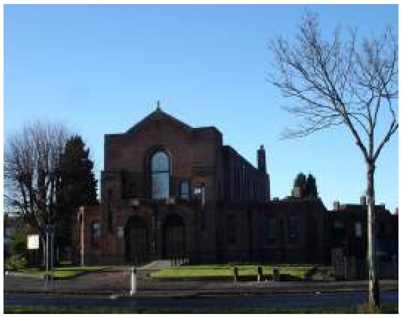

Aspley Methodist Church © Oxymoron (cc-by-sa/2.0)

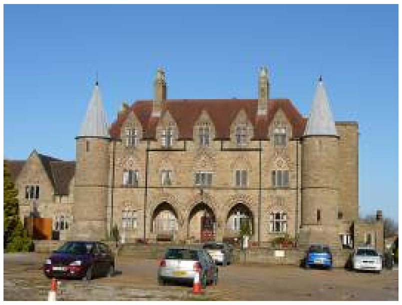

The old military barracks off Crompton Road, Macclesfield © Colin Park (cc-by-sa/2.0)

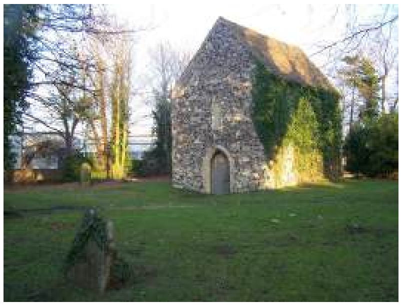

Remains of Church © David Anstiss (cc-by-sa/2.0)

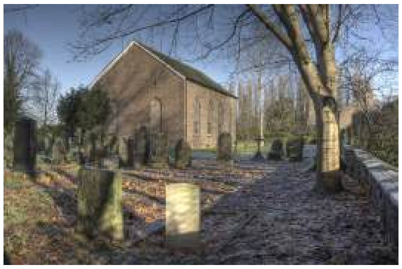

St George's Church, Carrington © Tom Jeffs (cc-by-sa/2.0)

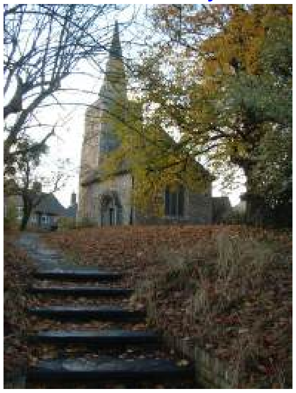

St Peter's Church, Castle Hill © PAUL FARMER (cc-by-sa/2.0)

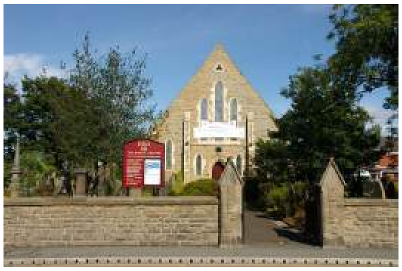

Jesus The King Centre © Alexander P Kapp (cc-by-sa/2.0)

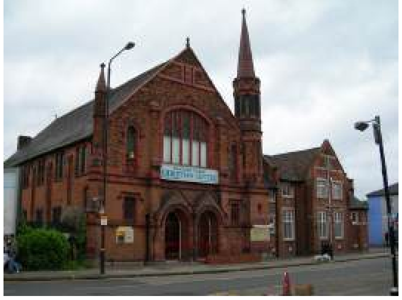

Manor Park Christian Centre, 34 High Street North, E12 © Danny P Robinson (cc-by-sa/2.0)

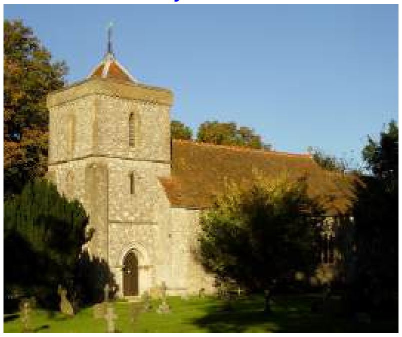

St Mary's Church, Herriard © Hugh Chevallier (cc-by-sa/2.0)

3.25 City

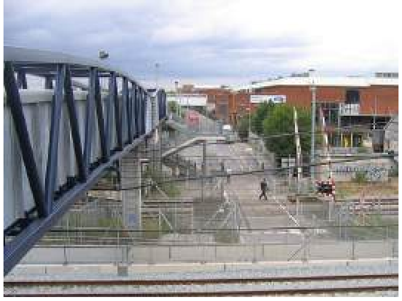
Footbridge superseding a level crossing © Stephen Craven (cc-by-sa/2.0)

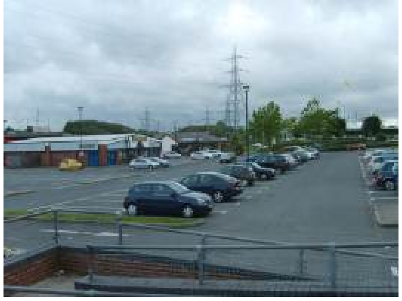
Keyway Retail Park © Gordon Griffiths (cc-by-sa/2.0)

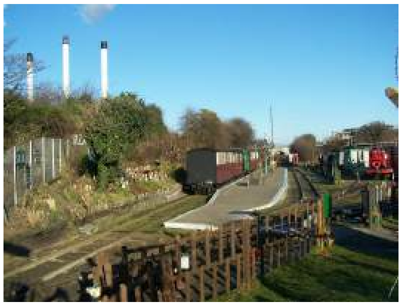
Kemsley Down Railway Station © David Anstiss (cc-by-sa/2.0)

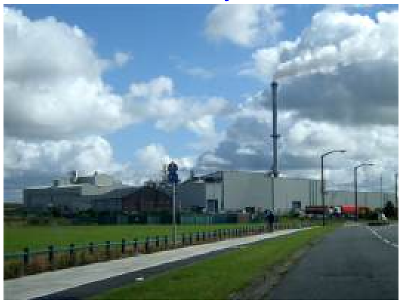
Sonae Factory, Kirkby © Tom Pennington (cc-by-sa/2.0)

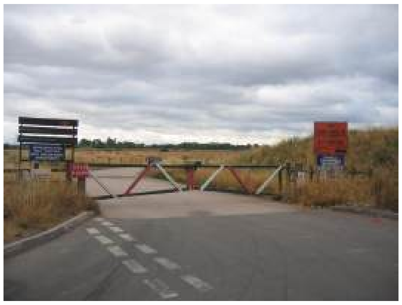
Entrance to Coleshill Quarry © David Stowell (cc-by-sa/2.0)

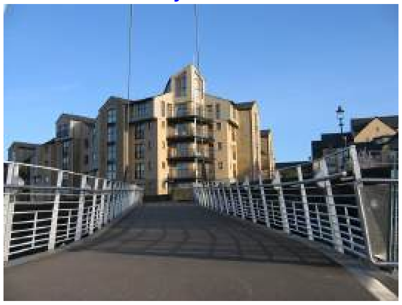
Modern flats © Ian Taylor (cc-by-sa/2.0)

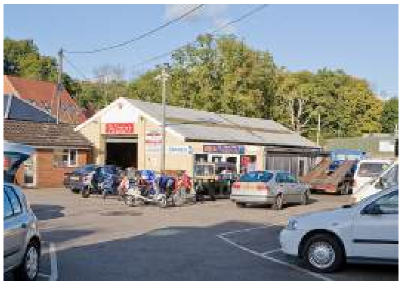
H. J. Dunford & Sons (motors) Ltd, Main Road © Peter Facey (cc-by-sa/2.0)

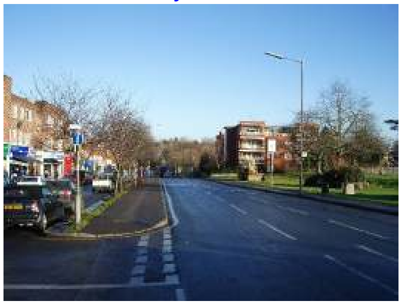
High Road, Chigwell © John Davies (cc-by-sa/2.0)

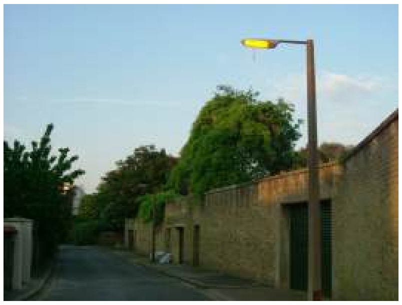
British Grove South, W4 © Phillip Perry (cc-by-sa/2.0)

3.26 Cloud

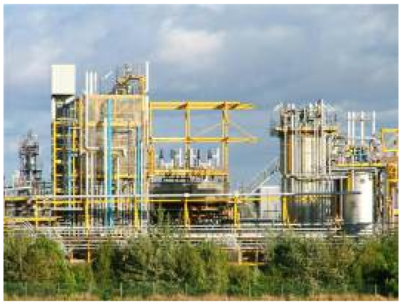

Hydro Polymers PVC Plant © Mick Garratt (cc-by-sa/2.0)

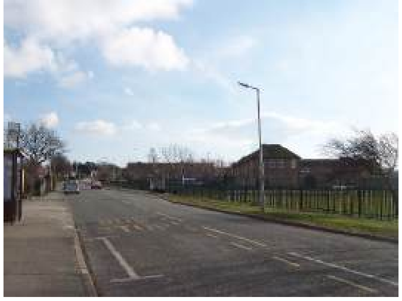

Pensby High School for Boys, Irby Road © Sue Morgan (cc-by-sa/2.0)

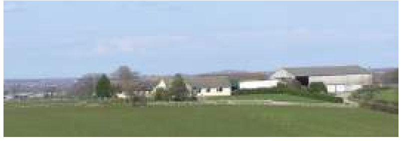

Barton Grange Farm © Hugh Mortimer (cc-by-sa/2.0)

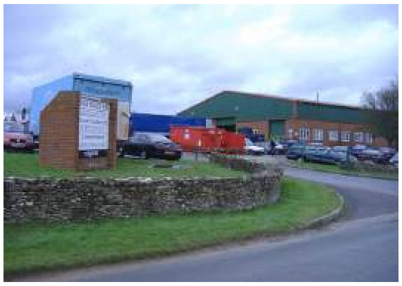

Kemble business park © Roger Cornfoot (cc-by-sa/2.0)

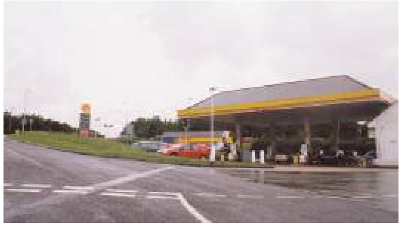

Bere Regis: garage at the end of the A31 © Chris Downer (cc-by-sa/2.0)

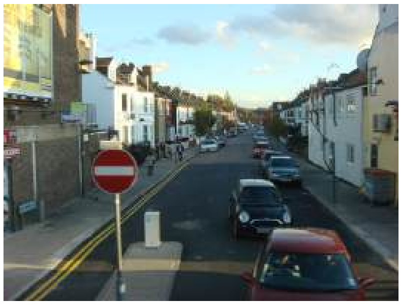

Oak Grove © Oxyman (cc-by-sa/2.0)

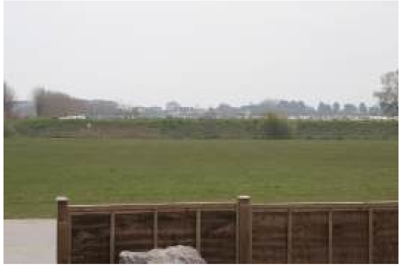

Selsey © Brendan and Ruth McCartney (cc-by-sa/2.0)

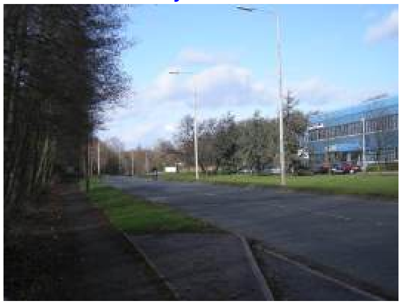

Halesfield 1 © Row17 (cc-by-sa/2.0)

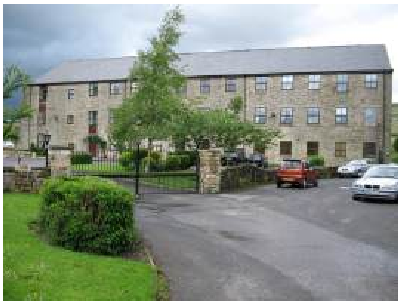

The Meadows at Red Lumb © Paul Anderson (cc-by-sa/2.0)

3.27 Commercial Building

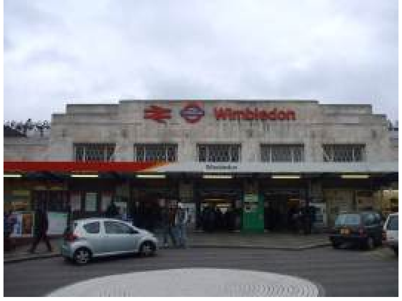

Wimbledon Station © Phillip Perry (cc-by-sa/2.0)

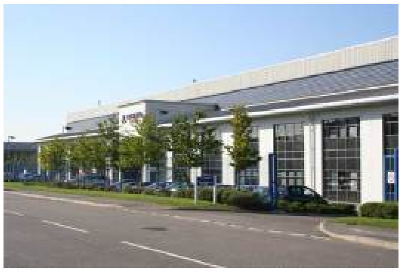

Brantano Footwear Building © Les Carruthers (cc-by-sa/2.0)

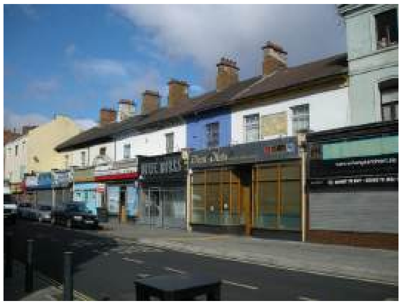

Coventry-Far Gosford Street © Ian Rob (cc-by-sa/2.0)

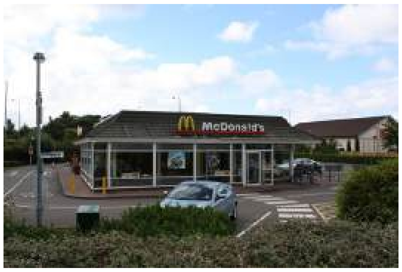

McDonald's Restaurant, Bermuda Park © Richard Kay (cc-by-sa/2.0)

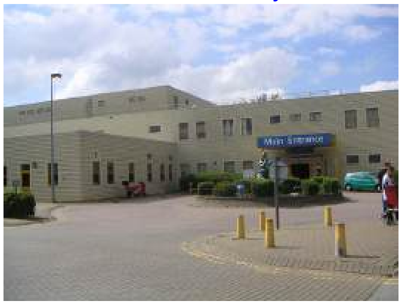

Milton Keynes General Hospital © Mr Biz (cc-by-sa/2.0)

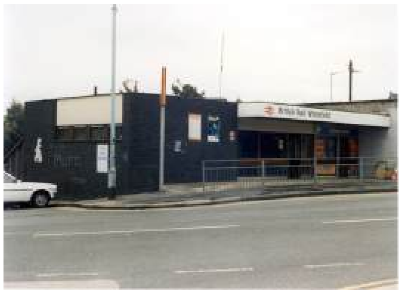

Whitefield station building 1988 © Peter Whatley (cc-by-sa/2.0)

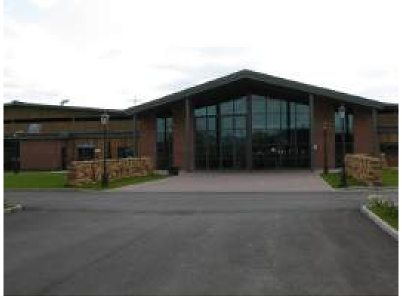

Thirsk Auction Mart © Gordon Hatton (cc-by-sa/2.0)

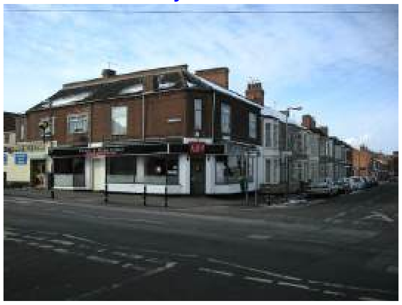

Rugby-Murray Road © Ian Rob (cc-by-sa/2.0)

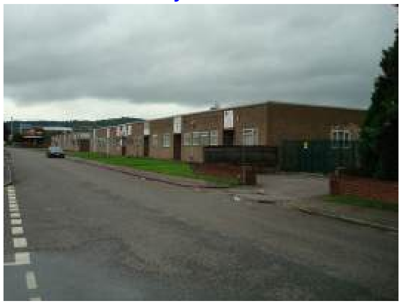

Vestry Road, Sevenoaks © Stacey Harris (cc-by-sa/2.0)

3.28 Concrete Bridge

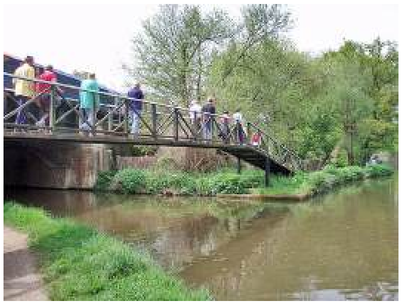

Wey Navigation/ Basingstoke Canal Junction © Andy Stephenson (cc-by-sa/2.0)

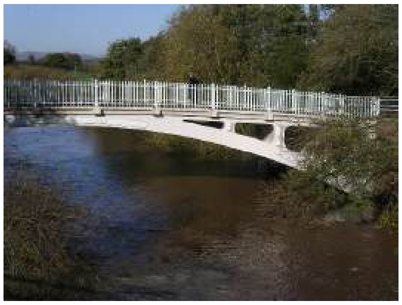

Stanford “old” bridge © Richard Greenwood (cc-by-sa/2.0)

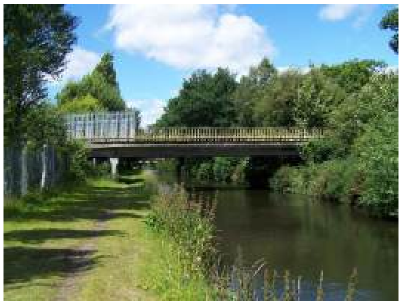

Canal Bridge, Four Ashes © Geoff Pick (cc-by-sa/2.0)

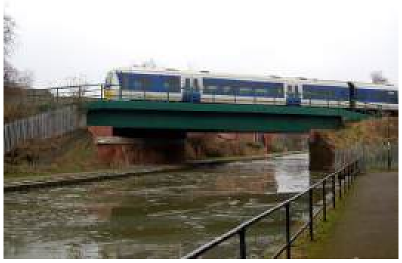

Canal railway bridge, Leamington © Andy F (cc-by-sa/2.0)

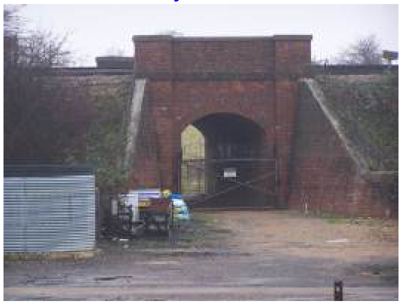

Railbridge over farm track © David Anstiss (cc-by-sa/2.0)

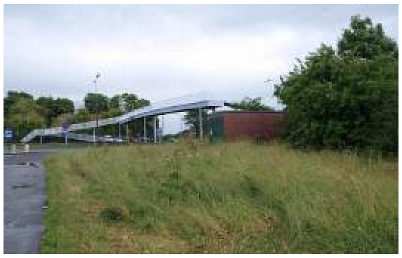

Footbridge crossing the A1 © Steve Fareham (cc-by-sa/2.0)

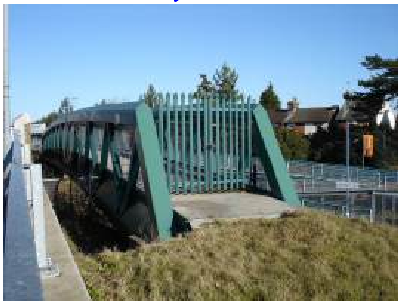

Old footbridge, Derby Road station © Oxymoron (cc-by-sa/2.0)

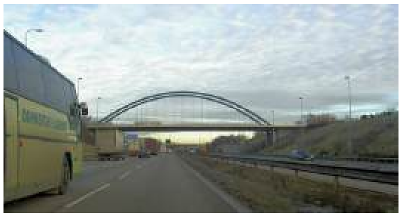

Steel arch bridge over the M42 © Steve Fareham (cc-by-sa/2.0)

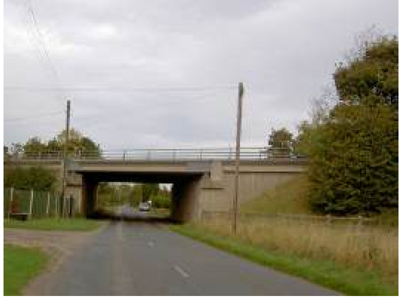

A1 motorway over Springwell Lane © Steve Fareham (cc-by-sa/2.0)

3.29 Condominium

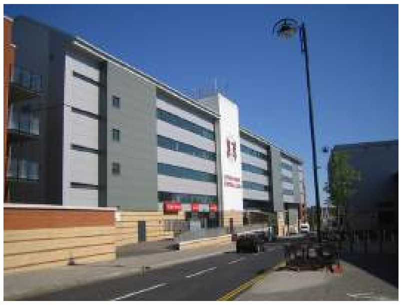

Leyton Orient Football Club © Nigel Cox (cc-by-sa/2.0)

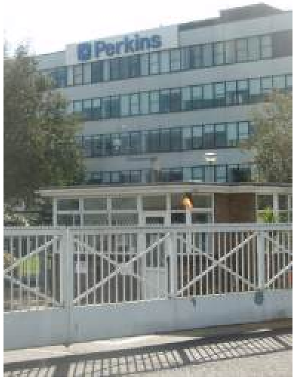

Perkins Engines © Michael Trolove (cc-by-sa/2.0)

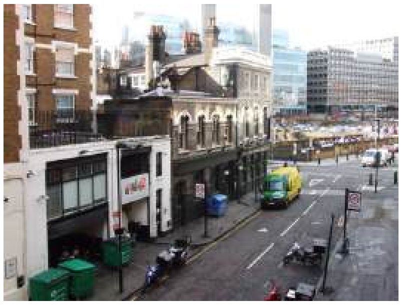

Warner Street, near Clerkenwell © Chris Whippet (cc-by-sa/2.0)

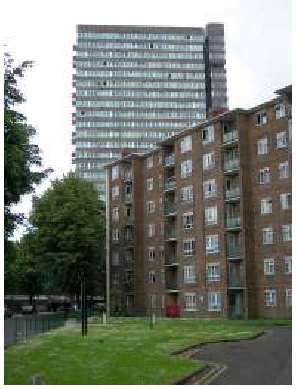

Bradley House and Maydew House © Danny P Robinson (cc-by-sa/2.0)

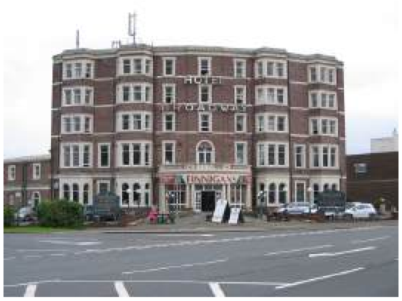

Hotel Broadway © Ian Taylor (cc-by-sa/2.0)

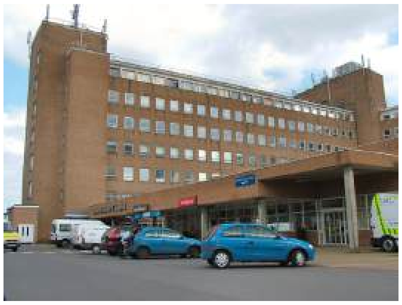

Queen Elizabeth II Hospital W.G.C. © Melvyn Cousins (cc-by-sa/2.0)

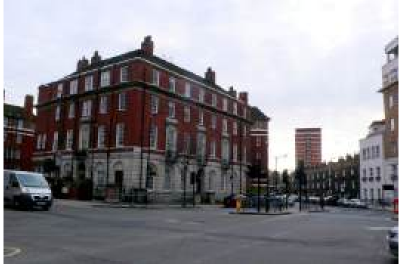

Junction of Ebury Bridge and Ebury Bridge Rd © Nigel Mykura (cc-by-sa/2.0)

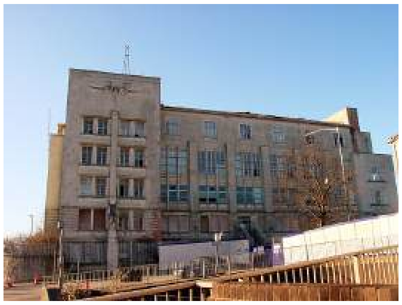

New Filton House © Linda Bailey (cc-by-sa/2.0)

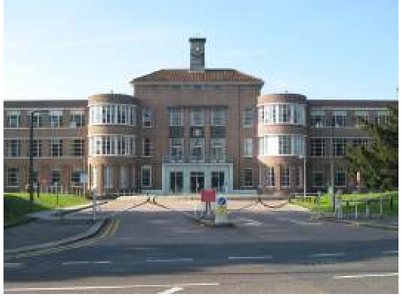

Watford: West Herts College, Hempstead Road campus © Nigel Cox (cc-by-sa/2.0)

3.30 Cottage

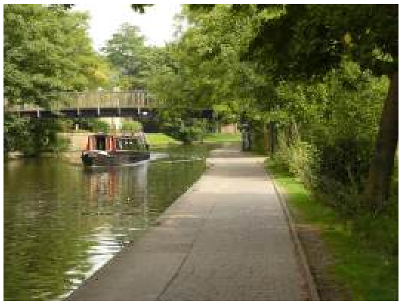

Nottingham Canal © Andy Jamieson (cc-by-sa/2.0)

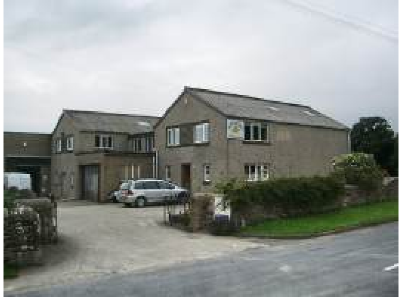

Honeycomb Company, Galgate © Alexander P Kapp (cc-by-sa/2.0)

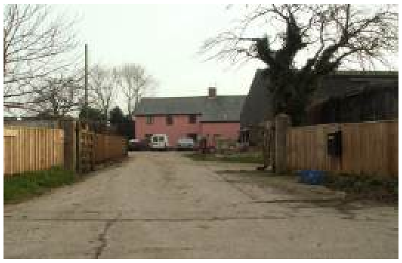

A view of Grange Farm © Robert Edwards (cc-by-sa/2.0)

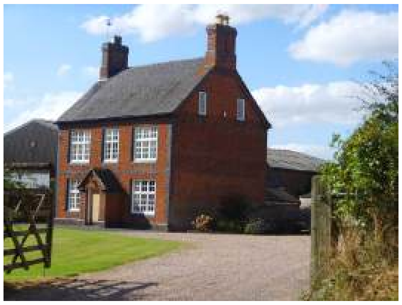

Burleigh Farm © A Holmes (cc-by-sa/2.0)

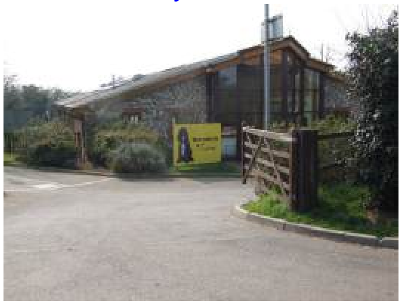

Dog's Trust, Rehoming Centre © Ian Robertson (cc-by-sa/2.0)

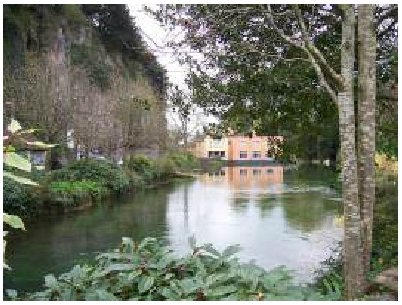

Cheddar Gorge © Pam Goodey (cc-by-sa/2.0)

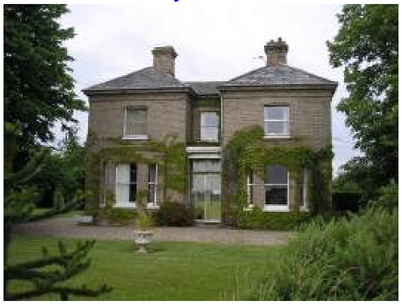

Morehams Hall © NA (cc-by-sa/2.0)

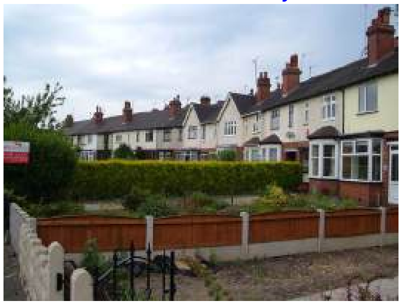

Crescent, Dimsdale Parade East, Wolstanton © Derek Harper (cc-by-sa/2.0)

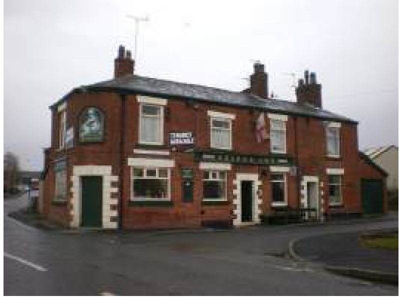

Bridge Inn, Moorhey Street © Alexander P Kapp (cc-by-sa/2.0)

3.31 Cumulus

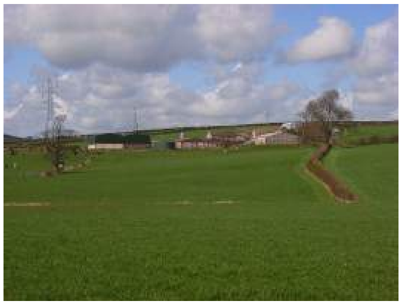

Bank House © Andrew Smith (cc-by-sa/2.0)

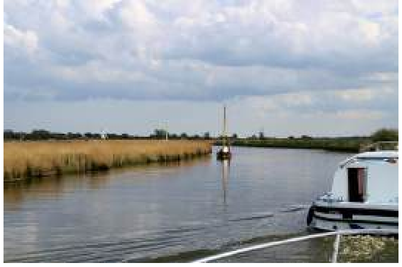

River Thurne above Thurne © Pierre Terre (cc-by-sa/2.0)

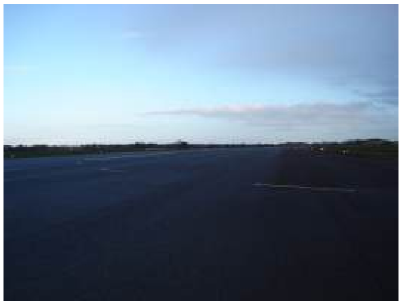

Main Runway, RAF Leeming © Frank Glover (cc-by-sa/2.0)

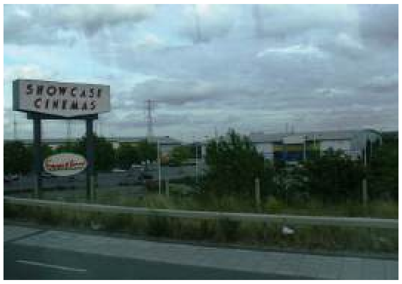

Showcase Cinemas, Barking © Phillip Perry (cc-by-sa/2.0)

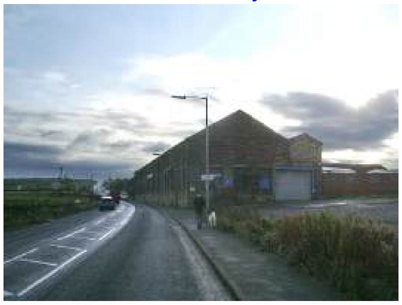

Denholme Velvet © Alexander P Kapp (cc-by-sa/2.0)

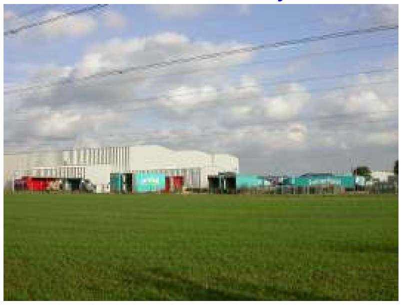

Park Farm Industrial Estate, South © Kokai (cc-by-sa/2.0)

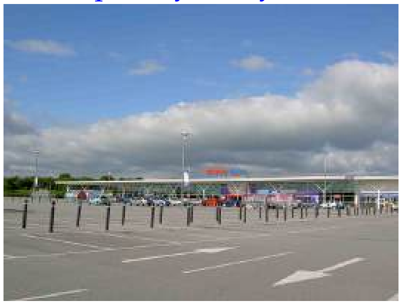

Early morning in Tesco's car park © Steve Fareham (cc-by-sa/2.0)

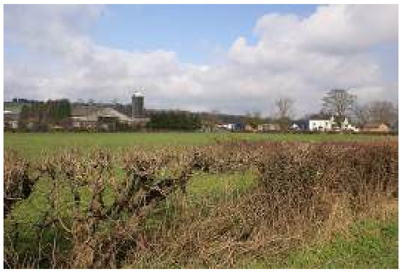

Holme Farm, Hangingbridge © Nikki Mahadevan (cc-by-sa/2.0)

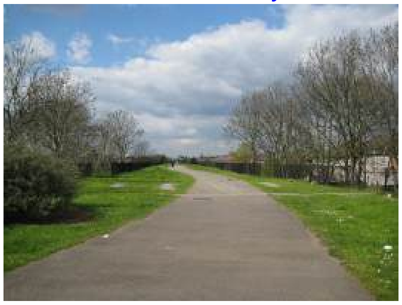

East Ham: The Greenway and the Northern Outfall Sewer © Nigel Cox (cc-by-sa/2.0)

3.32 Daytime

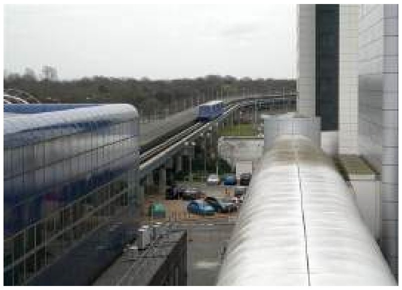

Monorail At Gatwick Airport © Mary and Angus Hogg (cc-by-sa/2.0)

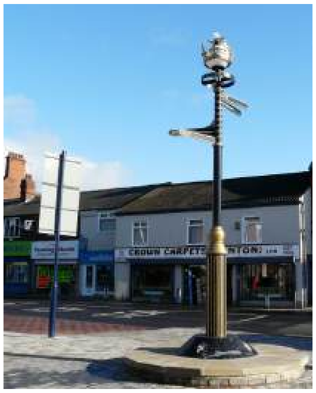

The Crown Pole © Gerald England (cc-by-sa/2.0)

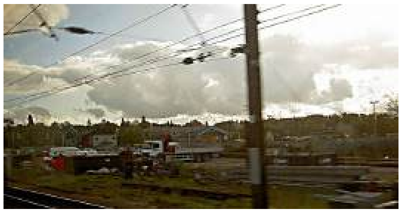

Network Rail maintenance north of Hitchin station © Steve Fareham (cc-by-sa/2.0)

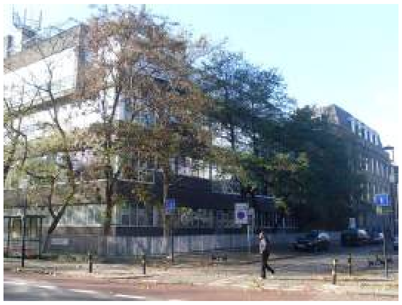

Vauxhall Telephone Exchange © David Hillas (cc-by-sa/2.0)

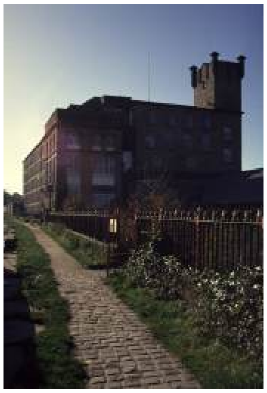

Adelphi Mill, Bollington © Chris Allen (cc-by-sa/2.0)

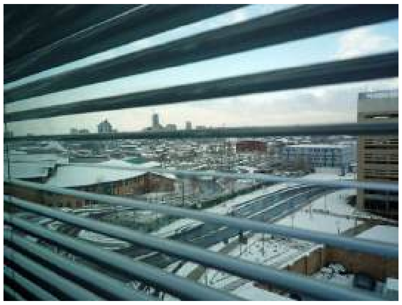

Outlook from Ward 82 Bexley Wing, St James Hospital © Julian Paren (cc-by-sa/2.0)

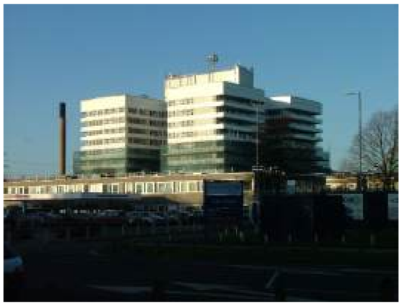

Lister Hospital, Stevenage. © Robin Hall (cc-by-sa/2.0)

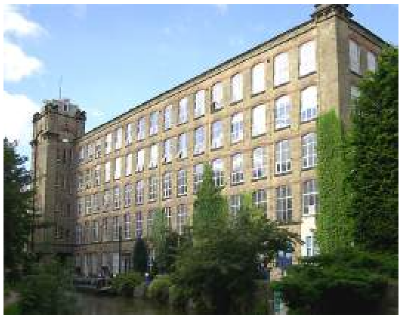

Clarence Mill, Bollington, Cheshire © Roger D Kidd (cc-by-sa/2.0)

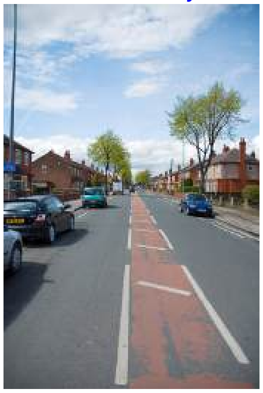

Holden Road, Leigh © Dave Green (cc-by-sa/2.0)

3.33 Dirt Road

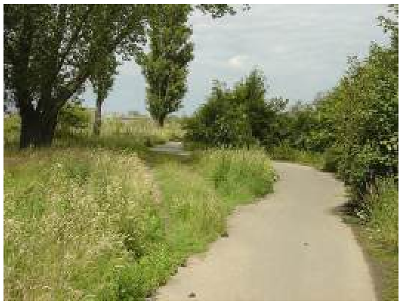

Path to the foreshore © Alan Murray-Rust (cc-by-sa/2.0)

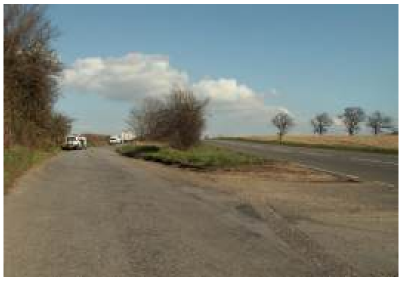

A Lay-by along the A.143 © Robert Edwards (cc-by-sa/2.0)

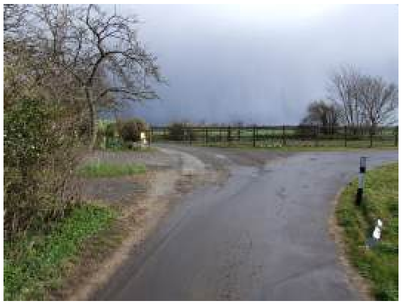

Paddock and Stables Entrance, Kemp's Corner © Ian Robertson (cc-by-sa/2.0)

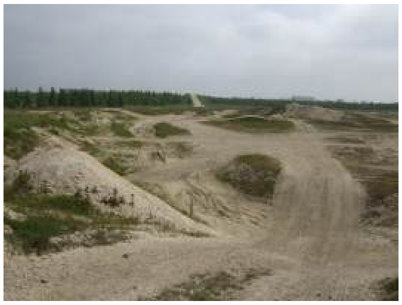

Cross Country Driving Circuit © Siobhan Brennan-Raymond (cc-by-sa/2.0)

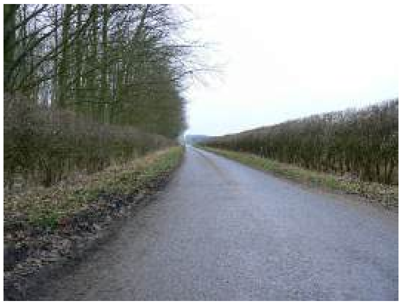

The road to Down Ampney © Brian Robert Marshall (cc-by-sa/2.0)

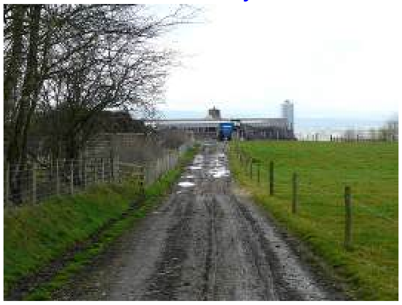

Hilltop Farm © Rose and Trev Clough (cc-by-sa/2.0)

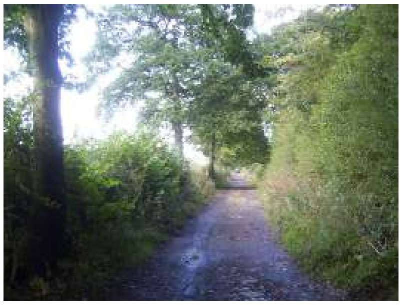

The Lymes Road © Iain McDonald (cc-by-sa/2.0)

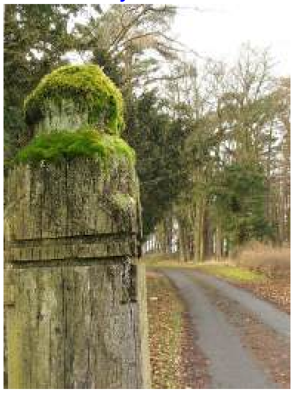

Old Gatepost to Newton Hall © Christine Westerback (cc-by-sa/2.0)

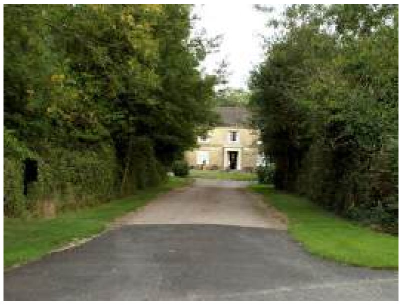

House at High Street Green, Suffolk © Robert Edwards (cc-by-sa/2.0)

3.34 Downtown

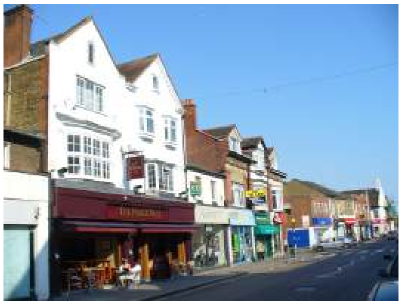

High Street, Walton-on-Thames © Colin Smith (cc-by-sa/2.0)

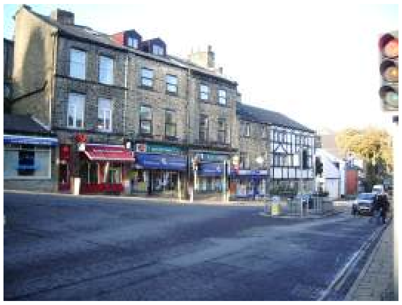

Shops on Halifax Road, Ripponden © Alexander P Kapp (cc-by-sa/2.0)

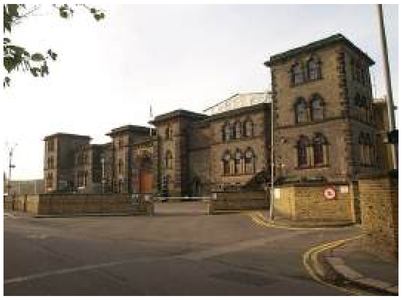

Wandsworth Prison © Derek Harper (cc-by-sa/2.0)

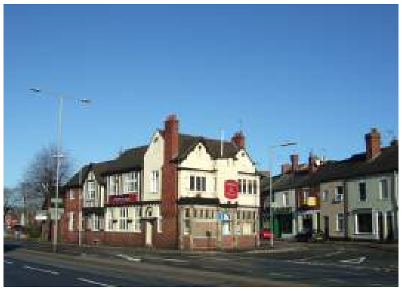

The Wagon and Horses © Simon Huguet (cc-by-sa/2.0)

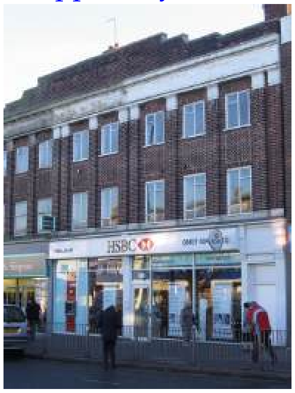

HSBC Bank Northfield. Sorting code 40-11-20 © Roy Hughes (cc-by-sa/2.0)

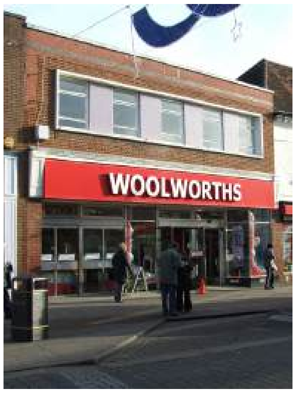

Woolworths Sudbury © Keith Evans (cc-by-sa/2.0)

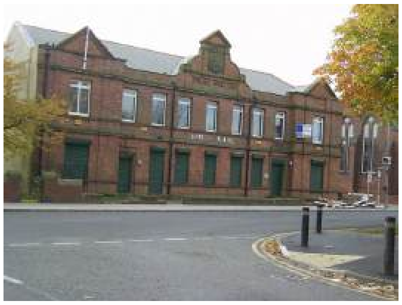

Police Station © george hurrell (cc-by-sa/2.0)

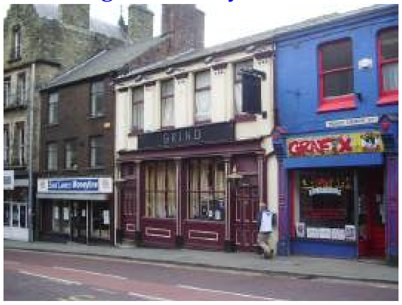

Grind, Church Street, Blackburn © Alexander P Kapp (cc-by-sa/2.0)

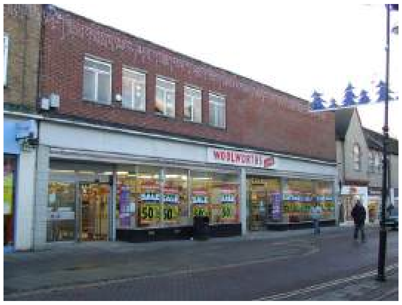

Woolworths Haverhill © Keith Evans (cc-by-sa/2.0)

3.35 Driveway

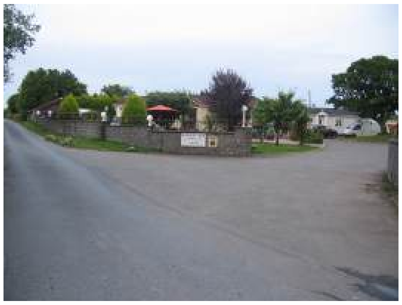

Mayfair Park © Phil Williams (cc-by-sa/2.0)

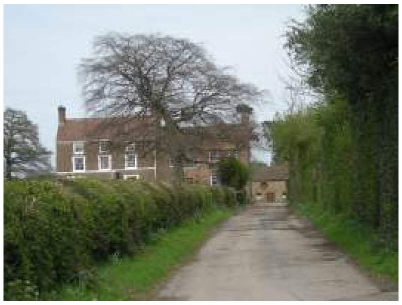

Drive to The Hills Farm © Row17 (cc-by-sa/2.0)

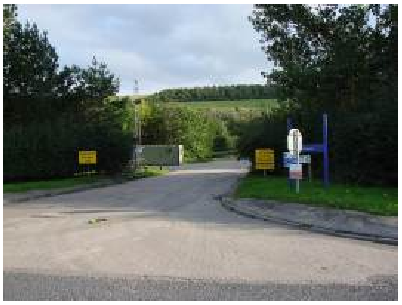

Entrance to Gale Common ash disposal site. © Bill Henderson (cc-by-sa/2.0)

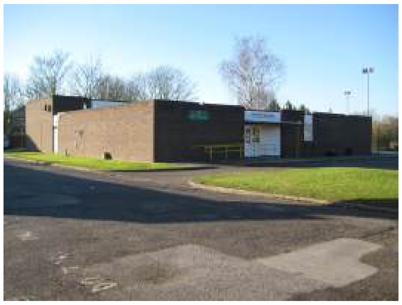

Shepperton Youth Centre © Nigel Cox (cc-by-sa/2.0)

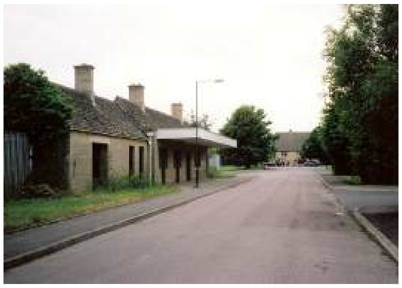

Bourton on the water Station © Raymond Knapman (cc-by-sa/2.0)

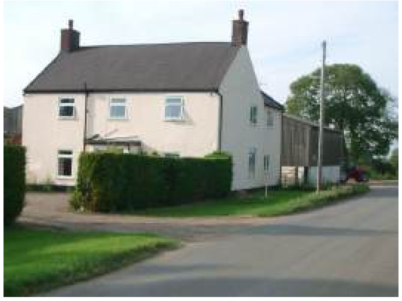

Telegraph Farm © Andrew Longton (cc-by-sa/2.0)

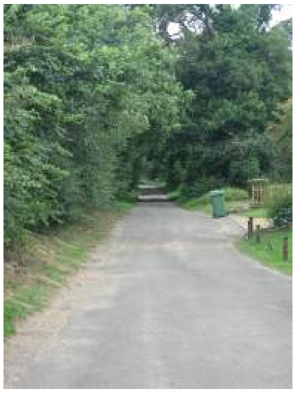

Bin hangin' around © Alison Rawson (cc-by-sa/2.0)

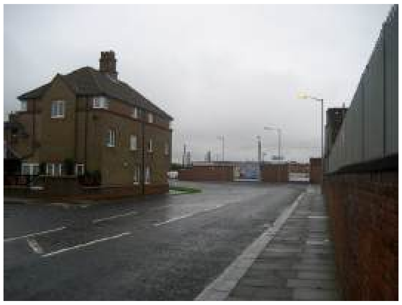

Outside Wimbourne Quay © Chris Heaton (cc-by-sa/2.0)

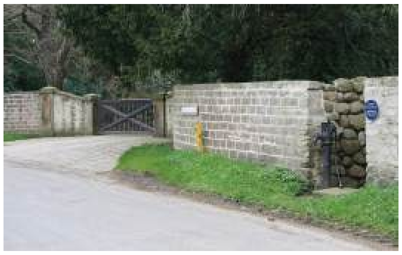

Village Pump © David Rogers (cc-by-sa/2.0)

3.36 Dusk

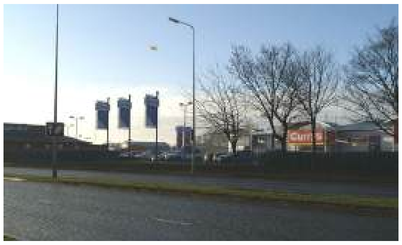
Alban Retail Park, beside the A49 © David Long (cc-by-sa/2.0)

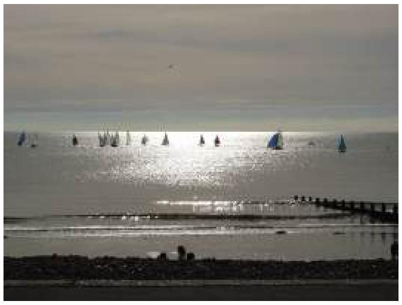
Autumn sailing at Felpham (3) © Roger Brooks (cc-by-sa/2.0)

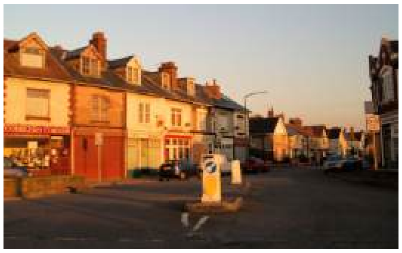
'Cobblers Corner' © Steve Fareham (cc-by-sa/2.0)

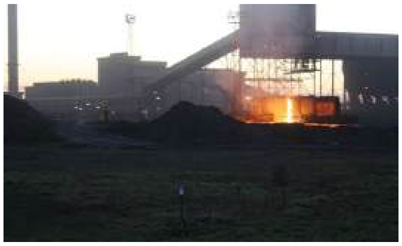
Emptying the Slag © Mick Garratt (cc-by-sa/2.0)

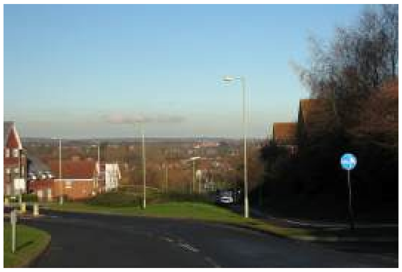
Bucksford Lane, Ashford, Kent © Oast House Archive (cc-by-sa/2.0)

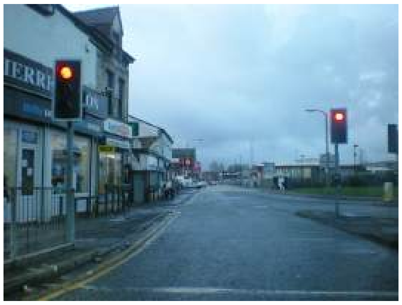
Broughton Street, Manchester © Alexander P Kapp (cc-by-sa/2.0)

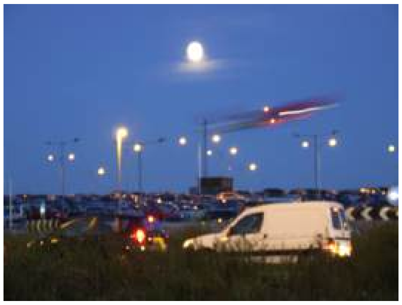
Fly me to the moon © Thomas Nugent (cc-by-sa/2.0)

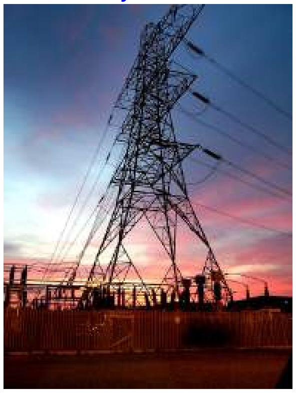
Morning glory © R lee (cc-by-sa/2.0)

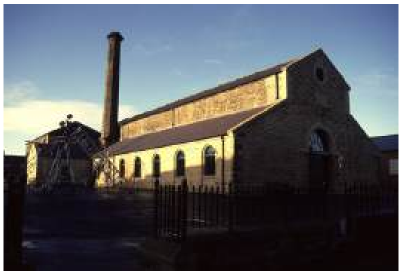
Elsecar Heritage Centre © Chris Allen (cc-by-sa/2.0)

3.37 Ecoregion

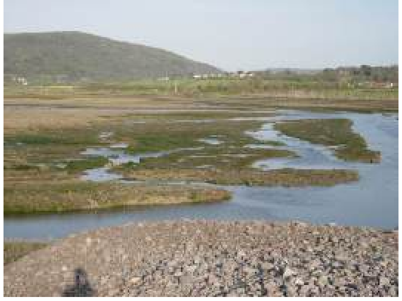

Porlock Saltings © Hugh Venables (cc-by-sa/2.0)

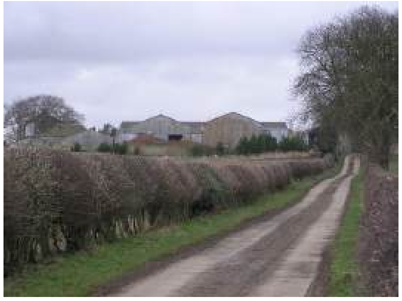

Lane to Hutton Magna © Hugh Mortimer (cc-by-sa/2.0)

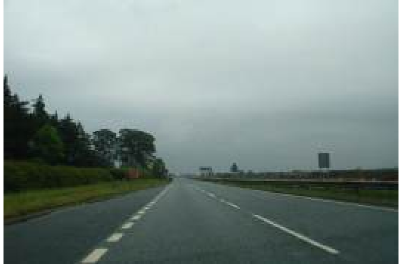

A74 - The last 10km © Raymond Okonski (cc-by-sa/2.0)

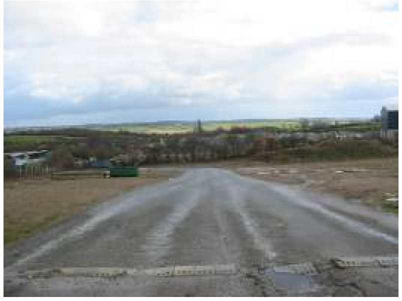

Tip and Industrial Area on Cotes Park Industrial Estate © Mike Bardill (cc-by-sa/2.0)

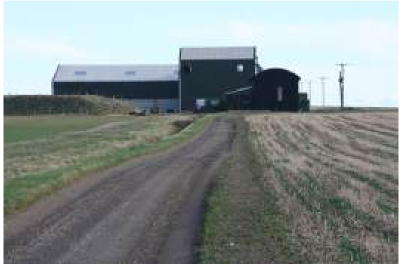

Top Farm © Duncan Grey (cc-by-sa/2.0)

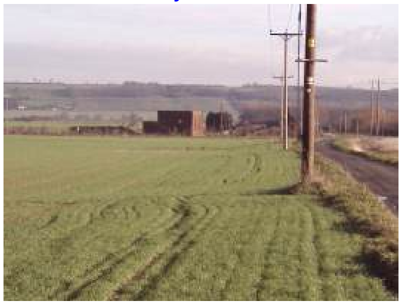

Scotney Farm Lane © fred roberts (cc-by-sa/2.0)

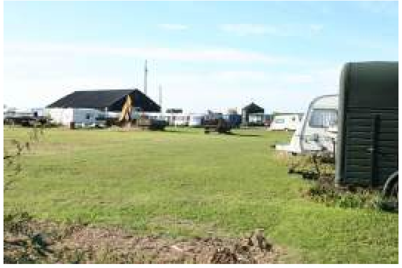

Maarnford Farm, used as a caravan store © Duncan Grey (cc-by-sa/2.0)

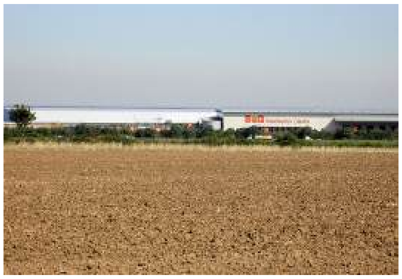

DIY anyone? © Steve Fareham (cc-by-sa/2.0)

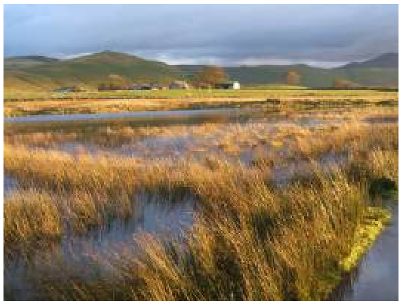

Baggra Yeat Farm © David Brown (cc-by-sa/2.0)

3.38 Electrical Supply

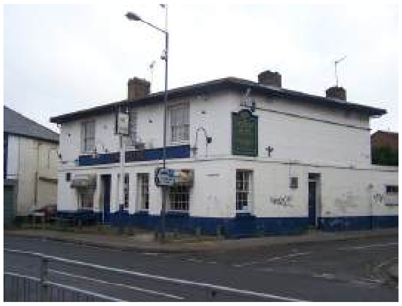

The Rose Pub, Northfleet © David Anstiss (cc-by-sa/2.0)

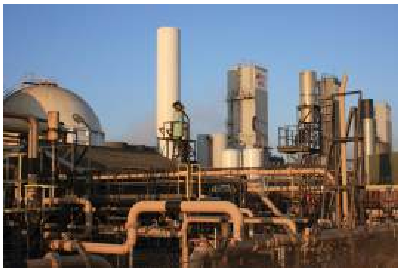

B.O.C Works © Mick Garratt (cc-by-sa/2.0)

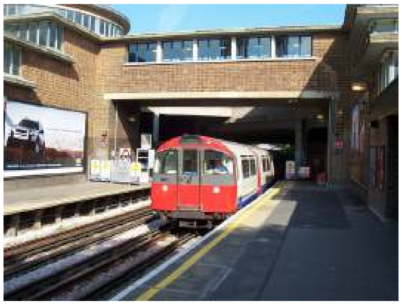

Park Royal station © Phillip Perry (cc-by-sa/2.0)

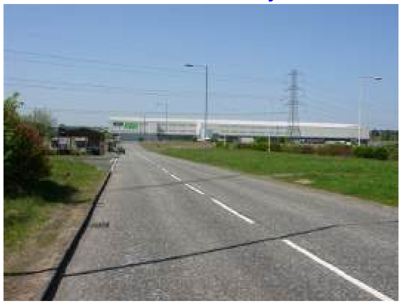

ASDA distribution warehouse © Brian Abbott (cc-by-sa/2.0)

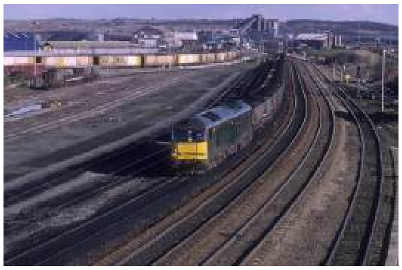

Coal for the Steel plant at Scunthorpe © roger geach (cc-by-sa/2.0)

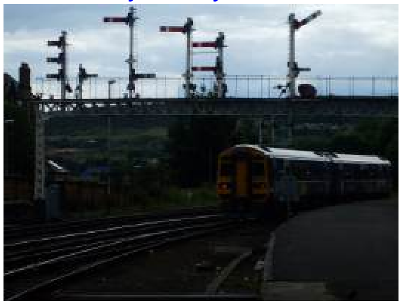

Scarborough station signal gantry © Ashley Dace (cc-by-sa/2.0)

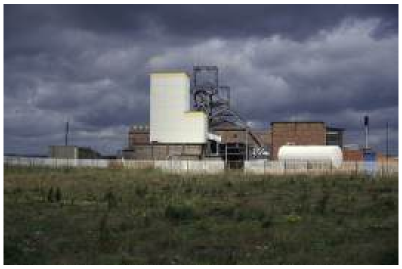

Ellington Colliery © Chris Allen (cc-by-sa/2.0)

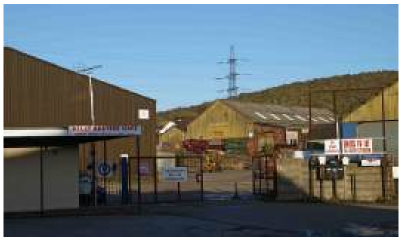

Belly Busters caf © Steve Fareham (cc-by-sa/2.0)

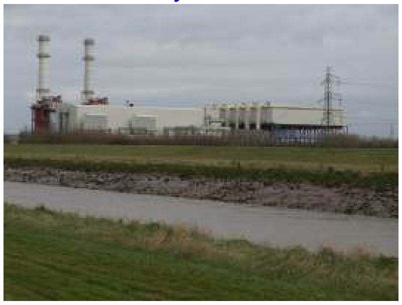

Power Station © Ian Simons (cc-by-sa/2.0)

3.39 Electricity

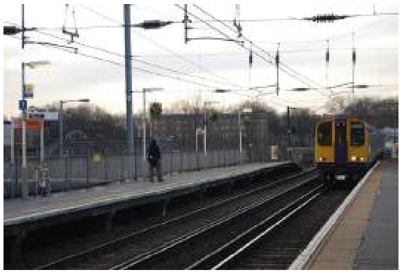

Stratford train arriving at Hackney Wick Station © N Chadwick (cc-by-sa/2.0)

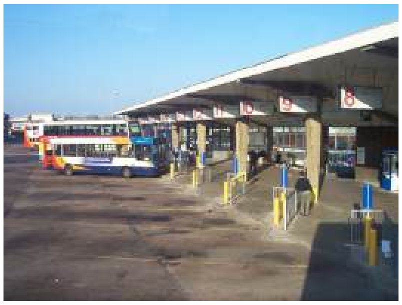

Exeter : Exeter Bus Station © Lewis Clarke (cc-by-sa/2.0)

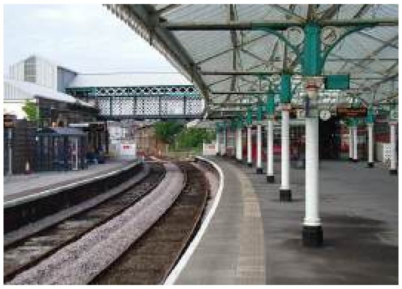

Bridlington Station © Paul Glazzard (cc-by-sa/2.0)

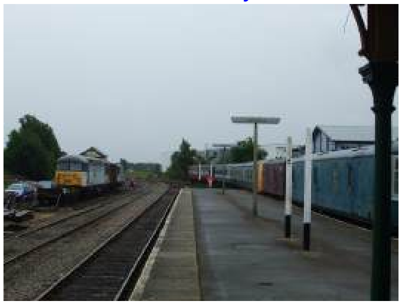

Dereham station © Ashley Dace (cc-by-sa/2.0)

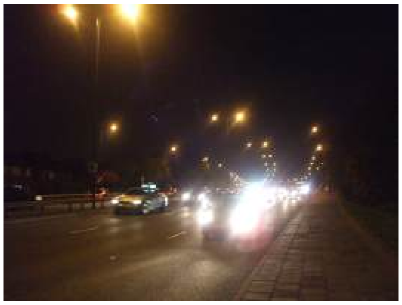

Western Avenue © Phillip Perry (cc-by-sa/2.0)

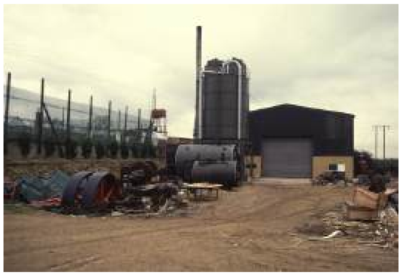

Markham Grange power house © Chris Allen (cc-by-sa/2.0)

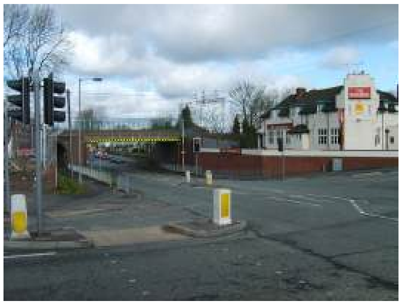

Fordhouse Road Junction © Gordon Griffiths (cc-by-sa/2.0)

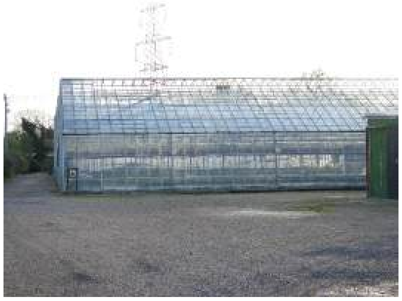

Greenhouses © Hugh Venables (cc-by-sa/2.0)

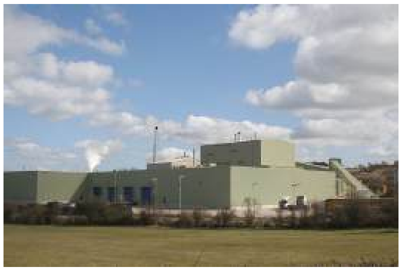

Gypsum works © Alan Murray-Rust (cc-by-sa/2.0)

3.40 Engineering

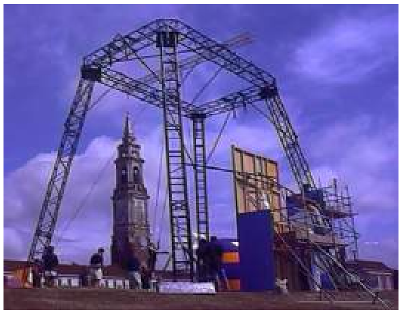

It's a Knock Out set frames the school tower © John Goldsmith (cc-by-sa/2.0)

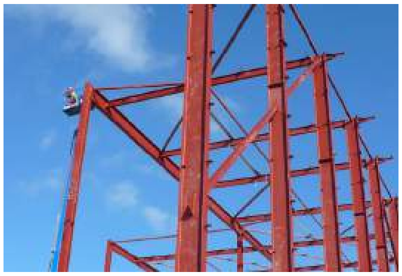

Erection of steel Frame Building, Yeovil (3) © Nigel Mykura (cc-by-sa/2.0)

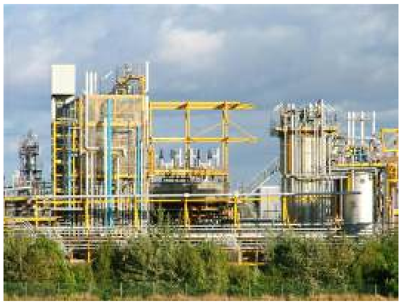

Hydro Polymers PVC Plant © Mick Garratt (cc-by-sa/2.0)

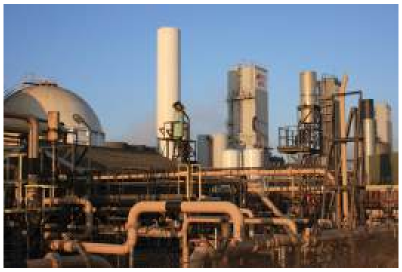

B.O.C Works © Mick Garratt (cc-by-sa/2.0)

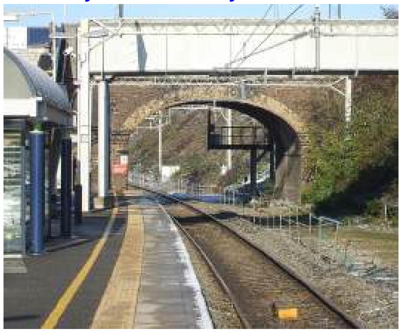

Tring Station - Station Road Bridge, Eastern Arch © Rob Farrow (cc-by-sa/2.0)

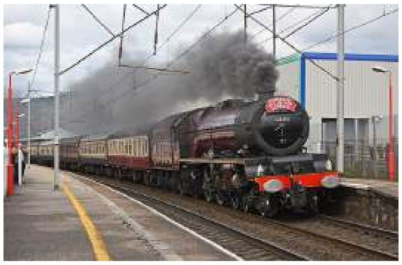

Princess Elizabeth at Penrith © Alan Taylor (cc-by-sa/2.0)

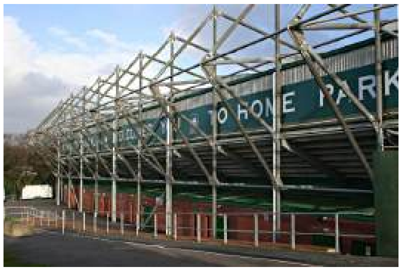

Home Park, the home of Plymouth Argyle Football Club © Tony Atkin (cc-by-sa/2.0)

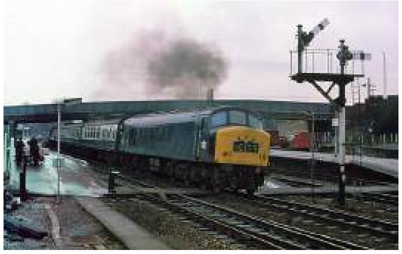

St. Pancras Bound © Martin Addison (cc-by-sa/2.0)

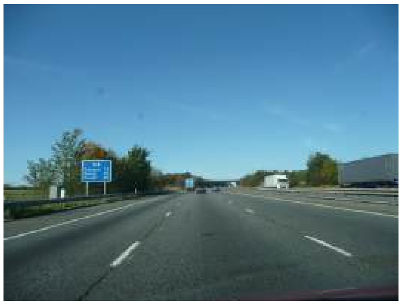

Wiltshire : The M4 Motorway © Lewis Clarke (cc-by-sa/2.0)

3.41 Evening

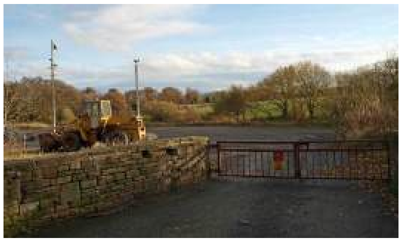

Kirklees Pinfold Storage depot © Steve Fareham (cc-by-sa/2.0)

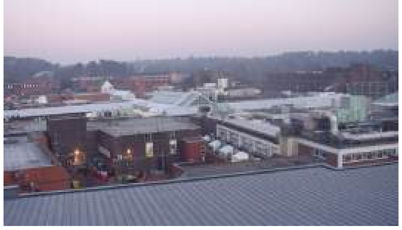

Aerial view from top storey of The Atrium looking across Central Camberley (including Main Square) © Roger (cc-by-sa/2.0)

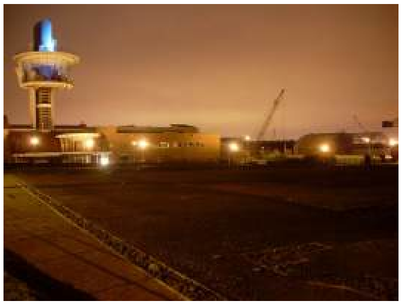

Museum and viewing tower, Segedunum © Stephen Sweeney (cc-by-sa/2.0)

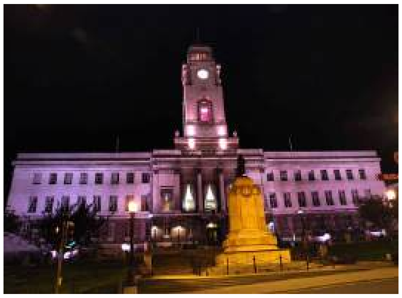

Barnsley Town Hall front elevation © Steve Fareham (cc-by-sa/2.0)

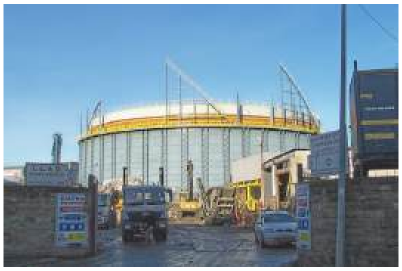

Neepsend Gasometer Deflated, Neepsend Lane, Sheffield © Terry Robinson (cc-by-sa/2.0)

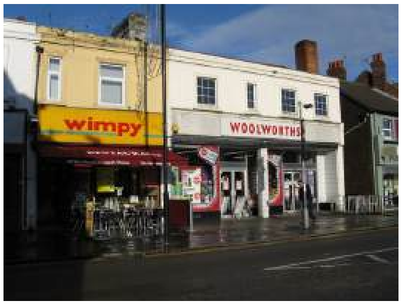

Woolworth's, Coulsdon, Surrey © Dr Neil Clifton (cc-by-sa/2.0)

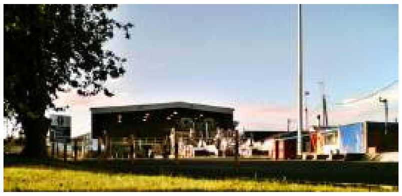

Frozen Food Factory at Autby near North Thoresby © Bob Emm (cc-by-sa/2.0)

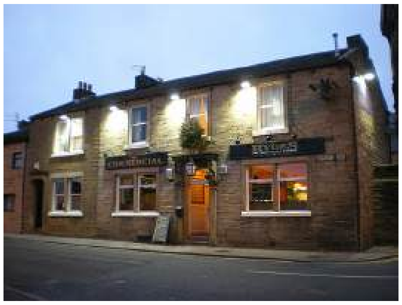

The Commercial, Briercliffe Road © Alexander P Kapp (cc-by-sa/2.0)

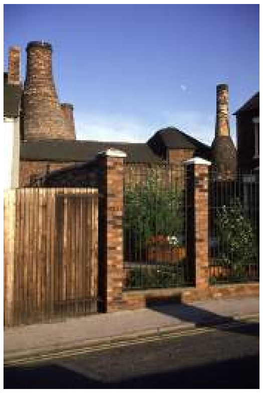

Gladstone Pottery Museum © Chris Allen (cc-by-sa/2.0)

3.42 Event

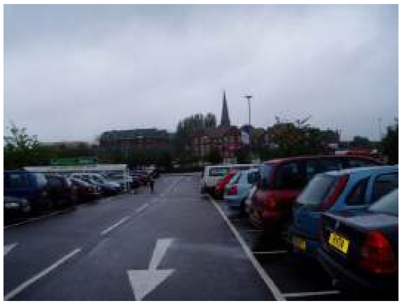

Tesco car park, Uttoxeter © Eirian Evans (cc-by-sa/2.0)

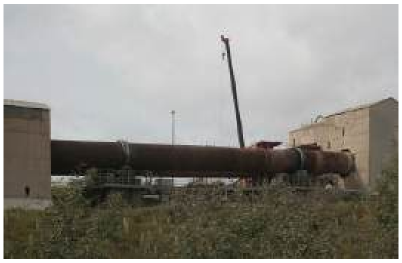

Rotary furnace at Steetley Dolomite © Alan Murray-Rust (cc-by-sa/2.0)

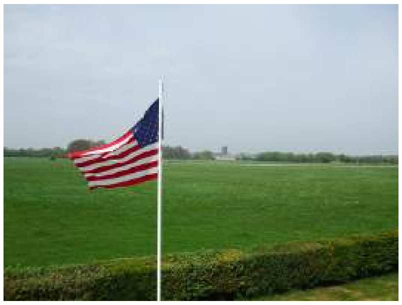

The American flag at Seething airfield © Ashley Dace (cc-by-sa/2.0)

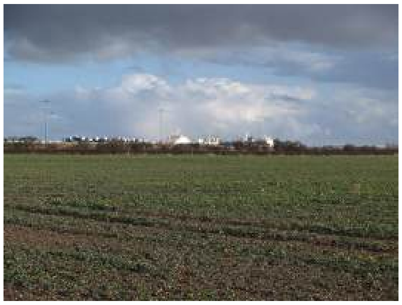

Looking towards the Humber © David Wright (cc-by-sa/2.0)

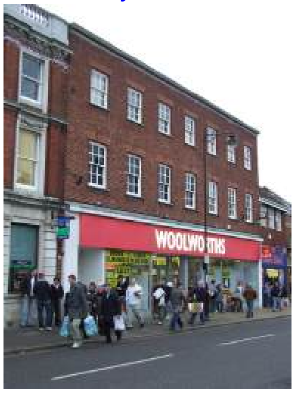

Woolworths Dereham © Keith Evans (cc-by-sa/2.0)

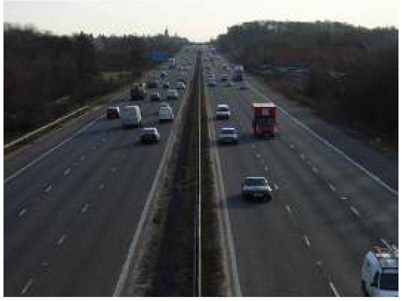

The M3, Hartley Wintney © Andrew Smith (cc-by-sa/2.0)

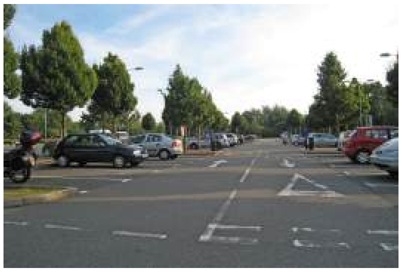

Car Park at Motorway Services © Oast House Archive (cc-by-sa/2.0)

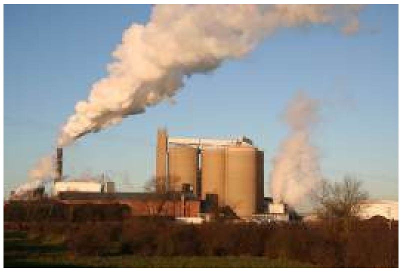

Newark Sugar Factory © Richard Croft (cc-by-sa/2.0)

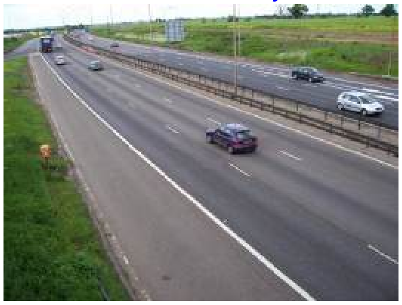

M1 at junction 8 © Rob Hinkley (cc-by-sa/2.0)

3.43 Family Car

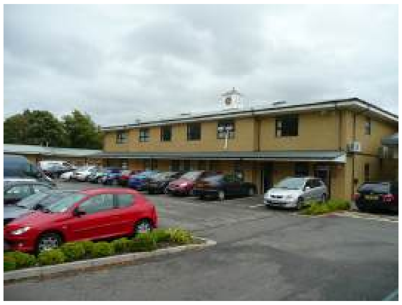

Business units at Cressex Business Park © Jonathan Billinger (cc-by-sa/2.0)

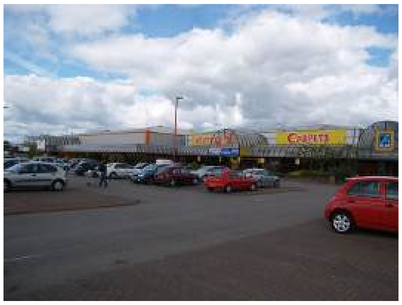

Tees Bay Retail Park © Stephen McCulloch (cc-by-sa/2.0)

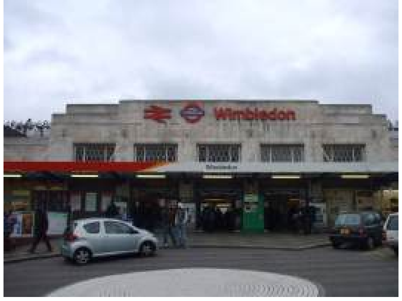

Wimbledon Station © Phillip Perry (cc-by-sa/2.0)

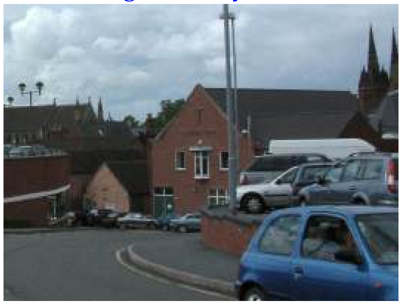

Gresley Row © Gerald England (cc-by-sa/2.0)

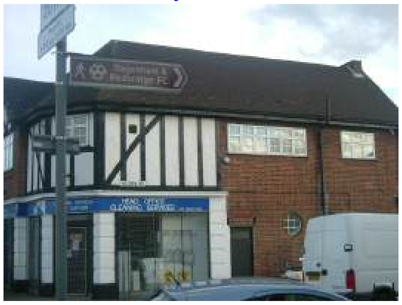

Victoria Road, Dagenham © Phillip Perry (cc-by-sa/2.0)

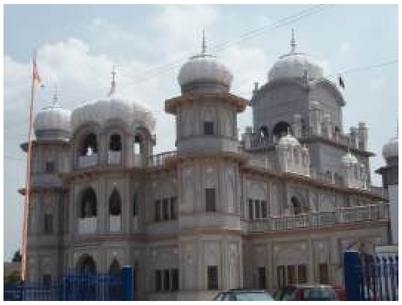

Temple © Richard Schmidt (cc-by-sa/2.0)

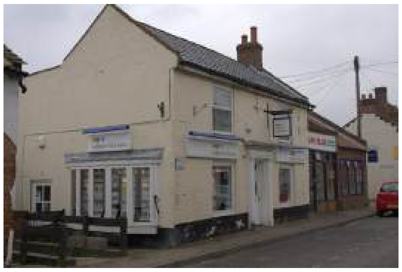

Estate Agents, Stalham High St © Pauline A Marsh (cc-by-sa/2.0)

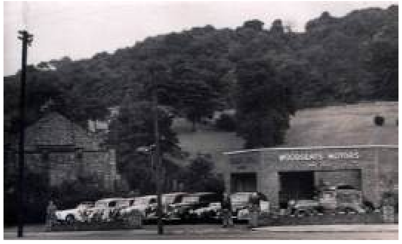

Woodseats Motors Circa 1950 Woodseats Jnc Cobnar Road © Richard Newall (cc-by-sa/2.0)

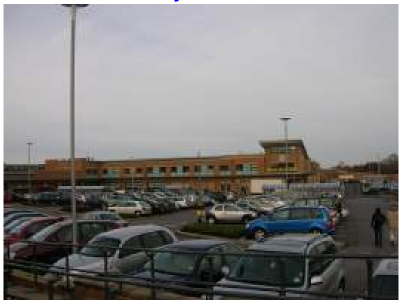

Gloucester, Abbeydale: Morrisons © Alby (cc-by-sa/2.0)

3.44 Field

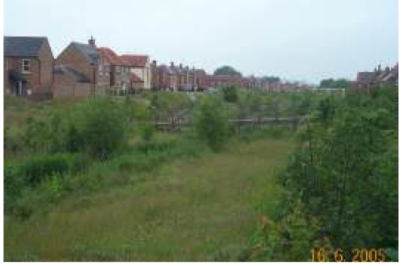

Aylesbury: Bear Brook, Fairford Leys © Nigel Cox (cc-by-sa/2.0)

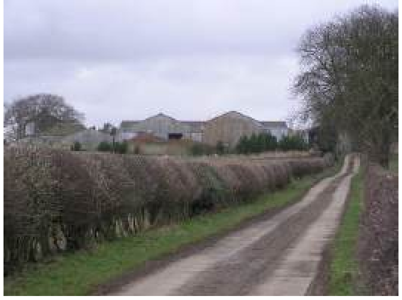

Lane to Hutton Magna © Hugh Mortimer (cc-by-sa/2.0)

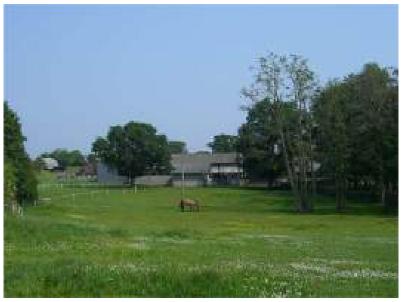

Bridge House Farm © Steve Lewin (cc-by-sa/2.0)

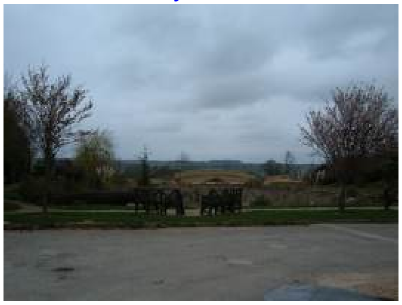

Decorative Bridge & Pond © Mr Biz (cc-by-sa/2.0)

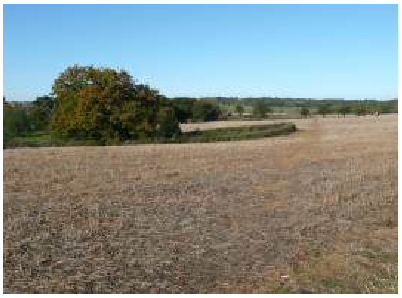

Path across the stubble, Bessingham © Humphrey Bolton (cc-by-sa/2.0)

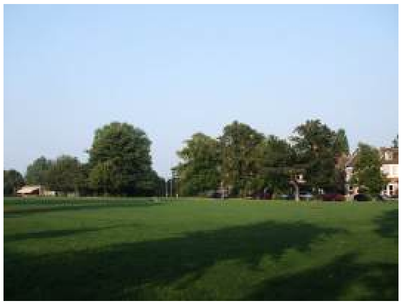

Alexandra Road recreation ground (3) © Linda Craven (cc-by-sa/2.0)

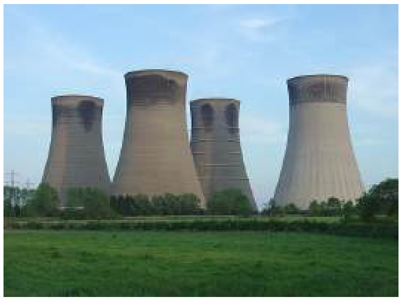

Cooling towers, West Burton Power Station © Alan Murray-Rust (cc-by-sa/2.0)

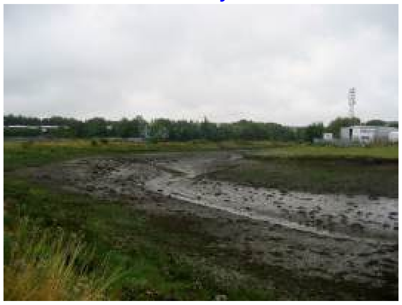

Creek in Cowpen New Town © Chris Heaton (cc-by-sa/2.0)

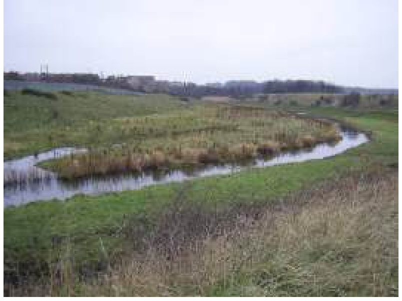

Lyneburn © george hurrell (cc-by-sa/2.0)

3.45 Fixture

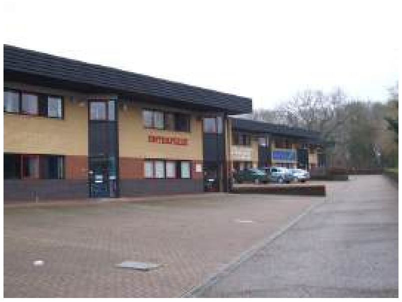

Industrial Units © Ian Robertson (cc-by-sa/2.0)

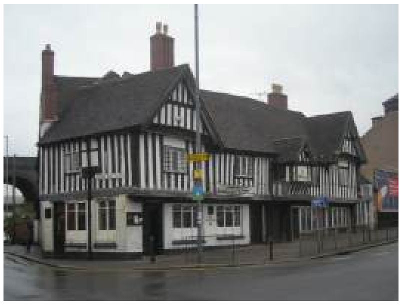

The Old Crown Inn © Row17 (cc-by-sa/2.0)

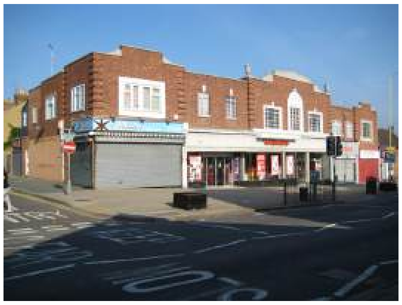

North Watford: Woolworths © Nigel Cox (cc-by-sa/2.0)

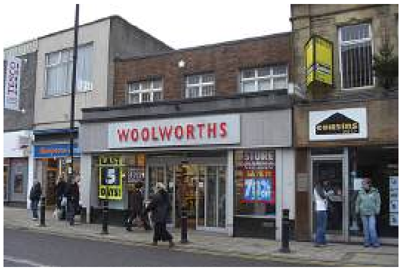

Woolworths, Market Street, Shaw © michael ely (cc-by-sa/2.0)

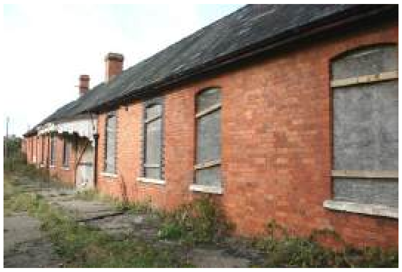

Lydd Station © Mark Duncan (cc-by-sa/2.0)

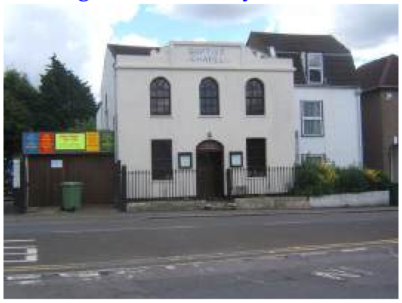

Free Grace Baptist Chapel, Belvedere © Andrew Hill (cc-by-sa/2.0)

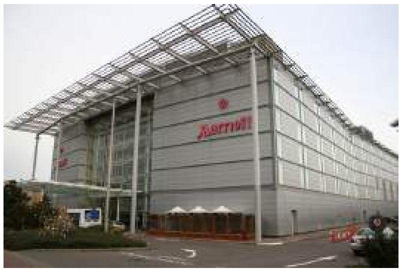

Marriott Hotel © Richard Croft (cc-by-sa/2.0)

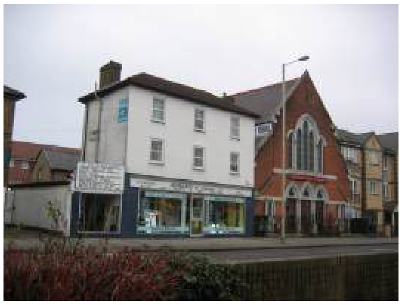

Cheshunt Free Church © Talisman (cc-by-sa/2.0)

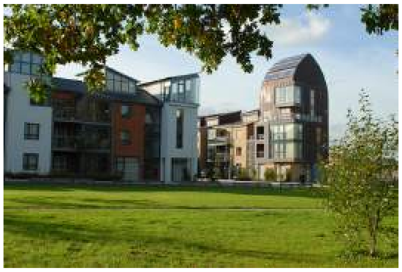

Newhall Farm housing development © John Allen (cc-by-sa/2.0)

3.46 Flower

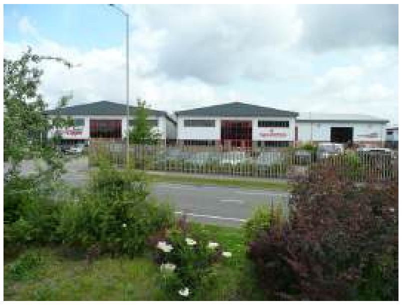

Business units © Jonathan Billinger (cc-by-sa/2.0)

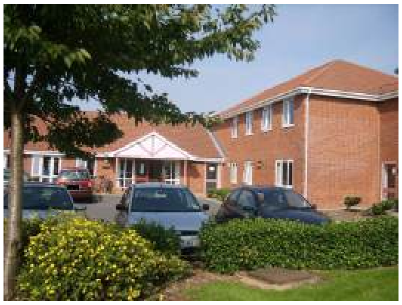

Altham Meadows © Michael Graham (cc-by-sa/2.0)

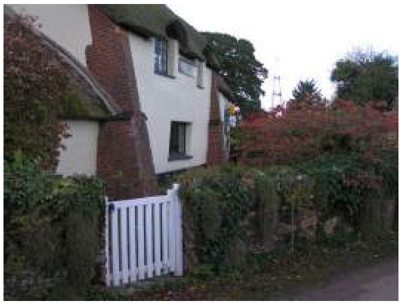

Barton Cross restaurant, Huxham © Rob Purvis (cc-by-sa/2.0)

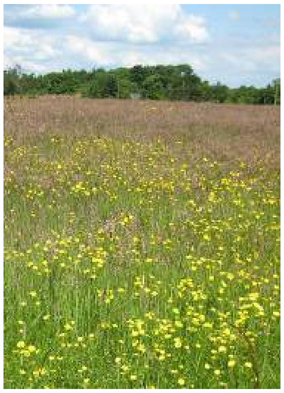

Waist high in buttercups, Pengethley © Pauline E (cc-by-sa/2.0)

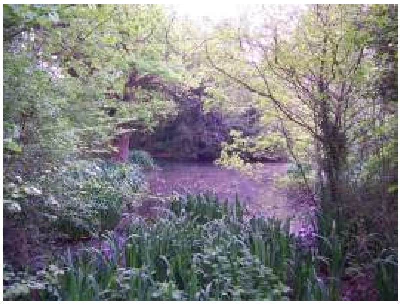

The Pond - Birklands Meadow © anthony helm (cc-by-sa/2.0)

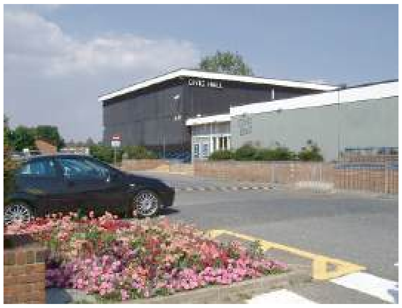

Grays Civic Hall (Blackshots) © Glyn Baker (cc-by-sa/2.0)

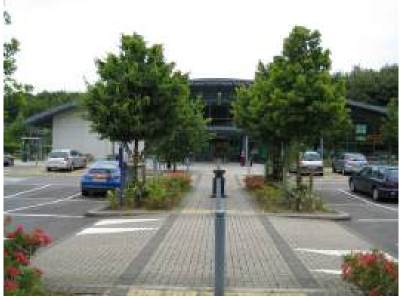

M3 Motorway: Winchester Services (northbound) © Nigel Cox (cc-by-sa/2.0)

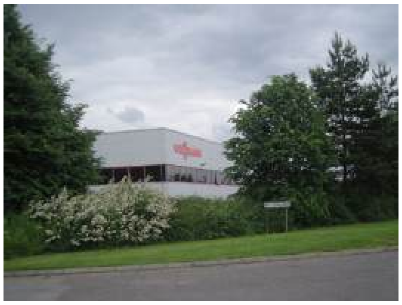

Factory at Hortonwood © Row17 (cc-by-sa/2.0)

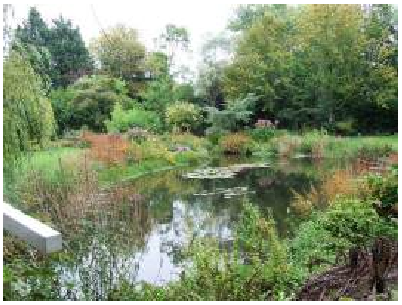

Gooderstone Water Gardens, Gooderstone, Norfolk © David Mills (cc-by-sa/2.0)

3.47 Fluvial Landforms of Streams

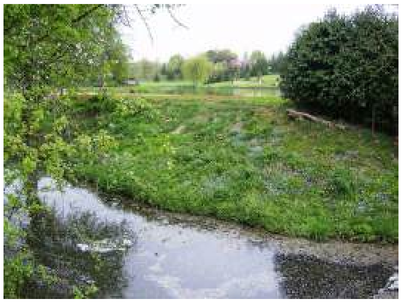

Fish pond at Leigh Lodge © Graham Horn (cc-by-sa/2.0)

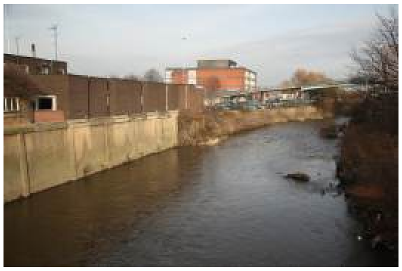

River Don © Richard Croft (cc-by-sa/2.0)

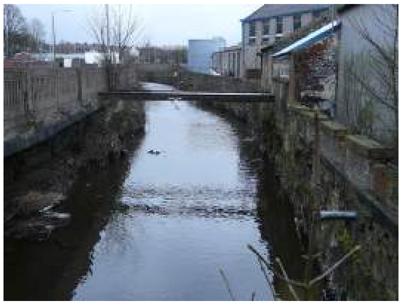

River Darwen © Tony Mercer (cc-by-sa/2.0)

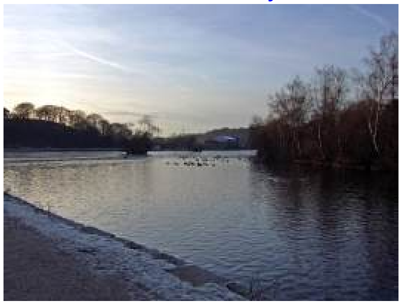

Etherow Country Park © Bob Abell (cc-by-sa/2.0)

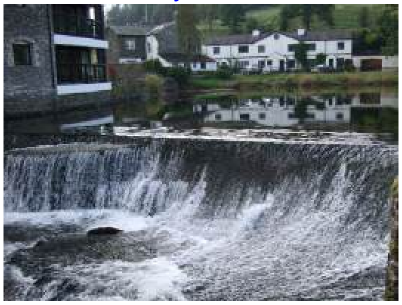

Weir at Cowan Head © Adie Jackson (cc-by-sa/2.0)

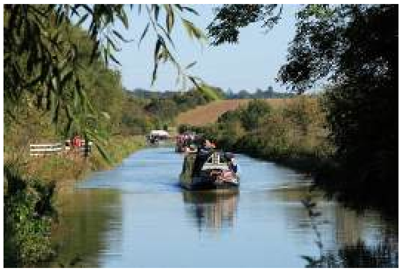

Boats on the Kennet and Avon Canal © Doug Lee (cc-by-sa/2.0)

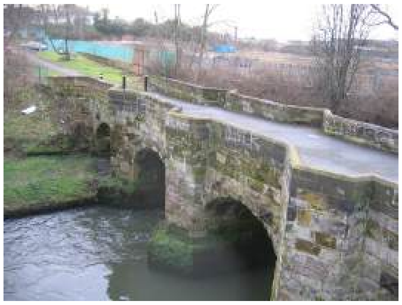

"Zig-Zag" Bridge crossing the River Tame. Perry Barr. © Roy Hughes (cc-by-sa/2.0)

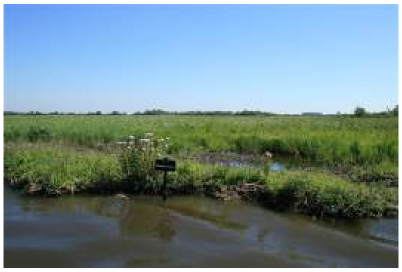

Bank erosion, River Bure © Katy Walters (cc-by-sa/2.0)

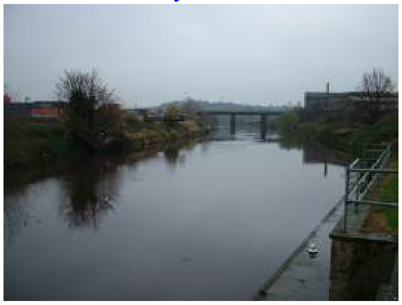

River Calder, Wakefield © John Goldsmith (cc-by-sa/2.0)

3.48 Font

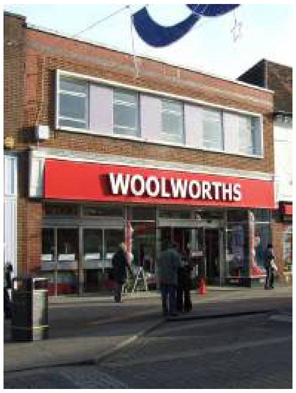

Woolworths Sudbury © Keith Evans (cc-by-sa/2.0)

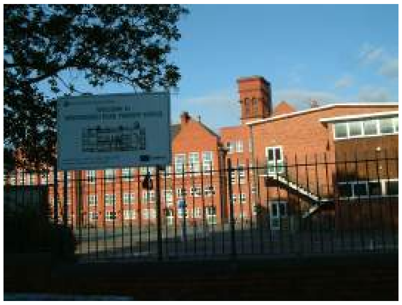

Woodchurch Road Primary School, Birkenhead © Robin Lucas (cc-by-sa/2.0)

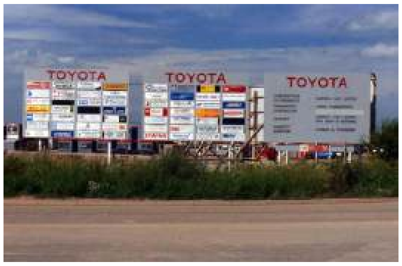

Toyota Car Factory, Burnaston, Derby © mike smith (cc-by-sa/2.0)

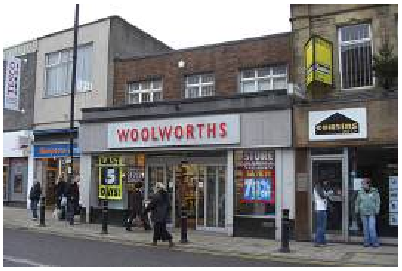

Woolworths, Market Street, Shaw © michael ely (cc-by-sa/2.0)

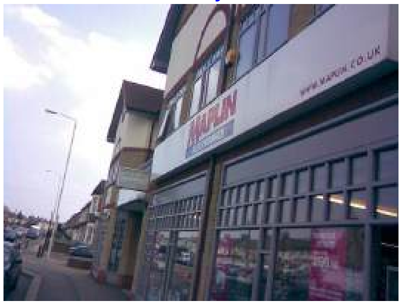

Maplin Store on Green Lane © Robert Lamb (cc-by-sa/2.0)

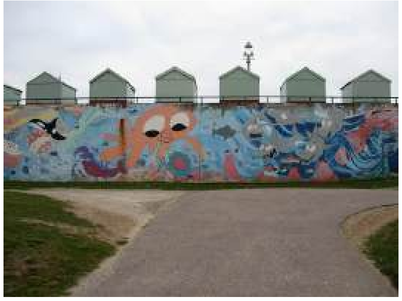

Mural, Hove Lagoon © Simon Carey (cc-by-sa/2.0)

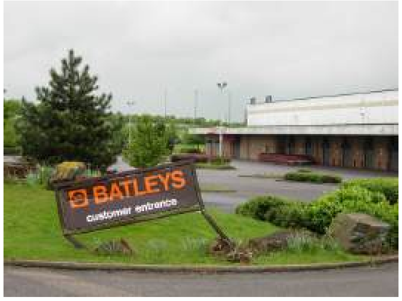

Batleys Cash & Carry, Cross Point Business Park © Stephen McKay (cc-by-sa/2.0)

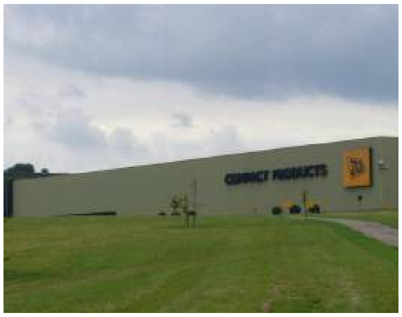

JCB Compact Products © Roger W Haworth (cc-by-sa/2.0)

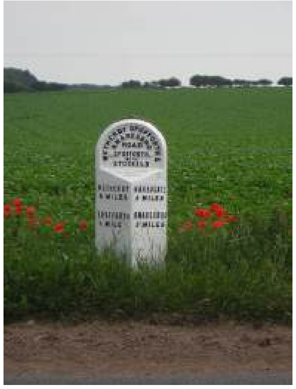

Milestone at Crosper Farm © manonabike (cc-by-sa/2.0)

3.49 Forest

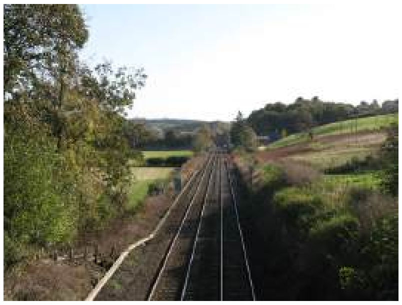

The Newcastle-Carlisle railway line near Dilston (2) © Mike Quinn (cc-by-sa/2.0)

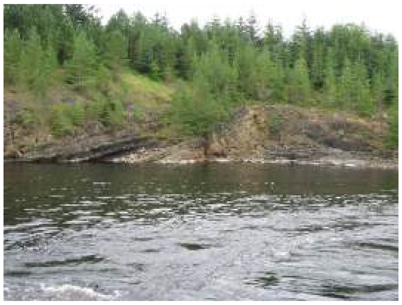

Plashetts Quarry © Pete Saunders (cc-by-sa/2.0)

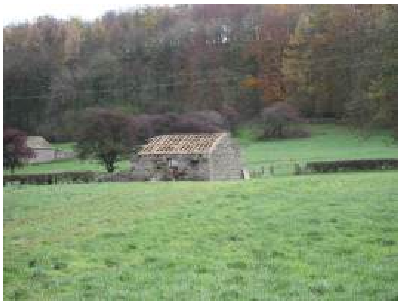

A Roofless Barn © Roger Gilbertson (cc-by-sa/2.0)

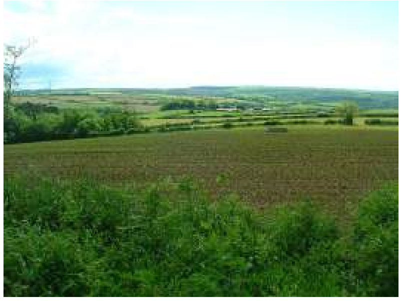

Looking down to Lower Croan © William Bartlett (cc-by-sa/2.0)

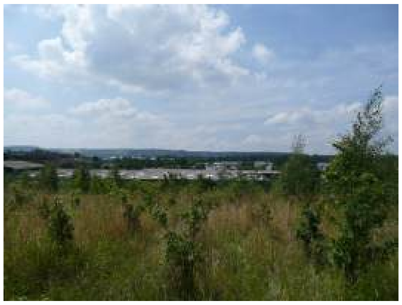

Wentworth Industrial Park © Wendy North (cc-by-sa/2.0)

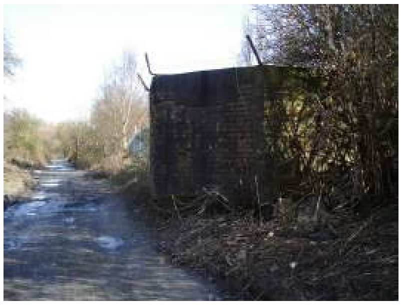

Pillbox watches over the abandoned railway © Jimmy Reeves (cc-by-sa/2.0)

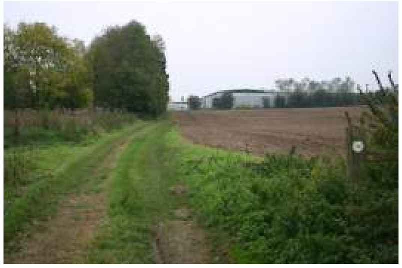

Approaching the Long Crendon Industrial estate © Shaun Ferguson (cc-by-sa/2.0)

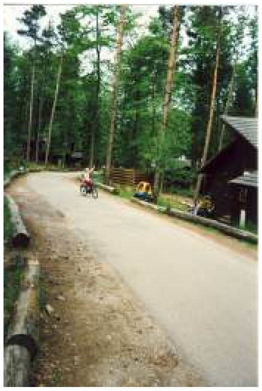

Centerparc - Whinfell Forest © Peter Whatley (cc-by-sa/2.0)

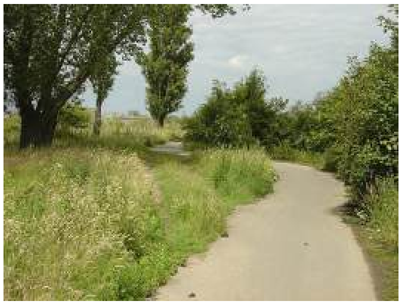

Path to the foreshore © Alan Murray-Rust (cc-by-sa/2.0)

3.50 Freeway

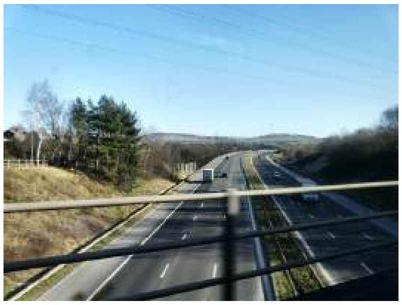

Crossing the M67 © Gerald England (cc-by-sa/2.0)

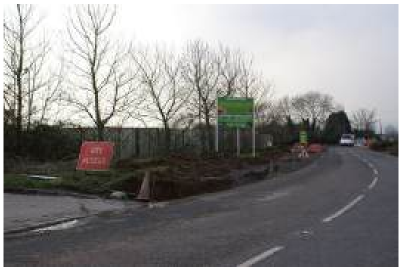

Cheddar Road Works © Adrian and Janet Quantock (cc-by-sa/2.0)

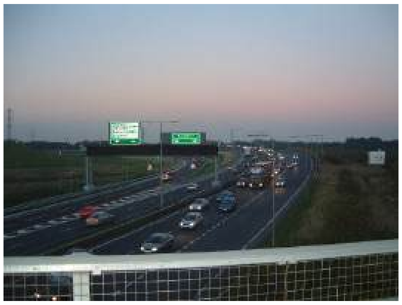

Rush Hour Traffic © Steve McShane (cc-by-sa/2.0)

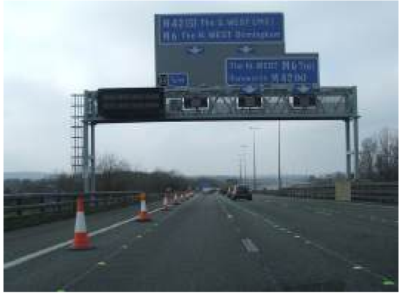

M6 / M6 toll Gantry © Richard cattel (cc-by-sa/2.0)

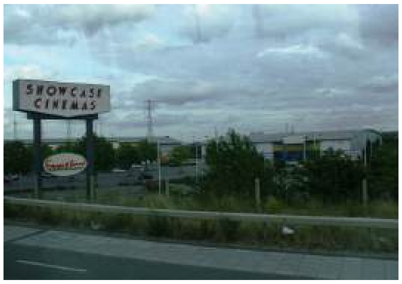

Showcase Cinemas, Barking © Phillip Perry (cc-by-sa/2.0)

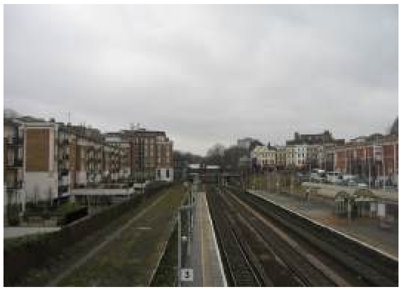

Kensington Olympia station © Mr Ignavy (cc-by-sa/2.0)

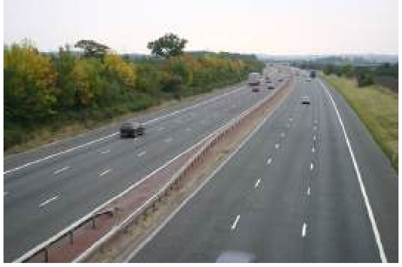

Looking South on M40 © Shaun Ferguson (cc-by-sa/2.0)

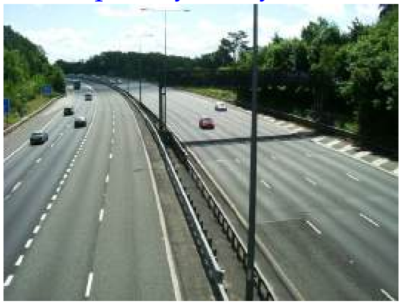

M40 motorway © Phillip Perry (cc-by-sa/2.0)

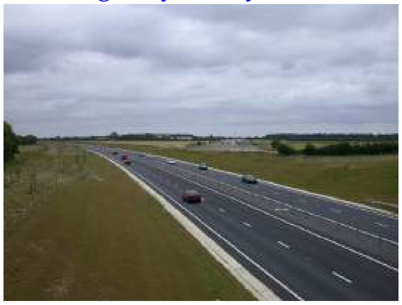

The new A428, and Bourn Airfield runway © Keith Edkins (cc-by-sa/2.0)

3.51 Garden

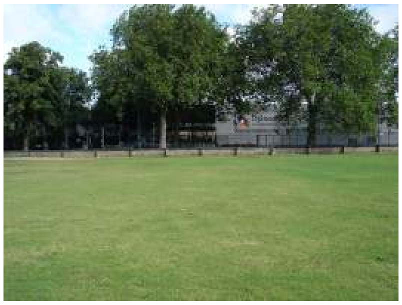

Djanogly City Academy - Forest Campus © Oxymoron (cc-by-sa/2.0)

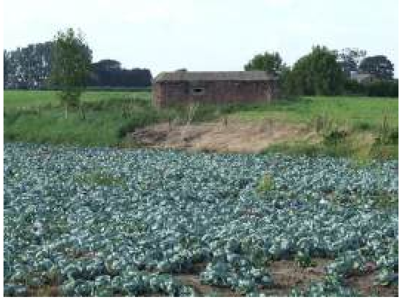

Pill Box © Tony Bennett (cc-by-sa/2.0)

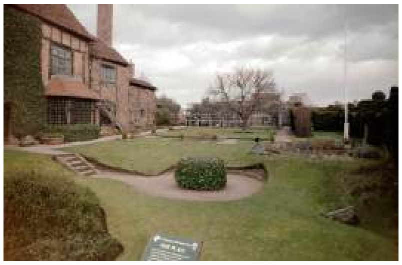

New Place gardens © Keith Edkins (cc-by-sa/2.0)

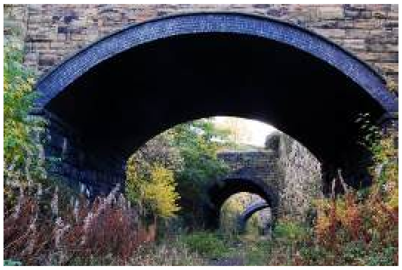

Heckmondwicke cutting-The Leeds new line © philld (cc-by-sa/2.0)

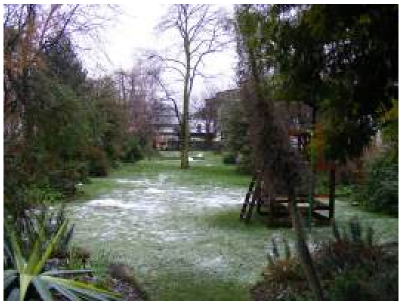

Gardens in Markham Square © PAUL FARMER (cc-by-sa/2.0)

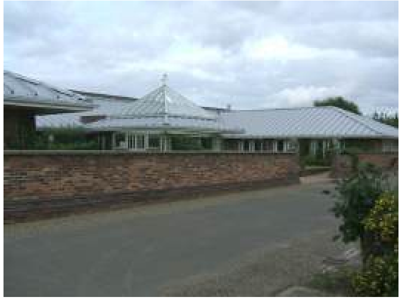

Dunston Hill Hospital © Bill Henderson (cc-by-sa/2.0)

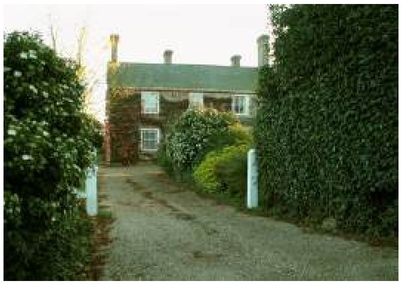

Farmhouse at Spring Farm © Robert Edwards (cc-by-sa/2.0)

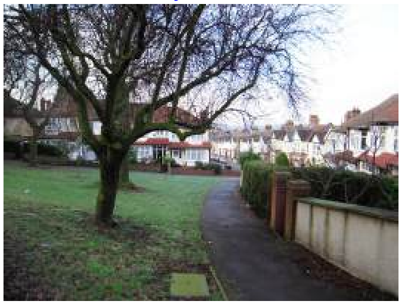

Annsworth Crescent (off Grange Road) © Chris L L (cc-by-sa/2.0)

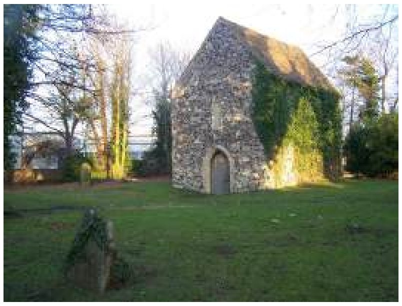

Remains of Church © David Anstiss (cc-by-sa/2.0)

3.52 Gas

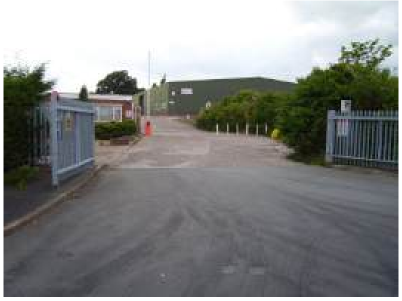

Muller England © Richard Webb (cc-by-sa/2.0)

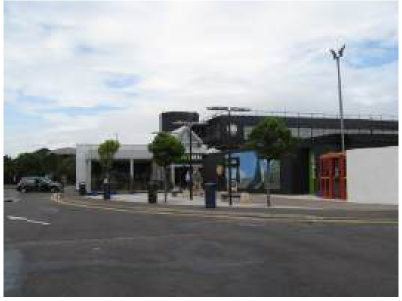

Keele Services - northbound side © NA (cc-by-sa/2.0)

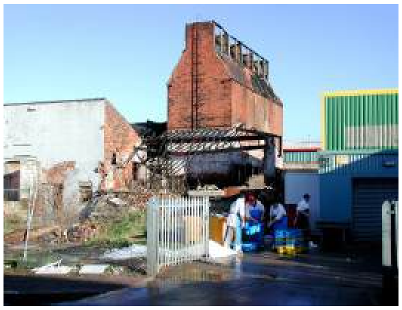

Subway Street, Hull © Paul Glazzard (cc-by-sa/2.0)

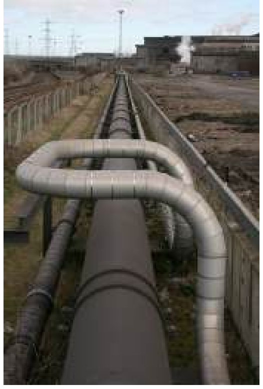

Pipelines © Mick Garratt (cc-by-sa/2.0)

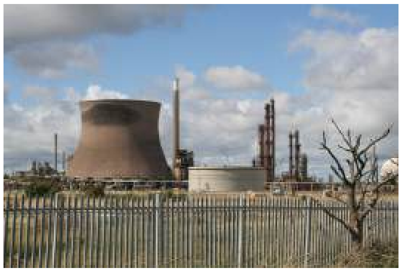

Wilton International © Alan Murray-Rust (cc-by-sa/2.0)

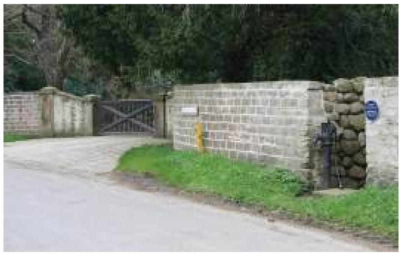

Village Pump © David Rogers (cc-by-sa/2.0)

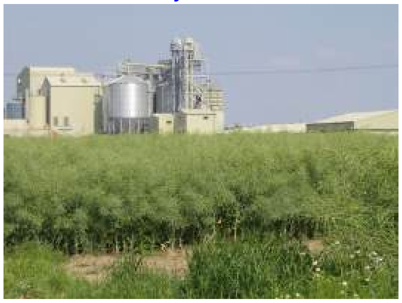

Green Farm © Graham Horn (cc-by-sa/2.0)

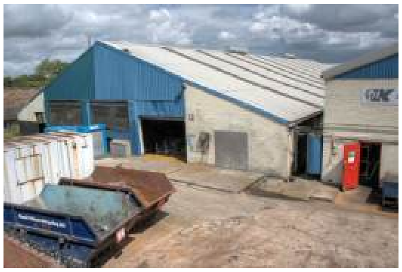

Lionweld Kennedy Works © Mick Garratt (cc-by-sa/2.0)

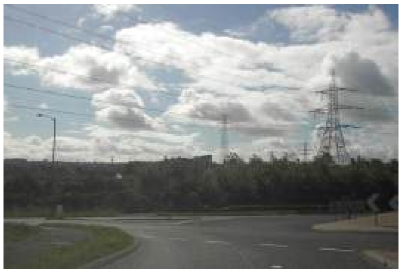

Stargate Roundabout © Darren Haddock (cc-by-sa/2.0)

3.53 Girder Bridge

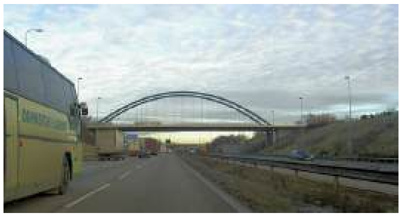

Steel arch bridge over the M42 © Steve Fareham (cc-by-sa/2.0)

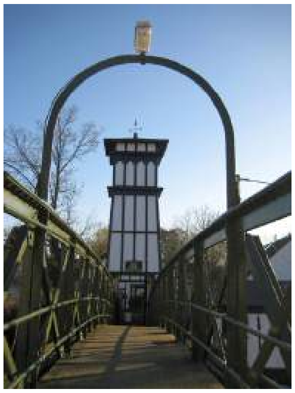

Sunbury Court Island: Footbridge and Tower © Nigel Cox (cc-by-sa/2.0)

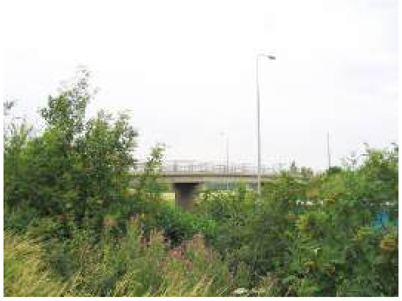

M6 Bridge © Dave Smethurst (cc-by-sa/2.0)

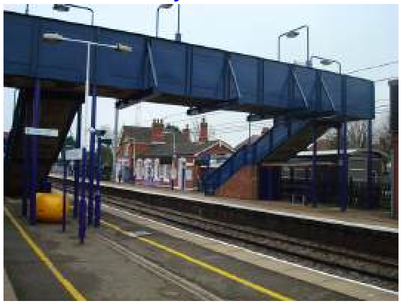

Leagrave Station © Stacey Harris (cc-by-sa/2.0)

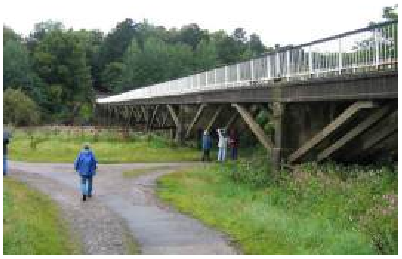

Old Tramroad Bridge, Preston © A-M-Jervis (cc-by-sa/2.0)

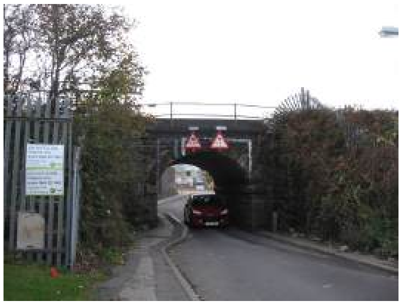

Low Bridge, Heysham Road © Sue Adair (cc-by-sa/2.0)

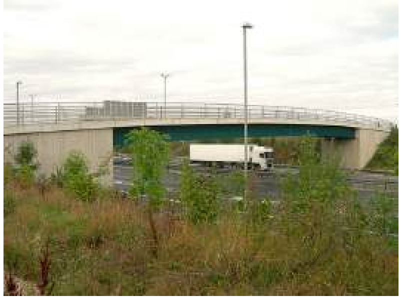

Footbridge over M11 south of M11/A120 interchange © John Smith (cc-by-sa/2.0)

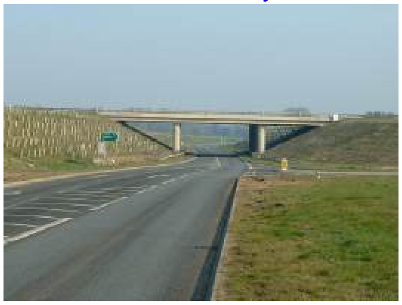

A11 crossing B1111 © Keith Evans (cc-by-sa/2.0)

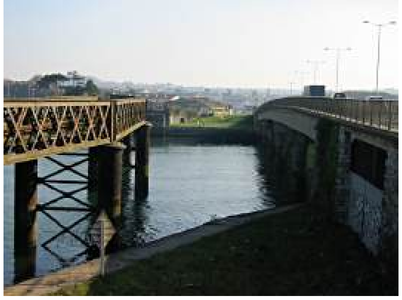

Two Bridges Cross The Laira © Tony Atkin (cc-by-sa/2.0)

3.54 Grass

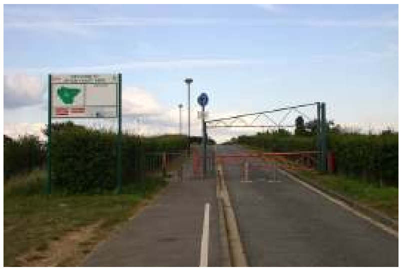

Entrance to Upton Court park © Shaun Ferguson (cc-by-sa/2.0)

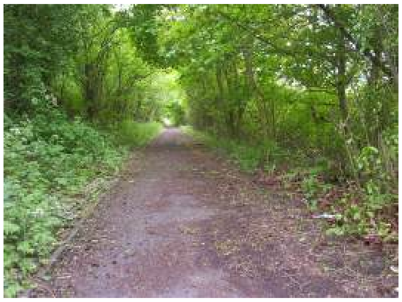

Footpath © Weston Beggard (cc-by-sa/2.0)

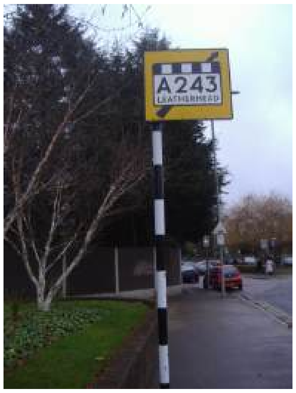

Pre-Worboys sign Tolworth © David Howard (cc-by-sa/2.0)

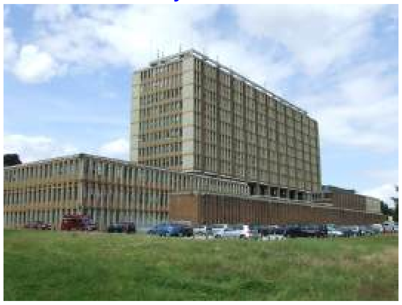

County Hall © Keith Evans (cc-by-sa/2.0)

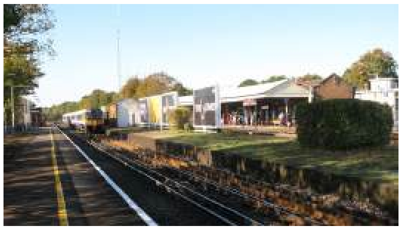

Walton-on-Thames station (3) © Mike Quinn (cc-by-sa/2.0)

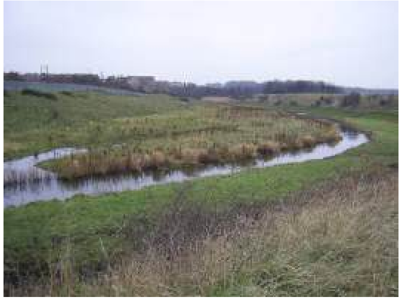

Lyneburn © george hurrell (cc-by-sa/2.0)

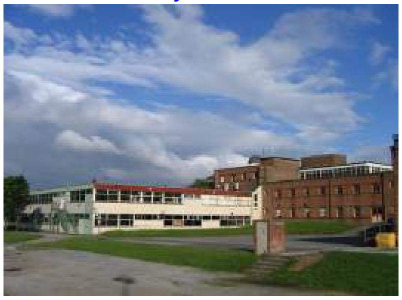

Norton College, Sheffield © Graham Hardy (cc-by-sa/2.0)

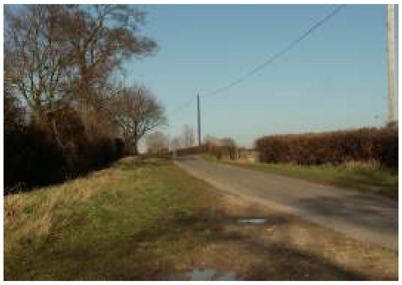

Part of Old Church Road © Robert Edwards (cc-by-sa/2.0)

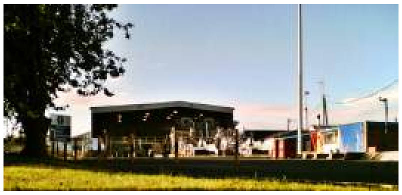

Frozen Food Factory at Autby near North Thoresby © Bob Emm (cc-by-sa/2.0)

3.55 Grass Family

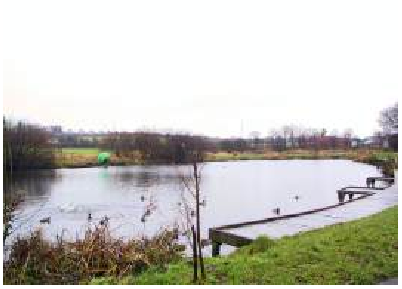

Radcliffe Anglers © Roger May (cc-by-sa/2.0)

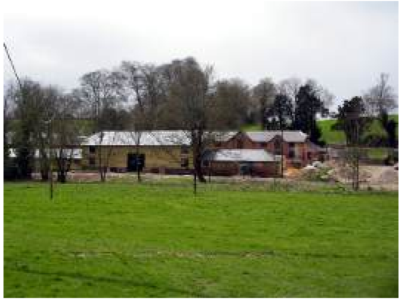

Nine Mile Water Farm © Peter Jordan (cc-by-sa/2.0)

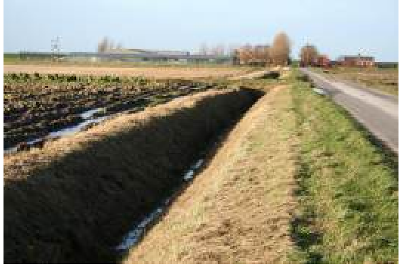

Marsh Farm © Roger Whittleston (cc-by-sa/2.0)

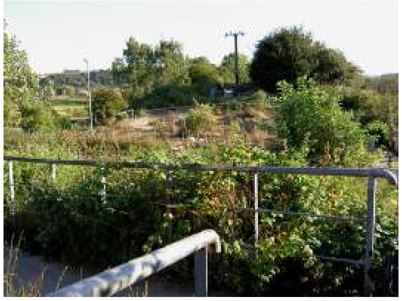

Zig zag access onto Trans Pennine Trail. © Steve Fareham (cc-by-sa/2.0)

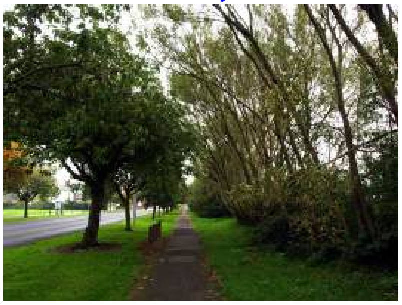

Sutton Road's Southern Footpath © Andy Beecroft (cc-by-sa/2.0)

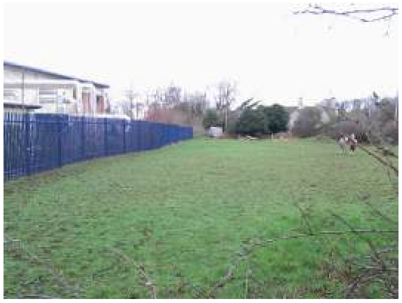

Boundary fence © David Luther Thomas (cc-by-sa/2.0)

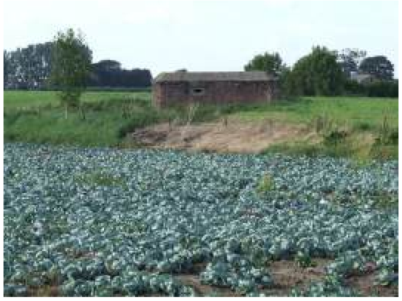

Pill Box © Tony Bennett (cc-by-sa/2.0)

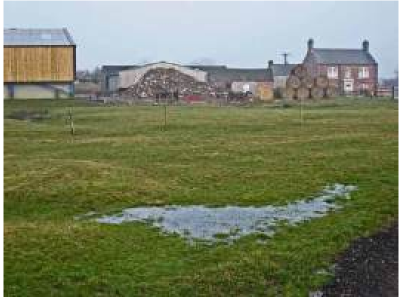

Low Swainston Farm © Oliver Dixon (cc-by-sa/2.0)

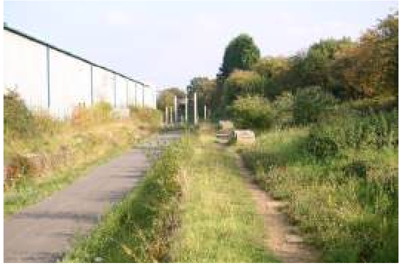

Site of the old Thame railway Station © Shaun Ferguson (cc-by-sa/2.0)

3.56 Grassland

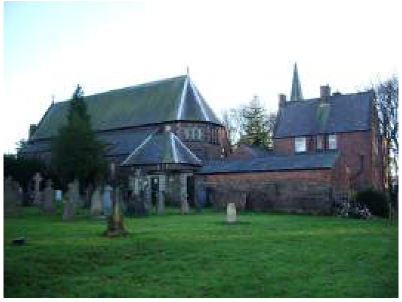

St Mary's Catholic Church, Euxton © Alexander P Kapp (cc-by-sa/2.0)

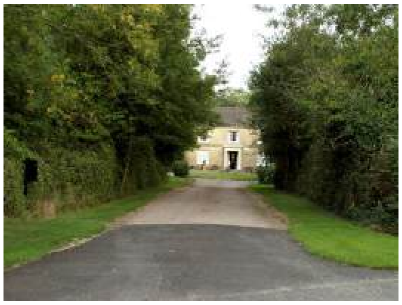

House at High Street Green, Suffolk © Robert Edwards (cc-by-sa/2.0)

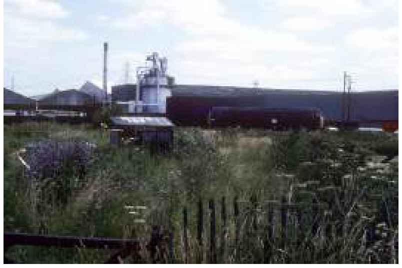

Freight train at Rye House © roger geach (cc-by-sa/2.0)

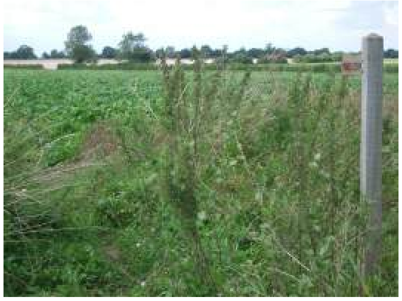

Footpath to Wood Green © Andrew Longton (cc-by-sa/2.0)

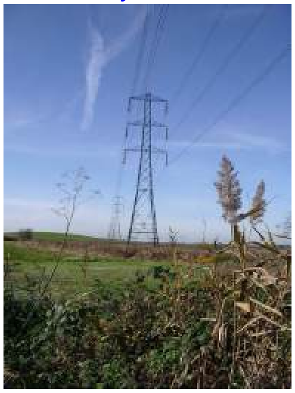

Pylons off Seasalter Lane © pam fray (cc-by-sa/2.0)

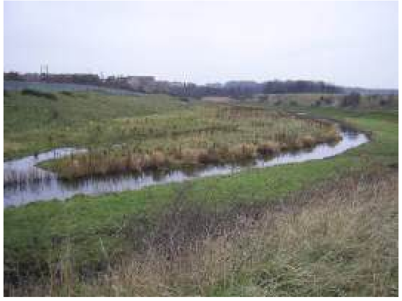

Lyneburn © george hurrell (cc-by-sa/2.0)

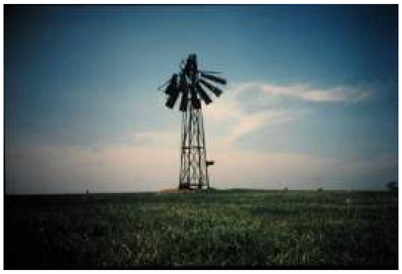

Sevington windpump © Michael Roots (cc-by-sa/2.0)

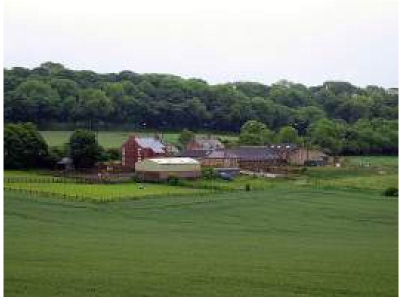

Over the Hill Farm © Roger Smith (cc-by-sa/2.0)

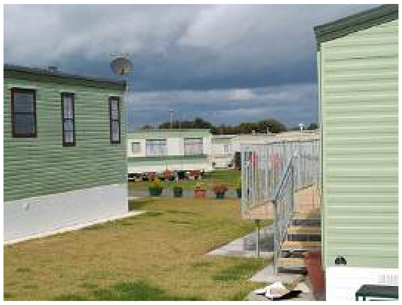

Hazel Grove Holiday Park © Stephen McCulloch (cc-by-sa/2.0)

3.57 Groundcover

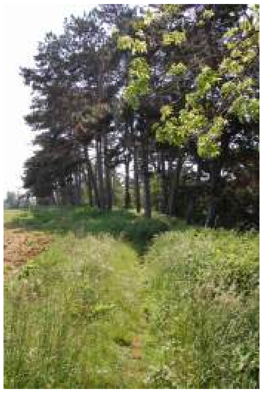

Part of Lodge Plantation and footpath © Roger Miller (cc-by-sa/2.0)

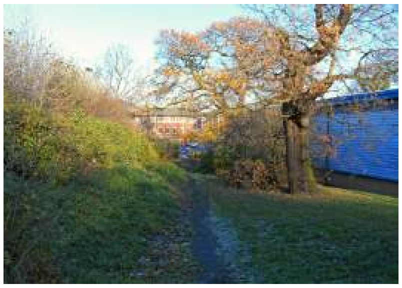

Public footpath on the Meridian Business Park © Mat Fascione (cc-by-sa/2.0)

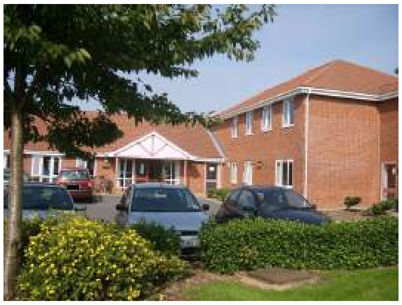

Altham Meadows © Michael Graham (cc-by-sa/2.0)

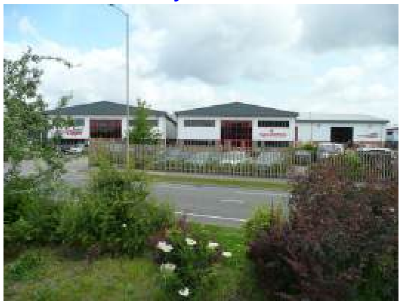

Business units © Jonathan Billinger (cc-by-sa/2.0)

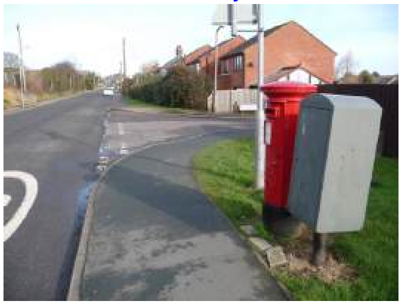

Lytchett Matravers: postbox BH16 258, Wareham Road © Chris Downer (cc-by-sa/2.0)

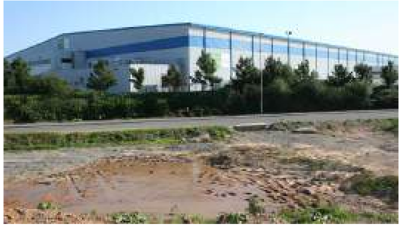

Waitrose Regional Distribution Centre © Les Carruthers (cc-by-sa/2.0)

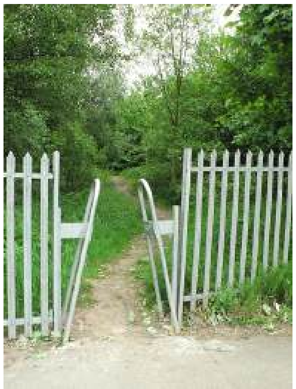

Squeeze stile by Ring Road, West Park © Rich Tea (cc-by-sa/2.0)

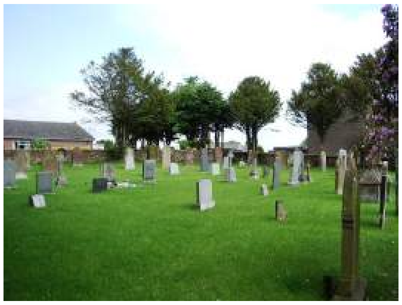

Graveyard, Outgang Road, Aspatria © Alexander P Kapp (cc-by-sa/2.0)

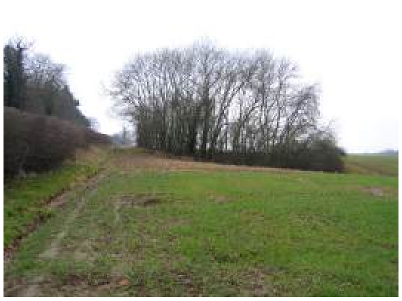

Wooded former marlpit, Raynham, Norfolk © Rodney Burton (cc-by-sa/2.0)

3.58 Headquarters

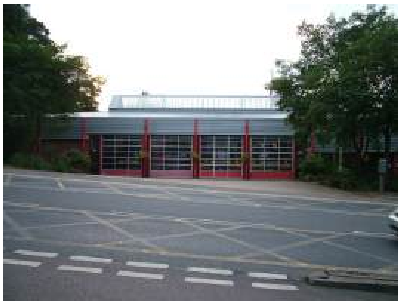

Fire Station, Bury St. Edmunds © John Goldsmith (cc-by-sa/2.0)

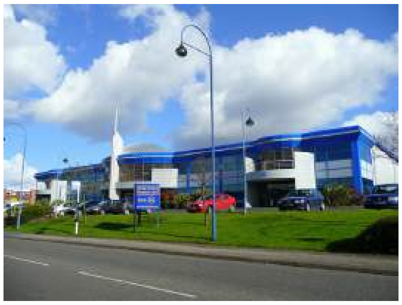

A Really Useful business, Pensnett Trading Estate © Jonathan Billinger (cc-by-sa/2.0)

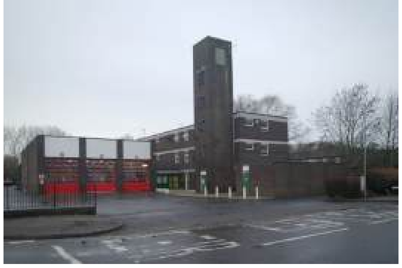

Binley fire station © Kevin Hale (cc-by-sa/2.0)

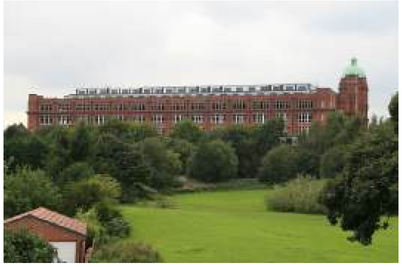

Astley Bridge Mill © Chris Allen (cc-by-sa/2.0)

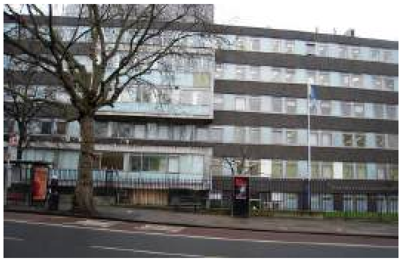

King's College Hospital Dental © Chris L L (cc-by-sa/2.0)

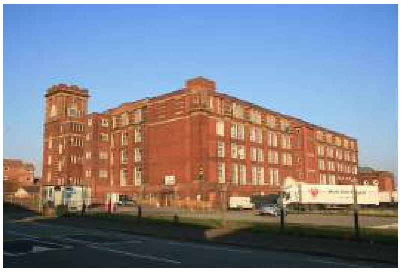

Pilot Mill, Bury © Chris Allen (cc-by-sa/2.0)

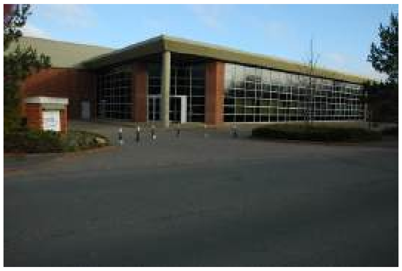

Business unit, Enigma Business Park © Philip Halling (cc-by-sa/2.0)

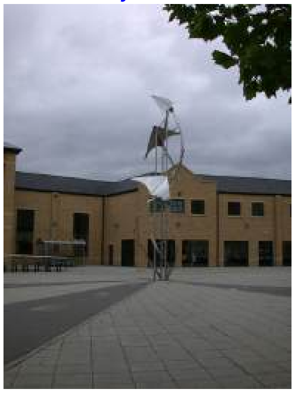

Flight © Keith Edkins (cc-by-sa/2.0)

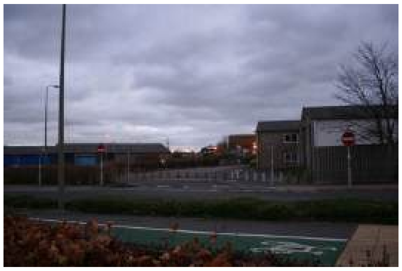

Rich's sidings © Bill Nicholls (cc-by-sa/2.0)

3.59 Highland

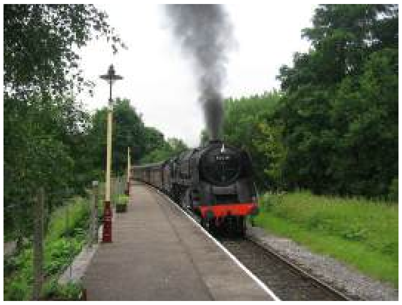

Summerseat Railway Station © Paul Anderson (cc-by-sa/2.0)

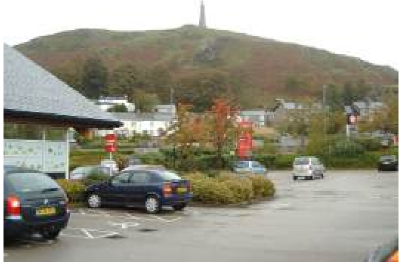

Booths Car Park © Darrin Antrobus (cc-by-sa/2.0)

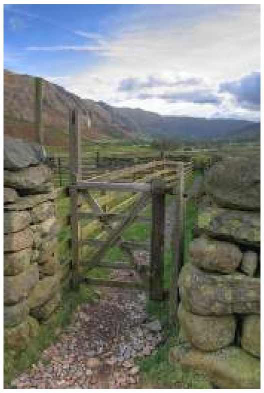

Sheepfold, Oxendale © Mick Garratt (cc-by-sa/2.0)

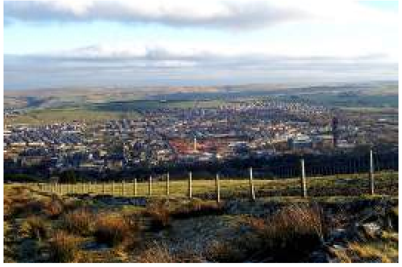

Darwen © Allister Combe (cc-by-sa/2.0)

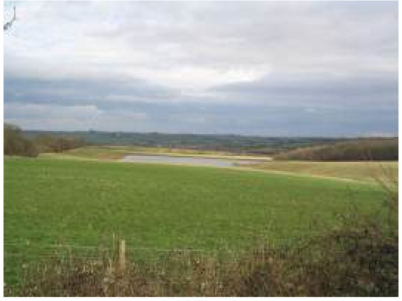

New lake © Tim Heaton (cc-by-sa/2.0)

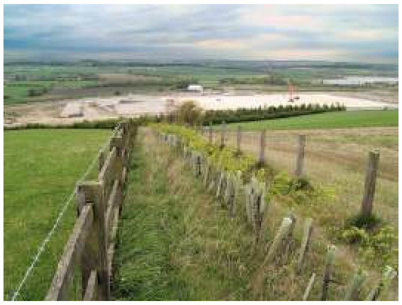

Site preparation for new warehouse. © Steve Fareham (cc-by-sa/2.0)

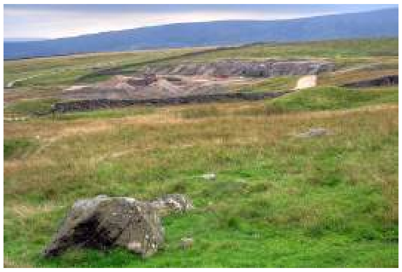

General View of the Beever Mine Workings © Mick Garratt (cc-by-sa/2.0)

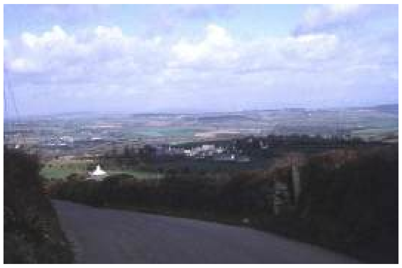

Lane to Upton Cross © Trevor Rickard (cc-by-sa/2.0)

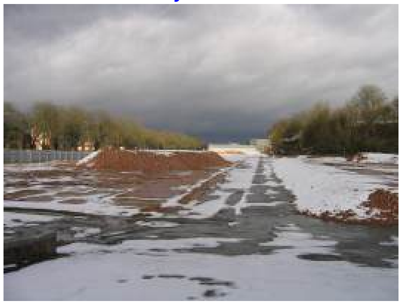

Factory razed to the ground - Longbridge © Roy Hughes (cc-by-sa/2.0)

3.60 Hill

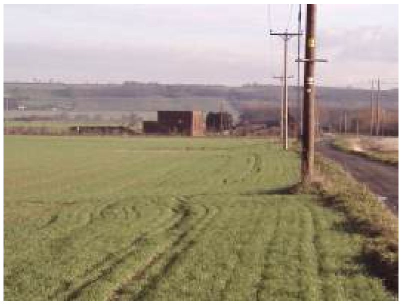

Scotney Farm Lane © fred roberts (cc-by-sa/2.0)

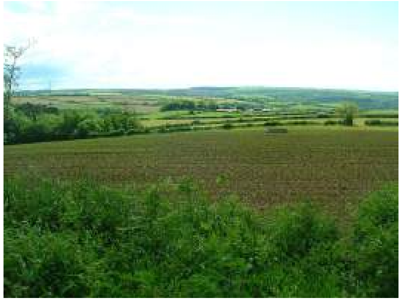

Looking down to Lower Croan © William Bartlett (cc-by-sa/2.0)

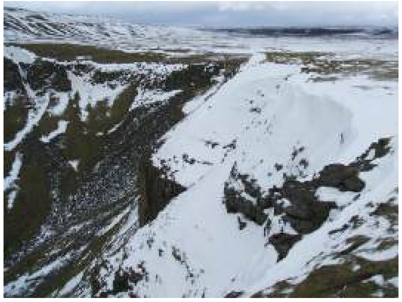

High Cup Nick © David Brown (cc-by-sa/2.0)

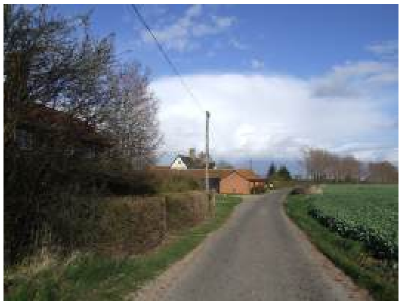

Clintergate © Ian Robertson (cc-by-sa/2.0)

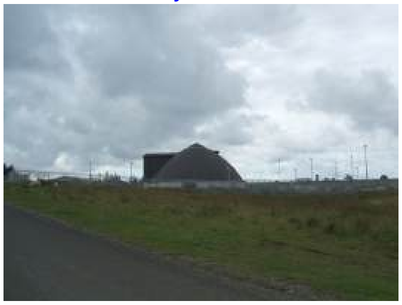

Highways Agency building, M6 © David Brown (cc-by-sa/2.0)

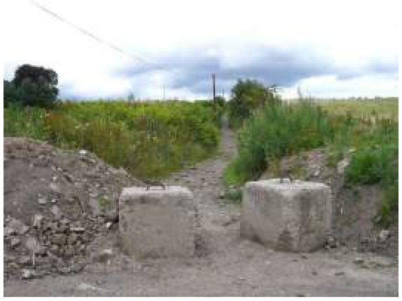

Track past Latebrook House © Steve Lewin (cc-by-sa/2.0)

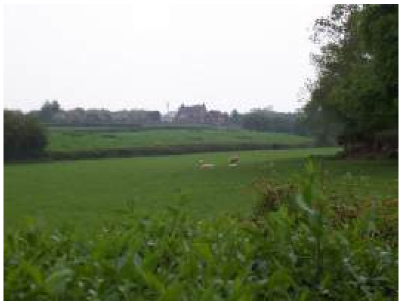

Moss Hill Farm © Rob Hinkley (cc-by-sa/2.0)

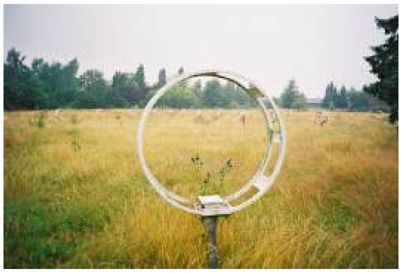

The Aerial Field, Stirling Lines © Paul Taunton (cc-by-sa/2.0)

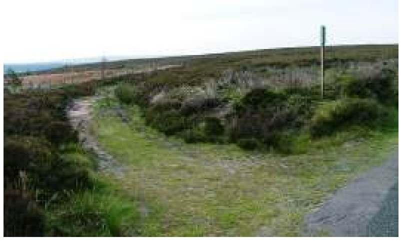

Public Bridleway to High Gill Beck © Mick Garratt (cc-by-sa/2.0)

3.61 Home

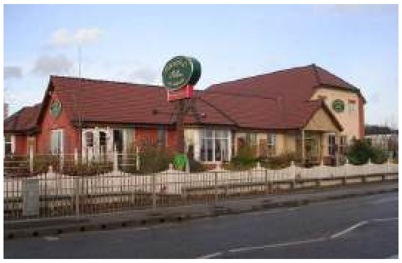

Winding Wheel - Junction 32 Retail Outlet © Betty Longbottom (cc-by-sa/2.0)

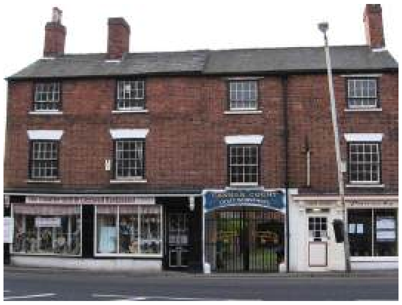

Chesterfield - Cannon Court © Dave Bevis (cc-by-sa/2.0)

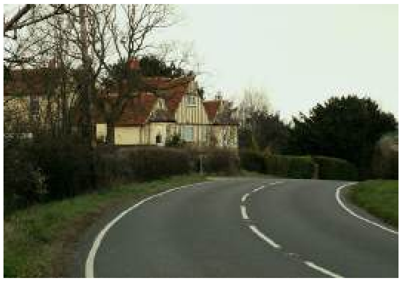

The farmhouse at Brownings Farm © Robert Edwards (cc-by-sa/2.0)

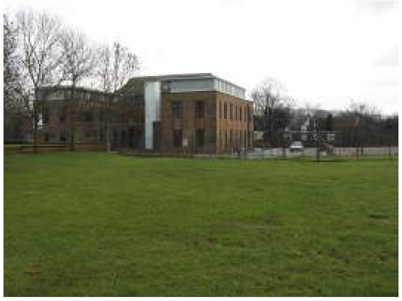

Awaiting An Owner © Peter Whatley (cc-by-sa/2.0)

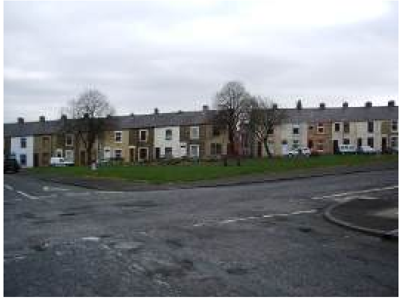

Open space © Alexander P Kapp (cc-by-sa/2.0)

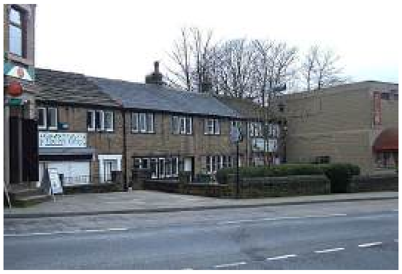

Holmfirth Road, Meltham © michael ely (cc-by-sa/2.0)

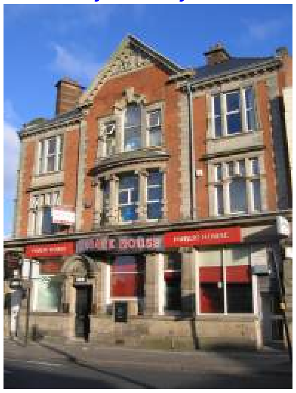

Midland Bank 382 Stratford Road. Sorting code 40-11-32 © Roy Hughes (cc-by-sa/2.0)

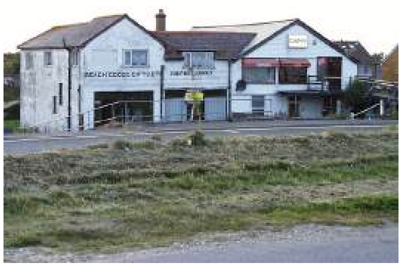

Derelict Shops © Stuart Vickers (cc-by-sa/2.0)

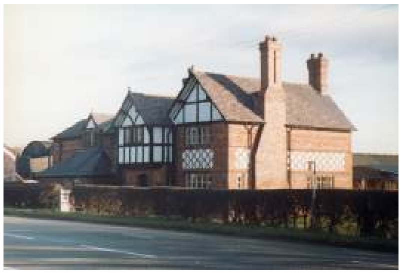

Manor Farm, Tabley © Paul Ravenscroft (cc-by-sa/2.0)

3.62 Horizon

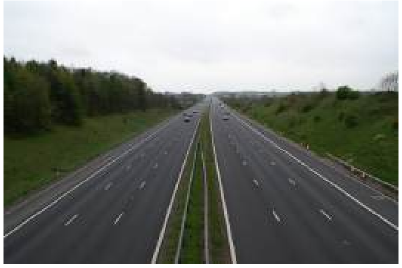

M5 to Exeter © Adrian and Janet Quantock (cc-by-sa/2.0)

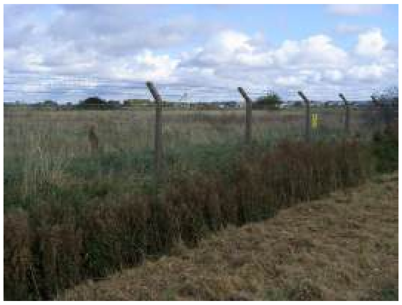

Chalgrove airfield © Shaun Ferguson (cc-by-sa/2.0)

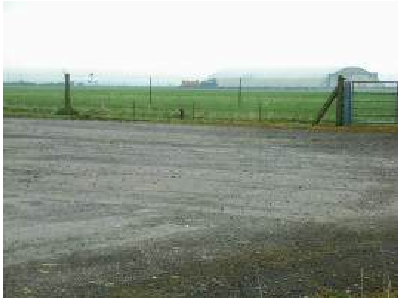

Hangars at Little Rissington Airfield © David Luther Thomas (cc-by-sa/2.0)

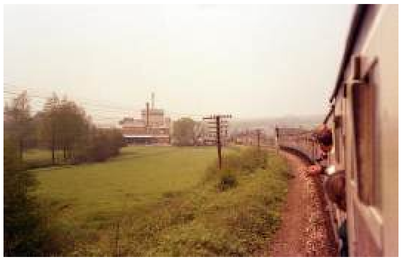

Torbay Express © John Lucas (cc-by-sa/2.0)

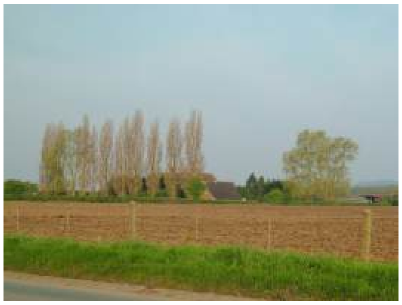

Old Farm guest house © Andy and Hilary (cc-by-sa/2.0)

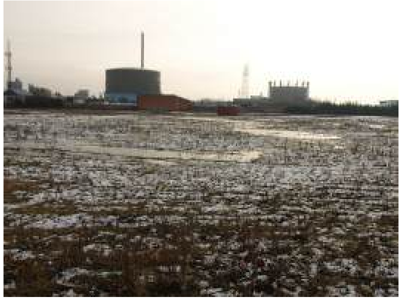

Snow-covered Industrial Wasteland © Andy Beecroft (cc-by-sa/2.0)

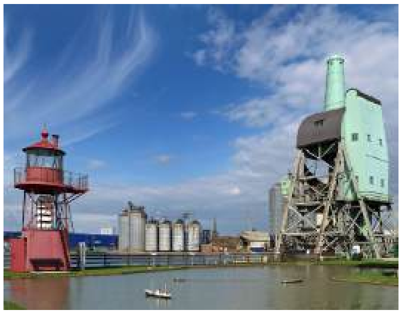

Goole Model Boat Club pond © Steve Fareham (cc-by-sa/2.0)

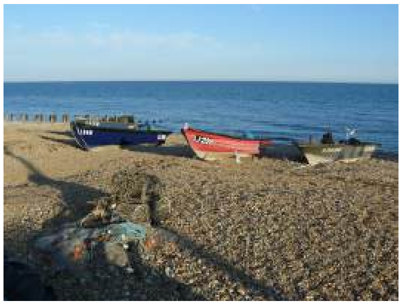

Bognor Beach © Martin Horsfall (cc-by-sa/2.0)

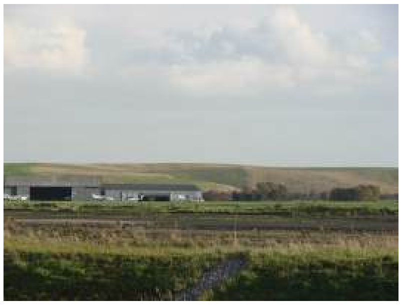

View across the Proving Ground, Sherburn in Elmet Air Field. © Bill Henderson (cc-by-sa/2.0)

3.63 House

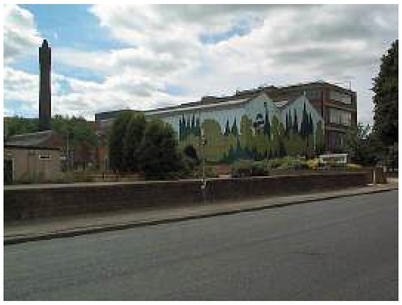

Pool paper mill © David Spencer (cc-by-sa/2.0)

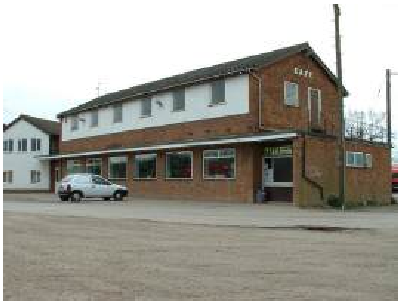

Kates Cafe © Keith Evans (cc-by-sa/2.0)

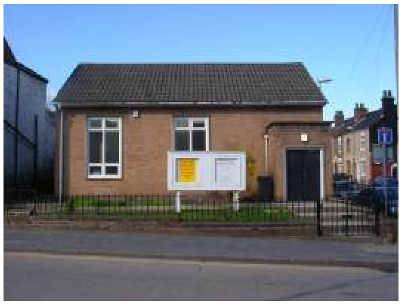

Blurton Free Baptist Church © Andrew (cc-by-sa/2.0)

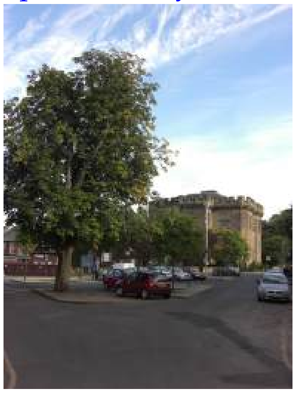

Morpeth Court © Anthony Foster (cc-by-sa/2.0)

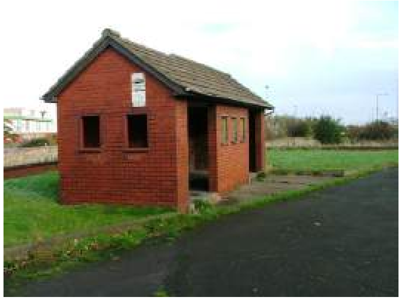

Bus Shelter Outside of Corus's Lackenby Works © Mick Garratt (cc-by-sa/2.0)

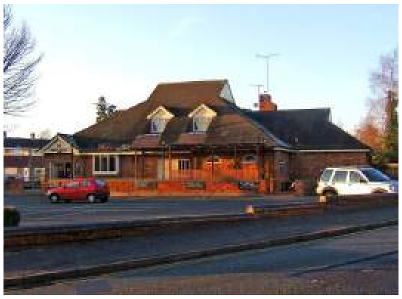

Jacksons, Marlpool Lane, Kidderminster © P L Chadwick (cc-by-sa/2.0)

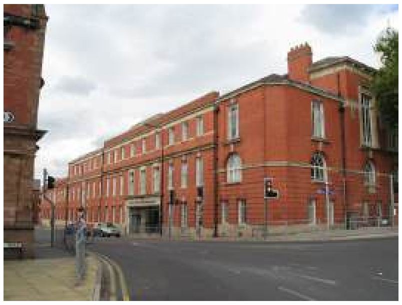

Sale Town Hall - side aspect © NA (cc-by-sa/2.0)

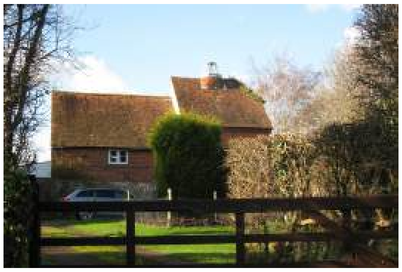

Oast House at Stonestile House, Stonestile Road, Headcorn, Kent © Oast House Archive (cc-by-sa/2.0)

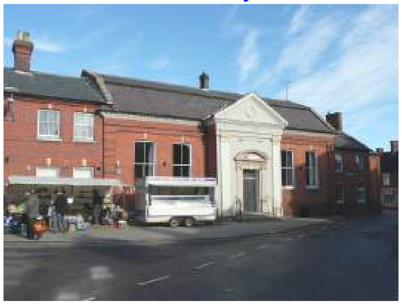

Town Hall and market stalls, Aylsham © Humphrey Bolton (cc-by-sa/2.0)

3.64 Human Settlement

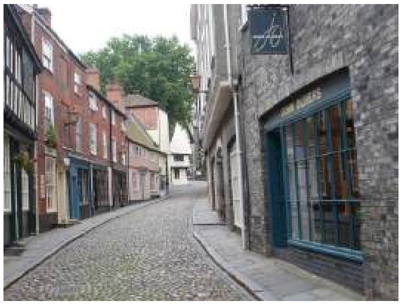

Elm Hill, Norwich © pam fray (cc-by-sa/2.0)

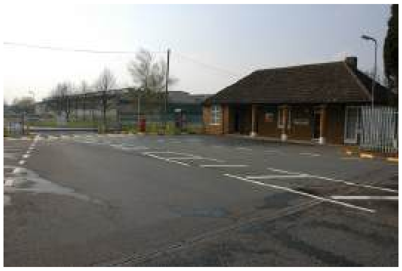

Rushock Trading Estate © Philip Halling (cc-by-sa/2.0)

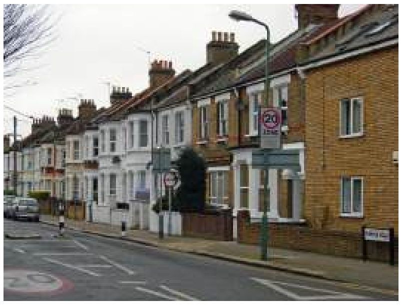

Purves Road, Kensal Green © Stephen McKay (cc-by-sa/2.0)

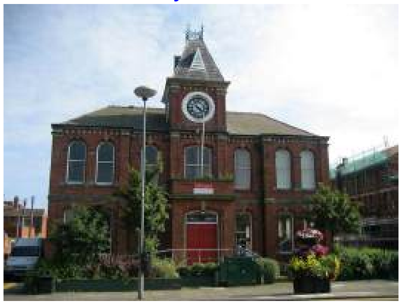

Public Library in Blyth © Chris Heaton (cc-by-sa/2.0)

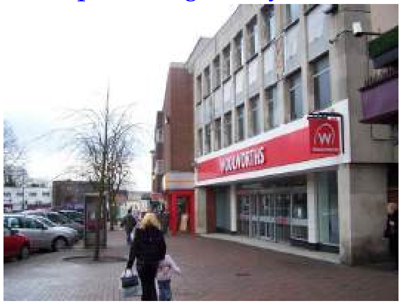

Woolworths, Cannock © Geoff Pick (cc-by-sa/2.0)

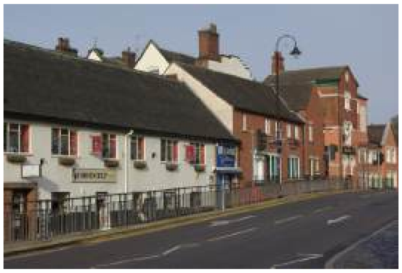

Stafford Street, Stone © Stephen McKay (cc-by-sa/2.0)

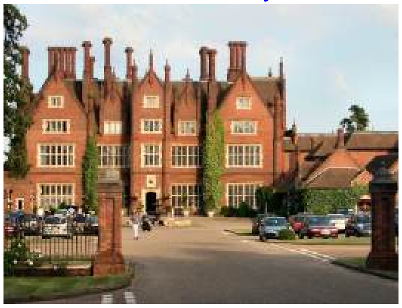

Dunston Hall Hotel © Lis Burke (cc-by-sa/2.0)

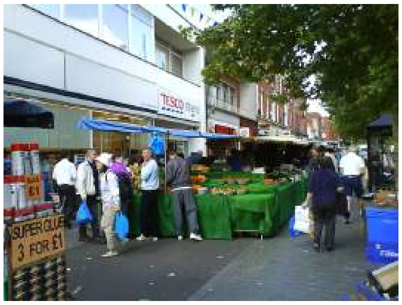

Market Day © Gary Fellows (cc-by-sa/2.0)

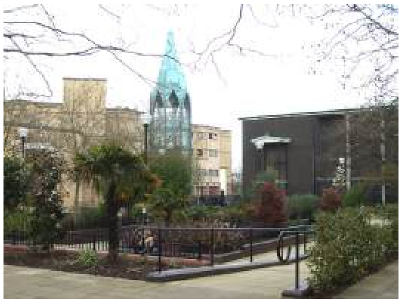

Church Garden © terry joyce (cc-by-sa/2.0)

3.65 Industry

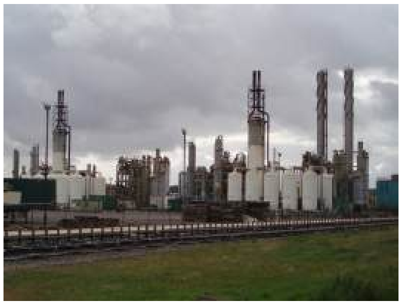

Severnside fertilizer works © Sharon Loxton (cc-by-sa/2.0)

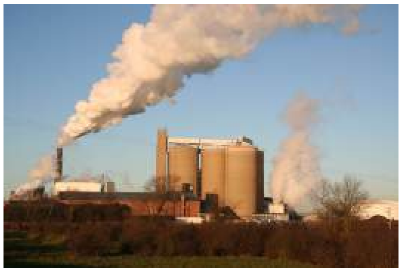

Newark Sugar Factory © Richard Croft (cc-by-sa/2.0)

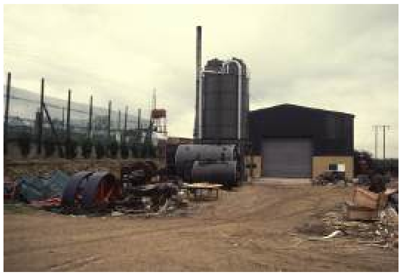

Markham Grange power house © Chris Allen (cc-by-sa/2.0)

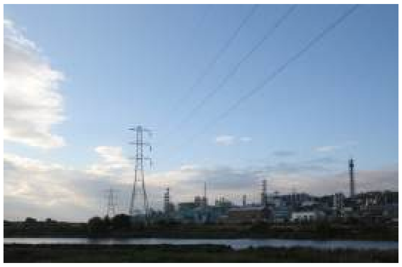

Evening at Weston Point © Alan Murray-Rust (cc-by-sa/2.0)

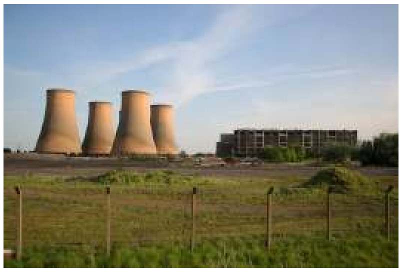

High Marnham partially demolished © Richard Croft (cc-by-sa/2.0)

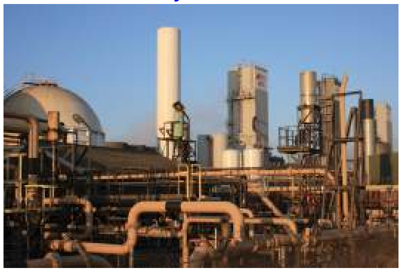

B.O.C Works © Mick Garratt (cc-by-sa/2.0)

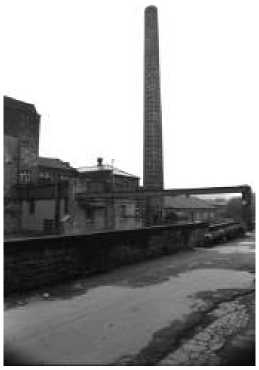

Chimney, Milnsbridge © Chris Allen (cc-by-sa/2.0)

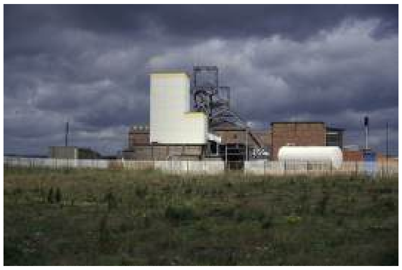

Ellington Colliery © Chris Allen (cc-by-sa/2.0)

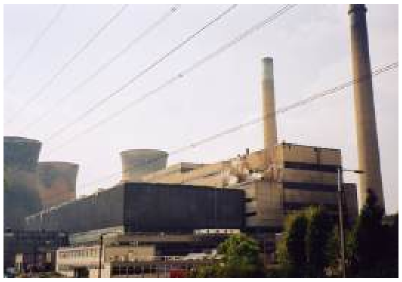

Drakelow 'C' Power station © Chris Bell (cc-by-sa/2.0)

3.66 Infrastructure

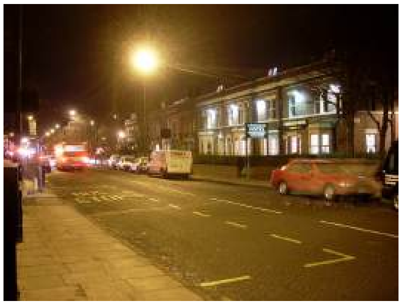

Grainger Hotel, Newcastle © Stephen Sweeney (cc-by-sa/2.0)

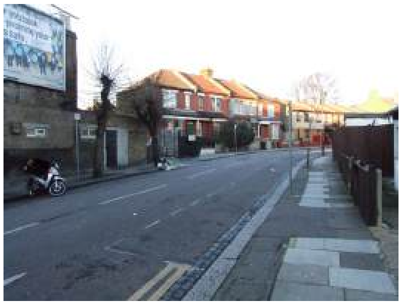

Vincent Road, West Green © Chris Whippet (cc-by-sa/2.0)

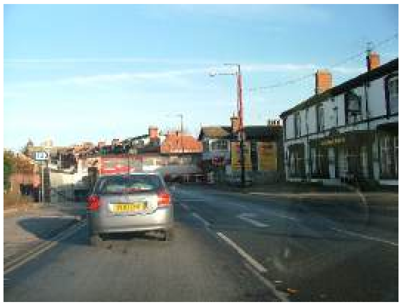

Starbeck level crossing © Robin Hall (cc-by-sa/2.0)

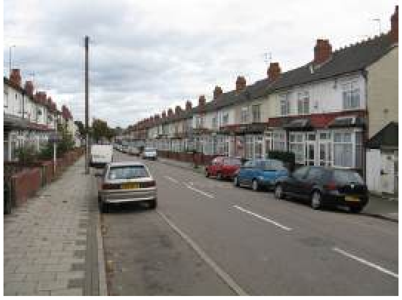

Mary Road © Peter Whatley (cc-by-sa/2.0)

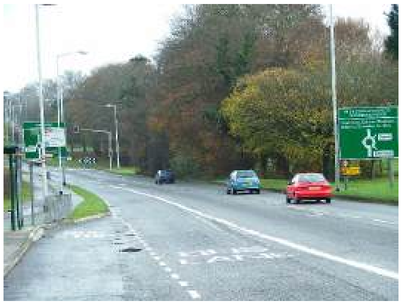

Approach to Derriford Roundabout © Gwyn Jones (cc-by-sa/2.0)

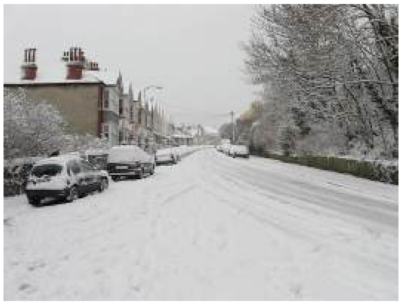

Millers Road © Simon Carey (cc-by-sa/2.0)

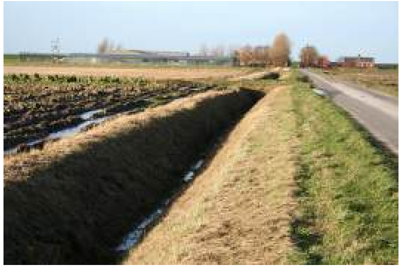

Marsh Farm © Roger Whittleston (cc-by-sa/2.0)

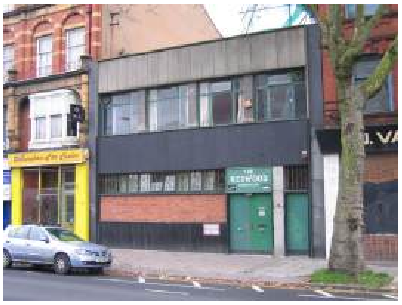

Midland Bank Bristol Street. Sorting code 40-11-39 © Roy Hughes (cc-by-sa/2.0)

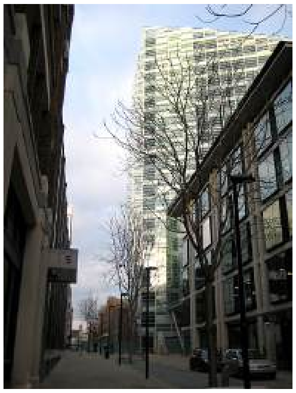

New office block on Wilson Street (2) © Zorba the Geek (cc-by-sa/2.0)

3.67 Lacustrine Plain

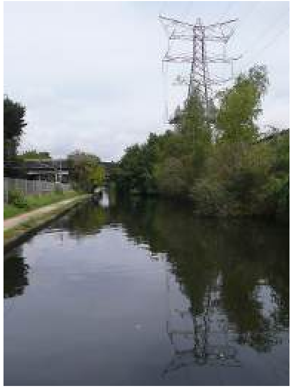

Worcester and Birmingham Canal south of Bournville © Roger D Kidd (cc-by-sa/2.0)

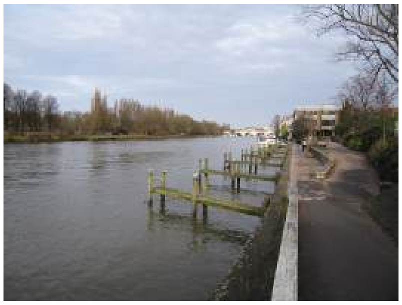

River Walk - Kingston © Bob Parkes (cc-by-sa/2.0)

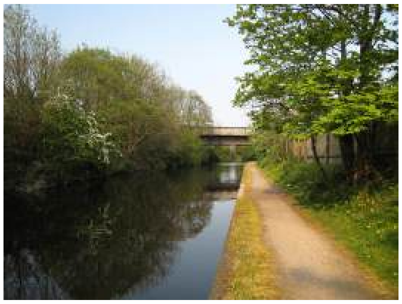

Birmingham & Fazeley Canal © David Stowell (cc-by-sa/2.0)

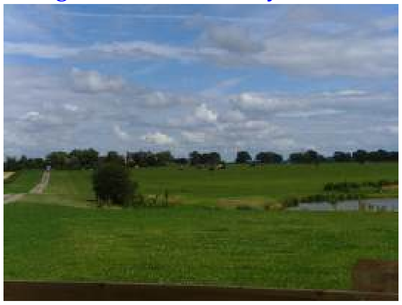

Ferney Heyes Farm © A Holmes (cc-by-sa/2.0)

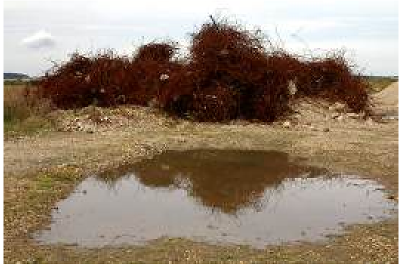

Scrap metal © Mr T (cc-by-sa/2.0)

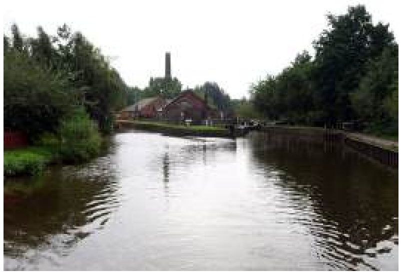

The Caldon Canal and Trent & Mersey Canal Junction © Andy Beecroft (cc-by-sa/2.0)

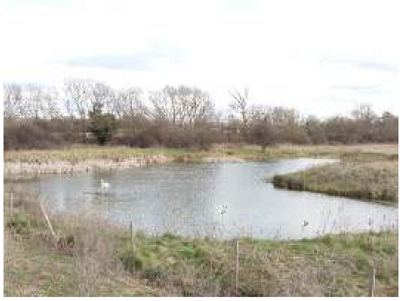

Pond on Stanwell Moor © David Hawgood (cc-by-sa/2.0)

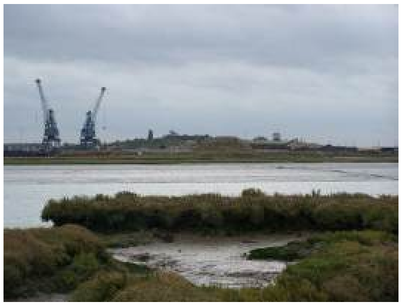

Across Long Reach in the Swale © David Anstiss (cc-by-sa/2.0)

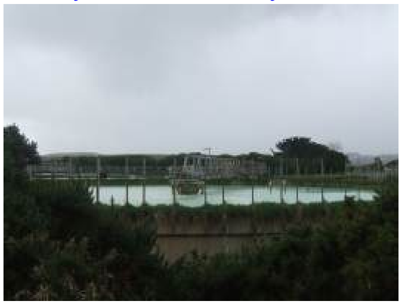

Processing tanks at Trebal Refinery © John M (cc-by-sa/2.0)

3.68 Lake

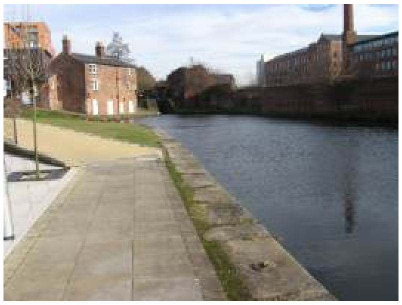

Ashton Canal near New Islington Wharf at Ancoats © Chris Wimbush (cc-by-sa/2.0)

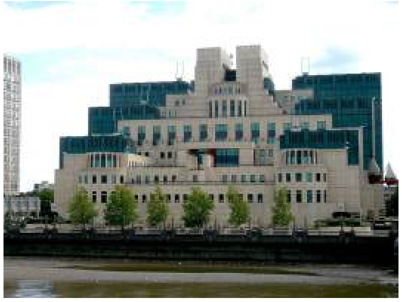

MI6 Building © Iain Crump (cc-by-sa/2.0)

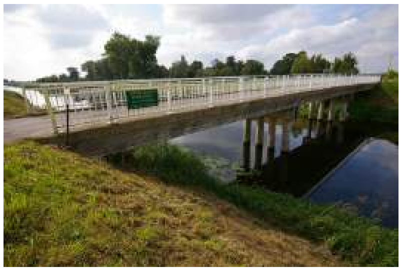

Christchurch Farm Bridge © Ben Harris (cc-by-sa/2.0)

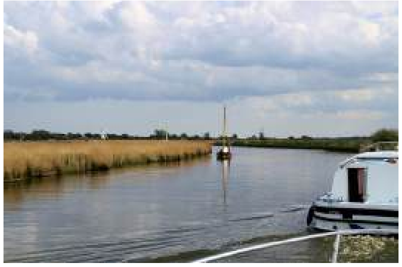

River Thurne above Thurne © Pierre Terre (cc-by-sa/2.0)

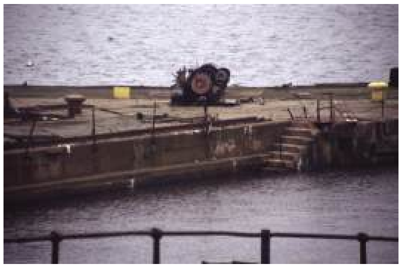

Middle Dock, South Shields © Chris Allen (cc-by-sa/2.0)

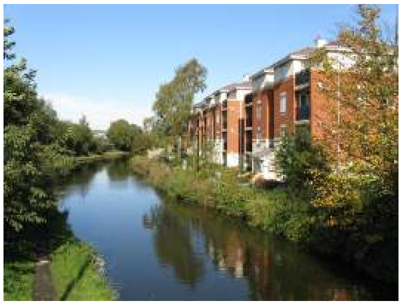

Modern Apartments by the Dudley Canal, Old Hill © Peter Whatley (cc-by-sa/2.0)

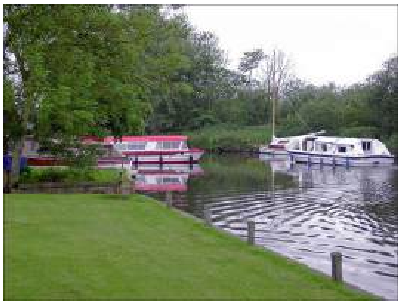

Belaugh Staithe © Renata Edge (cc-by-sa/2.0)

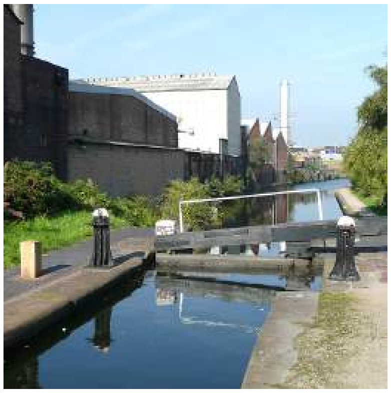

Birmingham and Fazeley Canal at Lock No 22, Aston © Roger D Kidd (cc-by-sa/2.0)

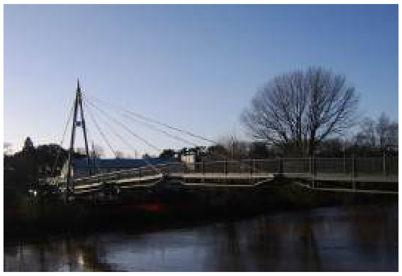

Sabrinas Bridge Over The Severn © Mr M Evison (cc-by-sa/2.0)

3.69 Land Lot

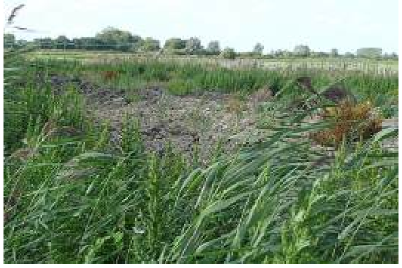

Here be dredgings © Graham Horn (cc-by-sa/2.0)

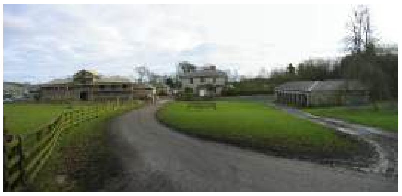

Park Farm, Hulne Park, Alnwick © Les Hull (cc-by-sa/2.0)

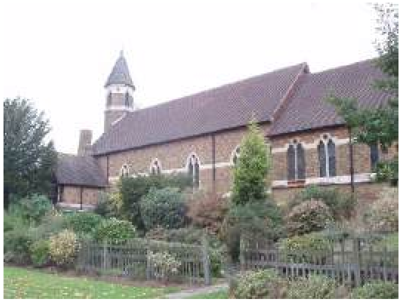

Kingsbury Parish Church, Holy Innocents © David Hawgood (cc-by-sa/2.0)

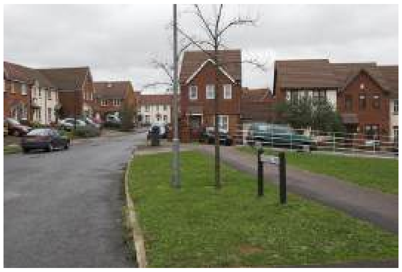

Great Ashby, Stevenage © Richard Thomas (cc-by-sa/2.0)

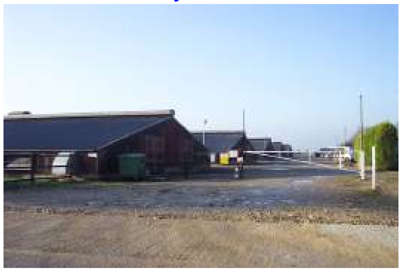

Lutton Gate poultry sheds © Jonathan Billinger (cc-by-sa/2.0)

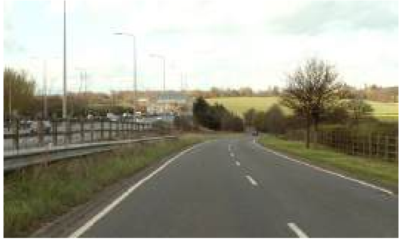

Hobbs Cross Road © Robert Edwards (cc-by-sa/2.0)

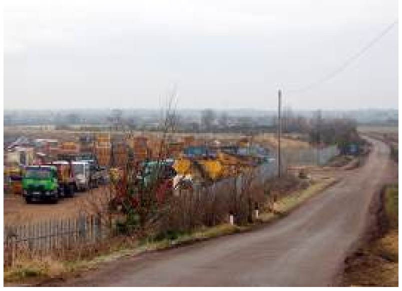

Skips galore, Napton © Andy F (cc-by-sa/2.0)

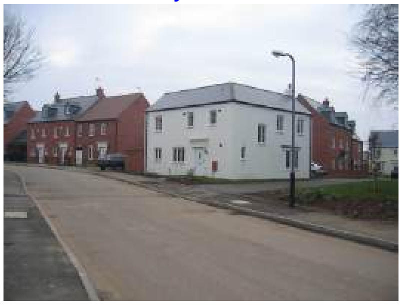

Long Fellow Road, Bridgetown © David Stowell (cc-by-sa/2.0)

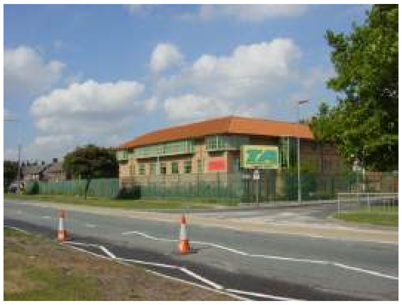

TA Centre and Field Hospital © Sue Adair (cc-by-sa/2.0)

3.70 Land Vehicle

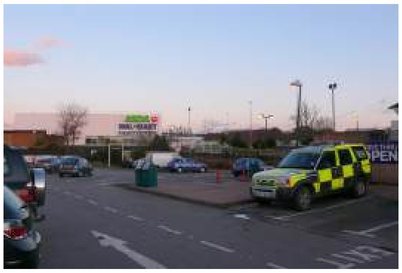

Asda, Cribbs, Causeway © Nigel Mykura (cc-by-sa/2.0)

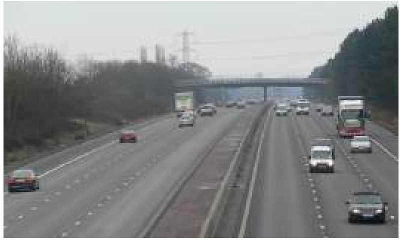

South along the M1 Motorway © Mat Fascione (cc-by-sa/2.0)

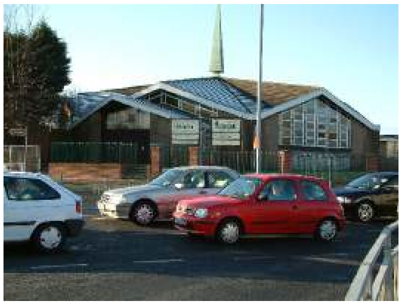

Our Lady of Walsingham © Peter Hodge (cc-by-sa/2.0)

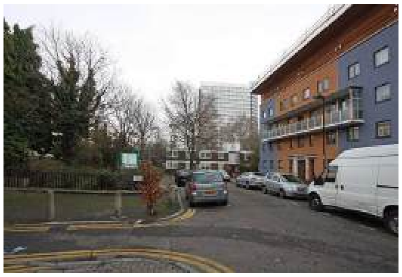

Roscoe Street, Finsbury © John Salmon (cc-by-sa/2.0)

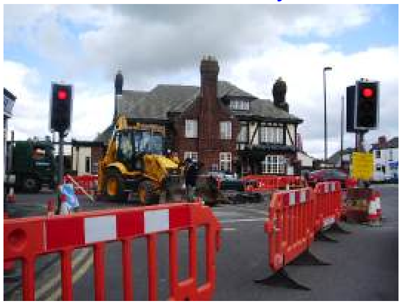

"The Stag Inn" Orrell Post, Orrell © Alexander P Kapp (cc-by-sa/2.0)

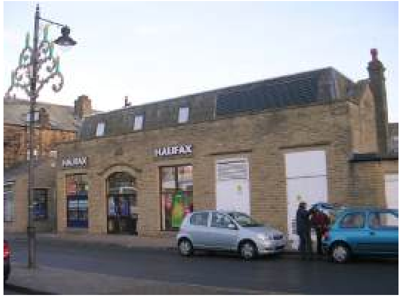

HBOS - Market Place © Betty Longbottom (cc-by-sa/2.0)

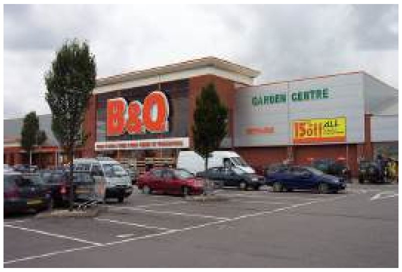

B & Q Superstore, Bristol © Jonathan Billinger (cc-by-sa/2.0)

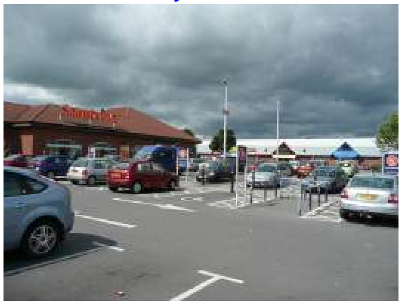

Sainsbury's Store © Jonathan Billinger (cc-by-sa/2.0)

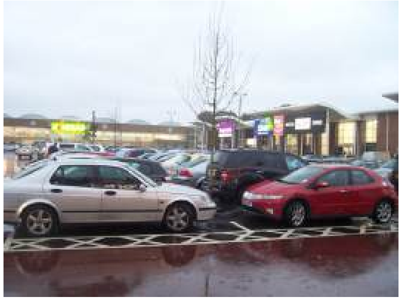

Bournemouth : Castlemore Retail Park © Lewis Clarke (cc-by-sa/2.0)

3.71 Landmark

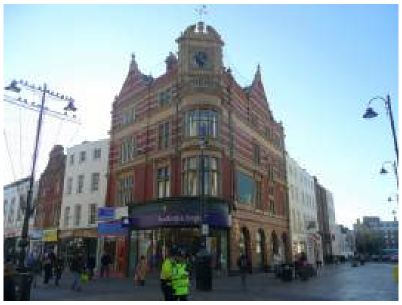

Classic bank building © Jonathan Billinger (cc-by-sa/2.0)

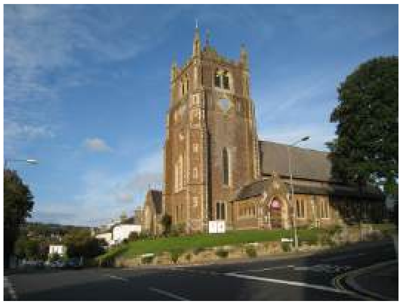

Christ Church, Blacklands, Laton Road, Hastings, East Sussex © Oast House Archive (cc-by-sa/2.0)

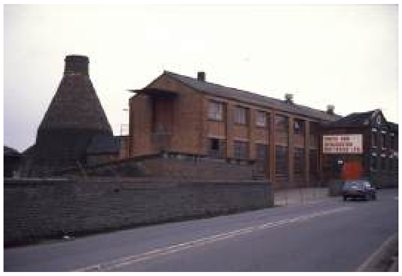

Price & Kensington, The National Teapot Works © Chris Allen (cc-by-sa/2.0)

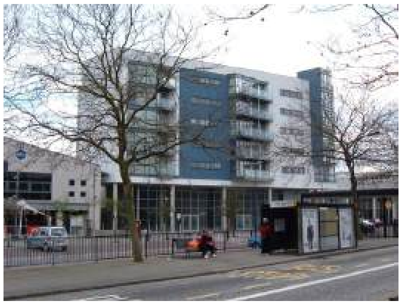

New residential & retail building © Mr Biz (cc-by-sa/2.0)

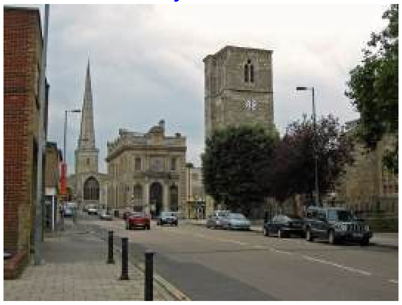

Holyrood Church, Southampton © southpix (cc-by-sa/2.0)

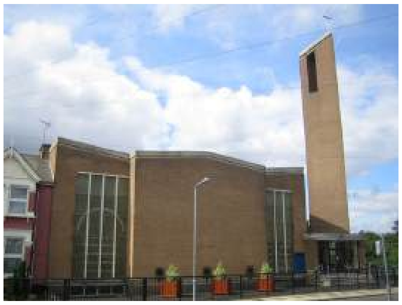

Ilford: St John the Baptist's Catholic Church © Nigel Cox (cc-by-sa/2.0)

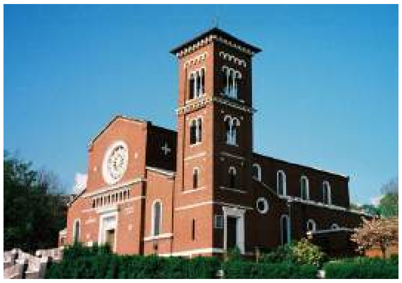

Sheffield: attractive red-brick church © Chris Downer (cc-by-sa/2.0)

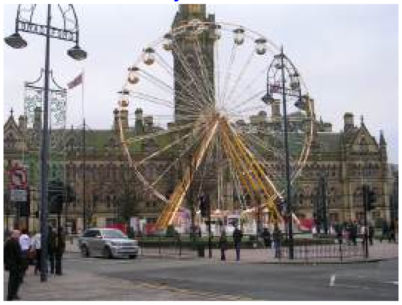

Giant Wheel - Centenary Square 2008 © Betty Longbottom (cc-by-sa/2.0)

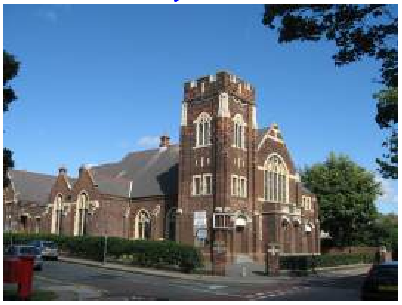

Aigburth Methodist Church © Sue Adair (cc-by-sa/2.0)

3.72 Landscape

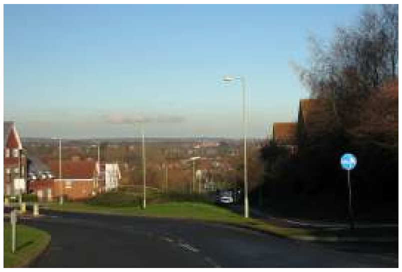

Bucksford Lane, Ashford, Kent © Oast House Archive (cc-by-sa/2.0)

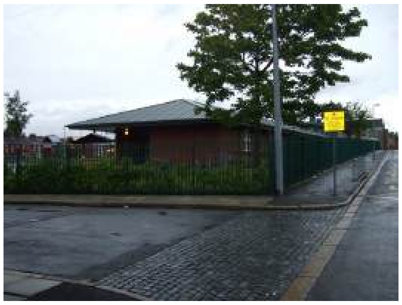

Heygreen School © Colin Pyle (cc-by-sa/2.0)

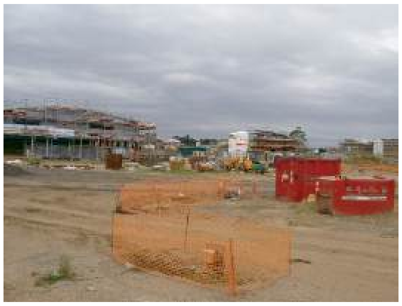

Building a Barracks © Glyn Baker (cc-by-sa/2.0)

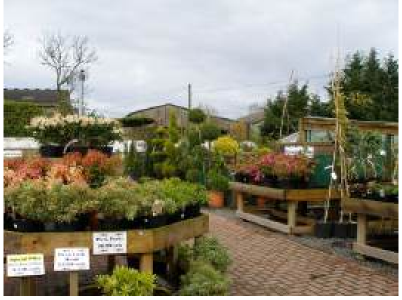

Wilkinson's Garden Centre based at East Brocks Farm © Carol Rose (cc-by-sa/2.0)

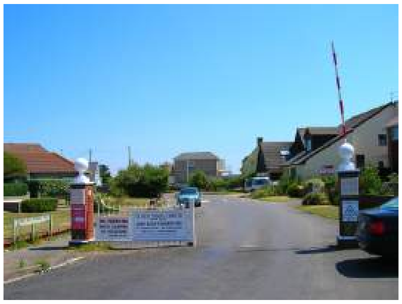

Elmer Sands, Elmer © Simon Carey (cc-by-sa/2.0)

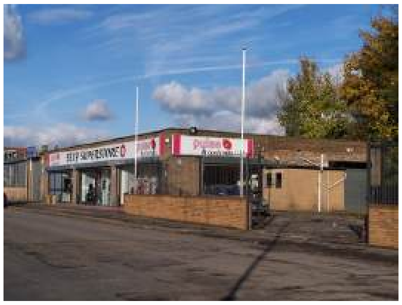

Pulse & Cocktails, Penistone Road, Sheffield © Terry Robinson (cc-by-sa/2.0)

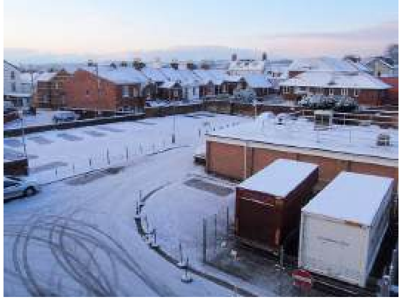

Car park behind St Thomas' station, Exeter © Derek Harper (cc-by-sa/2.0)

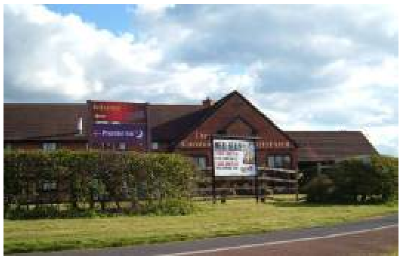

Premier Inn Crewe © Margaret Sutton (cc-by-sa/2.0)

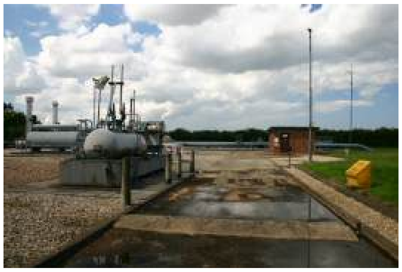

Harper's Green pumping station © Bob Jones (cc-by-sa/2.0)

3.73 Landscaping

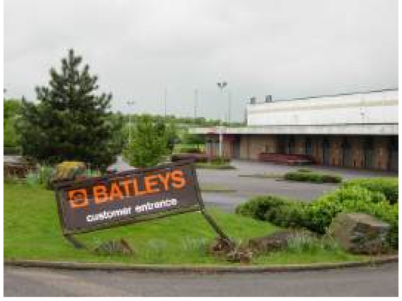

Batleys Cash & Carry, Cross Point Business Park © Stephen McKay (cc-by-sa/2.0)

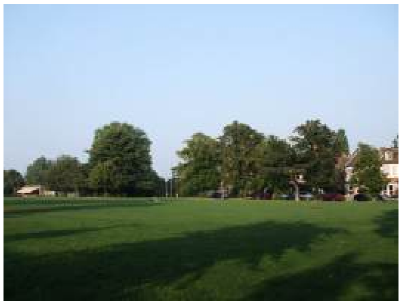

Alexandra Road recreation ground (3) © Linda Craven (cc-by-sa/2.0)

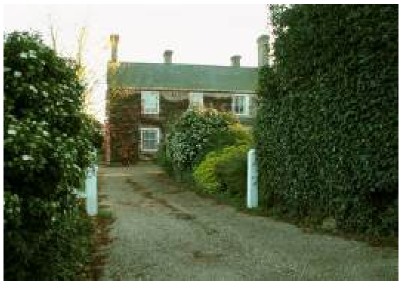

Farmhouse at Spring Farm © Robert Edwards (cc-by-sa/2.0)

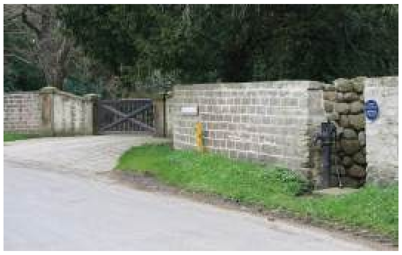

Village Pump © David Rogers (cc-by-sa/2.0)

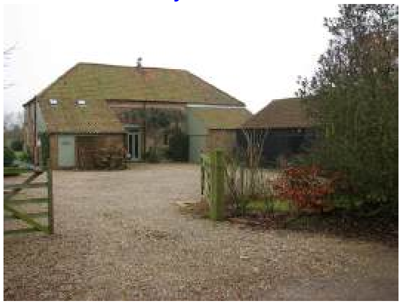

An attractive converted barn © Evelyn Simak (cc-by-sa/2.0)

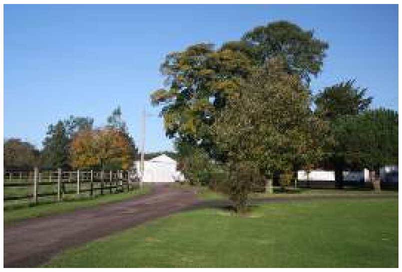

Approach to Hall Farm, West Wretham © Bob Jones (cc-by-sa/2.0)

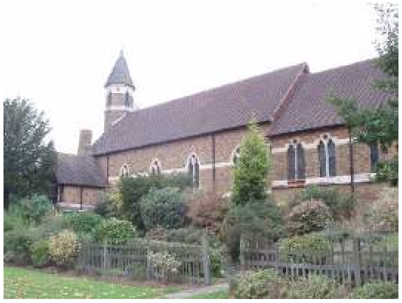

Kingsbury Parish Church, Holy Innocents © David Hawgood (cc-by-sa/2.0)

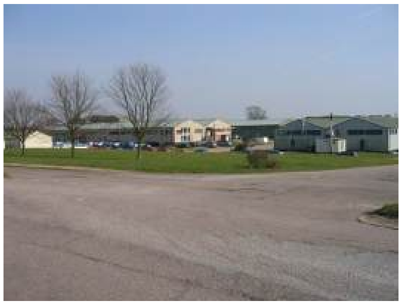

Factory, Tattersett Industrial and Retail Park © Nigel Jones (cc-by-sa/2.0)

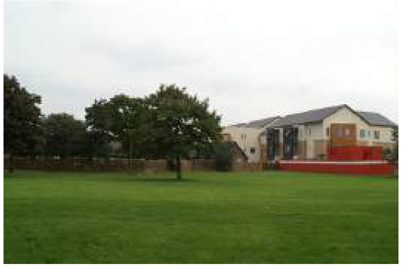

Change in view © David Long (cc-by-sa/2.0)

3.74 Lane

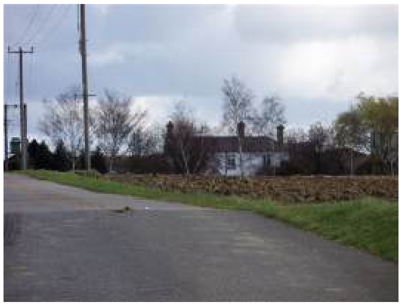

Farm © d brewerton (cc-by-sa/2.0)

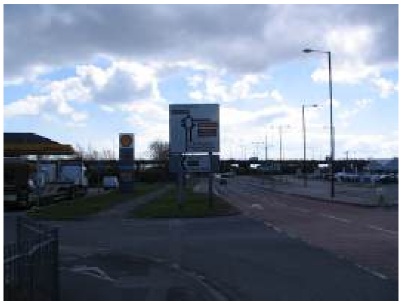

Eastern Road / A27 junction © Andy W (cc-by-sa/2.0)

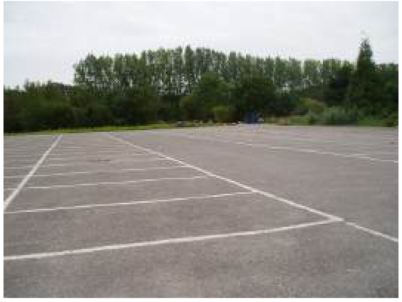

Car park and beyond © Peter Holmes (cc-by-sa/2.0)

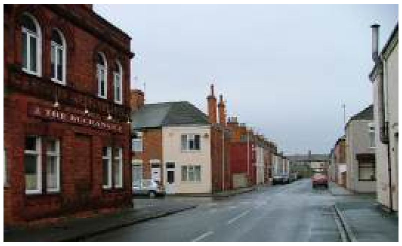

The Buchanan and Gray Street © Mick Garratt (cc-by-sa/2.0)

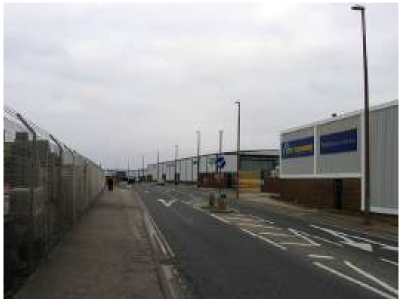

Malthouse Industrial Estate, Brighton Road © Simon Carey (cc-by-sa/2.0)

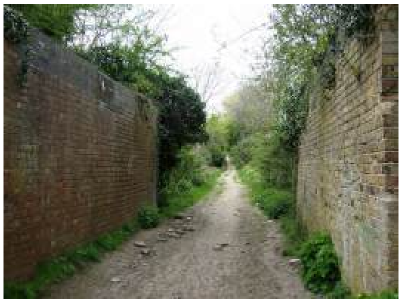

Old railway bridge approaching Stamford © Graham Horn (cc-by-sa/2.0)

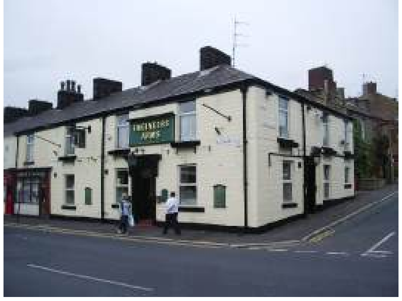

Engineers Arms, Duckworth Street, Darwen © Alexander P Kapp (cc-by-sa/2.0)

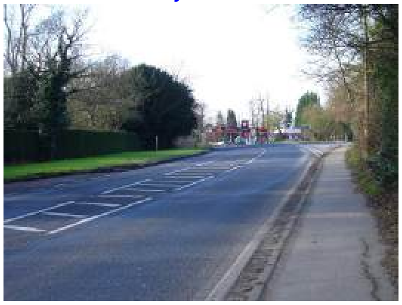

Petrol station on the A20 © Penny Mayes (cc-by-sa/2.0)

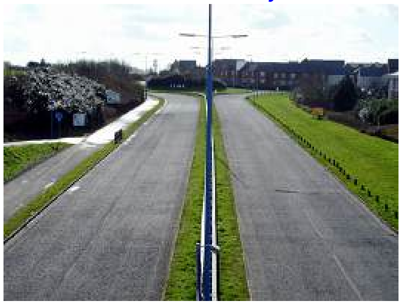

Looking south along Great Stoke Way © Linda Bailey (cc-by-sa/2.0)

3.75 Lawn

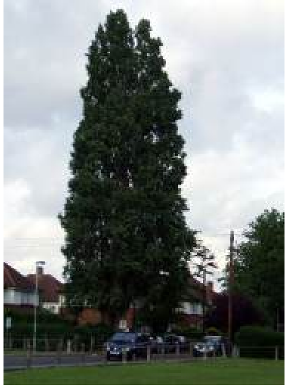

Ancient Lombardy Poplar Tree. © Graham Newell (cc-by-sa/2.0)

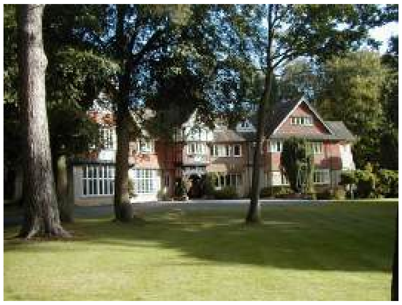

Branksome Conference & Training Centre © Martin Elliff (cc-by-sa/2.0)

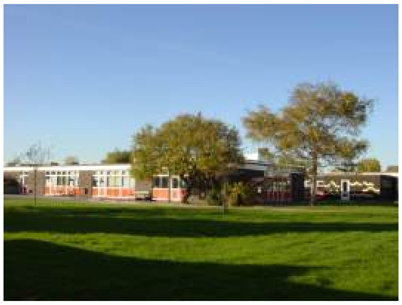

Sandbrook Primary School, Stavordale Road © Sue Adair (cc-by-sa/2.0)

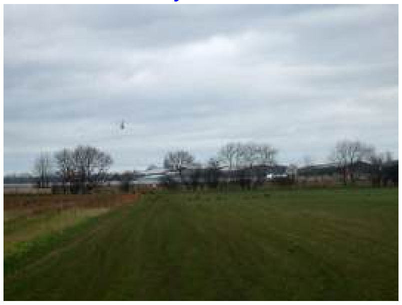

Helicopter Search around Full Sutton Industrial Estate © Keith Laverack (cc-by-sa/2.0)

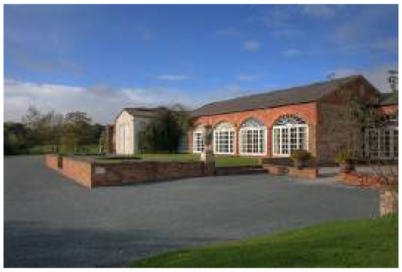

The Orangery, Camp Hill © Mick Garratt (cc-by-sa/2.0)

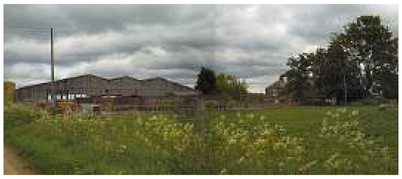

Panorama of Givendale Grange farm © Duncan Lilly (cc-by-sa/2.0)

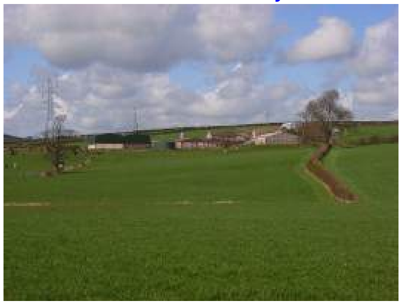

Bank House © Andrew Smith (cc-by-sa/2.0)

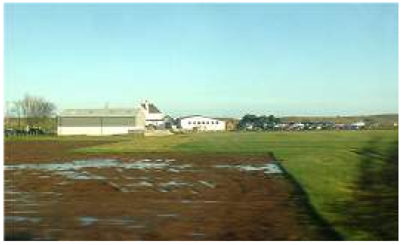

Goswick Golfers' Clubhouse © Stanley Howe (cc-by-sa/2.0)

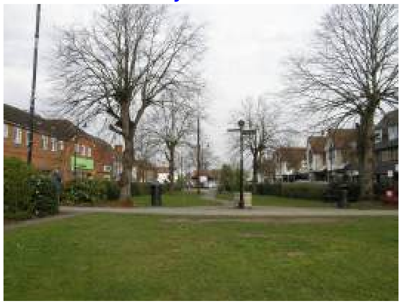

Thatcham: The Broadway © Chris Downer (cc-by-sa/2.0)

3.76 Leisure

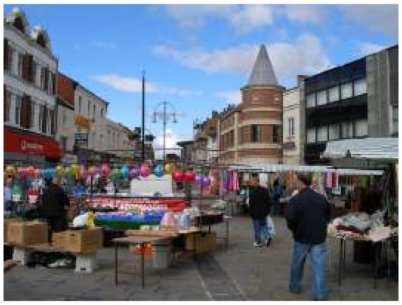

Dovecot Street from the marketplace © Carol Rose (cc-by-sa/2.0)

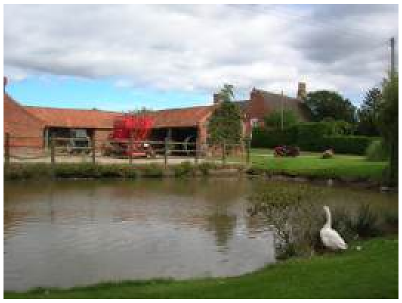

Berry Hall © DS Pugh (cc-by-sa/2.0)

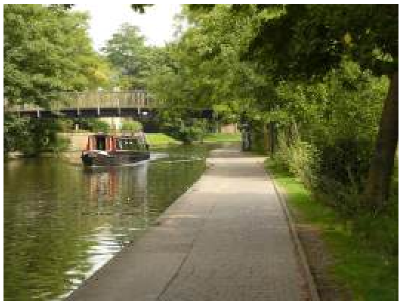

Nottingham Canal © Andy Jamieson (cc-by-sa/2.0)

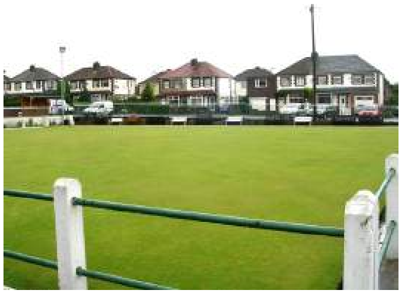

Old Bank Bowling Green - Sunny Bank Avenue, Sunny Bank Road © Betty Longbottom (cc-by-sa/2.0)

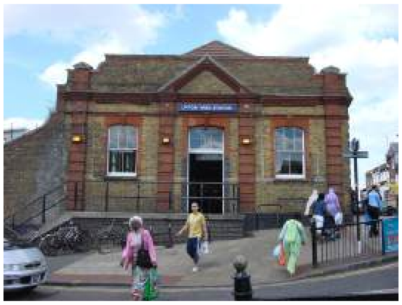

Upton Park tube station, Queen's Road entrance © Oxyman (cc-by-sa/2.0)

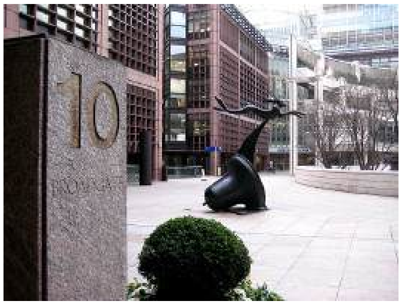

Hare and Bell sculpture . . . © Zorba the Geek (cc-by-sa/2.0)

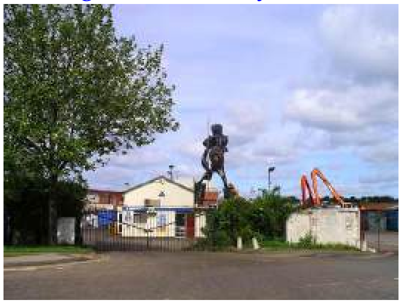

Sims Metal Recycling, Mansfield Road, Derby © mike smith (cc-by-sa/2.0)

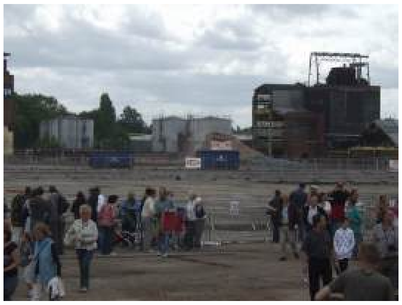

After the dust had settled © John M (cc-by-sa/2.0)

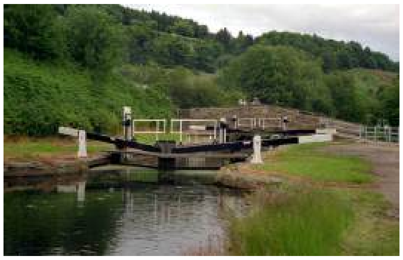

Riddings Lock No 6, Huddersfield Broad Canal © Dr Neil Clifton (cc-by-sa/2.0)

3.77 Lighting

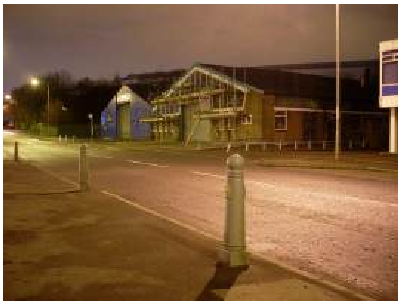

Industrial properties on Saltmeadows Road © Stephen Sweeney (cc-by-sa/2.0)

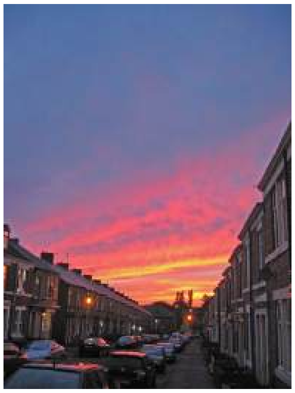

Northbourne Street, Gateshead. © wfmillar (cc-by-sa/2.0)

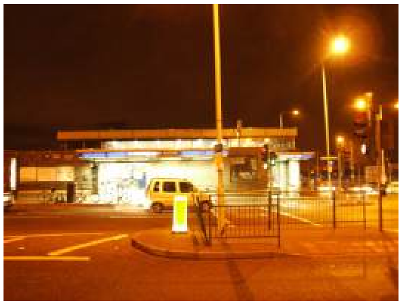

Blackhorse Road Underground Station, Walthamstow © Chris Whippet (cc-by-sa/2.0)

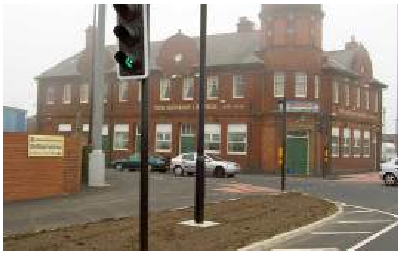

The Hendon Grange Free House © Steve Fareham (cc-by-sa/2.0)

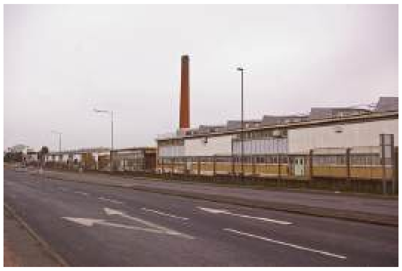

Thales © Ian Capper (cc-by-sa/2.0)

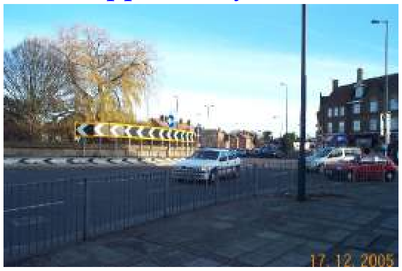

Mill Hill: Apex Corner, NW7 © Nigel Cox (cc-by-sa/2.0)

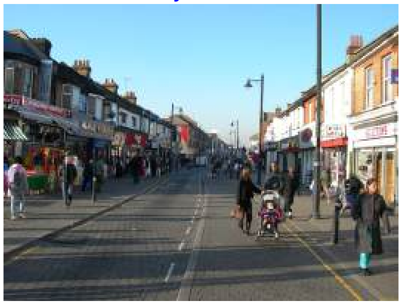

High Street North, E6 © Danny P Robinson (cc-by-sa/2.0)

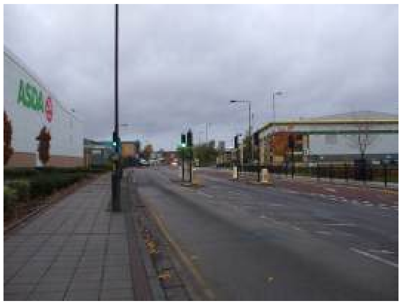

Coronation Road, NW10 © Phillip Perry (cc-by-sa/2.0)

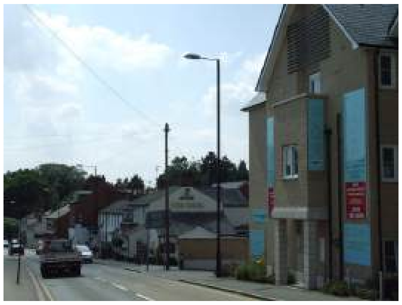

Silver Street © Thomas Nugent (cc-by-sa/2.0)

3.78 Line

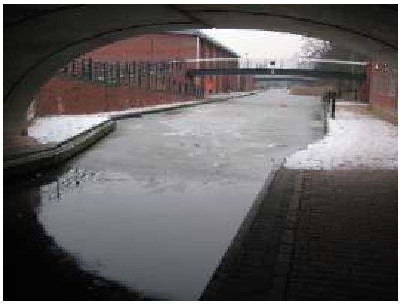

Worcester and Birmingham Canal © Philip Halling (cc-by-sa/2.0)

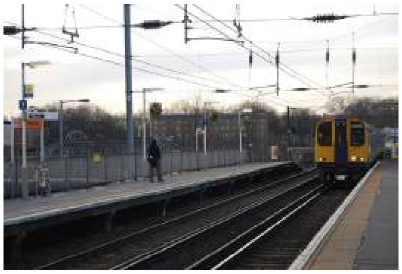

Stratford train arriving at Hackney Wick Station © N Chadwick (cc-by-sa/2.0)

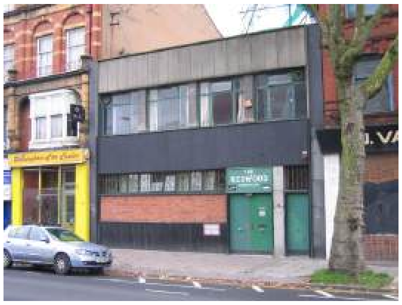

Midland Bank Bristol Street. Sorting code 40-11-39 © Roy Hughes (cc-by-sa/2.0)

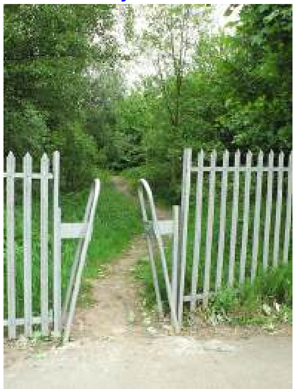

Squeeze stile by Ring Road, West Park © Rich Tea (cc-by-sa/2.0)

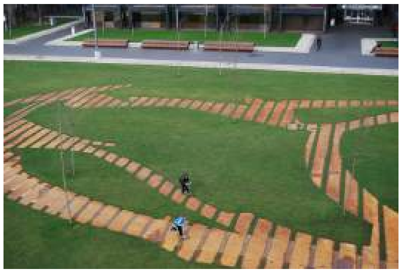

Open space, in front of MIMA © hayley green (cc-by-sa/2.0)

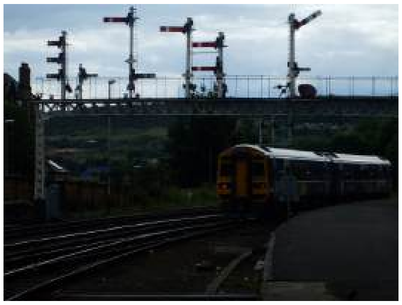

Scarborough station signal gantry © Ashley Dace (cc-by-sa/2.0)

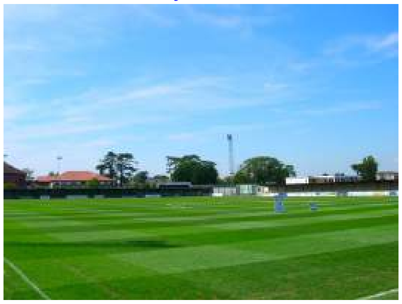

Nyewood Lane, Bognor Regis Town FC © Simon Carey (cc-by-sa/2.0)

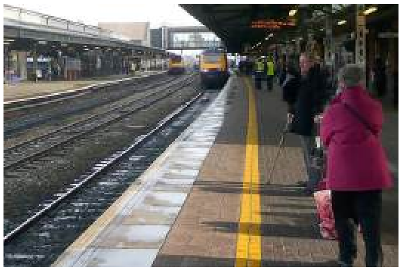

Reading station © Graham Horn (cc-by-sa/2.0)

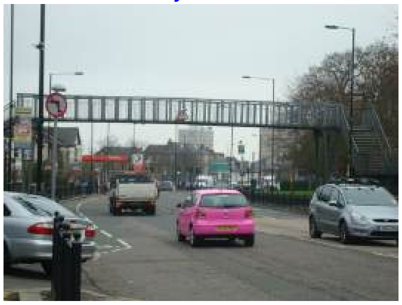

Bounds Green Road, London N11 © Stacey Harris (cc-by-sa/2.0)

3.79 Machine

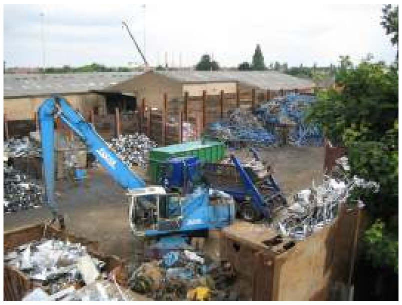

Edmonton: Scrap metal merchants © Nigel Cox (cc-by-sa/2.0)

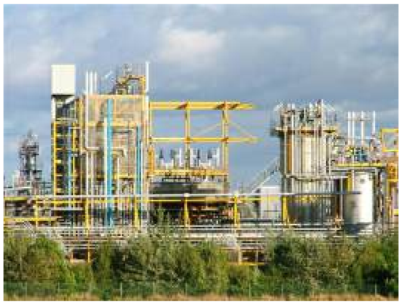

Hydro Polymers PVC Plant © Mick Garratt (cc-by-sa/2.0)

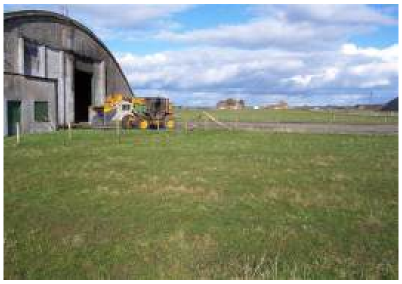

Outlying Hangar © John Holmes (cc-by-sa/2.0)

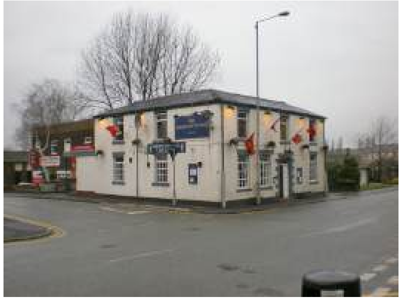

The Marston Tavern, Rochdale Road © Alexander P Kapp (cc-by-sa/2.0)

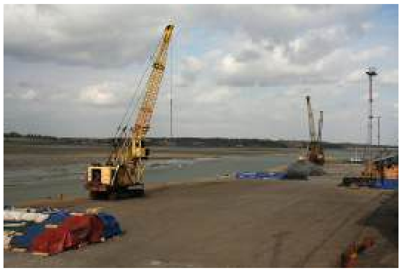

Mistley Quay © Bob Jones (cc-by-sa/2.0)

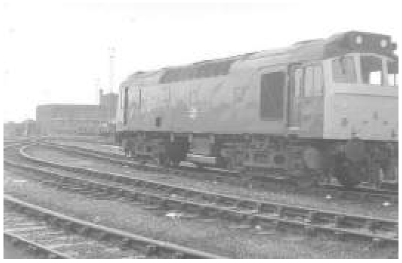

Newton Heath railway lands © Peter Whatley (cc-by-sa/2.0)

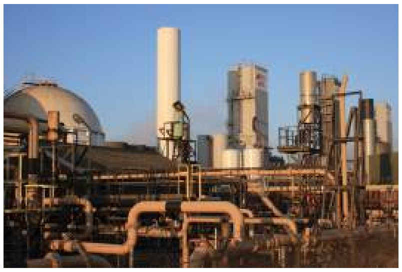

B.O.C Works © Mick Garratt (cc-by-sa/2.0)

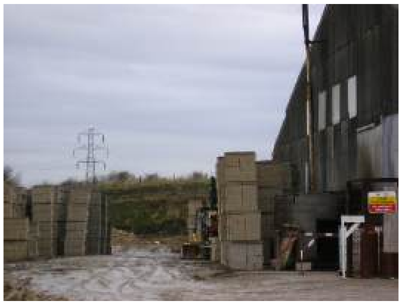

Westby brickworks © Roger W Haworth (cc-by-sa/2.0)

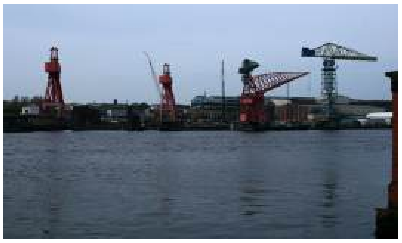

Swan Hunter © Peter McDermott (cc-by-sa/2.0)

3.80 Manor House

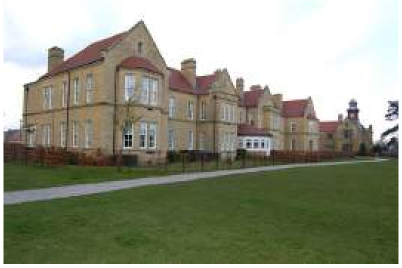

Former Scalebor Hospital © John Sparshatt (cc-by-sa/2.0)

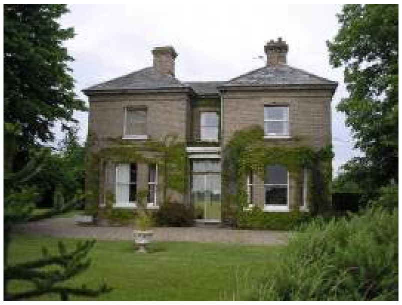

Morehams Hall © NA (cc-by-sa/2.0)

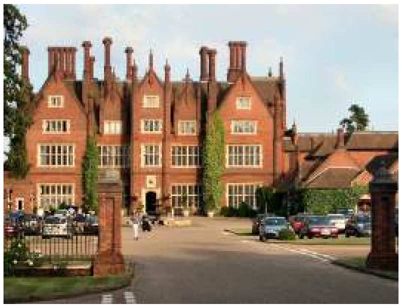

Dunston Hall Hotel © Lis Burke (cc-by-sa/2.0)

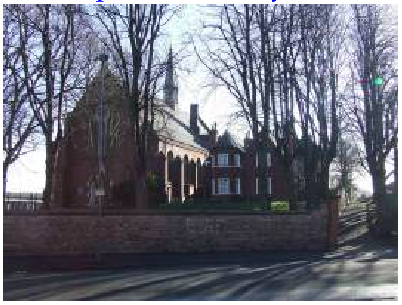

St Mary's Church and Rectory. Grimsby © David Hebb (cc-by-sa/2.0)

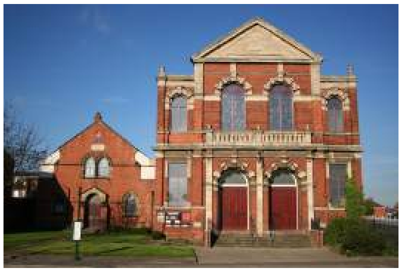

Ashby Wesleyan Methodist Church © Richard Croft (cc-by-sa/2.0)

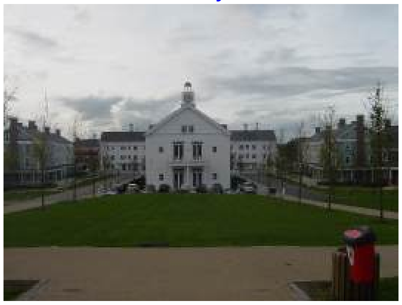

The Hamptons © Tony Grant (cc-by-sa/2.0)

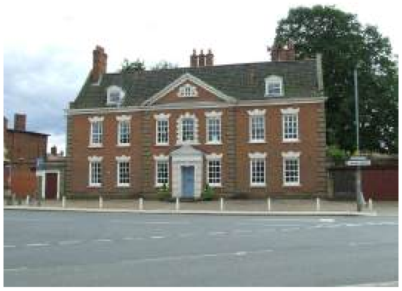

Oakleigh House © Keith Evans (cc-by-sa/2.0)

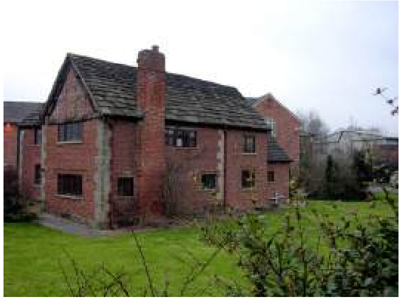

"Brook Farm" , Handforth © Terry Walsh (cc-by-sa/2.0)

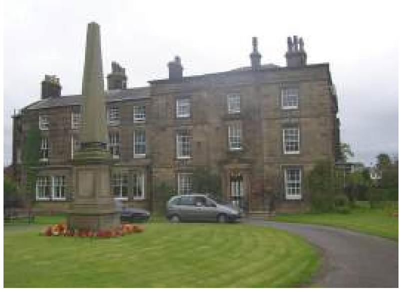

The Bushell Hospital, Goosnargh © Humphrey Bolton (cc-by-sa/2.0)

3.81 Meadow

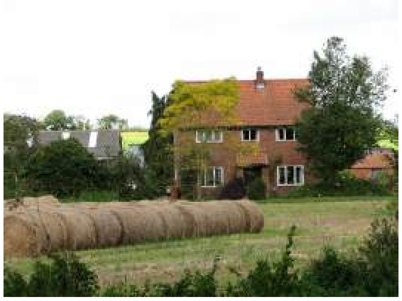

Langmere Farm © Evelyn Simak (cc-by-sa/2.0)

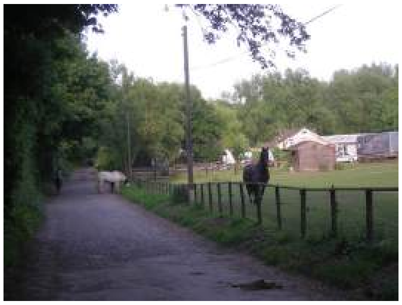

Telford Equestrian Centre © Row17 (cc-by-sa/2.0)

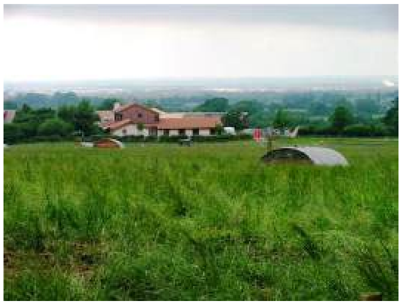

Larchfield Community © Mick Garratt (cc-by-sa/2.0)

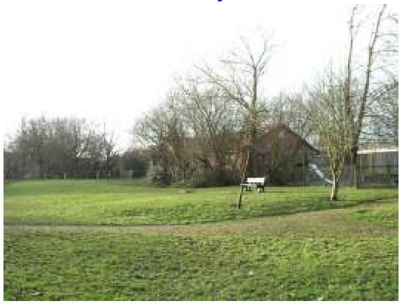

Hookwood Memorial Hall © Andy Potter (cc-by-sa/2.0)

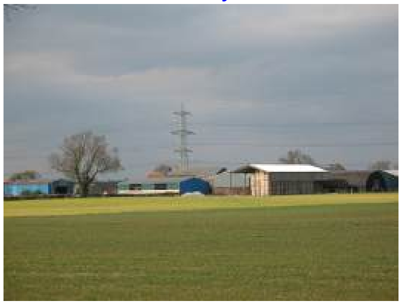

Forest Hall Farm near Alne © Gordon Hatton (cc-by-sa/2.0)

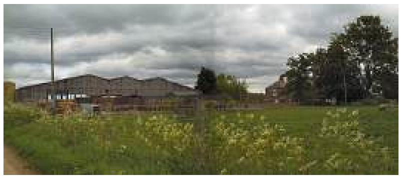

Panorama of Givendale Grange farm © Duncan Lilly (cc-by-sa/2.0)

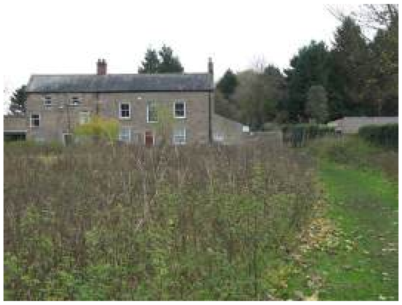

Cottages at the west end of Tyne Green © Mike Quinn (cc-by-sa/2.0)

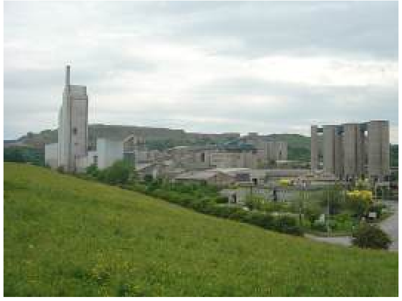

Cauldon Cement Works © Alan Murray-Rust (cc-by-sa/2.0)

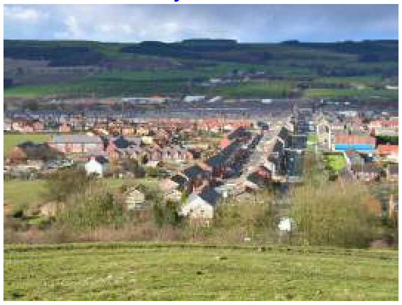

Langley Park © Oliver Dixon (cc-by-sa/2.0)

3.82 Metropolis

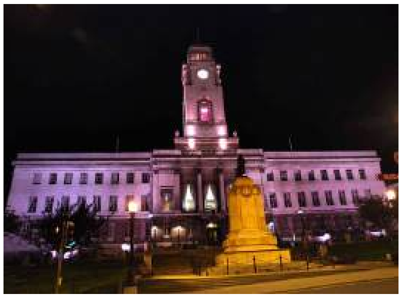

Barnsley Town Hall front elevation © Steve Fareham (cc-by-sa/2.0)

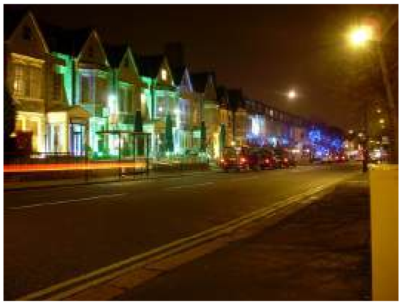

Osborne Road, Jesmond © Stephen Sweeney (cc-by-sa/2.0)

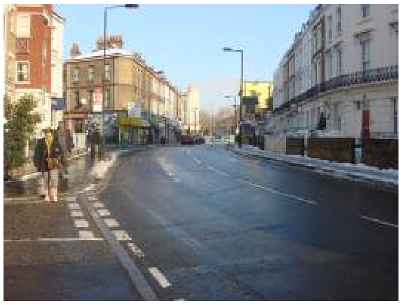

Great Western Road © Oxyman (cc-by-sa/2.0)

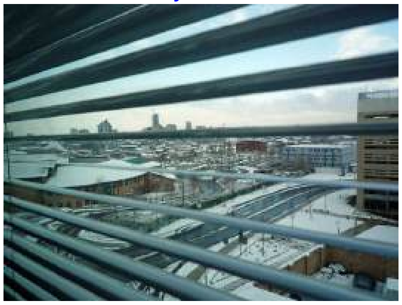

Outlook from Ward 82 Bexley Wing, St James Hospital © Julian Paren (cc-by-sa/2.0)

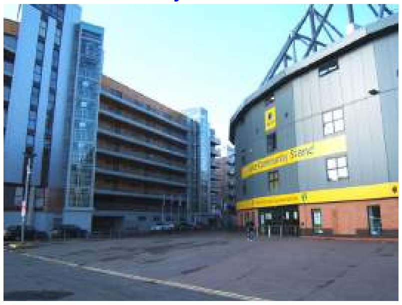

Norwich City FC, Carrow Road © Martin Thirkettle (cc-by-sa/2.0)

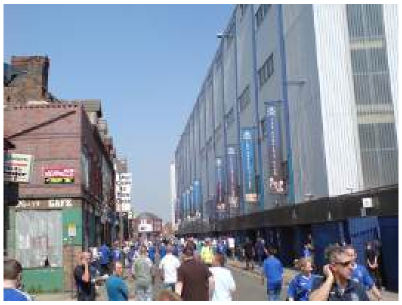

Goodison Road, Liverpool L4 © Frank Glover (cc-by-sa/2.0)

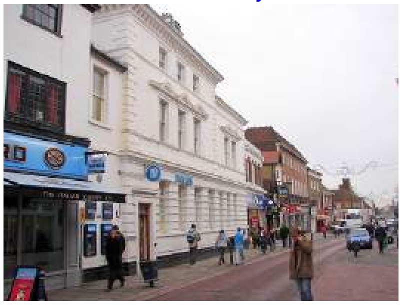

Barclays Bank, Hitchin © John Lucas (cc-by-sa/2.0)

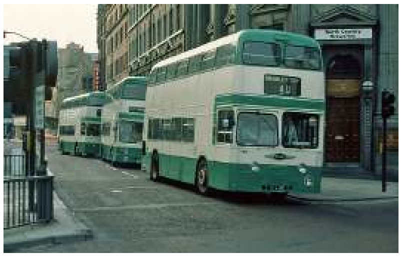

Buses on Boar Lane © Martin Addison (cc-by-sa/2.0)

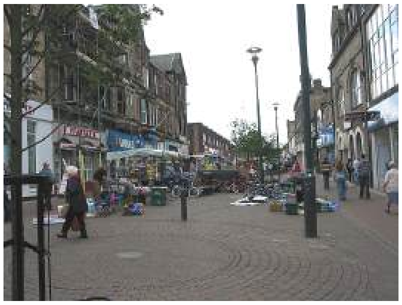

Middle Street, Consett © Pauline E (cc-by-sa/2.0)

3.83 Metropolitan Area

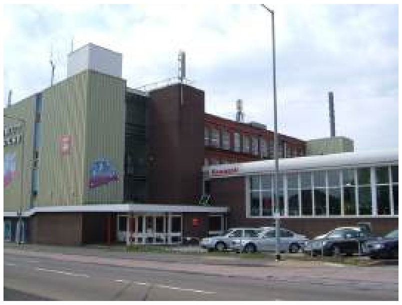

Swift House, Liverpool Rd, Newcastle under Lyme © Derek Harper (cc-by-sa/2.0)

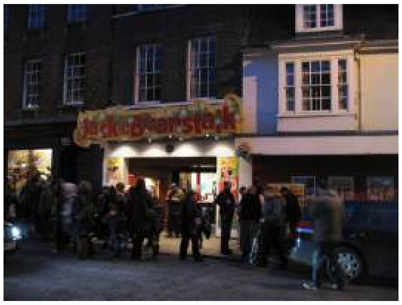

Cambridge panto season © Mr Ignavy (cc-by-sa/2.0)

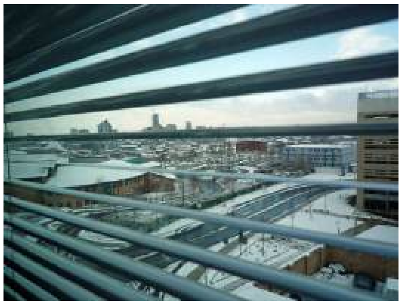

Outlook from Ward 82 Bexley Wing, St James Hospital © Julian Paren (cc-by-sa/2.0)

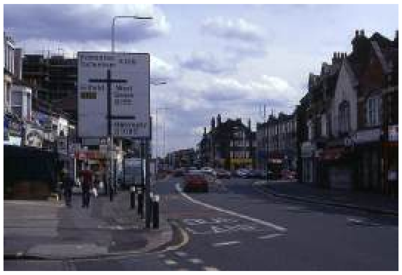

A109, Lordship Lane © Chris Heaton (cc-by-sa/2.0)

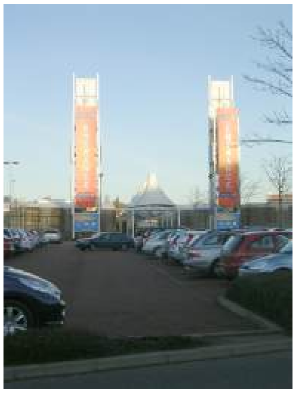

Junction 32 Retail Outlet - Glasshoughton © Betty Longbottom (cc-by-sa/2.0)

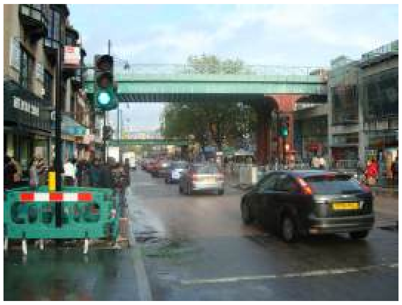

Brixton Town Centre © Stacey Harris (cc-by-sa/2.0)

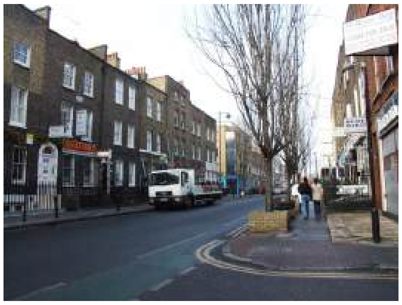

New Road, Whitechapel © Chris Whippet (cc-by-sa/2.0)

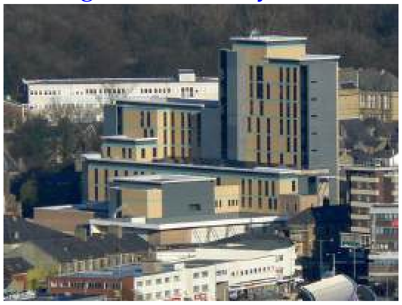

Modern Development in Burnley © Chris Tomlinson (cc-by-sa/2.0)

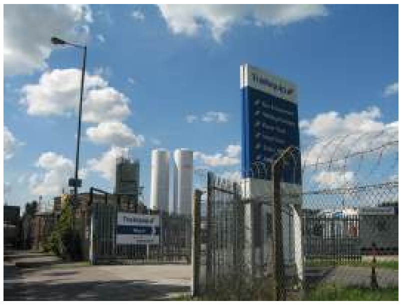

BOC Tradequip, St Helens © Sue Adair (cc-by-sa/2.0)

3.84 Mixed Use

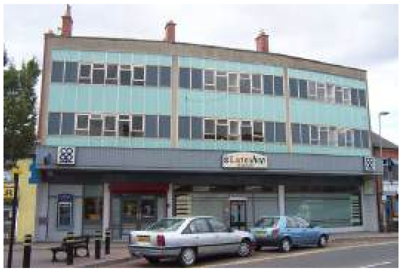

Barwell Co-op © David Walton (cc-by-sa/2.0)

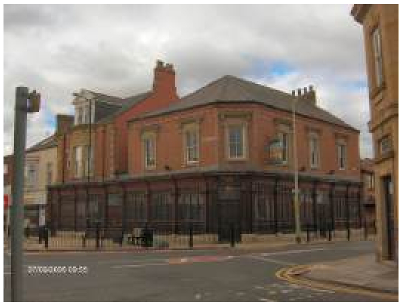

Adam & Eve - Laygate © Bill Richardson (cc-by-sa/2.0)

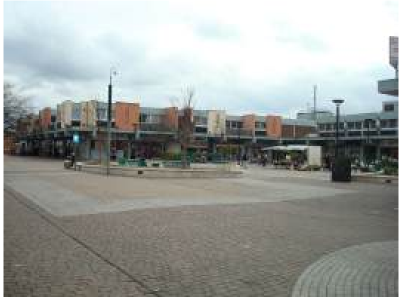

Shopping Centre, London Road, Swanley © Stacey Harris (cc-by-sa/2.0)

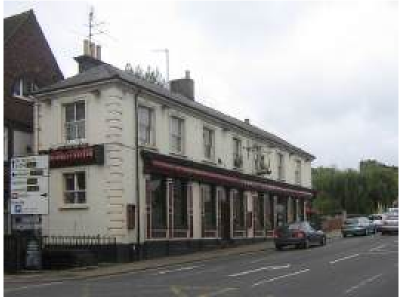

The Compleat Angler © Ian Capper (cc-by-sa/2.0)

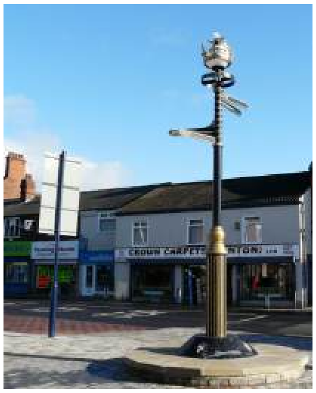

The Crown Pole © Gerald England (cc-by-sa/2.0)

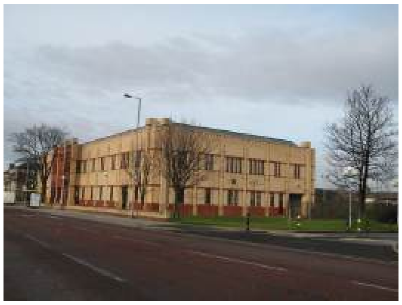

South Sefton Magistrate's Court © Sue Adair (cc-by-sa/2.0)

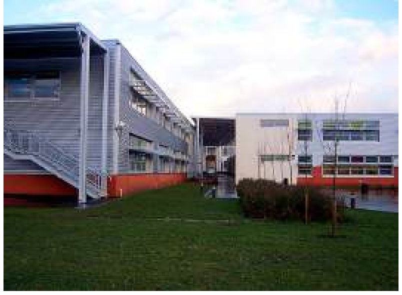

Holmesdale Technology College, Snodland © Richard Dorrell (cc-by-sa/2.0)

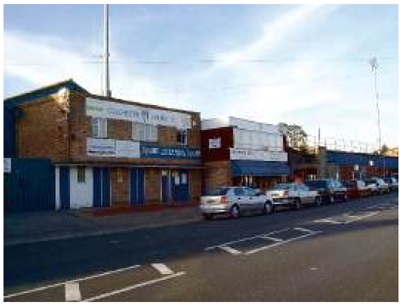

Colchester United's Layer Rd Ground © Glyn Baker (cc-by-sa/2.0)

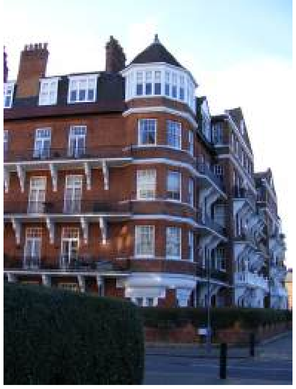

Mansion Block Prince of Wales Drive SW11 © PAUL FARMER (cc-by-sa/2.0)

3.85 Mode of Transport

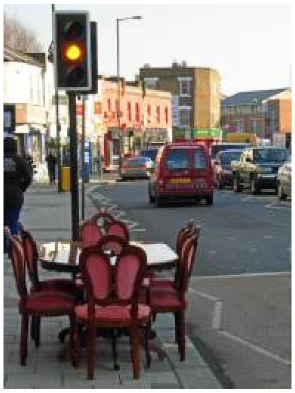

Harrow Road, Kensal Green © Stephen McKay (cc-by-sa/2.0)

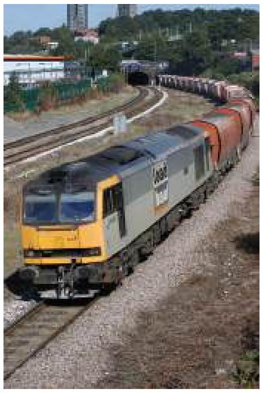

EWS Freight Train © Mr B Wilder (cc-by-sa/2.0)

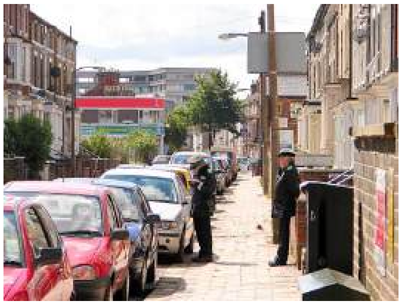

Alexandra Road, Bedford © John Lucas (cc-by-sa/2.0)

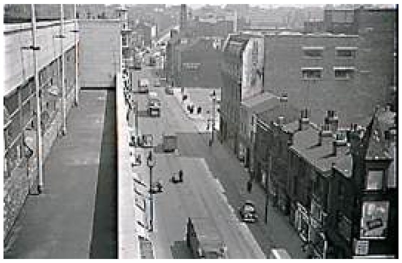

London Road, Liverpool, 1955 © Gordon Cragg (cc-by-sa/2.0)

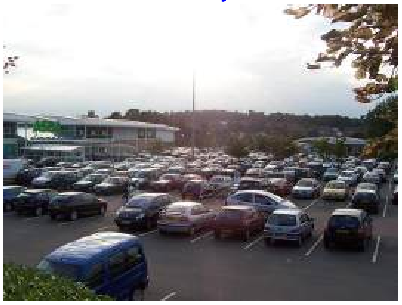

Asda, Queslett © Adrian Bailey (cc-by-sa/2.0)

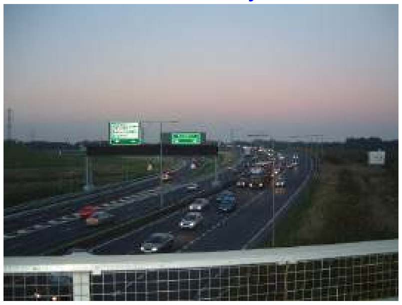

Rush Hour Traffic © Steve McShane (cc-by-sa/2.0)

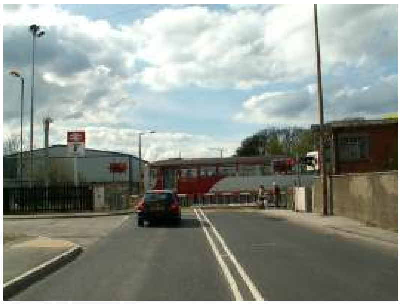

The 16:10 arriving at Dodworth Station © John Fielding (cc-by-sa/2.0)

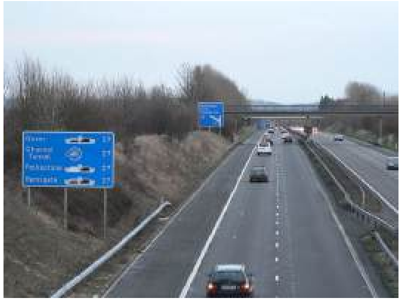

27 miles from everywhere © Chris Downer (cc-by-sa/2.0)

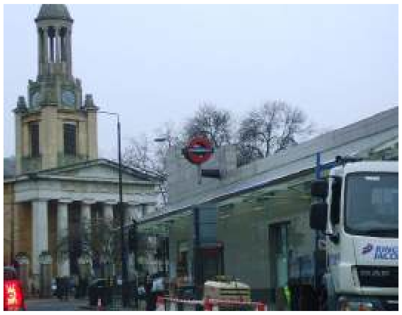

Oval station, SE11 © Phillip Perry (cc-by-sa/2.0)

3.86 Monochrome

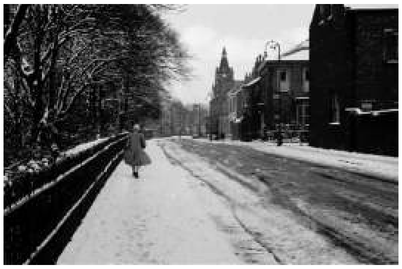

Winter in Sunderland © N T Stobbs (cc-by-sa/2.0)

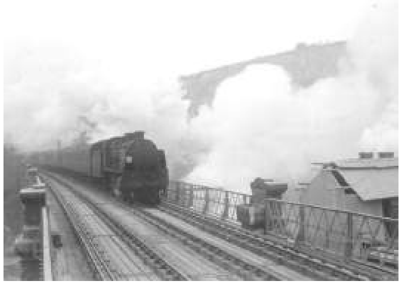

Down steam train on Riddlesdown Viaduct © Tudor Hughes (cc-by-sa/2.0)

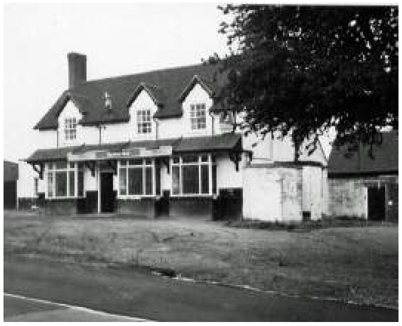

The Robin Hood, Merry Hill © Brian Clift (cc-by-sa/2.0)

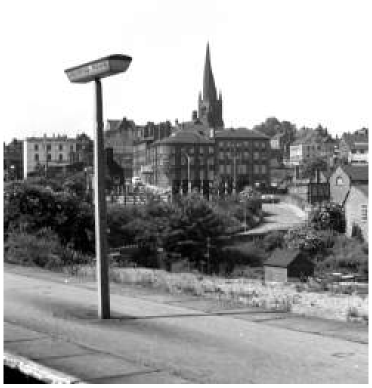

Crooked Spire from Chesterfield Midland Station © Andrew Tatlow (cc-by-sa/2.0)

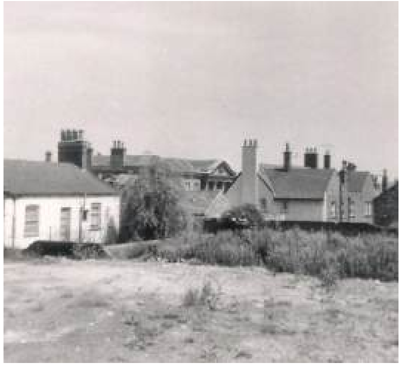

Back of Front Street © Gerald England (cc-by-sa/2.0)

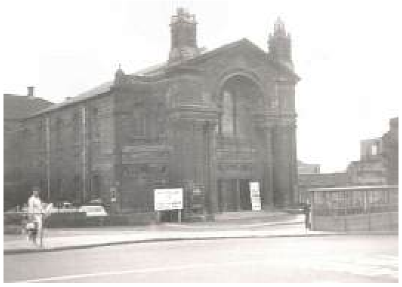

Burslem Central Mission © Gerald England (cc-by-sa/2.0)

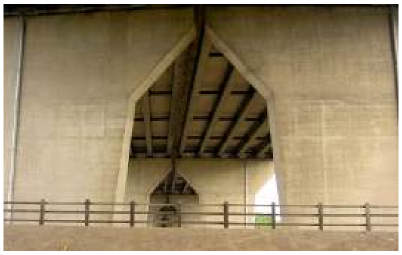

Supporting the motorways © Steve Fareham (cc-by-sa/2.0)

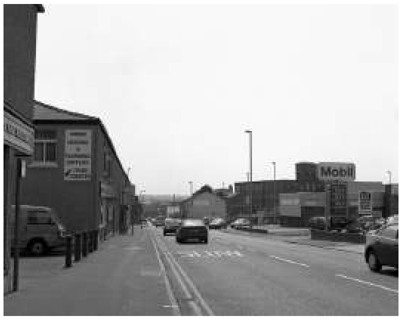

Whitworth Road, Rochdale © Dr Neil Clifton (cc-by-sa/2.0)

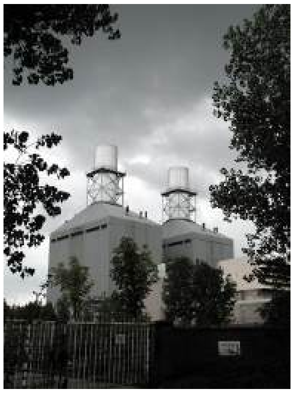

Little Barford Power Station © Paul Glazzard (cc-by-sa/2.0)

3.87 Monument

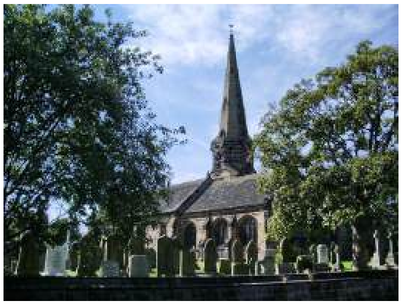

St Michael, Aughton Parish Church © Alexander P Kapp (cc-by-sa/2.0)

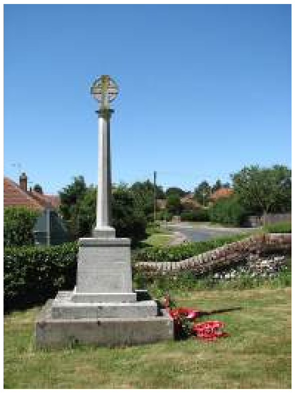

St Andrew's church - war memorial © Evelyn Simak (cc-by-sa/2.0)

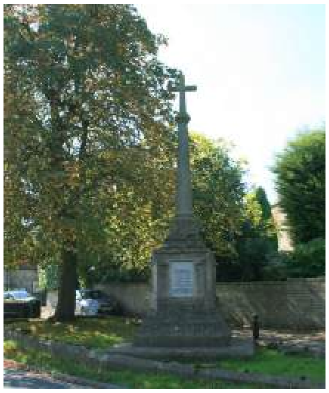

2008 : War Memorial, Market Place, Colerne © Maurice Pullin (cc-by-sa/2.0)

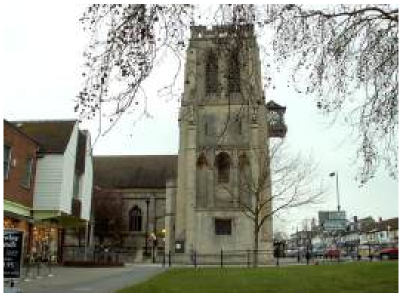

St. John the Baptist church in Epping High Street © Robert Edwards (cc-by-sa/2.0)

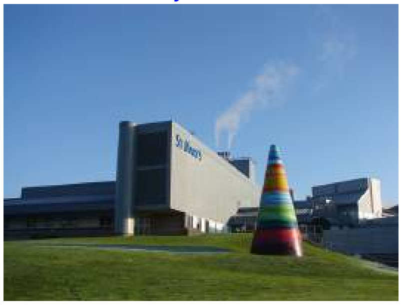

St Mary's Hospital © Mark Pilbeam (cc-by-sa/2.0)

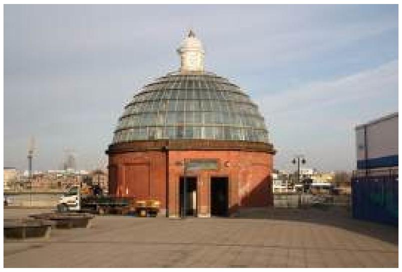

Foot tunnel entrance © Richard Croft (cc-by-sa/2.0)

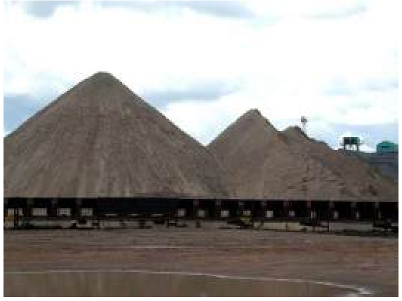

Stockpiles © Helen Wilkinson (cc-by-sa/2.0)

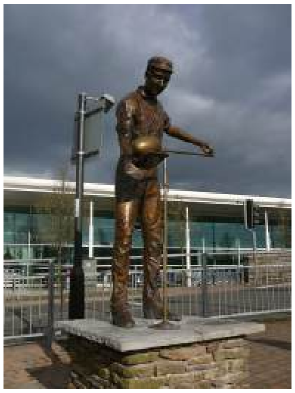

'The Glass Blower' Nailsea © Colin Park (cc-by-sa/2.0)

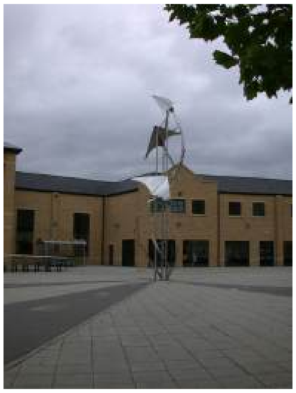

Flight © Keith Edkins (cc-by-sa/2.0)

3.88 Morning

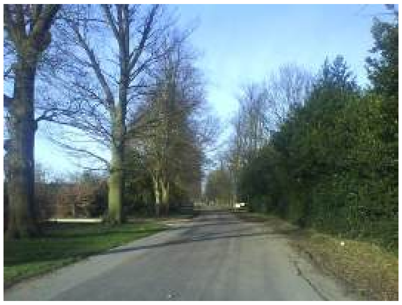

Hatching Green © Gary Fellows (cc-by-sa/2.0)

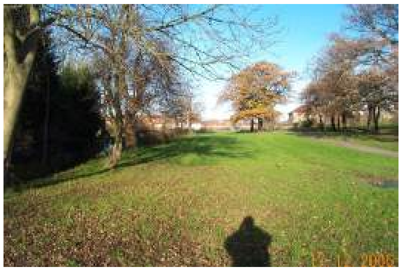

Burnt Oak: Watling Park © Nigel Cox (cc-by-sa/2.0)

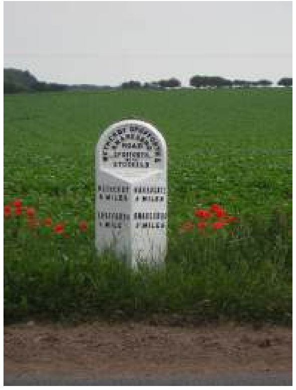

Milestone at Crosper Farm © manonabike (cc-by-sa/2.0)

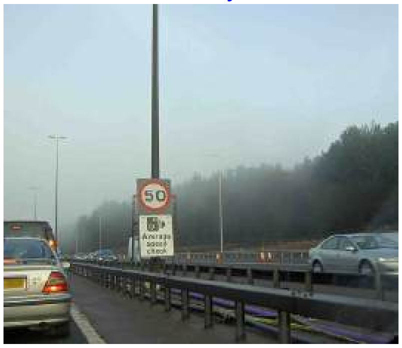

If only! © Steve Fareham (cc-by-sa/2.0)

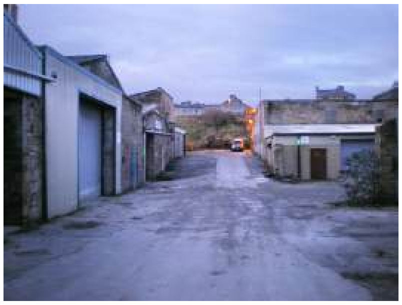

Grove Mill, Padiham © Alexander P Kapp (cc-by-sa/2.0)

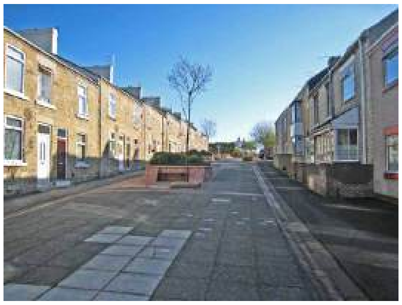

Half Moon Lane, Spennymoor © Oliver Dixon (cc-by-sa/2.0)

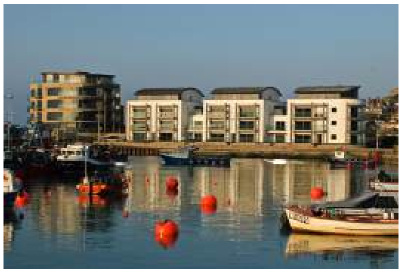

West Bay Development © Tony Watkins (cc-by-sa/2.0)

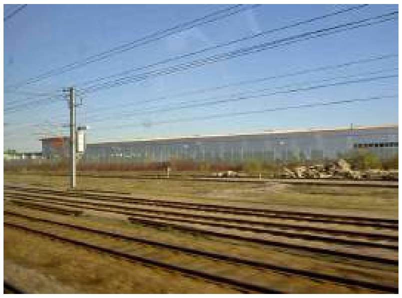

B&Q Peterborough © Steve Fareham (cc-by-sa/2.0)

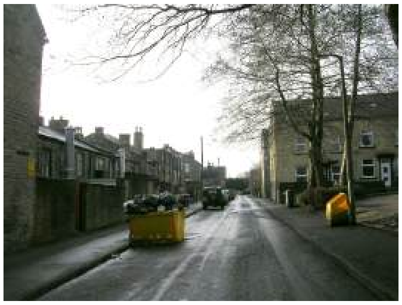

Lombard Street - Queen's Road © Betty Longbottom (cc-by-sa/2.0)

3.89 Motor Vehicle

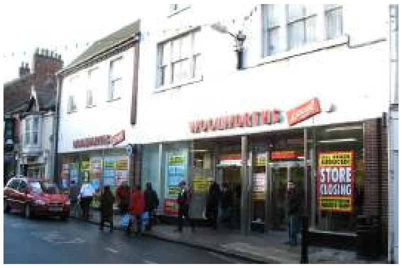

Nearing the End © Gordon Hatton (cc-by-sa/2.0)

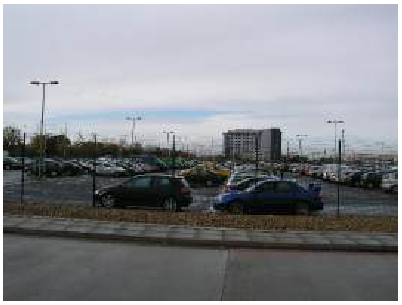

BAA Staff Parking At Heathrow © Jon Clark (cc-by-sa/2.0)

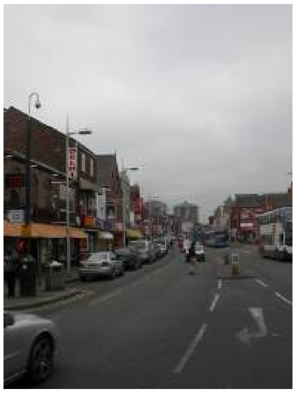

Rusholme, Curry Mile © Mike Faherty (cc-by-sa/2.0)

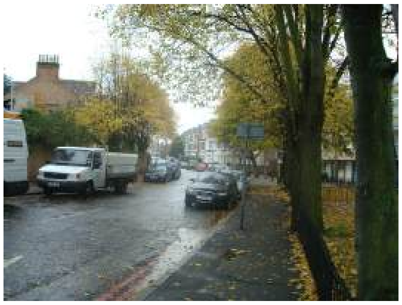

Montrell Road, SW2 © Stacey Harris (cc-by-sa/2.0)

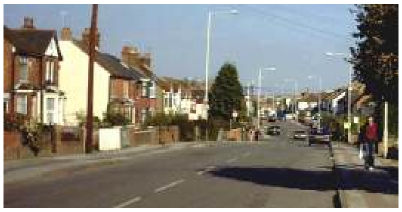

Kingsnorth Road, South Ashford © Adam Colton (cc-by-sa/2.0)

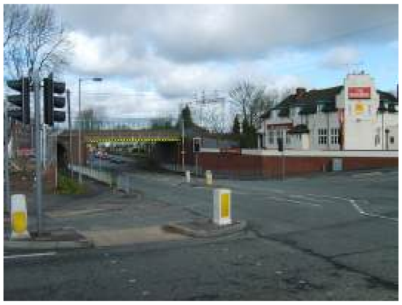

Fordhouse Road Junction © Gordon Griffiths (cc-by-sa/2.0)

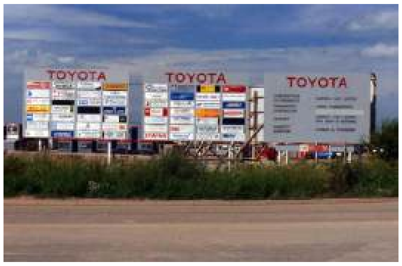

Toyota Car Factory, Burnaston, Derby © mike smith (cc-by-sa/2.0)

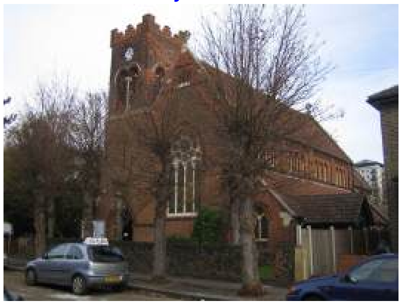

Chadwell Heath: St Chad's Church © Nigel Cox (cc-by-sa/2.0)

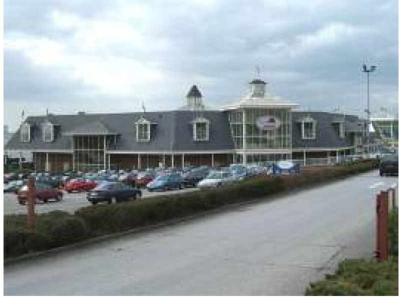

Freeport, Talke © Steve Lewin (cc-by-sa/2.0)

3.90 Mountain

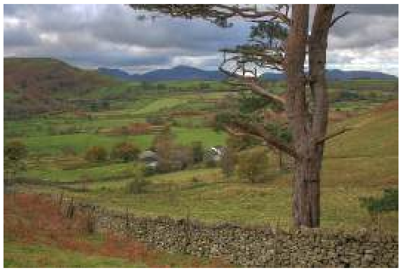

View Down on Sykes © Mick Garratt (cc-by-sa/2.0)

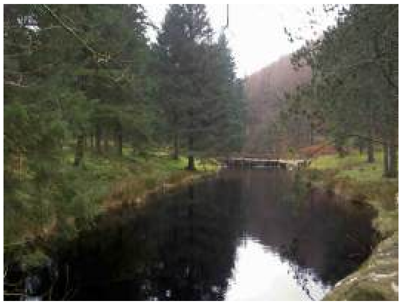

Ditch clough entering Howden reservoir © Steve Fareham (cc-by-sa/2.0)

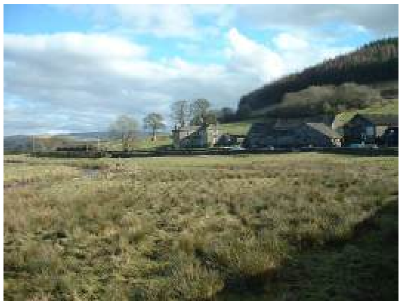

Spital Farm © David Medcalf (cc-by-sa/2.0)

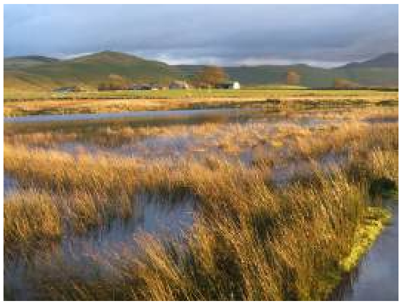

Baggra Yeat Farm © David Brown (cc-by-sa/2.0)

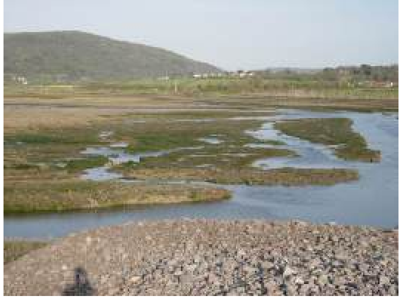

Porlock Saltings © Hugh Venables (cc-by-sa/2.0)

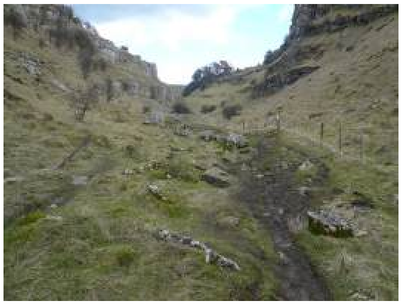

Lathkill Dale Footpath © Alan Heardman (cc-by-sa/2.0)

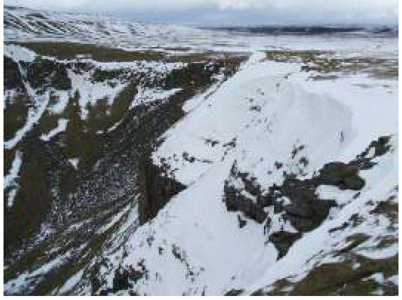

High Cup Nick © David Brown (cc-by-sa/2.0)

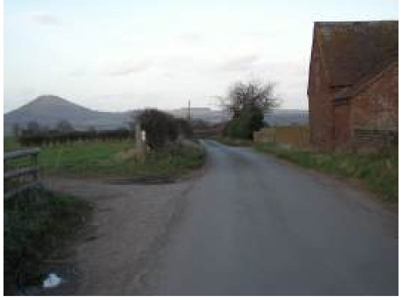

Passing Cressage House. © Row17 (cc-by-sa/2.0)

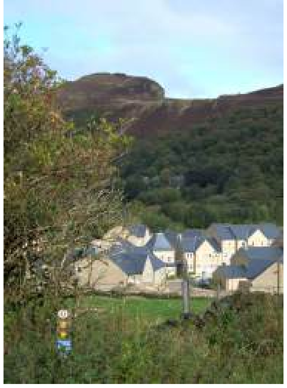

Pennine Bridleway, Carrbrook © michael ely (cc-by-sa/2.0)

3.91 Natural Environment

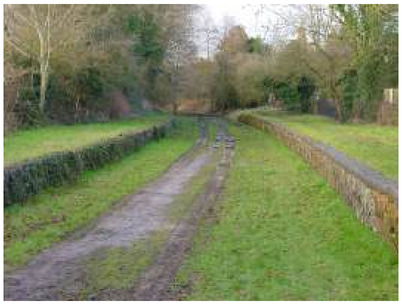

Site of Denstone Railway Station © Linda Bailey (cc-by-sa/2.0)

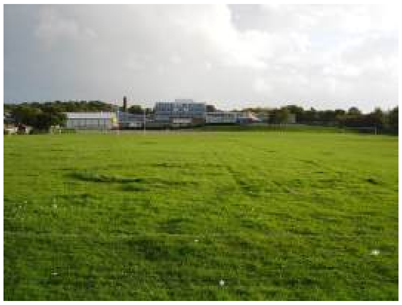

Playing fields © Roger McLachlan (cc-by-sa/2.0)

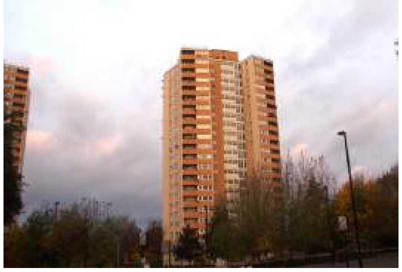

Acton tower blocks, W3 © Phillip Perry (cc-by-sa/2.0)

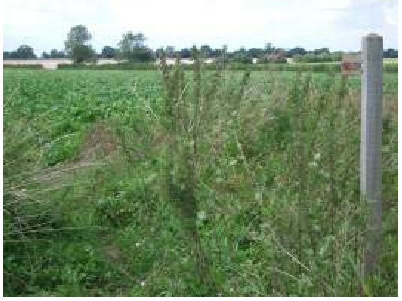

Footpath to Wood Green © Andrew Longton (cc-by-sa/2.0)

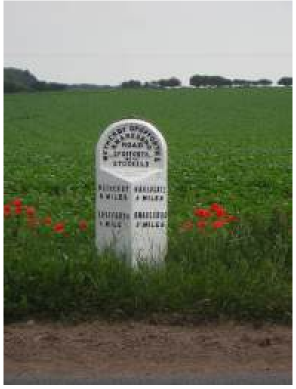

Milestone at Crosper Farm © manonabike (cc-by-sa/2.0)

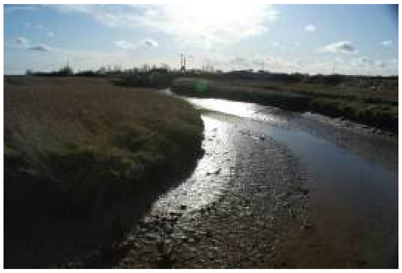

Tidal creek, looking south-west © Oxymoron (cc-by-sa/2.0)

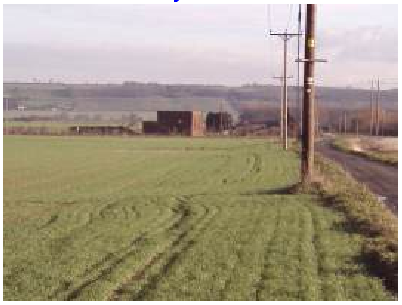

Scotney Farm Lane © fred roberts (cc-by-sa/2.0)

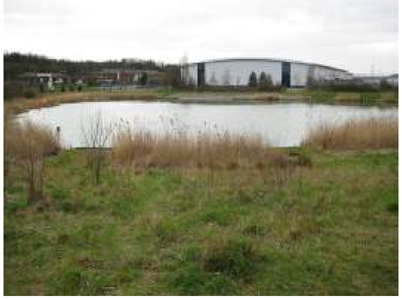

Greenhithe: Crossways Business Park © Nigel Cox (cc-by-sa/2.0)

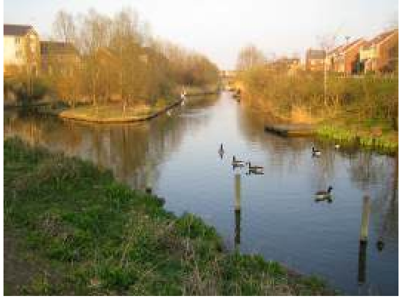

Thamesmead: Gallions Canal © Nigel Cox (cc-by-sa/2.0)

3.92 Natural Landscape

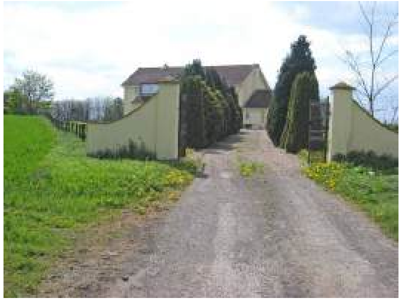

Chilton Grange © Oliver Dixon (cc-by-sa/2.0)

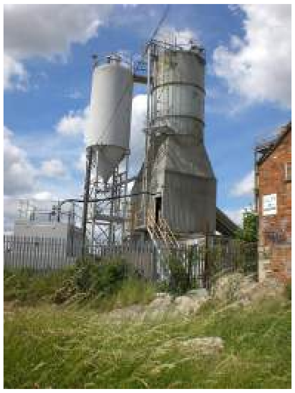

Concrete plant on Soar Island © Evan (cc-by-sa/2.0)

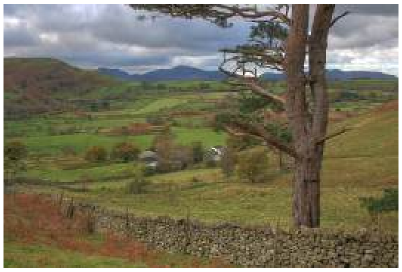

View Down on Sykes © Mick Garratt (cc-by-sa/2.0)

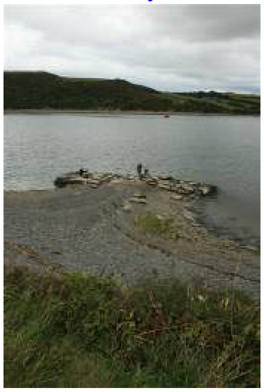

Camel fishermen © Katy Walters (cc-by-sa/2.0)

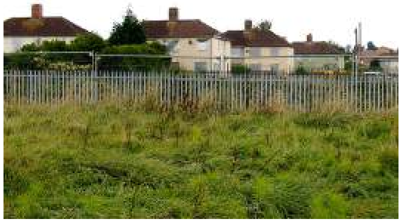

Derelict houses waiting to be demolished © Linda Bailey (cc-by-sa/2.0)

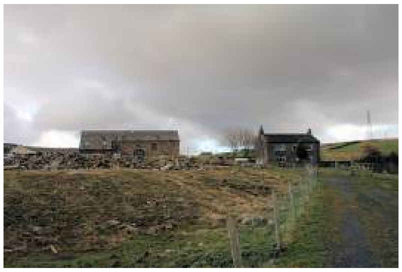

Merrill Head Cliviger © Kevin Rushton (cc-by-sa/2.0)

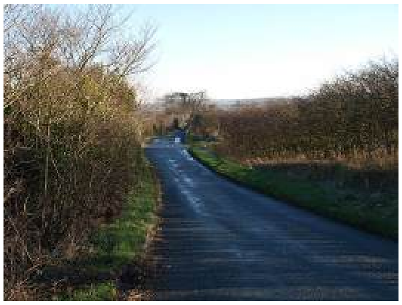

Sneaton Thorpe Lane © Stephen McCulloch (cc-by-sa/2.0)

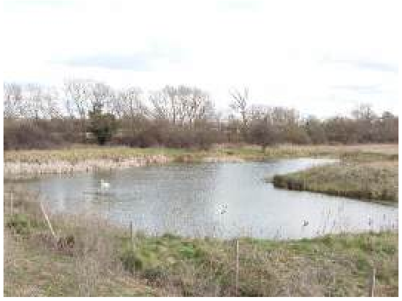

Pond on Stanwell Moor © David Hawgood (cc-by-sa/2.0)

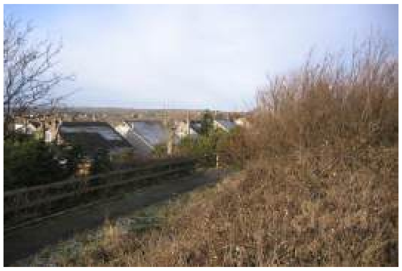

Thornhill. © John Holmes (cc-by-sa/2.0)

3.93 Neighbourhood

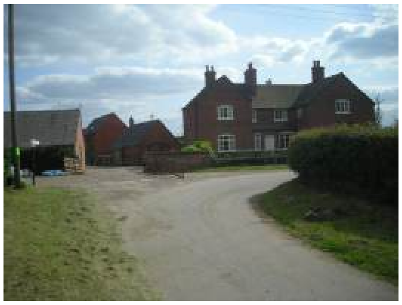

Lane past Isombridge Farm © Row17 (cc-by-sa/2.0)

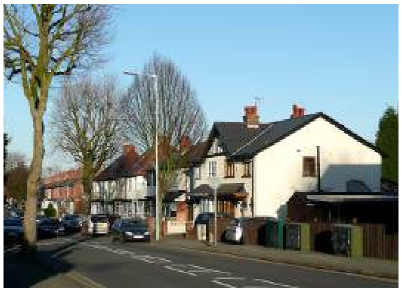

Lea Road, Penn Fields, Wolverhampton © Roger D Kidd (cc-by-sa/2.0)

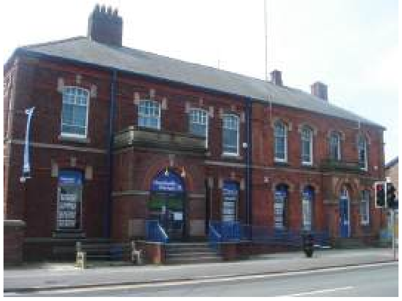

Former Walton-le-Dale Council Offices, Bamber Bridge © Margaret Clough (cc-by-sa/2.0)

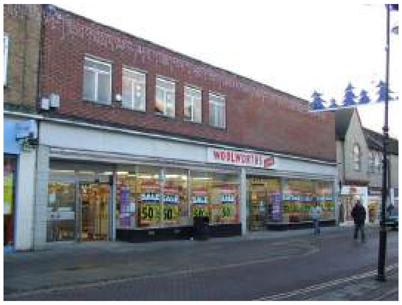

Woolworths Haverhill © Keith Evans (cc-by-sa/2.0)

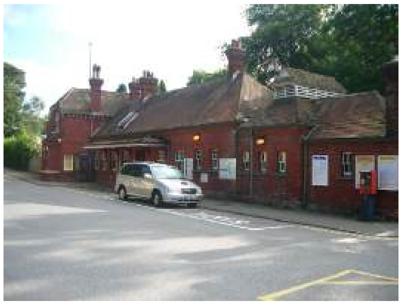

Oxshott railway station © Andrew Longton (cc-by-sa/2.0)

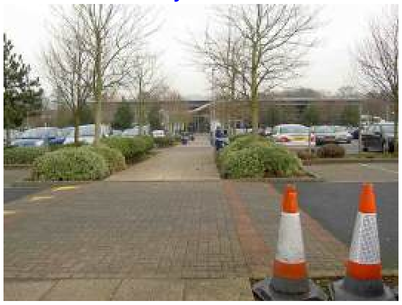

Hopwood Park motorway services © Steve Fareham (cc-by-sa/2.0)

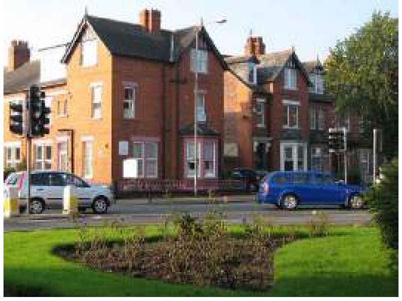

Warwick Road, Carlisle © Lis Burke (cc-by-sa/2.0)

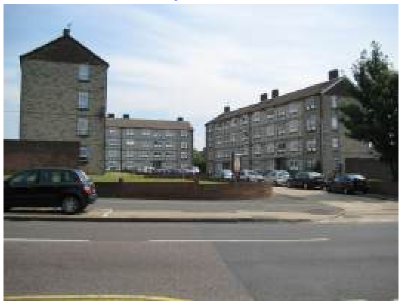

Collier Row: Prospect Place © Nigel Cox (cc-by-sa/2.0)

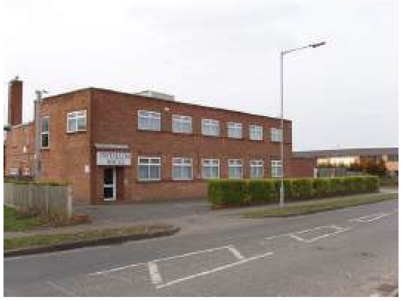

Offices in Manor Way, Borehamwood © David Hawgood (cc-by-sa/2.0)

3.94 Nonbuilding Structure

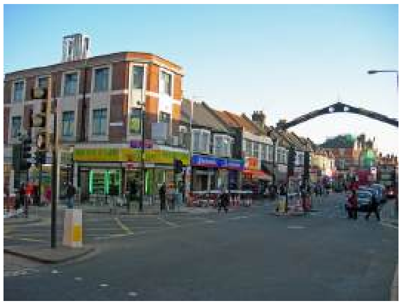

High Street North, E12 (3) © Danny P Robinson (cc-by-sa/2.0)

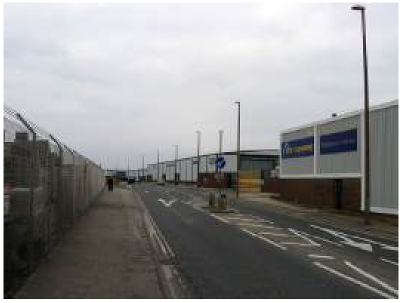

Malthouse Industrial Estate, Brighton Road © Simon Carey (cc-by-sa/2.0)

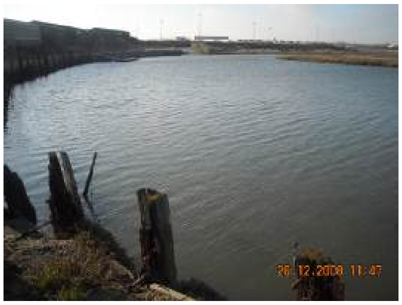

Murston Wharf, Milton Creek © Joe White (cc-by-sa/2.0)

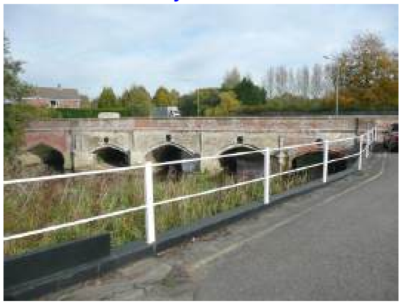

The old bridge, Nethergate, Saxlingham © Humphrey Bolton (cc-by-sa/2.0)

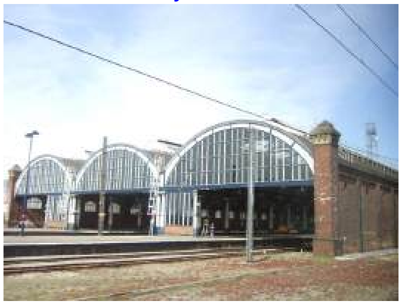

Bank Top Station, Darlington © Stanley Howe (cc-by-sa/2.0)

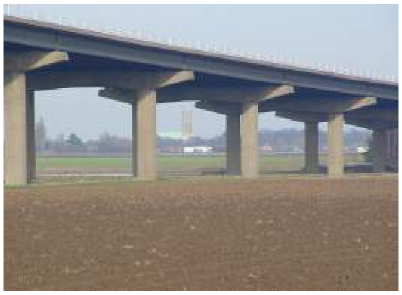

The Ouse Bridge © Peter Church (cc-by-sa/2.0)

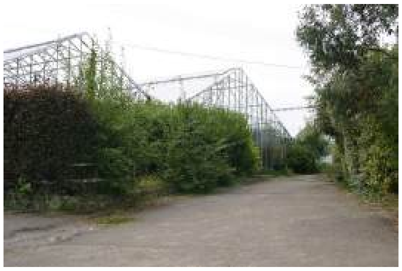

Glenholme nurseries © Shaun Ferguson (cc-by-sa/2.0)

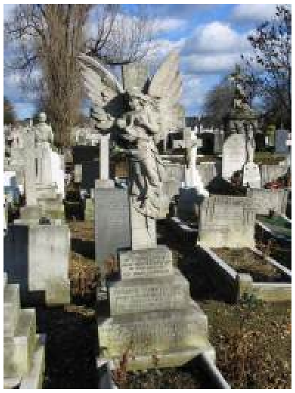

St Patrick's Cemetery, Langthorne Road, Leytonstone, London E11 © John Salmon (cc-by-sa/2.0)

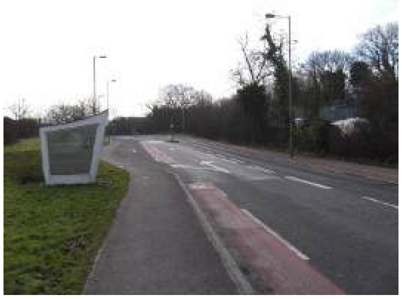

Welcome to Gosport © Barry Shimmon (cc-by-sa/2.0)

3.95 Overhead Power Line

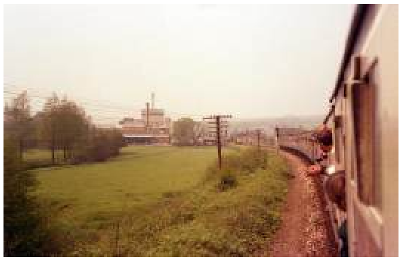

Torbay Express © John Lucas (cc-by-sa/2.0)

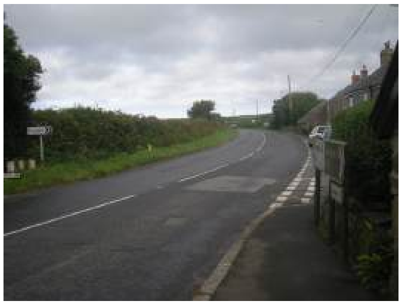

A30, next stop Penzance © Row17 (cc-by-sa/2.0)

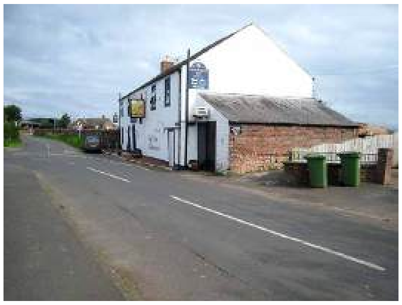

Centurion Inn, Walton © Oliver Dixon (cc-by-sa/2.0)

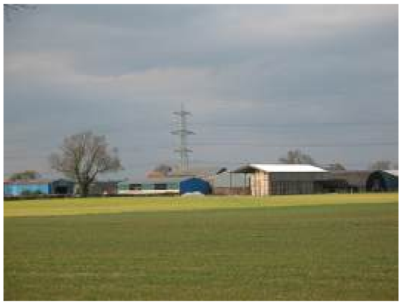

Forest Hall Farm near Alne © Gordon Hatton (cc-by-sa/2.0)

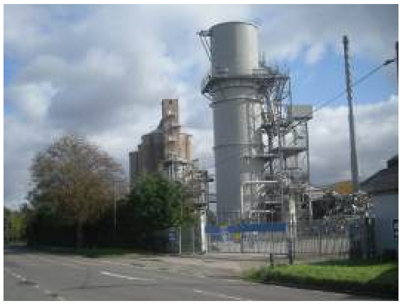

Sugar Beet Factory being demolished © Row17 (cc-by-sa/2.0)

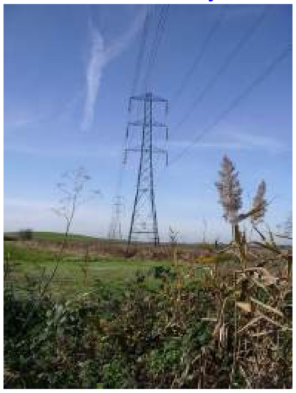

Pylons off Seasalter Lane © pam fray (cc-by-sa/2.0)

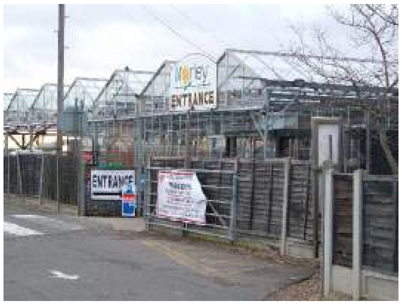

Morley © Julieanne Savage (cc-by-sa/2.0)

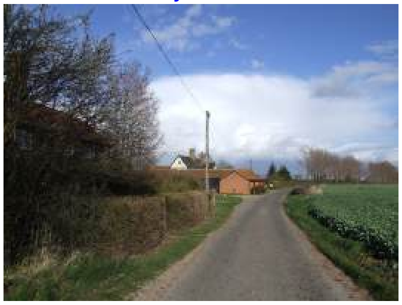

Clintergate © Ian Robertson (cc-by-sa/2.0)

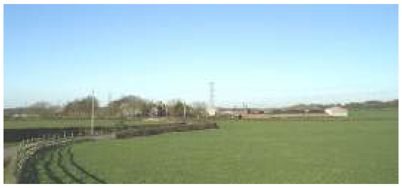

White Carr Farm & Kennels, north of M55 © David Long (cc-by-sa/2.0)

3.96 Overpass

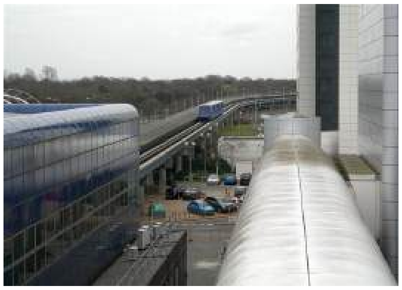

Monorail At Gatwick Airport © Mary and Angus Hogg (cc-by-sa/2.0)

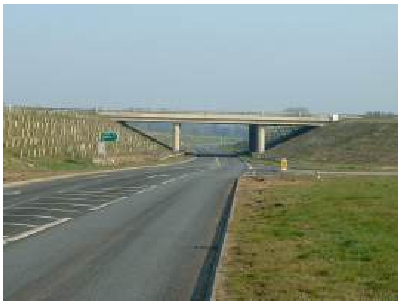

A11 crossing B1111 © Keith Evans (cc-by-sa/2.0)

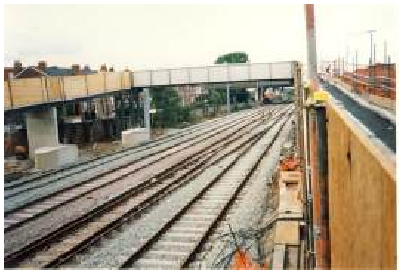

Altrincham approach © Peter Whatley (cc-by-sa/2.0)

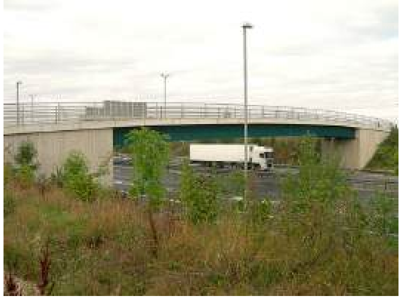

Footbridge over M11 south of M11/A120 interchange © John Smith (cc-by-sa/2.0)

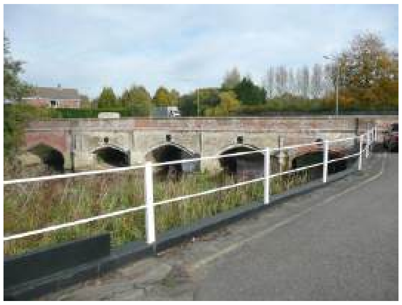

The old bridge, Nethergate, Saxlingham © Humphrey Bolton (cc-by-sa/2.0)

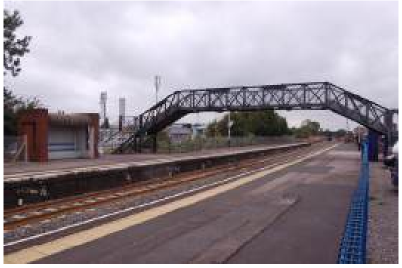

Patchway railway station © Roger Davies (cc-by-sa/2.0)

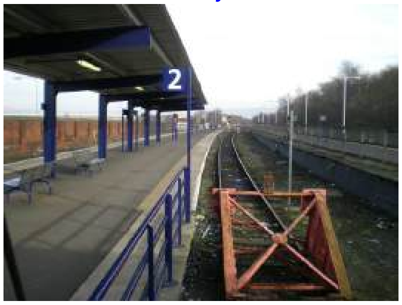

End of the line 2 © R lee (cc-by-sa/2.0)

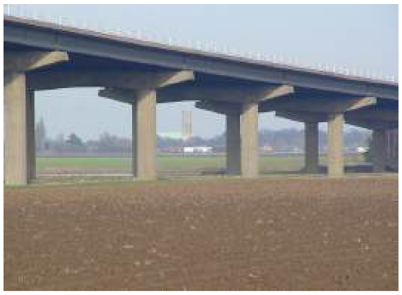

The Ouse Bridge © Peter Church (cc-by-sa/2.0)

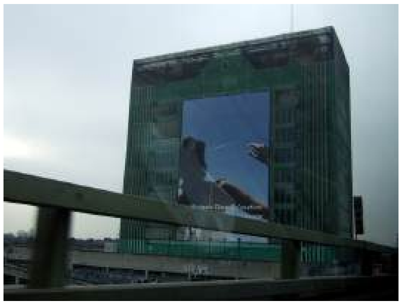

2009 and the Alfa Laval building is still empty © Natasha Ceridwen de Chroustchoff (cc-by-sa/2.0)

3.97 Parking

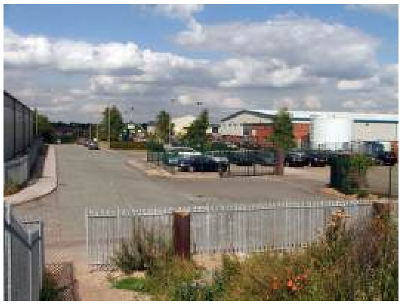

Industrial Estate, Ratcher Way © James Hill (cc-by-sa/2.0)

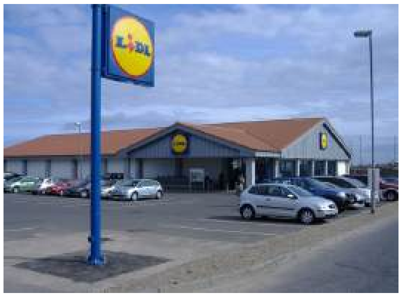

Lidl supermarket, Ryhope © Roger Cornfoot (cc-by-sa/2.0)

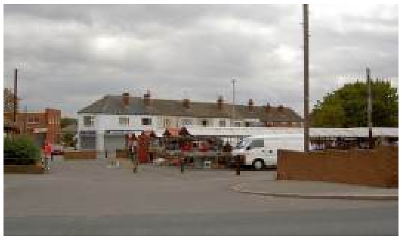

Outdoor market. © Steve Fareham (cc-by-sa/2.0)

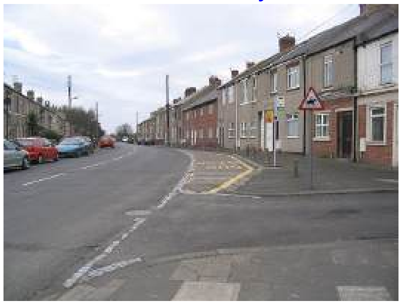

Red Row © Walter Baxter (cc-by-sa/2.0)

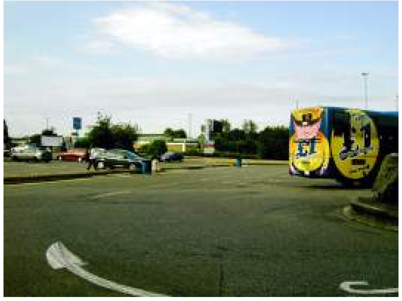

Car & coach parking at Watford Gap M1 Services © Slbs (cc-by-sa/2.0)

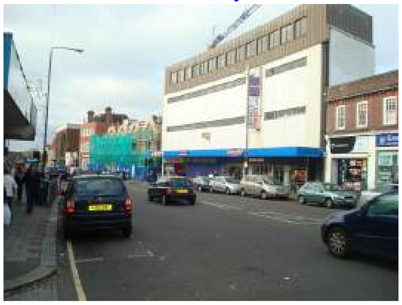

Eltham High Street, London SE9 © Stacey Harris (cc-by-sa/2.0)

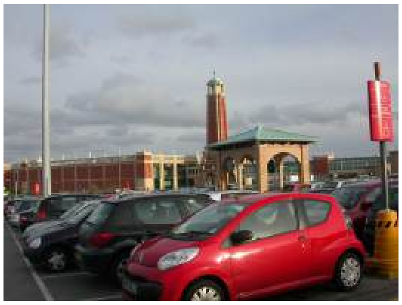

Trafford Centre, car park © Mike Faherty (cc-by-sa/2.0)

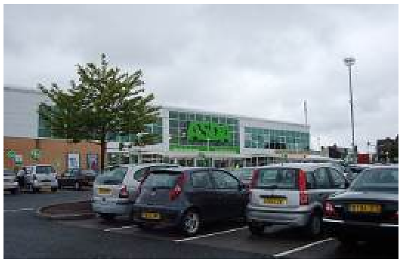

Asda, Rooley Lane, Bowling, Bradford © michael ely (cc-by-sa/2.0)

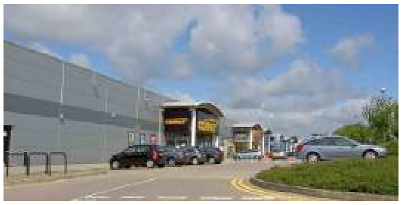

Central retail park © Steve Fareham (cc-by-sa/2.0)

3.98 Parking Lot

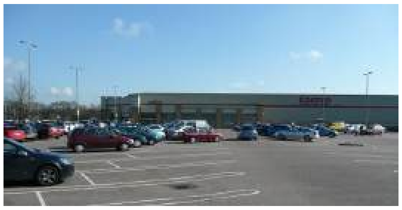

Costco superstore, Watford © Jonathan Billinger (cc-by-sa/2.0)

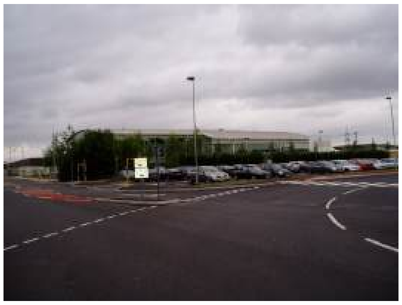

Capenhurst Technology Park © Eirian Evans (cc-by-sa/2.0)

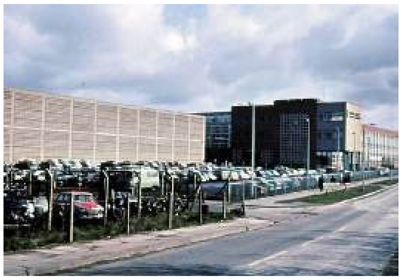

Pressed Steel 1967 © Gordon Hatton (cc-by-sa/2.0)

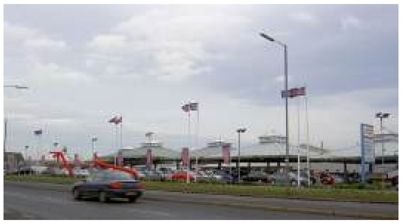

Perry's Vauxhall dealership on Wheatley Hall Road © Steve Fareham (cc-by-sa/2.0)

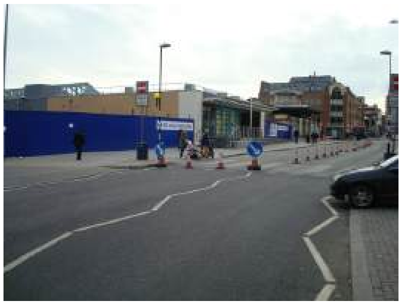

Woolwich Arsenal DLR Station © Stacey Harris (cc-by-sa/2.0)

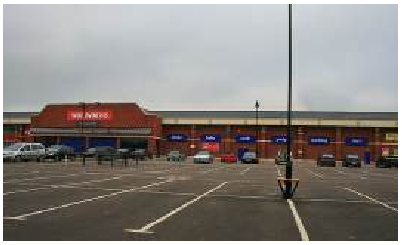

Newark Woolworths © David Lally (cc-by-sa/2.0)

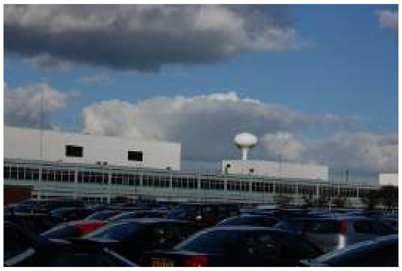

The Onion Water Tower, Basildon © Trevor Harris (cc-by-sa/2.0)

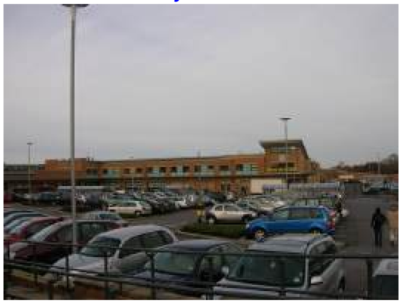

Gloucester, Abbeydale: Morrisons © Alby (cc-by-sa/2.0)

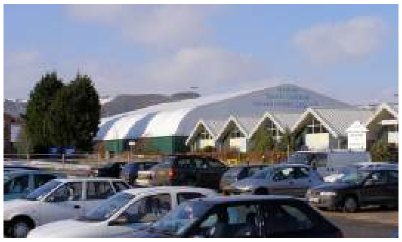

Harlow Sports Centre, Sussex Downs College, Eastbourne © Kevin Gordon (cc-by-sa/2.0)

3.99 Passenger Car

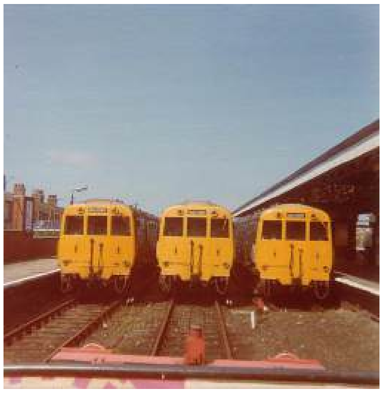

Awaiting departure © Peter Whatley (cc-by-sa/2.0)

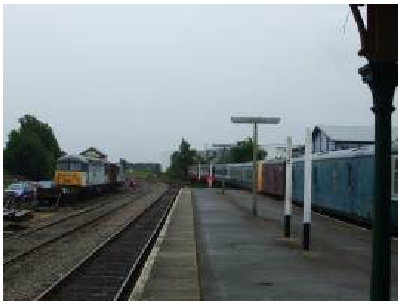

Dereham station © Ashley Dace (cc-by-sa/2.0)

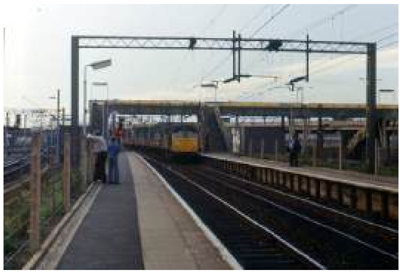

Class 25 enters Bescot Station on a short Freight © Tim Marshall (cc-by-sa/2.0)

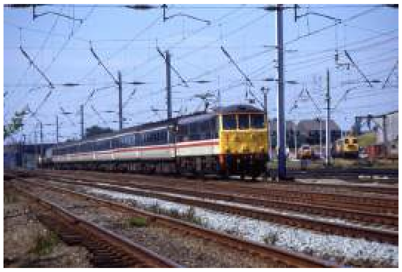

Train passing Springs Branch © David Ashcroft (cc-by-sa/2.0)

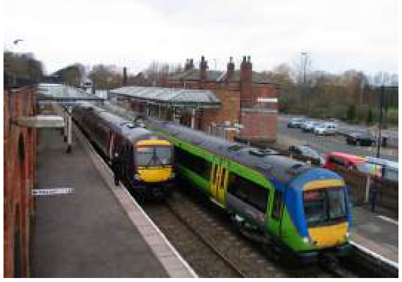

Trains to Stansted and Birmingham © Andrew Tatlow (cc-by-sa/2.0)

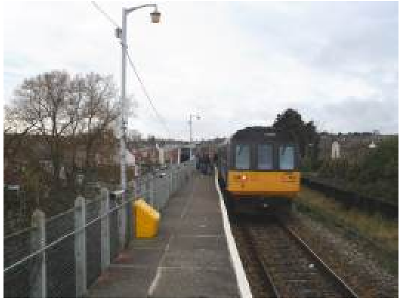

Polsloe Bridge Halt © Roger Cornfoot (cc-by-sa/2.0)

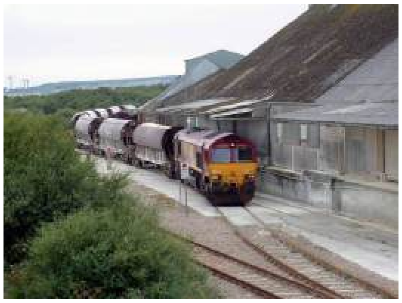

Loading Clay for Cliffe Vale © roger geach (cc-by-sa/2.0)

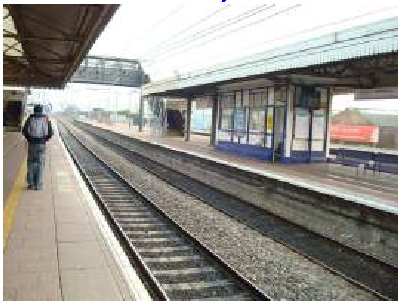

Hayes and Harlington Railway Station © Stacey Harris (cc-by-sa/2.0)

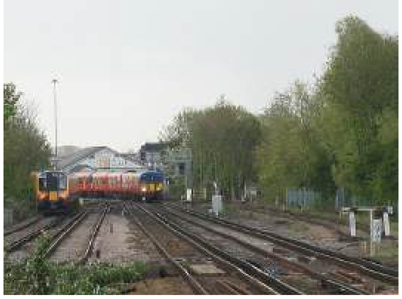

Railway between Wandsworth and Clapham Junction © Stephen Craven (cc-by-sa/2.0)

3.100 Pasture

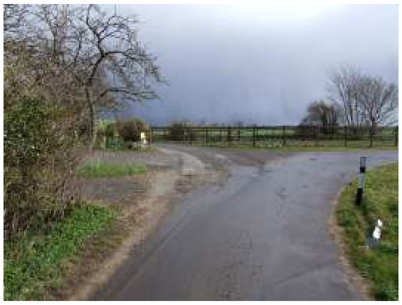

Paddock and Stables Entrance, Kemp's Corner © Ian Robertson (cc-by-sa/2.0)

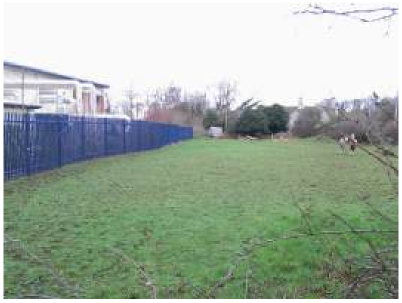

Boundary fence © David Luther Thomas (cc-by-sa/2.0)

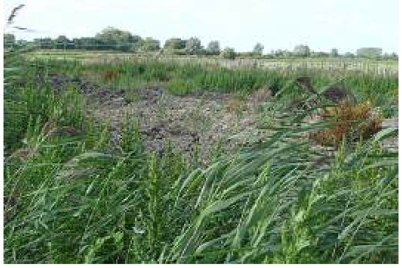

Here be dredgings © Graham Horn (cc-by-sa/2.0)

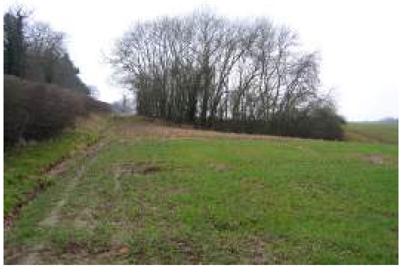

Wooded former marlpit, Raynham, Norfolk © Rodney Burton (cc-by-sa/2.0)

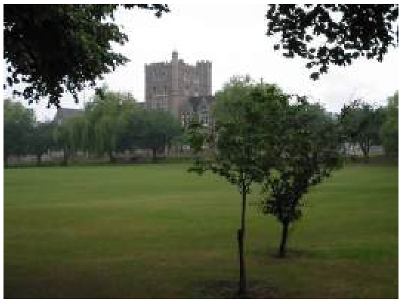

Rotherham Grammar School © Chris Field (cc-by-sa/2.0)

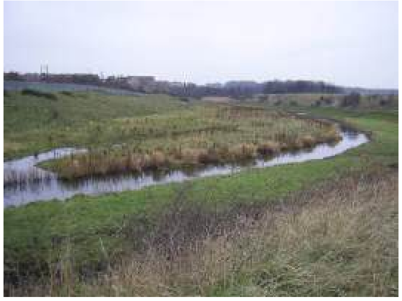

Lyneburn © george hurrell (cc-by-sa/2.0)

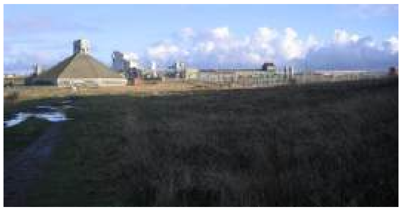

The Phospate Store. © John Holmes (cc-by-sa/2.0)

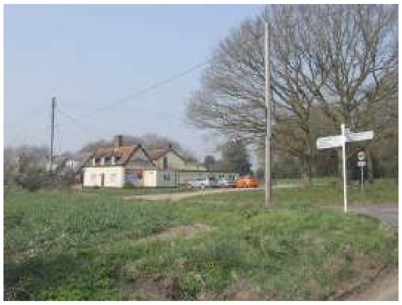

The Garden House © Ian Robertson (cc-by-sa/2.0)

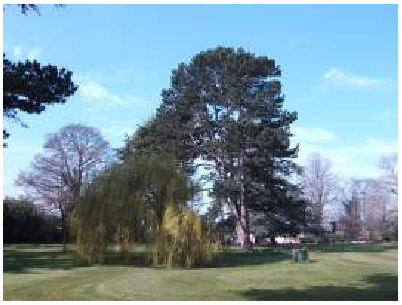

Trees in the grounds of Windsor House © Jonathan Billinger (cc-by-sa/2.0)

3.101 Pedestrian

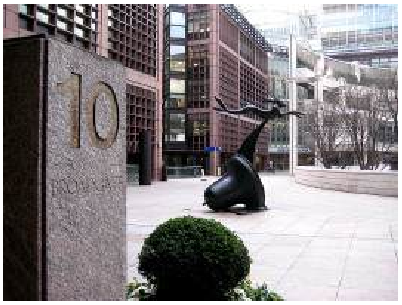

Hare and Bell sculpture . . . © Zorba the Geek (cc-by-sa/2.0)

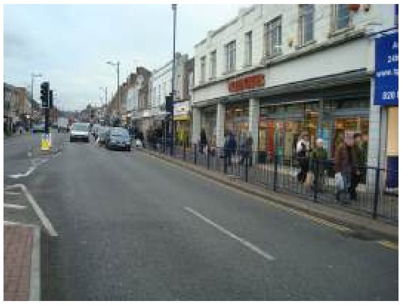

Bellegrove Road, Welling © Stacey Harris (cc-by-sa/2.0)

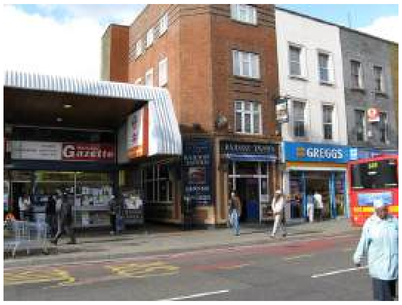

The 'Railway Tavern', Dalston © Dr Neil Clifton (cc-by-sa/2.0)

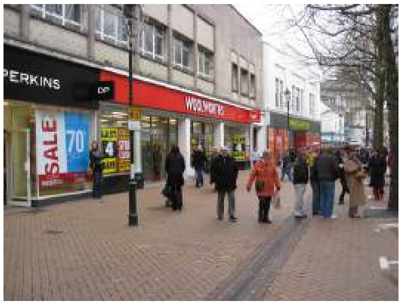

Mansfield - West Gate © Alan Heardman (cc-by-sa/2.0)

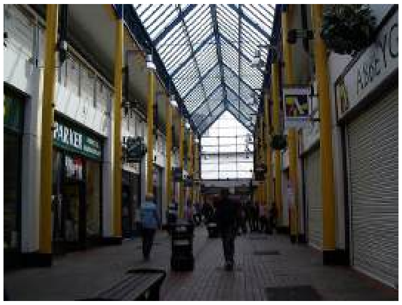

The former Heron Way now known as Abbeygate © kevin roe (cc-by-sa/2.0)

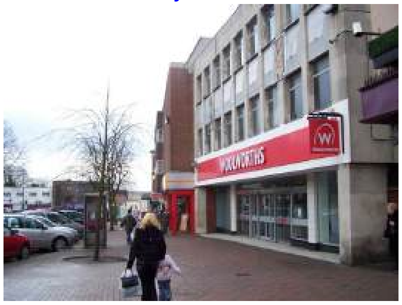

Woolworths, Cannock © Geoff Pick (cc-by-sa/2.0)

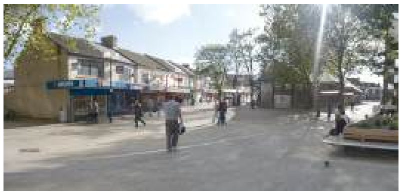

Swindon : Market Street © Lewis Clarke (cc-by-sa/2.0)

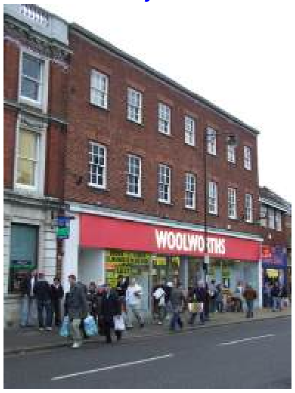

Woolworths Dereham © Keith Evans (cc-by-sa/2.0)

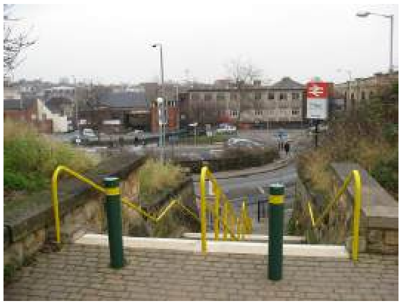

Mansfield - Approaching Station © Alan Heardman (cc-by-sa/2.0)

3.102 Personal Luxury Car

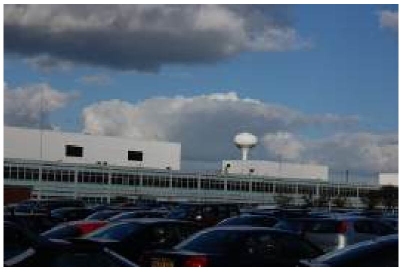

The Onion Water Tower, Basildon © Trevor Harris (cc-by-sa/2.0)

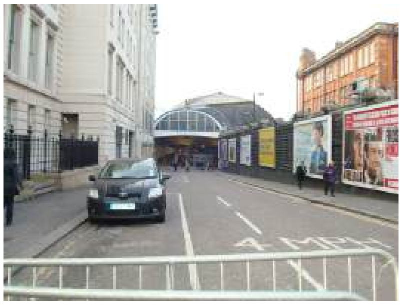

Paddington Station © Stacey Harris (cc-by-sa/2.0)

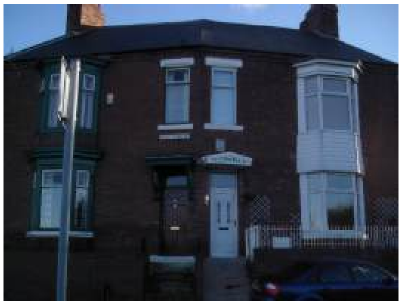

Dean Terrace © MSX (cc-by-sa/2.0)

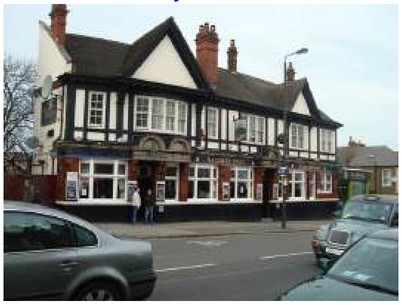

Railway Bell Public House, Tooting © Stacey Harris (cc-by-sa/2.0)

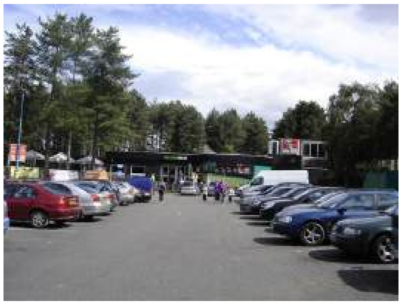

Fleet Services, M3 London bound © Julian P Guffogg (cc-by-sa/2.0)

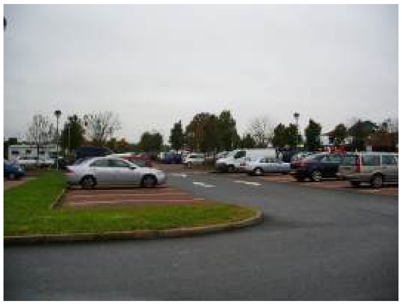

Car park at Warwick Services on the M40 © Alexander P Kapp (cc-by-sa/2.0)

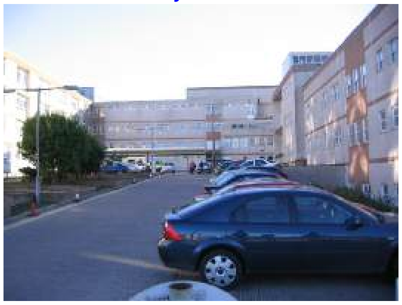

West Cumberland Hospital © John Holmes (cc-by-sa/2.0)

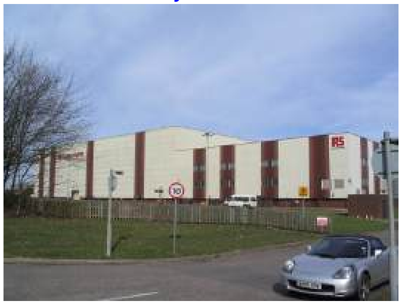

Corby industry - new © Tim Heaton (cc-by-sa/2.0)

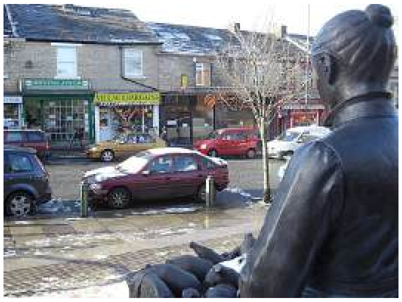

Stamford Street, Top Mossley © michael ely (cc-by-sa/2.0)

3.103 Place of Worship

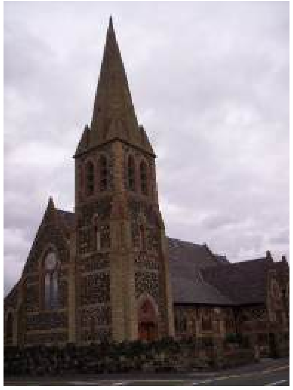

St Peter's Church, Parr, St Helens © S Parish (cc-by-sa/2.0)

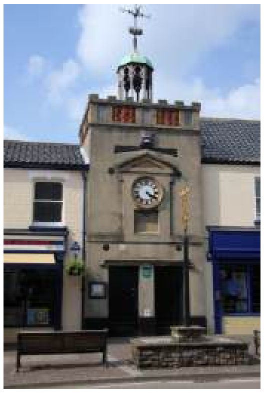

Watton Clock Tower © Bob Jones (cc-by-sa/2.0)

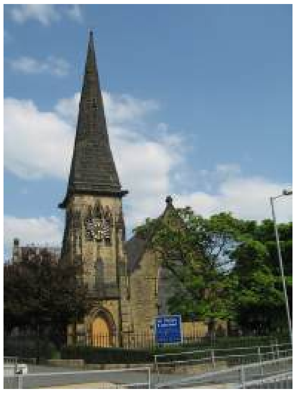

St Philip's, Litherland © Sue Adair (cc-by-sa/2.0)

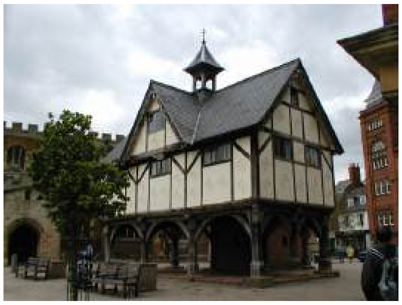

The old Market Place © Row17 (cc-by-sa/2.0)

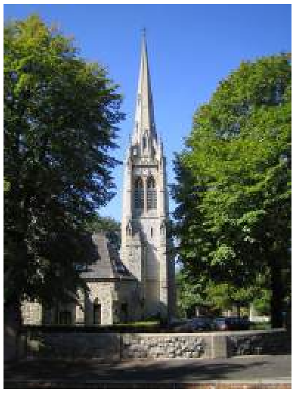

Ealing: St Stephen's Court, The Avenue, W13 © Nigel Cox (cc-by-sa/2.0)

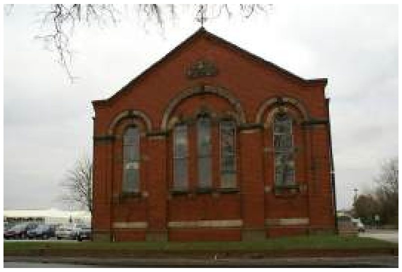

Former Primitive Methodist Chapel © David Long (cc-by-sa/2.0)

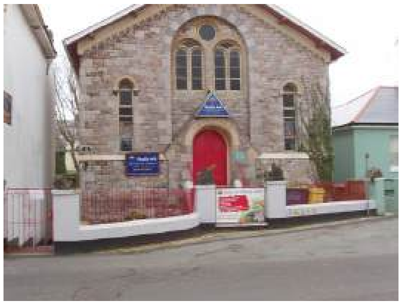

Childcare Centre in Babbacombe © Jennifer Vaughan (cc-by-sa/2.0)

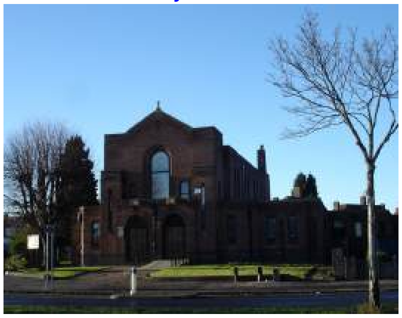

Aspley Methodist Church © Oxymoron (cc-by-sa/2.0)

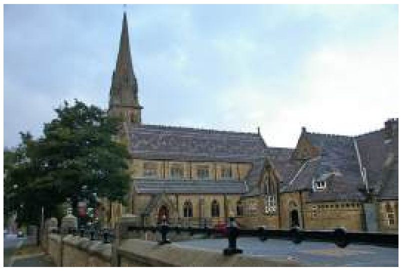

Parish Church of St Mary, Balderstone © Alexander P Kapp (cc-by-sa/2.0)

3.104 Plain

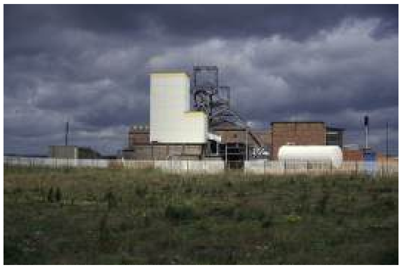

Ellington Colliery © Chris Allen (cc-by-sa/2.0)

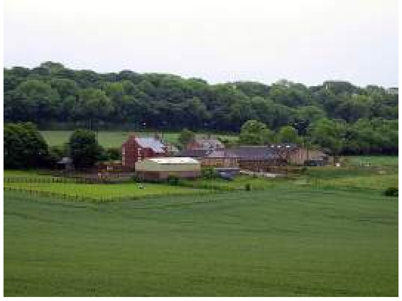

Over the Hill Farm © Roger Smith (cc-by-sa/2.0)

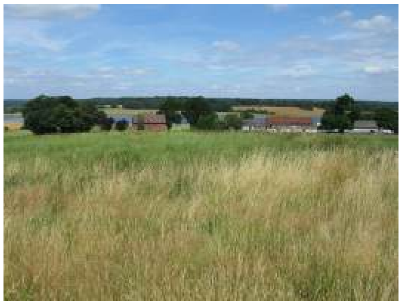

Dairy House Farm, Blithfield, Staffordshire. © Alan Slater (cc-by-sa/2.0)

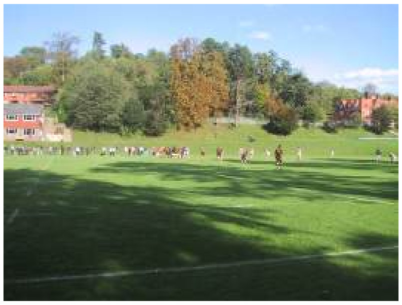

Rugby at Caterham School © David Cumberland (cc-by-sa/2.0)

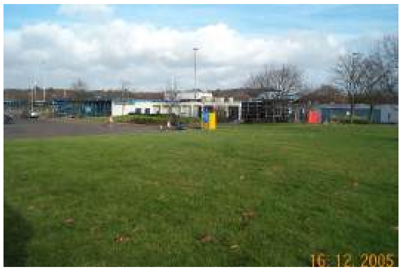

M1 Motorway: London Gateway Services © Nigel Cox (cc-by-sa/2.0)

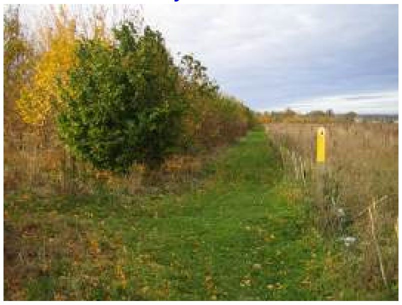

Biggleswade: Footpath near Top Field Farm © Nigel Cox (cc-by-sa/2.0)

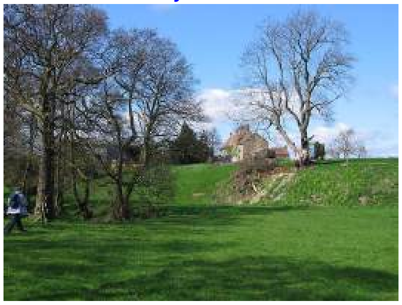

Streatlam Grove Farm © Roger Smith (cc-by-sa/2.0)

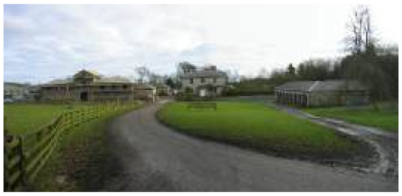

Park Farm, Hulne Park, Alnwick © Les Hull (cc-by-sa/2.0)

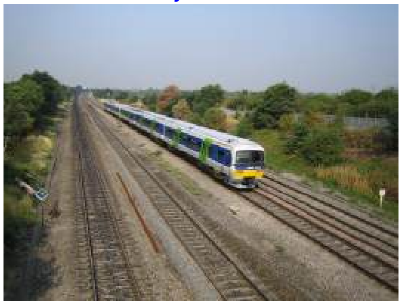

Iver: Railway lines © Nigel Cox (cc-by-sa/2.0)

3.105 Plant

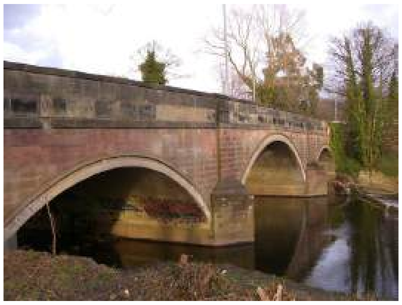

Bridge at Otterspool © Peter Fuller (cc-by-sa/2.0)

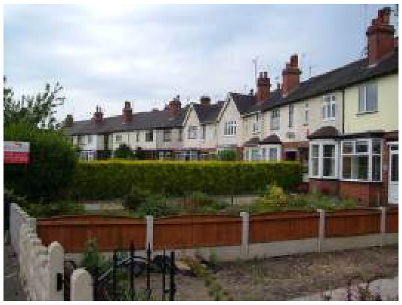

Crescent, Dimsdale Parade East, Wolstanton © Derek Harper (cc-by-sa/2.0)

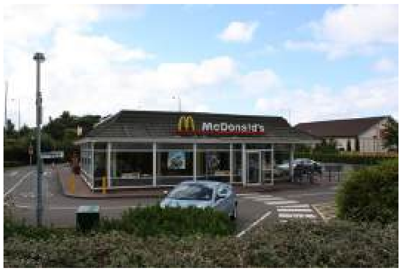

McDonald's Restaurant, Bermuda Park © Richard Kay (cc-by-sa/2.0)

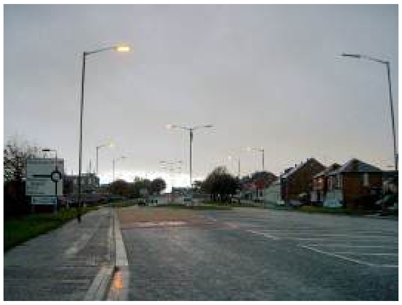

Glebe Road © george hurrell (cc-by-sa/2.0)

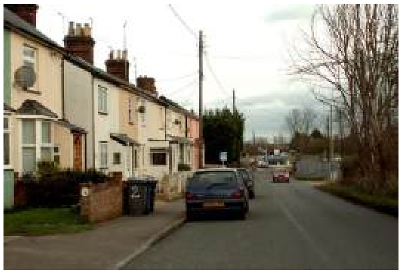

The end of the road from Withersfield © Robert Edwards (cc-by-sa/2.0)

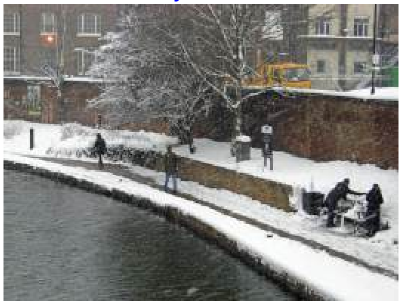

Regent's Canal, King's Cross © Stephen McKay (cc-by-sa/2.0)

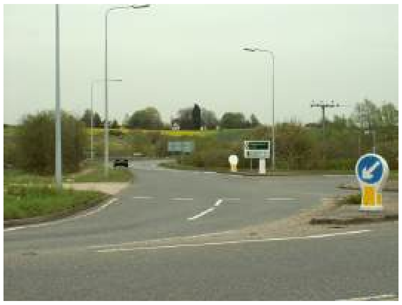

Part of the A130, Great Waltham by-pass © Robert Edwards (cc-by-sa/2.0)

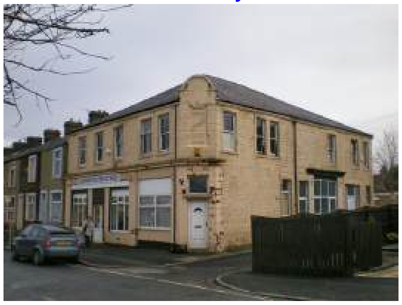

Former Nelson Co-operative Society shop © Alexander P Kapp (cc-by-sa/2.0)

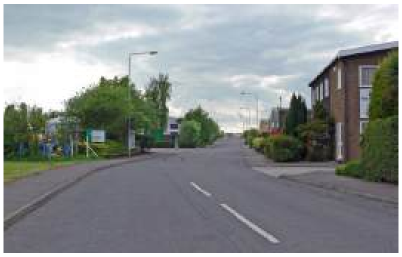

Ivanhoe Industrial Estate © Mat Fascione (cc-by-sa/2.0)

3.106 Plant Community

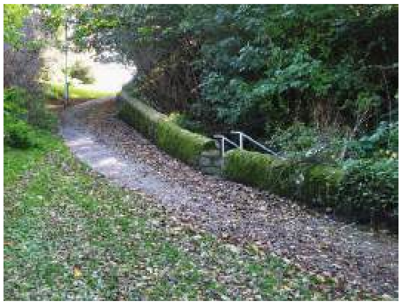

Path past the Kep Well © Mike Quinn (cc-by-sa/2.0)

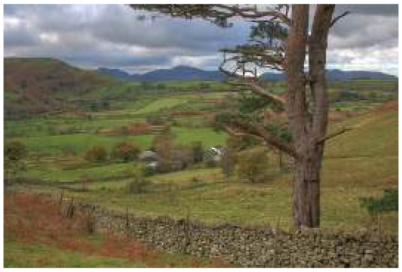

View Down on Sykes © Mick Garratt (cc-by-sa/2.0)

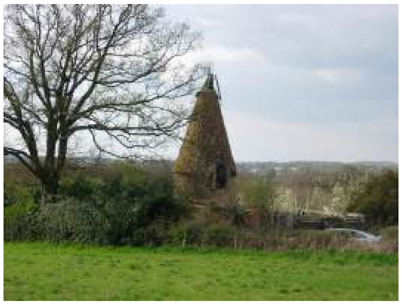

An Oast House in Disrepair © Jean Barrow (cc-by-sa/2.0)

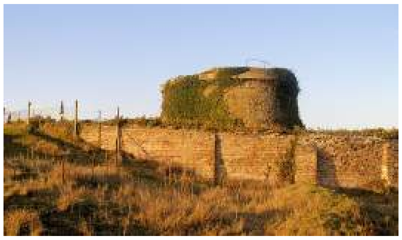

The Enchantress Tower, Martello Tower 28, Rye Harbour © Kevin Gordon (cc-by-sa/2.0)

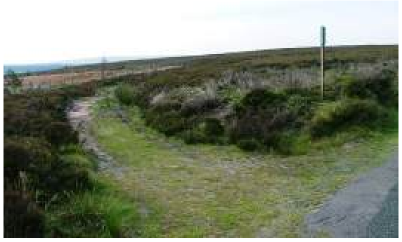

Public Bridleway to High Gill Beck © Mick Garratt (cc-by-sa/2.0)

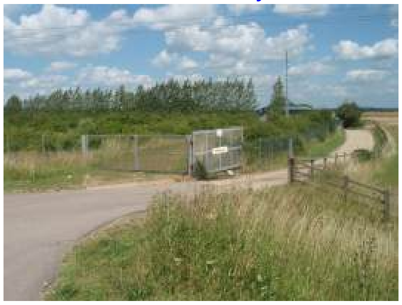

Limekiln Lane © Michael Trolove (cc-by-sa/2.0)

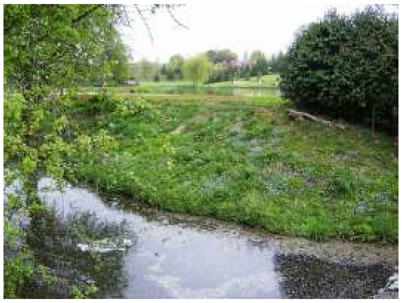

Fish pond at Leigh Lodge © Graham Horn (cc-by-sa/2.0)

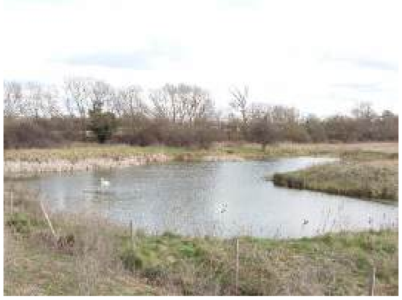

Pond on Stanwell Moor © David Hawgood (cc-by-sa/2.0)

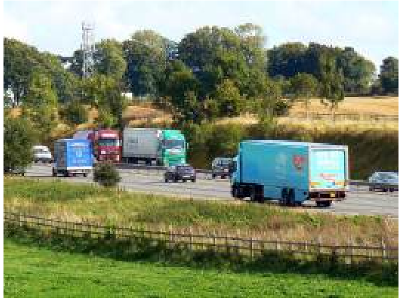

M4 near Peaks Downs, Wiltshire © Brian Robert Marshall (cc-by-sa/2.0)

3.107 Pole

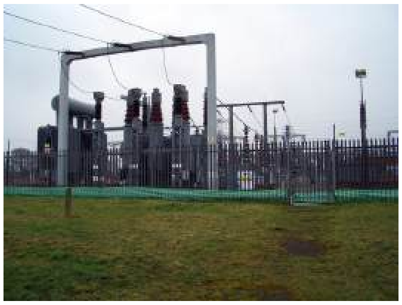

Electric grid Station © Keith Wright (cc-by-sa/2.0)

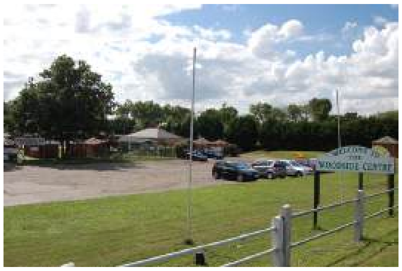

Woodside Centre, A127 (Westbound), Rayleigh © Trevor Harris (cc-by-sa/2.0)

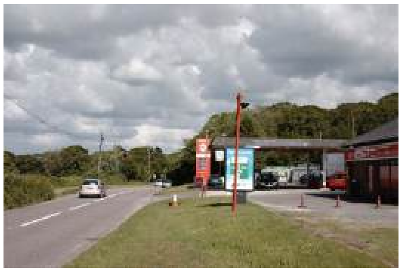

Fishleigh Rock Garage © David Brinicombe (cc-by-sa/2.0)

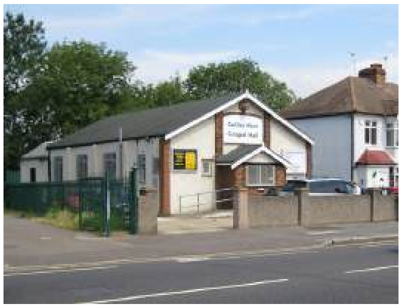

Collier Row Gospel Hall © Nigel Cox (cc-by-sa/2.0)

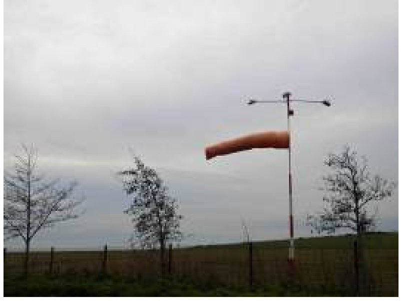

Windsock on the perimeter of Sheffield City Airport © Steve Fareham (cc-by-sa/2.0)

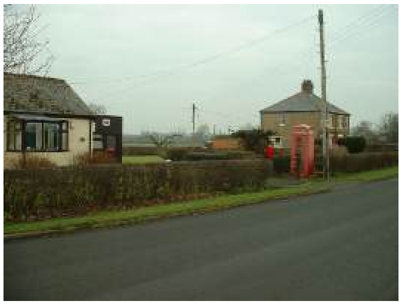

Rawcliffe Post Office © David Medcalf (cc-by-sa/2.0)

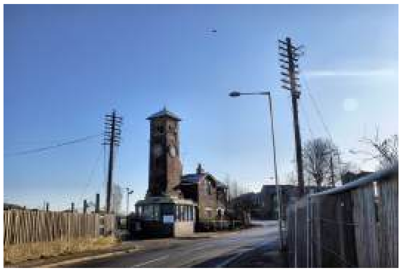

Ashford : Railway Works entrance and clocktower © Chris Morley (cc-by-sa/2.0)

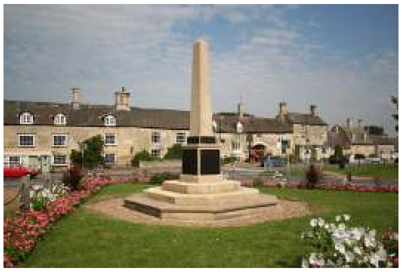

Weldon War Memorial © Richard Croft (cc-by-sa/2.0)

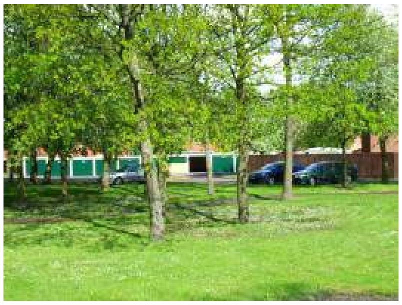

Garage block through the trees © Oliver Dixon (cc-by-sa/2.0)

3.108 Pollution

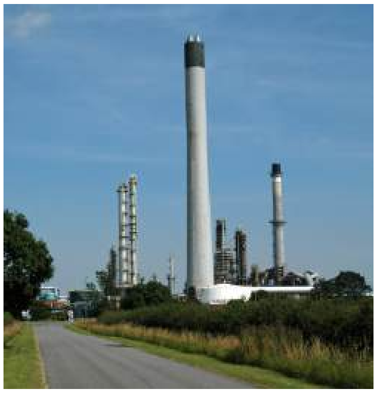
Chimney at North Killingholme Refinery © David Wright (cc-by-sa/2.0)

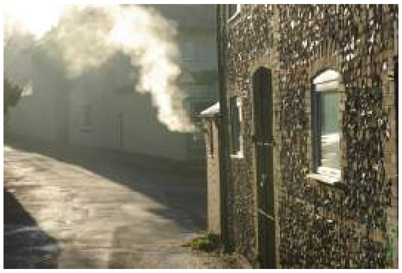
Nether Row, Thetford © Stephen McKay (cc-by-sa/2.0)

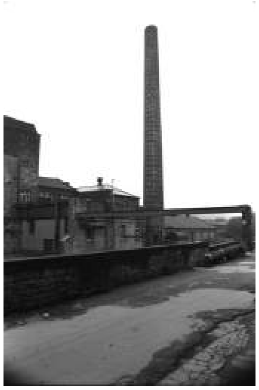
Chimney, Milnsbridge © Chris Allen (cc-by-sa/2.0)

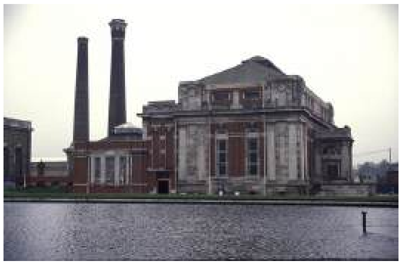
Kempton Pumping Station © Chris Allen (cc-by-sa/2.0)

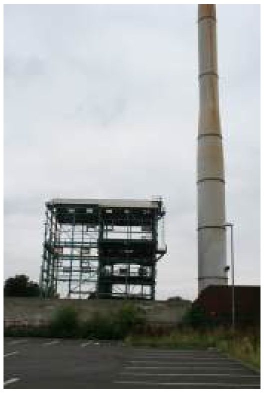
Demolition of a Controversial Factory © David Lally (cc-by-sa/2.0)

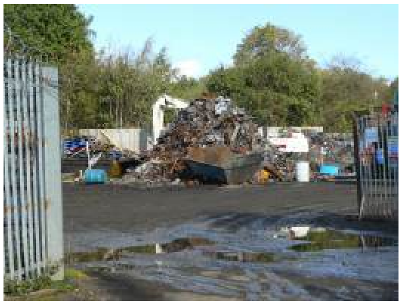
Scrap yard, Oak Lane, Kingswinford © Jonathan Billinger (cc-by-sa/2.0)

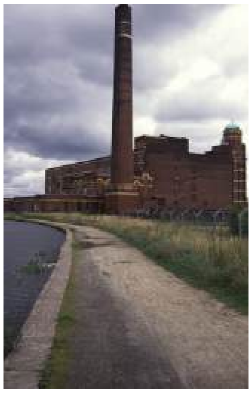
Butts Mill, Leigh © Chris Allen (cc-by-sa/2.0)

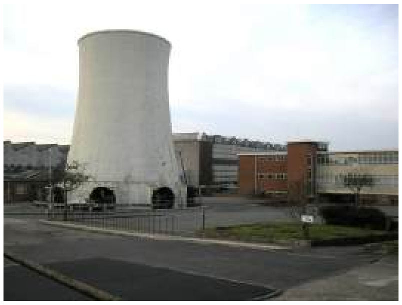
Rugby Alstom Works © Ian Rob (cc-by-sa/2.0)

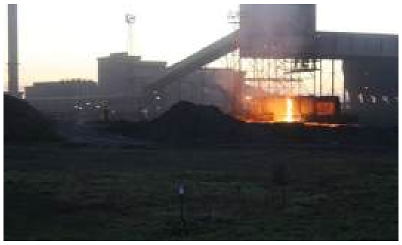
Emptying the Slag © Mick Garratt (cc-by-sa/2.0)

3.109 Power Station

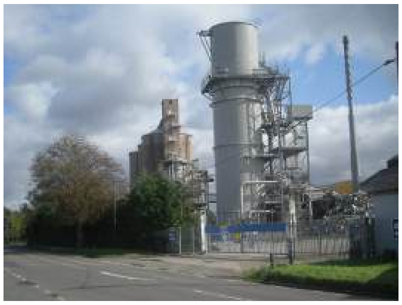

Sugar Beet Factory being demolished © Row17 (cc-by-sa/2.0)

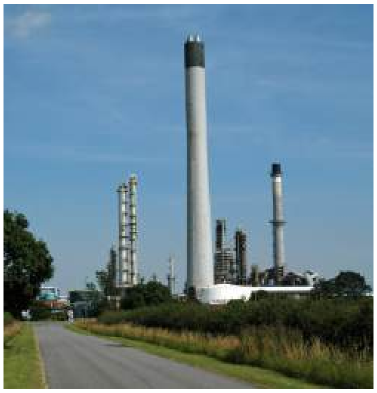

Chimney at North Killingholme Refinery © David Wright (cc-by-sa/2.0)

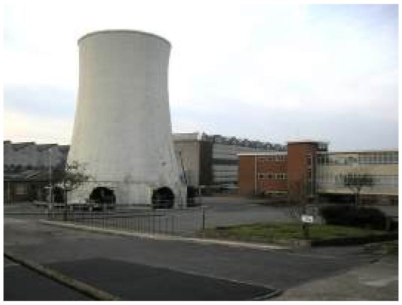

Rugby Alstom Works © Ian Rob (cc-by-sa/2.0)

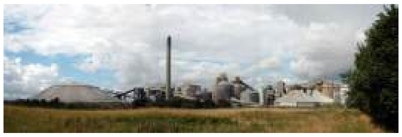

Cemex Plant, Ferriby Sluice © David Wright (cc-by-sa/2.0)

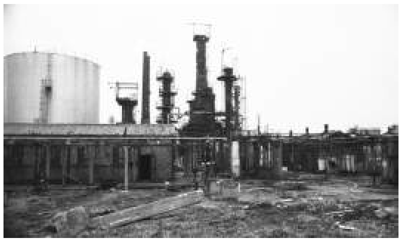

Burmah-Castrol Refinery, Stanlow © Chris Allen (cc-by-sa/2.0)

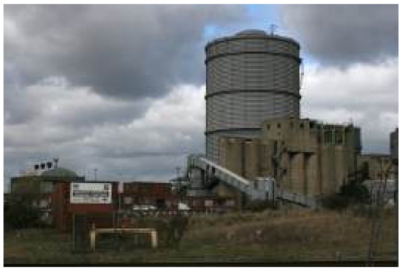

Gas Holder, South Bank Coke Works © Mick Garratt (cc-by-sa/2.0)

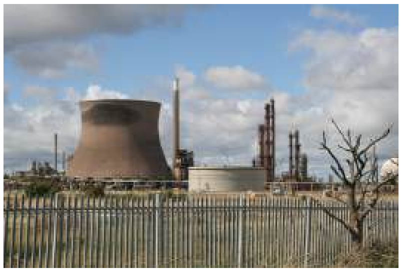

Wilton International © Alan Murray-Rust (cc-by-sa/2.0)

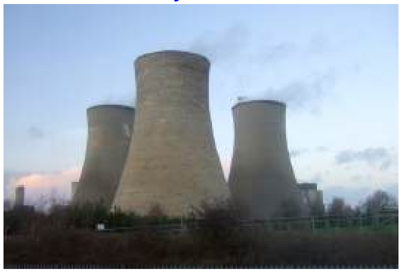

Cooling towers at dusk © Natasha Ceridwen de Chroustchoff (cc-by-sa/2.0)

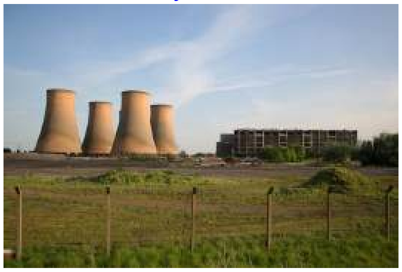

High Marnham partially demolished © Richard Croft (cc-by-sa/2.0)

3.110 Prairie

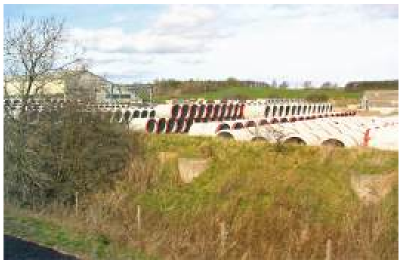

Pipes for all © Alastair Seagroatt (cc-by-sa/2.0)

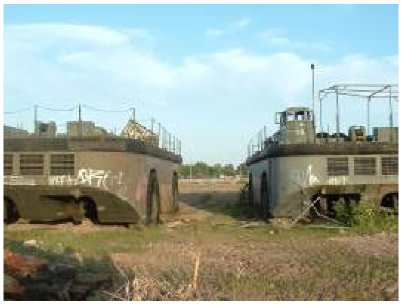

Amphibious vehicles at the Pound's scrap yard © Deanna Earley (cc-by-sa/2.0)

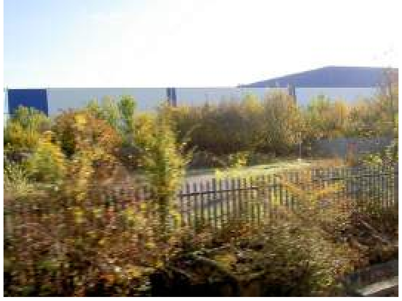

Ikea warehouse Peterborough © Steve Fareham (cc-by-sa/2.0)

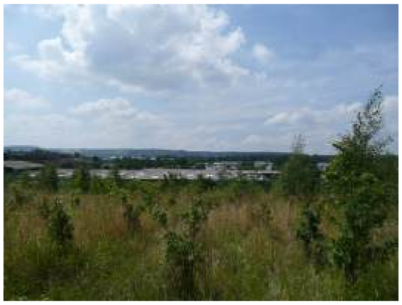

Wentworth Industrial Park © Wendy North (cc-by-sa/2.0)

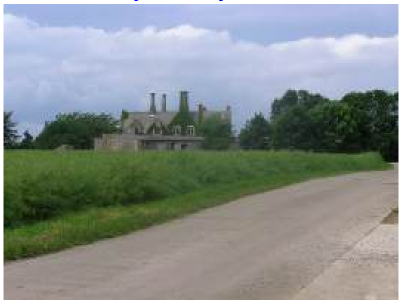

Park House : Stanwick St.John © Hugh Mortimer (cc-by-sa/2.0)

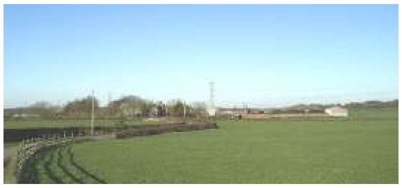

White Carr Farm & Kennels, north of M55 © David Long (cc-by-sa/2.0)

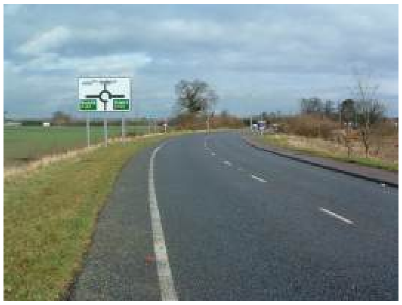

Roundabout Ahead © Keith Evans (cc-by-sa/2.0)

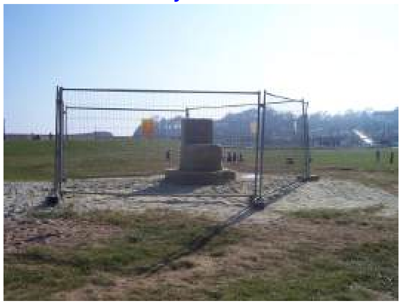

Dawlish Warren : Sand Sculpture © Lewis Clarke (cc-by-sa/2.0)

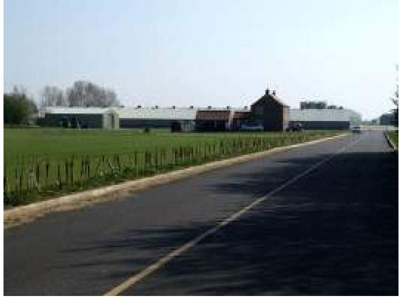

Farm on East Kirkby Aerodrome © Dave Hitchborne (cc-by-sa/2.0)

3.111 Property

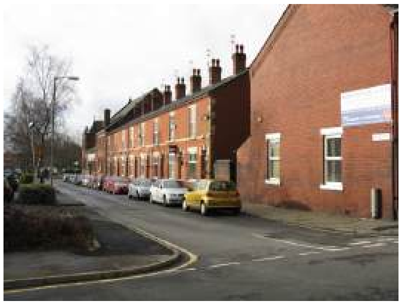
Crown Street © Peter Whatley (cc-by-sa/2.0)

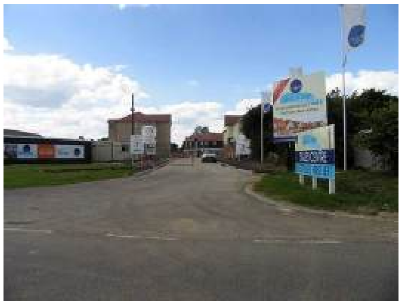
New houses for sale © John Salmon (cc-by-sa/2.0)

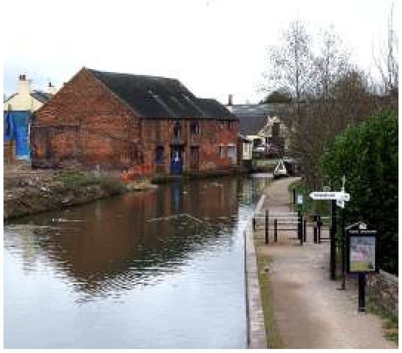
Birmingham & Fazeley Canal, Fazeley Junction © Rob Farrow (cc-by-sa/2.0)

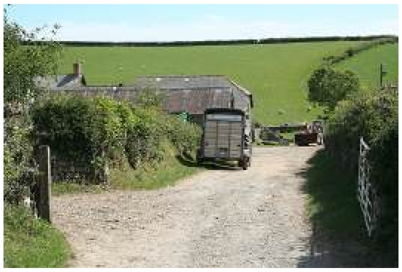
Bishops Tawton: entrance to Shilstone Farm © Martin Bodman (cc-by-sa/2.0)

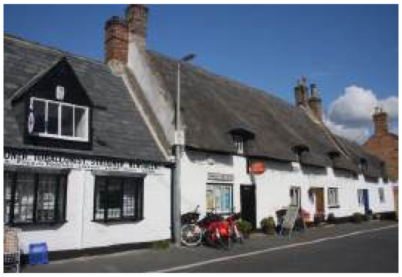
Swavesey post office © Bob Jones (cc-by-sa/2.0)

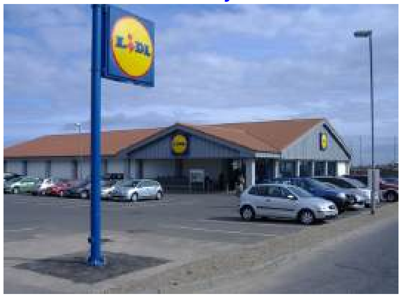
Lidl supermarket, Ryhope © Roger Cornfoot (cc-by-sa/2.0)

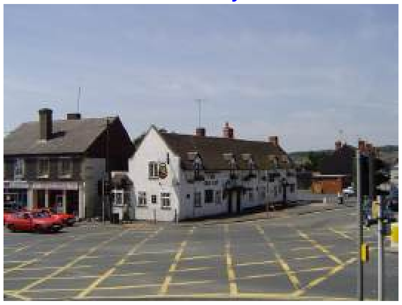
Old Cat © James Skirving (cc-by-sa/2.0)

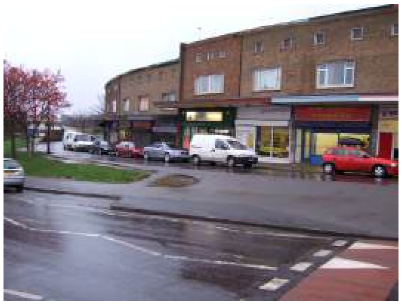
Shops at Hackenthorpe. © John Poyser (cc-by-sa/2.0)

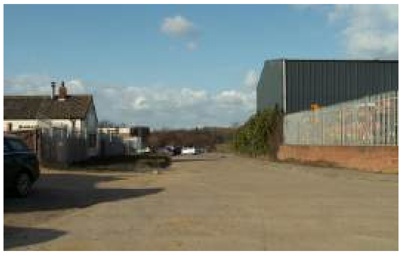
Part of the industrial estate on Ongar Road © Robert Edwards (cc-by-sa/2.0)

3.112 Public Space

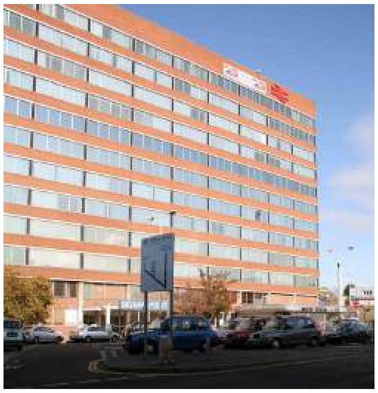

Offices and Swindon station © roger geach (cc-by-sa/2.0)

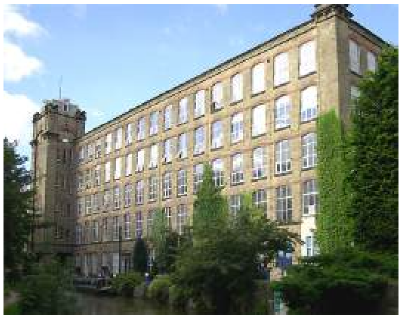

Clarence Mill, Bollington, Cheshire © Roger D Kidd (cc-by-sa/2.0)

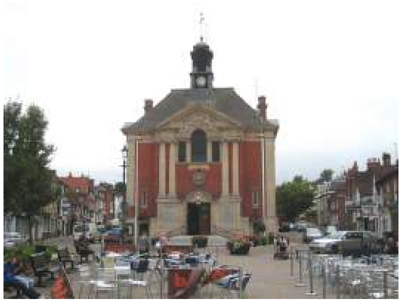

Henley Town Hall © Rod Allday (cc-by-sa/2.0)

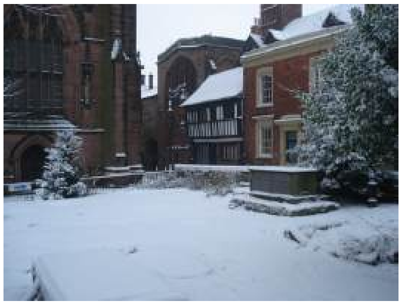

Bayley Lane from St Michael's churchyard © A J Paxton (cc-by-sa/2.0)

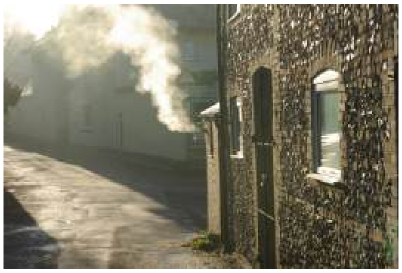

Nether Row, Thetford © Stephen McKay (cc-by-sa/2.0)

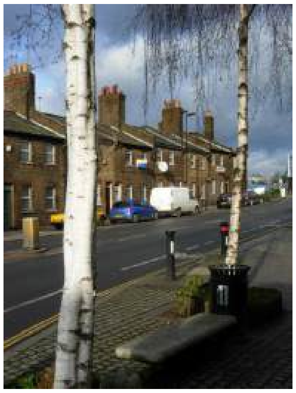

Old Oak Lane, Harlesden © Stephen McKay (cc-by-sa/2.0)

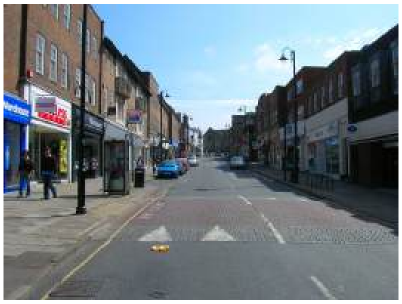

London Road © Simon Carey (cc-by-sa/2.0)

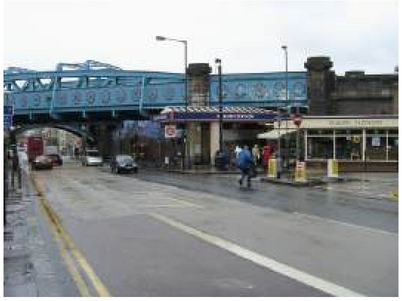

Kilburn Underground Station © Shaun Ferguson (cc-by-sa/2.0)

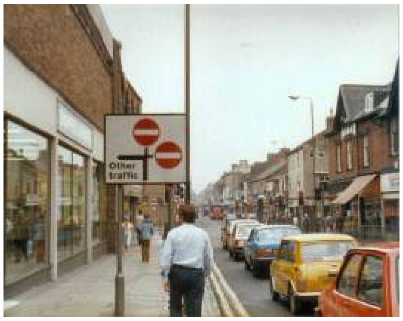

Other - than what? © Keith Edkins (cc-by-sa/2.0)

3.113 Public Transport

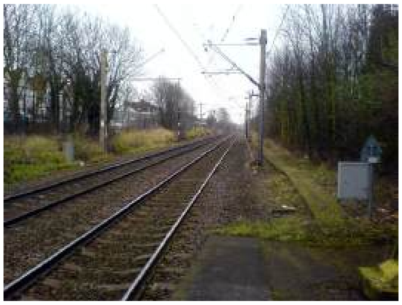

Southend-on-Sea: From Southend East Station looking east © Trevor Durritt (cc-by-sa/2.0)

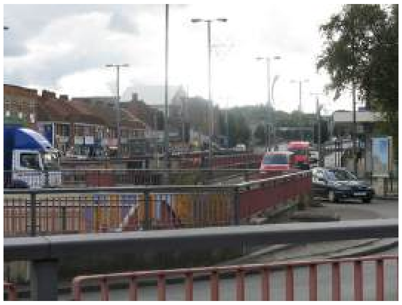

Perry Barr interchange © Peter Whatley (cc-by-sa/2.0)

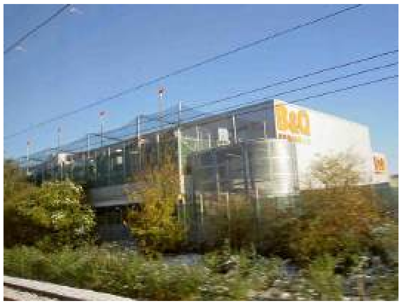

B&Q Stevenage © Steve Fareham (cc-by-sa/2.0)

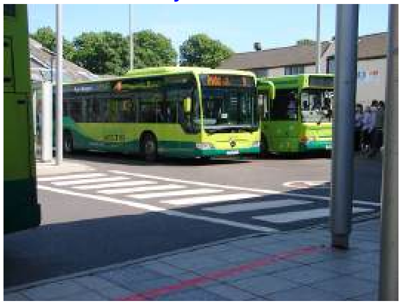

Newport bus station © John Lucas (cc-by-sa/2.0)

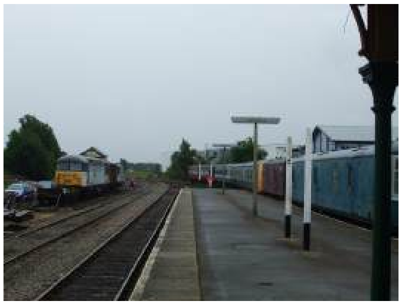

Dereham station © Ashley Dace (cc-by-sa/2.0)

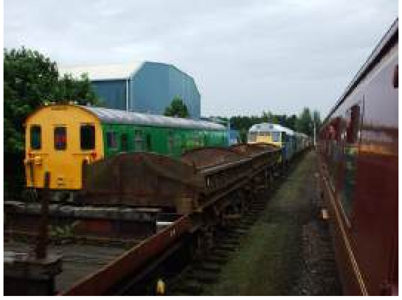

Dereham sidings © Ashley Dace (cc-by-sa/2.0)

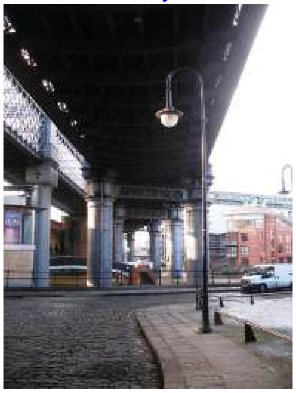

Under the railway © SMJ (cc-by-sa/2.0)

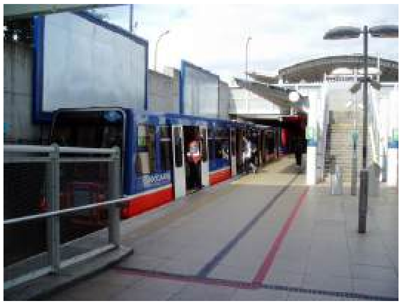

Lewisham DLR station © Dr Neil Clifton (cc-by-sa/2.0)

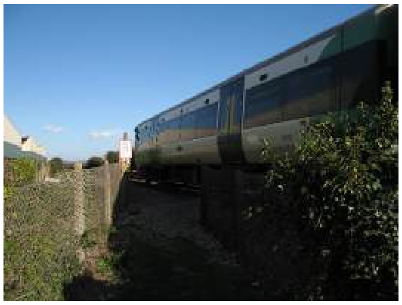

Train crossing © Mark Holland (cc-by-sa/2.0)

3.114 Public Utility

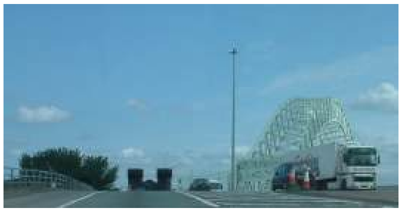

Approaching The Bridge © Gerald England (cc-by-sa/2.0)

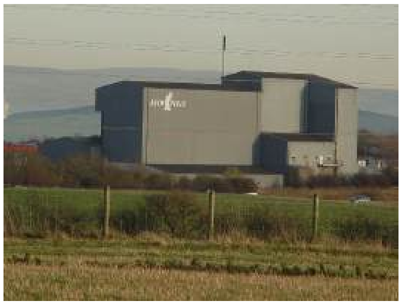

Modern factory, Penrith © Malcolm Street (cc-by-sa/2.0)

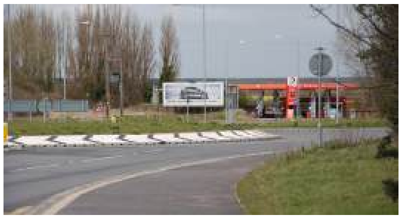

Richborough roundabout © david mills (cc-by-sa/2.0)

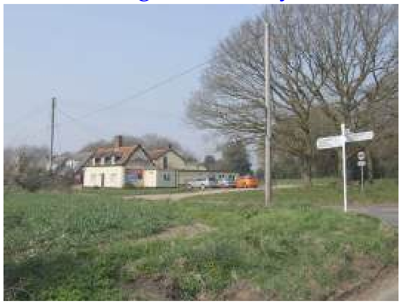

The Garden House © Ian Robertson (cc-by-sa/2.0)

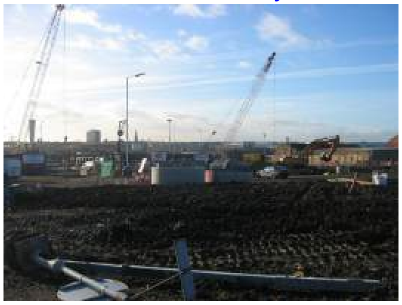

Work on the New Tyne Tunnel Crossing, Howdon © Les Hull (cc-by-sa/2.0)

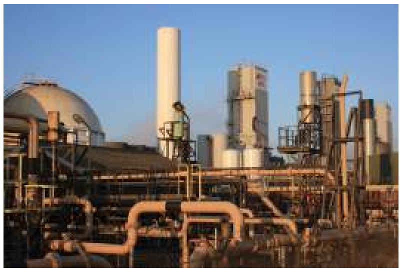

B.O.C Works © Mick Garratt (cc-by-sa/2.0)

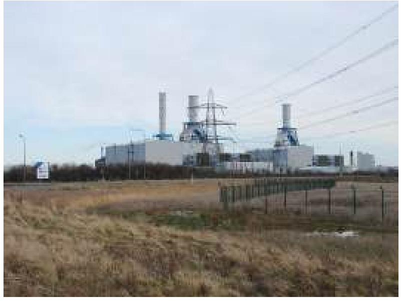

South Humber Power Station © Ian Paterson (cc-by-sa/2.0)

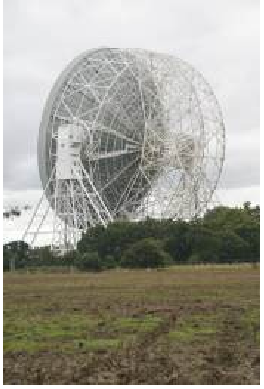

Jodrell Bank Radio telescope © Richard Styles (cc-by-sa/2.0)

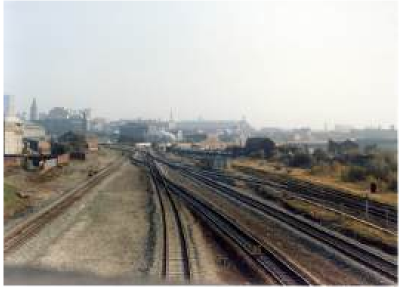

Ordsall Lane junction - new layout for Windsor Link © Peter Whatley (cc-by-sa/2.0)

3.115 Railway

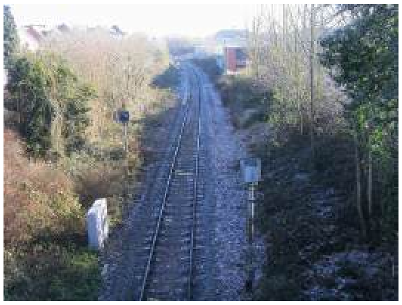

Single track towards the Cowley Works © Shaun Ferguson (cc-by-sa/2.0)

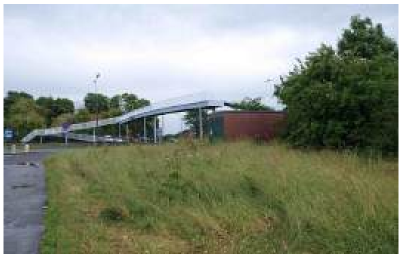

Footbridge crossing the A1 © Steve Fareham (cc-by-sa/2.0)

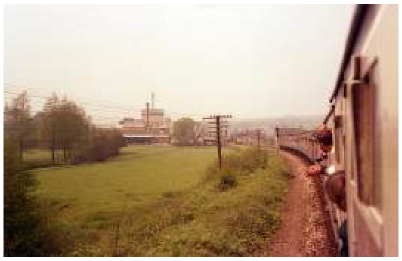

Torbay Express © John Lucas (cc-by-sa/2.0)

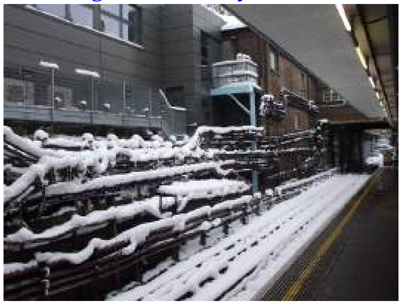

Earls Court Station on a snowy day, looking west © John Lord (cc-by-sa/2.0)

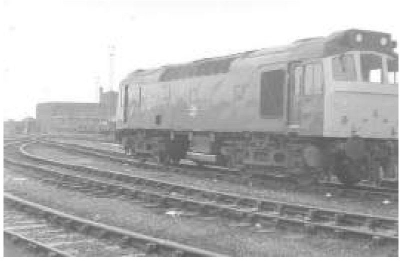

Newton Heath railway lands © Peter Whatley (cc-by-sa/2.0)

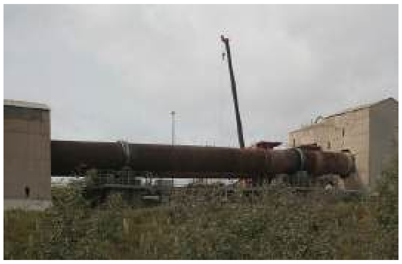

Rotary furnace at Steetley Dolomite © Alan Murray-Rust (cc-by-sa/2.0)

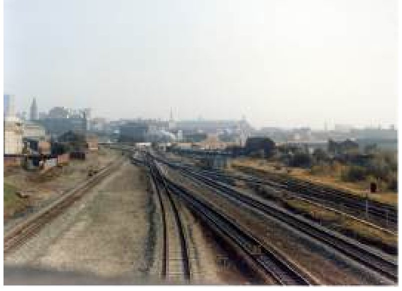

Ordsall Lane junction - new layout for Windsor Link © Peter Whatley (cc-by-sa/2.0)

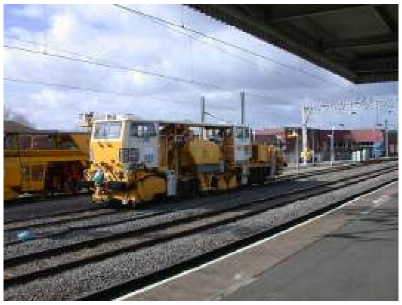

Ballast Regulator at Nuneaton © Keith Edkins (cc-by-sa/2.0)

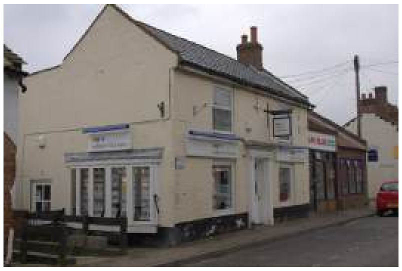

Estate Agents, Stalham High St © Pauline A Marsh (cc-by-sa/2.0)

3.116 Real Estate

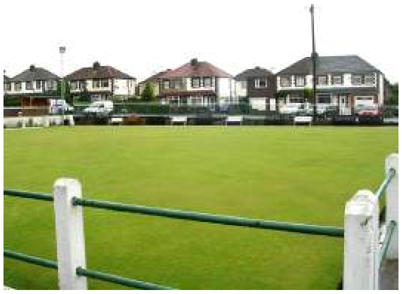

Old Bank Bowling Green - Sunny Bank Avenue, Sunny Bank Road © Betty Longbottom (cc-by-sa/2.0)

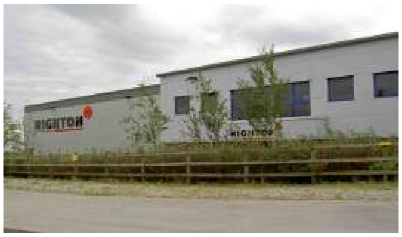

Righton © Steve Fareham (cc-by-sa/2.0)

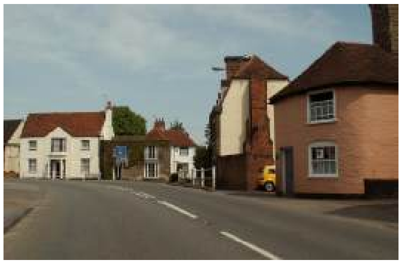

Old houses along the B1024 at Kelvedon © Robert Edwards (cc-by-sa/2.0)

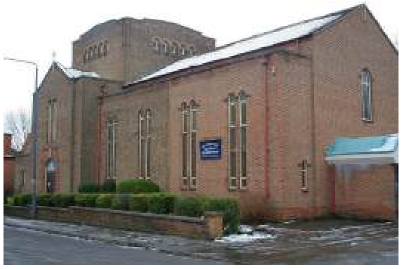

Our Lady of the Assumption © David Lally (cc-by-sa/2.0)

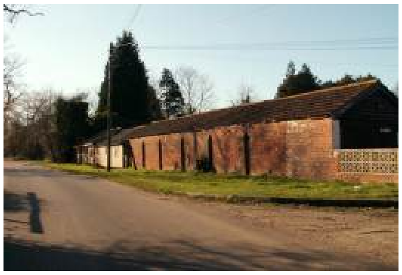

Part of Reed's Farm on Cow Watering Lane © Robert Edwards (cc-by-sa/2.0)

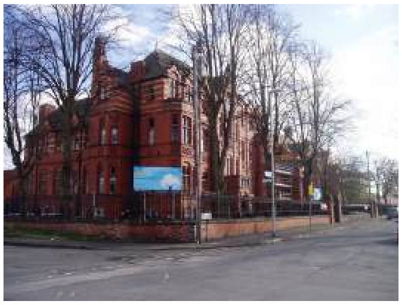

William Hulme Junior School Whalley Range © R Greenhalgh (cc-by-sa/2.0)

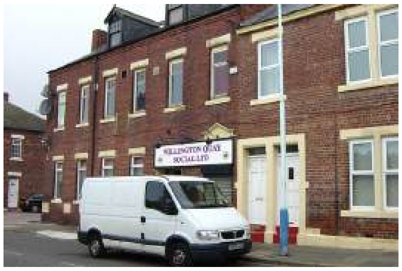

Willington Quay Social © Mac McCarron (cc-by-sa/2.0)

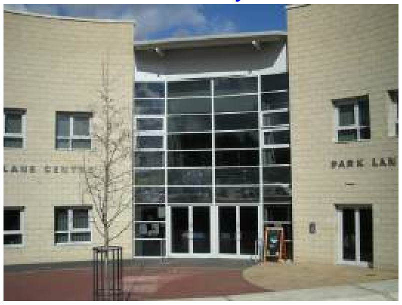

Entrance to the new Park Lane Community Centre, Woodside. © Row17 (cc-by-sa/2.0)

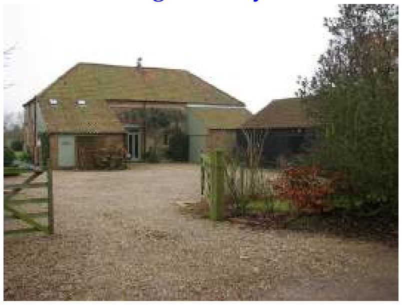

An attractive converted barn © Evelyn Simak (cc-by-sa/2.0)

3.117 Recreation

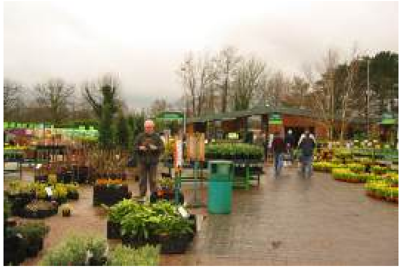
Bridgemere Garden World © Espresso Addict (cc-by-sa/2.0)

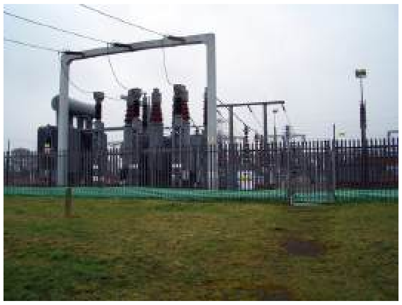
Electric grid Station © Keith Wright (cc-by-sa/2.0)

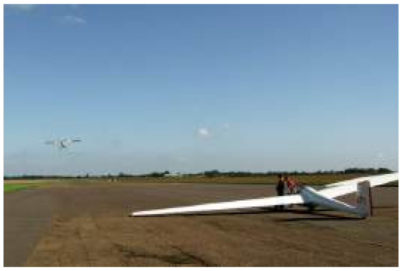
Rattlesden Gliding Club © David Ayrton (cc-by-sa/2.0)

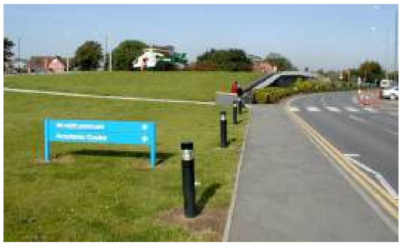
Great North Air Ambulance © Mick Garratt (cc-by-sa/2.0)

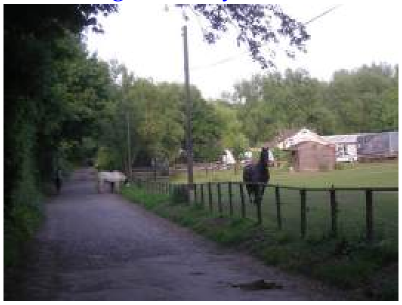
Telford Equestrian Centre © Row17 (cc-by-sa/2.0)

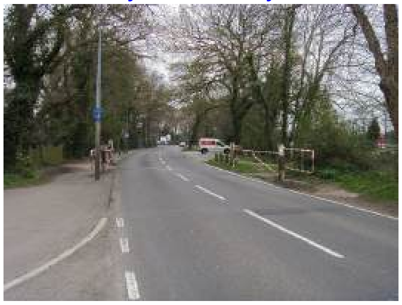
Passing across old railways tracks © Shaun Ferguson (cc-by-sa/2.0)

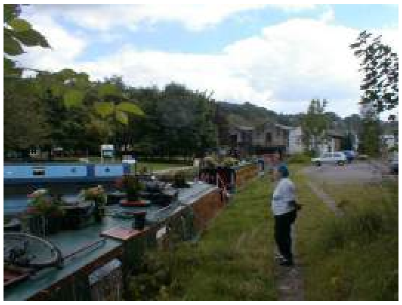
Whaley Bridge © Gerald England (cc-by-sa/2.0)

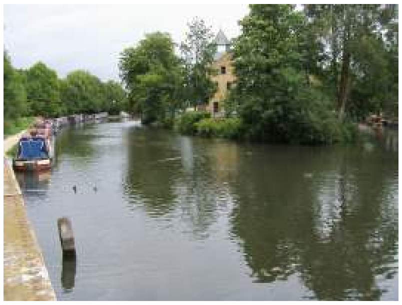
Grand Union Canal © Shaun Ferguson (cc-by-sa/2.0)

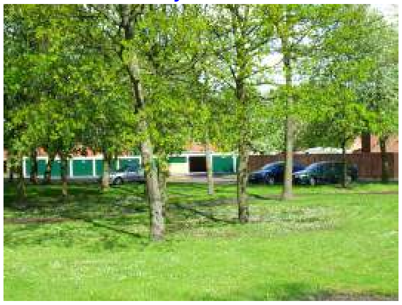
Garage block through the trees © Oliver Dixon (cc-by-sa/2.0)

3.118 Reflection

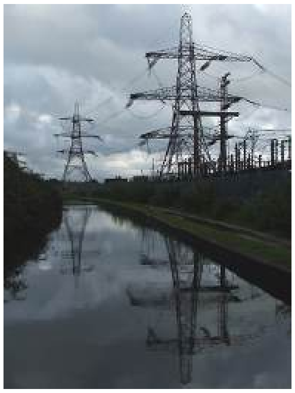

Canal and Pylons, Nechells, Birmingham © Roger D Kidd (cc-by-sa/2.0)

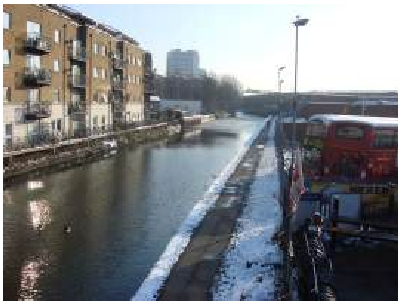

Grand Union Canal from Great Western Rd © Oxyman (cc-by-sa/2.0)

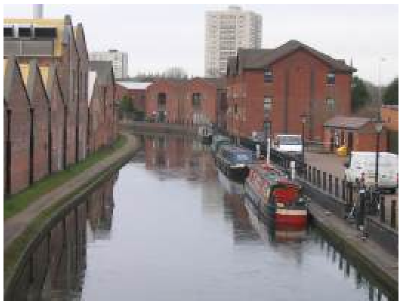

Birmingham & Fazeley Canal from Cuckoo Bridge © Roy Hughes (cc-by-sa/2.0)

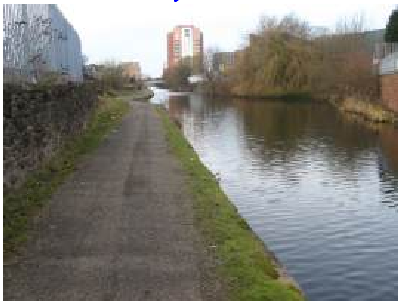

Ashton Canal © Chris Wimbush (cc-by-sa/2.0)

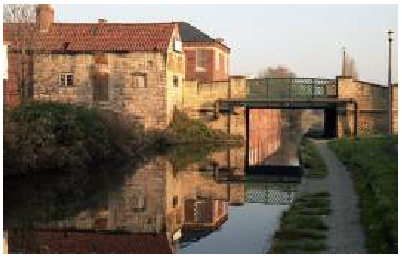

Worksop - Prior Well Bridge © Dave Bevis (cc-by-sa/2.0)

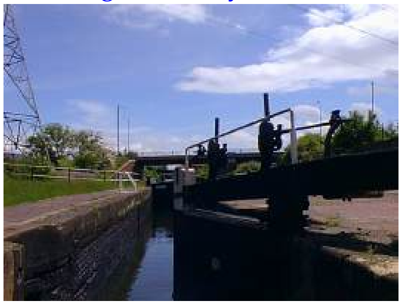

Minworth Top Lock © Nick Atty (cc-by-sa/2.0)

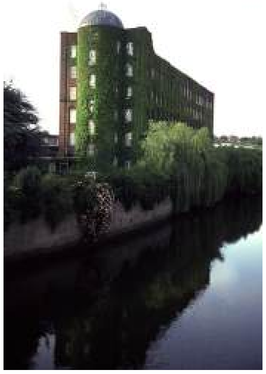

Jarrolds Mill, Cowgate © Chris Allen (cc-by-sa/2.0)

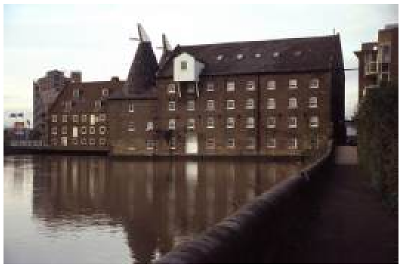

Three Mills, Bow © Chris Allen (cc-by-sa/2.0)

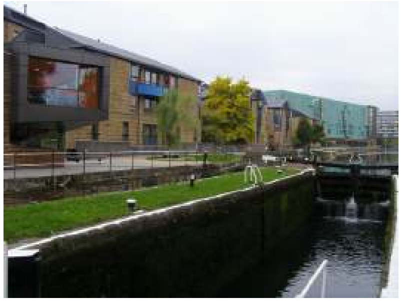

Mile End lock © Shaun Ferguson (cc-by-sa/2.0)

3.119 Reservoir

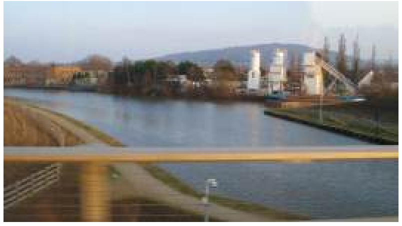

View from the new canal bridge © David Robinson (cc-by-sa/2.0)

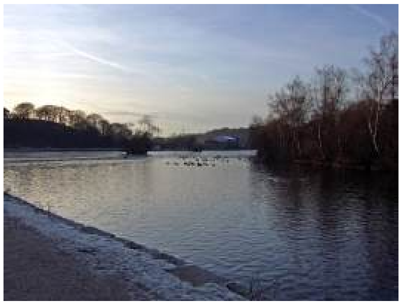

Etherow Country Park © Bob Abell (cc-by-sa/2.0)

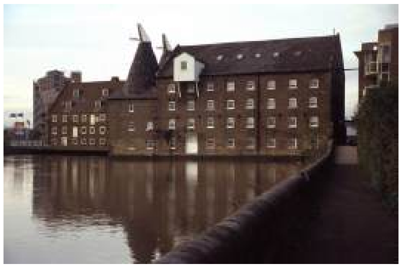

Three Mills, Bow © Chris Allen (cc-by-sa/2.0)

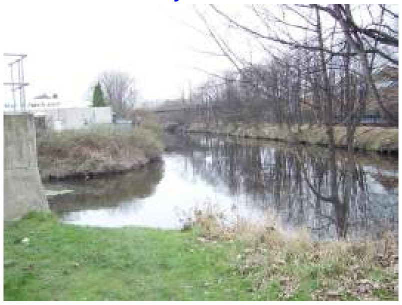

Confluence of River Rother with River Don © Shelagh Craven (cc-by-sa/2.0)

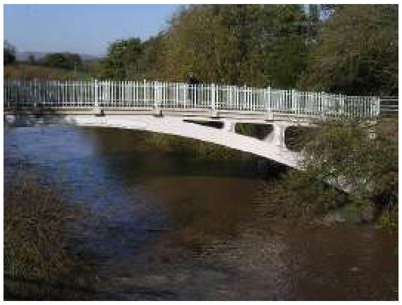

Stanford "old" bridge © Richard Greenwood (cc-by-sa/2.0)

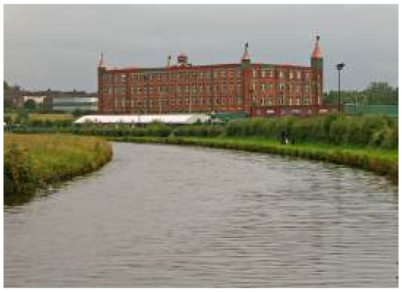

Botany Bay © Mr T (cc-by-sa/2.0)

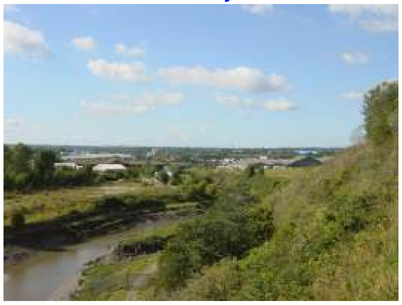

Ditton Marsh © Sue Adair (cc-by-sa/2.0)

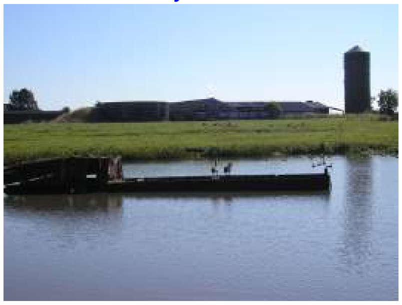

Higginslane Farm and Billinge Green Flash © Iain Lees (cc-by-sa/2.0)

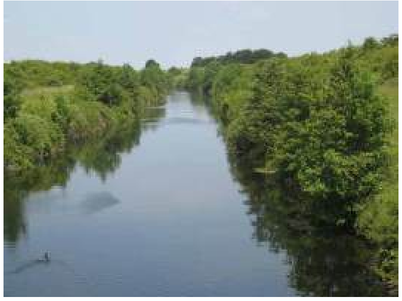

Cut-off Channel, near Feltwell © Oliver Dixon (cc-by-sa/2.0)

3.120 Residential Area

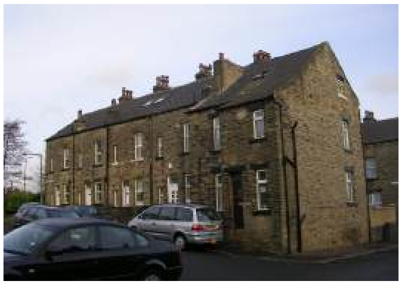

Hawthorn Terrace - Hyde Park Road © Betty Longbottom (cc-by-sa/2.0)

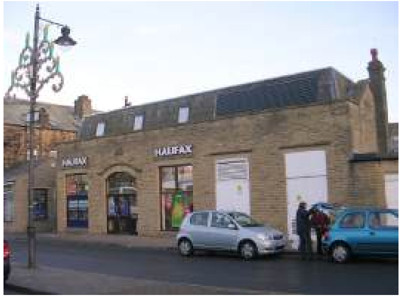

HBOS - Market Place © Betty Longbottom (cc-by-sa/2.0)

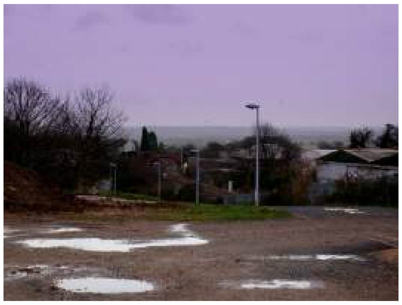

Chancel Bridge © Jan Baker (cc-by-sa/2.0)

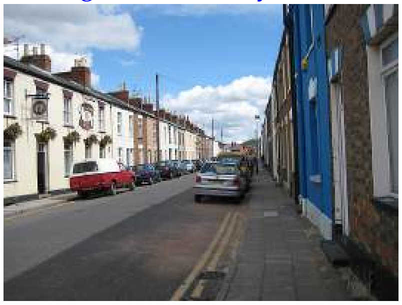

Adam & Eve pub, Townsend Street © Pauline E (cc-by-sa/2.0)

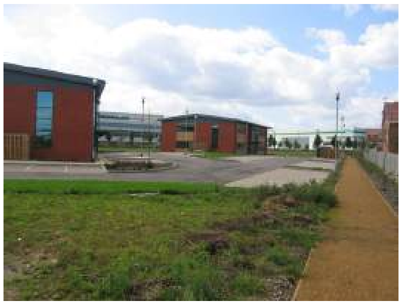

New offices at the site of Coventry Colliery © David Stowell (cc-by-sa/2.0)

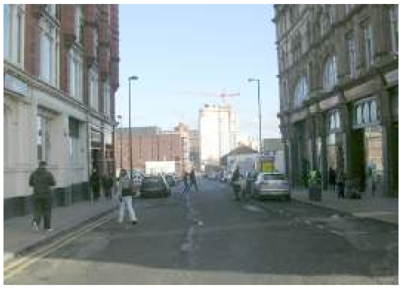

Ludgate Hill - Vicar Lane © Betty Longbottom (cc-by-sa/2.0)

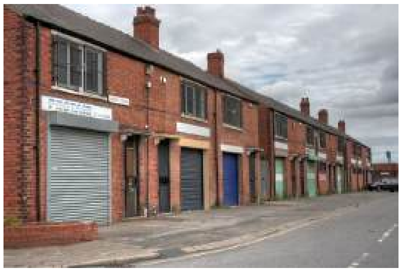

Lorne Street © Mick Garratt (cc-by-sa/2.0)

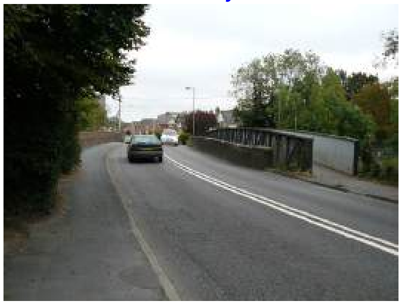

Andover - Weyhill Road © Chris Talbot (cc-by-sa/2.0)

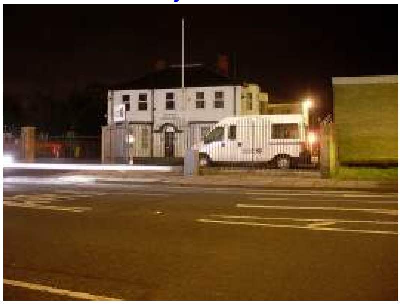

St John's Ambulance, Westgate Road © Stephen Sweeney (cc-by-sa/2.0)

3.121 Retail

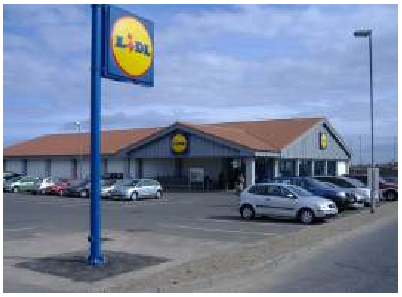

Lidl supermarket, Ryhope © Roger Cornfoot (cc-by-sa/2.0)

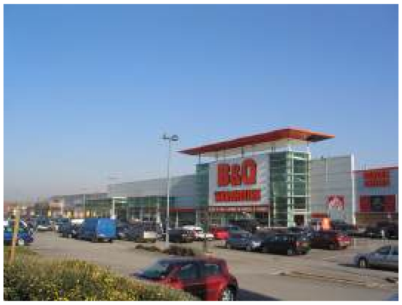

Weekend DIY © Tim Heaton (cc-by-sa/2.0)

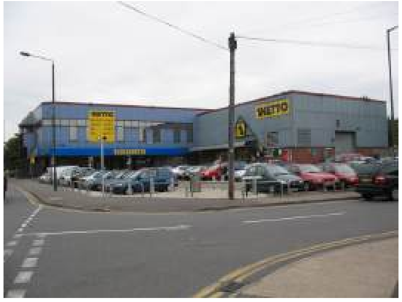

Shops on the corner of Love Lane © Stephen Craven (cc-by-sa/2.0)

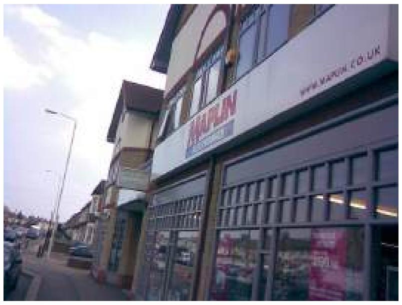

Maplin Store on Green Lane © Robert Lamb (cc-by-sa/2.0)

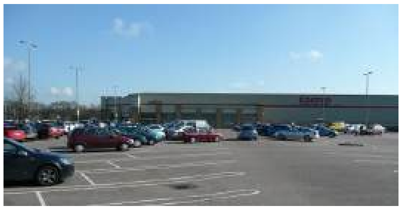

Costco superstore, Watford © Jonathan Billinger (cc-by-sa/2.0)

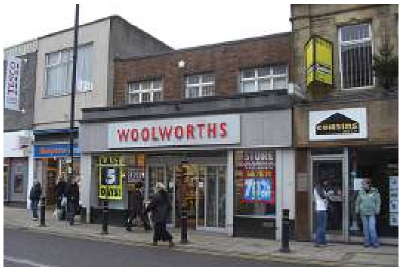

Woolworths, Market Street, Shaw © michael ely (cc-by-sa/2.0)

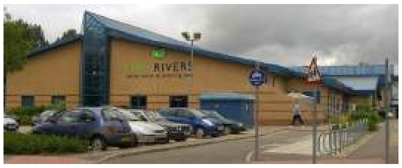

Five Rivers leisure centre in Salisbury © DHL (cc-by-sa/2.0)

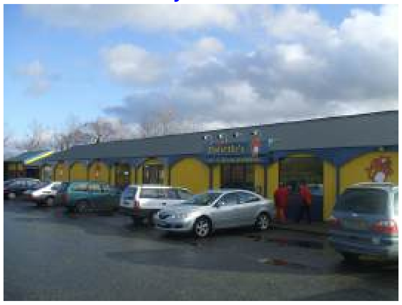

Pet Supplies © John M (cc-by-sa/2.0)

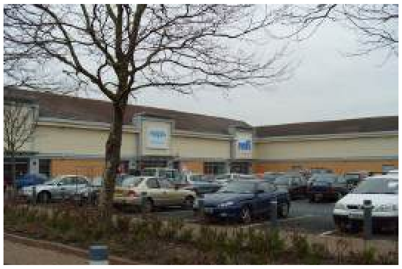

Retail Park At Blackpole © Mr M Evison (cc-by-sa/2.0)

3.122 Riparian Zone

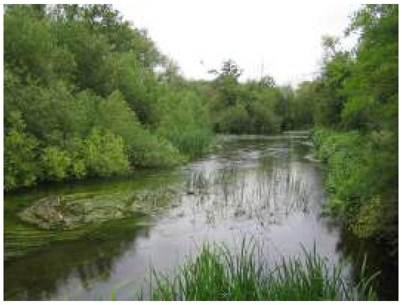
River Colne near West Drayton © Nigel Cox (cc-by-sa/2.0)

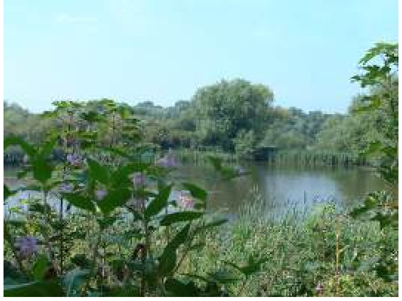
The Scrape © John Phillips (cc-by-sa/2.0)

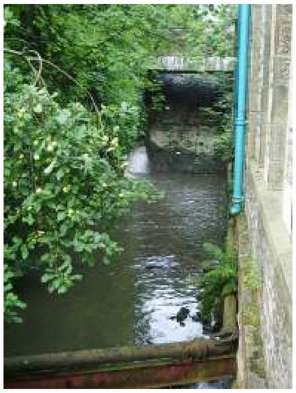
Mearley Brook as it flows along Stalwart Carpet Works © Alexander P Kapp (cc-by-sa/2.0)

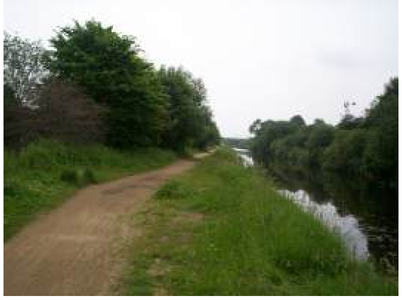
Sheffield to Keadby Canal © Jonathan Clitheroe (cc-by-sa/2.0)

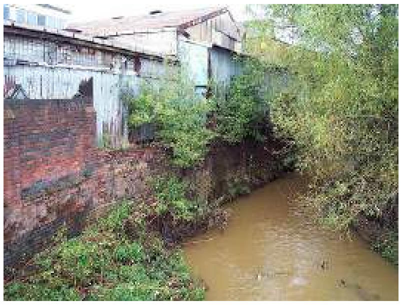
Industrial decline at the River Stour © Kieron McMahon (cc-by-sa/2.0)

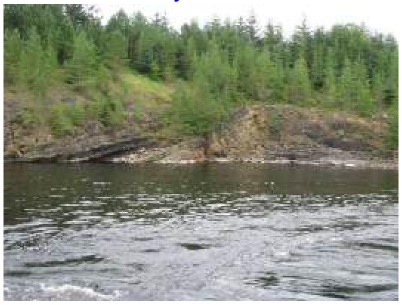
Plashetts Quarry © Pete Saunders (cc-by-sa/2.0)

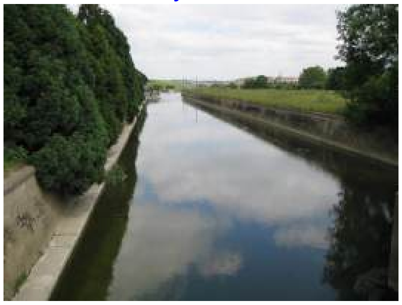
River Lee Diversion near Chingford © Nigel Cox (cc-by-sa/2.0)

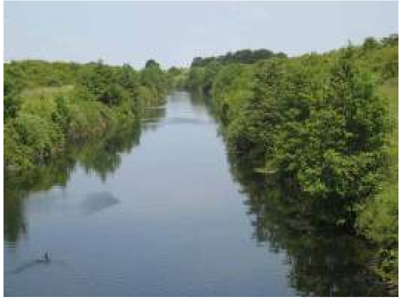
Cut-off Channel, near Feltwell © Oliver Dixon (cc-by-sa/2.0)

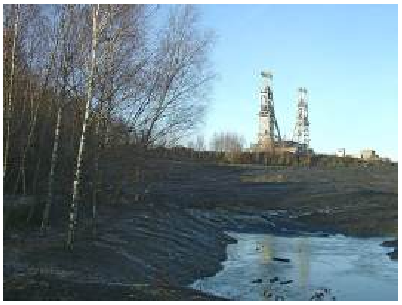
Clipstone Colliery from the Robin Hood Way © Alan Murray-Rust (cc-by-sa/2.0)

3.123 Road

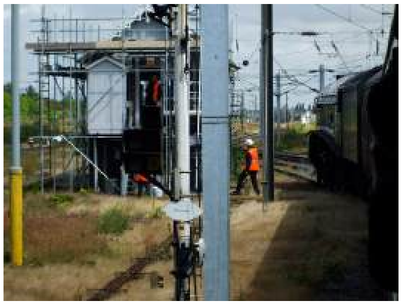

Steam locomotive and signal box © Ashley Dace (cc-by-sa/2.0)

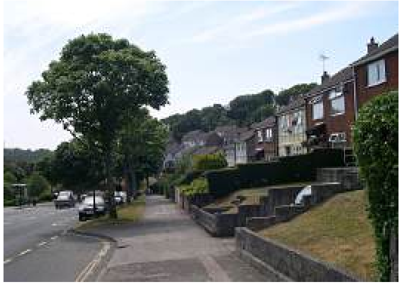

Southway Drive © Tony Atkin (cc-by-sa/2.0)

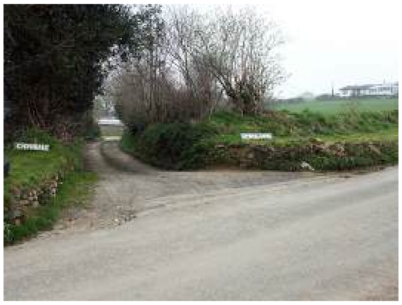

Lane leading to Chynhale © Fred James (cc-by-sa/2.0)

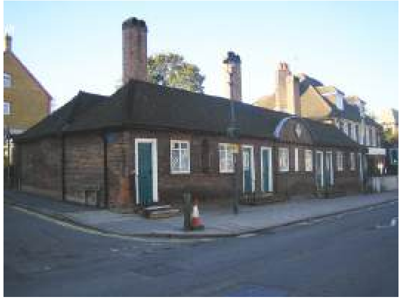

Berkhamsted: Sayer's Almshouses © Nigel Cox (cc-by-sa/2.0)

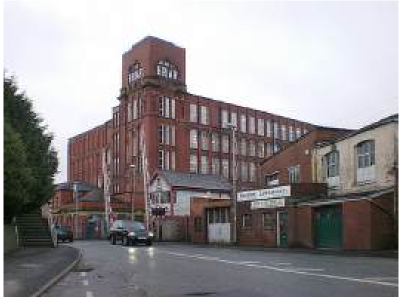

Briar Mill, Beal Lane, Shaw © Alexander P Kapp (cc-by-sa/2.0)

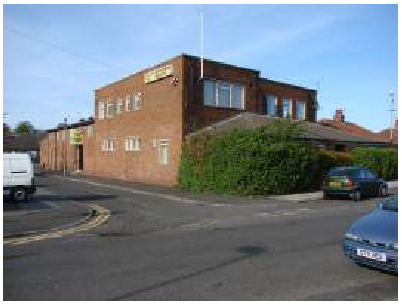

The Royal British Legion Ex Servicemen's Club, Forest Hall. © Bill Henderson (cc-by-sa/2.0)

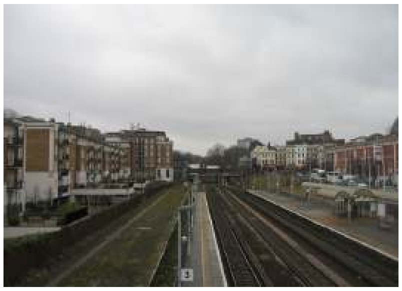

Kensington Olympia station © Mr Ignavy (cc-by-sa/2.0)

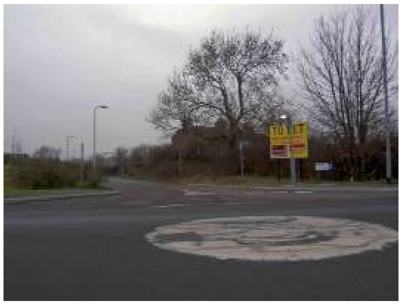

Temple Point office park mini roundabout © Steve Fareham (cc-by-sa/2.0)

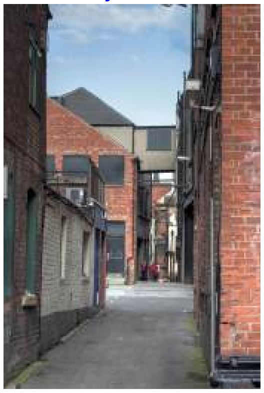

Mechanic's Yard © Mick Garratt (cc-by-sa/2.0)

3.124 Road Surface

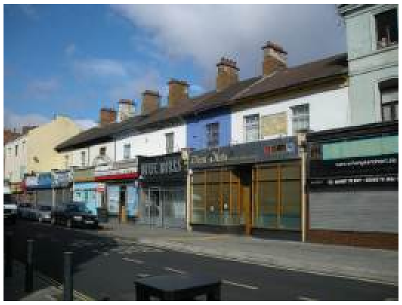

Coventry-Far Gosford Street © Ian Rob (cc-by-sa/2.0)

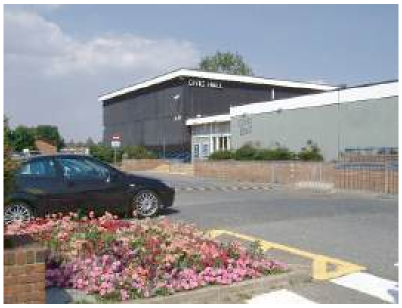

Grays Civic Hall (Blackshots) © Glyn Baker (cc-by-sa/2.0)

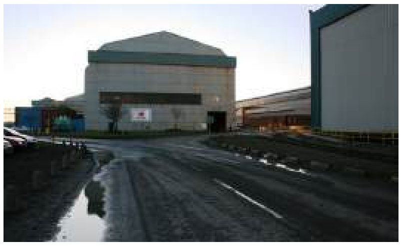

20″ Rolling Mill © Mick Garratt (cc-by-sa/2.0)

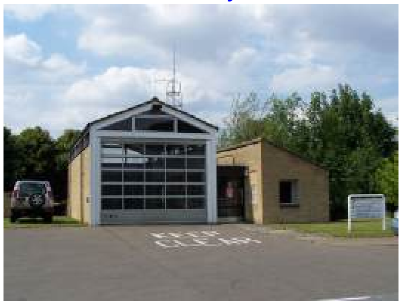

Ixworth Fire Station © Geoff Pick (cc-by-sa/2.0)

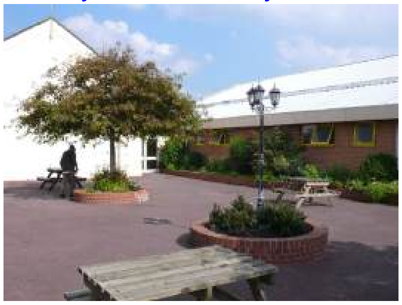

Lyme Bay Holiday Village, Seaton © Nigel Mykura (cc-by-sa/2.0)

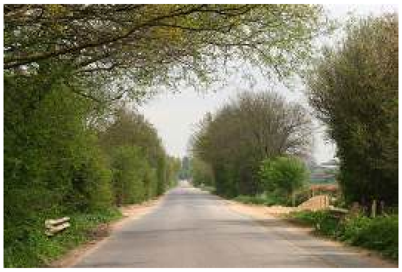

Cassington, the course of the old railway line © Martin Loader (cc-by-sa/2.0)

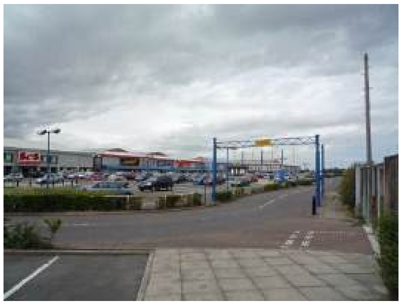

Entrance to the Alexandra Dock Retail Park © David Wright (cc-by-sa/2.0)

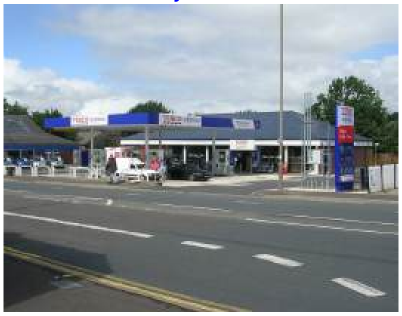

Tesco Express - Wakefield Road © Betty Longbottom (cc-by-sa/2.0)

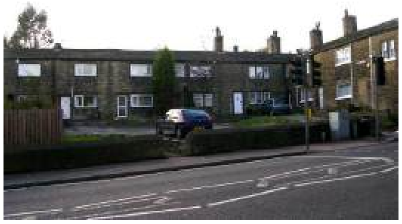

Mason Square - off Keighley Road © Betty Longbottom (cc-by-sa/2.0)

3.125 Rock

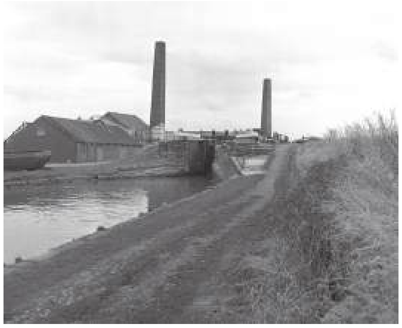
The tail of Lock No 73, Trent and Mersey Canal © Dr Neil Clifton (cc-by-sa/2.0)

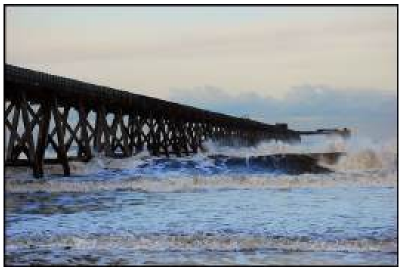
Steetley pier © philld (cc-by-sa/2.0)

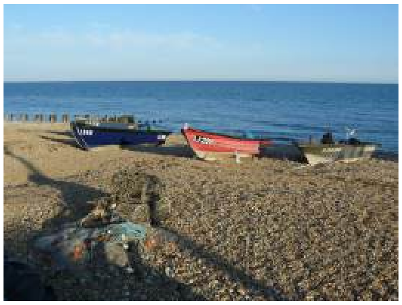
Bognor Beach © Martin Horsfall (cc-by-sa/2.0)

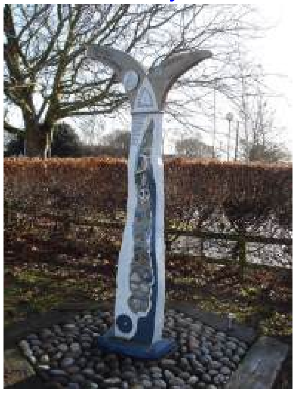
National cycle route marker post by Ipswich hospital © Oxymoron (cc-by-sa/2.0)

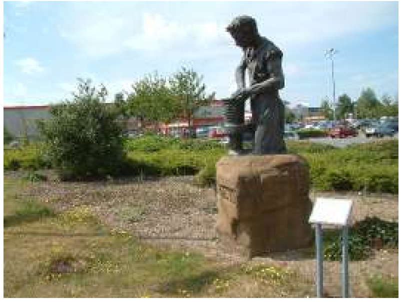
The Craftsman © Stuart Buchan (cc-by-sa/2.0)

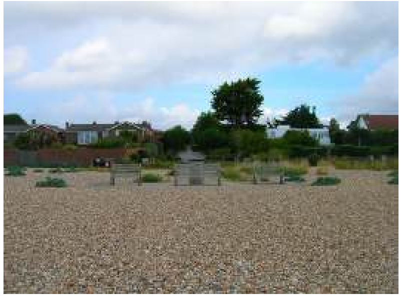
Aldwick Bay Estate © Simon Carey (cc-by-sa/2.0)

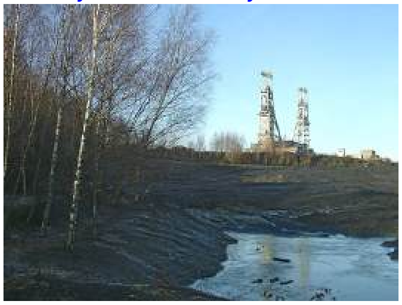
Clipstone Colliery from the Robin Hood Way © Alan Murray-Rust (cc-by-sa/2.0)

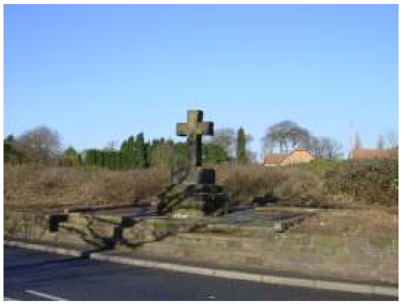
Ancient Stone Cross, Mill Lane © Sue Adair (cc-by-sa/2.0)

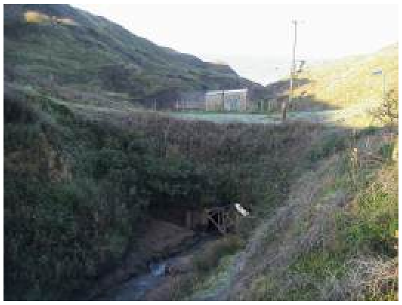
Shepherd's Chine © Shaun Ferguson (cc-by-sa/2.0)

3.126 Rolling

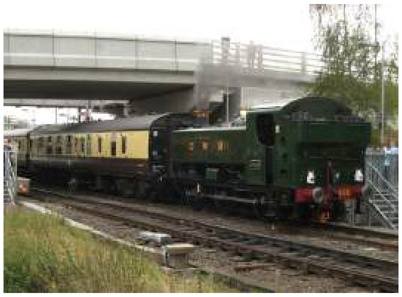

First steam hauled train at Coleshill Parkway © David Stowell (cc-by-sa/2.0)

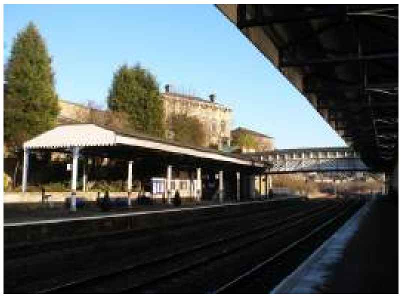

Dewsbury Station © SMJ (cc-by-sa/2.0)

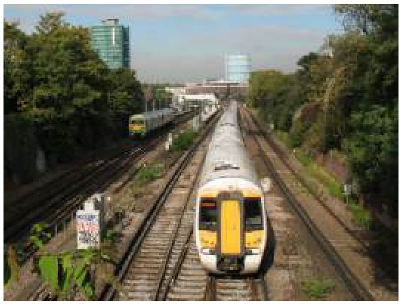

Main line from Victoria © Stephen Craven (cc-by-sa/2.0)

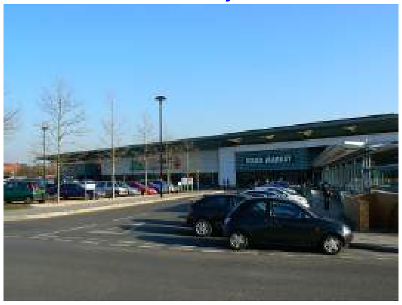

Asda Wal-mart, Thamesdown Drive, Swindon © Brian Robert Marshall (cc-by-sa/2.0)

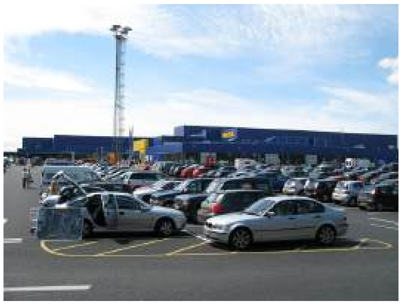

Ikea Warrington © Paul Anderson (cc-by-sa/2.0)

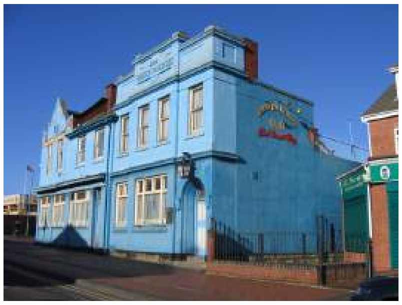

Waggon & Horses Pub, Sandwell © Roy Hughes (cc-by-sa/2.0)

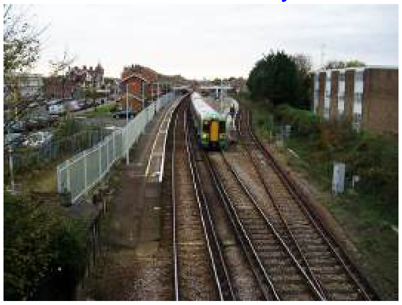

Worthing Station © Peter Holmes (cc-by-sa/2.0)

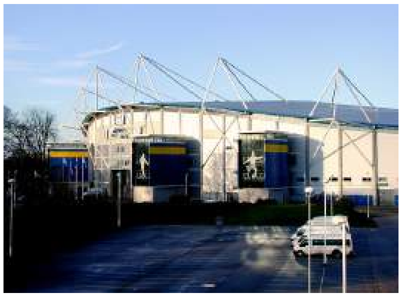

KC Stadium, Hull © Paul Glazzard (cc-by-sa/2.0)

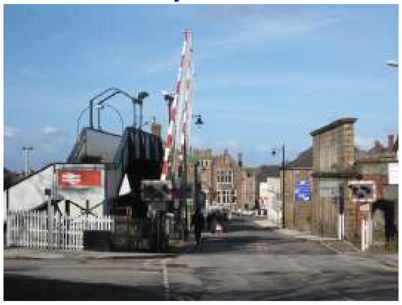

Level crossing at Camborne Station © Rod Allday (cc-by-sa/2.0)

3.127 Rural Area

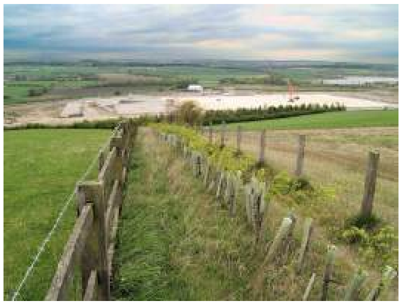

Site preparation for new warehouse. © Steve Fareham (cc-by-sa/2.0)

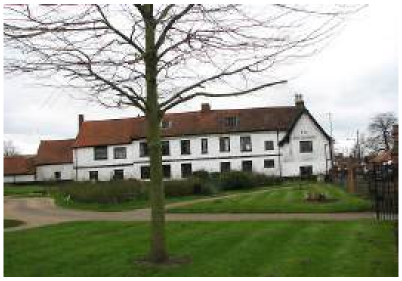

The Griffin Hotel © Evelyn Simak (cc-by-sa/2.0)

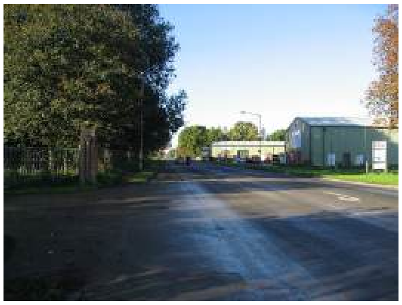

Entering Watton © Roger Gilbertson (cc-by-sa/2.0)

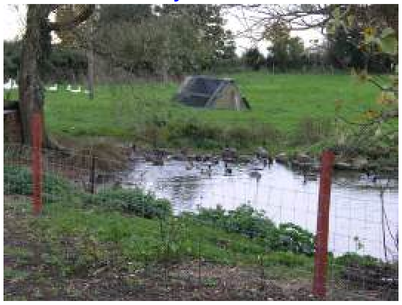

Duckpond in Shingleford Farm © Hywel Williams (cc-by-sa/2.0)

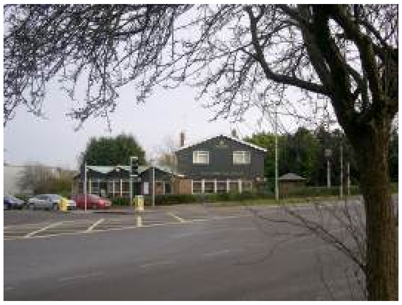

The Minden Rose public house, Bury St. Edmunds © John Goldsmith (cc-by-sa/2.0)

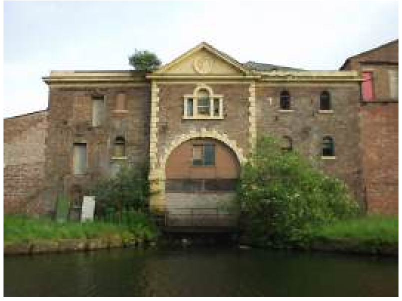

Canal Warehouse, Broadheath, Altrincham © Phil Champion (cc-by-sa/2.0)

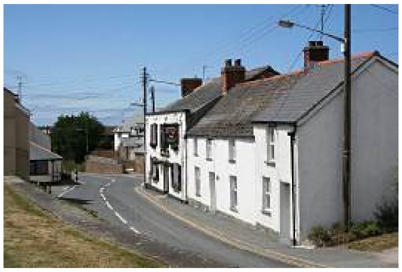

St Issey Village © Tony Atkin (cc-by-sa/2.0)

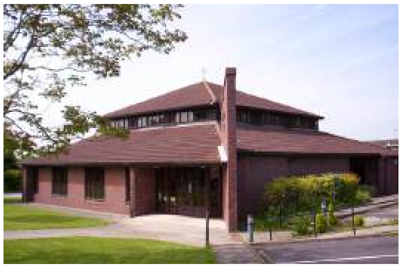

St Basil and All Saints - shared church © S Parish (cc-by-sa/2.0)

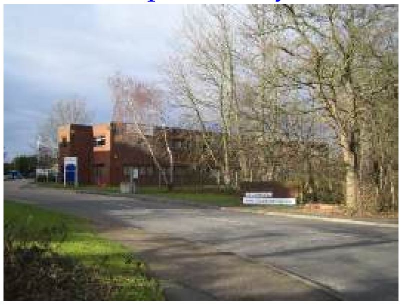

Borehamwood Industrial Estate © John Winterbottom (cc-by-sa/2.0)

3.128 Shade

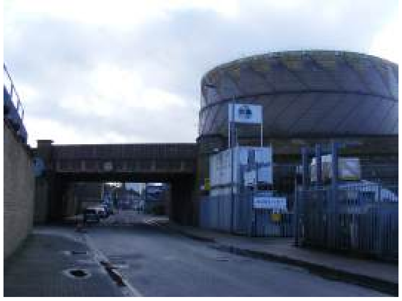

Rail Bridge over Smugglers Way © PAUL FARMER (cc-by-sa/2.0)

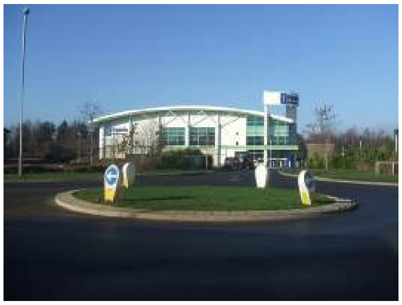

Health Club on the Broadlands Development © John M (cc-by-sa/2.0)

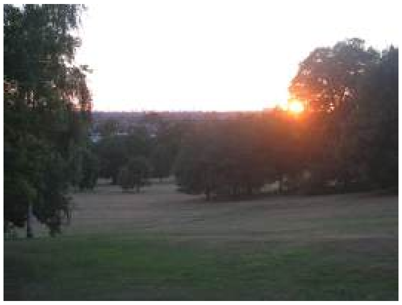

Elmdon Park, Solihull © John Evans (cc-by-sa/2.0)

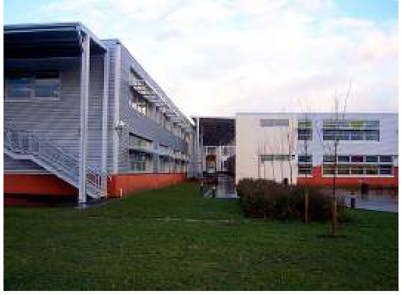

Holmesdale Technology College, Snodland © Richard Dorrell (cc-by-sa/2.0)

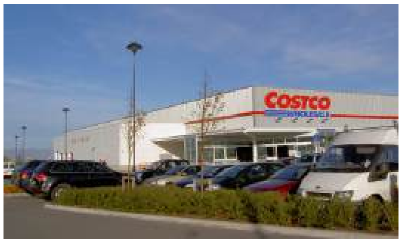

Costco warehouse Sheffield © Steve Fareham (cc-by-sa/2.0)

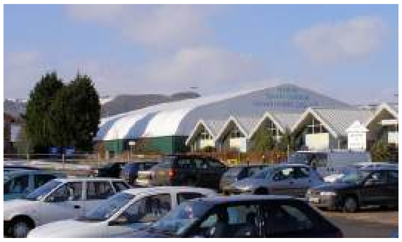

Harlow Sports Centre, Sussex Downs College, Eastbourne © Kevin Gordon (cc-by-sa/2.0)

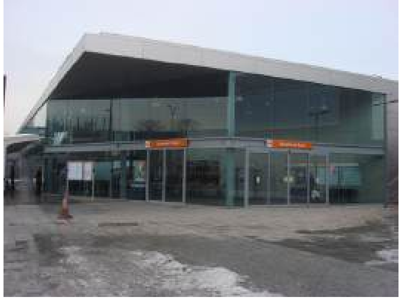

Shepherd's Bush London Overground station © Oxyman (cc-by-sa/2.0)

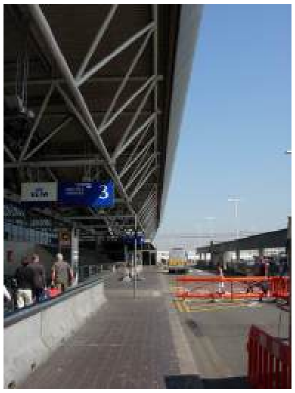

Heathrow Terminal 4 © Hugh Venables (cc-by-sa/2.0)

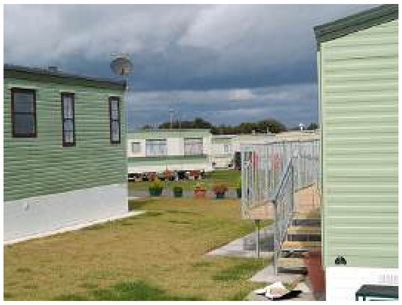

Hazel Grove Holiday Park © Stephen McCulloch (cc-by-sa/2.0)

3.129 Shoulder

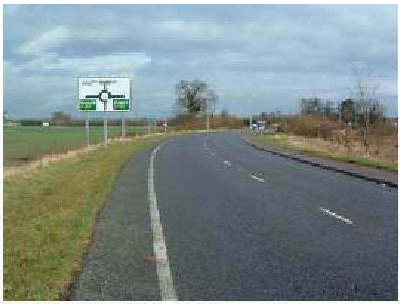

Roundabout Ahead © Keith Evans (cc-by-sa/2.0)

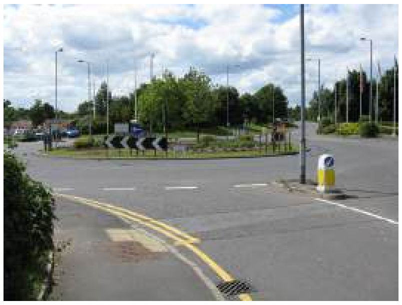

Wainwright Road roundabout © Peter Whatley (cc-by-sa/2.0)

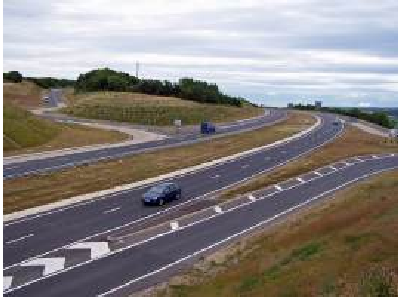

Site of Merrymeet roundabout © Richard Dorrell (cc-by-sa/2.0)

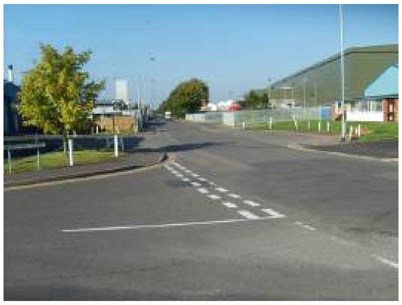

Heath Mill Close © Gordon Griffiths (cc-by-sa/2.0)

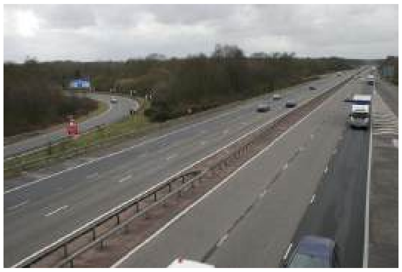

Hawley © Brendan and Ruth McCartney (cc-by-sa/2.0)

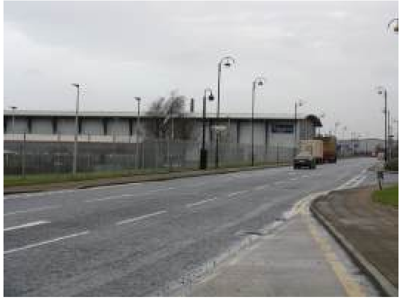

Trafford Park - Third Avenue, looking north © Peter Whatley (cc-by-sa/2.0)

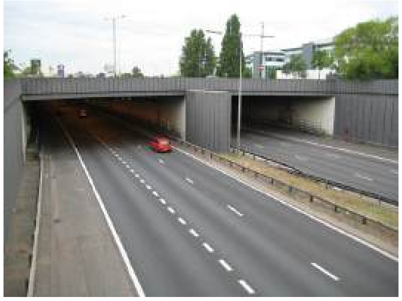

A1(M) Hatfield Tunnel © Nigel Cox (cc-by-sa/2.0)

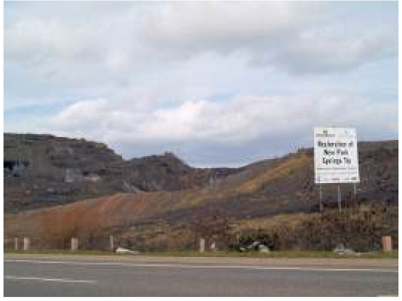

New park springs aka Grimethorpe 'muck stack'. © Steve Fareham (cc-by-sa/2.0)

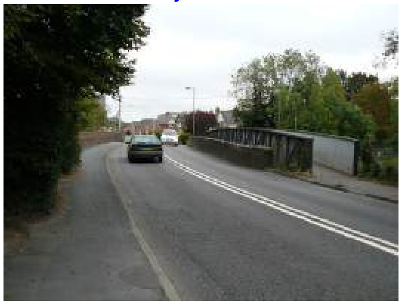

Andover - Weyhill Road © Chris Talbot (cc-by-sa/2.0)

3.130 Shrub

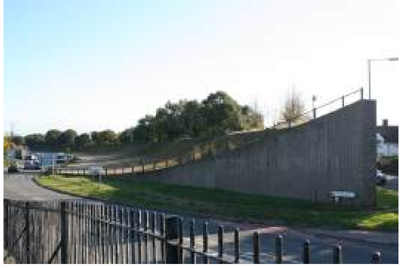

Brooklands circuit © David Ashcroft (cc-by-sa/2.0)

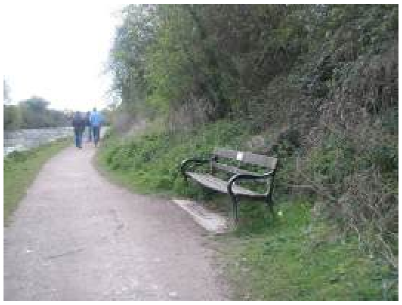

Walkers passing Peter West's seat © Basher Eyre (cc-by-sa/2.0)

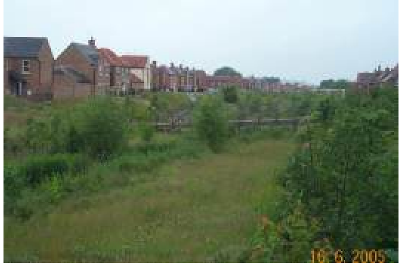

Aylesbury: Bear Brook, Fairford Leys © Nigel Cox (cc-by-sa/2.0)

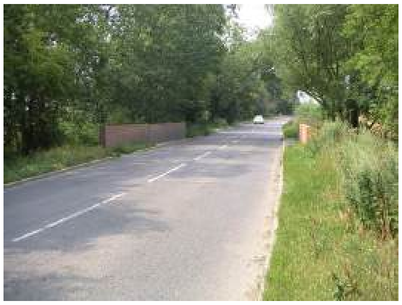

Kingston Bridge © Mr Biz (cc-by-sa/2.0)

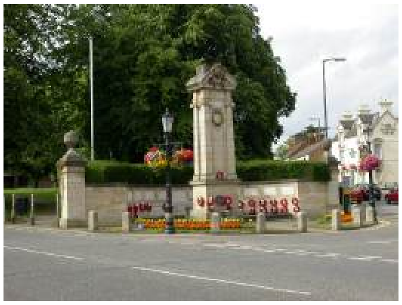

War Memorial, Broad Green © Kokai (cc-by-sa/2.0)

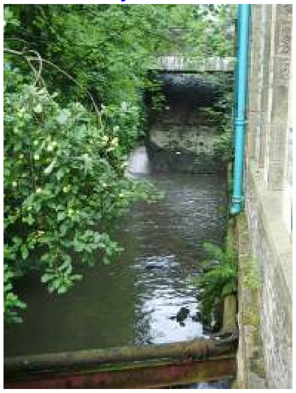

Mearley Brook as it flows along Stalwart Carpet Works © Alexander P Kapp (cc-by-sa/2.0)

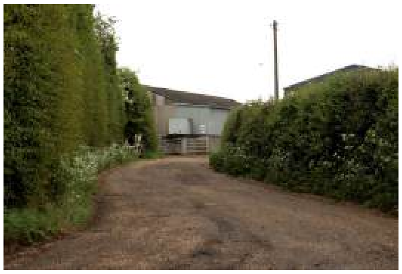

Part of Ampers Wick Farm © Robert Edwards (cc-by-sa/2.0)

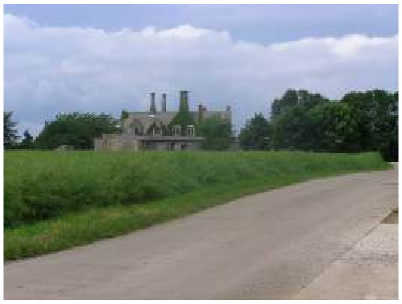

Park House : Stanwick St.John © Hugh Mortimer (cc-by-sa/2.0)

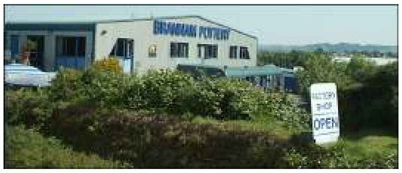

Brannam Pottery, Barnstaple © Mike Crowe (cc-by-sa/2.0)

3.131 Sidewalk

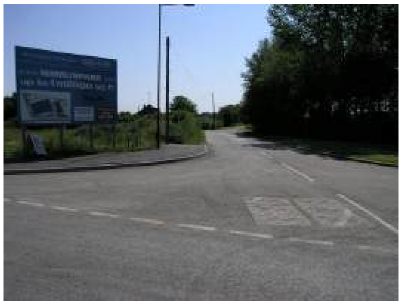

Nimbuspark © Michael Patterson (cc-by-sa/2.0)

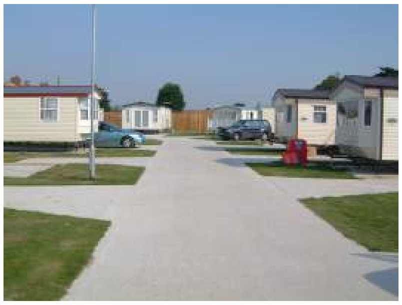

Caravan Park, Greatstone-On-Sea © Stacey Harris (cc-by-sa/2.0)

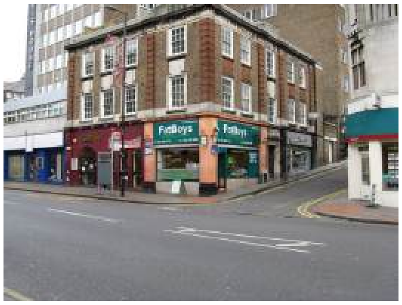

Fat Boys sandwich bar, Croydon © Dr Neil Clifton (cc-by-sa/2.0)

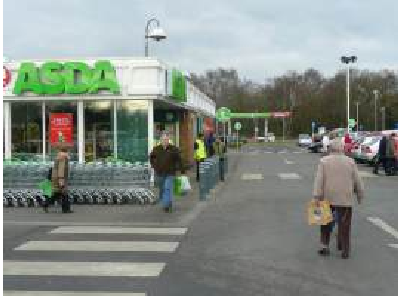

Asda superstore, Scunthorpe © Jonathan Billinger (cc-by-sa/2.0)

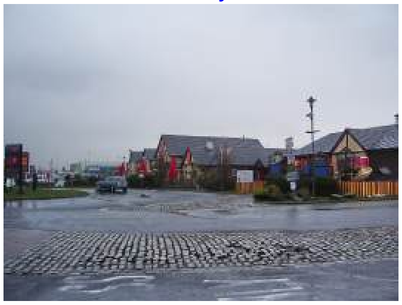

The Air Balloon, Squires Gate Lane, Blackpool © Alexander P Kapp (cc-by-sa/2.0)

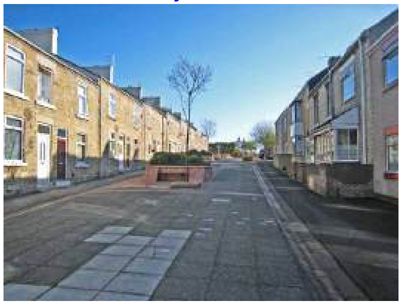

Half Moon Lane, Spennymoor © Oliver Dixon (cc-by-sa/2.0)

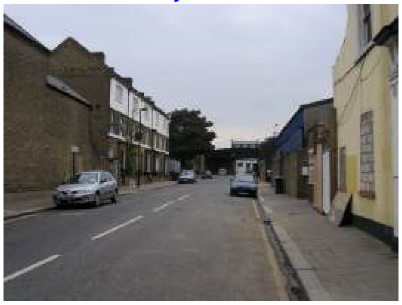

Padfield road © Shaun Ferguson (cc-by-sa/2.0)

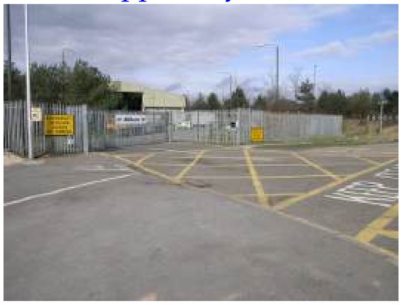

Highways Depot © Michael Patterson (cc-by-sa/2.0)

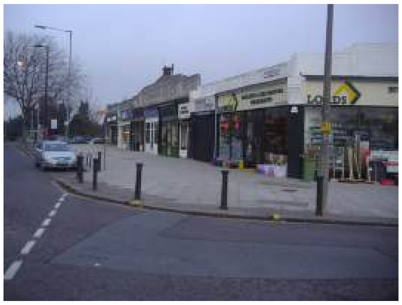

Bush Hill Parade © David Howard (cc-by-sa/2.0)

3.132 Sky

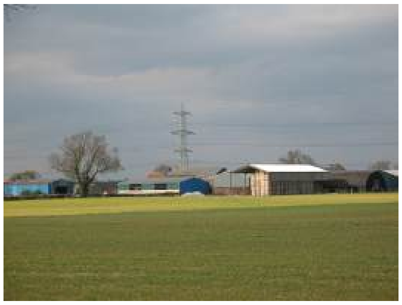

Forest Hall Farm near Alne © Gordon Hatton (cc-by-sa/2.0)

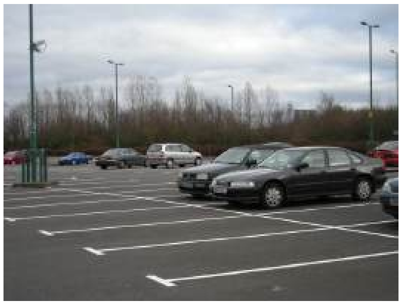

Meole Brace Park & Ride Car Park © Row17 (cc-by-sa/2.0)

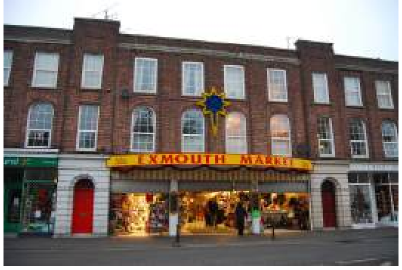

Exmouth Market © N Chadwick (cc-by-sa/2.0)

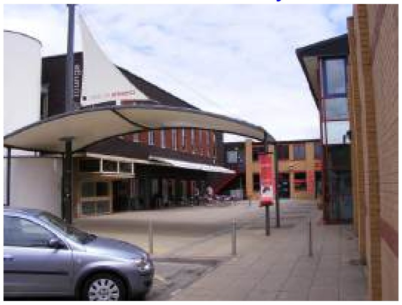

Staffordshire University Cafe © Gordon Griffiths (cc-by-sa/2.0)

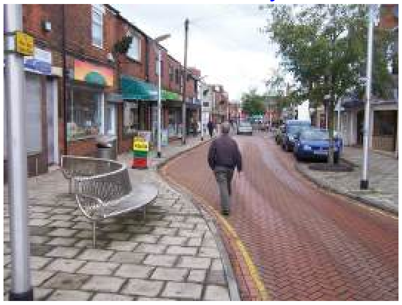

Street Scene - Scunthorpe © Colin Babb (cc-by-sa/2.0)

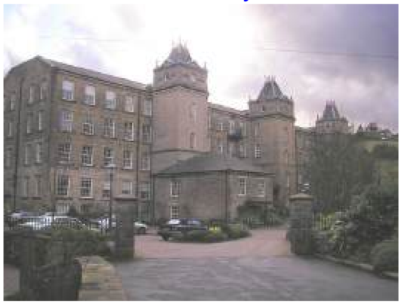

Barkisland Mill © John Illingworth (cc-by-sa/2.0)

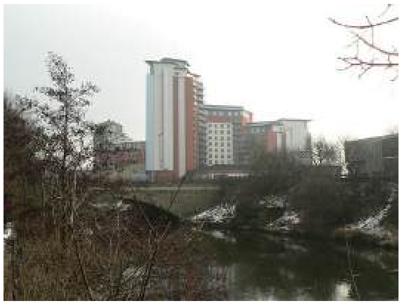

Wellington Road Bridge © Rich Tea (cc-by-sa/2.0)

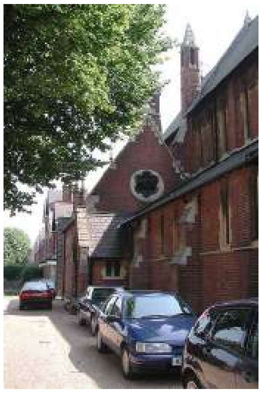

St Mary, Lansdowne Road, London N17 © John Salmon (cc-by-sa/2.0)

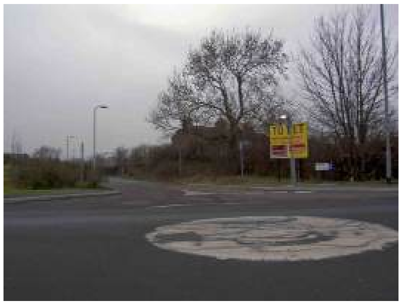

Temple Point office park mini roundabout © Steve Fareham (cc-by-sa/2.0)

3.133 Slope

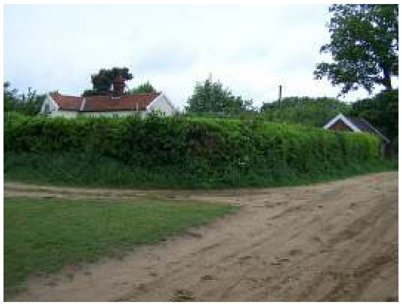

Cottage entrance © Claire Haystead (cc-by-sa/2.0)

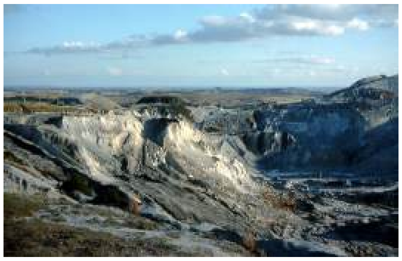

Lee Moor China Clay Works 1979 © Crispin Purdye (cc-by-sa/2.0)

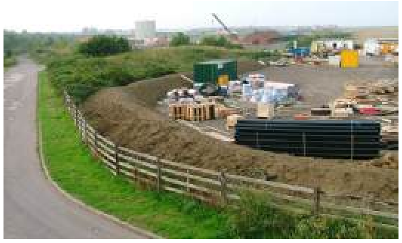

Builders Storage Yard © Mick Garratt (cc-by-sa/2.0)

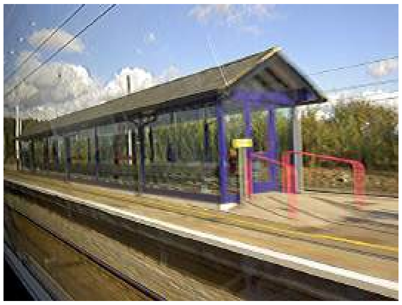

Speeding through St Neots railway station © Steve Fareham (cc-by-sa/2.0)

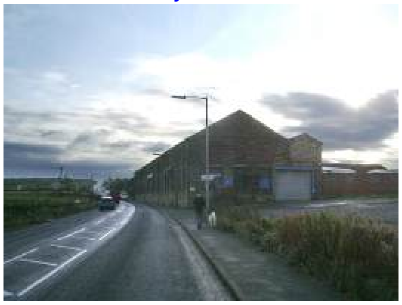

Denholme Velvet © Alexander P Kapp (cc-by-sa/2.0)

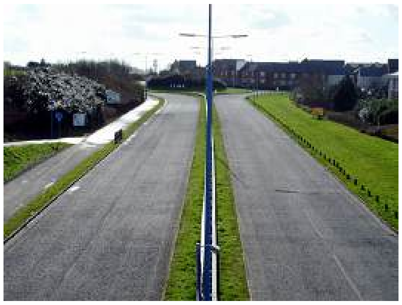

Looking south along Great Stoke Way © Linda Bailey (cc-by-sa/2.0)

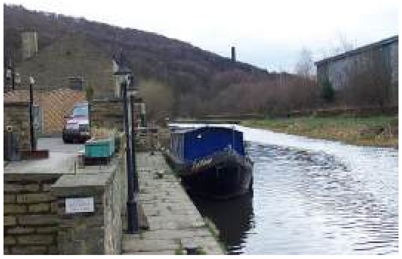

Narrow Boat on the Calder & Hebble Navigation at Elland Park Wood © Richard Kay (cc-by-sa/2.0)

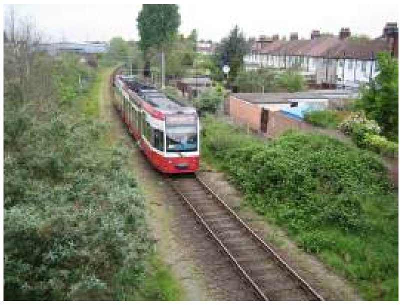

Mitcham: Tramlink © Nigel Cox (cc-by-sa/2.0)

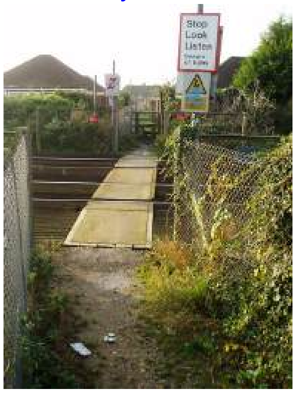

Footpath crossing West Coastway rail line © Peter Holmes (cc-by-sa/2.0)

3.134 Snow

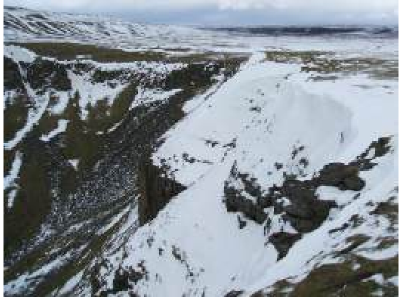

High Cup Nick © David Brown (cc-by-sa/2.0)

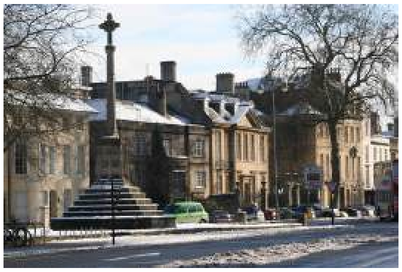

St Giles © Chris Denny (cc-by-sa/2.0)

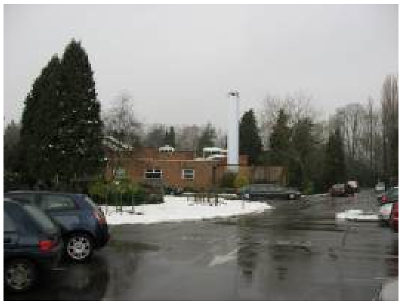

Aldershot Crematorium © Bilbo (cc-by-sa/2.0)

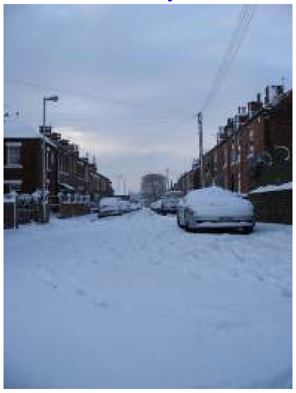

George Street in the snow © SMJ (cc-by-sa/2.0)

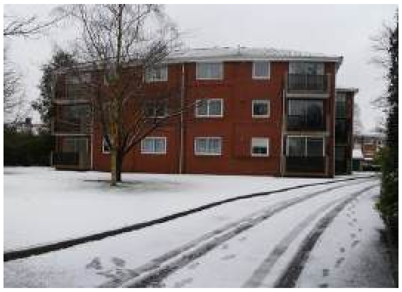

Bournemouth : Flats on Wimborne Road © Lewis Clarke (cc-by-sa/2.0)

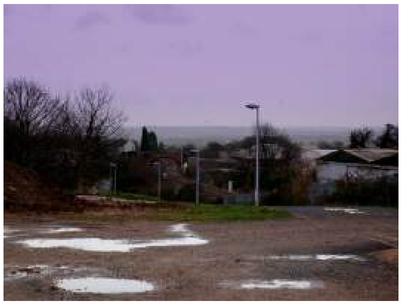

Chancel Bridge © Jan Baker (cc-by-sa/2.0)

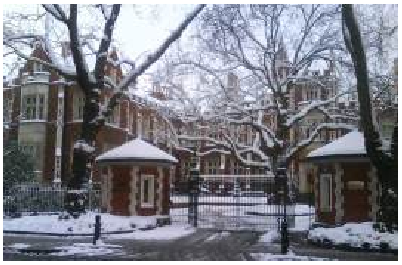

Old Royal Brompton Hospital Fulham Road © PAUL FARMER (cc-by-sa/2.0)

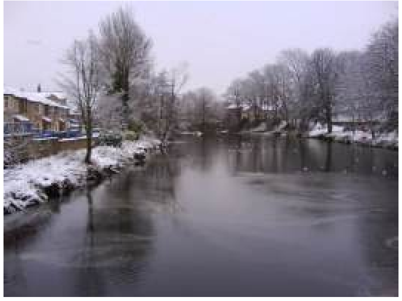

Downstream from Elliott Bridge © john sayers (cc-by-sa/2.0)

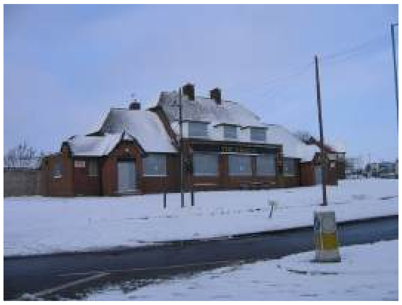

The Dingle Public House. © Roy Hughes (cc-by-sa/2.0)

3.135 Soil

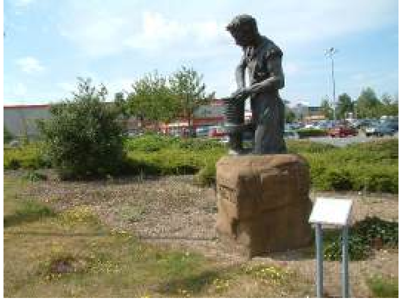

The Craftsman © Stuart Buchan (cc-by-sa/2.0)

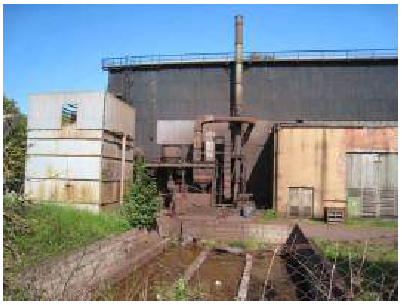

Former Wolsingham Steelworks © Oliver Dixon (cc-by-sa/2.0)

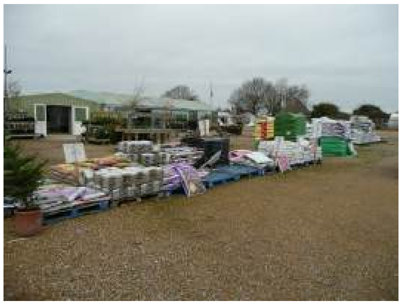

Chalcroft Nurseries © Jonathan Billinger (cc-by-sa/2.0)

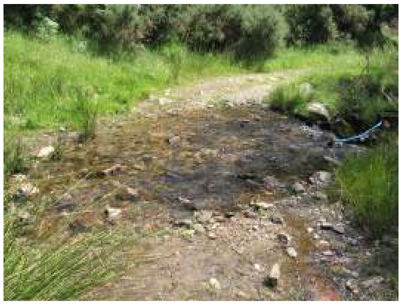

Ford in Sparcombe Water © Arjen Bax (cc-by-sa/2.0)

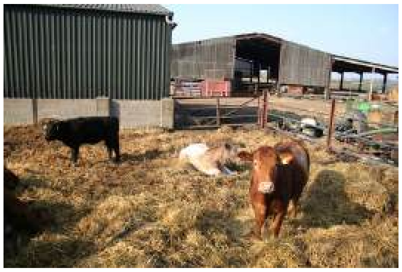

Manor farm cattle © Richard Croft (cc-by-sa/2.0)

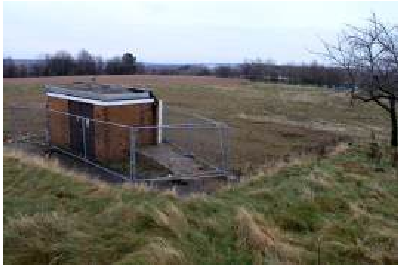

Site of St Peter's RC Secondary Modern School © David Lally (cc-by-sa/2.0)

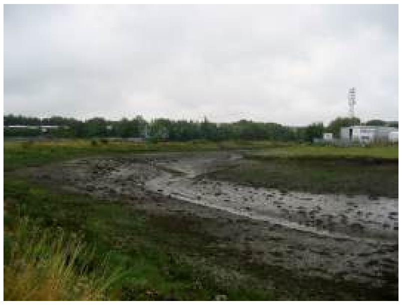

Creek in Cowpen New Town © Chris Heaton (cc-by-sa/2.0)

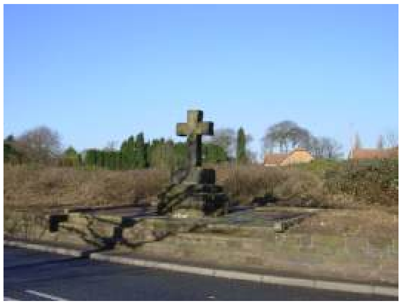

Ancient Stone Cross, Mill Lane © Sue Adair (cc-by-sa/2.0)

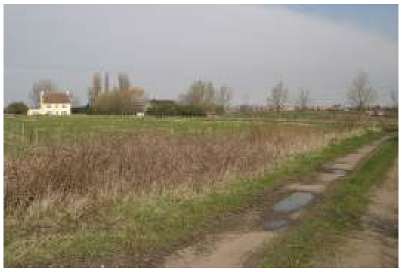

Track and farm view © Peter Jeynes (cc-by-sa/2.0)

3.136 Sport Venue

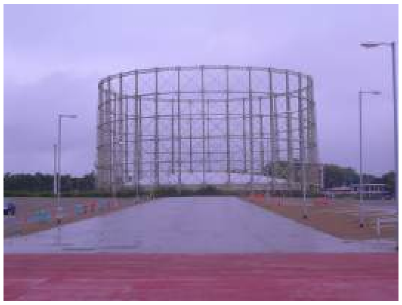

Fill her up please. © ANDY RAMMY (cc-by-sa/2.0)

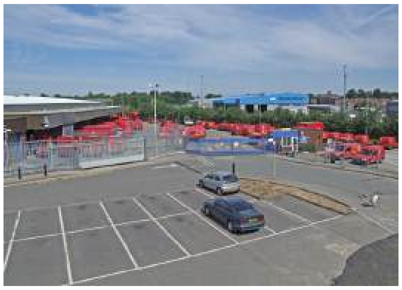

Royal Mail © Dennis Turner (cc-by-sa/2.0)

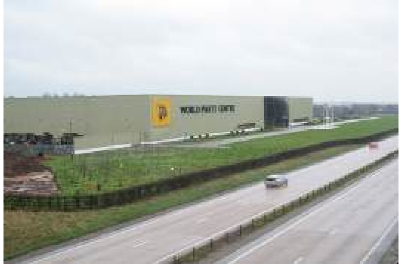

JCB World Parts Centre near Uttoxeter © Alan Murray-Rust (cc-by-sa/2.0)

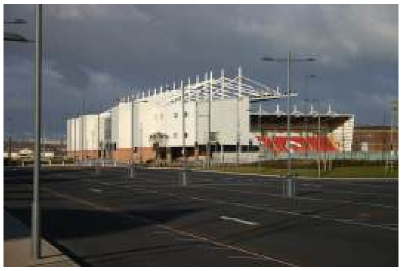

Blackpool football ground © R lee (cc-by-sa/2.0)

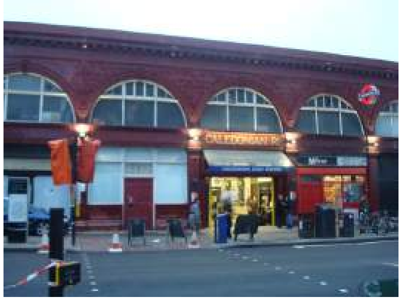

Caledonian Road Underground Station © Stacey Harris (cc-by-sa/2.0)

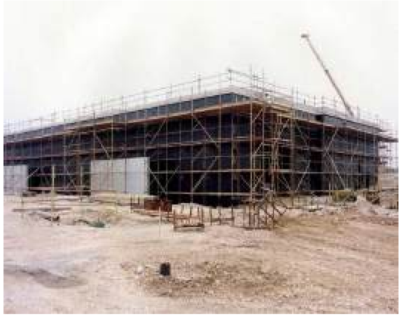

GAMA Under Construction © Jonathan Sayers (cc-by-sa/2.0)

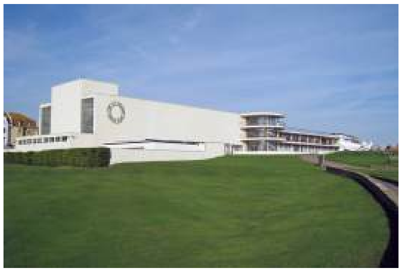

De La Warr Pavilion, Bexhill-on-Sea, East Sussex © Oast House Archive (cc-by-sa/2.0)

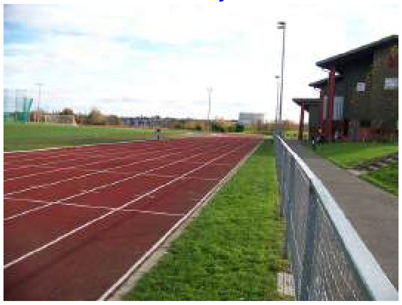

Sports facility at Canterbury High School © Elliott Simpson (cc-by-sa/2.0)

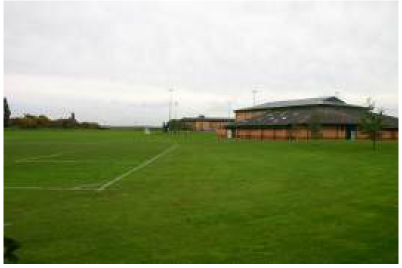

Sports Field and Sports Centre, Stafford © Stephen Pearce (cc-by-sa/2.0)

3.137 Stadium

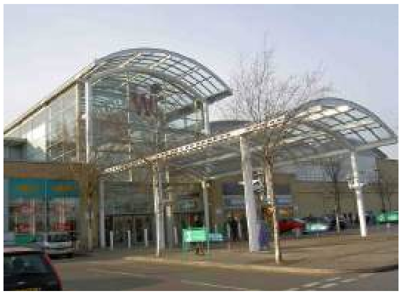
White Rose shopping mall © Steve Fareham (cc-by-sa/2.0)

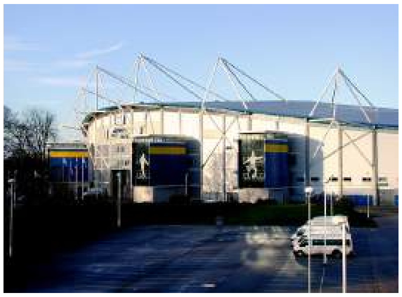
KC Stadium, Hull © Paul Glazzard (cc-by-sa/2.0)

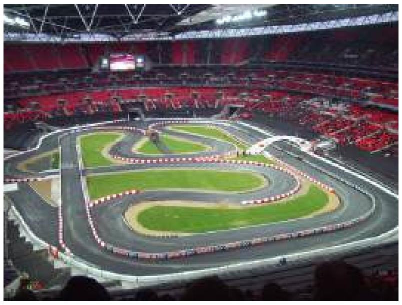
Wembley Stadium Race of Champions © Ben Ashcroft (cc-by-sa/2.0)

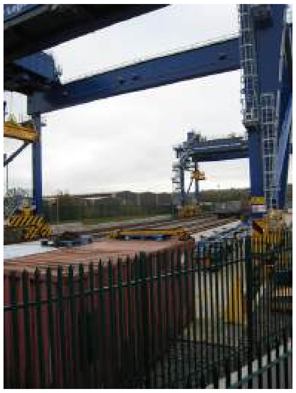
Birmingham Intermodal Freight Terminal © David Stowell (cc-by-sa/2.0)

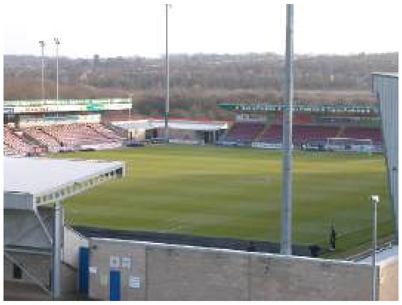
Northampton - Sixfields © Ian Rob (cc-by-sa/2.0)

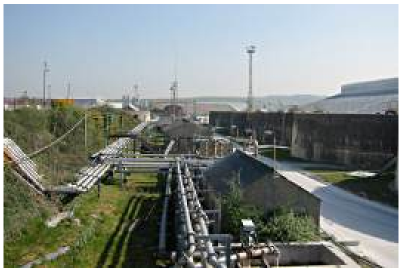
China Clay Processing Works © Tony Atkin (cc-by-sa/2.0)

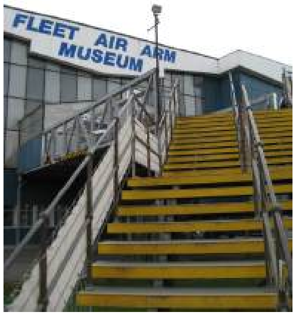

The entrance to the museum © Alison Rawson (cc-by-sa/2.0)

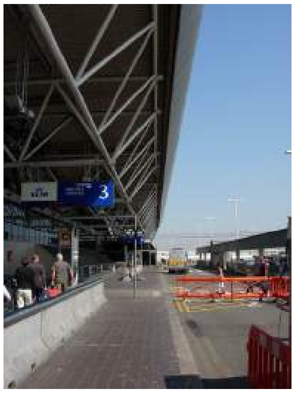
Heathrow Terminal 4 © Hugh Venables (cc-by-sa/2.0)

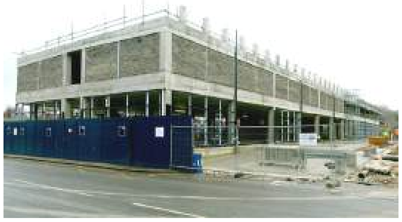
New Building under Construction © Andy Beecroft (cc-by-sa/2.0)

3.138 Street

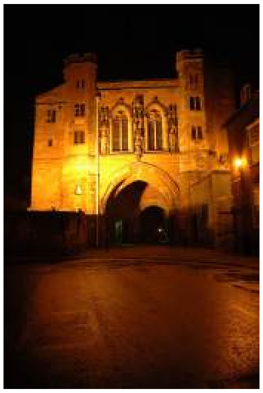

Floodlit Edgar Tower © Philip Halling (cc-by-sa/2.0)

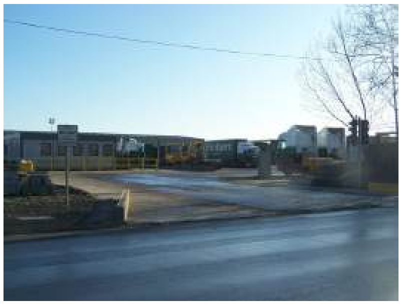

Eddie Stobart Shunt Works © David Anstiss (cc-by-sa/2.0)

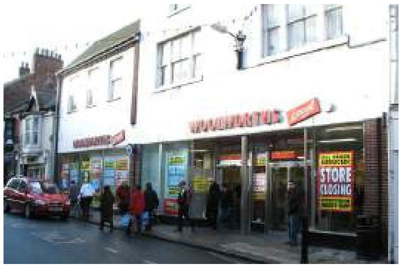

Nearing the End © Gordon Hatton (cc-by-sa/2.0)

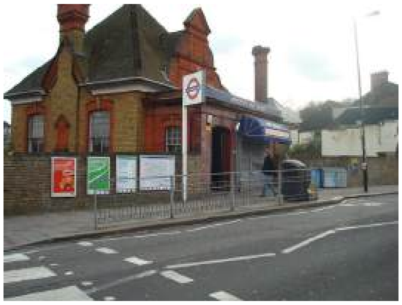

Wimbledon Park Underground Station © Stacey Harris (cc-by-sa/2.0)

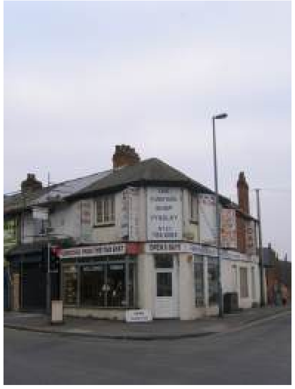

Midland Bank Tyseley Sub Office, 40-11-01 © Roy Hughes (cc-by-sa/2.0)

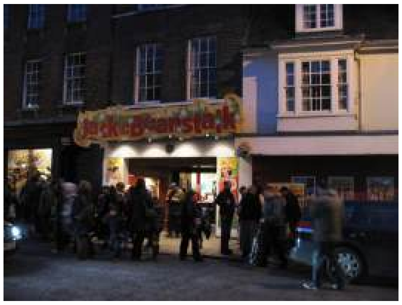

Cambridge panto season © Mr Ignavy (cc-by-sa/2.0)

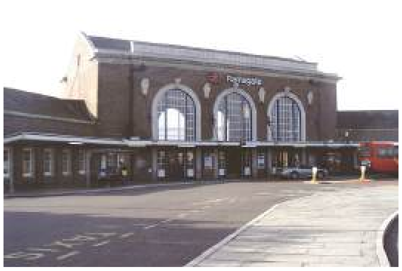

Ramsgate station © David Kemp (cc-by-sa/2.0)

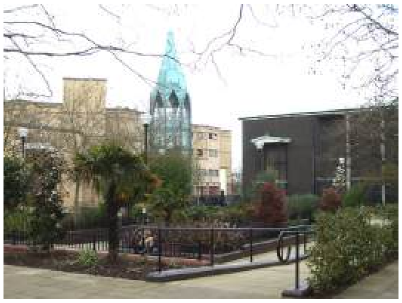

Church Garden © terry joyce (cc-by-sa/2.0)

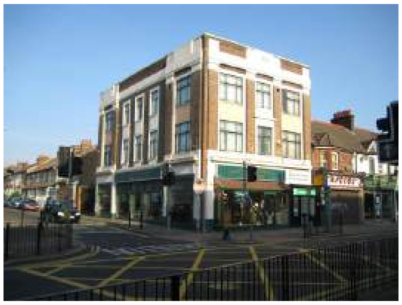

Watford: St Albans Road © Nigel Cox (cc-by-sa/2.0)

3.139 Street Light

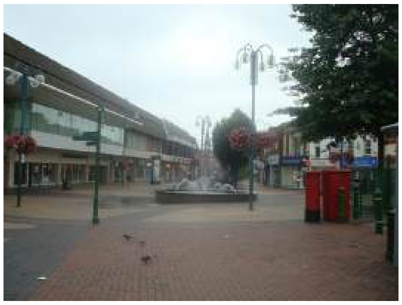

Broadway, Bexleyheath © Stacey Harris (cc-by-sa/2.0)

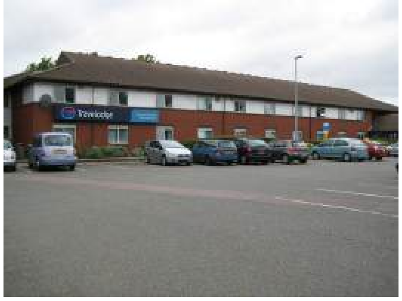

M4 Motorway: Heston Services: Travelodge © Nigel Cox (cc-by-sa/2.0)

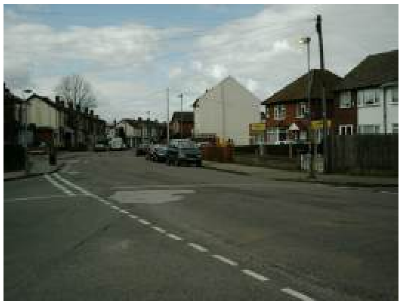

St Margarets Road © Carl Baker (cc-by-sa/2.0)

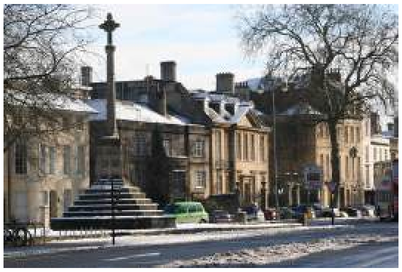

St Giles © Chris Denny (cc-by-sa/2.0)

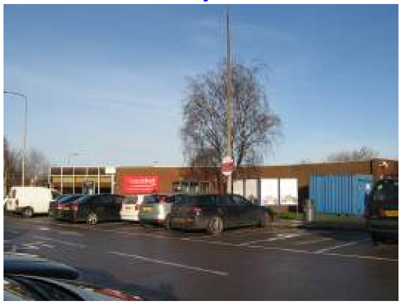

M6 - Sandbach Services northbound © Peter Whatley (cc-by-sa/2.0)

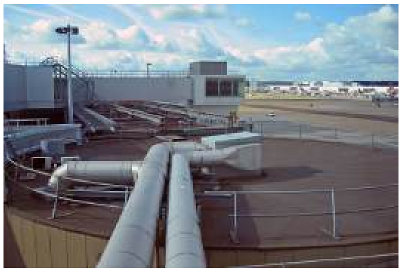

Gatwick South Terminal © John Allan (cc-by-sa/2.0)

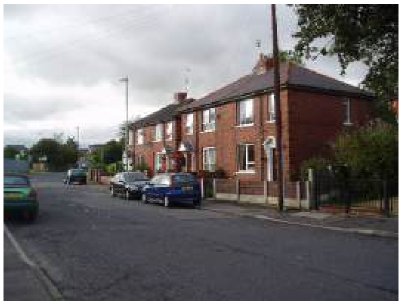

Former Local Authority houses, Ings Avenue, Rochdale, Lancashire © Dr Neil Clifton (cc-by-sa/2.0)

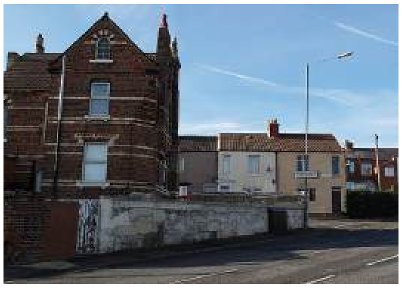

Older part of New Skelton © Stephen McCulloch (cc-by-sa/2.0)

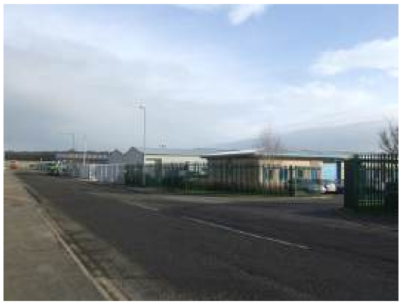

Industrial units on the outskirts of Gainsborough © Jonathan Billinger (cc-by-sa/2.0)

3.140 Suburb

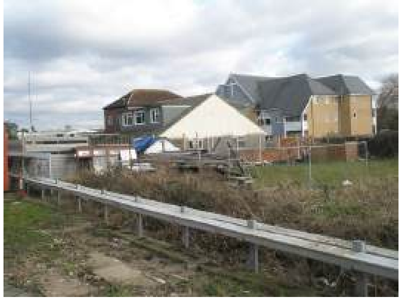

Looking from Bedhampton Railway Station towards Palk Road © Basher Eyre (cc-by-sa/2.0)

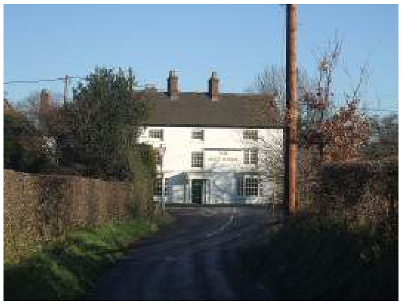

The Malt House from Cliff Hall Lane © Rob Farrow (cc-by-sa/2.0)

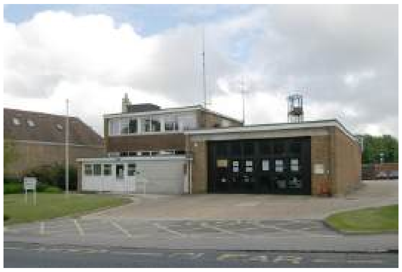

Crowborough fire station © Kevin Hale (cc-by-sa/2.0)

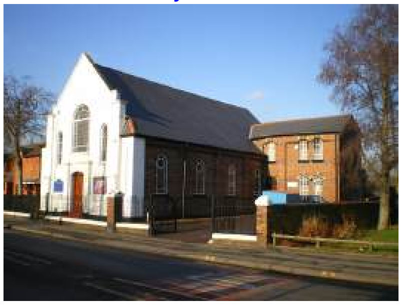

The Bethel United Church of Jesus Christ Apostolic © Richard Law (cc-by-sa/2.0)

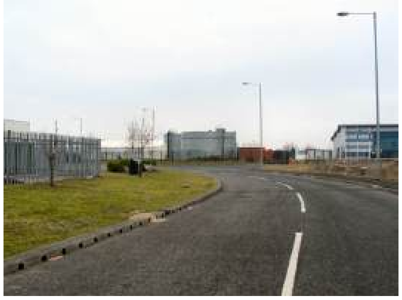

Deeside Industrial Area © Roger May (cc-by-sa/2.0)

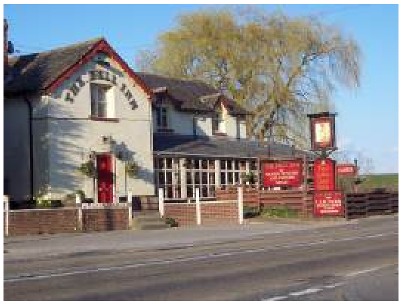

The Bell Inn, Winterbourne Stoke © Maigheach-gheal (cc-by-sa/2.0)

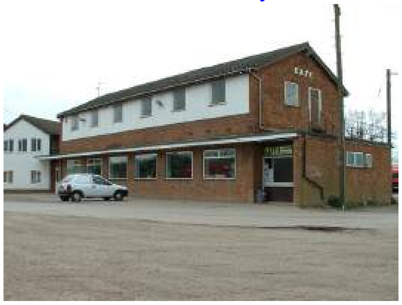

Kates Cafe © Keith Evans (cc-by-sa/2.0)

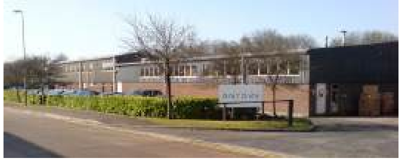

Industrial units on Wembley Road © Keith Williams (cc-by-sa/2.0)

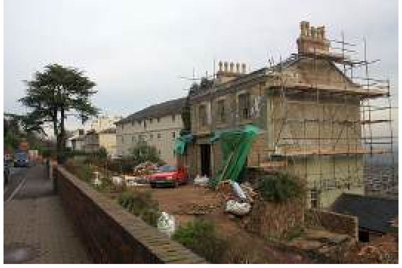

Renovation work, Worcester Road, Malvern © Bob Embleton (cc-by-sa/2.0)

3.141 Sunlight

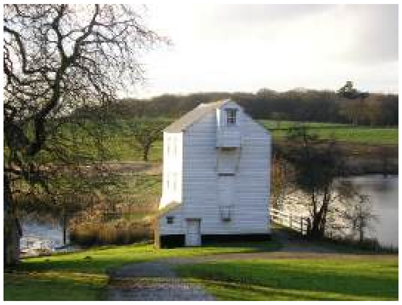

Thorrington Tide Mill © Roger W Haworth (cc-by-sa/2.0)

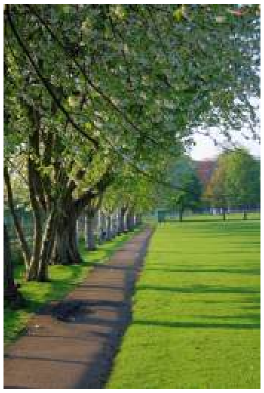

Footpath Around Playing Fields © Mick Garratt (cc-by-sa/2.0)

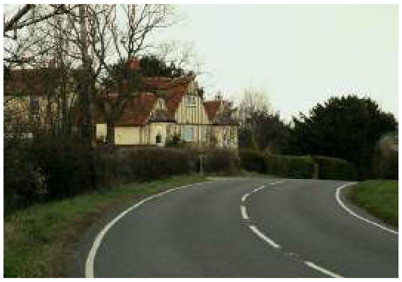

The farmhouse at Brownings Farm © Robert Edwards (cc-by-sa/2.0)

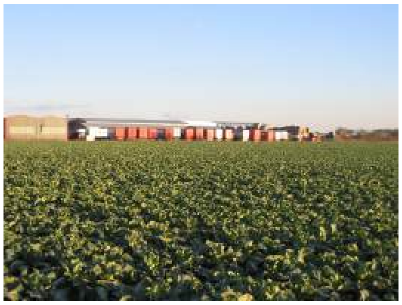

Haulage Depot, Blackjack, Swineshead © Rodney Burton (cc-by-sa/2.0)

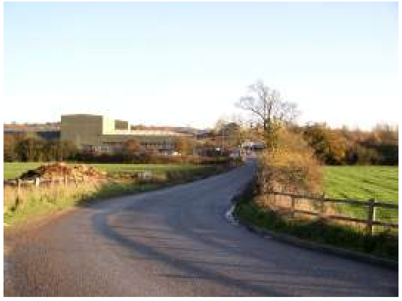

Industrial site, Calne © Chris Henley (cc-by-sa/2.0)

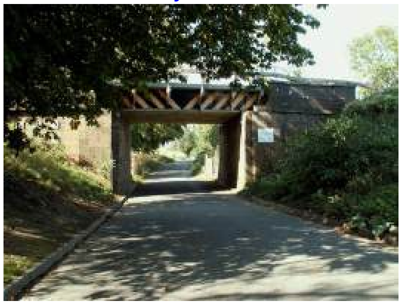

Railway bridge at Hatfield Peverel, Essex © Robert Edwards (cc-by-sa/2.0)

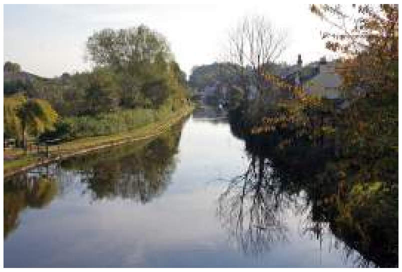

Erewash Canal looking south from Sandiacre bridge © David Pinney (cc-by-sa/2.0)

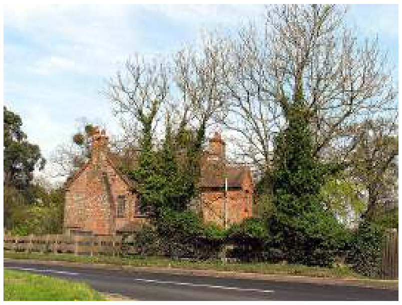

Paices Hill © Pam Brophy (cc-by-sa/2.0)

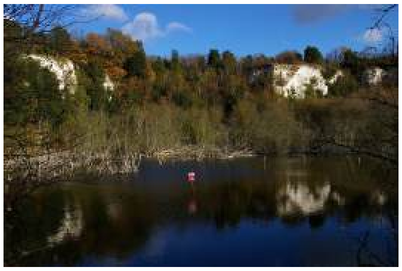

Pond in the Pit © Glyn Baker (cc-by-sa/2.0)

3.142 Tar

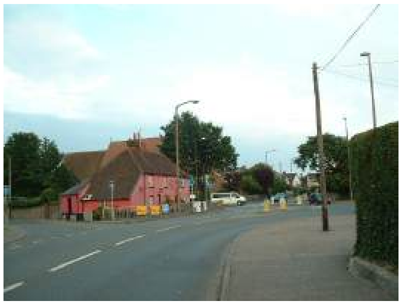

Gateway to Bersted © Chris Shaw (cc-by-sa/2.0)

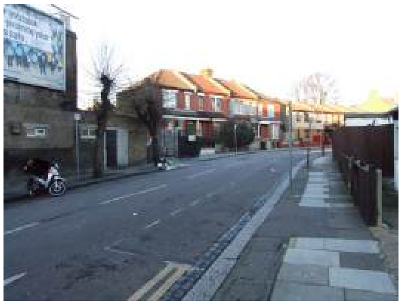

Vincent Road, West Green © Chris Whippet (cc-by-sa/2.0)

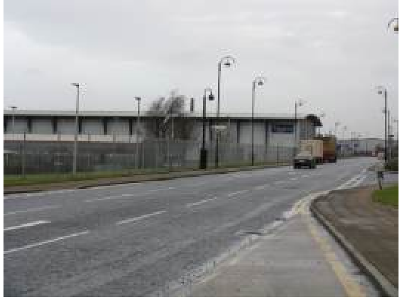

Trafford Park - Third Avenue, looking north © Peter Whatley (cc-by-sa/2.0)

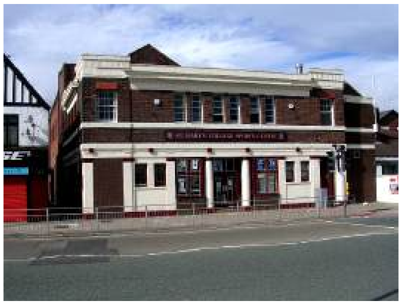

St. Mary's College Sports Centre © Robert Brown (cc-by-sa/2.0)

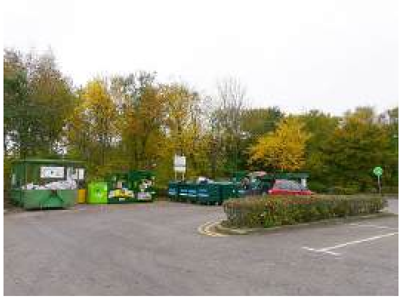

Recycling point © Rose and Trev Clough (cc-by-sa/2.0)

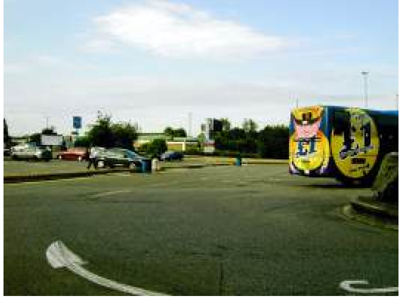

Car & coach parking at Watford Gap M1 Services © Slbs (cc-by-sa/2.0)

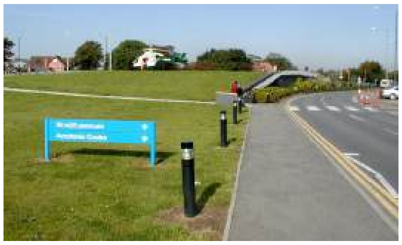

Great North Air Ambulance © Mick Garratt (cc-by-sa/2.0)

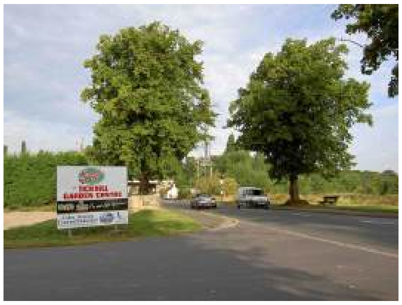

Entrance to Cherry Lane garden centre © Steve Fareham (cc-by-sa/2.0)

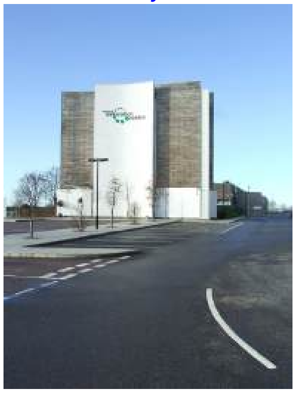

Keystone Innovation Centre © Keith Evans (cc-by-sa/2.0)

3.143 Thoroughfare

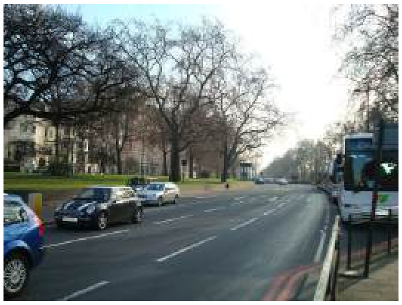

Park Lane, London W1K © Stacey Harris (cc-by-sa/2.0)

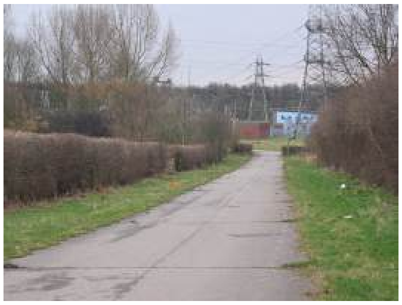

Electricity sub-station, Yew Tree Estate, Walsall. © Frank Smith (cc-by-sa/2.0)

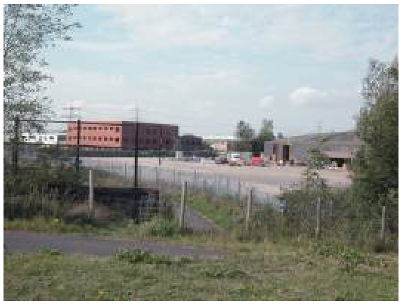

Industrial Estate Deeside © Dennis Turner (cc-by-sa/2.0)

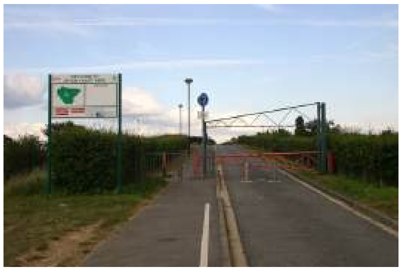

Entrance to Upton Court park © Shaun Ferguson (cc-by-sa/2.0)

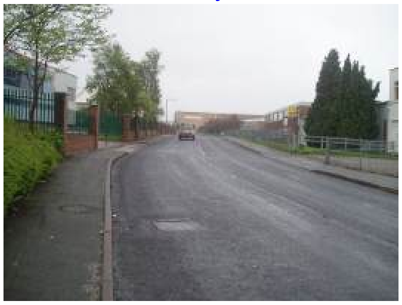

Codnor Gate Industrial Estate © Geoff Dunn (cc-by-sa/2.0)

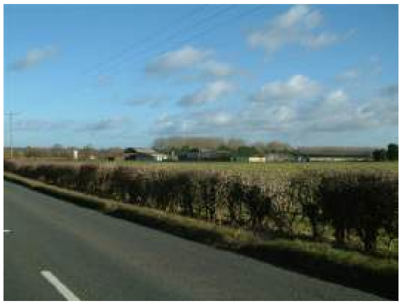

Farmland at Garford © Colin Bates (cc-by-sa/2.0)

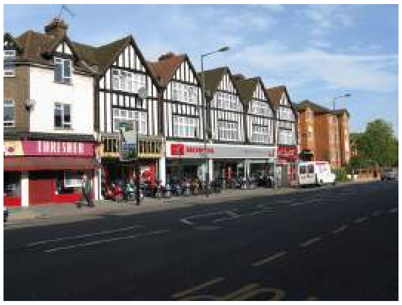

Doble Motorcycles, Brighton Road, Coulsdon © Dr Neil Clifton (cc-by-sa/2.0)

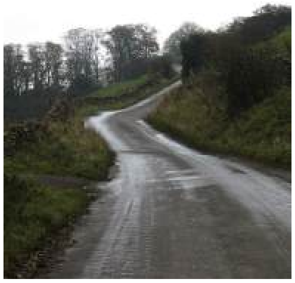

Witton Steeps © Gordon Hatton (cc-by-sa/2.0)

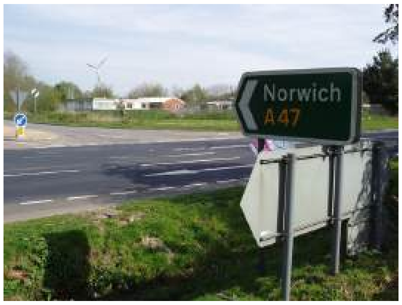

Norwich this way! © David Williams (cc-by-sa/2.0)

3.144 Tints And Shades

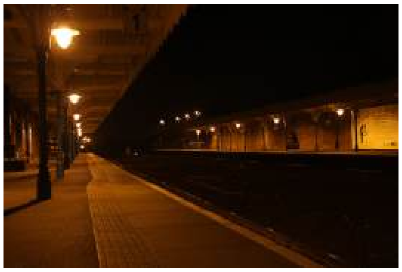

Bury St Edmunds Station © Hugh Venables (cc-by-sa/2.0)

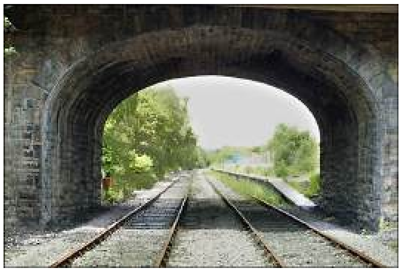

Site of the former Heathfield Station © Mike Crowe (cc-by-sa/2.0)

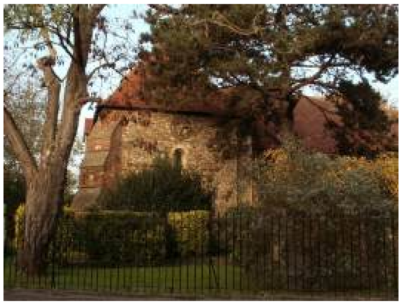

St. Andrew's church, Heybridge, Essex © Robert Edwards (cc-by-sa/2.0)

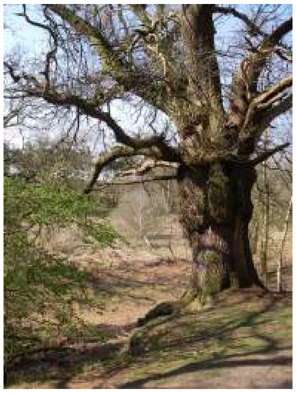

Veteran Oak - Rendlesham Forest © Simon Leatherdale (cc-by-sa/2.0)

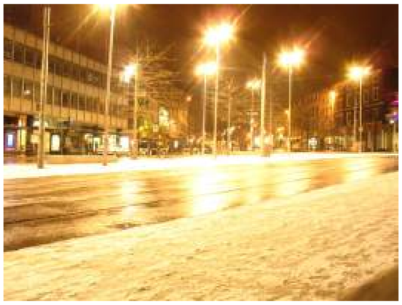

Snow in Nottingham 9.50ish pm © Andy Jamieson (cc-by-sa/2.0)

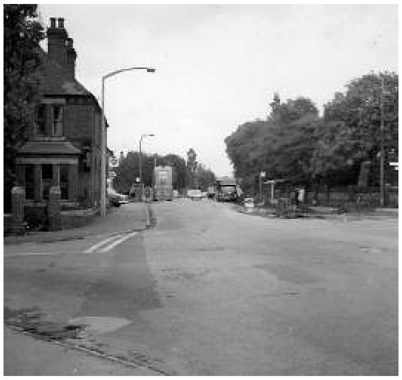

Holly Hall © Brian Clift (cc-by-sa/2.0)

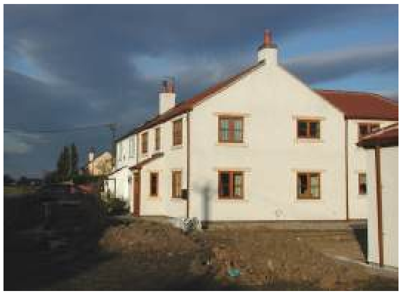

Hagg Lane Farm, Hemingbrough © Paul Glazzard (cc-by-sa/2.0)

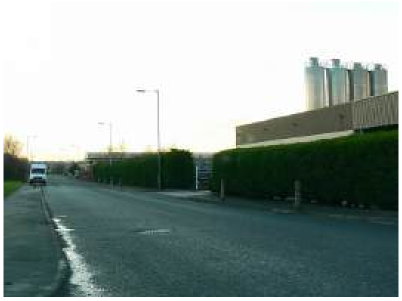

Deceuninck Ltd, Stanier Road, Porte Marsh Industrial Estate, Calne © Brian Robert Marshall (cc-by-sa/2.0)

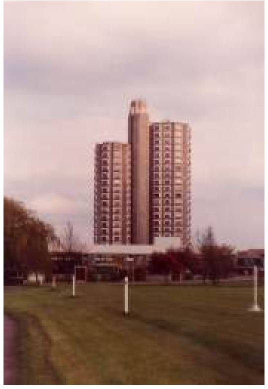

Towers, Loughborough University © Mike Pennington (cc-by-sa/2.0)

3.145 Tire

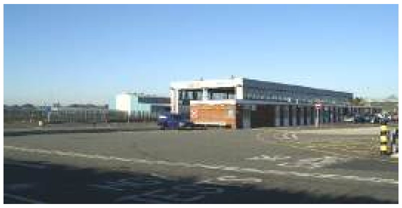

Kirkham Goods Vehicle Testing Station © David Long (cc-by-sa/2.0)

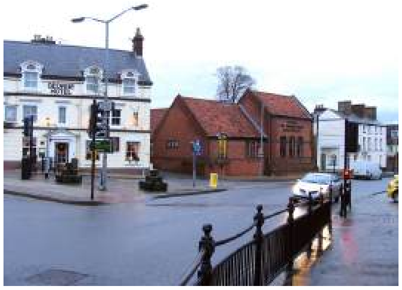

George Hotel, Mangate Street, Swaffham © John Salmon (cc-by-sa/2.0)

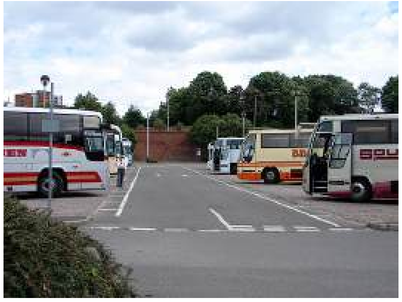

Northampton Railway Station © John Lucas (cc-by-sa/2.0)

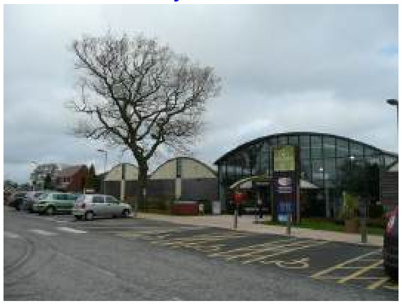

Blooms Garden Centre, Rugby © Jonathan Billinger (cc-by-sa/2.0)

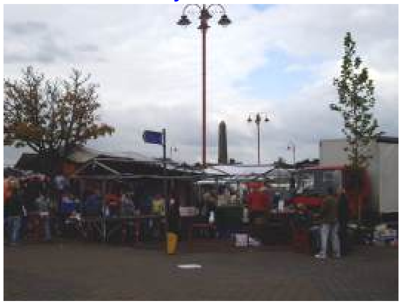

Street Market, Newton le Willows © Tom Pennington (cc-by-sa/2.0)

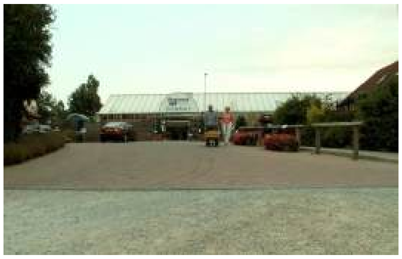

Perrywood Nurseries, Inworth, Essex © Robert Edwards (cc-by-sa/2.0)

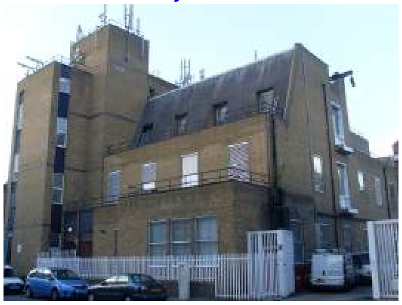

Walworth Telephone Exchange © David Hillas (cc-by-sa/2.0)

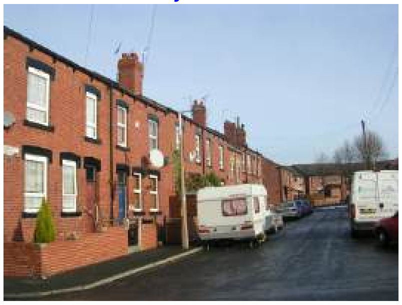

Tilbury Avenue - Tilbury Road © Betty Longbottom (cc-by-sa/2.0)

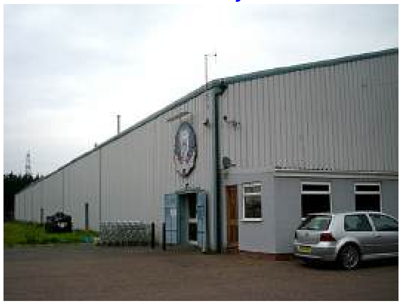

Beer warehouse © Row17 (cc-by-sa/2.0)

3.146 Tower

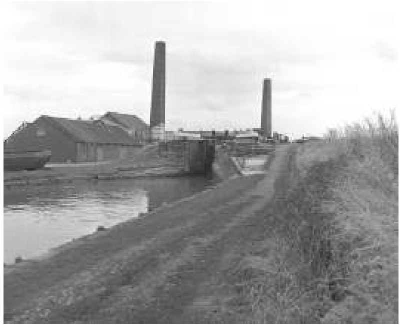

The tail of Lock No 73, Trent and Mersey Canal © Dr Neil Clifton (cc-by-sa/2.0)

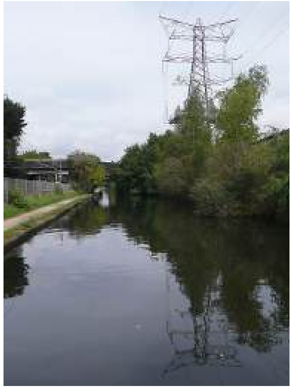

Worcester and Birmingham Canal south of Bournville © Roger D Kidd (cc-by-sa/2.0)

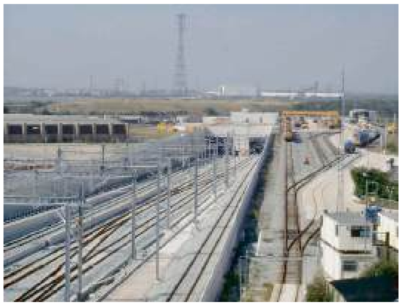

Thames Tunnel © Glyn Baker (cc-by-sa/2.0)

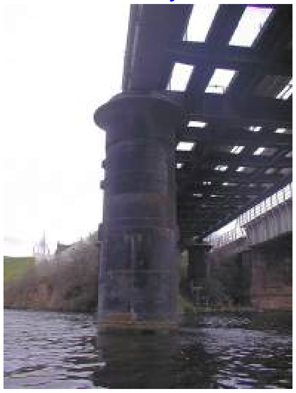

Pier of Disused Bridge © Mick Garratt (cc-by-sa/2.0)

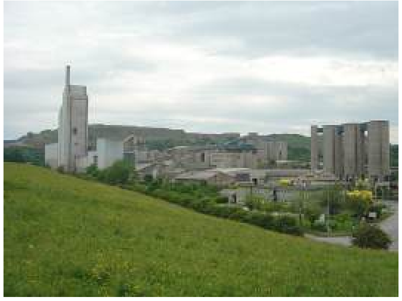

Cauldon Cement Works © Alan Murray-Rust (cc-by-sa/2.0)

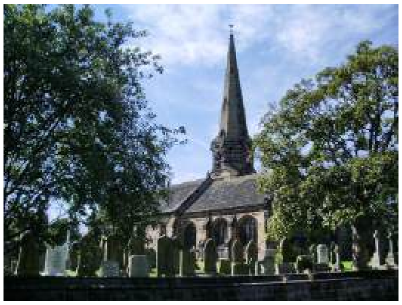

St Michael, Aughton Parish Church © Alexander P Kapp (cc-by-sa/2.0)

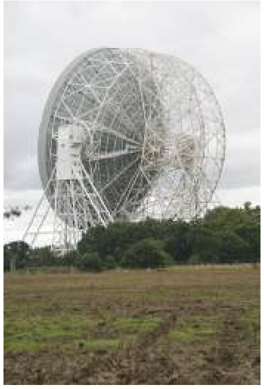

Jodrell Bank Radio telescope © Richard Styles (cc-by-sa/2.0)

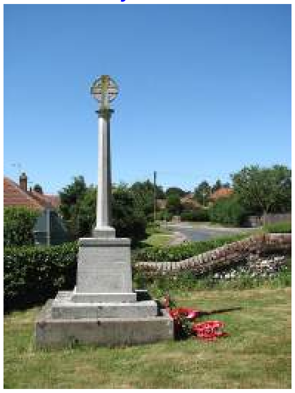

St Andrew's church - war memorial © Evelyn Simak (cc-by-sa/2.0)

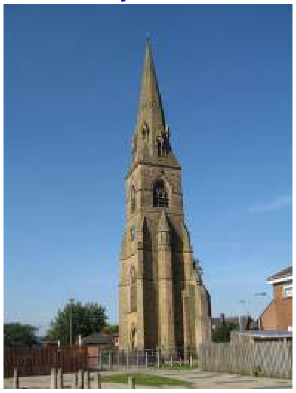

St George, Charlestown © Sue Adair (cc-by-sa/2.0)

3.147 Tower Block

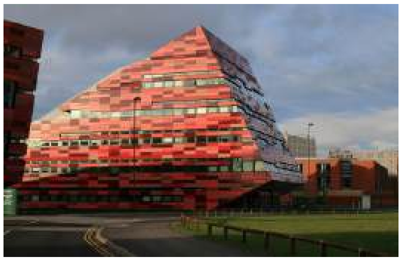

International House © Martin Jones (cc-by-sa/2.0)

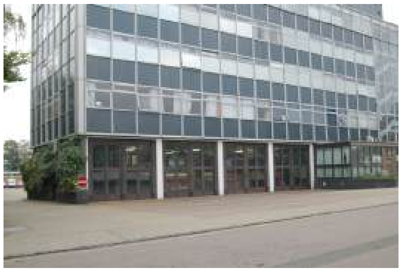

Hertford fire station © Kevin Hale (cc-by-sa/2.0)

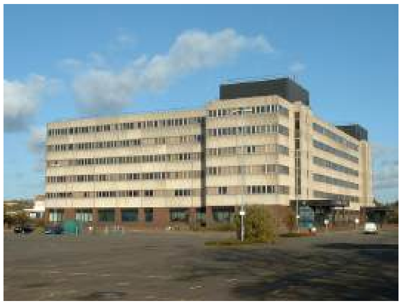

Anzani House © Keith Evans (cc-by-sa/2.0)

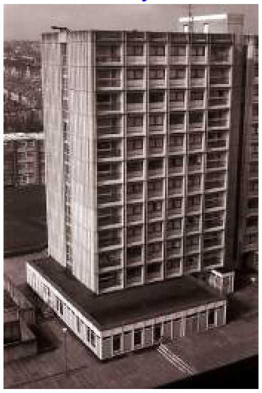

Flats in Coopersale Close © Geographer (cc-by-sa/2.0)

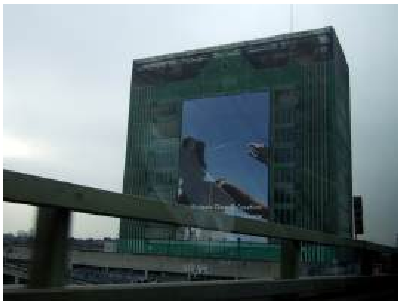

2009 and the Alfa Laval building is still empty © Natasha Ceridwen de Chroustchoff (cc-by-sa/2.0)

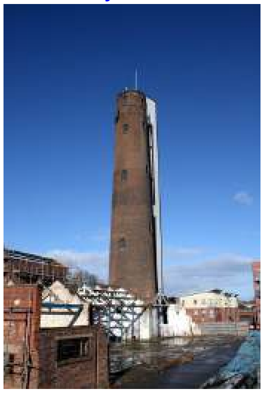

Lead Shot Tower, Boughton, Chester © Jeff Buck (cc-by-sa/2.0)

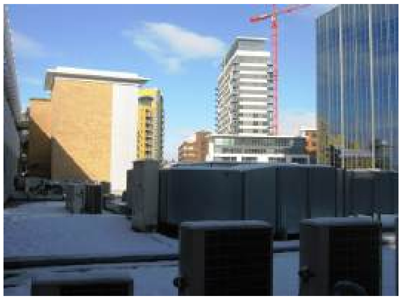

City centre from the car park © Mr Ignavy (cc-by-sa/2.0)

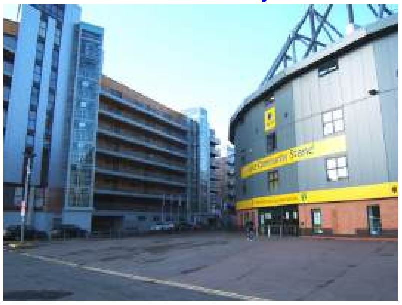

Norwich City FC, Carrow Road © Martin Thirkettle (cc-by-sa/2.0)

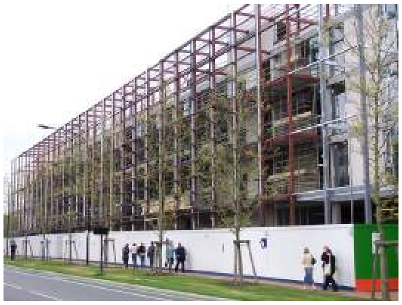

University of Southampton, Building 32 under construction © David Martin (cc-by-sa/2.0)

3.148 Trail

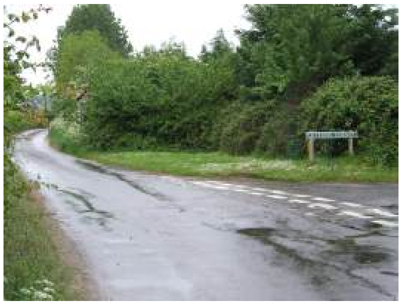

Cuttons Corner, Hemblington Hall Road © Ian Robertson (cc-by-sa/2.0)

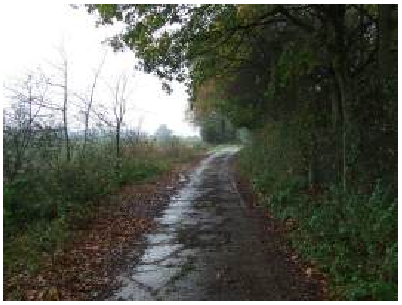

Former closed footpath © Keith Evans (cc-by-sa/2.0)

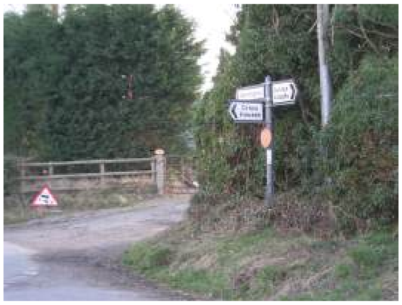

Lane junction near Berrington. © Row17 (cc-by-sa/2.0)

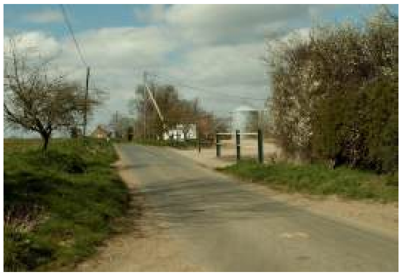

Part of Church Lane at Little Leighs © Robert Edwards (cc-by-sa/2.0)

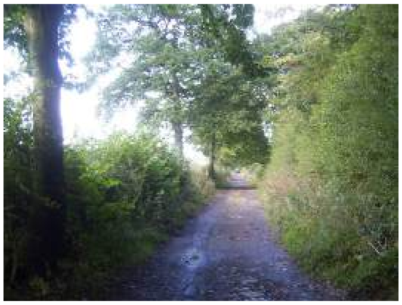

The Lymes Road © Iain McDonald (cc-by-sa/2.0)

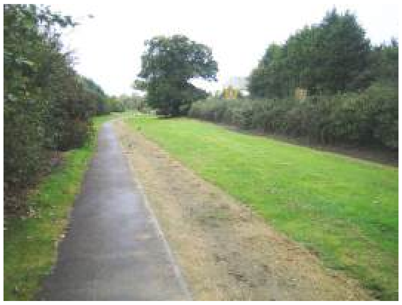

South Marston: Thornhill Road bridleway and footpath © Nigel Cox (cc-by-sa/2.0)

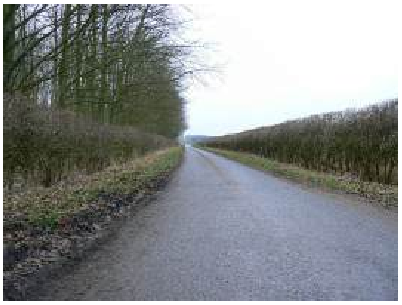

The road to Down Ampney © Brian Robert Marshall (cc-by-sa/2.0)

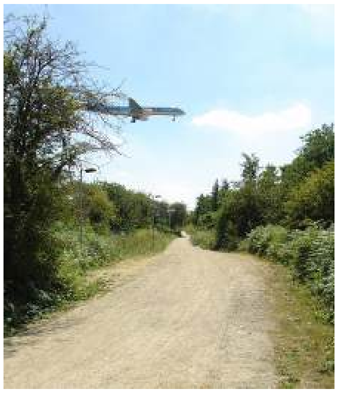

Cycle Route past Gatwick Airport, Near Crawley, West Sussex © Pete Chapman (cc-by-sa/2.0)

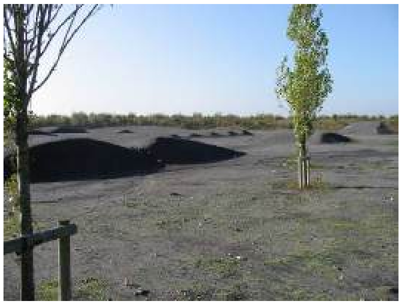

Part of the BMX track at Fowlmead © Nick Smith (cc-by-sa/2.0)

3.149 Train

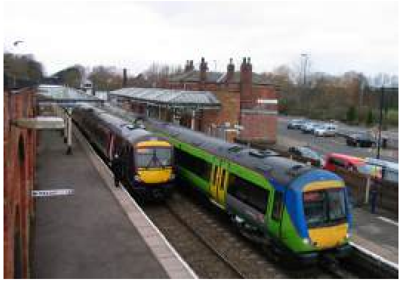

Trains to Stansted and Birmingham © Andrew Tatlow (cc-by-sa/2.0)

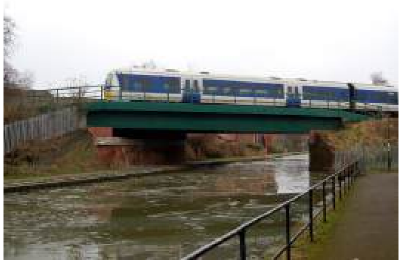

Canal railway bridge, Leamington © Andy F (cc-by-sa/2.0)

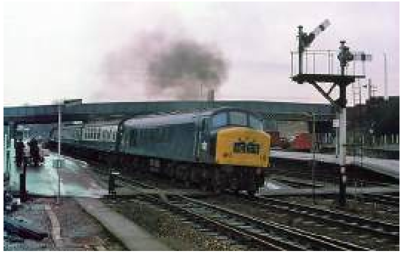

St. Pancras Bound © Martin Addison (cc-by-sa/2.0)

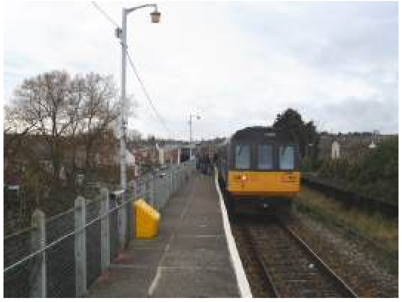

Polsloe Bridge Halt © Roger Cornfoot (cc-by-sa/2.0)

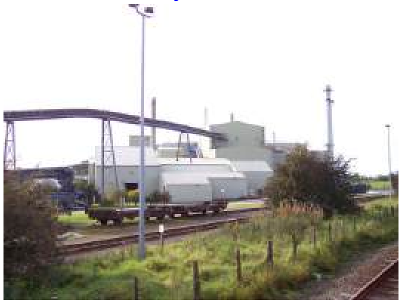

Kirkby Thore Gypsum plant © Raymond Knapman (cc-by-sa/2.0)

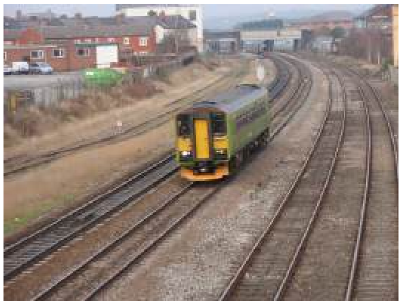

East Midlands train service to Crewe © James Haynes (cc-by-sa/2.0)

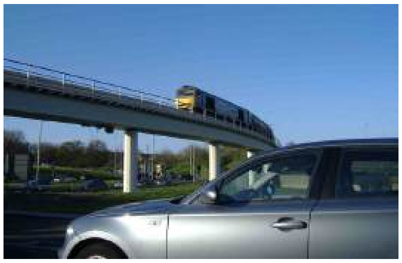

Road and rail © Roger Cornfoot (cc-by-sa/2.0)

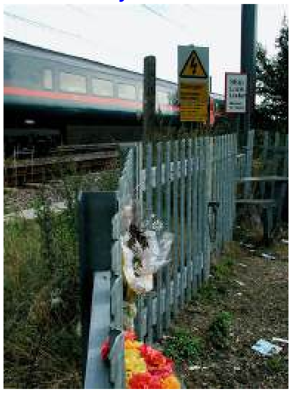

A public right of way © Paul Glazzard (cc-by-sa/2.0)

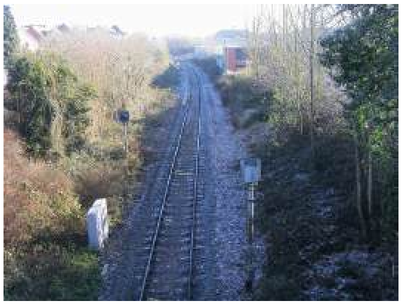

Single track towards the Cowley Works © Shaun Ferguson (cc-by-sa/2.0)

3.150 Train Station

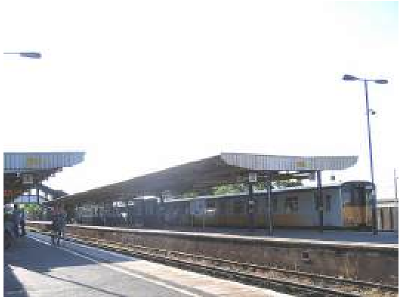

Sittingbourne station, west end © Stephen Craven (cc-by-sa/2.0)

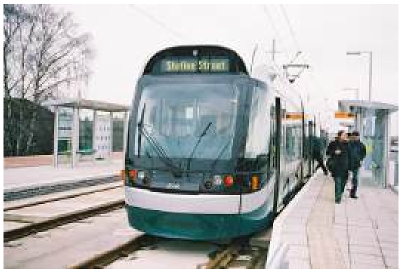

Tram for Nottingham at Hucknall station © Dr Neil Clifton (cc-by-sa/2.0)

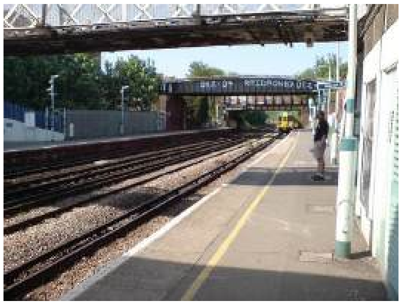

Platform 2, Brockley Station © Rich Tea (cc-by-sa/2.0)

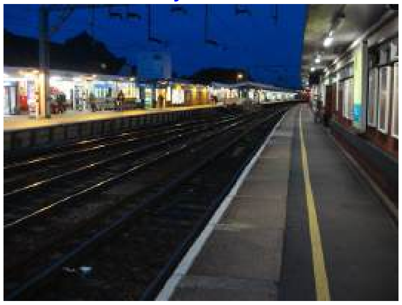

Colchester Station, platforms © Oxyman (cc-by-sa/2.0)

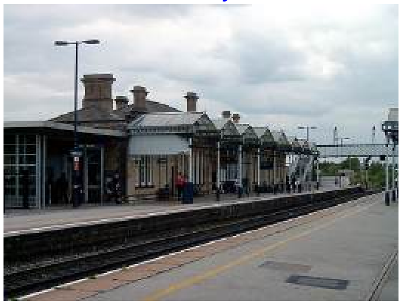

Loughborough Midland Station © Gordon Cragg (cc-by-sa/2.0)

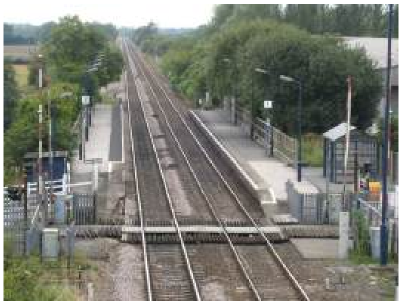

Sherburn-in-Elmet station © John Armitstead (cc-by-sa/2.0)

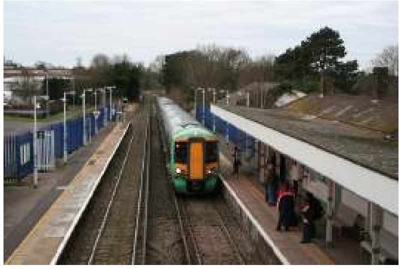

Ewell East station © Dr Neil Clifton (cc-by-sa/2.0)

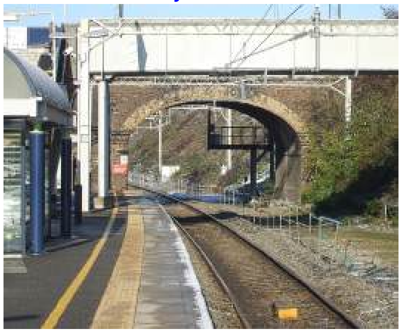

Tring Station - Station Road Bridge, Eastern Arch © Rob Farrow (cc-by-sa/2.0)

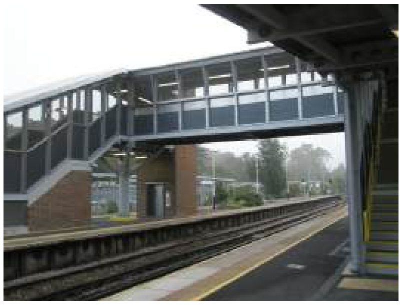

The new bridge at Haslemere Station © Basher Eyre (cc-by-sa/2.0)

3.151 Transmission Tower

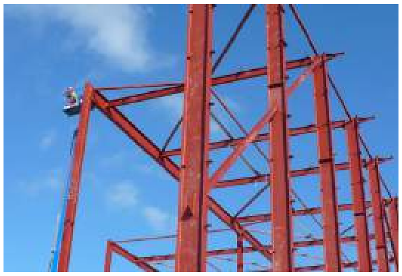
Erection of steel Frame Building, Yeovil (3) © Nigel Mykura (cc-by-sa/2.0)

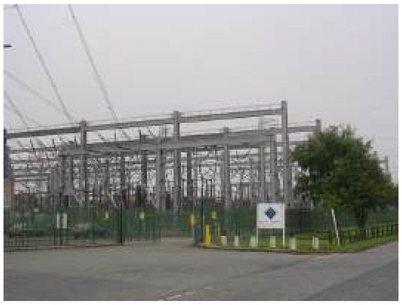
It's all about Power! © Keith Williamson (cc-by-sa/2.0)

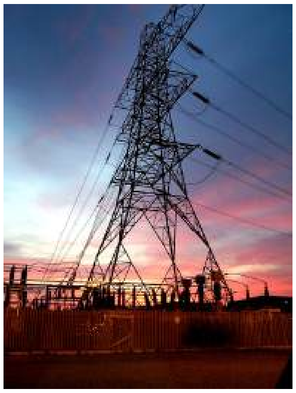
Morning glory © R lee (cc-by-sa/2.0)

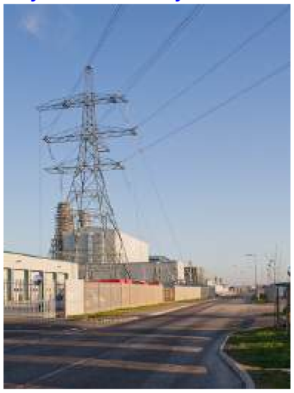
East Road, Marchwood Industrial Park © Peter Facey (cc-by-sa/2.0)

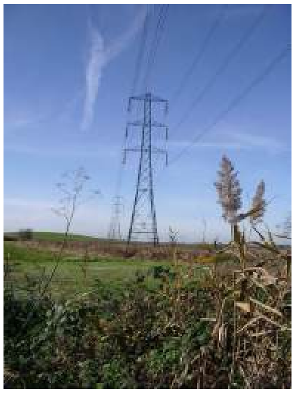
Pylons off Seasalter Lane © pam fray (cc-by-sa/2.0)

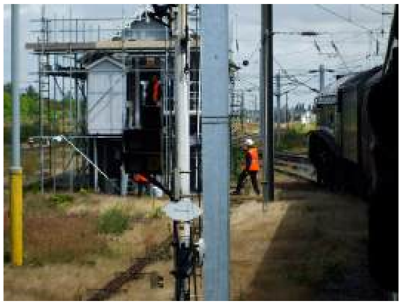
Steam locomotive and signal box © Ashley Dace (cc-by-sa/2.0)

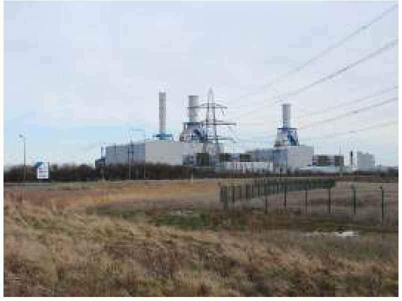
South Humber Power Station © Ian Paterson (cc-by-sa/2.0)

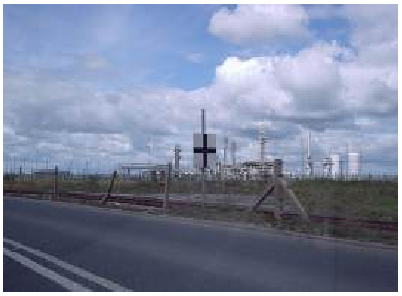
Industrial © Stephen McCulloch (cc-by-sa/2.0)

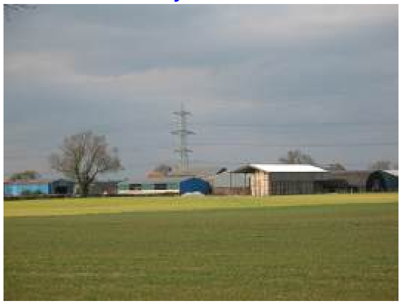
Forest Hall Farm near Alne © Gordon Hatton (cc-by-sa/2.0)

3.152 Transport Hub

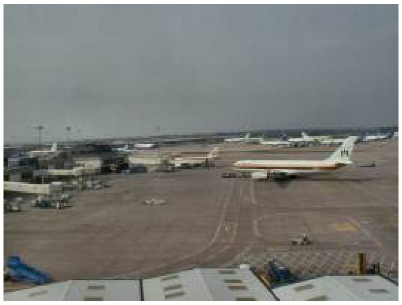

Plane Spotting © Gerald England (cc-by-sa/2.0)

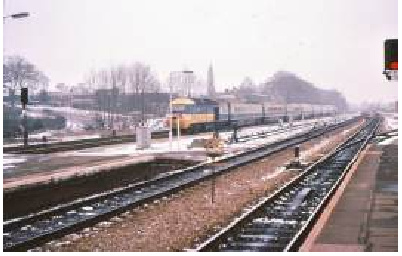

Up fast line HST at Maidenhead © Peter Whatley (cc-by-sa/2.0)

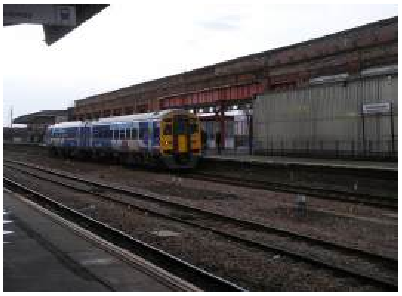

Wakefield Kirkgate © SMJ (cc-by-sa/2.0)

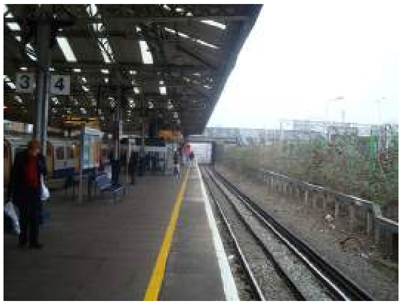

Queens Park Railway Station © Stacey Harris (cc-by-sa/2.0)

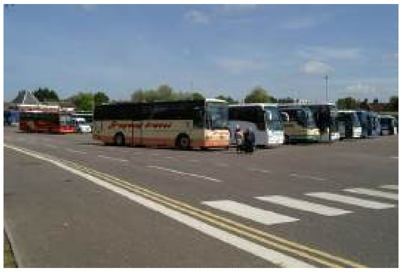

Canterbury coach park, Kingsmead © David Long (cc-by-sa/2.0)

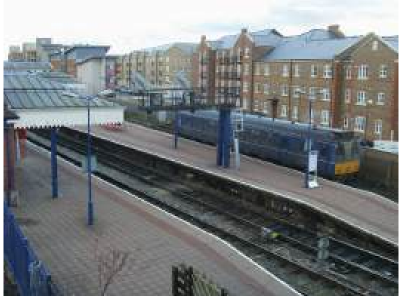

Aylesbury Railway Station © Shaun Ferguson (cc-by-sa/2.0)

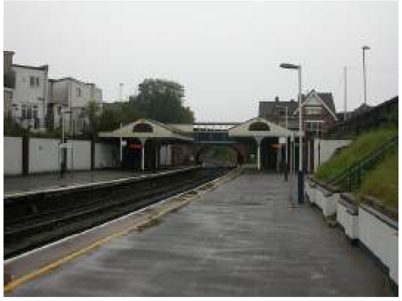

Branksome Station © Mike Faherty (cc-by-sa/2.0)

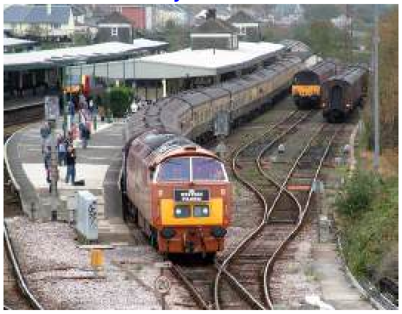

Saturday afternoon at Plymouth station © roger geach (cc-by-sa/2.0)

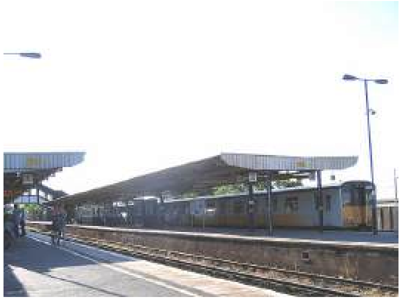

Sittingbourne station, west end © Stephen Craven (cc-by-sa/2.0)

3.153 Travel

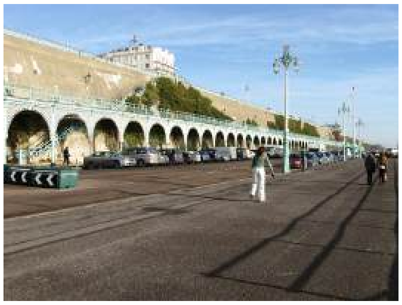

Madeira Drive © Simon Carey (cc-by-sa/2.0)

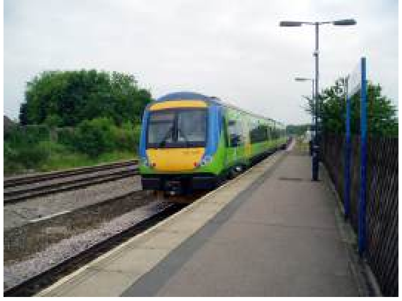

Syston station © Dr Neil Clifton (cc-by-sa/2.0)

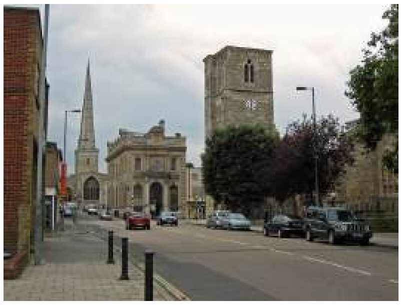

Holyrood Church, Southampton © southpix (cc-by-sa/2.0)

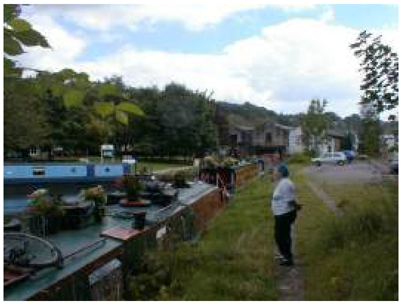

Whaley Bridge © Gerald England (cc-by-sa/2.0)

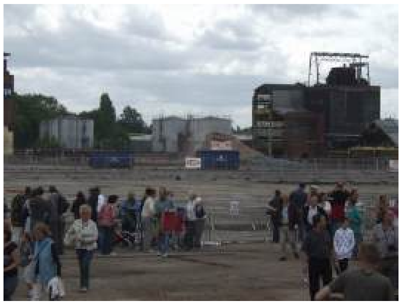

After the dust had settled © John M (cc-by-sa/2.0)

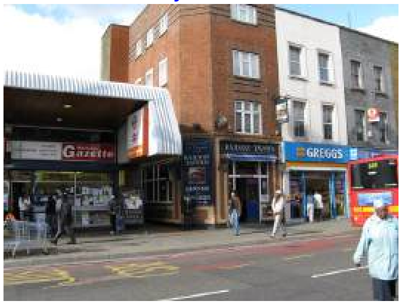

The 'Railway Tavern', Dalston © Dr Neil Clifton (cc-by-sa/2.0)

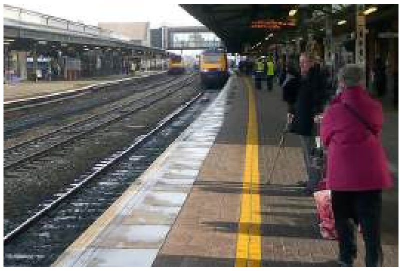

Reading station © Graham Horn (cc-by-sa/2.0)

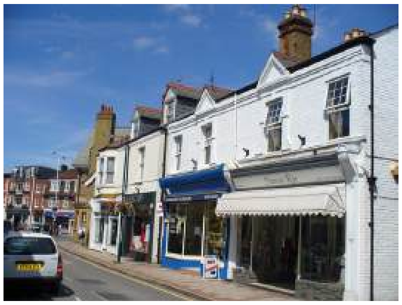

Baker Street © Colin Smith (cc-by-sa/2.0)

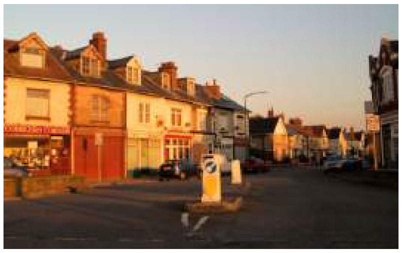

'Cobblers Corner' © Steve Fareham (cc-by-sa/2.0)

3.154 Tree

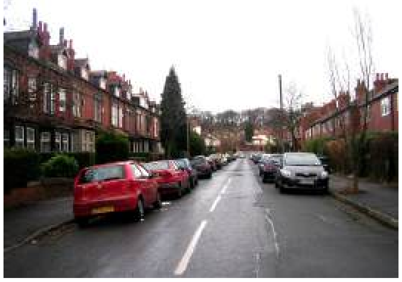

Ash Road - Kirkstall Lane © Betty Longbottom (cc-by-sa/2.0)

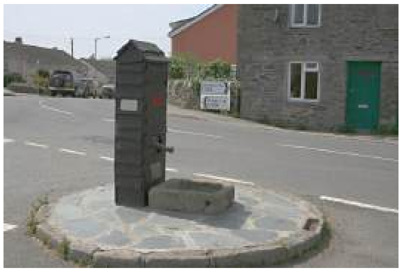

The Village Pump © Tony Atkin (cc-by-sa/2.0)

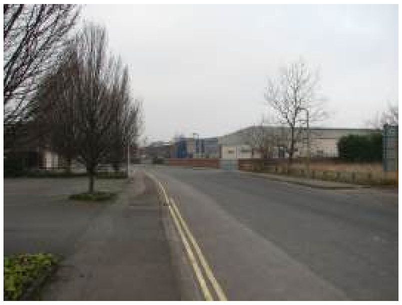

Back down the mead © Bill Nicholls (cc-by-sa/2.0)

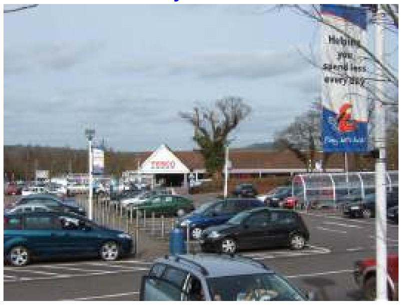

Tesco, Battishorne Way, Honiton © Tony Tooley (cc-by-sa/2.0)

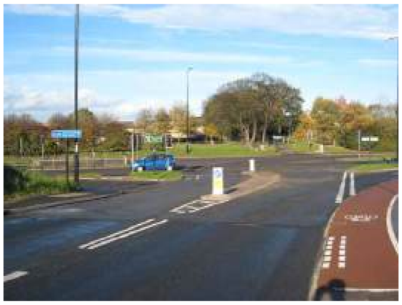

Junction on A689, Hartlepool © Oliver Dixon (cc-by-sa/2.0)

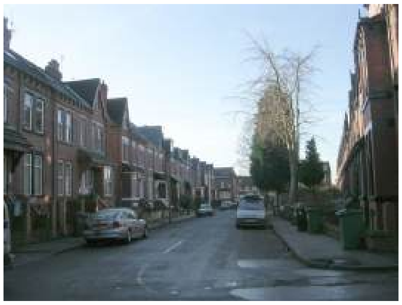

Brookfield Avenue - Harehills Avenue © Betty Longbottom (cc-by-sa/2.0)

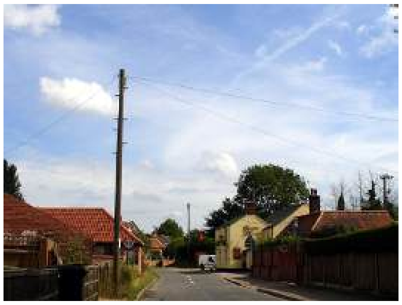

Thurlton village © Linda Bailey (cc-by-sa/2.0)

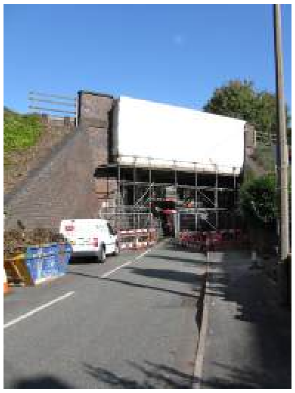

Beauty Bank Bridge under repair © Peter Whatley (cc-by-sa/2.0)

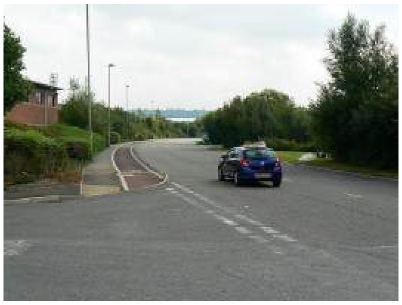

Waterwells Drive, near Hardwicke, Gloucestershire © Brian Robert Marshall (cc-by-sa/2.0)

3.155 Truck

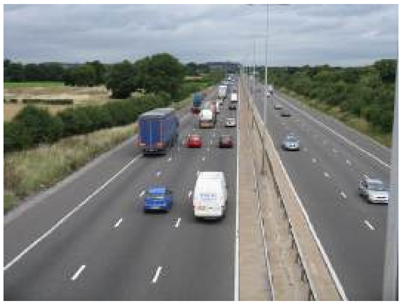

Green Street - the M5 motorway © Peter Whatley (cc-by-sa/2.0)

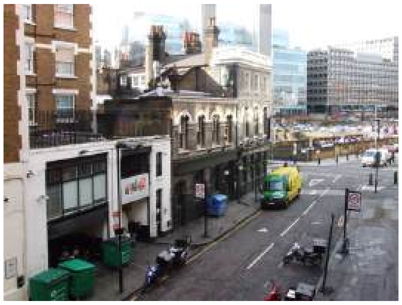

Warner Street, near Clerkenwell © Chris Whippet (cc-by-sa/2.0)

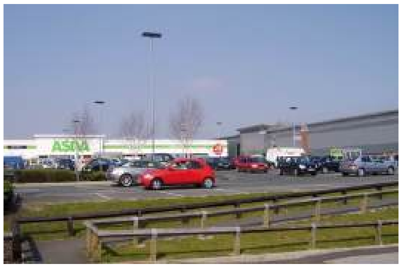

Burnden Park Retail Park © Mr M Evison (cc-by-sa/2.0)

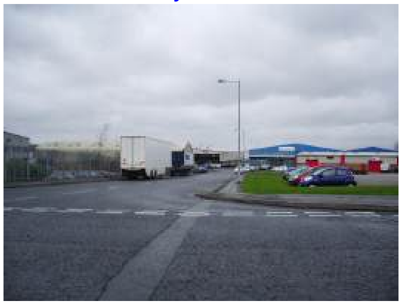

Farrington Road, Burnley © Alexander P Kapp (cc-by-sa/2.0)

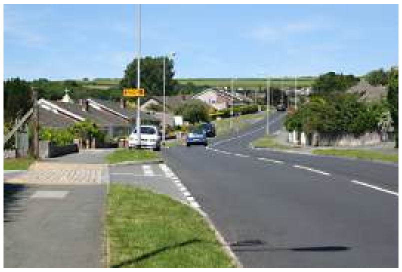

Larkham Lane © Tony Atkin (cc-by-sa/2.0)

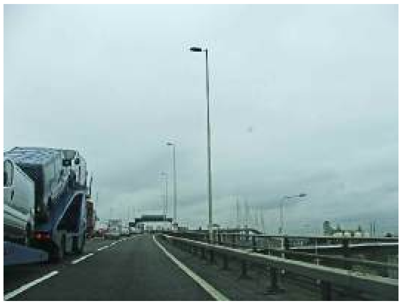

Approach to QE2 Bridge © Christine Matthews (cc-by-sa/2.0)

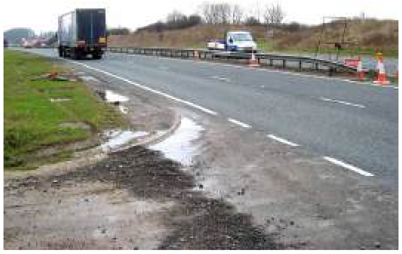

A19 at Middle Stotfold © Oliver Dixon (cc-by-sa/2.0)

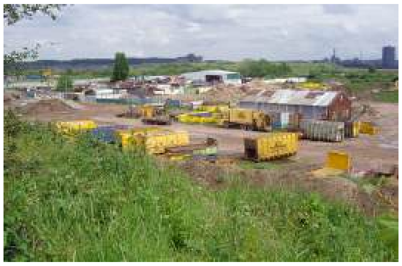

Waste site, Scunthorpe © Paul Harrop (cc-by-sa/2.0)

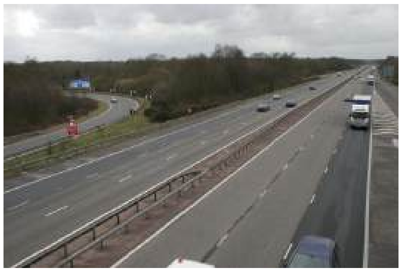

Hawley © Brendan and Ruth McCartney (cc-by-sa/2.0)

3.156 Trunk

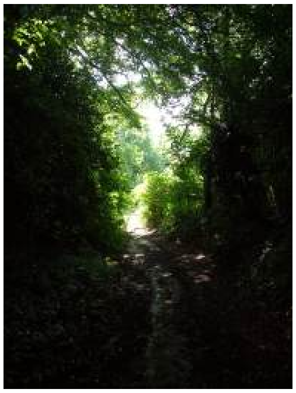

Crown Ash Hill TN16 © Philip Talmage (cc-by-sa/2.0)

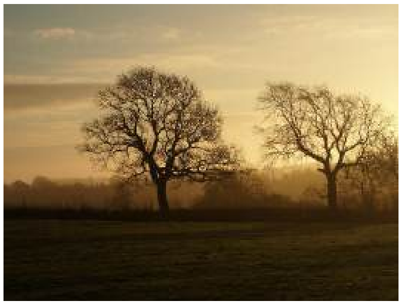

Wyse Hill, High Startforth © Cliff Occomore (cc-by-sa/2.0)

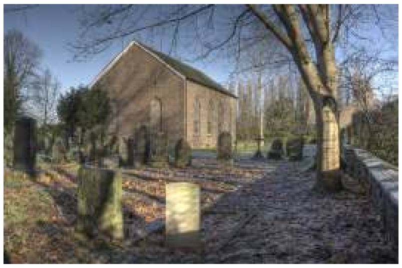

St George's Church, Carrington © Tom Jeffs (cc-by-sa/2.0)

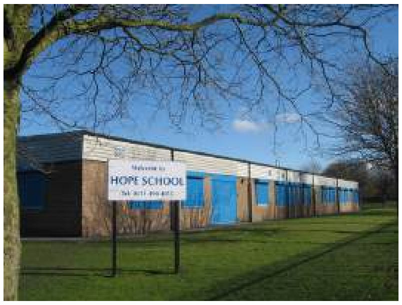

Hope School, Netherley © Sue Adair (cc-by-sa/2.0)

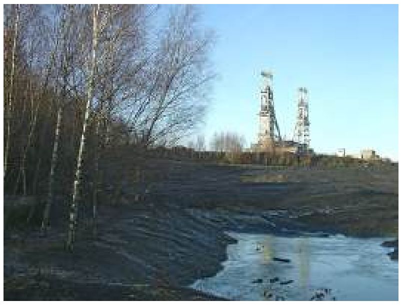

Clipstone Colliery from the Robin Hood Way © Alan Murray-Rust (cc-by-sa/2.0)

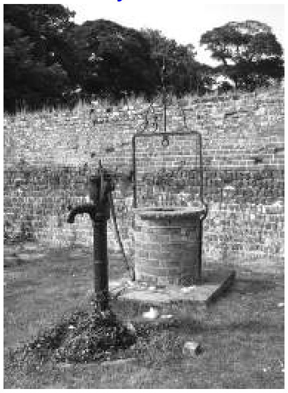

The old village pump and well © Evelyn Simak (cc-by-sa/2.0)

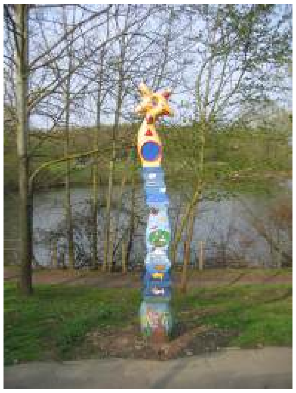

Milepost © David Stowell (cc-by-sa/2.0)

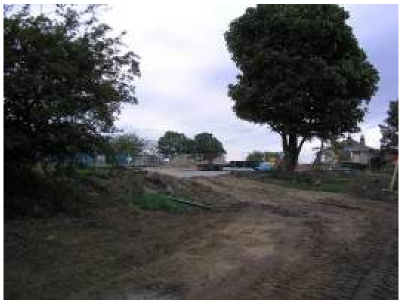

Building site - East Ardsley Primary School © Justin Credible (cc-by-sa/2.0)

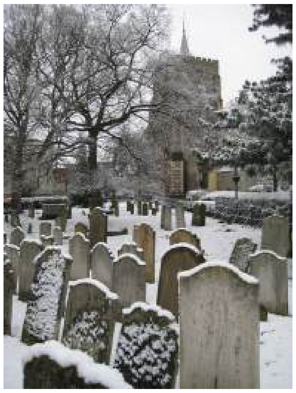

Watford: St Mary's Churchyard © Nigel Cox (cc-by-sa/2.0)

3.157 Twig

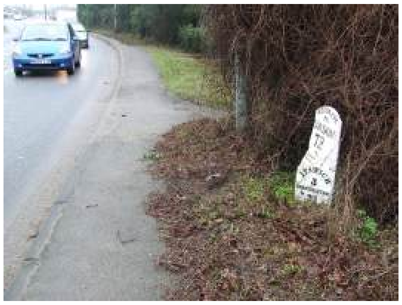

Old Cast Iron Milepost © Keith Evans (cc-by-sa/2.0)

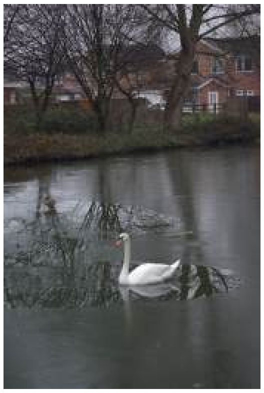

Swan on the Erewash Canal © Stephen McKay (cc-by-sa/2.0)

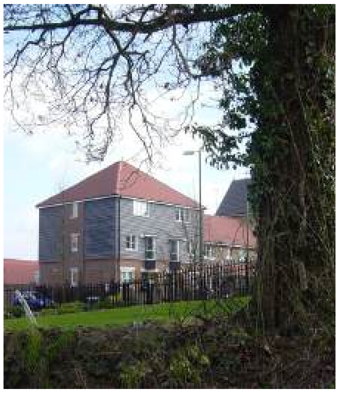

View from Long Lane towards new residential development © Euchiasmus (cc-by-sa/2.0)

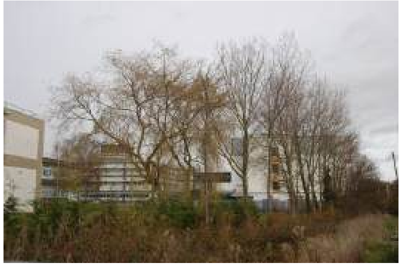

Basildon Hospital © Trevor Harris (cc-by-sa/2.0)

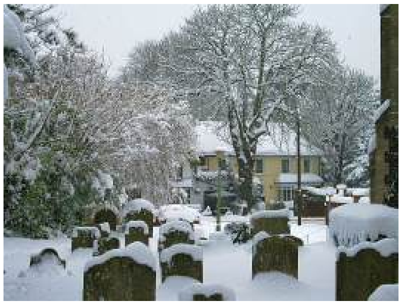

View from churchyard of St Martin of Tours © Hugh Craddock (cc-by-sa/2.0)

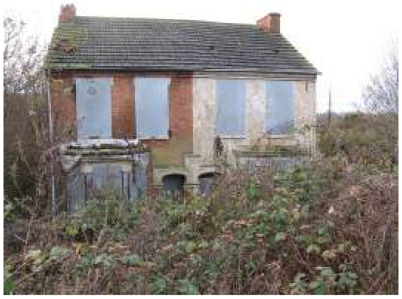

Houses disappearing into the undergrowth © M J Richardson (cc-by-sa/2.0)

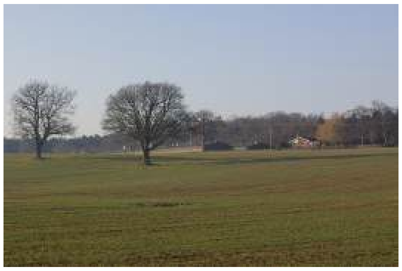

Coppice Farm © Ian Capper (cc-by-sa/2.0)

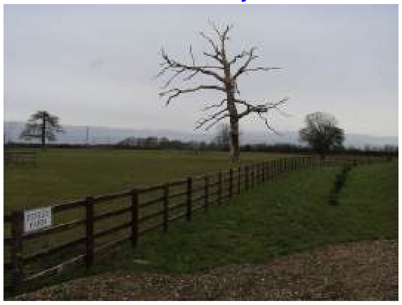

Tree still standing © Shaun Ferguson (cc-by-sa/2.0)

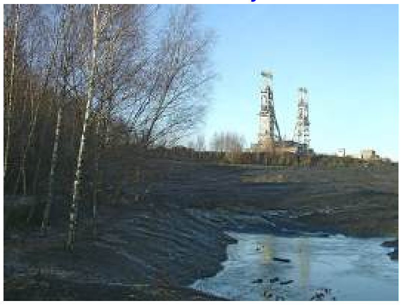

Clipstone Colliery from the Robin Hood Way © Alan Murray-Rust (cc-by-sa/2.0)

3.158 Urban Area

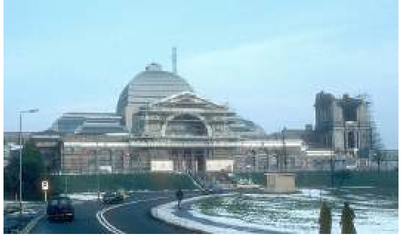

Alexandra Palace © E Gammie (cc-by-sa/2.0)

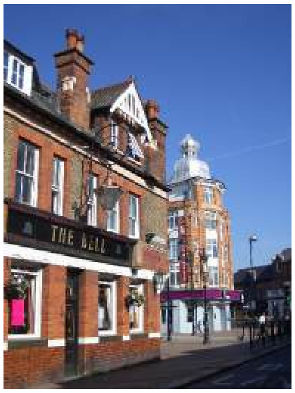

The Bell © Helene (cc-by-sa/2.0)

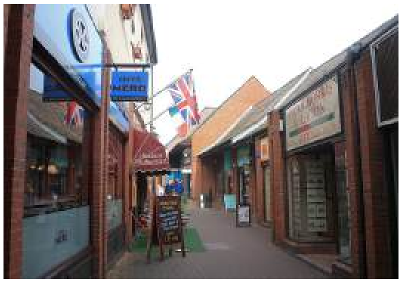

Church Walk, Great Malvern © Bob Embleton (cc-by-sa/2.0)

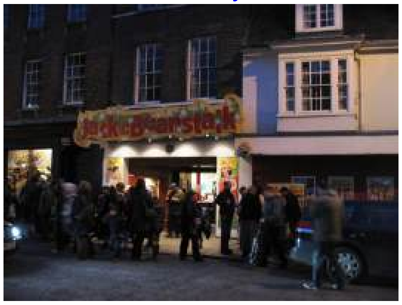

Cambridge panto season © Mr Ignavy (cc-by-sa/2.0)

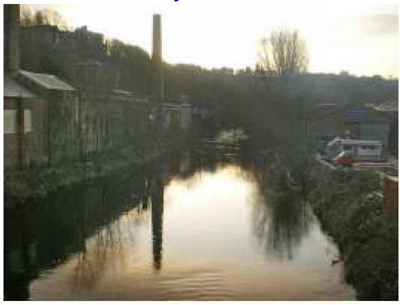

River Calder © Alexander P Kapp (cc-by-sa/2.0)

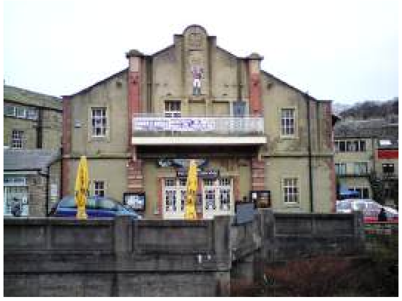

The Picturedrome © Tim Marchant (cc-by-sa/2.0)

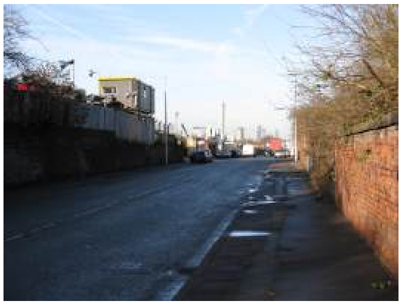

Gorton Road, Ashburys © Peter Whatley (cc-by-sa/2.0)

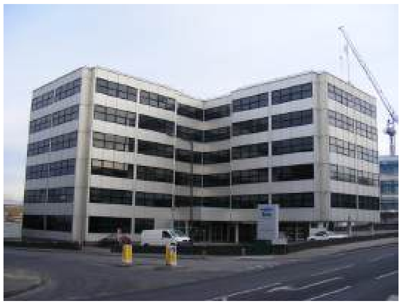

Office block - junction of Northolt Road / Stanley Road © PAUL FARMER (cc-by-sa/2.0)

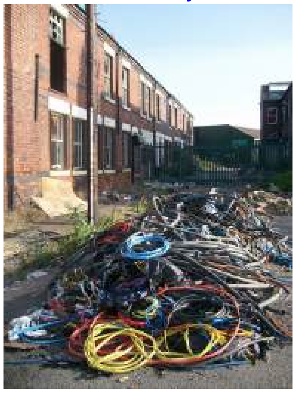

Melville Street © Lynne Shaw (cc-by-sa/2.0)

3.159 Urban Design

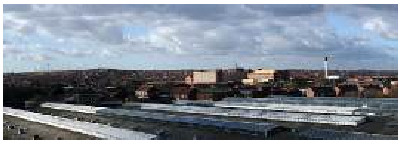

University Hospital of Hartlepool © George Ford (cc-by-sa/2.0)

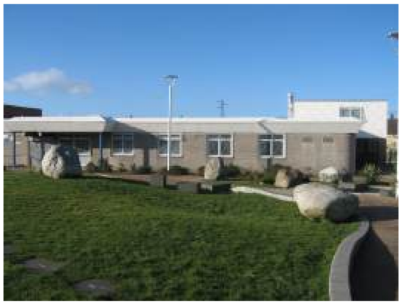

Netherton Health Centre © Sue Adair (cc-by-sa/2.0)

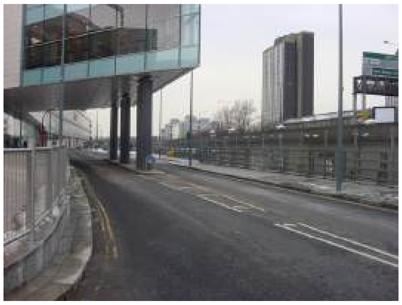

Westfield Way © Oxyman (cc-by-sa/2.0)

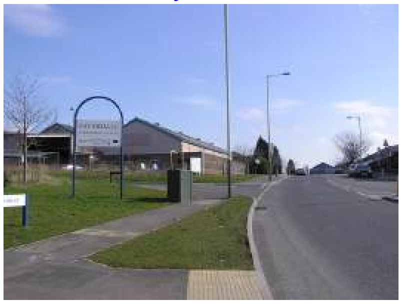

Faverdale Industrial Estate © Hugh Mortimer (cc-by-sa/2.0)

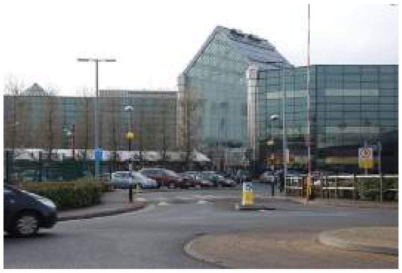

Merry Hill Shopping Centre © Brian Clift (cc-by-sa/2.0)

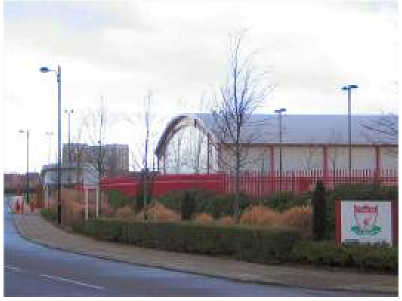

Football Academy © Roger May (cc-by-sa/2.0)

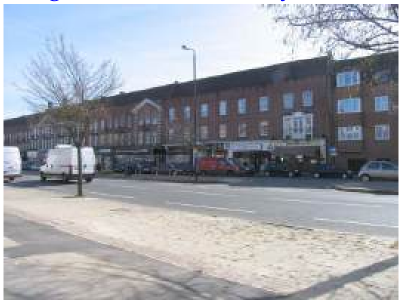

Uxbridge Road, Hatch End © John Salmon (cc-by-sa/2.0)

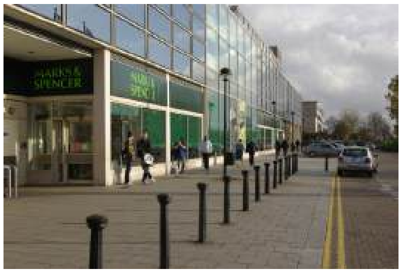

Milton Keynes Shopping Centre © Stephen McKay (cc-by-sa/2.0)

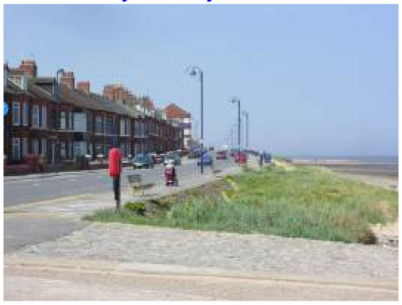

Granville Terrace © George Robinson (cc-by-sa/2.0)

3.160 Van

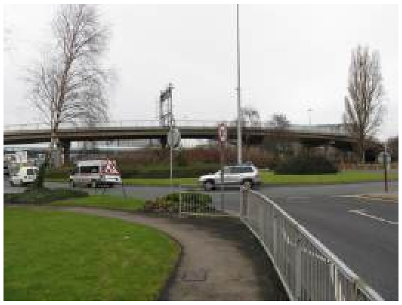
Manchester Airport - International Departures Flyover © Peter Whatley (cc-by-sa/2.0)

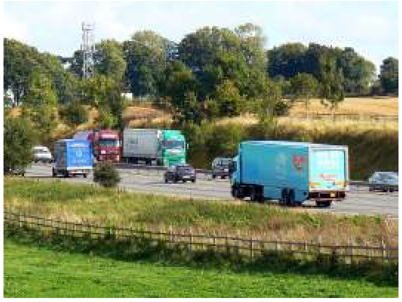
M4 near Peaks Downs, Wiltshire © Brian Robert Marshall (cc-by-sa/2.0)

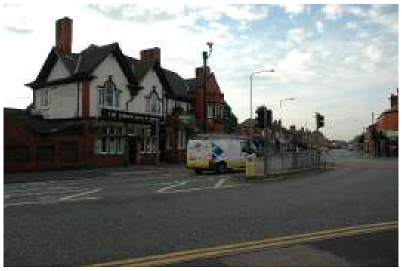
The Famous King and Queen pub © andy (cc-by-sa/2.0)

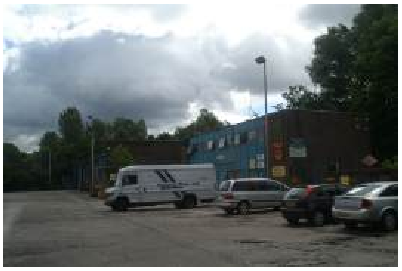
Arriva Bus Depot © David Long (cc-by-sa/2.0)

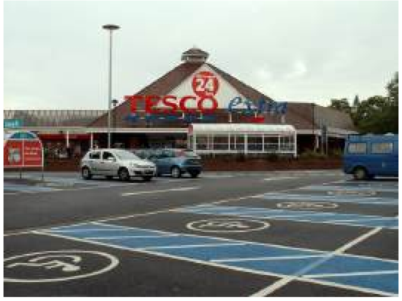

Tesco at High Wood, Colchester, Essex © Robert Edwards (cc-by-sa/2.0)

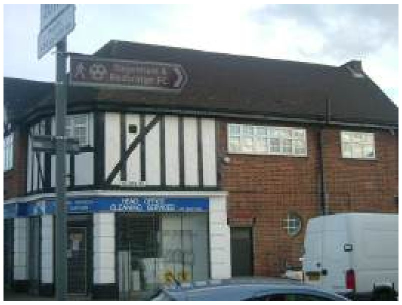
Victoria Road, Dagenham © Phillip Perry (cc-by-sa/2.0)

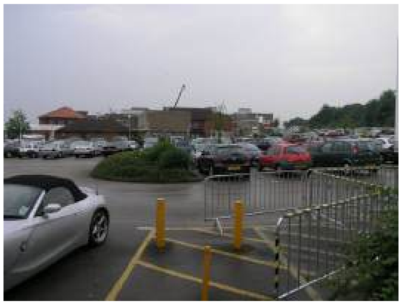
Queen's Hospital © Michael Patterson (cc-by-sa/2.0)

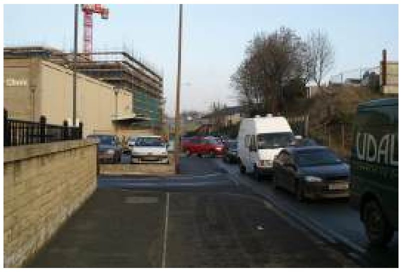
Caton Road, Lancaster, congested as usual. © David Long (cc-by-sa/2.0)

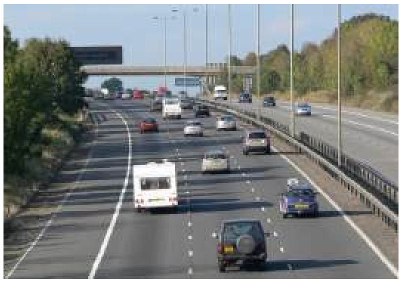
The M1 Motorway © Mat Fascione (cc-by-sa/2.0)

3.161 Vegetation

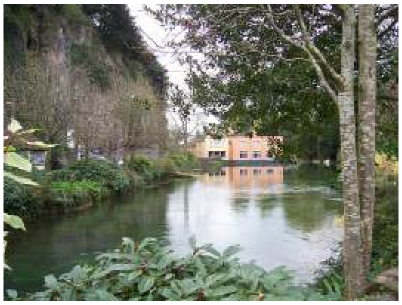

Cheddar Gorge © Pam Goodey (cc-by-sa/2.0)

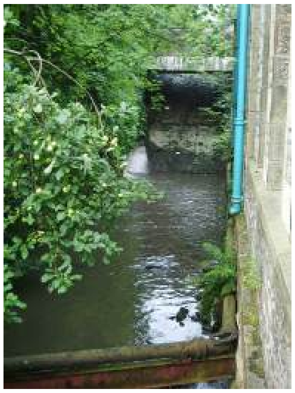

Mearley Brook as it flows along Stalwart Carpet Works © Alexander P Kapp (cc-by-sa/2.0)

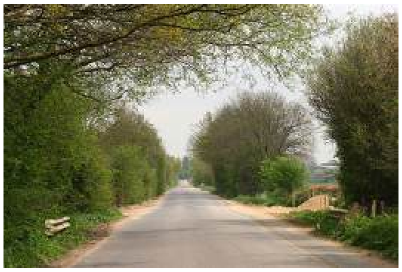

Cassington, the course of the old railway line © Martin Loader (cc-by-sa/2.0)

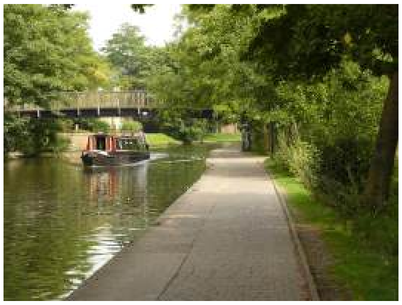

Nottingham Canal © Andy Jamieson (cc-by-sa/2.0)

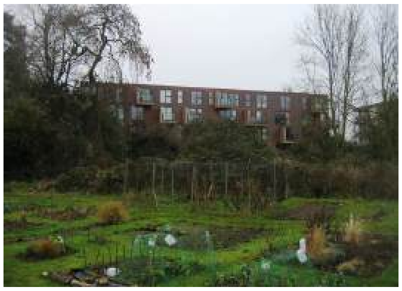

Do I fit in? © Mr Ignavy (cc-by-sa/2.0)

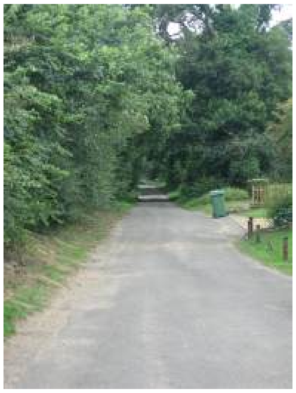

Bin hangin' around © Alison Rawson (cc-by-sa/2.0)

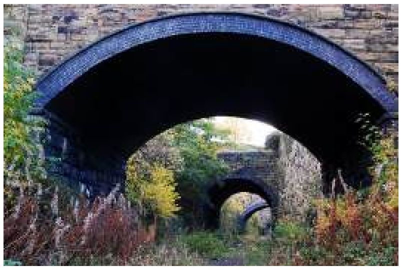

Heckmondwicke cutting-The Leeds new line © philld (cc-by-sa/2.0)

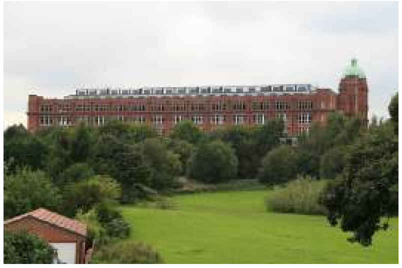

Astley Bridge Mill © Chris Allen (cc-by-sa/2.0)

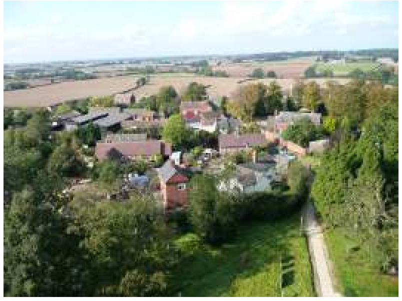

View to the north from Clifton Campville Church Spire © Brian Webster (cc-by-sa/2.0)

3.162 Vehicle

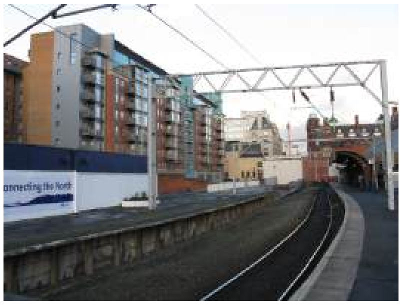

Manchester Oxford Road Station - Platform 5 © Peter Whatley (cc-by-sa/2.0)

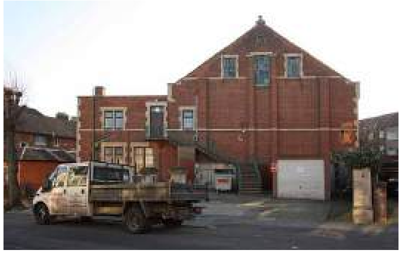

St Monica's Hall, Stonard Road, Palmers Green, London N13 © John Salmon (cc-by-sa/2.0)

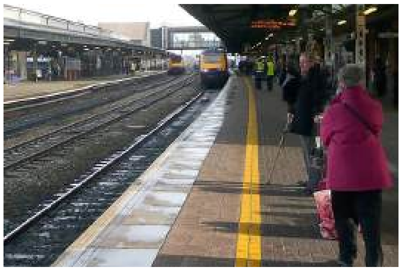

Reading station © Graham Horn (cc-by-sa/2.0)

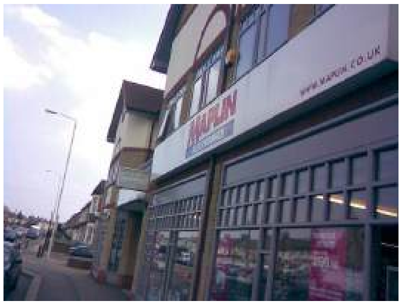

Maplin Store on Green Lane © Robert Lamb (cc-by-sa/2.0)

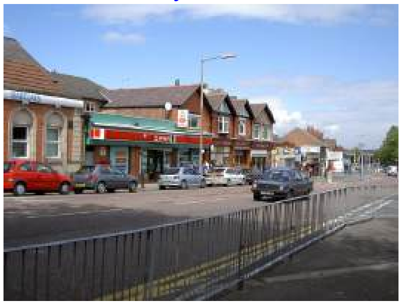

Claughton Village © David Quinn (cc-by-sa/2.0)

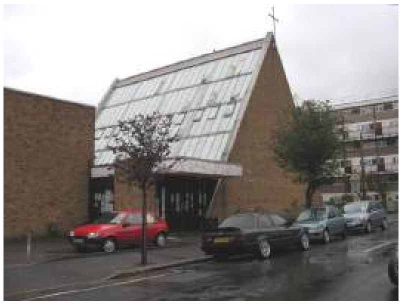

All Saints church, Leyton © Stephen Craven (cc-by-sa/2.0)

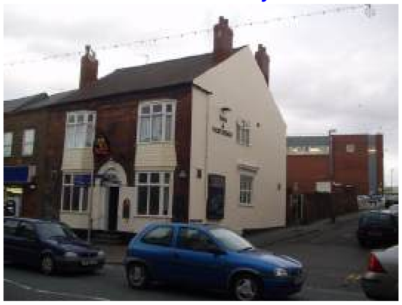

Dog & Partridge, Brierley Hill. © Brian Clift (cc-by-sa/2.0)

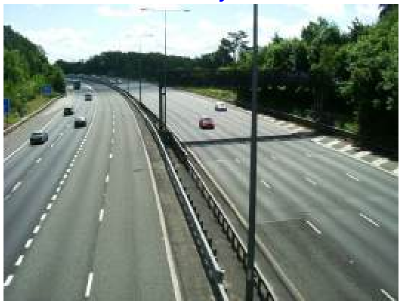

M40 motorway © Phillip Perry (cc-by-sa/2.0)

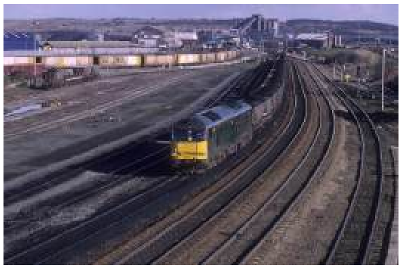

Coal for the Steel plant at Scunthorpe © roger geach (cc-by-sa/2.0)

3.163 Vehicle Registration Plate

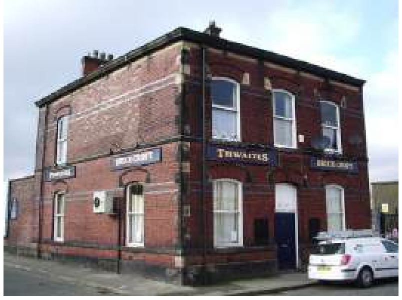

The Brickcroft, Brook Street, Bury © Alexander P Kapp (cc-by-sa/2.0)

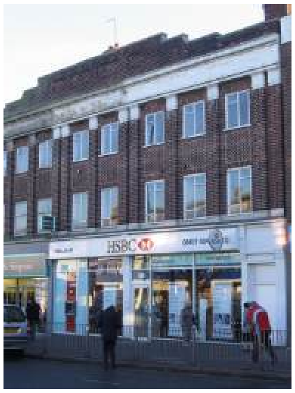

HSBC Bank Northfield. Sorting code 40-11-20 © Roy Hughes (cc-by-sa/2.0)

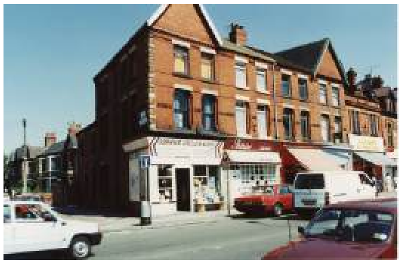

Shops in Green Lane © stan benbow (cc-by-sa/2.0)

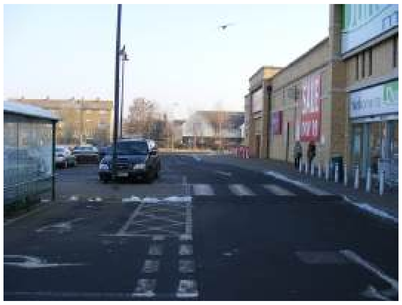

Car Park Homersham © PAUL FARMER (cc-by-sa/2.0)

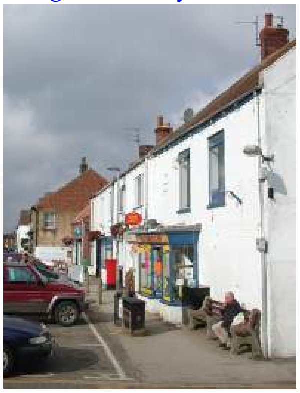

Market Place, Patrington © Paul Glazzard (cc-by-sa/2.0)

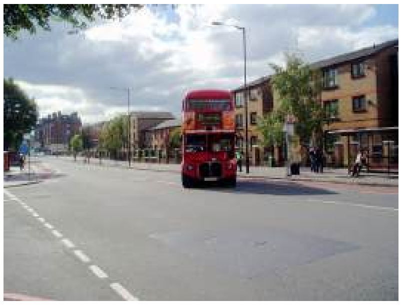

Routemaster near Clapton Pond © Dr Neil Clifton (cc-by-sa/2.0)

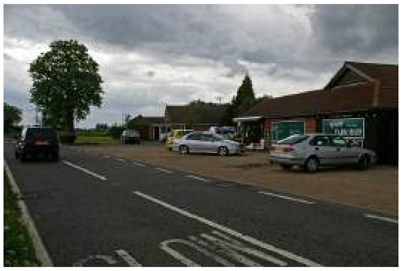

The Wrekin © Glyn Baker (cc-by-sa/2.0)

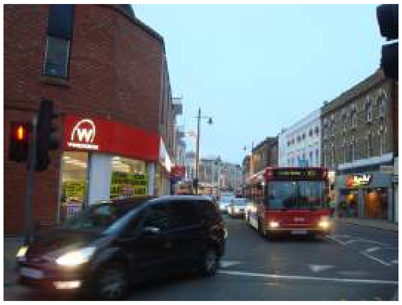

The Broadway, Wimbledon, London SW19 © Stacey Harris (cc-by-sa/2.0)

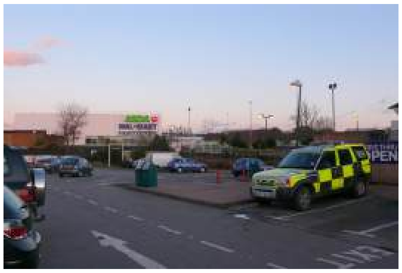

Asda, Cribbs, Causeway © Nigel Mykura (cc-by-sa/2.0)

3.164 Walkway

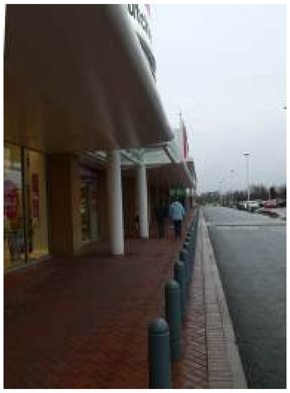

Greyhound Park arcade © Eirian Evans (cc-by-sa/2.0)

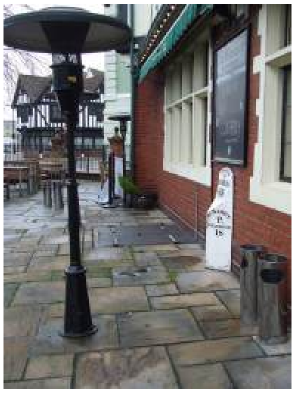

Old Cast Iron Milepost © Keith Evans (cc-by-sa/2.0)

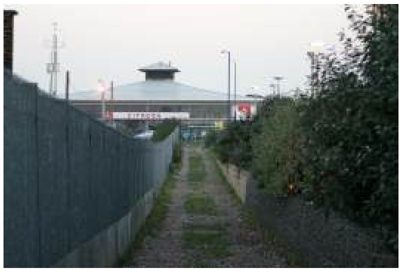

Public Footpath, Preston Industrial Estate © Mick Garratt (cc-by-sa/2.0)

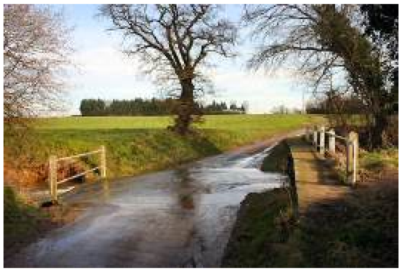

Finningham ford and footbridge © Bob Jones (cc-by-sa/2.0)

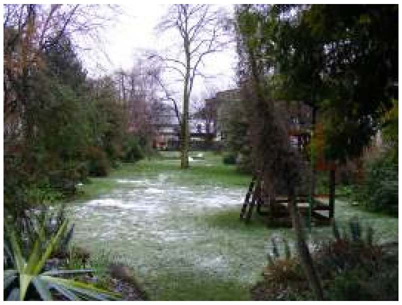

Gardens in Markham Square © PAUL FARMER (cc-by-sa/2.0)

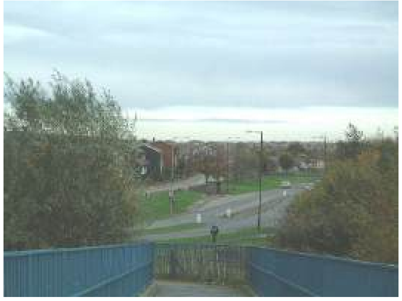

Town End © Steve McShane (cc-by-sa/2.0)

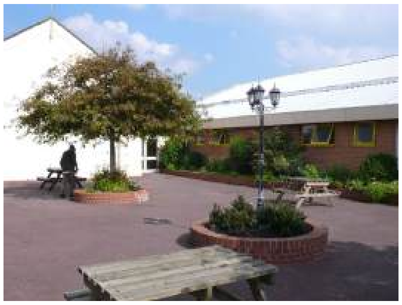

Lyme Bay Holiday Village, Seaton © Nigel Mykura (cc-by-sa/2.0)

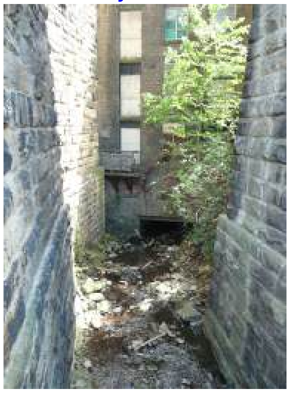

Underneath the arch, Market Street, Milnsbridge © Humphrey Bolton (cc-by-sa/2.0)

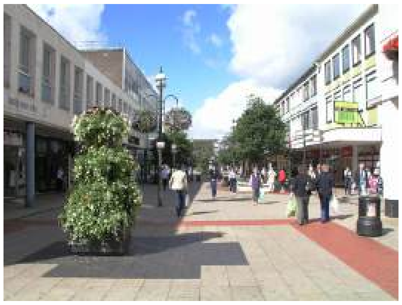

Queensway on a summer afternoon © Andy Potter (cc-by-sa/2.0)

3.165 Water

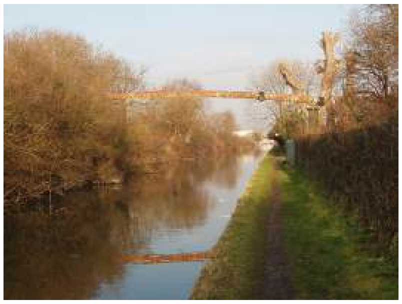
Pipe bridge over Paddington Branch canal © David Hawgood (cc-by-sa/2.0)

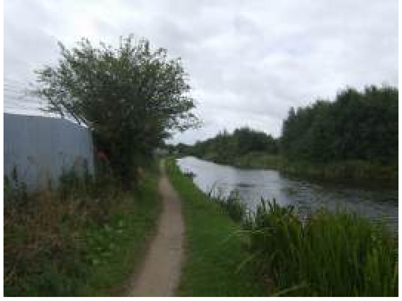
Wyrley and Essington Canal - near Pelsall Road © John M (cc-by-sa/2.0)

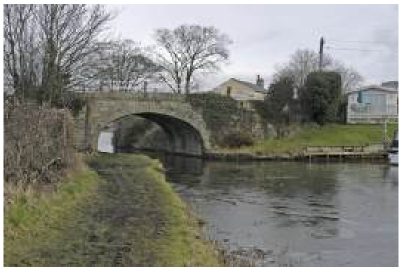
Winmarleigh Bridge (No 71) © Tom Richardson (cc-by-sa/2.0)

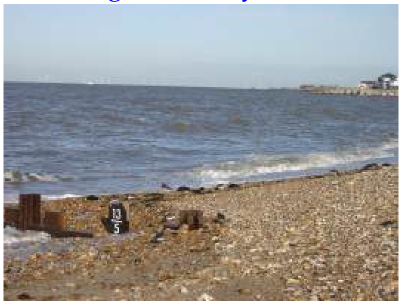
The beach with Turnstones. Seasalter © pam fray (cc-by-sa/2.0)

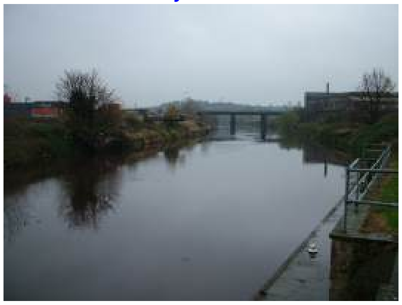
River Calder, Wakefield © John Goldsmith (cc-by-sa/2.0)

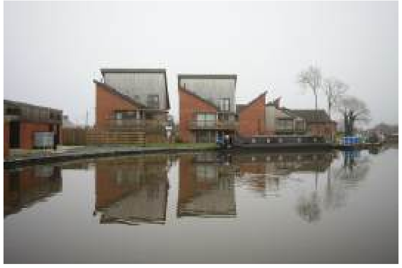
Eco Houses © Bob Jenkins (cc-by-sa/2.0)

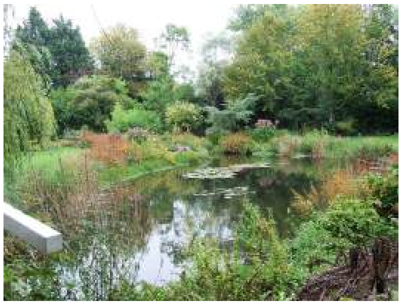
Gooderstone Water Gardens, Gooderstone, Norfolk © David Mills (cc-by-sa/2.0)

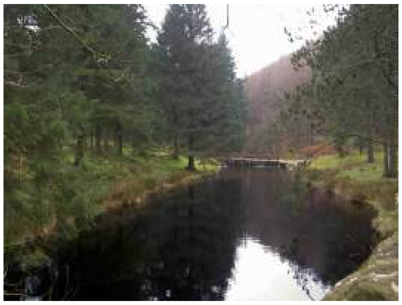
Ditch clough entering Howden reservoir © Steve Fareham (cc-by-sa/2.0)

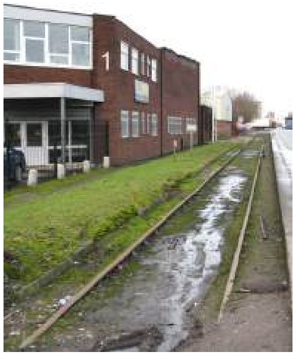
Disused Industrial Railway and Offices, Trafford Park Road © Peter Whatley (cc-by-sa/2.0)

3.166 Water Resources

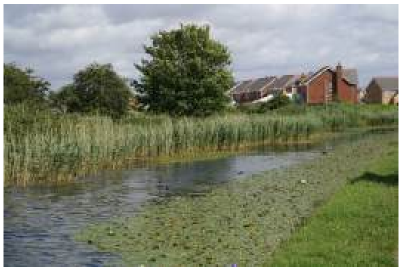

Leeds-Liverpool Canal at Waddicar © Mike Pennington (cc-by-sa/2.0)

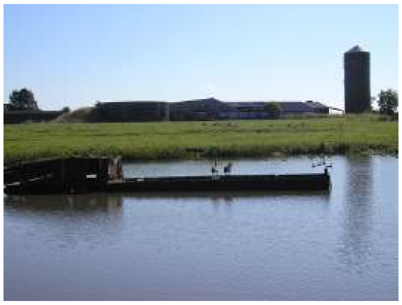

Higginslane Farm and Billinge Green Flash © Iain Lees (cc-by-sa/2.0)

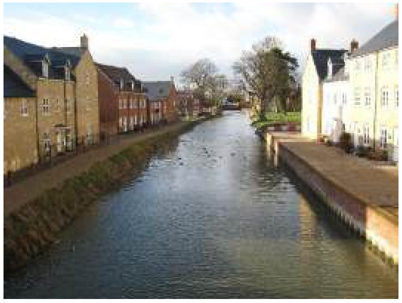

Ebley Wharf © David Stowell (cc-by-sa/2.0)

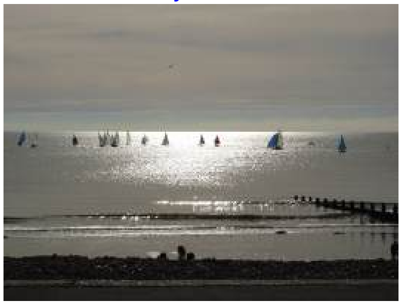

Autumn sailing at Felpham (3) © Roger Brooks (cc-by-sa/2.0)

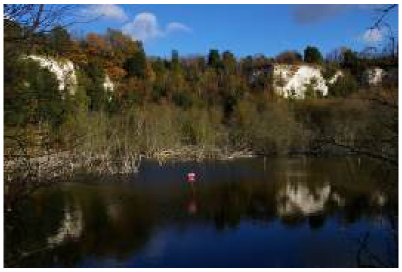

Pond in the Pit © Glyn Baker (cc-by-sa/2.0)

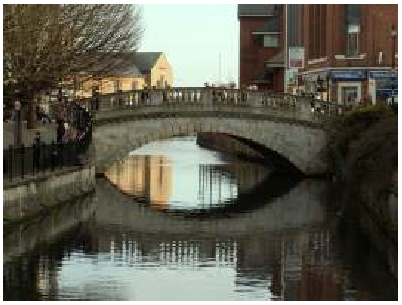

Moulsham Bridge over the river Can in Chelmsford © Robert Edwards (cc-by-sa/2.0)

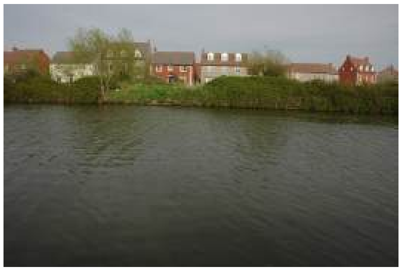

New housing at Quedgeley © Philip Halling (cc-by-sa/2.0)

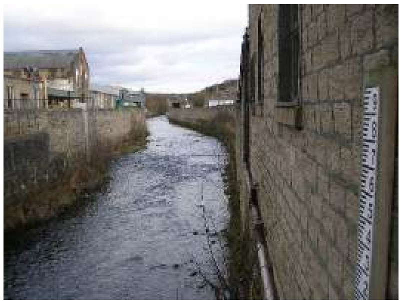

Fancy a swim anyone? © Paul Johnston-Knight (cc-by-sa/2.0)

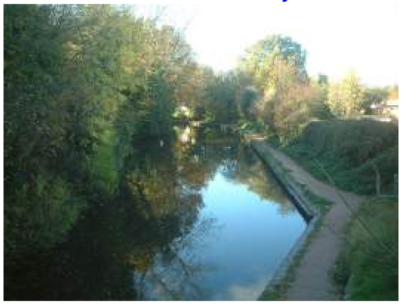

Happy Valley Boating © planetearthisblue (cc-by-sa/2.0)

3.167 Water Transportation

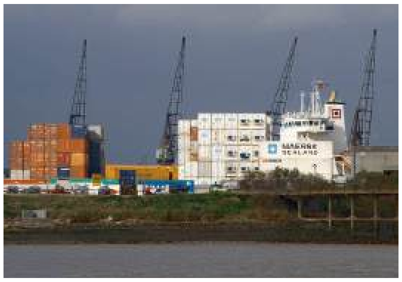

Container Stack at Tilbury © Glyn Baker (cc-by-sa/2.0)

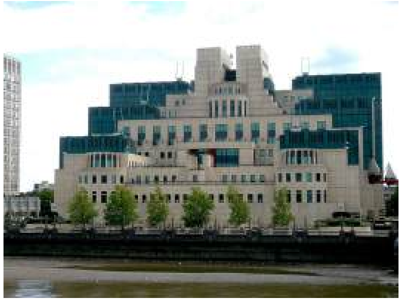

MI6 Building © Iain Crump (cc-by-sa/2.0)

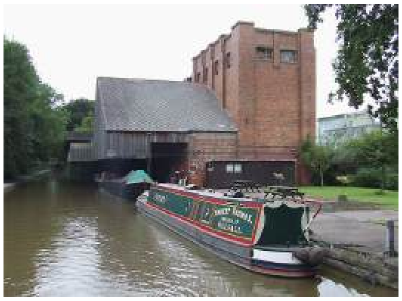

Cadbury Wharf, Knighton, Staffordshire © Roger D Kidd (cc-by-sa/2.0)

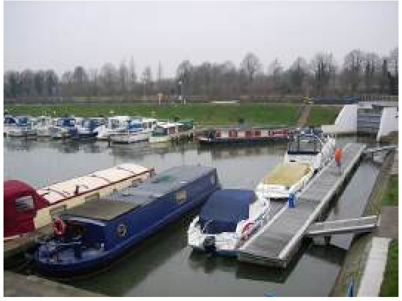

Thames Marina © Hugh Venables (cc-by-sa/2.0)

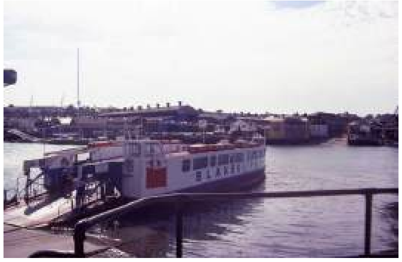

The Cowes Floating Bridge © Barry Shimmon (cc-by-sa/2.0)

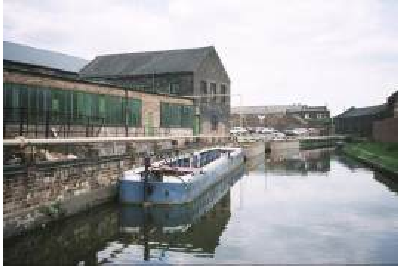

Potteries boats, Caldon Canal © David Stowell (cc-by-sa/2.0)

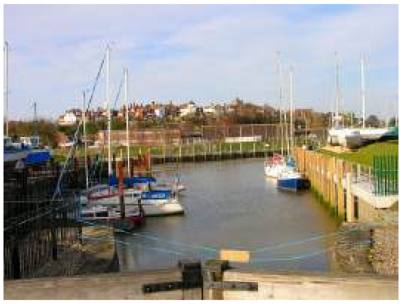

Confluence of the Brede and Tillingham Rivers © Simon Carey (cc-by-sa/2.0)

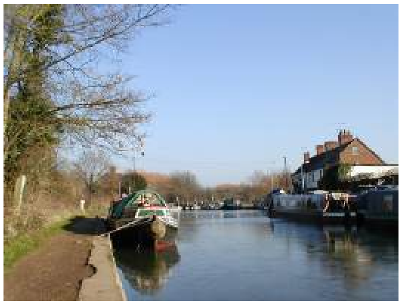

Grand Union Canal beside The Cape of Good Hope © Row17 (cc-by-sa/2.0)

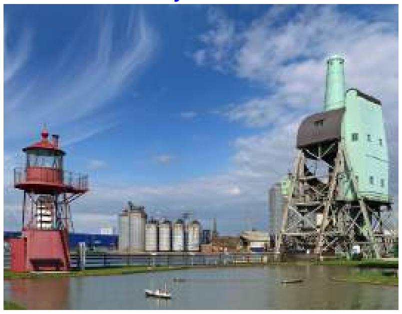

Goole Model Boat Club pond © Steve Fareham (cc-by-sa/2.0)

3.168 Watercourse

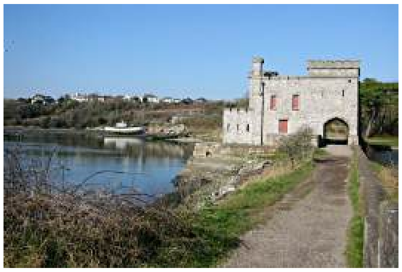
Radford Lake Causeway © Tony Atkin (cc-by-sa/2.0)

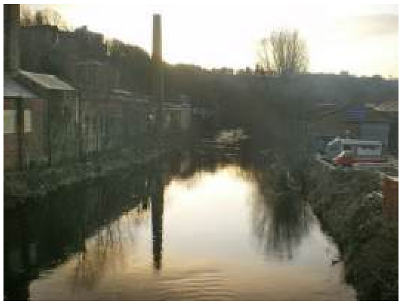
River Calder © Alexander P Kapp (cc-by-sa/2.0)

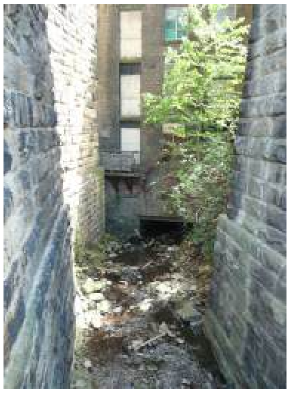
Underneath the arch, Market Street, Milnsbridge © Humphrey Bolton (cc-by-sa/2.0)

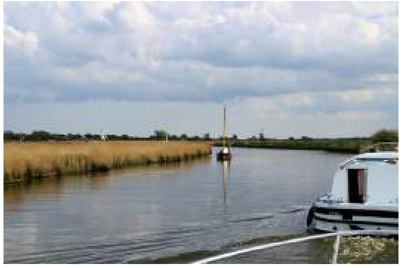
River Thurne above Thurne © Pierre Terre (cc-by-sa/2.0)

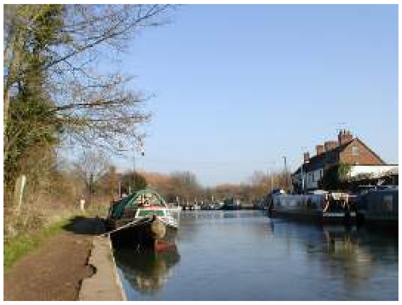
Grand Union Canal beside The Cape of Good Hope © Row17 (cc-by-sa/2.0)

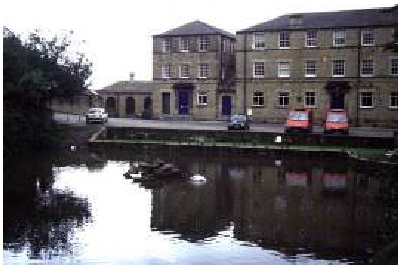
Nortonthorpe Mills, Scissett © Chris Allen (cc-by-sa/2.0)

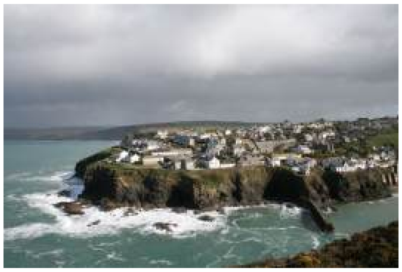
Port Isaac from Lobber Point © Hugh Craddock (cc-by-sa/2.0)

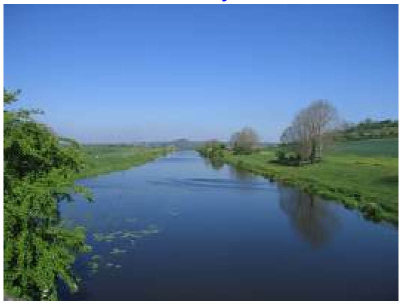
King's Sedgemoor Drain at Parchey Bridge © Dave Vaughan (cc-by-sa/2.0)

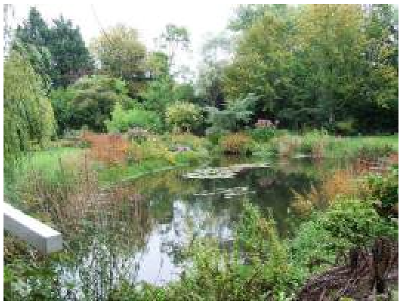
Gooderstone Water Gardens, Gooderstone, Norfolk © David Mills (cc-by-sa/2.0)

3.169 Waterway

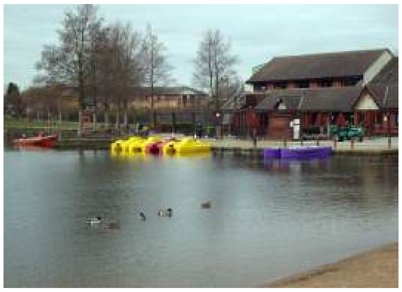

The Windsurfer © David Pickersgill (cc-by-sa/2.0)

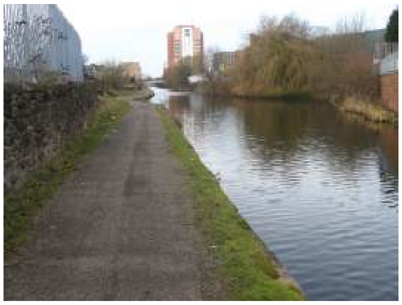

Ashton Canal © Chris Wimbush (cc-by-sa/2.0)

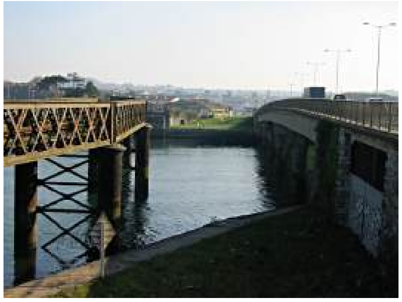

Two Bridges Cross The Laira © Tony Atkin (cc-by-sa/2.0)

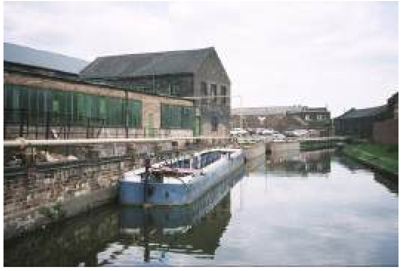

Potteries boats, Caldon Canal © David Stowell (cc-by-sa/2.0)

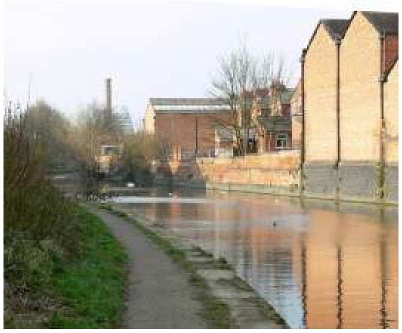

Grand Union Canal and towpath in Leicester. © Mat Fascione (cc-by-sa/2.0)

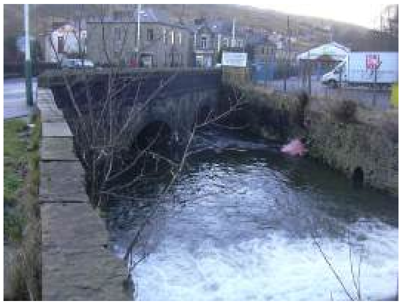

Bacup Road Bridge over the Irwell © Robert Wade (cc-by-sa/2.0)

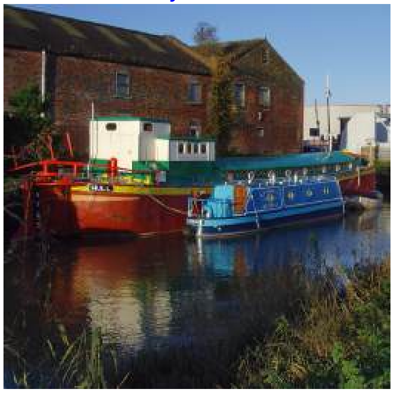

Barges at Grovehill, Beverley © Paul Harrop (cc-by-sa/2.0)

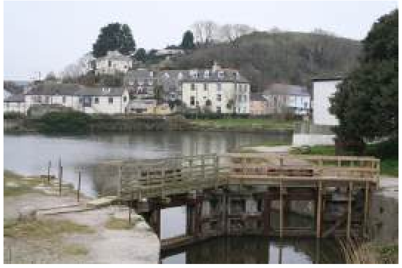

St Austell: Pentewan Harbour © Martin Bodman (cc-by-sa/2.0)

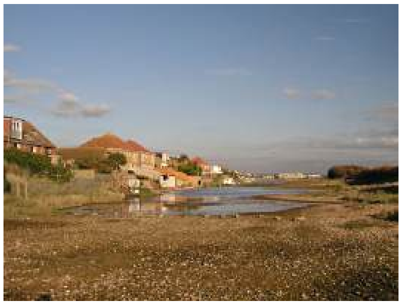

Widewater Lagoon Nature Reserve © Andy Potter (cc-by-sa/2.0)

3.170 Wetland

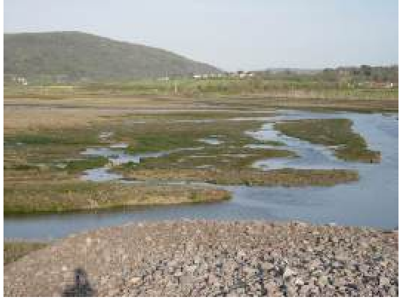

Porlock Saltings © Hugh Venables (cc-by-sa/2.0)

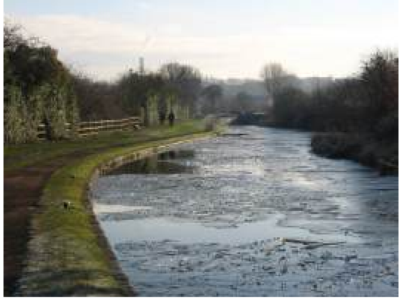

Oxley - ice below Lock 18 © Dave Bevis (cc-by-sa/2.0)

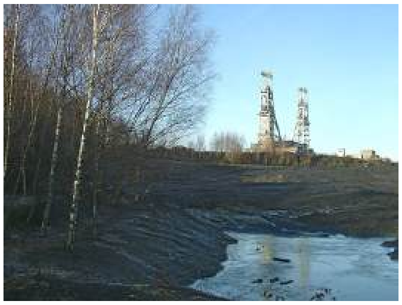

Clipstone Colliery from the Robin Hood Way © Alan Murray-Rust (cc-by-sa/2.0)

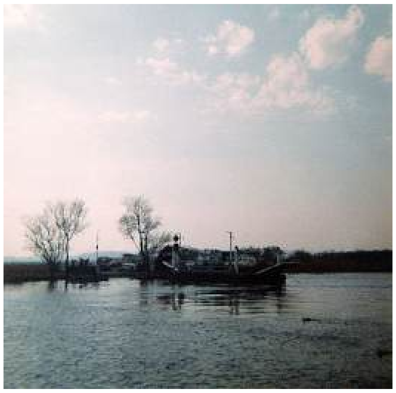

Reedham Ferry © Gerald England (cc-by-sa/2.0)

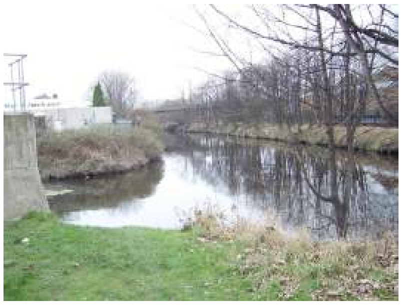

Confluence of River Rother with River Don © Shelagh Craven (cc-by-sa/2.0)

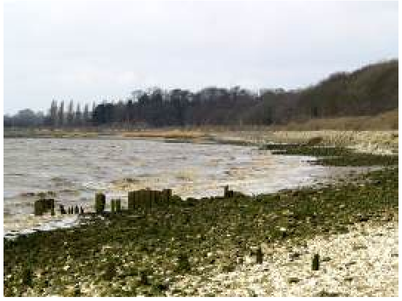

The River Humber Foreshore west of Hessle © Andy Beecroft (cc-by-sa/2.0)

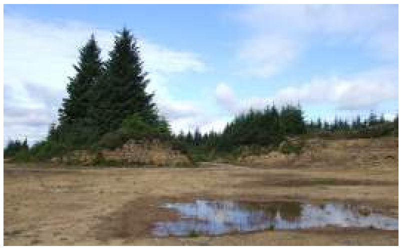

Green's Crag © Peter McDermott (cc-by-sa/2.0)

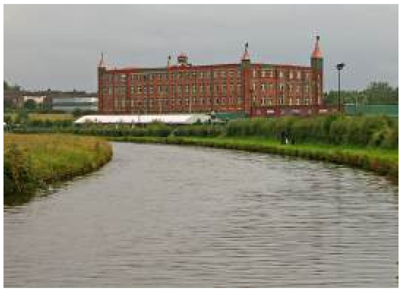

Botany Bay © Mr T (cc-by-sa/2.0)

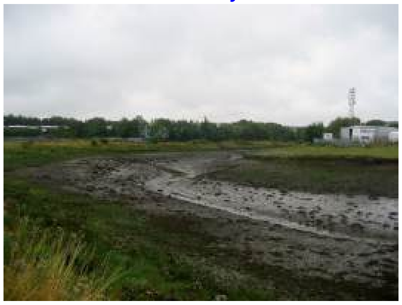

Creek in Cowpen New Town © Chris Heaton (cc-by-sa/2.0)

3.171 Winter

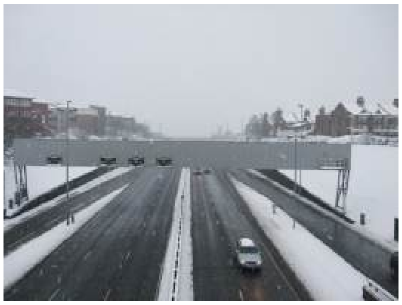

The A102 on a snowy day © Linda Craven (cc-by-sa/2.0)

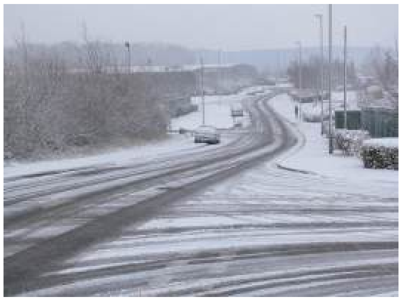

Centurion Way, Leicester © Mat Fascione (cc-by-sa/2.0)

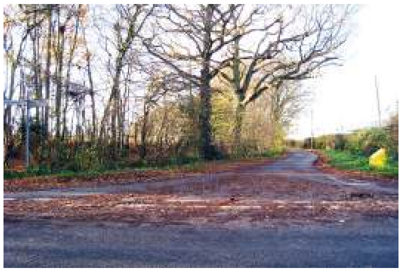

Crump's Lane, Grafty Green, Kent © Oast House Archive (cc-by-sa/2.0)

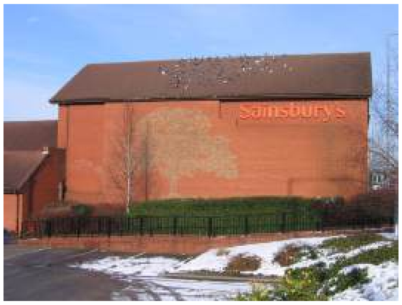

Sainsbury's Selly Oak with "Oaktree" © Roy Hughes (cc-by-sa/2.0)

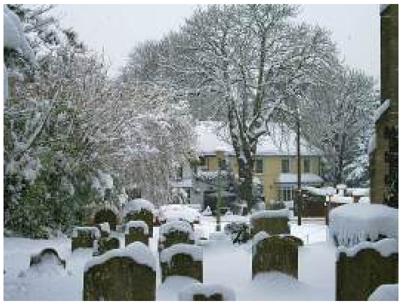

View from churchyard of St Martin of Tours © Hugh Craddock (cc-by-sa/2.0)

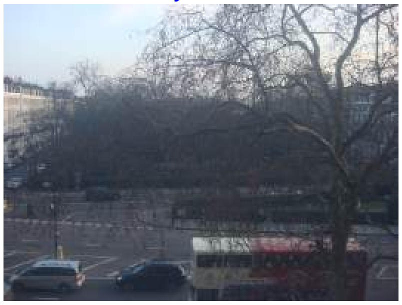

Thurloe Square from the V & A © Oxyman (cc-by-sa/2.0)

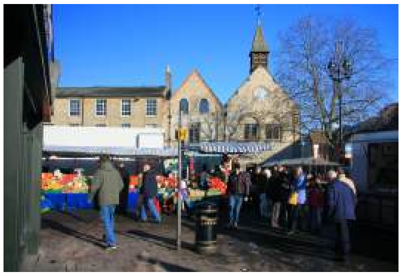

Market day in Bury St Edmunds © Bob Jones (cc-by-sa/2.0)

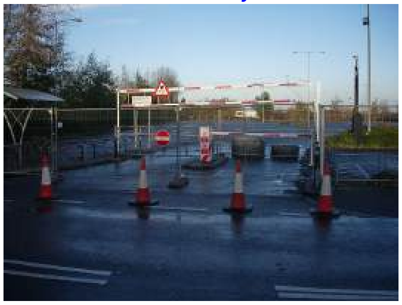

Cowley Road Don't Park and Don't Ride © Hugh Venables (cc-by-sa/2.0)

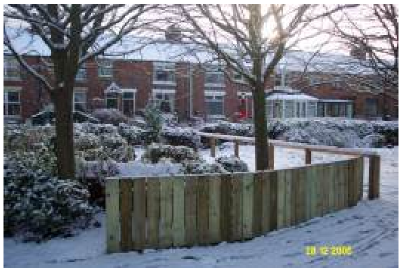

Street Houses on West Bridge Street © Andy Brass (cc-by-sa/2.0)

3.172 Wood

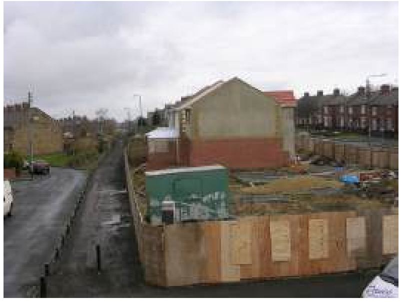
Shoddy new housing at Leadgate © Oliver Dixon (cc-by-sa/2.0)

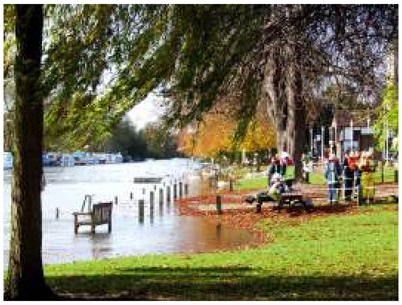
River Green, Thorpe in flood © Martin Thirkettle (cc-by-sa/2.0)

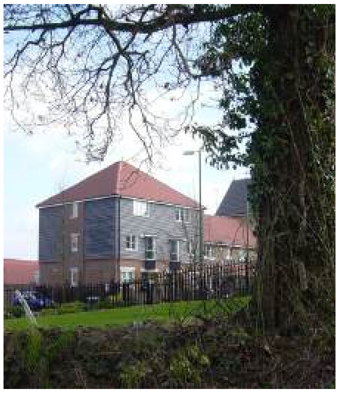
View from Long Lane towards new residential development © Euchiasmus (cc-by-sa/2.0)

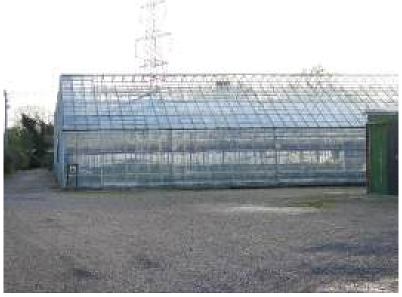
Greenhouses © Hugh Venables (cc-by-sa/2.0)

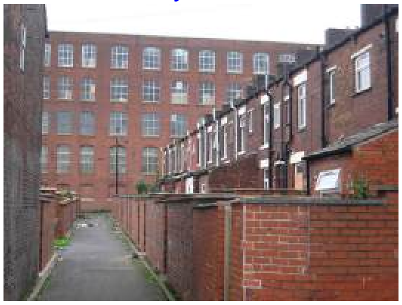
Rear of Eleanor Street Oldham © Paul Anderson (cc-by-sa/2.0)

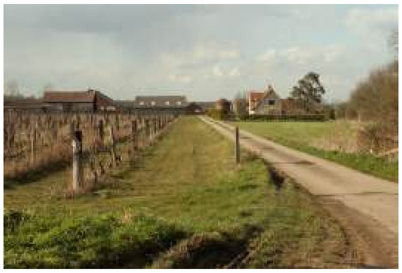
Looking at New Hall Farm from Baron's Lane © Robert Edwards (cc-by-sa/2.0)

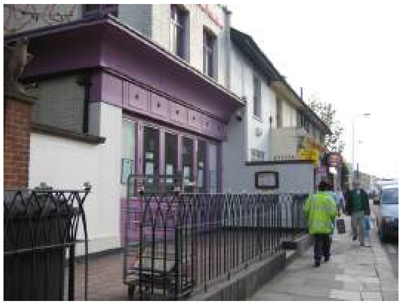
Tea Rooms des Artistes, Wandsworth Road, London © Oast House Archive (cc-by-sa/2.0)

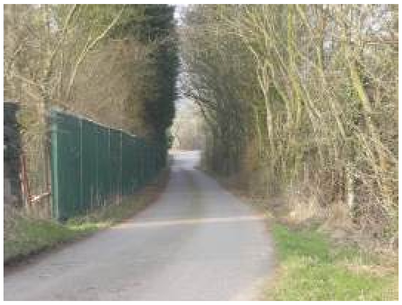
Lane beside Industrial Estate © Row17 (cc-by-sa/2.0)

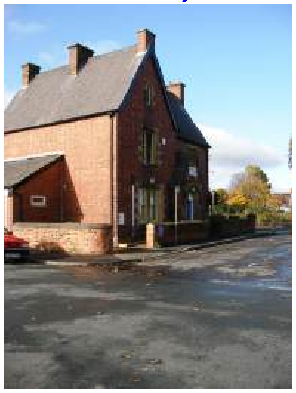
Youth Centre Ossett © SMJ (cc-by-sa/2.0)

3.173 Woody Plant

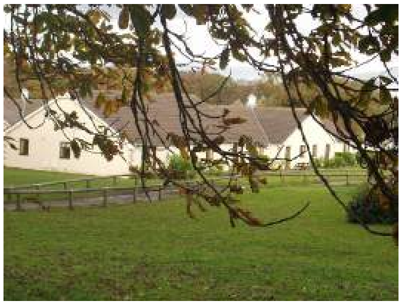

Ribby Hall Holiday Village © G McK (cc-by-sa/2.0)

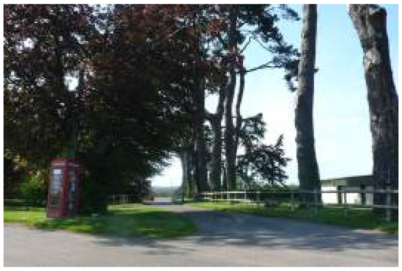

Woodsford © Nigel Mykura (cc-by-sa/2.0)

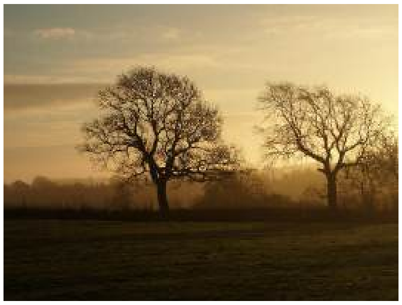

Wyse Hill, High Startforth © Cliff Occomore (cc-by-sa/2.0)

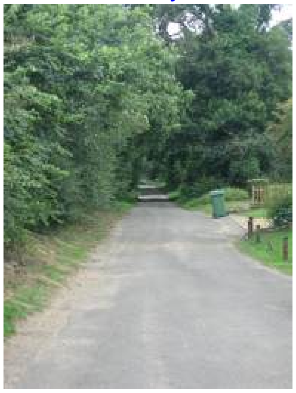

Bin hangin' around © Alison Rawson (cc-by-sa/2.0)

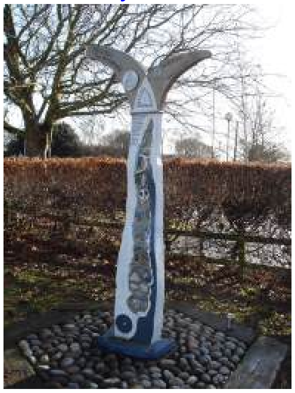

National cycle route marker post by Ipswich hospital © Oxymoron (cc-by-sa/2.0)

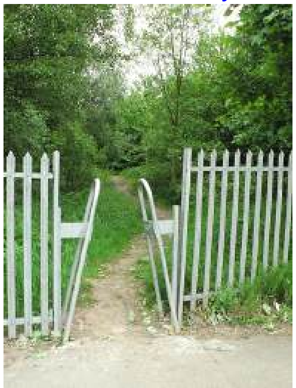

Squeeze stile by Ring Road, West Park © Rich Tea (cc-by-sa/2.0)

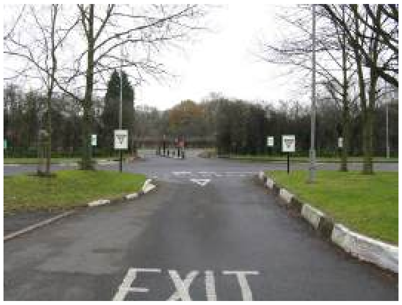

Feeling Lucky? © Peter Whatley (cc-by-sa/2.0)

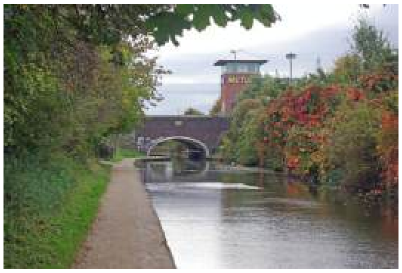

Coventry Canal, Courthouse Green © Stephen McKay (cc-by-sa/2.0)

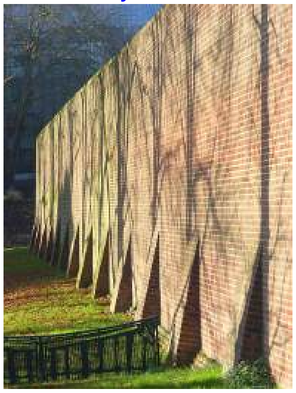

Prison wall, Reading © Andrew Smith (cc-by-sa/2.0)

4 Full Regression Results

4.1 Main Regression

Table S174: Complete regression results.

term	estimate	std.error	statistic	p.value	conf
Listed Building	0.61	0.05	12.31	0.00	[0.52, 0.71]
Agriculture	0.27	0.11	2.48	0.01	[0.06, 0.48]
Apartment	-0.01	0.08	-0.17	0.86	[-0.17, 0.14]
Asphalt	-0.27	0.05	-5.61	0.00	[-0.36, -0.17]
Atmospheric Phenomenon	-0.16	0.12	-1.30	0.20	[-0.4, 0.08]
Automotive Design	-0.21	0.11	-1.95	0.05	[-0.43, 0]
Automotive Exterior	-0.06	0.06	-0.97	0.33	[-0.18, 0.06]
Automotive Lighting	-0.10	0.06	-1.65	0.10	[-0.22, 0.02]
Automotive Parking Light	-0.20	0.06	-3.09	0.00	[-0.32, -0.07]
Automotive Tail Brake Light	0.08	0.13	0.65	0.51	[-0.16, 0.33]
Automotive Tire	0.02	0.09	0.19	0.85	[-0.15, 0.19]
Automotive Wheel System	-0.01	0.13	-0.11	0.91	[-0.27, 0.25]
Biome	0.35	0.17	2.09	0.04	[0.02, 0.68]
Boat	1.14	0.18	6.20	0.00	[0.78, 1.49]
Body Owater	0.20	0.13	1.45	0.15	[-0.07, 0.46]
Branch	0.27	0.11	2.46	0.01	[0.05, 0.48]
Bridge	-0.01	0.11	-0.11	0.91	[-0.22, 0.2]
Building	-0.21	0.04	-4.80	0.00	[-0.3, -0.13]
Building Material	-0.11	0.14	-0.80	0.42	[-0.39, 0.16]
Bus	0.11	0.12	0.93	0.35	[-0.13, 0.35]
Canal	0.17	0.13	1.31	0.19	[-0.09, 0.44]
Car	0.11	0.06	1.90	0.06	[0, 0.23]
Channel	0.11	0.14	0.78	0.44	[-0.17, 0.38]
Chapel	0.35	0.18	1.96	0.05	[0, 0.7]
Church	0.46	0.14	3.27	0.00	[0.18, 0.73]
City	-0.12	0.04	-2.87	0.00	[-0.2, -0.04]
Cloud	-0.08	0.03	-2.54	0.01	[-0.14, -0.02]
Commercial Building	-0.26	0.06	-4.07	0.00	[-0.38, -0.13]
Concrete Bridge	-0.23	0.17	-1.34	0.18	[-0.56, 0.11]
Condominium	0.12	0.11	1.12	0.26	[-0.09, 0.34]
Cottage	0.36	0.05	6.55	0.00	[0.25, 0.47]
Cumulus	-0.06	0.07	-0.83	0.40	[-0.2, 0.08]
Daytime	-0.01	0.14	-0.04	0.96	[-0.27, 0.26]
Dirt Road	0.09	0.16	0.54	0.59	[-0.23, 0.4]
Downtown	0.14	0.10	1.37	0.17	[-0.06, 0.35]
Driveway	0.19	0.14	1.37	0.17	[-0.08, 0.46]
Dusk	0.23	0.15	1.49	0.14	[-0.07, 0.53]
Ecoregion	0.30	0.13	2.41	0.02	[0.06, 0.55]
Electrical Supply	-0.04	0.13	-0.33	0.74	[-0.31, 0.22]
Electricity	-0.07	0.06	-1.07	0.28	[-0.19, 0.06]
Engineering	0.03	0.13	0.24	0.81	[-0.22, 0.28]
Evening	-0.16	0.12	-1.32	0.19	[-0.39, 0.08]
Event	-0.12	0.08	-1.48	0.14	[-0.29, 0.04]
Family Car	-0.01	0.12	-0.09	0.93	[-0.25, 0.23]

Field	-0.09	0.10	-0.91	0.36	[-0.28, 0.1]
Fixture	-0.02	0.07	-0.27	0.78	[-0.15, 0.11]
Flower	0.19	0.14	1.37	0.17	[-0.08, 0.45]
Fluvial Landforms Ostreams	0.19	0.14	1.41	0.16	[-0.08, 0.46]
Font	-0.22	0.13	-1.75	0.08	[-0.47, 0.03]
Forest	0.68	0.12	5.83	0.00	[0.45, 0.91]
Freeway	-0.15	0.14	-1.10	0.27	[-0.42, 0.12]
Garden	0.05	0.15	0.36	0.72	[-0.24, 0.35]
Gas	-0.21	0.05	-4.51	0.00	[-0.3, -0.12]
Girder Bridge	0.30	0.15	1.97	0.05	[0, 0.61]
Grass	-0.06	0.05	-1.29	0.20	[-0.15, 0.03]
Grass Family	0.09	0.09	1.00	0.32	[-0.09, 0.26]
Grassland	0.07	0.08	0.91	0.36	[-0.09, 0.24]
Groundcover	0.03	0.08	0.31	0.75	[-0.14, 0.19]
Headquarters	0.00	0.11	0.01	0.99	[-0.22, 0.22]
Highland	0.33	0.14	2.36	0.02	[0.06, 0.6]
Highway	-0.23	0.11	-2.03	0.04	[-0.46, -0.01]
Hill	0.11	0.09	1.17	0.24	[-0.07, 0.3]
Home	0.02	0.10	0.24	0.81	[-0.17, 0.22]
Horizon	0.19	0.07	2.87	0.00	[0.06, 0.32]
House	0.15	0.04	4.16	0.00	[0.08, 0.22]
Human Settlement	0.13	0.09	1.50	0.13	[-0.04, 0.3]
Industry	0.29	0.18	1.65	0.10	[-0.06, 0.64]
Infrastructure	-0.03	0.05	-0.68	0.49	[-0.14, 0.07]
Intercept	2.60	0.12	20.95	0.00	[2.35, 2.84]
Lacustrine Plain	0.04	0.14	0.29	0.77	[-0.23, 0.31]
Lake	0.58	0.11	5.03	0.00	[0.35, 0.8]
Land Lot	-0.25	0.04	-5.57	0.00	[-0.34, -0.16]
Land Vehicle	0.07	0.06	1.15	0.25	[-0.05, 0.2]
Landmark	0.12	0.09	1.36	0.17	[-0.05, 0.29]
Landscape	0.03	0.04	0.60	0.55	[-0.06, 0.11]
Landscaping	0.03	0.16	0.21	0.83	[-0.28, 0.35]
Lane	0.08	0.06	1.40	0.16	[-0.03, 0.19]
Lawn	0.10	0.10	1.01	0.31	[-0.09, 0.29]
Leisure	-0.07	0.09	-0.81	0.42	[-0.24, 0.1]
Lighting	0.07	0.14	0.52	0.60	[-0.2, 0.34]
Line	-0.17	0.12	-1.37	0.17	[-0.41, 0.07]
Machine	-0.23	0.13	-1.73	0.08	[-0.49, 0.03]
Manor House	0.40	0.13	3.01	0.00	[0.14, 0.66]
Meadow	0.12	0.09	1.27	0.20	[-0.06, 0.29]
Metropolis	0.05	0.11	0.45	0.65	[-0.17, 0.28]
Metropolitan Area	-0.09	0.08	-1.20	0.23	[-0.25, 0.06]
Mixed Use	-0.05	0.05	-0.92	0.36	[-0.15, 0.06]
Mode Otransport	-0.04	0.06	-0.71	0.48	[-0.16, 0.08]
Monochrome	0.33	0.13	2.53	0.01	[0.07, 0.58]
Monument	0.37	0.16	2.32	0.02	[0.06, 0.69]
Morning	-0.02	0.10	-0.19	0.85	[-0.21, 0.18]
Motor Vehicle	-0.17	0.05	-3.44	0.00	[-0.26, -0.07]

Mountain	1.14	0.16	7.00	0.00	[0.82, 1.46]
Natural Environment	0.02	0.12	0.18	0.86	[-0.22, 0.26]
Natural Landscape	0.33	0.06	5.05	0.00	[0.2, 0.45]
Neighbourhood	0.05	0.04	1.21	0.23	[-0.03, 0.14]
Nonbuilding Structure	-0.13	0.09	-1.49	0.14	[-0.3, 0.04]
Overhead Power Line	-0.21	0.08	-2.64	0.01	[-0.37, -0.06]
Overpass	-0.27	0.19	-1.42	0.15	[-0.64, 0.1]
Parking	-0.07	0.06	-1.17	0.24	[-0.18, 0.05]
Parking Lot	-0.21	0.11	-1.86	0.06	[-0.43, 0.01]
Passenger Car	-0.27	0.17	-1.58	0.11	[-0.62, 0.07]
Pasture	-0.08	0.10	-0.83	0.40	[-0.28, 0.11]
Pedestrian	0.01	0.10	0.10	0.92	[-0.19, 0.21]
Personal Luxury Car	0.01	0.12	0.07	0.94	[-0.23, 0.24]
Place Oworship	-0.21	0.22	-0.96	0.34	[-0.63, 0.22]
Plain	0.08	0.08	0.91	0.36	[-0.09, 0.24]
Plant	0.07	0.04	1.78	0.08	[-0.01, 0.14]
Plant Community	0.36	0.14	2.47	0.01	[0.07, 0.64]
Pole	0.01	0.06	0.11	0.91	[-0.1, 0.12]
Pollution	-0.12	0.16	-0.75	0.45	[-0.45, 0.2]
Power Station	-0.32	0.20	-1.58	0.11	[-0.72, 0.08]
Prairie	0.13	0.10	1.34	0.18	[-0.06, 0.32]
Property	0.02	0.06	0.34	0.73	[-0.09, 0.13]
Public Space	-0.08	0.08	-0.95	0.34	[-0.24, 0.08]
Public Transport	0.03	0.12	0.24	0.81	[-0.21, 0.27]
Public Utility	0.01	0.06	0.21	0.83	[-0.11, 0.13]
Railway	0.09	0.12	0.78	0.43	[-0.14, 0.32]
Real Estate	-0.15	0.05	-2.91	0.00	[-0.25, -0.05]
Recreation	0.11	0.11	0.97	0.33	[-0.11, 0.32]
Reflection	-0.07	0.13	-0.57	0.57	[-0.33, 0.18]
Reservoir	-0.38	0.13	-3.00	0.00	[-0.63, -0.13]
Residential Area	-0.04	0.04	-1.08	0.28	[-0.12, 0.03]
Retail	-0.19	0.13	-1.50	0.13	[-0.44, 0.06]
Riparian Zone	0.28	0.16	1.80	0.07	[-0.03, 0.59]
Road	-0.01	0.04	-0.17	0.86	[-0.08, 0.07]
Road Surface	0.11	0.05	2.39	0.02	[0.02, 0.21]
Rock	0.45	0.17	2.68	0.01	[0.12, 0.77]
Rolling	-0.14	0.08	-1.88	0.06	[-0.29, 0.01]
Rural Area	0.07	0.05	1.54	0.12	[-0.02, 0.16]
Shade	-0.02	0.07	-0.25	0.80	[-0.15, 0.12]
Shoulder	-0.15	0.12	-1.26	0.21	[-0.38, 0.08]
Shrub	0.02	0.07	0.33	0.74	[-0.12, 0.17]
Sidewalk	-0.07	0.05	-1.48	0.14	[-0.17, 0.02]
Sky	0.02	0.08	0.30	0.76	[-0.13, 0.18]
Slope	0.03	0.07	0.38	0.71	[-0.12, 0.18]
Snow	0.46	0.12	3.72	0.00	[0.22, 0.7]
Soil	-0.15	0.08	-1.90	0.06	[-0.3, 0]
Sport Venue	-0.22	0.17	-1.30	0.19	[-0.55, 0.11]
Stadium	-0.21	0.19	-1.11	0.27	[-0.57, 0.16]

Street	0.09	0.05	1.78	0.07	[-0.01, 0.19]
Street Light	-0.10	0.04	-2.74	0.01	[-0.17, -0.03]
Suburb	0.00	0.05	0.06	0.95	[-0.1, 0.11]
Sunlight	0.20	0.13	1.48	0.14	[-0.06, 0.46]
Tar	-0.15	0.05	-3.17	0.00	[-0.25, -0.06]
Thoroughfare	0.04	0.04	1.03	0.30	[-0.04, 0.13]
Tints And Shades	0.11	0.06	1.70	0.09	[-0.02, 0.24]
Tire	-0.02	0.05	-0.36	0.72	[-0.12, 0.08]
Tower	0.14	0.09	1.61	0.11	[-0.03, 0.31]
Tower Block	-0.27	0.12	-2.26	0.02	[-0.51, -0.04]
Trail	0.15	0.17	0.90	0.37	[-0.18, 0.47]
Train	0.32	0.16	2.05	0.04	[0.01, 0.63]
Train Station	0.08	0.16	0.51	0.61	[-0.23, 0.4]
Transmission Tower	-0.35	0.19	-1.84	0.07	[-0.72, 0.02]
Transport Hub	-0.08	0.14	-0.62	0.54	[-0.35, 0.18]
Travel	-0.10	0.11	-0.90	0.37	[-0.32, 0.12]
Tree	0.01	0.04	0.39	0.70	[-0.06, 0.09]
Truck	-0.11	0.09	-1.34	0.18	[-0.28, 0.05]
Trunk	0.42	0.14	2.89	0.00	[0.14, 0.7]
Twig	-0.01	0.11	-0.09	0.93	[-0.23, 0.21]
Urban Area	-0.07	0.08	-0.87	0.39	[-0.23, 0.09]
Urban Design	-0.07	0.04	-1.83	0.07	[-0.15, 0.01]
Van	0.04	0.14	0.31	0.75	[-0.24, 0.33]
Vegetation	0.06	0.12	0.53	0.60	[-0.17, 0.3]
Vehicle	-0.04	0.05	-0.65	0.52	[-0.14, 0.07]
Vehicle Registration Plate	-0.03	0.06	-0.56	0.58	[-0.14, 0.08]
Walkway	0.26	0.14	1.87	0.06	[-0.01, 0.53]
Water	0.19	0.09	2.10	0.04	[0.01, 0.36]
Water Resources	0.02	0.13	0.15	0.88	[-0.23, 0.27]
Water Transportation	-0.35	0.23	-1.52	0.13	[-0.81, 0.1]
Watercourse	0.40	0.12	3.28	0.00	[0.16, 0.64]
Waterway	-0.19	0.11	-1.76	0.08	[-0.39, 0.02]
Wetland	-0.26	0.16	-1.69	0.09	[-0.57, 0.04]
Winter	-0.20	0.10	-1.92	0.05	[-0.4, 0]
Wood	0.00	0.06	0.07	0.94	[-0.1, 0.11]
Woody Plant	0.13	0.11	1.16	0.25	[-0.09, 0.36]

4.2 Grade of Listed Building

Table S175: Complete regression results.

term	estimate	std.error	statistic	p.value	conf
Grade I	1.19	0.15	7.85	0.00	[0.89, 1.49]
Grade II*	1.00	0.12	8.46	0.00	[0.77, 1.23]
Grade II	0.50	0.06	8.90	0.00	[0.39, 0.6]
Agriculture	0.27	0.11	2.50	0.01	[0.06, 0.48]

Apartment	-0.01	0.08	-0.10	0.92	[-0.17, 0.15]
Asphalt	-0.27	0.05	-5.67	0.00	[-0.36, -0.17]
Atmospheric Phenomenon	-0.17	0.12	-1.36	0.17	[-0.41, 0.07]
Automotive Design	-0.22	0.11	-1.99	0.05	[-0.43, 0]
Automotive Exterior	-0.05	0.06	-0.88	0.38	[-0.17, 0.07]
Automotive Lighting	-0.09	0.06	-1.51	0.13	[-0.22, 0.03]
Automotive Parking Light	-0.20	0.06	-3.05	0.00	[-0.32, -0.07]
Automotive Tail Brake Light	0.09	0.13	0.70	0.49	[-0.16, 0.33]
Automotive Tire	0.02	0.09	0.20	0.84	[-0.15, 0.19]
Automotive Wheel System	-0.02	0.13	-0.16	0.88	[-0.28, 0.24]
Biome	0.35	0.17	2.11	0.04	[0.02, 0.68]
Boat	1.15	0.18	6.34	0.00	[0.8, 1.51]
Body Owater	0.19	0.13	1.43	0.15	[-0.07, 0.45]
Branch	0.28	0.11	2.55	0.01	[0.06, 0.49]
Bridge	0.00	0.11	0.00	1.00	[-0.21, 0.21]
Building	-0.21	0.04	-4.85	0.00	[-0.3, -0.13]
Building Material	-0.10	0.14	-0.75	0.45	[-0.38, 0.17]
Bus	0.13	0.12	1.06	0.29	[-0.11, 0.37]
Canal	0.20	0.13	1.49	0.14	[-0.06, 0.46]
Car	0.11	0.06	1.93	0.05	[0, 0.23]
Channel	0.14	0.14	0.97	0.33	[-0.14, 0.41]
Chapel	0.41	0.18	2.30	0.02	[0.06, 0.75]
Church	0.38	0.14	2.70	0.01	[0.1, 0.65]
City	-0.12	0.04	-2.87	0.00	[-0.2, -0.04]
Cloud	-0.08	0.03	-2.69	0.01	[-0.14, -0.02]
Commercial Building	-0.25	0.06	-4.02	0.00	[-0.37, -0.13]
Concrete Bridge	-0.20	0.17	-1.20	0.23	[-0.54, 0.13]
Condominium	0.11	0.11	1.04	0.30	[-0.1, 0.33]
Cottage	0.36	0.05	6.67	0.00	[0.26, 0.47]
Cumulus	-0.06	0.07	-0.81	0.42	[-0.2, 0.08]
Daytime	-0.01	0.13	-0.06	0.95	[-0.27, 0.26]
Dirt Road	0.09	0.16	0.54	0.59	[-0.23, 0.4]
Downtown	0.14	0.10	1.37	0.17	[-0.06, 0.35]
Driveway	0.19	0.14	1.39	0.17	[-0.08, 0.46]
Dusk	0.20	0.15	1.32	0.19	[-0.1, 0.5]
Ecoregion	0.30	0.13	2.41	0.02	[0.06, 0.55]
Electrical Supply	-0.04	0.13	-0.32	0.75	[-0.3, 0.22]
Electricity	-0.06	0.06	-0.99	0.32	[-0.19, 0.06]
Engineering	0.02	0.13	0.13	0.90	[-0.23, 0.26]
Evening	-0.16	0.12	-1.40	0.16	[-0.39, 0.07]
Event	-0.15	0.08	-1.76	0.08	[-0.31, 0.02]
Family Car	-0.02	0.12	-0.15	0.88	[-0.26, 0.22]
Field	-0.08	0.10	-0.78	0.43	[-0.27, 0.12]
Fixture	-0.01	0.07	-0.22	0.83	[-0.15, 0.12]
Flower	0.20	0.14	1.46	0.14	[-0.07, 0.46]
Fluvial Landforms Ostreams	0.19	0.14	1.39	0.16	[-0.08, 0.46]
Font	-0.21	0.13	-1.66	0.10	[-0.46, 0.04]
Forest	0.69	0.12	5.93	0.00	[0.46, 0.92]

Freeway	-0.15	0.14	-1.10	0.27	[-0.42, 0.12]
Garden	0.03	0.15	0.23	0.82	[-0.26, 0.33]
Gas	-0.21	0.05	-4.45	0.00	[-0.3, -0.12]
Girder Bridge	0.29	0.15	1.86	0.06	[-0.02, 0.59]
Grass	-0.06	0.05	-1.40	0.16	[-0.15, 0.03]
Grass Family	0.09	0.09	1.06	0.29	[-0.08, 0.27]
Grassland	0.07	0.08	0.91	0.36	[-0.09, 0.23]
Groundcover	0.02	0.08	0.29	0.77	[-0.14, 0.19]
Headquarters	0.01	0.11	0.13	0.89	[-0.2, 0.23]
Highland	0.33	0.14	2.38	0.02	[0.06, 0.6]
Highway	-0.23	0.11	-2.03	0.04	[-0.46, -0.01]
Hill	0.11	0.09	1.22	0.22	[-0.07, 0.3]
Home	0.04	0.10	0.41	0.68	[-0.15, 0.24]
Horizon	0.19	0.07	2.87	0.00	[0.06, 0.32]
House	0.14	0.04	4.08	0.00	[0.07, 0.21]
Human Settlement	0.12	0.09	1.40	0.16	[-0.05, 0.29]
Industry	0.30	0.18	1.67	0.10	[-0.05, 0.64]
Infrastructure	-0.03	0.05	-0.65	0.52	[-0.13, 0.07]
Intercept	2.58	0.12	20.86	0.00	[2.33, 2.82]
Lacustrine Plain	0.04	0.14	0.32	0.75	[-0.22, 0.31]
Lake	0.57	0.11	5.03	0.00	[0.35, 0.8]
Land Lot	-0.24	0.04	-5.49	0.00	[-0.33, -0.16]
Land Vehicle	0.07	0.06	1.10	0.27	[-0.05, 0.19]
Landmark	0.11	0.09	1.27	0.20	[-0.06, 0.28]
Landscape	0.03	0.04	0.63	0.53	[-0.06, 0.11]
Landscaping	0.04	0.16	0.27	0.79	[-0.27, 0.35]
Lane	0.09	0.06	1.51	0.13	[-0.03, 0.2]
Lawn	0.09	0.10	0.96	0.34	[-0.1, 0.28]
Leisure	-0.06	0.09	-0.63	0.53	[-0.23, 0.12]
Lighting	0.07	0.14	0.54	0.59	[-0.19, 0.34]
Line	-0.17	0.12	-1.39	0.16	[-0.41, 0.07]
Machine	-0.20	0.13	-1.53	0.13	[-0.47, 0.06]
Manor House	0.40	0.13	3.00	0.00	[0.14, 0.66]
Meadow	0.12	0.09	1.28	0.20	[-0.06, 0.29]
Metropolis	0.06	0.11	0.53	0.59	[-0.16, 0.29]
Metropolitan Area	-0.10	0.08	-1.27	0.21	[-0.25, 0.05]
Mixed Use	-0.04	0.05	-0.75	0.45	[-0.14, 0.06]
Mode Otransport	-0.04	0.06	-0.69	0.49	[-0.16, 0.08]
Monochrome	0.32	0.13	2.51	0.01	[0.07, 0.57]
Monument	0.42	0.16	2.63	0.01	[0.11, 0.73]
Morning	-0.02	0.10	-0.23	0.82	[-0.22, 0.17]
Motor Vehicle	-0.17	0.05	-3.47	0.00	[-0.26, -0.07]
Mountain	1.17	0.16	7.19	0.00	[0.85, 1.48]
Natural Environment	0.03	0.12	0.24	0.81	[-0.21, 0.27]
Natural Landscape	0.33	0.06	5.17	0.00	[0.21, 0.46]
Neighbourhood	0.06	0.04	1.37	0.17	[-0.03, 0.15]
Nonbuilding Structure	-0.12	0.09	-1.42	0.16	[-0.29, 0.05]
Overhead Power Line	-0.21	0.08	-2.64	0.01	[-0.37, -0.06]

Overpass	-0.30	0.19	-1.60	0.11	[-0.67, 0.07]
Parking	-0.07	0.06	-1.12	0.26	[-0.18, 0.05]
Parking Lot	-0.21	0.11	-1.85	0.06	[-0.43, 0.01]
Passenger Car	-0.29	0.17	-1.69	0.09	[-0.63, 0.05]
Pasture	-0.08	0.10	-0.79	0.43	[-0.27, 0.12]
Pedestrian	0.03	0.10	0.27	0.79	[-0.17, 0.23]
Personal Luxury Car	0.00	0.12	0.00	1.00	[-0.23, 0.23]
Place Oworship	-0.31	0.22	-1.43	0.15	[-0.74, 0.12]
Plain	0.08	0.08	0.92	0.36	[-0.09, 0.24]
Plant	0.07	0.04	1.75	0.08	[-0.01, 0.14]
Plant Community	0.35	0.14	2.45	0.01	[0.07, 0.63]
Pole	0.01	0.06	0.15	0.88	[-0.1, 0.12]
Pollution	-0.11	0.16	-0.64	0.52	[-0.43, 0.22]
Power Station	-0.31	0.20	-1.51	0.13	[-0.7, 0.09]
Prairie	0.13	0.10	1.32	0.19	[-0.06, 0.32]
Property	0.03	0.06	0.55	0.58	[-0.08, 0.14]
Public Space	-0.10	0.08	-1.17	0.24	[-0.26, 0.06]
Public Transport	0.03	0.12	0.26	0.80	[-0.21, 0.27]
Public Utility	0.01	0.06	0.14	0.89	[-0.11, 0.13]
Railway	0.09	0.12	0.78	0.44	[-0.14, 0.32]
Real Estate	-0.15	0.05	-2.97	0.00	[-0.26, -0.05]
Recreation	0.09	0.11	0.86	0.39	[-0.12, 0.31]
Reflection	-0.09	0.13	-0.73	0.47	[-0.35, 0.16]
Reservoir	-0.41	0.13	-3.30	0.00	[-0.66, -0.17]
Residential Area	-0.04	0.04	-0.99	0.32	[-0.12, 0.04]
Retail	-0.19	0.13	-1.48	0.14	[-0.44, 0.06]
Riparian Zone	0.27	0.16	1.72	0.09	[-0.04, 0.58]
Road	-0.01	0.04	-0.13	0.89	[-0.08, 0.07]
Road Surface	0.13	0.05	2.66	0.01	[0.03, 0.22]
Rock	0.46	0.17	2.80	0.01	[0.14, 0.79]
Rolling	-0.14	0.08	-1.82	0.07	[-0.29, 0.01]
Rural Area	0.07	0.05	1.59	0.11	[-0.02, 0.16]
Shade	-0.01	0.07	-0.16	0.88	[-0.14, 0.12]
Shoulder	-0.15	0.12	-1.27	0.21	[-0.38, 0.08]
Shrub	0.03	0.07	0.39	0.70	[-0.12, 0.17]
Sidewalk	-0.06	0.05	-1.32	0.19	[-0.16, 0.03]
Sky	0.03	0.08	0.42	0.67	[-0.12, 0.19]
Slope	0.02	0.07	0.30	0.77	[-0.12, 0.17]
Snow	0.44	0.12	3.56	0.00	[0.2, 0.68]
Soil	-0.15	0.08	-1.87	0.06	[-0.3, 0.01]
Sport Venue	-0.22	0.17	-1.28	0.20	[-0.55, 0.12]
Stadium	-0.20	0.19	-1.09	0.28	[-0.57, 0.16]
Street	0.08	0.05	1.64	0.10	[-0.02, 0.18]
Street Light	-0.10	0.04	-2.76	0.01	[-0.17, -0.03]
Suburb	0.01	0.05	0.10	0.92	[-0.1, 0.11]
Sunlight	0.19	0.13	1.39	0.16	[-0.08, 0.45]
Tar	-0.16	0.05	-3.27	0.00	[-0.25, -0.06]
Thoroughfare	0.05	0.04	1.11	0.27	[-0.04, 0.13]

Tints And Shades	0.11	0.06	1.74	0.08	[-0.01, 0.24]
Tire	-0.02	0.05	-0.36	0.72	[-0.12, 0.08]
Tower	0.11	0.09	1.31	0.19	[-0.06, 0.28]
Tower Block	-0.27	0.12	-2.24	0.03	[-0.51, -0.03]
Trail	0.14	0.16	0.85	0.40	[-0.18, 0.46]
Train	0.34	0.16	2.18	0.03	[0.03, 0.65]
Train Station	0.10	0.16	0.60	0.55	[-0.22, 0.41]
Transmission Tower	-0.34	0.19	-1.80	0.07	[-0.7, 0.03]
Transport Hub	-0.09	0.13	-0.63	0.53	[-0.35, 0.18]
Travel	-0.10	0.11	-0.89	0.38	[-0.32, 0.12]
Tree	0.01	0.04	0.33	0.74	[-0.06, 0.08]
Truck	-0.12	0.09	-1.38	0.17	[-0.28, 0.05]
Trunk	0.40	0.14	2.77	0.01	[0.12, 0.68]
Twig	-0.01	0.11	-0.11	0.91	[-0.23, 0.21]
Urban Area	-0.08	0.08	-1.00	0.32	[-0.24, 0.08]
Urban Design	-0.07	0.04	-1.89	0.06	[-0.15, 0]
Van	0.06	0.14	0.39	0.70	[-0.22, 0.34]
Vegetation	0.07	0.12	0.56	0.57	[-0.17, 0.3]
Vehicle	-0.04	0.05	-0.66	0.51	[-0.14, 0.07]
Vehicle Registration Plate	-0.03	0.06	-0.56	0.57	[-0.14, 0.08]
Walkway	0.24	0.14	1.72	0.08	[-0.03, 0.51]
Water	0.20	0.09	2.26	0.02	[0.03, 0.37]
Water Resources	0.02	0.13	0.19	0.85	[-0.23, 0.28]
Water Transportation	-0.36	0.23	-1.54	0.12	[-0.81, 0.1]
Watercourse	0.40	0.12	3.33	0.00	[0.17, 0.64]
Waterway	-0.20	0.11	-1.92	0.05	[-0.41, 0]
Wetland	-0.25	0.16	-1.59	0.11	[-0.55, 0.06]
Winter	-0.20	0.10	-1.92	0.06	[-0.4, 0]
Wood	0.00	0.06	0.01	1.00	[-0.11, 0.11]
Woody Plant	0.12	0.11	1.04	0.30	[-0.11, 0.34]

4.3 Date of Listing

Table S176: Complete regression results.

term	estimate	std.error	statistic	p.value	conf
Year Group[1949,1966)	0.95	0.09	10.15	0.00	[0.77, 1.14]
Year Group[1966,1976)	0.62	0.09	6.81	0.00	[0.44, 0.8]
Year Group[1976,1987)	0.57	0.09	6.16	0.00	[0.39, 0.75]
Year Group[1987,2023]	0.38	0.09	4.11	0.00	[0.2, 0.57]
Agriculture	0.26	0.11	2.47	0.01	[0.05, 0.47]
Apartment	-0.02	0.08	-0.19	0.85	[-0.17, 0.14]
Asphalt	-0.26	0.05	-5.60	0.00	[-0.36, -0.17]
Atmospheric Phenomenon	-0.17	0.12	-1.38	0.17	[-0.41, 0.07]
Automotive Design	-0.22	0.11	-2.03	0.04	[-0.43, -0.01]
Automotive Exterior	-0.06	0.06	-0.96	0.34	[-0.18, 0.06]

Automotive Lighting	-0.10	0.06	-1.54	0.12	[-0.22, 0.03]
Automotive Parking Light	-0.20	0.06	-3.06	0.00	[-0.32, -0.07]
Automotive Tail Brake Light	0.07	0.13	0.57	0.57	[-0.17, 0.32]
Automotive Tire	0.02	0.09	0.19	0.85	[-0.15, 0.19]
Automotive Wheel System	-0.02	0.13	-0.15	0.88	[-0.28, 0.24]
Biome	0.34	0.17	2.07	0.04	[0.02, 0.67]
Boat	1.16	0.18	6.37	0.00	[0.8, 1.52]
Body Owater	0.18	0.13	1.37	0.17	[-0.08, 0.45]
Branch	0.28	0.11	2.51	0.01	[0.06, 0.49]
Bridge	-0.02	0.11	-0.16	0.88	[-0.23, 0.19]
Building	-0.21	0.04	-4.77	0.00	[-0.3, -0.12]
Building Material	-0.10	0.14	-0.71	0.48	[-0.37, 0.17]
Bus	0.12	0.12	0.98	0.33	[-0.12, 0.36]
Canal	0.18	0.13	1.35	0.18	[-0.08, 0.44]
Car	0.11	0.06	1.95	0.05	[0, 0.23]
Channel	0.13	0.14	0.95	0.34	[-0.14, 0.41]
Chapel	0.37	0.18	2.08	0.04	[0.02, 0.72]
Church	0.45	0.14	3.23	0.00	[0.18, 0.73]
City	-0.12	0.04	-2.92	0.00	[-0.2, -0.04]
Cloud	-0.08	0.03	-2.62	0.01	[-0.14, -0.02]
Commercial Building	-0.25	0.06	-3.95	0.00	[-0.37, -0.12]
Concrete Bridge	-0.20	0.17	-1.15	0.25	[-0.53, 0.14]
Condominium	0.12	0.11	1.06	0.29	[-0.1, 0.33]
Cottage	0.36	0.05	6.51	0.00	[0.25, 0.46]
Cumulus	-0.06	0.07	-0.83	0.41	[-0.2, 0.08]
Daytime	0.00	0.14	0.03	0.97	[-0.26, 0.27]
Dirt Road	0.09	0.16	0.58	0.56	[-0.22, 0.41]
Downtown	0.15	0.10	1.45	0.15	[-0.05, 0.36]
Driveway	0.20	0.14	1.43	0.15	[-0.07, 0.46]
Dusk	0.21	0.15	1.37	0.17	[-0.09, 0.51]
Ecoregion	0.30	0.13	2.40	0.02	[0.05, 0.55]
Electrical Supply	-0.05	0.13	-0.37	0.71	[-0.31, 0.21]
Electricity	-0.06	0.06	-0.99	0.32	[-0.19, 0.06]
Engineering	0.03	0.13	0.23	0.82	[-0.22, 0.27]
Evening	-0.16	0.12	-1.39	0.17	[-0.39, 0.07]
Event	-0.13	0.08	-1.54	0.12	[-0.29, 0.03]
Family Car	-0.02	0.12	-0.13	0.90	[-0.26, 0.22]
Field	-0.08	0.10	-0.81	0.42	[-0.27, 0.11]
Fixture	-0.02	0.07	-0.29	0.77	[-0.15, 0.11]
Flower	0.19	0.14	1.38	0.17	[-0.08, 0.45]
Fluvial Landforms Ostreams	0.19	0.14	1.37	0.17	[-0.08, 0.46]
Font	-0.20	0.13	-1.60	0.11	[-0.45, 0.05]
Forest	0.68	0.12	5.83	0.00	[0.45, 0.91]
Freeway	-0.15	0.14	-1.06	0.29	[-0.42, 0.12]
Garden	0.03	0.15	0.22	0.83	[-0.26, 0.33]
Gas	-0.21	0.05	-4.49	0.00	[-0.3, -0.12]
Girder Bridge	0.30	0.15	1.92	0.05	[-0.01, 0.6]
Grass	-0.06	0.05	-1.24	0.21	[-0.15, 0.03]

Grass Family	0.09	0.09	1.01	0.31	[-0.08, 0.26]
Grassland	0.07	0.08	0.86	0.39	[-0.09, 0.23]
Groundcover	0.02	0.08	0.25	0.81	[-0.14, 0.19]
Headquarters	0.00	0.11	0.00	1.00	[-0.22, 0.22]
Highland	0.32	0.14	2.34	0.02	[0.05, 0.59]
Highway	-0.24	0.11	-2.08	0.04	[-0.46, -0.01]
Hill	0.11	0.09	1.20	0.23	[-0.07, 0.3]
Home	0.03	0.10	0.29	0.77	[-0.17, 0.23]
Horizon	0.19	0.07	2.89	0.00	[0.06, 0.32]
House	0.14	0.04	4.05	0.00	[0.07, 0.21]
Human Settlement	0.14	0.09	1.60	0.11	[-0.03, 0.3]
Industry	0.29	0.18	1.60	0.11	[-0.06, 0.63]
Infrastructure	-0.03	0.05	-0.57	0.57	[-0.13, 0.07]
Intercept	2.59	0.12	20.95	0.00	[2.35, 2.83]
Lacustrine Plain	0.03	0.14	0.19	0.85	[-0.24, 0.29]
Lake	0.58	0.11	5.07	0.00	[0.35, 0.8]
Land Lot	-0.25	0.04	-5.61	0.00	[-0.34, -0.16]
Land Vehicle	0.07	0.06	1.05	0.29	[-0.06, 0.19]
Landmark	0.11	0.09	1.27	0.20	[-0.06, 0.28]
Landscape	0.03	0.04	0.69	0.49	[-0.05, 0.11]
Landscaping	0.04	0.16	0.27	0.79	[-0.27, 0.35]
Lane	0.08	0.06	1.31	0.19	[-0.04, 0.19]
Lawn	0.10	0.10	1.01	0.31	[-0.09, 0.29]
Leisure	-0.07	0.09	-0.75	0.45	[-0.24, 0.11]
Lighting	0.08	0.14	0.58	0.56	[-0.19, 0.34]
Line	-0.18	0.12	-1.48	0.14	[-0.42, 0.06]
Machine	-0.22	0.13	-1.65	0.10	[-0.48, 0.04]
Manor House	0.39	0.13	2.96	0.00	[0.13, 0.65]
Meadow	0.12	0.09	1.32	0.19	[-0.06, 0.3]
Metropolis	0.05	0.11	0.46	0.65	[-0.17, 0.28]
Metropolitan Area	-0.09	0.08	-1.18	0.24	[-0.25, 0.06]
Mixed Use	-0.05	0.05	-0.94	0.35	[-0.15, 0.05]
Mode Otransport	-0.04	0.06	-0.71	0.48	[-0.16, 0.08]
Monochrome	0.32	0.13	2.51	0.01	[0.07, 0.58]
Monument	0.43	0.16	2.65	0.01	[0.11, 0.74]
Morning	-0.01	0.10	-0.10	0.92	[-0.2, 0.19]
Motor Vehicle	-0.17	0.05	-3.57	0.00	[-0.27, -0.08]
Mountain	1.16	0.16	7.11	0.00	[0.84, 1.47]
Natural Environment	0.03	0.12	0.23	0.82	[-0.21, 0.26]
Natural Landscape	0.33	0.06	5.07	0.00	[0.2, 0.45]
Neighbourhood	0.05	0.04	1.17	0.24	[-0.04, 0.14]
Nonbuilding Structure	-0.13	0.09	-1.47	0.14	[-0.3, 0.04]
Overhead Power Line	-0.22	0.08	-2.66	0.01	[-0.37, -0.06]
Overpass	-0.29	0.19	-1.52	0.13	[-0.66, 0.08]
Parking	-0.06	0.06	-1.10	0.27	[-0.18, 0.05]
Parking Lot	-0.21	0.11	-1.86	0.06	[-0.43, 0.01]
Passenger Car	-0.29	0.17	-1.70	0.09	[-0.63, 0.05]
Pasture	-0.08	0.10	-0.85	0.40	[-0.28, 0.11]

Pedestrian	0.01	0.10	0.14	0.89	[-0.19, 0.21]
Personal Luxury Car	0.01	0.12	0.08	0.94	[-0.23, 0.24]
Place Oworship	-0.34	0.22	-1.53	0.13	[-0.76, 0.09]
Plain	0.08	0.08	0.93	0.35	[-0.09, 0.24]
Plant	0.07	0.04	1.74	0.08	[-0.01, 0.14]
Plant Community	0.35	0.14	2.44	0.01	[0.07, 0.63]
Pole	0.01	0.06	0.12	0.90	[-0.1, 0.12]
Pollution	-0.11	0.16	-0.70	0.49	[-0.43, 0.21]
Power Station	-0.31	0.20	-1.53	0.12	[-0.71, 0.09]
Prairie	0.13	0.10	1.34	0.18	[-0.06, 0.32]
Property	0.02	0.06	0.30	0.77	[-0.09, 0.13]
Public Space	-0.08	0.08	-1.02	0.31	[-0.24, 0.08]
Public Transport	0.04	0.12	0.36	0.72	[-0.2, 0.29]
Public Utility	0.01	0.06	0.23	0.82	[-0.1, 0.13]
Railway	0.10	0.12	0.81	0.42	[-0.14, 0.33]
Real Estate	-0.15	0.05	-2.96	0.00	[-0.26, -0.05]
Recreation	0.10	0.11	0.94	0.35	[-0.11, 0.32]
Reflection	-0.09	0.13	-0.71	0.48	[-0.35, 0.16]
Reservoir	-0.40	0.13	-3.15	0.00	[-0.64, -0.15]
Residential Area	-0.04	0.04	-1.01	0.31	[-0.12, 0.04]
Retail	-0.19	0.13	-1.45	0.15	[-0.44, 0.06]
Riparian Zone	0.29	0.16	1.86	0.06	[-0.02, 0.6]
Road	-0.01	0.04	-0.22	0.82	[-0.09, 0.07]
Road Surface	0.12	0.05	2.53	0.01	[0.03, 0.21]
Rock	0.45	0.17	2.72	0.01	[0.13, 0.78]
Rolling	-0.14	0.08	-1.82	0.07	[-0.29, 0.01]
Rural Area	0.07	0.05	1.62	0.11	[-0.02, 0.16]
Shade	-0.01	0.07	-0.15	0.88	[-0.14, 0.12]
Shoulder	-0.15	0.12	-1.24	0.21	[-0.38, 0.08]
Shrub	0.04	0.07	0.47	0.64	[-0.11, 0.18]
Sidewalk	-0.07	0.05	-1.49	0.14	[-0.17, 0.02]
Sky	0.03	0.08	0.35	0.72	[-0.13, 0.18]
Slope	0.02	0.07	0.33	0.74	[-0.12, 0.17]
Snow	0.45	0.12	3.66	0.00	[0.21, 0.69]
Soil	-0.15	0.08	-1.94	0.05	[-0.3, 0]
Sport Venue	-0.22	0.17	-1.31	0.19	[-0.55, 0.11]
Stadium	-0.21	0.19	-1.10	0.27	[-0.57, 0.16]
Street	0.09	0.05	1.88	0.06	[0, 0.19]
Street Light	-0.10	0.04	-2.75	0.01	[-0.17, -0.03]
Suburb	0.00	0.05	0.00	1.00	[-0.1, 0.1]
Sunlight	0.19	0.13	1.39	0.17	[-0.08, 0.45]
Tar	-0.16	0.05	-3.26	0.00	[-0.25, -0.06]
Thoroughfare	0.05	0.04	1.06	0.29	[-0.04, 0.13]
Tints And Shades	0.11	0.06	1.68	0.09	[-0.02, 0.23]
Tire	-0.02	0.05	-0.29	0.77	[-0.12, 0.09]
Tower	0.14	0.09	1.56	0.12	[-0.03, 0.3]
Tower Block	-0.28	0.12	-2.31	0.02	[-0.52, -0.04]
Trail	0.15	0.17	0.88	0.38	[-0.18, 0.47]

Train	0.33	0.16	2.09	0.04	[0.02, 0.64]
Train Station	0.07	0.16	0.46	0.65	[-0.24, 0.39]
Transmission Tower	-0.35	0.19	-1.85	0.06	[-0.71, 0.02]
Transport Hub	-0.08	0.14	-0.61	0.54	[-0.35, 0.18]
Travel	-0.13	0.11	-1.20	0.23	[-0.36, 0.09]
Tree	0.02	0.04	0.42	0.68	[-0.06, 0.09]
Truck	-0.12	0.09	-1.36	0.17	[-0.28, 0.05]
Trunk	0.40	0.14	2.80	0.01	[0.12, 0.69]
Twig	-0.03	0.11	-0.25	0.80	[-0.25, 0.19]
Urban Area	-0.07	0.08	-0.89	0.37	[-0.23, 0.09]
Urban Design	-0.07	0.04	-1.86	0.06	[-0.15, 0]
Van	0.06	0.14	0.39	0.70	[-0.22, 0.34]
Vegetation	0.07	0.12	0.60	0.55	[-0.16, 0.31]
Vehicle	-0.04	0.05	-0.75	0.45	[-0.15, 0.07]
Vehicle Registration Plate	-0.03	0.06	-0.49	0.62	[-0.14, 0.08]
Walkway	0.25	0.14	1.83	0.07	[-0.02, 0.52]
Water	0.19	0.09	2.14	0.03	[0.02, 0.36]
Water Resources	0.01	0.13	0.09	0.93	[-0.24, 0.26]
Water Transportation	-0.35	0.23	-1.51	0.13	[-0.8, 0.1]
Watercourse	0.41	0.12	3.35	0.00	[0.17, 0.64]
Waterway	-0.20	0.11	-1.85	0.06	[-0.4, 0.01]
Wetland	-0.25	0.16	-1.59	0.11	[-0.55, 0.06]
Winter	-0.20	0.10	-1.97	0.05	[-0.41, 0]
Wood	0.00	0.06	-0.01	0.99	[-0.11, 0.11]
Woody Plant	0.13	0.11	1.10	0.27	[-0.1, 0.35]

4.4 Date of Construction of Listed Building

Table S177: Complete regression results.

term	estimate	std.error	statistic	p.value	conf
Built C15 Or Earlier	1.29	0.16	7.99	0.00	[0.97, 1.6]
Built C16	1.21	0.19	6.50	0.00	[0.84, 1.57]
Built C17	0.81	0.15	5.36	0.00	[0.51, 1.1]
Built C18	0.61	0.12	5.12	0.00	[0.37, 0.84]
Built C19	0.45	0.07	6.28	0.00	[0.31, 0.59]
Built C20	0.51	0.11	4.45	0.00	[0.28, 0.73]
Agriculture	0.27	0.11	2.57	0.01	[0.06, 0.48]
Apartment	0.00	0.08	-0.04	0.97	[-0.16, 0.16]
Asphalt	-0.26	0.05	-5.59	0.00	[-0.36, -0.17]
Atmospheric Phenomenon	-0.16	0.12	-1.31	0.19	[-0.4, 0.08]
Automotive Design	-0.21	0.11	-1.91	0.06	[-0.42, 0.01]
Automotive Exterior	-0.06	0.06	-1.05	0.30	[-0.18, 0.06]
Automotive Lighting	-0.10	0.06	-1.65	0.10	[-0.22, 0.02]
Automotive Parking Light	-0.21	0.06	-3.22	0.00	[-0.33, -0.08]
Automotive Tail Brake Light	0.08	0.13	0.61	0.54	[-0.17, 0.32]

Automotive Tire	0.01	0.09	0.13	0.90	[-0.16, 0.18]
Automotive Wheel System	-0.01	0.13	-0.04	0.97	[-0.26, 0.25]
Biome	0.36	0.17	2.19	0.03	[0.04, 0.69]
Boat	1.14	0.18	6.28	0.00	[0.79, 1.5]
Body Owater	0.20	0.13	1.50	0.13	[-0.06, 0.46]
Branch	0.28	0.11	2.54	0.01	[0.06, 0.49]
Bridge	-0.03	0.11	-0.26	0.79	[-0.24, 0.18]
Building	-0.21	0.04	-4.88	0.00	[-0.3, -0.13]
Building Material	-0.09	0.14	-0.68	0.50	[-0.37, 0.18]
Bus	0.13	0.12	1.04	0.30	[-0.11, 0.37]
Canal	0.18	0.13	1.34	0.18	[-0.08, 0.44]
Car	0.12	0.06	1.98	0.05	[0, 0.23]
Channel	0.12	0.14	0.83	0.40	[-0.16, 0.39]
Chapel	0.42	0.18	2.35	0.02	[0.07, 0.76]
Church	0.38	0.14	2.65	0.01	[0.1, 0.65]
City	-0.12	0.04	-2.88	0.00	[-0.2, -0.04]
Cloud	-0.08	0.03	-2.60	0.01	[-0.14, -0.02]
Commercial Building	-0.26	0.06	-4.08	0.00	[-0.38, -0.13]
Concrete Bridge	-0.19	0.17	-1.12	0.26	[-0.53, 0.14]
Condominium	0.13	0.11	1.24	0.22	[-0.08, 0.35]
Cottage	0.35	0.05	6.48	0.00	[0.25, 0.46]
Cumulus	-0.06	0.07	-0.79	0.43	[-0.2, 0.08]
Daytime	-0.01	0.13	-0.10	0.92	[-0.28, 0.25]
Dirt Road	0.07	0.16	0.41	0.68	[-0.25, 0.38]
Downtown	0.17	0.10	1.62	0.11	[-0.04, 0.37]
Driveway	0.18	0.14	1.33	0.18	[-0.09, 0.45]
Dusk	0.21	0.15	1.40	0.16	[-0.09, 0.51]
Ecoregion	0.31	0.13	2.44	0.01	[0.06, 0.55]
Electrical Supply	-0.04	0.13	-0.32	0.75	[-0.3, 0.22]
Electricity	-0.06	0.06	-0.96	0.34	[-0.19, 0.06]
Engineering	0.03	0.13	0.22	0.82	[-0.22, 0.27]
Evening	-0.15	0.12	-1.24	0.21	[-0.38, 0.08]
Event	-0.13	0.08	-1.52	0.13	[-0.29, 0.04]
Family Car	-0.03	0.12	-0.21	0.83	[-0.27, 0.21]
Field	-0.09	0.10	-0.94	0.35	[-0.28, 0.1]
Fixture	-0.02	0.07	-0.34	0.74	[-0.15, 0.11]
Flower	0.19	0.14	1.44	0.15	[-0.07, 0.46]
Fluvial Landforms Ostreams	0.19	0.14	1.36	0.17	[-0.08, 0.45]
Font	-0.21	0.13	-1.66	0.10	[-0.46, 0.04]
Forest	0.69	0.12	5.94	0.00	[0.47, 0.92]
Freeway	-0.15	0.14	-1.12	0.26	[-0.42, 0.12]
Garden	0.03	0.15	0.20	0.84	[-0.27, 0.33]
Gas	-0.21	0.05	-4.49	0.00	[-0.3, -0.12]
Girder Bridge	0.30	0.15	1.92	0.05	[-0.01, 0.6]
Grass	-0.07	0.05	-1.42	0.16	[-0.16, 0.02]
Grass Family	0.08	0.09	0.92	0.36	[-0.09, 0.25]
Grassland	0.07	0.08	0.92	0.36	[-0.09, 0.24]
Groundcover	0.02	0.08	0.27	0.78	[-0.14, 0.19]

Headquarters	0.02	0.11	0.14	0.89	[-0.2, 0.23]
Highland	0.33	0.14	2.42	0.02	[0.06, 0.6]
Highway	-0.23	0.11	-2.05	0.04	[-0.46, -0.01]
Hill	0.10	0.09	1.08	0.28	[-0.08, 0.29]
Home	0.04	0.10	0.40	0.69	[-0.16, 0.24]
Horizon	0.19	0.07	2.84	0.00	[0.06, 0.32]
House	0.14	0.04	3.94	0.00	[0.07, 0.21]
Human Settlement	0.14	0.09	1.60	0.11	[-0.03, 0.3]
Industry	0.29	0.18	1.61	0.11	[-0.06, 0.63]
Infrastructure	-0.03	0.05	-0.61	0.54	[-0.13, 0.07]
Intercept	2.58	0.12	20.88	0.00	[2.34, 2.82]
Lacustrine Plain	0.03	0.14	0.20	0.84	[-0.24, 0.3]
Lake	0.60	0.11	5.28	0.00	[0.38, 0.83]
Land Lot	-0.25	0.04	-5.57	0.00	[-0.34, -0.16]
Land Vehicle	0.07	0.06	1.15	0.25	[-0.05, 0.2]
Landmark	0.13	0.09	1.56	0.12	[-0.04, 0.3]
Landscape	0.03	0.04	0.58	0.56	[-0.06, 0.11]
Landscaping	0.05	0.16	0.30	0.76	[-0.26, 0.36]
Lane	0.08	0.06	1.40	0.16	[-0.03, 0.19]
Lawn	0.08	0.10	0.78	0.43	[-0.11, 0.26]
Leisure	-0.06	0.09	-0.66	0.51	[-0.23, 0.11]
Lighting	0.07	0.14	0.49	0.63	[-0.2, 0.33]
Line	-0.18	0.12	-1.47	0.14	[-0.42, 0.06]
Machine	-0.22	0.13	-1.64	0.10	[-0.48, 0.04]
Manor House	0.45	0.13	3.37	0.00	[0.19, 0.71]
Meadow	0.13	0.09	1.47	0.14	[-0.04, 0.31]
Metropolis	0.06	0.11	0.53	0.59	[-0.16, 0.29]
Metropolitan Area	-0.09	0.08	-1.14	0.26	[-0.24, 0.06]
Mixed Use	-0.05	0.05	-0.99	0.32	[-0.16, 0.05]
Mode Otransport	-0.04	0.06	-0.67	0.50	[-0.16, 0.08]
Monochrome	0.32	0.13	2.53	0.01	[0.07, 0.58]
Monument	0.43	0.16	2.67	0.01	[0.12, 0.75]
Morning	-0.03	0.10	-0.26	0.79	[-0.22, 0.17]
Motor Vehicle	-0.17	0.05	-3.50	0.00	[-0.26, -0.07]
Mountain	1.17	0.16	7.22	0.00	[0.85, 1.49]
Natural Environment	0.03	0.12	0.24	0.81	[-0.21, 0.26]
Natural Landscape	0.32	0.06	4.96	0.00	[0.19, 0.44]
Neighbourhood	0.06	0.04	1.30	0.19	[-0.03, 0.15]
Nonbuilding Structure	-0.13	0.09	-1.46	0.15	[-0.3, 0.04]
Overhead Power Line	-0.22	0.08	-2.71	0.01	[-0.38, -0.06]
Overpass	-0.28	0.19	-1.49	0.14	[-0.65, 0.09]
Parking	-0.07	0.06	-1.18	0.24	[-0.18, 0.05]
Parking Lot	-0.21	0.11	-1.90	0.06	[-0.43, 0.01]
Passenger Car	-0.29	0.17	-1.69	0.09	[-0.63, 0.05]
Pasture	-0.06	0.10	-0.65	0.51	[-0.26, 0.13]
Pedestrian	0.01	0.10	0.09	0.93	[-0.19, 0.21]
Personal Luxury Car	0.02	0.12	0.13	0.89	[-0.22, 0.25]
Place Oworship	-0.30	0.22	-1.36	0.17	[-0.72, 0.13]

Plain	0.08	0.08	0.90	0.37	[-0.09, 0.24]
Plant	0.07	0.04	1.82	0.07	[-0.01, 0.15]
Plant Community	0.36	0.14	2.53	0.01	[0.08, 0.64]
Pole	0.00	0.06	0.05	0.96	[-0.11, 0.11]
Pollution	-0.12	0.16	-0.71	0.48	[-0.44, 0.2]
Power Station	-0.32	0.20	-1.56	0.12	[-0.71, 0.08]
Prairie	0.13	0.10	1.33	0.18	[-0.06, 0.32]
Property	0.03	0.06	0.46	0.65	[-0.08, 0.14]
Public Space	-0.08	0.08	-0.98	0.33	[-0.24, 0.08]
Public Transport	0.05	0.12	0.42	0.67	[-0.19, 0.29]
Public Utility	0.01	0.06	0.20	0.84	[-0.11, 0.13]
Railway	0.11	0.12	0.92	0.36	[-0.12, 0.34]
Real Estate	-0.16	0.05	-3.02	0.00	[-0.26, -0.05]
Recreation	0.11	0.11	0.99	0.32	[-0.11, 0.32]
Reflection	-0.08	0.13	-0.59	0.56	[-0.33, 0.18]
Reservoir	-0.38	0.13	-2.99	0.00	[-0.62, -0.13]
Residential Area	-0.04	0.04	-1.05	0.29	[-0.12, 0.04]
Retail	-0.18	0.13	-1.44	0.15	[-0.43, 0.07]
Riparian Zone	0.29	0.16	1.81	0.07	[-0.02, 0.59]
Road	0.00	0.04	-0.08	0.94	[-0.08, 0.07]
Road Surface	0.12	0.05	2.51	0.01	[0.03, 0.21]
Rock	0.45	0.17	2.68	0.01	[0.12, 0.78]
Rolling	-0.15	0.08	-1.95	0.05	[-0.3, 0]
Rural Area	0.08	0.05	1.67	0.09	[-0.01, 0.17]
Shade	0.00	0.07	-0.02	0.98	[-0.13, 0.13]
Shoulder	-0.15	0.12	-1.29	0.20	[-0.38, 0.08]
Shrub	0.03	0.07	0.43	0.67	[-0.11, 0.18]
Sidewalk	-0.07	0.05	-1.49	0.14	[-0.17, 0.02]
Sky	0.03	0.08	0.42	0.67	[-0.12, 0.19]
Slope	0.02	0.07	0.31	0.76	[-0.12, 0.17]
Snow	0.47	0.12	3.82	0.00	[0.23, 0.71]
Soil	-0.14	0.08	-1.82	0.07	[-0.29, 0.01]
Sport Venue	-0.22	0.17	-1.30	0.19	[-0.55, 0.11]
Stadium	-0.20	0.19	-1.09	0.27	[-0.57, 0.16]
Street	0.09	0.05	1.83	0.07	[-0.01, 0.19]
Street Light	-0.10	0.04	-2.76	0.01	[-0.17, -0.03]
Suburb	0.00	0.05	0.03	0.98	[-0.1, 0.11]
Sunlight	0.21	0.13	1.56	0.12	[-0.05, 0.47]
Tar	-0.15	0.05	-3.13	0.00	[-0.25, -0.06]
Thoroughfare	0.05	0.04	1.05	0.29	[-0.04, 0.13]
Tints And Shades	0.11	0.06	1.69	0.09	[-0.02, 0.24]
Tire	-0.01	0.05	-0.22	0.83	[-0.11, 0.09]
Tower	0.14	0.09	1.60	0.11	[-0.03, 0.31]
Tower Block	-0.28	0.12	-2.31	0.02	[-0.52, -0.04]
Trail	0.15	0.16	0.93	0.35	[-0.17, 0.48]
Train	0.32	0.16	2.03	0.04	[0.01, 0.63]
Train Station	0.08	0.16	0.52	0.61	[-0.23, 0.4]
Transmission Tower	-0.34	0.19	-1.84	0.07	[-0.71, 0.02]

Transport Hub	-0.10	0.13	-0.71	0.47	[-0.36, 0.17]
Travel	-0.14	0.11	-1.22	0.22	[-0.36, 0.08]
Tree	0.01	0.04	0.26	0.80	[-0.06, 0.08]
Truck	-0.12	0.09	-1.38	0.17	[-0.28, 0.05]
Trunk	0.39	0.14	2.68	0.01	[0.1, 0.67]
Twig	-0.01	0.11	-0.07	0.94	[-0.23, 0.21]
Urban Area	-0.06	0.08	-0.76	0.44	[-0.22, 0.1]
Urban Design	-0.07	0.04	-1.77	0.08	[-0.15, 0.01]
Van	0.06	0.14	0.41	0.68	[-0.22, 0.34]
Vegetation	0.06	0.12	0.52	0.61	[-0.17, 0.3]
Vehicle	-0.03	0.05	-0.60	0.55	[-0.14, 0.07]
Vehicle Registration Plate	-0.03	0.06	-0.58	0.56	[-0.14, 0.08]
Walkway	0.26	0.14	1.85	0.06	[-0.02, 0.53]
Water	0.19	0.09	2.15	0.03	[0.02, 0.36]
Water Resources	0.01	0.13	0.07	0.94	[-0.25, 0.26]
Water Transportation	-0.35	0.23	-1.53	0.13	[-0.81, 0.1]
Watercourse	0.41	0.12	3.37	0.00	[0.17, 0.65]
Waterway	-0.20	0.11	-1.91	0.06	[-0.41, 0.01]
Wetland	-0.26	0.16	-1.70	0.09	[-0.57, 0.04]
Winter	-0.20	0.10	-1.94	0.05	[-0.4, 0]
Wood	0.00	0.06	-0.06	0.95	[-0.11, 0.1]
Woody Plant	0.14	0.11	1.22	0.22	[-0.08, 0.36]

5 Prominence of Listed Building

This analysis looks at the nature of listed building presence in a photograph. ‘Only building (not main subject)’ means there is a single building in the photo but it is not the main subject of the photograph. ‘Only building (main subject)’ refers to photographs where the photograph contains a single building which is the subject of that photograph. ‘One among many listed’ contains a set of listed buildings but no unlisted buildings. ‘One among mixture’ contains more than one listed buildings and one or more unlisted buildings. ‘One among other buildings’ contains one listed building and one or more listed buildings. ‘Marginal / obscured’ technically contains a listed building but it features only marginally or is obscured by another object.

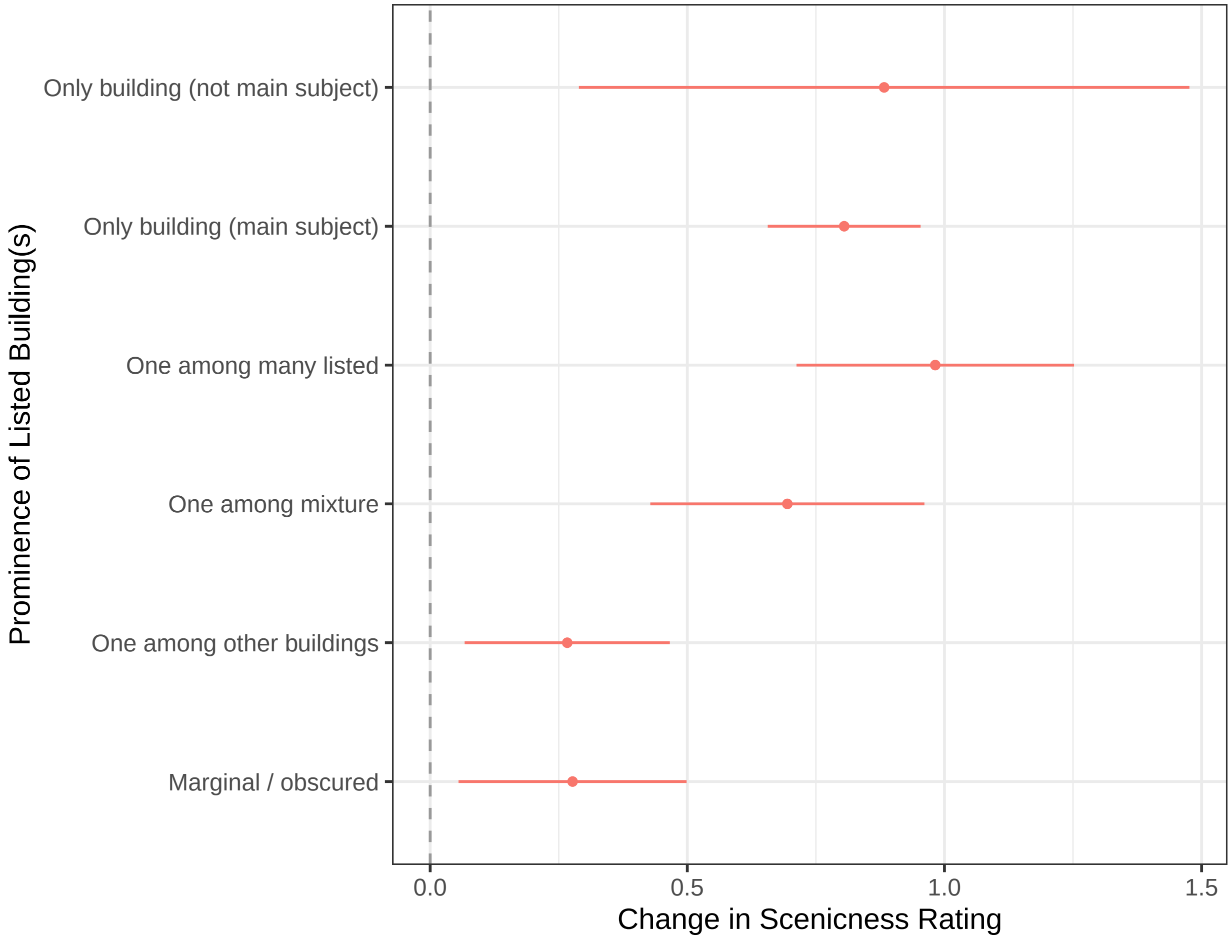

Figure 2: Regression of Scenicness by Prominence of Listed Building

Table S178: Complete regression results.

term	estimate	std.error	statistic	p.value	conf
Only Building (Not Main Subject)	0.88	0.30	2.91	0.00	[0.29, 1.48]
Only Building (Main Subject)	0.81	0.08	10.62	0.00	[0.66, 0.95]
One Among Many Listed	0.98	0.14	7.13	0.00	[0.71, 1.25]
One Among Mixture	0.69	0.14	5.11	0.00	[0.43, 0.96]
One Among Other Buildings	0.27	0.10	2.62	0.01	[0.07, 0.47]
Marginal / Obscured	0.28	0.11	2.45	0.01	[0.06, 0.5]
Agriculture	0.28	0.11	2.62	0.01	[0.07, 0.49]
Apartment	-0.02	0.08	-0.20	0.84	[-0.17, 0.14]
Asphalt	-0.26	0.05	-5.56	0.00	[-0.35, -0.17]
Atmospheric Phenomenon	-0.16	0.12	-1.35	0.18	[-0.4, 0.07]
Automotive Design	-0.22	0.11	-1.98	0.05	[-0.43, 0]
Automotive Exterior	-0.06	0.06	-1.00	0.32	[-0.18, 0.06]
Automotive Lighting	-0.10	0.06	-1.61	0.11	[-0.22, 0.02]
Automotive Parking Light	-0.20	0.06	-3.07	0.00	[-0.32, -0.07]

Automotive Tail Brake Light	0.08	0.13	0.65	0.51	[-0.16, 0.33]
Automotive Tire	0.02	0.09	0.20	0.84	[-0.15, 0.19]
Automotive Wheel System	0.00	0.13	-0.01	0.99	[-0.26, 0.26]
Biome	0.34	0.17	2.02	0.04	[0.01, 0.66]
Boat	1.10	0.18	6.03	0.00	[0.74, 1.46]
Body Owater	0.20	0.13	1.47	0.14	[-0.07, 0.46]
Branch	0.29	0.11	2.65	0.01	[0.08, 0.5]
Bridge	-0.02	0.11	-0.19	0.85	[-0.23, 0.19]
Building	-0.21	0.04	-4.71	0.00	[-0.29, -0.12]
Building Material	-0.10	0.14	-0.72	0.47	[-0.37, 0.17]
Bus	0.14	0.12	1.12	0.26	[-0.1, 0.38]
Canal	0.15	0.13	1.15	0.25	[-0.11, 0.41]
Car	0.12	0.06	2.03	0.04	[0, 0.23]
Channel	0.10	0.14	0.73	0.46	[-0.17, 0.37]
Chapel	0.32	0.18	1.82	0.07	[-0.02, 0.67]
Church	0.42	0.14	3.00	0.00	[0.15, 0.69]
City	-0.12	0.04	-2.92	0.00	[-0.2, -0.04]
Cloud	-0.08	0.03	-2.60	0.01	[-0.14, -0.02]
Commercial Building	-0.26	0.06	-4.20	0.00	[-0.39, -0.14]
Concrete Bridge	-0.25	0.17	-1.44	0.15	[-0.58, 0.09]
Condominium	0.12	0.11	1.06	0.29	[-0.1, 0.33]
Cottage	0.35	0.05	6.49	0.00	[0.25, 0.46]
Cumulus	-0.06	0.07	-0.88	0.38	[-0.2, 0.08]
Daytime	0.00	0.13	0.01	0.99	[-0.26, 0.27]
Dirt Road	0.07	0.16	0.46	0.64	[-0.24, 0.39]
Downtown	0.13	0.10	1.28	0.20	[-0.07, 0.34]
Driveway	0.19	0.14	1.36	0.18	[-0.08, 0.45]
Dusk	0.23	0.15	1.53	0.13	[-0.07, 0.53]
Ecoregion	0.31	0.13	2.46	0.01	[0.06, 0.56]
Electrical Supply	-0.04	0.13	-0.30	0.76	[-0.3, 0.22]
Electricity	-0.07	0.06	-1.07	0.29	[-0.19, 0.06]
Engineering	0.03	0.13	0.26	0.80	[-0.21, 0.28]
Evening	-0.17	0.12	-1.42	0.16	[-0.4, 0.06]
Event	-0.12	0.08	-1.45	0.15	[-0.28, 0.04]
Family Car	-0.01	0.12	-0.05	0.96	[-0.25, 0.23]
Field	-0.09	0.10	-0.95	0.34	[-0.29, 0.1]
Fixture	-0.01	0.07	-0.10	0.92	[-0.14, 0.12]
Flower	0.17	0.14	1.24	0.22	[-0.1, 0.43]
Fluvial Landforms Ostreams	0.19	0.14	1.35	0.18	[-0.08, 0.45]
Font	-0.21	0.13	-1.64	0.10	[-0.45, 0.04]
Forest	0.68	0.12	5.84	0.00	[0.45, 0.91]
Freeway	-0.15	0.14	-1.12	0.26	[-0.42, 0.12]
Garden	0.04	0.15	0.27	0.78	[-0.25, 0.34]
Gas	-0.21	0.05	-4.44	0.00	[-0.3, -0.12]
Girder Bridge	0.31	0.15	2.02	0.04	[0.01, 0.61]
Grass	-0.06	0.05	-1.36	0.17	[-0.15, 0.03]
Grass Family	0.10	0.09	1.09	0.28	[-0.08, 0.27]
Grassland	0.08	0.08	1.04	0.30	[-0.08, 0.24]

Groundcover	0.03	0.08	0.36	0.72	[-0.13, 0.19]
Headquarters	0.01	0.11	0.09	0.93	[-0.21, 0.23]
Highland	0.32	0.14	2.30	0.02	[0.05, 0.59]
Highway	-0.24	0.11	-2.11	0.04	[-0.47, -0.02]
Hill	0.12	0.09	1.25	0.21	[-0.07, 0.3]
Home	0.03	0.10	0.31	0.75	[-0.16, 0.23]
Horizon	0.19	0.07	2.86	0.00	[0.06, 0.32]
House	0.15	0.04	4.22	0.00	[0.08, 0.22]
Human Settlement	0.13	0.09	1.50	0.13	[-0.04, 0.29]
Industry	0.30	0.18	1.69	0.09	[-0.05, 0.65]
Infrastructure	-0.03	0.05	-0.59	0.56	[-0.13, 0.07]
Intercept	2.59	0.12	20.96	0.00	[2.35, 2.83]
Lacustrine Plain	0.04	0.14	0.33	0.74	[-0.22, 0.31]
Lake	0.58	0.11	5.09	0.00	[0.36, 0.8]
Land Lot	-0.24	0.04	-5.46	0.00	[-0.33, -0.16]
Land Vehicle	0.07	0.06	1.16	0.25	[-0.05, 0.2]
Landmark	0.08	0.09	0.90	0.37	[-0.09, 0.25]
Landscape	0.03	0.04	0.65	0.52	[-0.06, 0.11]
Landscaping	0.01	0.16	0.06	0.95	[-0.3, 0.32]
Lane	0.09	0.06	1.54	0.12	[-0.02, 0.2]
Lawn	0.09	0.10	0.91	0.36	[-0.1, 0.28]
Leisure	-0.04	0.09	-0.48	0.63	[-0.21, 0.13]
Lighting	0.08	0.14	0.61	0.54	[-0.18, 0.35]
Line	-0.18	0.12	-1.44	0.15	[-0.42, 0.06]
Machine	-0.21	0.13	-1.60	0.11	[-0.47, 0.05]
Manor House	0.35	0.13	2.61	0.01	[0.09, 0.61]
Meadow	0.11	0.09	1.19	0.23	[-0.07, 0.28]
Metropolis	0.06	0.11	0.53	0.60	[-0.16, 0.29]
Metropolitan Area	-0.08	0.08	-1.05	0.29	[-0.24, 0.07]
Mixed Use	-0.05	0.05	-0.86	0.39	[-0.15, 0.06]
Mode Otransport	-0.03	0.06	-0.57	0.57	[-0.15, 0.08]
Monochrome	0.37	0.13	2.85	0.00	[0.11, 0.62]
Monument	0.35	0.16	2.16	0.03	[0.03, 0.66]
Morning	0.00	0.10	-0.01	0.99	[-0.2, 0.19]
Motor Vehicle	-0.16	0.05	-3.39	0.00	[-0.26, -0.07]
Mountain	1.16	0.16	7.15	0.00	[0.84, 1.48]
Natural Environment	0.03	0.12	0.23	0.82	[-0.21, 0.26]
Natural Landscape	0.33	0.06	5.11	0.00	[0.2, 0.45]
Neighbourhood	0.06	0.04	1.27	0.20	[-0.03, 0.14]
Nonbuilding Structure	-0.12	0.09	-1.37	0.17	[-0.29, 0.05]
Overhead Power Line	-0.21	0.08	-2.56	0.01	[-0.37, -0.05]
Overpass	-0.25	0.19	-1.30	0.19	[-0.62, 0.12]
Parking	-0.07	0.06	-1.18	0.24	[-0.18, 0.05]
Parking Lot	-0.21	0.11	-1.88	0.06	[-0.43, 0.01]
Passenger Car	-0.27	0.17	-1.58	0.11	[-0.61, 0.07]
Pasture	-0.07	0.10	-0.72	0.47	[-0.27, 0.12]
Pedestrian	0.01	0.10	0.11	0.91	[-0.19, 0.21]
Personal Luxury Car	0.02	0.12	0.16	0.87	[-0.22, 0.25]

Place Oworship	-0.18	0.22	-0.85	0.39	[-0.61, 0.24]
Plain	0.08	0.08	0.91	0.36	[-0.09, 0.24]
Plant	0.07	0.04	1.73	0.08	[-0.01, 0.14]
Plant Community	0.36	0.14	2.49	0.01	[0.08, 0.64]
Pole	0.01	0.06	0.09	0.93	[-0.1, 0.12]
Pollution	-0.14	0.16	-0.86	0.39	[-0.46, 0.18]
Power Station	-0.32	0.20	-1.56	0.12	[-0.71, 0.08]
Prairie	0.12	0.10	1.28	0.20	[-0.07, 0.31]
Property	0.01	0.06	0.26	0.79	[-0.1, 0.12]
Public Space	-0.09	0.08	-1.11	0.27	[-0.25, 0.07]
Public Transport	0.04	0.12	0.34	0.74	[-0.2, 0.28]
Public Utility	0.01	0.06	0.12	0.90	[-0.11, 0.13]
Railway	0.10	0.12	0.81	0.42	[-0.14, 0.33]
Real Estate	-0.15	0.05	-2.84	0.00	[-0.25, -0.05]
Recreation	0.10	0.11	0.90	0.37	[-0.12, 0.31]
Reflection	-0.08	0.13	-0.58	0.56	[-0.33, 0.18]
Reservoir	-0.37	0.13	-2.96	0.00	[-0.62, -0.13]
Residential Area	-0.05	0.04	-1.18	0.24	[-0.12, 0.03]
Retail	-0.18	0.13	-1.43	0.15	[-0.43, 0.07]
Riparian Zone	0.30	0.16	1.90	0.06	[-0.01, 0.61]
Road	-0.01	0.04	-0.15	0.88	[-0.08, 0.07]
Road Surface	0.12	0.05	2.45	0.01	[0.02, 0.21]
Rock	0.47	0.17	2.84	0.00	[0.15, 0.79]
Rolling	-0.15	0.08	-1.99	0.05	[-0.3, 0]
Rural Area	0.07	0.05	1.45	0.15	[-0.02, 0.15]
Shade	-0.01	0.07	-0.14	0.89	[-0.14, 0.12]
Shoulder	-0.15	0.12	-1.30	0.19	[-0.38, 0.08]
Shrub	0.02	0.07	0.28	0.78	[-0.12, 0.17]
Sidewalk	-0.07	0.05	-1.50	0.13	[-0.17, 0.02]
Sky	0.02	0.08	0.27	0.79	[-0.13, 0.18]
Slope	0.02	0.07	0.33	0.74	[-0.12, 0.17]
Snow	0.44	0.12	3.62	0.00	[0.2, 0.68]
Soil	-0.15	0.08	-1.96	0.05	[-0.3, 0]
Sport Venue	-0.22	0.17	-1.28	0.20	[-0.55, 0.12]
Stadium	-0.20	0.19	-1.09	0.28	[-0.57, 0.16]
Street	0.09	0.05	1.72	0.08	[-0.01, 0.18]
Street Light	-0.09	0.04	-2.53	0.01	[-0.16, -0.02]
Suburb	0.00	0.05	0.08	0.93	[-0.1, 0.11]
Sunlight	0.19	0.13	1.44	0.15	[-0.07, 0.45]
Tar	-0.16	0.05	-3.22	0.00	[-0.25, -0.06]
Thoroughfare	0.05	0.04	1.19	0.23	[-0.03, 0.14]
Tints And Shades	0.09	0.06	1.41	0.16	[-0.04, 0.22]
Tire	-0.02	0.05	-0.38	0.70	[-0.12, 0.08]
Tower	0.17	0.09	1.95	0.05	[0, 0.34]
Tower Block	-0.26	0.12	-2.15	0.03	[-0.5, -0.02]
Trail	0.14	0.16	0.87	0.39	[-0.18, 0.47]
Train	0.34	0.16	2.18	0.03	[0.03, 0.65]
Train Station	0.07	0.16	0.43	0.67	[-0.25, 0.38]

Transmission Tower	-0.36	0.19	-1.93	0.05	[-0.73, 0.01]
Transport Hub	-0.11	0.13	-0.80	0.43	[-0.37, 0.16]
Travel	-0.07	0.11	-0.64	0.52	[-0.29, 0.15]
Tree	0.01	0.04	0.32	0.75	[-0.06, 0.08]
Truck	-0.11	0.09	-1.31	0.19	[-0.28, 0.06]
Trunk	0.43	0.14	2.98	0.00	[0.15, 0.71]
Twig	-0.01	0.11	-0.10	0.92	[-0.23, 0.21]
Urban Area	-0.07	0.08	-0.92	0.36	[-0.23, 0.08]
Urban Design	-0.07	0.04	-1.78	0.07	[-0.15, 0.01]
Van	0.05	0.14	0.38	0.71	[-0.23, 0.33]
Vegetation	0.07	0.12	0.57	0.57	[-0.17, 0.3]
Vehicle	-0.04	0.05	-0.77	0.44	[-0.15, 0.06]
Vehicle Registration Plate	-0.04	0.06	-0.76	0.45	[-0.15, 0.07]
Walkway	0.25	0.14	1.83	0.07	[-0.02, 0.52]
Water	0.19	0.09	2.15	0.03	[0.02, 0.36]
Water Resources	0.01	0.13	0.11	0.91	[-0.24, 0.27]
Water Transportation	-0.30	0.23	-1.31	0.19	[-0.76, 0.15]
Watercourse	0.40	0.12	3.30	0.00	[0.16, 0.64]
Waterway	-0.17	0.11	-1.65	0.10	[-0.38, 0.03]
Wetland	-0.26	0.16	-1.66	0.10	[-0.56, 0.05]
Winter	-0.17	0.10	-1.63	0.10	[-0.37, 0.03]
Wood	0.00	0.06	-0.03	0.97	[-0.11, 0.11]
Woody Plant	0.13	0.11	1.18	0.24	[-0.09, 0.36]

6 Propensity Score Matching

We use the R package *MatchIt* to conduct propensity score matching to estimate the effect of the presence of a listed building; this creates covariate balance between photographs were a listed building is present and those without a listed building, thus accounting for confounding due to the Google Vision covariates. We used full matching on the propensity score. Scores were estimated using a generalized linear model. After matching, all absolute standardised mean differences for the covariates were below 0.1, except that for `chapel` which had an absolute standardised mean difference of 0.16 and `font` with 0.11. Full matching uses all treated and all control units, so no units were discarded by the matching.

We repeated our main analysis using the matched data. The coefficient on listed buildings was not significantly different from the unmatched analysis ($\beta = 0.521, CI = [0.433, 0.609], p < 0.001, N = 3,843$).

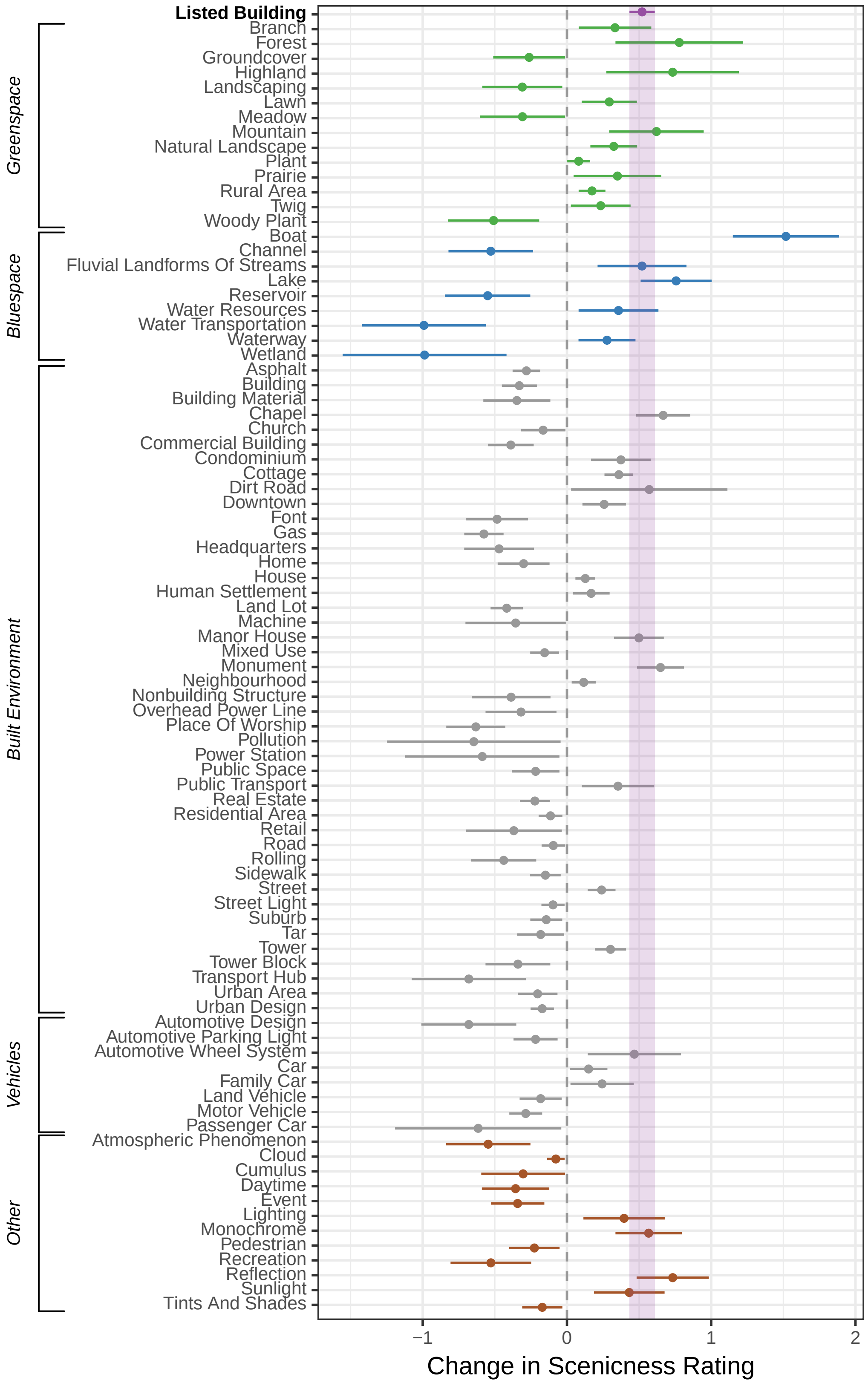
Listed Building
Branch
Forest
Groundcover
Highland
Landscaping
Lawn
Meadow
Mountain
Natural Landscape
Plant
Prairie
Rural Area
Twig
Woody Plant
Boat
Channel
Fluvial Landforms Of Streams
Lake
Reservoir
Water Resources
Water Transportation
Waterway
Wetland
Asphalt
Building
Building Material
Chapel
Church
Commercial Building
Condominium
Cottage
Dirt Road
Downtown
Font
Gas
Headquarters
Home
House
Human Settlement
Land Lot
Machine
Manor House
Mixed Use
Monument
Neighbourhood
Nonbuilding Structure
Overhead Power Line
Place Of Worship
Pollution
Power Station
Public Space
Public Transport
Real Estate
Residential Area
Retail
Road
Rolling
Sidewalk
Street
Street Light
Suburb
Tar
Tower
Tower Block
Transport Hub
Urban Area
Urban Design
Automotive Design
Automotive Parking Light
Automotive Wheel System
Car
Family Car
Land Vehicle
Motor Vehicle
Passenger Car
Atmospheric Phenomenon
Cloud
Cumulus
Daytime
Event
Lighting
Monochrome
Pedestrian
Recreation
Reflection
Sunlight
Tints And Shades
Greenspace
Bluespace
Built Environment
Vehicles
Other
−1
0
1
2
Change in Scenicness Rating

Table S179: Complete regression results.

term	estimate	std.error	statistic	p.value	conf
Listed Building	0.52	0.04	11.64	0.00	[0.43, 0.61]
Agriculture	-0.11	0.16	-0.64	0.52	[-0.43, 0.22]
Apartment	0.06	0.07	0.85	0.40	[-0.08, 0.19]
Asphalt	-0.28	0.05	-5.74	0.00	[-0.38, -0.19]
Atmospheric Phenomenon	-0.55	0.15	-3.65	0.00	[-0.84, -0.25]
Automotive Design	-0.68	0.17	-4.05	0.00	[-1.01, -0.35]
Automotive Exterior	-0.08	0.07	-1.07	0.28	[-0.22, 0.06]
Automotive Lighting	-0.11	0.07	-1.51	0.13	[-0.25, 0.03]
Automotive Parking Light	-0.22	0.08	-2.79	0.01	[-0.37, -0.06]
Automotive Tail Brake Light	0.12	0.14	0.83	0.41	[-0.16, 0.4]
Automotive Tire	-0.07	0.29	-0.23	0.82	[-0.63, 0.5]
Automotive Wheel System	0.47	0.16	2.84	0.00	[0.14, 0.79]
Biome	0.25	0.18	1.37	0.17	[-0.11, 0.6]
Boat	1.52	0.19	8.09	0.00	[1.15, 1.89]
Body Owater	-0.20	0.15	-1.27	0.21	[-0.5, 0.11]
Branch	0.33	0.13	2.60	0.01	[0.08, 0.58]
Bridge	0.15	0.11	1.38	0.17	[-0.06, 0.37]
Building	-0.33	0.06	-5.33	0.00	[-0.45, -0.21]
Building Material	-0.35	0.12	-2.93	0.00	[-0.58, -0.12]
Bus	0.03	0.11	0.30	0.76	[-0.18, 0.24]
Canal	0.21	0.15	1.38	0.17	[-0.09, 0.5]
Car	0.15	0.07	2.25	0.02	[0.02, 0.28]
Channel	-0.53	0.15	-3.53	0.00	[-0.82, -0.24]
Chapel	0.67	0.10	6.97	0.00	[0.48, 0.85]
Church	-0.17	0.08	-2.10	0.04	[-0.32, -0.01]
City	-0.07	0.05	-1.59	0.11	[-0.16, 0.02]
Cloud	-0.08	0.03	-2.48	0.01	[-0.14, -0.02]
Commercial Building	-0.39	0.08	-4.80	0.00	[-0.55, -0.23]
Concrete Bridge	-0.27	0.26	-1.03	0.30	[-0.79, 0.25]
Condominium	0.37	0.11	3.54	0.00	[0.17, 0.58]
Cottage	0.36	0.05	7.07	0.00	[0.26, 0.46]
Cumulus	-0.30	0.15	-2.05	0.04	[-0.59, -0.01]
Daytime	-0.36	0.12	-2.99	0.00	[-0.59, -0.12]
Dirt Road	0.57	0.28	2.06	0.04	[0.03, 1.11]
Downtown	0.26	0.08	3.36	0.00	[0.11, 0.41]
Driveway	-0.28	0.22	-1.30	0.19	[-0.71, 0.14]
Dusk	0.05	0.13	0.40	0.69	[-0.21, 0.32]
Ecoregion	0.00	0.23	0.00	1.00	[-0.45, 0.45]
Electrical Supply	0.06	0.27	0.23	0.81	[-0.47, 0.6]
Electricity	0.01	0.08	0.17	0.86	[-0.14, 0.17]
Engineering	0.08	0.20	0.41	0.68	[-0.3, 0.46]
Evening	-0.03	0.11	-0.30	0.77	[-0.25, 0.18]
Event	-0.34	0.09	-3.62	0.00	[-0.53, -0.16]
Family Car	0.24	0.11	2.18	0.03	[0.02, 0.46]

Field	0.28	0.14	1.95	0.05	[0, 0.56]
Fixture	-0.04	0.06	-0.68	0.49	[-0.16, 0.08]
Flower	0.21	0.13	1.62	0.10	[-0.04, 0.46]
Fluvial Landforms Ostreams	0.52	0.16	3.31	0.00	[0.21, 0.83]
Font	-0.48	0.11	-4.43	0.00	[-0.7, -0.27]
Forest	0.78	0.23	3.45	0.00	[0.34, 1.22]
Freeway	-0.05	1.04	-0.04	0.97	[-2.09, 2]
Garden	0.21	0.14	1.51	0.13	[-0.06, 0.48]
Gas	-0.58	0.07	-8.28	0.00	[-0.71, -0.44]
Girder Bridge	-0.15	0.27	-0.54	0.59	[-0.68, 0.39]
Grass	0.03	0.05	0.60	0.55	[-0.07, 0.13]
Grass Family	0.09	0.13	0.69	0.49	[-0.16, 0.33]
Grassland	-0.04	0.13	-0.30	0.77	[-0.3, 0.22]
Groundcover	-0.26	0.13	-2.06	0.04	[-0.51, -0.01]
Headquarters	-0.47	0.12	-3.82	0.00	[-0.71, -0.23]
Highland	0.73	0.23	3.13	0.00	[0.27, 1.19]
Highway	-0.20	0.28	-0.70	0.49	[-0.75, 0.36]
Hill	0.21	0.12	1.81	0.07	[-0.02, 0.44]
Home	-0.30	0.09	-3.28	0.00	[-0.48, -0.12]
Horizon	-0.20	0.11	-1.73	0.08	[-0.42, 0.03]
House	0.13	0.04	3.63	0.00	[0.06, 0.2]
Human Settlement	0.17	0.07	2.58	0.01	[0.04, 0.3]
Industry	0.43	0.28	1.53	0.13	[-0.12, 0.98]
Infrastructure	-0.07	0.05	-1.21	0.23	[-0.17, 0.04]
Intercept	3.05	0.12	24.99	0.00	[2.82, 3.29]
Lacustrine Plain	-0.30	0.19	-1.54	0.12	[-0.68, 0.08]
Lake	0.76	0.13	6.03	0.00	[0.51, 1]
Land Lot	-0.42	0.06	-7.31	0.00	[-0.53, -0.31]
Land Vehicle	-0.18	0.07	-2.45	0.01	[-0.33, -0.04]
Landmark	-0.03	0.05	-0.47	0.64	[-0.13, 0.08]
Landscape	0.07	0.05	1.30	0.19	[-0.03, 0.17]
Landscaping	-0.31	0.14	-2.19	0.03	[-0.59, -0.03]
Lane	0.07	0.07	1.05	0.30	[-0.06, 0.2]
Lawn	0.29	0.10	3.01	0.00	[0.1, 0.48]
Leisure	-0.09	0.09	-0.94	0.35	[-0.27, 0.1]
Lighting	0.40	0.14	2.76	0.01	[0.11, 0.68]
Line	-0.18	0.15	-1.18	0.24	[-0.48, 0.12]
Machine	-0.36	0.18	-2.01	0.05	[-0.7, -0.01]
Manor House	0.50	0.09	5.67	0.00	[0.33, 0.67]
Meadow	-0.31	0.15	-2.05	0.04	[-0.6, -0.01]
Metropolis	-0.08	0.09	-0.88	0.38	[-0.25, 0.1]
Metropolitan Area	0.06	0.07	0.83	0.40	[-0.07, 0.19]
Mixed Use	-0.15	0.05	-3.03	0.00	[-0.26, -0.05]
Mode Otransport	-0.01	0.07	-0.12	0.90	[-0.14, 0.12]
Monochrome	0.57	0.12	4.83	0.00	[0.34, 0.8]
Monument	0.65	0.08	7.82	0.00	[0.49, 0.81]
Morning	-0.01	0.11	-0.07	0.94	[-0.23, 0.22]
Motor Vehicle	-0.29	0.06	-4.92	0.00	[-0.4, -0.17]

Mountain	0.62	0.17	3.72	0.00	[0.29, 0.95]
Natural Environment	-0.25	0.23	-1.09	0.27	[-0.7, 0.2]
Natural Landscape	0.32	0.08	3.92	0.00	[0.16, 0.49]
Neighbourhood	0.12	0.04	2.74	0.01	[0.03, 0.2]
Nonbuilding Structure	-0.39	0.14	-2.78	0.01	[-0.66, -0.11]
Overhead Power Line	-0.32	0.13	-2.54	0.01	[-0.56, -0.07]
Overpass	-0.15	0.30	-0.51	0.61	[-0.73, 0.43]
Parking	0.02	0.08	0.31	0.75	[-0.13, 0.18]
Parking Lot	-0.10	0.84	-0.12	0.90	[-1.75, 1.54]
Passenger Car	-0.62	0.29	-2.09	0.04	[-1.19, -0.04]
Pasture	-0.19	0.13	-1.47	0.14	[-0.45, 0.06]
Pedestrian	-0.23	0.09	-2.53	0.01	[-0.4, -0.05]
Personal Luxury Car	-0.03	0.20	-0.13	0.90	[-0.43, 0.37]
Place Oworship	-0.63	0.10	-6.05	0.00	[-0.84, -0.43]
Plain	0.18	0.13	1.38	0.17	[-0.08, 0.45]
Plant	0.08	0.04	2.01	0.04	[0, 0.16]
Plant Community	0.50	1.09	0.46	0.65	[-1.64, 2.63]
Pole	-0.10	0.06	-1.64	0.10	[-0.22, 0.02]
Pollution	-0.65	0.31	-2.10	0.04	[-1.25, -0.04]
Power Station	-0.59	0.27	-2.15	0.03	[-1.12, -0.05]
Prairie	0.35	0.16	2.26	0.02	[0.05, 0.65]
Property	0.08	0.05	1.47	0.14	[-0.03, 0.18]
Public Space	-0.22	0.08	-2.58	0.01	[-0.38, -0.05]
Public Transport	0.35	0.13	2.77	0.01	[0.1, 0.6]
Public Utility	0.11	0.10	1.05	0.29	[-0.09, 0.3]
Railway	0.04	0.14	0.31	0.76	[-0.23, 0.32]
Real Estate	-0.22	0.05	-4.19	0.00	[-0.33, -0.12]
Recreation	-0.53	0.14	-3.70	0.00	[-0.81, -0.25]
Reflection	0.73	0.13	5.74	0.00	[0.48, 0.98]
Reservoir	-0.55	0.15	-3.65	0.00	[-0.85, -0.25]
Residential Area	-0.11	0.04	-2.72	0.01	[-0.2, -0.03]
Retail	-0.37	0.17	-2.17	0.03	[-0.7, -0.04]
Riparian Zone	-0.30	0.24	-1.24	0.21	[-0.78, 0.17]
Road	-0.09	0.04	-2.28	0.02	[-0.18, -0.01]
Road Surface	0.04	0.05	0.82	0.41	[-0.06, 0.14]
Rock	-0.06	0.17	-0.33	0.74	[-0.4, 0.28]
Rolling	-0.44	0.11	-3.82	0.00	[-0.66, -0.21]
Rural Area	0.17	0.05	3.67	0.00	[0.08, 0.27]
Shade	0.09	0.10	0.90	0.37	[-0.11, 0.29]
Shoulder	-0.16	0.92	-0.18	0.86	[-1.96, 1.63]
Shrub	0.11	0.08	1.32	0.19	[-0.05, 0.27]
Sidewalk	-0.15	0.05	-2.76	0.01	[-0.26, -0.04]
Sky	-0.01	0.07	-0.11	0.91	[-0.15, 0.14]
Slope	-0.01	0.12	-0.10	0.92	[-0.26, 0.23]
Snow	0.19	0.11	1.73	0.08	[-0.03, 0.41]
Soil	-0.17	0.27	-0.63	0.53	[-0.7, 0.36]
Sport Venue	-0.41	0.37	-1.12	0.26	[-1.13, 0.31]
Stadium	-0.40	0.39	-1.03	0.30	[-1.17, 0.37]

Street	0.24	0.05	4.89	0.00	[0.14, 0.34]
Street Light	-0.10	0.04	-2.36	0.02	[-0.18, -0.02]
Suburb	-0.14	0.06	-2.54	0.01	[-0.25, -0.03]
Sunlight	0.43	0.12	3.46	0.00	[0.19, 0.68]
Tar	-0.18	0.08	-2.20	0.03	[-0.34, -0.02]
Thoroughfare	0.08	0.05	1.52	0.13	[-0.02, 0.18]
Tints And Shades	-0.17	0.07	-2.41	0.02	[-0.31, -0.03]
Tire	0.00	0.05	0.08	0.93	[-0.1, 0.11]
Tower	0.30	0.05	5.51	0.00	[0.19, 0.41]
Tower Block	-0.34	0.11	-2.97	0.00	[-0.56, -0.12]
Trail	-0.03	1.20	-0.03	0.98	[-2.38, 2.32]
Train	0.19	0.16	1.16	0.25	[-0.13, 0.5]
Train Station	0.27	0.20	1.36	0.17	[-0.12, 0.66]
Transmission Tower	-0.37	1.27	-0.29	0.77	[-2.86, 2.12]
Transport Hub	-0.68	0.20	-3.37	0.00	[-1.08, -0.28]
Travel	-0.07	0.08	-0.89	0.37	[-0.23, 0.09]
Tree	0.01	0.04	0.33	0.74	[-0.06, 0.09]
Truck	-0.05	0.13	-0.42	0.68	[-0.31, 0.2]
Trunk	0.29	0.17	1.71	0.09	[-0.04, 0.63]
Twig	0.23	0.11	2.21	0.03	[0.03, 0.44]
Urban Area	-0.20	0.07	-2.89	0.00	[-0.34, -0.07]
Urban Design	-0.17	0.04	-4.16	0.00	[-0.25, -0.09]
Van	0.07	0.19	0.36	0.72	[-0.3, 0.44]
Vegetation	0.22	0.14	1.54	0.12	[-0.06, 0.49]
Vehicle	0.06	0.06	0.99	0.32	[-0.06, 0.18]
Vehicle Registration Plate	-0.03	0.06	-0.57	0.57	[-0.15, 0.08]
Walkway	-0.02	0.21	-0.12	0.91	[-0.43, 0.38]
Water	-0.18	0.11	-1.65	0.10	[-0.39, 0.03]
Water Resources	0.36	0.14	2.53	0.01	[0.08, 0.63]
Water Transportation	-0.99	0.22	-4.52	0.00	[-1.42, -0.56]
Watercourse	0.21	0.14	1.46	0.14	[-0.07, 0.48]
Waterway	0.28	0.10	2.75	0.01	[0.08, 0.48]
Wetland	-0.99	0.29	-3.41	0.00	[-1.56, -0.42]
Winter	-0.02	0.09	-0.26	0.79	[-0.2, 0.15]
Wood	-0.05	0.05	-0.95	0.34	[-0.16, 0.05]
Woody Plant	-0.51	0.16	-3.15	0.00	[-0.83, -0.19]
